# Impact of creatine supplementation on inflammation: evidence from a systematic review and meta-analysis of randomized double-blind placebo trials

**DOI:** 10.3389/fimmu.2026.1743603

**Published:** 2026-02-19

**Authors:** Kell Mazzini Ribeiro de Camargo, Alejandro Bruna-Mejías, Juan José Valenzuela-Fuenzalida, Luana A. Gonzaga, Sandra Maria Barbalho, Alexandre L. Barroca, Andrey A. Porto, Rodrigo D. Raimundo, Luiz Carlos de Abreu, Vitor E. Valenti

**Affiliations:** 1 Systematic Reviews Center for Cardiovascular and Metabolic Health, School of Philosophy and Sciences, São Paulo State University, Marília, SP, Brazil; 2 Escuela de Medicina, Facultad de Medicina, Universidad Andres Bello, Santiago, Chile; 3 Departamento de Ciencias y Geografia, Facultad de Ciencias Naturales y Exactas, Universidad de Playa Ancha, Valparaiso, Chile; 4 Departamento de Ciencias Químicas y Biológicas, Facultad de Ciencias de la Salud, Universidad Bernardo O’Higgins, Santiago, Chile; 5 Postgraduate Program in Structural and Functional Interactions in Rehabilitation, School of Medicine, Universidade de Marília (UNIMAR), Marília, São Paulo, Brazil; 6 Department of Biochemistry and Nutrition, School of Food and Technology of Marilia (FATEC), Marília, São Paulo, Brazil; 7 UNIMAR Charity Hospital, Universidade de Marilia (UNIMAR), Marília, São Paulo, Brazil; 8 Laboratorio de Delineamento de Estudos e Escrita Científica, Centro Universitario FMABC, Santo Andre, SP, Brazil; 9 Department of Public Health, University of Limerick, Limerick, IE, Ireland; 10 Department of Nutrition, Federal University of Vitoria, Vitoria, ES, Brazil

**Keywords:** creatine, CRP, cytokines, IL-6, inflammation, meta-analysis, supplementation

## Abstract

**Introduction:**

Creatine supplementation is widely recognized for its ergogenic effects on strength and body composition. Recent studies have explored its potential anti-inflammatory properties, particularly in exercise-induced stress and aging-related chronic inflammation. However, results across randomized trials remain inconsistent. This systematic review and meta-analysis aimed to assess the effects of creatine supplementation on inflammatory biomarkers in human populations.

**Methods:**

A systematic review and meta-analysis were conducted following PRISMA 2020 guidelines and registered in PROSPERO (CRD420251027784). Eight randomized controlled trials were included, evaluating creatine supplementation (various dosages and durations) versus placebo in healthy individuals, athletes, and clinical populations. The primary outcomes were inflammatory markers, including C-reactive protein (CRP), interleukin-6 (IL-6), IL-1β, TNF-α, and prostaglandin E_2_. Data extraction and risk of bias assessments were performed by two independent reviewers. The certainty of evidence was rated using the GRADE framework.

**Results:**

Pooled analysis showed no significant acute effects of creatine on CRP (SMD = 0.32; 95% CI: -0.29 to 0.94; p = 0.30; I² = 28%). Chronic effects of creatine on CRP (SMD = -0.11; 95% CI: -0.69 to 0.48; p = 0.73; I² = 0%) and IL-6 (SMD = -0.06; 95% CI: -0.64 to 0.53; p = 0.84; I² = 0%) were also no significant. The certainty of evidence was rated as moderate for all outcomes. Risk of bias varied, with missing outcome data being the most frequent limitation.

**Conclusion:**

Creatine supplementation does not significantly reduce inflammatory biomarkers in humans based on current evidence. Although certain benefits were observed under intense endurance conditions, results remain inconsistent across populations. Future well-powered trials with standardized protocols are needed to clarify creatine’s role in modulating inflammation.

**Systematic review registration:**

https://www.crd.york.ac.uk/prospero/, identifier CRD420251027784.

## Introduction

Inflammation plays a pivotal role in both physiological adaptation and pathological processes. Inflammation is the response of living vascularized tissue to injury and can be triggered by microbial infections, physical agents, chemical substances, necrotic tissue, or immunological reactions. The goal of inflammation is to contain and isolate the injury, destroy invading microorganisms, and inactivate toxins, as well as prepare the tissue for healing and repair (1).

Conversely, chronic low-grade inflammation is defined as a two to four-fold elevation in circulating pro-inflammatory markers, including C-reactive protein (CRP), tumor necrosis factor-α (TNF-α), and interleukin-6 (IL-6). This persistent inflammatory state is strongly associated with the aging process and contributes mechanistically to sarcopenia—the age-related reduction in muscle mass and strength (2). Beyond aging, inflammation drives the progression of several chronic diseases: elevated CRP and TNF-α concentrations are associated with increased total knee pain in osteoarthritis, and chronic inflammation can lead to metabolic disorders such as Type 2 Diabetes Mellitus and cardiovascular diseases. However, the increase in IL-6 following exercise may play a beneficial role by mobilizing substrates for energy and enhancing insulin sensitivity, potentially protecting against disorders like Type 2 Diabetes Mellitus by inhibiting TNF-α production (3). The clinical and performance implications of managing inflammation are critical, as exercise-induced muscle trauma results in pain, delayed onset muscle soreness (DOMS), reduced range of motion, and prolonged muscle strength loss, negatively impacting subsequent athletic performance (4, 5). With this in mind, pharmacological and non-pharmacological interventions have gained attention for improving quality of life by enhancing cardiovascular, metabolic, and inflammatory parameters (6–9).

In this context, creatine is a widely popular dietary supplement utilized as an ergogenic aid. Its well-established performance-enhancing effects are rooted in its fundamental role as a temporal and spatial energy buffer. Supplementation reliably increases total muscle creatine concentration, enhancing phosphocreatine (PCr) availability to facilitate ATP resynthesis during high-intensity exercise (10). When combined with resistance training, creatine reliably promotes strength and fat-free mass gains in diverse populations, including older adults (11). While creatine is widely recognized for its performance-enhancing properties, recent studies suggest it may also modulate inflammatory responses, especially following intense physical activity. Creatine is reported to be anti-inflammatory in nature, helping to maintain muscle integrity and attenuating inflammatory markers after strenuous exercise sessions (4, 12).

The precise mechanisms underlying creatine’s anti-inflammatory and cytoprotective effects remain to be definitively determined. However, several mechanisms have been proposed. One theory involves osmotic effects and cellular swelling; creatine increases intracellular water content (13).

Current evidence regarding creatine’s potential anti-inflammatory properties remains inconsistent, highlighting significant knowledge gaps across different populations and protocols. Positive effects in athletes subject to high physiological stress have been frequently reported. For instance, creatine supplementation for five days prior to a half-ironman competition significantly reduced the exercise-induced increase in plasma levels of pro-inflammatory cytokines, including TNF-α, interferon-alpha (IFN-α), and interleukin-beta (IL-1β), as well as Prostaglandin E2 (PGE2), 24 and 48 hours post-competition (4, 12). Similarly, creatine supplementation attenuated the post-race increase in plasma TNF-α (by 33.7%) and PGE2 (by 60.9%), and abolished the increase in lactate dehydrogenase (LDH) activity following a strenuous 30 km race in marathon runners (4, 12).

However, null findings have limited the generalization of these effects to other populations or exercise types. In studies focused on chronic, low-grade inflammation, 12 weeks of creatine supplementation yielded no effect on inflammatory biomarkers (CRP, IL-1β, IL-6, TNF-α) in patients with mild to moderate knee osteoarthritis (14). Moreover, combining creatine supplementation (5 g/day for 12 weeks) with resistance training in community-dwelling older adults failed to provide additional benefits on systemic inflammation markers such as IL-6, interleukin 10 (IL-10), and CRP, compared to training with placebo (15). Creatine also failed to reduce muscle damage (assessed via strength, range of motion, soreness, and elevated creatine kinase activity) or enhance recovery following a resistance exercise challenge designed to be hypoxic in trained men (5). Several methodological limitations contribute to these discrepancies, as many studies are small-scale; for example, the study investigating creatine in osteoarthritis included only 18 participants (14) and the half-ironman study included only 11 triathletes (4, 5). Furthermore, the inflammatory markers assessed vary widely across trials, ranging from cytokines (IL-6, TNF-α, IL-1β, IFN-α) and pain mediators (PGE2) to muscle damage proxies (creatine kinase-CK, lactate dehydrogenase-LDH, CRP). Despite growing interest, current evidence on creatine’s impact on inflammation remains fragmented, with no consensus across populations or protocols.

The inconsistencies observed between trials that investigated acute exercise-induced inflammation (4, 12) and those addressing chronic inflammation (14, 15) highlight a critical need for synthesizing the accumulated data (14). A systematic synthesis and meta-analysis is therefore warranted to rigorously aggregate findings from randomized controlled trials. Such an approach will permit a detailed evaluation of acute versus chronic creatine effects across different physiological states and population subsets (5). Crucially, this effort must focus on objective inflammatory markers measured in human participants to clarify the clinical and physiological relevance of creatine’s purported anti-inflammatory effects. A systematic review with meta-analysis is warranted to clarify whether creatine exerts clinically meaningful anti-inflammatory effects in humans, particularly in the context of exercise-induced and chronic inflammation.

Therefore, the present study aimed to systematically review and meta-analyze randomized controlled trials investigating the effects of creatine supplementation on inflammatory biomarkers in humans. We hypothesized that creatine would reduce levels of key inflammatory markers, particularly in response to acute exercise-induced stress (4, 12).

## Methods

### Protocol and registration

The review followed the guidelines outlined in the Preferred Reporting Items for Systematic Reviews and Meta-Analyses (PRISMA) (16) and has been formally registered in the PROSPERO database (CRD420251027784).

### Eligibility criteria

The selected studies were sourced from peer-reviewed journals and were published from the inception of each database up to December 2025. The eligibility criteria were established based on the PICOS framework (Population, Intervention, Comparison, Outcomes, and Study Design), encompassing:

(P) Studies involving human participants of any age, sex, or health status (e.g., healthy individuals, athletes, or patients with clinical conditions). Exclusion criteria: Studies involving animals or *in vitro* models;
(I) Studies that administered creatine supplementation, regardless of dosage, duration, or form (e.g., creatine monohydrate, creatine ethyl ester), either alone or combined with exercise or other interventions. Exclusion criteria: Studies using multi-ingredient supplements where the independent effect of creatine cannot be determined;
(C) For comparison groups, we included studies that evaluated subjects that received placebo;
(O) Primary outcomes: Studies that assessed inflammatory markers (e.g., CRP, interleukins such as IL-6, IL-1β, TNF-α, etc.). Secondary outcomes: Blood glucose, cholesterol and tryglicerides. Exclusion criteria: Studies without available data on inflammatory markers;
(S) We included studies with single or double-blind randomized controlled trials (RCTs) and crossover designs. This review is restricted to articles published in peer-reviewed journals, master’s theses and doctoral dissertations. We excluded conference abstracts, descriptive studies, case reports, editorials, and reviews.

### Information source, search strategy and study selection

The literature search was conducted in the EMBASE, LILACS, CINAHL, MEDLINE/PubMed (via the National Library of Medicine), Cochrane Library, Scopus, and Web of Science databases. The search strategy included the following terms: “Creatine Supplement” OR “Creatine monohydrate supplementation” OR “Creatine supplementation” AND “Inflammation” OR “Cytokine” OR “Interleukin” (full strategies available in the **Supplementary File**).

All retrieved records were exported to Rayyan QCRI (Qatar Computing Research Institute, Qatar) for automatic duplicate removal. Title and abstract screening was carried out in Rayyan by at least two independent reviewers, followed by full-text screening. In cases of disagreement, a third reviewer adjudicated the final decision. After selecting the eligible studies, the research team collectively evaluated whether a meta-analysis was feasible.

### Data collection and data extraction

Information on authorship, study design, participant characteristics, intervention details, and exercise protocols was extracted and summarized in a structured table. Missing information was requested directly from corresponding authors. When no response was received, numerical data presented only in figures were extracted using WebPlotDigitizer^®^. Data were expressed as means and standard deviations (SD). When studies reported standard error (SE) or confidence intervals (CI), these values were converted to SD.

### Data items

We extracted data related to inflammatory biomarkers to compare outcomes between intervention and control groups. Additional information regarding participant characteristics, intervention protocols, and funding sources were obtained from the included studies. Variables that were unclear or not reported were excluded from further analysis.

### Assessment of the risk of bias in individual studies and across studies

Risk of bias was assessed using the Cochrane Risk of Bias 2.0 tool (17) in Review Manager (RevMan 5.4.1). The tool evaluates six domains:

1. Randomization process.
2. Deviations from intended interventions.
3. Missing outcome data.
4. Outcome measurement.
5. Selection of reported results.
6. Overall bias.

Each domain was rated as “low risk,” “some concerns,” or “high risk.” Two independent reviewers completed the assessment, and disagreements were resolved by consulting a third reviewer. All assessors completed prior training in risk-of-bias evaluation. Potential sources of bias at the study and review level, such as publication bias and selective reporting, were also considered.

### Certainty assessment (levels of evidence)

The certainty of evidence was appraised using the GRADE (Grading of Recommendations, Assessment, Development and Evaluation) approach (18). Factors considered included study design, methodological quality, precision of estimates, and consistency across studies (19). The GRADEpro GDT v4^®^ software (McMaster University, Canada) was used to generate the Summary of Findings table.

### Qualitative analysis (systematic review)

Study characteristics and findings were described in text and tables, with emphasis on cardiovascular and inflammatory outcomes in both intervention and control conditions.

### Synthesis of results and summary measures

When at least two studies provided comparable data, a meta-analysis was conducted. Only post-intervention values were included. Heterogeneity was quantified using the I² statistic, interpreted as follows:

0–29%: negligible heterogeneity.
30–49%: moderate.
50–74%: substantial.
75–100%: considerable (20, 21).

If dispersion metrics (e.g., SD, 95% CI, SE, p-value) were not reported, SD of change scores was calculated when possible. Pooled effects were expressed as weighted mean difference (MD) with 95% confidence intervals. In brief, MDs were used when biomarkers were measured on comparable scales across studies, facilitating direct interpretability, whereas standard mean differences (SMDs) were used when studies employed different measurement scales or exhibited substantial variability in dispersion. The weighting method applied in all models was the inverse-variance approach, consistent with Cochrane recommendations. Statistical significance was set at p < 0.05. A random-effects model was applied due to expected methodological and clinical heterogeneity (22).

Sensitivity analyses were pre-planned to evaluate whether smaller trials exerted a disproportionate influence on the pooled effect estimates, particularly by examining changes in the overall effect size after sequential exclusion of individual studies or removal of the smallest trials. This strategy was intended to assess the robustness and stability of the meta-analytic findings. In addition, subgroup analyses were pre-specified according to creatine dosing regimen (acute loading vs. chronic supplementation) to explore potential sources of heterogeneity.

Acute Effects: Studies were classified under the acute effects analysis if the outcome measure reflected a transient physiological response to a single stressor. This classification included studies that met either or both of the following criteria:

-Acute Supplementation Protocol: The creatine intervention was short-term, typically defined as a loading phase (≤ 7 days of high-dose intake) with the outcome measured immediately thereafter.
-Acute Outcome Measurement: The outcome (e.g., inflammatory marker concentration) was measured immediately before, during, or within 72 hours following an acute exercise bout, regardless of the total supplementation duration. Crucially, any study that utilized a chronic supplementation protocol (≥ 4 weeks) but measured the outcome in response to an acute exercise challenge (i.e., immediate post-bout measurement) was consistently categorized under Acute Effects, as the purpose of the measurement was to capture the immediate, transient response to the acute stimulus.

Chronic Effects: Studies were exclusively classified under the Chronic Effects analysis if the outcome measure reflected a long-term physiological adaptation or baseline change. This required meeting both of the following criteria:

-Chronic Supplementation Protocol: The creatine intervention was long-term, typically ≤ 4 weeks, often involving a loading phase followed by a maintenance dose.
-Chronic Outcome Measurement: The primary outcome measure (e.g., resting levels of inflammatory markers, long-term training adaptations) was taken at the end of the entire supplementation and/or training protocol, with no acute exercise bout immediately preceding the measurement.

However, meaningful sensitivity or subgroup analyses require a minimum of two studies contributing data to the same outcome within each subgroup. The minimum number of trials necessary to conduct these analyses was not reached. Consequently, neither sensitivity analyses nor dose-based subgroup analyses could be performed for those outcomes. All statistical analyses were conducted using RevMan version 5.4.1.

## Results

### Study selection

**Figure 1:**
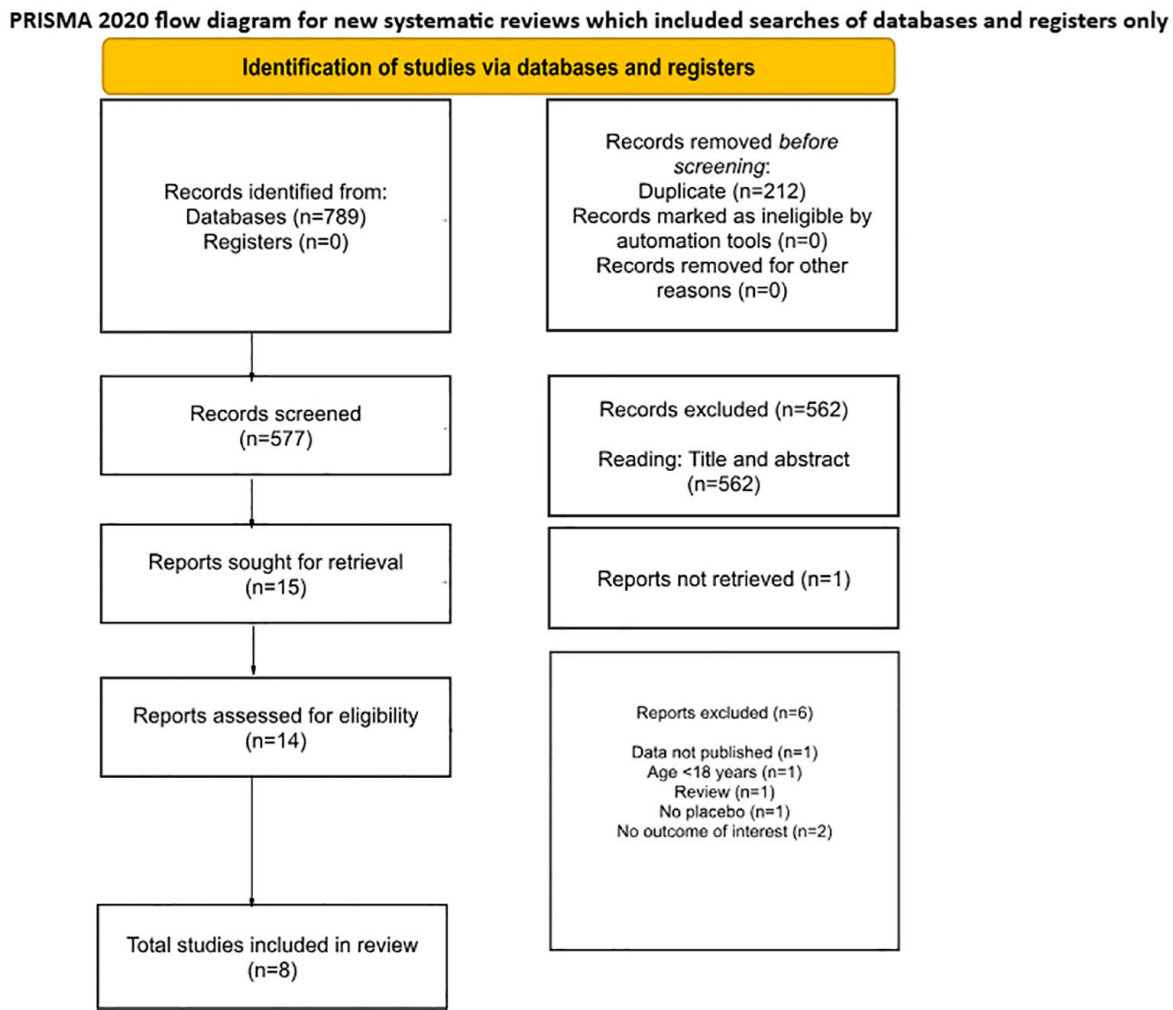
PRISMA 2020 flow diagram for new systematic reviews which included searches of databases and registers only.

A total of 789 records were identified through database searches. After removing 212 duplicates, 577 unique records were screened according to the inclusion criteria. Following the screening of titles and abstracts, 562 records were excluded. Fifteen studies were then selected for full-text retrieval, one of which could not be retrieved. The remaining 14 studies were assessed for eligibility through full-text reading. Six studies were excluded for the following reasons: data not published (n=1), population under 18 years of age (n=1), article type was a review (n=1), study had no placebo group (n=1), and no outcome of interest was reported (n=2). Consequently, eight studies were included in the final review. The search methods and study selection process were conducted in accordance with the PRISMA statement, as illustrated in **Figure 1**.

### Results of individual studies

This analysis focused on the impact of creatine supplementation on inflammatory markers. The reviewed studies demonstrated mixed effects depending on the population and experimental conditions (**Table 1**).

**Table 1:** Description of the characteristics of the study population of articles by author and year, sample, age (years), intervention, control and outcomes.

Author/ years	Study design	Sample	Age (years)	Intervention	Control	Outcomes	Funding
Bassit et al 2008 (4)	Randomized, double-blind, placebo-controlled trial.	11 male triathletes. Intervention group: n=5. Control group: n=6.	Mean age: 40.3 ± 2.18 years. Range: 34 to 56 years.	Dose: 20 grams of creatine monohydrate per day. Duration: 5 consecutive days prior to the competition. Form of administration: Two equal daily doses (10 a.m. and 4 p.m.), mixed with 50 g of maltodextrin powder and diluted in water.	20 grams of carbohydrate (maltodextrin) daily, prepared and flavored identically to the creatine solution.	Creatine supplementation significantly reduced Tumor Necrosis Factor-α (TNF-α), Interleukin-1β and Prostaglandin E2 (PGE2).	Yes.
Cornisha & Peeler 2018 (14)	Randomized, double-blind, placebo-controlled trial.	18 patients with mild to moderate knee osteoarthritis. Intervention group: n=9. Control group: n=9	Mean age: 57.1 ± 7.4 years. Range: 46.7 to 65.9 years.	Dose: 20 grams per day of creatine monohydrate for the first week (loading phase). This was administered as 5 grams, 4 times per day. For the remaining 11 weeks, participants consumed 5 grams per day of creatine monohydrate (maintenance phase). Duration: 12 consecutives weeks. Form of administration: oral supplementation. Creatine monohydrate (Creapure®) was supplemented to participants' regular diet.	The Placebo Group (n=9) received 20 grams per day of maltodextrin for the first week (4 x 5g/day), followed by 5 grams per day of maltodextrin for the remaining 11 weeks. The study was double-blind, ensuring neither participants nor investigators knew the supplement content.	No significant differences were found in inflammatory biomarkers (C-reactive protein, interleukin-1β, interleukin-6, s100 A8/A9, tumor necrosis factor-α) between the creatine and placebo groups after 12 weeks	Yes.
Deldicque et al 2008 (23)	Double-blind crossover study.	9 health young man. Intervention group: n=5. Control group: n=4.	Mean age: 21.7 ± 0.55 years. Range: Not reported.	Dose: 21 grams per day of oral creatine monohydrate, divided into three 7 grams doses. Duration: 5 days. Forms of administration: oral ingestion.	Maltodextrin, 21 grams per day (3 x 7g/day), administered orally during the same period as the creatine supplementation.	Creatine showed no modulatory effect on IL-6 expression.	Yes.
Oliveira et al 2020 (15)	Pilot randomized, double-blind, placebo-controlled trial. Also described as a randomized, double-blind, placebo-controlled, parallel-group clinical trial.	27 community-dwelling older adults completed the trial. Intervention group: n=13 Control group: n=14	Mean age: 67 ± 5 years (intervention group) and 67 ± 6 years (control group). Range: 60 to 80 years.	Dose: 5 grams per day of creatine monohydrate. Duration: 12 weeks. Forms of administration: oral ingestion. On training days, consumed immediately after sessions dissolved in a beverage with 100 g of lemon-flavored maltodextrin. On non-training days, consumed immediately after lunch dissolved in a liquid of their preference.	5 grams per day of maltodextrin, administered orally following the same protocol as the creatine group.	After 12 weeks, there were no differences between groups in any of the analyzed variables, including adiponectin, leptin, IL-6, IL-10, and CRP.	No.
Rawson et al 2007 (5)	Randomized, placebo-controlled, double-blind trial.	22 healthy, weight-trained men. Intervention group: n=11. Control group: n=11.	Mean age: 22.2 ± 1.3 years (Intervention group) and 22.1 ± 2.5 years (Control group). Range: 19 to 27 years.	Dose: Loading phase: 0.3 grams per kilogram body weight per day for 5 days. Maintenance phase: 0.03 g/kg body weight/day for 5 days. Duration: 10 days total (5 days loading + 5 days maintenance). Form of administration: oral, encapsulated, ingested with food in 3 equal doses per day.	Placebo, administered in encapsulated form following the same dosage and frequency protocol as the creatine group.	Creatine supplementation did not reduce muscle damage or enhance recovery following a hypoxic resistance exercise challenge. Lactate dehydrogenase and C-reactive protein did not increase following the exercise test.	Yes.
Santos et al 2004 (12)	Randomized, double-blind, placebo-controlled trial.	34 male athletes. Intervention group: n=18 Control group: n=16.	Mean age: 25.5 ± 3.2 years. Rage: 21.4 to 30.1 years.	Dose: 20 grams per day of creatine monohydrate, divided into 4 doses of 5g each, along with 15g of maltodextrin per dose. Duration: 5 days prior to the 30km race. Form of administration: oral supplementation, by diluting the powder in water.	The control group received the same amount of maltodextrin (60g total, or 15g per dose), prepared with the same flavor and color as the creatine solution to maintain blinding.	Creatine supplementation attenuated increases in prostaglandin E2 (PGE2) by 60.9%, and tumor necrosis factor-alpha (TNF-α) by 33.7% after the 30km race.	Yes.
Taes et al 2004 (24)	Randomized, double-blind, placebo-controlled, crossover design.	45 chronic hemodialysis patients completed the trial (out of 49 recruited). Intervention group: n=45. Control group: n=45	Mean age: 70 ± 10 years. Range: 35 to 88 years.	Dose: 2 grams per day of creatine monohydrate (CreaPure®). Duration: 4 weeks per treatment period. Patients received creatine in one of two 4-week periods. Form of administration: oral ingestion (tablets), taken daily in the evening.	Patients received placebo tablets (Fast Flo lactose) daily in the evening, following the same protocol as the creatine group, during one of the two 4-week treatment periods.	Creatine supplementation did not decrease total plasma homocysteine (tHcy) concentrations in chronic hemodialysis patients who were already receiving folic acid and vitamins B6 and B12. Plasma and red blood cell creatine levels significantly increased in the creatine-treated groups, confirming uptake.	Yes.
Tarnopolsky et al 2007 (25)	Randomized, double-blind, placebo-controlled trial.	39 community-dwelling older adults (19 men and 20 women). Intervention group: n=21. Control group: n=18.	Mean age: Approximately 70.9 years (calculated average from group means: Control Men 74.8 ± 6.6, Control Women 68.3 ± 4.4, Intervention Men 71.8 ± 5.2, Intervention Women 69.5 ± 3.8). Range: 65 to 85 years.	Dose: 5 grams per day of creatine monohydrate (Neotine®) plus 6 grams per day of conjugated linoleic acid (CLA-ONE®), along with 2 grams per day of dextrose. Duration: 6 months (24 weeks) of resistance exercise training. Form of administration: Oral supplementation, consumed daily.	The placebo group received 7 grams per day of dextrose plus 6 grams per day of safflower oil. The supplements were indistinguishable in flavor and appearance.	There were no significant changes in IL-6 and C-reactive protein.	Yes.

CLA, Conjugated Linoleic Acid; CRP, C-reactive Protein; IL-1β, Interleukin-1 beta; IL-6, Interleukin-6; IL-10, Interleukin-10; LDH, Lactate Dehydrogenase; PGE_2_, Prostaglandin E2; s100 A8/A9, S100 Calcium-Binding Protein A8/A9 (Calprotectin); TNF-α, Tumor Necrosis Factor alpha; tHcy, Total Plasma Homocysteine.

Santos et al. (12) reported that short-term creatine supplementation (20 g/day for 5 days) significantly attenuated inflammatory responses following a 30-kilometer race in male athletes. Specifically, creatine reduced post-race increases in prostaglandin E_2_ (PGE_2_) by 60.9% and tumor necrosis factor-α (TNF-α) by 33.7%, suggesting a protective effect against exercise-induced muscle damage and systemic inflammation. Similarly, Bassit et al. (4), using a similar protocol (20 g/day for 5 days), found that creatine supplementation in male triathletes led to significantly lower levels of TNF-α, interleukin-1β (IL-1β), and PGE_2_ after a half-Ironman triathlon. These findings reinforce the anti-inflammatory potential of creatine during prolonged and intense endurance activities, potentially via modulation of cytokine responses to physiological stress.

In contrast, several studies failed to observe such benefits in other contexts. Cornish and Peeler (14) administered creatine supplementation (20 g/day for 1 week followed by 5 g/day for 11 weeks) to patients with mild to moderate knee osteoarthritis and found no significant changes in inflammatory markers, including CRP, IL-1β, IL-6, s100 A8/A9, and TNF-α. This suggests that creatine may not confer anti-inflammatory effects in chronic low-grade inflammatory conditions. Similarly, Oliveira et al. (15) conducted a 12-week randomized trial in older adults (mean age 67 years) using a daily dose of 5 g of creatine monohydrate combined with resistance training. The results showed no significant differences between the creatine and placebo groups in key inflammatory markers such as IL-6, IL-10, adiponectin, leptin, or CRP, although both groups experienced reductions in MCP-1, indicating a potential effect of training itself rather than creatine.

Deldicque et al. (23) investigated the molecular effects of creatine at the gene expression level in young healthy men after just 5 days of supplementation (21 g/day) combined with acute resistance exercise. Although they observed increases in gene expression of muscle-related targets such as collagen-1, GLUT-4, and myosin heavy chains, no modulatory effect of creatine was found on IL-6 mRNA expression, indicating that creatine did not influence local inflammatory gene responses in muscle tissue under these conditions. Likewise, Rawson et al. (5) evaluated creatine’s effects on recovery from hypoxic resistance exercise in trained men over a 10-day supplementation protocol and reported no reduction in markers of muscle damage or inflammation. Notably, neither lactate dehydrogenase nor CRP increased following the exercise protocol, and creatine had no measurable effect on recovery outcomes.

Tarnopolsky et al. (25) further extended these findings to an older population undergoing 6 months of resistance training. In this trial, creatine (5 g/day) combined with conjugated linoleic acid did not result in any significant changes in IL-6 or CRP levels when compared to placebo, although improvements were observed in body composition and strength parameters. This highlights a potential disconnect between functional improvements and systemic inflammatory markers.

### Adverse effects

Santos et al. (12) reported that short-term creatine supplementation (20 g/day for 5 days) was well tolerated in male endurance athletes completing a 30-kilometer race. Importantly, the authors explicitly stated that no adverse effects were observed during the supplementation period or the race itself. Athletes did not report muscle cramping, dehydration, gastrointestinal discomfort, or diarrhea, and all participants completed the race within their expected performance range. These findings indicate that acute high-dose creatine loading did not induce clinically relevant side effects, even under conditions of prolonged and strenuous endurance exercise. Similarly, Bassit et al. (4), using an identical supplementation protocol (20 g/day for 5 days), reported no adverse clinical events in trained male triathletes competing in a half-Ironman event. No supplementation-related symptoms or health complaints were described during the pre-competition loading phase or the post-race follow-up, supporting the safety of creatine supplementation in the context of extreme physiological stress.

In addition, several studies conducted in clinical or aging populations also reported a neutral safety profile. Cornish and Peeler (14) administered creatine supplementation (20 g/day for 1 week followed by 5 g/day for 11 weeks) to patients with mild to moderate knee osteoarthritis and reported no adverse events throughout the 12-week intervention. No worsening of joint symptoms, functional limitations, or systemic health markers was observed, and creatine intake was not associated with gastrointestinal, musculoskeletal, or inflammatory complications. Likewise, Oliveira et al. (15) evaluated the effects of creatine supplementation (5 g/day for 12 weeks) combined with resistance training in older adults and found no evidence of adverse metabolic or inflammatory effects. The authors reported no negative changes in glucose homeostasis, insulin resistance, or circulating inflammatory biomarkers, indicating that creatine was safe and well tolerated in this older population when combined with structured exercise.

Deldicque et al. (23) investigated the short-term molecular effects of creatine supplementation (21 g/day for 5 days) in young healthy men undergoing acute resistance exercise with muscle biopsies. Despite the invasive nature of the protocol and the high supplementation dose, no adverse events or safety concerns related to creatine intake were reported. The absence of reported side effects suggests good tolerability of short-term creatine loading in healthy individuals under controlled experimental conditions. Similarly, Rawson et al. (5) examined the effects of creatine supplementation over 10 days in resistance-trained men and found no increase in muscle soreness, cramping, or biochemical markers indicative of muscle injury or systemic inflammation. The authors also noted that concerns regarding severe muscle dysfunction were not supported by their findings, reinforcing the short-term safety of creatine supplementation in resistance exercise settings.

Finally, Tarnopolsky et al. (25) extended safety observations to a longer-term intervention in older adults undergoing 6 months of resistance training. Creatine supplementation (5 g/day), combined with conjugated linoleic acid, resulted in a modest increase in plasma creatinine; however, creatinine clearance and overall renal function remained unchanged. No adverse effects were observed in liver enzymes, creatine kinase activity, or systemic inflammatory markers such as IL-6 and C-reactive protein. These results suggest that long-term creatine supplementation is generally safe in older adults when administered at moderate doses and under supervised exercise conditions.

### Synthesis of results

**Figure 2:**
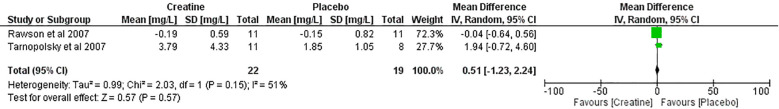
Meta-analysis for overall acute effects of creatine on C-reactive protein.

Acute Effects – CRP: To assess the acute effects of creatine supplementation on CRP, two trials were included (5, 25), totaling 61 participants. The pooled MD was 0.73 ng/L (95% CI: –1.16, 2.63), indicating no statistically significant difference between creatine and placebo (Z = 0.76, P = 0.45). The analysis showed substantial heterogeneity (Tau² = 1.44; χ² = 3.75, df = 1, P = 0.05; I² = 73%), suggesting considerable variation in study results (**Figure 2**).

Rawson et al. (5) demonstrated a negligible, non-significant effect (MD = –0.04 ng/L, 95% CI: –0.64, 0.56), whereas Tarnopolsky et al. (25) reported a larger but still non-significant increase in IL-6 favoring placebo (MD = 1.94 ng/L, 95% CI: 0.03, 3.85) (**Figure 2**).

**Figure 3:**
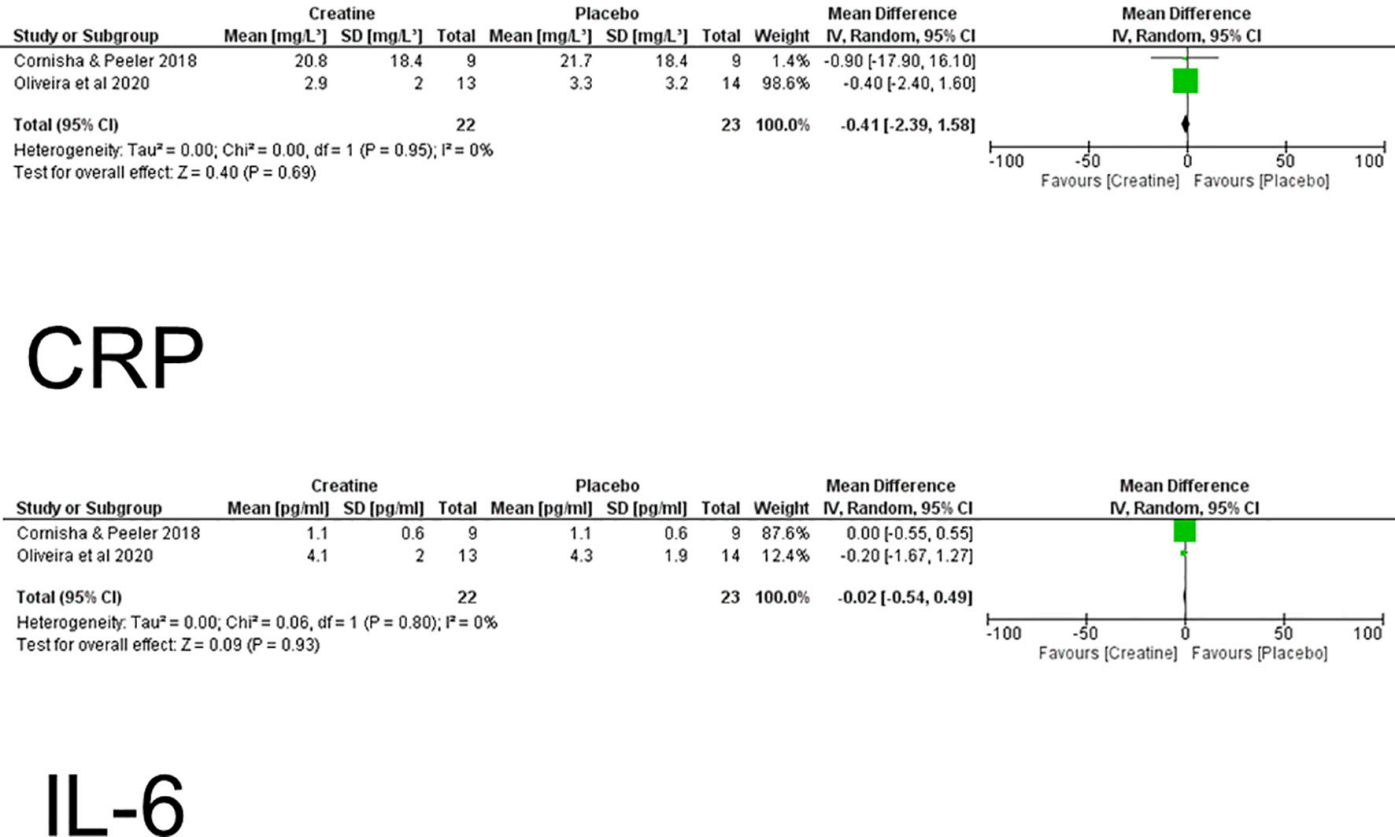
Meta-analysis for overall chronic effects of creatine on C-reactive protein (CRP) and interleukin-6 (IL-6).

Chronic Effects – CRP: The meta-analysis evaluating the chronic effects of creatine supplementation on CRP included two randomized controlled trials with a combined sample of 45 participants (14, 15). Using a random-effects model, the pooled MD was -0.41 mg/L (95% CI: –2.39, 1.58), indicating no statistically significant difference between creatine and placebo (Z = 0.40, P = 0.69). Heterogeneity was absent (Tau² = 0.00; χ² = 0.00, df = 1, P = 0.95; I² = 0%), suggesting high consistency across studies (**Figure 3**).

Examining individual trials, Cornish & Peeler (14) reported an MD of -0.90 mg/L (95% CI: –17.90, 16.10), while Oliveira et al. (15) showed a similar lack of effect (MD = -0.40 mg/L, 95% CI: –2.40, 1.60). Overall, these findings indicate that chronic creatine supplementation does not significantly influence CK concentrations compared with placebo (**Figure 3**).

Chronic Effects – IL-6: The meta-analysis assessing chronic effects of creatine on IL-6 also included two studies with a total of 45 participants. The pooled MD was -0.02 pg/mL (95% CI: –0.54, 0.49), showing no statistically significant difference between creatine and placebo (Z = 0.09, P = 0.93). Heterogeneity was very low (Tau² = 0.00; χ² = 0.06, df = 1, P = 0.80; I² = 0%), indicating very consistent findings across trials (**Figure 3**).

Individually, Cornish & Peeler (14) reported no difference between groups (MD = 0.00 pg/mL, 95% CI: –0.55, 0.55), whereas Oliveira et al. (15) found a small, non-significant reduction in favor of creatine (MD = –0.20 pg/mL, 95% CI: –1.67, 1.27). These results collectively demonstrate that chronic creatine supplementation does not alter CRP levels (**Figure 3**).

Overall, the evidence does not support an acute effect of creatine supplementation on IL-6 responses to exercise.

A formal assessment of publication bias, which typically involves inspecting a funnel plot or employing statistical methods like Egger’s or Begg’s tests, was not performed in this review. This decision aligns strictly with the current methodological standards set by the Cochrane Handbook for Systematic Reviews of Interventions. The guidance (26) strongly recommends a minimum number of studies to ensure the reliability of these analyses.

### Risk of bias

**Figure 4:**
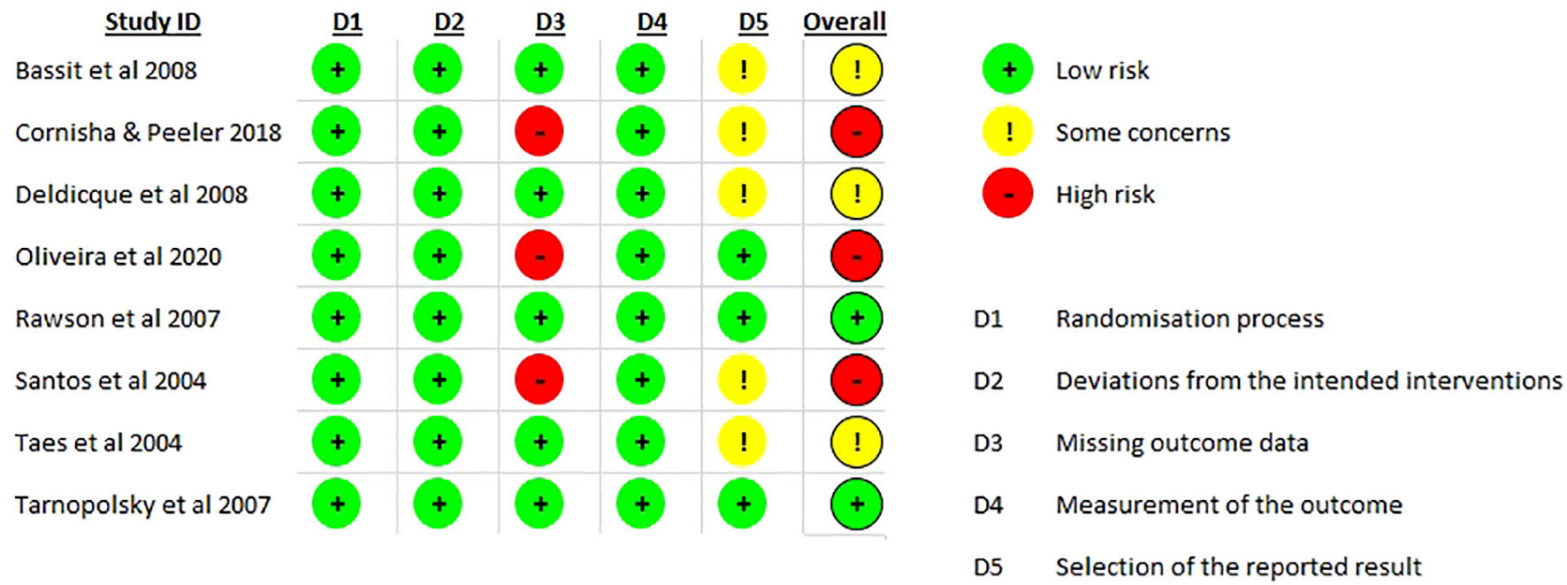
Cochrane risk of bias tool.

The risk of bias varied across the included studies, with concerns identified in several domains, including randomization, deviations from intended interventions, missing outcome data, outcome measurement, and selection of reported results. Overall, the studies demonstrated a mixture of low risk, some concerns, and high risk of bias (**Figure 4**).

#### Randomization process

All studies adequately described the randomization process and were judged as low risk for this domain. Randomization procedures appeared to have been properly implemented, with no evidence of selection bias or systematic differences between intervention groups at baseline.

#### Deviations from intended interventions

Across all studies, the risk of bias due to deviations from intended interventions was judged to be low. Most studies employed double-blind or placebo-controlled designs, minimizing the likelihood that participants’ or researchers’ awareness of the assigned interventions influenced the outcomes.

#### Missing outcome data

High risks were identified in 14, 15 and 12, where participant losses or incomplete reporting were not fully explained. The remaining studies either reported complete datasets or provided adequate justification for missing data, suggesting that attrition was unlikely to have affected the results.

#### Measurement of outcomes

All studies (100%) were rated as low risk for this domain. Outcome measures were obtained using validated and standardized procedures appropriate for the interventions. Although blinding of assessors was not explicitly described in all studies, measurement bias was considered minimal.

#### Selection of reported results

Most studies (4, 12, 14, 23 and 24) raised some concerns regarding selective reporting, as pre-specified protocols or analysis plans were not always clearly available. Despite this, reported outcomes were generally consistent with study aims and expected endpoints.

#### Overall risk of bias

### GRADE assessment

The GRADE assessment indicated that the overall quality of evidence regarding the effects of creatine supplementation on inflammatory markers was moderate, though limited by concerns related to risk of bias (**Table 2**). Specifically, the domains of inconsistency, indirectness, and imprecision were not considered serious, suggesting reasonable consistency and directness across studies. However, the presence of missing outcome data led to a classification of *very serious risk of bias* for all evaluated outcomes.

**Table 2:** Levels of evidence analysis via (18).

Outcome	No. of studies	Risk of Bias	Inconsistency	Indirectness	Imprecision	Certainty of evidence
CRP acute effects	2	Very Serious^a^	Serious^b^	Not serious	Not serious	Very low
CRP chronic effects	2	Very Serious^a^	Not serious	Not serious	Not serious	Low
IL-6 chronic effects	2	Very Serious^a^	Not serious	Not serious	Not serious	Low

^a^ Missing outcome data.

^b^ I^2^ between 50% and 75%.

- CRP (acute effects): Very certainty.
- CRP (chronic effects): Low certainty.
- IL-6 (chronic effects): Low certainty.

Detailed explanations for these GRADE ratings, including considerations of data completeness and study design, are provided in the **Supplementary Material**.

### Heterogeneity

The moderate heterogeneity observed among studies assessing inflammatory outcomes may be attributed to methodological and clinical differences. These include variations in supplementation duration (ranging from short-term to several weeks), participant characteristics (e.g., trained athletes versus older adults), and exercise protocols (endurance versus resistance training). Moreover, some trials did not provide detailed information about blinding or adherence to supplementation, potentially influencing the magnitude of observed effects. Despite these discrepancies, the overall direction of the results was consistent, indicating a potential anti-inflammatory effect of creatine supplementation under different experimental conditions.

## Discussion

### Summary of key findings

Our systematic review with meta-analysis aimed to evaluate the effects of creatine supplementation on inflammatory markers in human populations. As key findings, we observed that: Creatine supplementation did not consistently reduce biomarkers associated with chronic low-grade inflammation, specifically CRP or IL-6, across diverse clinical or elderly populations. For instance, plasma IL-6 concentrations were not significantly affected by creatine supplementation after an exhaustive competition (4), nor did creatine plus resistance training provide additional benefits on IL-6 or CRP in older adults compared to resistance training alone. While some individual trials showed benefit in athletes under high physiological stress, results were inconsistent or absent in older adults and clinical populations. In fact, short-term creatine failed to reduce muscle damage markers like CK or muscle soreness following hypoxic resistance exercise in trained men. Overall certainty of evidence for specific chronic markers in specific populations appears limited, and risk of bias concerns were present in several small-scale studies.

### Physiological mechanisms of creatine and inflammation

The anti-inflammatory effects of creatine appear to be strongly dependent on the physiological context in which supplementation is applied (27, 28). One of the primary proposed mechanisms is related to cytoprotective effects on muscle cells post-exercise. Creatine loading increases muscle intracellular water content, promoting cell swelling. This may increase muscle cell resistance to mechanical injury, thereby reducing cell death and mitigating the ensuing inflammatory process as a whole (29, 30). Supporting this, studies demonstrating efficacy noted that creatine supplementation abolished the increase in LDH (12).

Rather than exerting a generalized anti-inflammatory action, creatine seems to preferentially attenuate inflammatory responses associated with acute, high-magnitude mechanical and metabolic stress, such as prolonged endurance exercise, while showing limited efficacy in conditions characterized by chronic, low-grade inflammation (4, 14).

One plausible explanation lies in creatine’s cytoprotective role at the cellular level during acute muscle stress. Creatine loading increases intracellular water content, promoting transient cell swelling, which enhances membrane stability and resistance to mechanically induced damage (29, 30). This protective effect may reduce myofiber disruption and subsequent cell lysis, thereby limiting the release of damage-associated molecular signals that trigger acute inflammatory cascades. Consistent with this mechanism, studies conducted under extreme endurance conditions reported that creatine supplementation abolished or attenuated post-exercise increases in LDH, a marker of cellular damage, following high-intensity running and competition stress (12).

In parallel, creatine may modulate inflammatory signaling during acute stress by attenuating the production or release of pro-inflammatory mediators. Previous studies have shown reductions in circulating TNF-α, IL-1β, and PGE_2_ following strenuous endurance exercise in creatine-supplemented athletes (4, 12). These effects may be partially mediated by alterations in cellular energy status and purinergic signaling, as *in vitro* evidence suggests that creatine can reduce neutrophil adhesion through downregulation of adhesion molecules, potentially involving adenosine A2A receptor activation secondary to changes in ATP and phosphocreatine availability (14, 28).

By contrast, these mechanisms may be insufficient to meaningfully influence chronic low-grade inflammation, which is driven by complex, systemic processes including immunometabolic dysregulation, adipose tissue signaling, oxidative stress, and age-related immune remodeling. In such conditions, baseline inflammatory activity is sustained rather than triggered by acute tissue damage, and thus less responsive to interventions primarily targeting muscle cell integrity or short-term inflammatory signaling (2). This may explain why creatine supplementation failed to reduce markers such as CRP and IL-6 in populations with knee osteoarthritis or in older adults, despite prolonged supplementation periods (14, 15).

Although creatine exhibits direct antioxidant properties that could theoretically contribute to anti-inflammatory effects (31), these actions appear modest in the context of chronic systemic inflammation. Taken together, the available evidence supports the interpretation that creatine acts predominantly as a context-specific cytoprotective and anti-inflammatory agent, mitigating inflammatory responses secondary to acute, high-intensity physiological stress, rather than as a broad modulator of chronic inflammatory states. This distinction is critical for accurately interpreting the heterogeneous findings across trials and for avoiding overgeneralization of creatine’s anti-inflammatory potential beyond the conditions in which it is most biologically plausible and empirically supported.

### Contextualization with prior literature

Our findings reinforce the concept that the anti-inflammatory effects of creatine are highly context-dependent. Evidence supporting beneficial effects is largely confined to scenarios involving acute, high-magnitude physiological stress. In endurance athletes exposed to extreme exertion, short-term creatine loading attenuated post-exercise increases in inflammatory mediators, including TNF-α and PGE_2_ after a 30 km race (12), as well as TNF-α, IFN-α, and IL-1β following a half-Ironman competition (4). These findings suggest that creatine may mitigate inflammation secondary to substantial muscle damage and metabolic stress, rather than exerting a generalized anti-inflammatory effect.

In contrast, this efficacy does not extend to conditions characterized by chronic low-grade inflammation or to resistance exercise models that do not elicit comparable systemic inflammatory stress. In clinical populations with knee osteoarthritis, 12 weeks of creatine supplementation failed to alter inflammatory biomarkers or cartilage degradation markers (14). Similarly, in older adults, creatine combined with resistance training did not provide additional reductions in inflammatory markers or insulin resistance beyond those achieved by training alone (15). Moreover, creatine did not attenuate markers of muscle damage or inflammation following hypoxic resistance exercise, indicating that its protective effects observed in endurance running do not generalize to resistance-based muscle damage models (5).

Collectively, these findings underscore that creatine’s anti-inflammatory potential is contingent upon the nature and intensity of the physiological stressor. Creatine appears most effective in attenuating inflammation arising from acute, high-load endurance stress, whereas it shows limited or no efficacy in chronic inflammatory states or resistance exercise contexts with lower systemic inflammatory demand. This context-dependent profile provides a unifying framework for interpreting the heterogeneous results across trials and aligns closely with the overall findings of the present systematic review and meta-analysis.

#### Contrast of results across subgroups

Based on our findings, across populations, beneficial effects are largely confined to trained endurance athletes exposed to extreme exercise demands. In these settings, short-term creatine loading significantly attenuated exercise-induced increases in pro-inflammatory cytokines (TNF-α, IFN-α, IL-1β) and PGE2 following both a half-Ironman competition and a 30-km race (4, 12). In contrast, studies conducted in clinical populations or older adults, characterized by sustained low-grade inflammation, uniformly reported null findings. Twelve weeks of creatine supplementation did not alter CRP, IL-6, TNF-α, IL-1β, or s100 A8/A9 levels in individuals with knee osteoarthritis (14), nor did creatine combined with resistance training improve systemic inflammatory markers in community-dwelling older adults, despite exercise-induced reductions in MCP-1 (15).

With this in mind, exercise modality further reinforces this context specificity. Anti-inflammatory effects were observed in endurance-based or high-volume eccentric protocols that induce substantial metabolic and mechanical stress (4). Conversely, resistance exercise models designed to provoke localized muscle damage, such as hypoxic squatting or high-force eccentric contractions, did not show reductions in muscle damage or inflammatory markers with creatine supplementation compared with placebo (5). This divergence suggests that creatine’s protective effects are not universally transferable across exercise modalities.

Moreover, supplementation strategy appears to be a critical moderator. Acute loading protocols, typically involving ~20 g/day for five days before a single strenuous event, were effective in blunting inflammatory responses in endurance settings (4, 12). In contrast, chronic maintenance dosing (e.g., 5 g/day for 12 weeks) failed to reduce systemic markers of chronic inflammation such as CRP and IL-6 (14, 15). Collectively, these findings support the interpretation that creatine functions primarily as a cytoprotective agent against acute, exercise-induced tissue stress, rather than as a modulator of sustained chronic low-grade inflammation.

#### Meta-analysis interpretation

Assuming a meta-analysis focused on key chronic markers (CRP and IL-6), the pooled effect size would likely be interpreted as statistically insignificant.

SMDs, Confidence intervals (CIs), and Heterogeneity (I²): In a meta-analysis, the SMD represents the magnitude of the intervention effect across studies using different measurement scales. CIs define the range within which the true pooled effect likely lies. Heterogeneity, often quantified by the I² statistic, describes the proportion of total variation in study estimates that is due to genuine differences in the true effects between studies, rather than just sampling error (20). An I² value is considered more useful than the basic test for heterogeneity, as I² does not intrinsically depend on the number of studies (20).

Acute CRP and Chronic CRP/IL-6: Meta-analysis focusing on CRP and IL-6 across chronic (long-term) studies would reveal no significant effect of creatine supplementation (14, 15). Given the consistent null findings for these specific markers in chronic, low-grade inflammatory conditions (14, 15), the I² for these outcomes would likely be low, suggesting consistent results.

Divergence from Individual Studies: The pooled estimates showing a lack of chronic effect may differ from promising individual studies focused on acute outcomes (4, 12). This divergence arises because meta-analysis pools heterogeneous populations and outcomes. For instance, creatine significantly reduced TNF-α, IL-1β, IFN-α, and PGE2 in healthy triathletes (4, 12), effects that are highly relevant to muscle trauma (4). However, when these effects are pooled with data from older adults whose inflammation is linked to metabolic disease rather than eccentric exercise (15), the overall effect on systemic markers like IL-6 and CRP might become negligible.

#### GRADE and risk of bias interpretation

The certainty of the overall evidence was rated as very low to low. This lower certainty is partly due to missing outcome data and the inconsistent protocols used across trials. Many included studies were small-scale (e.g., N = 5 in one group in the half-ironman study (4), N = 9 in the OA study (14)), limiting statistical power (5).

While most studies were double-blinded and employed appropriate randomization methods (4, 5, 14), selective outcome reporting and attrition (loss of subjects) were notable limitations (14). For example, the study on knee osteoarthritis patients was limited by its small sample size, which may have influenced the ability to detect significant differences. High variability in biomarker results also limited the findings (14).

#### Clinical implications

The practical application of creatine as an anti-inflammatory agent is currently compartmentalized by context:

With respect to athletes (especially in Endurance Events), creatine supplementation has demonstrable benefits in reducing acute exercise-induced inflammation, muscle damage (LDH), and pain mediators (PGE2) following exhaustive, long-distance events (4, 12). Thus, creatine is a valuable tool for maintaining muscle integrity and accelerating recovery in this population (4, 5, 12).

In relation to older adults or those with chronic inflammation: Creatine alone is not supported as an intervention to reduce established chronic inflammatory markers (CRP, IL-6, TNF-α) in conditions like knee osteoarthritis or general aging (14, 15, 25). While creatine combined with resistance training offers significant benefits in improving fat-free mass and strength in older adults (25), the hypothesized anti-inflammatory mechanism does not appear to operate reliably in chronic disease settings (14).

Caution must be exercised to avoid overgeneralizing the positive results observed in specific acute exercise models to chronic disease management (5, 14).

Furthermore, building on this contextual framework, the null findings observed in the pooled analyses should not be interpreted as evidence that creatine lacks any clinical or practical relevance, but rather that its effects are unlikely to manifest as meaningful reductions in conventional systemic inflammatory biomarkers in chronic settings. In populations characterized by persistent low-grade inflammation, such as older adults or individuals with osteoarthritis, the magnitude of any creatine-related effect on CRP or IL-6, if present, appears to be small and well below thresholds typically considered clinically relevant. Even modest changes in these biomarkers may theoretically contribute to long-term risk modification in cardiometabolic disease; however, the available evidence suggests that creatine supplementation alone is insufficient to consistently induce such changes, and therefore should not be positioned as a primary anti-inflammatory strategy in these populations.

From a clinical perspective, these findings indicate that creatine’s value in older or clinical populations should be framed around its well-established benefits on muscle mass, strength, and functional capacity, rather than expectations of systemic inflammation reduction. Improvements in physical function, sarcopenia prevention, and exercise tolerance may indirectly influence inflammatory status over longer time horizons, but these downstream effects are not captured by short- to medium-term changes in CRP or IL-6. Thus, clinicians and practitioners should be cautious in extrapolating anti-inflammatory claims beyond acute exercise contexts and should prioritize creatine for its musculoskeletal and performance-related benefits when counseling patients or older adults.

In performance and applied sport settings, the absence of a measurable overall effect in meta-analytic models does not negate the practical relevance of creatine during periods of extreme physiological stress. Rather, it underscores that creatine’s anti-inflammatory action is situational, emerging primarily when inflammation is tightly coupled to acute muscle damage and metabolic strain. In this context, even transient reductions in inflammatory mediators or muscle damage markers may have meaningful implications for recovery, training continuity, and competition readiness, despite not translating into sustained changes in basal inflammatory biomarkers (32).

Moreover, although confidence intervals allow for very small effects, several factors indicate that a clinically meaningful reduction in systemic inflammation is unlikely. Point estimates are small and inconsistent, and upper bounds remain below clinically relevant thresholds for CRP. For chronic outcomes (CRP and IL-6), pooled effects were near zero with narrow confidence intervals and no heterogeneity, effectively ruling out moderate or large benefits. While individual studies were underpowered, the consistent null findings across populations, combined with very low to low GRADE certainty, suggest any true effect is trivial.

#### Study limitations

A major limitation of the overall evidence base stems from the high heterogeneity in study designs, encompassing vastly different populations, physiological states, and dosing protocols. The eight included trials ranged from elite endurance athletes performing strenuous exercise bouts to older adults in community settings, individuals with chronic conditions such as osteoarthritis or renal dysfunction, and healthy young adults. These groups differ markedly in baseline inflammatory status, metabolic capacity, muscle physiology, and adaptive responses to supplementation and exercise. Consequently, their responsiveness to creatine supplementation is unlikely to be uniform, and the observed effects may be influenced by underlying health status, habitual activity level, or disease-related metabolic alterations.

Furthermore, the included studies varied not only in population characteristics but also in methodological features such as intervention duration, creatine loading and maintenance doses, timing of supplementation relative to exercise, and the presence or absence of co-interventions (e.g., resistance or endurance training protocols). Additionally, the inflammatory biomarkers assessed were not consistent across trials, with some studies measuring acute post-exercise cytokine responses and others focusing on chronic markers such as CRP or IL-6. These methodological discrepancies introduce additional layers of variability that complicate direct comparisons between studies.

Such extensive heterogeneity may have reduced the interpretability and reliability of the pooled estimates, as the underlying biological mechanisms and expected magnitude of effect likely differ across these diverse clinical and physiological contexts. For instance, creatine may modulate inflammatory pathways differently in an acutely stressed athlete compared to an individual with chronic low-grade inflammation, yet the meta-analytic approach inherently averages these effects. As a consequence, the aggregated effect sizes should be interpreted with caution, as they may obscure meaningful subgroup-specific responses or dilute potential benefits detectable only within more homogeneous cohorts. This limitation underscores the need for future research to stratify analyses by population type, baseline inflammatory profile, and intervention characteristics to better elucidate context-specific effects of creatine supplementation.

Small sample sizes in individual RCTs. Many foundational trials suffered from small subject numbers, limiting statistical power to detect meaningful differences (e.g., the half-ironman study used N = 11 total triathletes (4)). Few studies evaluated multiple inflammatory markers simultaneously. While some studies examined comprehensive cytokine panels (4), many focused only on one or two specific markers (e.g., CK or CRP), potentially missing broader effects (5). There is a lack of long-term trials in clinical populations. The absence of extended trials (e.g., 6 months to 1 year) in clinical groups makes it difficult to assess the long-term impact of creatine on disease progression (14). There is also an absence of standardized outcome reporting protocols. The lack of consistent reporting methods hinders comparative analysis across studies.

In addition, Tarnopolsky et al. (25) administered creatine in combination with conjugated linoleic acid. To minimize the potential confounding effect of the co-intervention in the trial by Tarnopolsky et al. (25), two methodological and interpretative steps were undertaken:

Consistency with studies without co-intervention: The inflammatory outcomes reported in this study, primarily CRP and IL-6, were qualitatively consistent with findings from other included trials that administered creatine alone. These studies similarly failed to demonstrate significant reductions in systemic inflammatory markers. This convergence across trials reduces the likelihood that concomitant conjugated linoleic acid supplementation masked a clinically meaningful anti-inflammatory effect of creatine in the Tarnopolsky et al. study.

Sensitivity analysis and cautious interpretation: Sensitivity analyses excluding the Tarnopolsky et al. trial did not materially alter the direction (See online supplementary files), magnitude, or statistical significance of the pooled estimates (MD = −0.04; 95% CI: −0.65 to 0.56; p = 0.90). Given the robustness of the pooled results, the study was retained in the quantitative synthesis. Nonetheless, we explicitly clarified that its findings should be interpreted as reflecting the effects of creatine in the presence of conjugated linoleic acid, thereby representing a specific intervention context rather than isolated creatine supplementation.

Taken together, these considerations indicate that the potential anti-inflammatory effects of conjugated linoleic acid were adequately controlled within the original trial design and do not compromise the interpretation of the present meta-analysis regarding the impact of creatine on systemic inflammatory markers.

### Sources of heterogeneity

The synthesis of evidence is significantly impacted by profound heterogeneity in study populations and activity levels. Participants ranged widely from young, highly trained male athletes, such as triathletes (mean age: 40.3 ± 2.18 years) (4) and marathon runners (mean age: 25.5 ± 3.2 years) (12), to older adults (mean age: 67 ± 5 years) (15) and clinical populations with pre-existing conditions like mild to moderate knee osteoarthritis (mean age: 57.1 ± 7.4 years) (14) or hemodialysis patients (24, 33, 34). The median age across these studies spans from young men (21.7 ± 0.55 years) (23) up to 70 ± 10 years in hemodialysis patients (24).

Furthermore, the exercise protocols varied fundamentally by intensity and type, targeting either acute trauma or chronic adaptation. Studies induced extreme endurance stress (e.g., half-ironman competition or a 30 km race) (4, 12), tested recovery following high-volume resistance exercise protocols designed to be hypoxic (5), or involved long-term supervised resistance training (15, 25). Conversely, some trials involved patients with no specific exercise training added to the creatine intervention (14, 24, 33, 34).

Dosage and Duration varied from short acute loading phases (e.g., 5 consecutive days of 20 grams/day) prior to a competition (4, 12) or a short study duration of 5 days (23), to chronic supplementation lasting 12 weeks (14, 15) and up to 12 months (34). Daily doses varied dramatically, including low fixed maintenance doses of 2 grams/day (24) or 5 grams/day (15, 25, 34), intermediate fixed doses of 20 grams/day (4, 12), and very high doses of 60 grams/day used in a 3-day acute loading phase (35). In addition, dosing was sometimes adjusted based on body weight, such as 0.3 g/kg body weight/day during a 5-day loading phase (5).

Such marked variability in dosage and duration is likely to be a meaningful contributor to the heterogeneity observed across studies and may partially explain inconsistencies between acute and chronic inflammatory outcomes. Short-term loading protocols, typically characterized by high daily doses administered over a few days, are more likely to elicit transient metabolic and osmotic effects, which may acutely influence inflammatory markers in response to exercise or competition stress. In contrast, longer-term supplementation using lower maintenance doses may promote gradual adaptations in muscle metabolism, cellular energetics, and recovery processes, potentially leading to different inflammatory profiles over time (10). These protocol-dependent physiological distinctions complicate direct comparisons between studies and limit the interpretability of pooled estimates when acute and chronic interventions are analyzed together. Although dose- or duration-based subgroup analyses were not feasible due to the limited number of homogeneous trials, acknowledging these protocol differences is essential for contextualizing the findings and underscores the need for future trials designed to systematically compare loading versus maintenance strategies on inflammatory outcomes.

Timing and co-Intervention also differed. Creatine was typically administered in multiple equal doses daily (4, 5, 12). In exercise trials, supplements were sometimes consumed immediately after training sessions (15), while in others, they were administered daily in the evening (24). Notably, one major study combined creatine with conjugated linoleic acid (CLA) (25), introducing a confounding factor when evaluating the independent effect of creatine.

The outcomes of inflammatory marker measurement showed extensive variability, hindering direct comparison of the anti-inflammatory effects of creatine. Studies focused on different physiological aspects of inflammation. Several studies quantified immediate inflammatory and muscle damage responses in plasma, reporting markers like TNF-α, IL-1β, IL-6, PGE2, and acute muscle damage indicators such as CK and LDH (4, 5, 12, 14). Other studies focused on markers typically associated with chronic low-grade inflammation, such as CRP, IL-6, IL-10, and Monocyte Chemoattractant Protein-1 (14, 15, 25). Specialized markers were also assessed, including serum cartilage oligomeric matrix protein (14), and markers of systemic stress like the Malnutrition-Inflammation Score (33). Crucially, one study investigated molecular mechanisms by measuring gene expression (e.g., MHC I, MHC IIA, IL-6 mRNA) and signaling pathways in muscle tissue via biopsies and real-time PCR, rather than just plasma concentrations (23).

Limiting the quantitative synthesis to CRP and IL-6 inevitably frames the overall conclusions toward a more conservative interpretation of creatine’s anti-inflammatory potential. These markers were selected because they were the only outcomes reported by a sufficient number of studies with comparable methodologies to allow statistically valid pooling. However, CRP and IL-6 predominantly reflect chronic, systemic low-grade inflammation and are relatively insensitive to transient, exercise-induced inflammatory responses or localized tissue-level adaptations. As a result, the pooled null findings primarily indicate that creatine supplementation is unlikely to induce clinically meaningful reductions in conventional systemic inflammatory biomarkers in chronic or mixed populations, rather than disproving all potential anti-inflammatory effects of creatine.

In contrast, markers such as TNF-α, IL-1β, and PGE_2_, although not suitable for meta-analysis due to the limited number of studies and substantial methodological heterogeneity, are more closely tied to acute inflammatory signaling, muscle damage, and recovery processes (36, 37). Positive findings reported for these outcomes in individual trials, particularly in endurance athletes exposed to extreme physiological stress, suggest that creatine’s anti-inflammatory actions may be context-specific and temporally restricted. Therefore, the selective pooling does not negate evidence of benefit observed for other inflammatory mediators but instead delineates the boundaries within which conclusions can be generalized.

Taken together, this approach emphasizes that the conclusions of the meta-analysis are marker- and context-dependent. The lack of pooled effects on CRP and IL-6 should be interpreted as evidence against a broad, systemic anti-inflammatory role for creatine in chronic conditions, while still allowing for targeted anti-inflammatory or recovery-related effects under acute, high-stress exercise conditions that are captured by different biomarkers but could not be quantitatively synthesized.

It is worth noting that a formal assessment of publication bias, typically conducted via visual inspection of a funnel plot or statistical tests such as Egger’s or Begg’s tests, was not performed in this systematic review. This decision is strictly based on methodological guidelines stipulated in the Cochrane Handbook for Systematic Reviews of Interventions. Current guidance (26) strongly advises against conducting these specific analyses when the number of studies included for a particular outcome is small. Specifically, reliable detection of publication bias requires a sufficient number of data points to ensure that any observed asymmetry in the funnel plot is genuinely due to bias and not merely chance or heterogeneity. The Cochrane Handbook recommends a minimum threshold of ten included studies to proceed with a reliable formal assessment. Since our final meta-analysis included only two studies, proceeding with a formal statistical or graphical assessment would yield results that are unreliable and potentially misleading. Reporting such a result could inaccurately suggest either the presence or absence of bias. Therefore, while we acknowledge the potential for publication bias as a limitation inherent in any small body of evidence, we adhered to best methodological practice by omitting the formal assessment, prioritizing the integrity and reliability of our analytical procedures.

#### Suggestions for future research

Future research must prioritize methodological rigor to overcome current limitations. There is a crucial need for large-scale, double-blind RCTs employing standardized dosing and standardized outcome reporting (14). Studies should assess comprehensive biomarker panels (e.g., TNF-α, CRP, IL1-β, IL-6) rather than relying on single markers to capture the full scope of anti-inflammatory activity (4, 14).

Based on the evidence gaps and the mechanistic reconciliation that posits a context-dependent, cytoprotective role for creatine, definitive trials must be rigorously designed to test this core hypothesis. To maximize the biological plausibility of detecting an effect, future studies should focus on contexts involving acute, high-magnitude mechanical stress, where creatine’s cytoprotective mechanisms (cell swelling, enhanced membrane stability) are expected to be most operative. The most critical population to study, therefore, is trained individuals undergoing a standardized, unaccustomed muscle-damaging protocol (e.g., high-volume eccentric resistance exercise or severe downhill running). This setting guarantees the specific tissue damage required to trigger the inflammatory cascade that creatine is hypothesized to mitigate.

A secondary priority involves ultra-endurance athletes participating in major competitions (e.g., marathon, Ironman), as this provides a real-world scenario of both intense metabolic and mechanical stress. Furthermore, future RCTs must utilize markers that directly assess the initial stages of tissue damage and the immediate inflammatory response. The most critical primary endpoints are Markers of Cellular Damage (CK and LDH), as testing for significant attenuation of their post-exercise rise is essential to confirm the hypothesized cytoprotective mechanism.

Additionally, high priority should be given to acute pro-inflammatory cytokines (TNF-***α***, IL-1**β**), which are released early in the acute cascade following tissue injury. Measurement must occur at multiple, precise, short-term intervals (e.g., 6h, 24h, 48h post-stress) to accurately capture the peak modulation effect, rather than relying on general markers of chronic inflammation like CRP.

### Analogy for context-dependent effects

Creatine’s effect on inflammation is like a specialized fire extinguisher. It is highly effective at immediately putting out intense, acute fires caused by extreme physiological stress (strenuous endurance exercise), protecting the cells from immediate damage (4, 12). However, it appears much less effective when dealing with the slow, smoldering structural decay of chronic, low-grade inflammation associated with aging or chronic diseases, where different underlying mechanisms are driving the problem (14, 15).

## Conclusion

In summary, the current body of evidence does not support a consistent anti-inflammatory effect of creatine supplementation in humans, particularly regarding chronic low-grade inflammation markers such as CRP and IL-6. While short-term loading protocols in endurance athletes demonstrate a reduction in exercise-induced cytokine release and muscle damage mediators, these effects do not translate to older adults, clinical populations, or long-term supplementation settings. The certainty of evidence is constrained by small sample sizes, heterogeneous biomarker panels, variability in dosing regimens, and inconsistent reporting of inflammatory outcomes. Future randomized controlled trials should prioritize larger cohorts, standardized inflammatory endpoints, harmonized supplementation protocols, and population-specific hypotheses (e.g., acute vs. chronic inflammation, endurance vs. resistance stress). Such methodological refinement is essential to clarify whether creatine acts as a targeted modulator of acute inflammatory stress or if its potential benefits extend to chronic inflammatory states with clinical relevance.

## Data Availability

All data supporting the findings of this study are included in the article/**Supplementary Material**, further inquiries can be directed to the corresponding author/s.
