# Correction: Impact of creatine supplementation on inflammation: evidence from a systematic review and meta-analysis of randomized double-blind placebo trials

**DOI:** 10.3389/fimmu.2026.1840983

**Published:** 2026-04-10

**Authors:** Kell Mazzini Ribeiro de Camargo, Alejandro Bruna-Mejías, Juan José Valenzuela-Fuenzalida, Luana A. Gonzaga, Sandra Maria Barbalho, Alexandre L. Barroca, Andrey A. Porto, Rodrigo D. Raimundo, Luiz Carlos de Abreu, Vitor E. Valenti

**Affiliations:** 1 Systematic Reviews Center for Cardiovascular and Metabolic Health, School of Philosophy and Sciences, São Paulo State University, Marília, SP, Brazil; 2 Escuela de Medicina, Facultad de Medicina, Universidad Andres Bello, Santiago, Chile; 3 Departamento de Ciencias y Geografia, Facultad de Ciencias Naturales y Exactas, Universidad de Playa Ancha, Valparaiso, Chile; 4 Departamento de Ciencias Químicas y Biológicas, Facultad de Ciencias de la Salud, Universidad Bernardo O’Higgins, Santiago, Chile; 5 Postgraduate Program in Structural and Functional Interactions in Rehabilitation, School of Medicine, Universidade de Mar´ılia (UNIMAR), Marília, São Paulo, Brazil; 6 Department of Biochemistry and Nutrition, School of Food and Technology of Marilia (FATEC), Marília, São Paulo, Brazil; 7 UNIMAR Charity Hospital, Universidade de Marilia (UNIMAR), Marília, São Paulo, Brazil; 8 Laboratorio de Delineamento de Estudos e Escrita Cient´ıfica, Centro Universitario FMABC, Santo Andre, SP, Brazil; 9 Department of Public Health, University of Limerick, Limerick, IE, Ireland; 10 Department of Nutrition, Federal University of Vitoria, Vitoria, ES, Brazil

**Keywords:** creatine, CRP, cytokines, IL-6, inflammation, meta-analysis, supplementation

Alejandro Bruna-Mejías was erroneously assigned to affiliation, “Systematic Reviews Center for Cardiovascular and Metabolic Health, School of Philosophy and Sciences, São Paulo State University, Marília, SP, Brazil” and affiliation “Escuela de Medicina, Facultad de Medicina, Universidad Andres Bello, Santiago, 8370146, Chile,” was omitted. The correct affiliations for Alejandro Bruna-Mejías are: “Escuela de Medicina, Facultad de Medicina, Universidad Andres Bello, Santiago, Chile” and “Departamento de Ciencias y Geografia, Facultad de Ciencias Naturales y Exactas, Universidad de Playa Ancha, Valparaiso, Chile.”

Juan Jose Valenzuela-Fuenzalida was erroneously assigned to Affiliations 1 and 2, “Systematic Reviews Center for Cardiovascular and Metabolic Health, School of Philosophy and Sciences, São Paulo State University, Marília, SP, Brazil” and “Departamento de Ciencias y Geografia, Facultad de Ciencias Naturales y Exactas, Universidad de Playa Ancha, Valparaiso, Chile.” The correct affiliations for Juan Jose Valenzuela-Fuenzalida are affiliations “Departamento de Ciencias Químicas y Biológicas, Facultad de Ciencias de la Salud, Universidad Bernardo O’Higgins, Santiago, Chile”

The original version of this article has been updated.

