# Supplementary material for: Impact of creatine supplementation on inflammation: evidence from a systematic review and meta-analysis of randomized double-blind placebo trials
Source: Front Immunol. 2026 Feb 19;17:1743603. doi: 10.3389/fimmu.2026.1743603 (PMC12961398; doi:10.3389/fimmu.2026.1743603)
Supplement: Supplementary file 1 [file DataSheet1.docx]

| **Database** | **Search Strategy** |
| --- | --- |
| PUBMED | ("Creatine Supplement"[Title/Abstract] OR "Creatine supplementation"[Title/Abstract] OR "Creatine monohydrate supplementation"[Title/Abstract] OR "Creatine"[MeSH])  AND  ("Inflammation"[MeSH Terms] OR "Cytokines"[MeSH Terms] OR "Interleukins"[MeSH Terms] OR "Inflammation"[Title/Abstract] OR "Cytokine"[Title/Abstract] OR "Interleukin"[Title/Abstract]) |
| EMBASE | ('creatine supplementation'/exp OR 'creatine supplement':ti,ab OR 'creatine monohydrate supplementation':ti,ab)  AND  ('inflammation'/exp OR 'cytokine'/exp OR 'interleukin'/exp OR inflammation:ti,ab OR cytokine:ti,ab OR interleukin:ti,ab) |
| LILACS | ("Creatine Supplement" OR "Creatine supplementation" OR "Creatine monohydrate supplementation")  AND  ("Inflammation" OR "Cytokine" OR "Interleukin") |
| Cochrane Library | ("Creatine Supplement" OR "Creatine supplementation" OR "Creatine monohydrate supplementation")  AND  ("Inflammation" OR "Cytokine" OR "Interleukin") |
| SCOPUS | TITLE-ABS-KEY("Creatine Supplement" OR "Creatine supplementation" OR "Creatine monohydrate supplementation")  AND  TITLE-ABS-KEY("Inflammation" OR "Cytokine" OR "Interleukin") |
| Web of Science (Coleção principal) | TS=("Creatine Supplement" OR "Creatine supplementation" OR "Creatine monohydrate supplementation")  AND  TS=("Inflammation" OR "Cytokine" OR "Interleukin") |
| CINAHL | ("Creatine Supplement" OR "Creatine supplementation" OR "Creatine monohydrate supplementation")  AND  ("Inflammation" OR "Cytokine" OR "Interleukin") |
