# Supplementary material for: Impact of creatine supplementation on inflammation: evidence from a systematic review and meta-analysis of randomized double-blind placebo trials
Source: Front Immunol. 2026 Feb 19;17:1743603. doi: 10.3389/fimmu.2026.1743603 (PMC12961398; doi:10.3389/fimmu.2026.1743603)
Supplement: Supplementary file 2 [file SupplementaryFile1.zip › SR Creatine inflammatory markers (Kell Doutorado). /Lista de revistas.docx]

**É projeto FAPESP, pagam auxílio publicação.**

Frontiers in Immunology (procure alguma edição especial) (5.9 IF): <https://www.frontiersin.org/journals/immunology>

Frontiers in Nutrition (procure alguma edição especial) (5.1 IF): https://www.frontiersin.org/journals/nutrition

Scientific Reports (procure alguma edição especial) (3.9 IF): <https://www.nature.com/srep/>

Frontiers in Medicine (procure alguma edição especial) (3 IF): <https://www.frontiersin.org/journals/medicine>

BMC Nutrition (procure alguma edição especial) (2.2 IF): https://bmcnutr.biomedcentral.com/
