# Supplementary material for: Impact of creatine supplementation on inflammation: evidence from a systematic review and meta-analysis of randomized double-blind placebo trials
Source: Front Immunol. 2026 Feb 19;17:1743603. doi: 10.3389/fimmu.2026.1743603 (PMC12961398; doi:10.3389/fimmu.2026.1743603)
Supplement: Supplementary file 2 [file SupplementaryFile1.zip › SR Creatine inflammatory markers (Kell Doutorado). /Para submeter/Manuscript.docx]

**Impact of Creatine Supplementation on Inflammation: Evidence from a Systematic Review and Meta-Analysis of Randomized Double-Blind Placebo Trials**

Kell Mazzini Ribeiro de Camargo^1^, Alejandro Bruna-Mejías^2^, Juan José Valenzuela^2^, Luana A. Gonzaga^1^, Sandra Maria Barbalho^3, 4, 5^, Alexandre L. Barroca^1^, Andrey A. Porto^1^, Rodrigo D. Raimundo^8^, Luiz Carlos de Abreu^9,10^, Vitor E. Valenti^1^

^1^Systematic Reviews Center for Cardiovascular and Metabolic Health, São Paulo State University, School of Philosophy and Sciences, Marília, SP, Brazil. ^2^Departamento de Ciencias y Geografía, Facultad de Ciencias Naturales y Exactas, Universidad de Playa Ancha, Valparaíso 2360072, Chile. ^3^Postgraduate Program in Structural and Functional Interactions in Rehabilitation, School of Medicine, Universidade de Marília (UNIMAR), Marília 17525-902, São Paulo, Brazil. ^4^Department of Biochemistry and Nutrition, School of Food and Technology of Marília (FATEC), Marília 17500-000, São Paulo, Brazil. ^5^Research Coordinator, UNIMAR Charity Hospital, Universidade de Marília (UNIMAR), Marília 17525-902, São Paulo, Brazil. ^6^Department of Biochemistry and Pharmacology, School of Medicine, Faculdade de Medicina de Marília. (FAMEMA), Marília 17519-030, São Paulo, Brazil. ^7^Department of Administration, Associate Degree in Hospital Management, Universidade de Marília (UNIMAR), Marília 17525-902, São Paulo, Brazil. ^8^Laboratório de Delineamento de Estudos e Escrita Científica, Centro Universitário FMABC, Santo André, SP, Brazil. ^9^University of Limerick - Sarsfield Ave Limerick, Limerick, IE, Ireland. ^10^Federal University of Vitoria, Vitoria, ES, Brazil.

***Corresponding author:** Vitor E. Valenti

Sao Paulo State University, UNESP.

Av. HyginoMuzzi Filho, 737

Marília, SP, Brazil - 17.525-000

E-mail: vitor.valenti@unesp.br

**ABSTRACT**

**Introduction:** Creatine supplementation is widely recognized for its ergogenic effects on strength and body composition. Recent studies have explored its potential anti-inflammatory properties, particularly in exercise-induced stress and aging-related chronic inflammation. However, results across randomized trials remain inconsistent. This systematic review and meta-analysis aimed to assess the effects of creatine supplementation on inflammatory biomarkers in human populations. **Methods:** A systematic review and meta-analysis were conducted following PRISMA 2020 guidelines and registered in PROSPERO (CRD420251027784). Eight randomized controlled trials were included, evaluating creatine supplementation (various dosages and durations) versus placebo in healthy individuals, athletes, and clinical populations. The primary outcomes were inflammatory markers, including C-reactive protein (CRP), interleukin-6 (IL-6), IL-1β, TNF-α, and prostaglandin E₂. Data extraction and risk of bias assessments were performed by two independent reviewers. The certainty of evidence was rated using the GRADE framework. **Results:** Pooled analysis showed no significant acute effects of creatine on CRP (SMD = 0.32; 95% CI: -0.29 to 0.94; p = 0.30; I² = 28%). Chronic effects of creatine on CRP (SMD = -0.11; 95% CI: -0.69 to 0.48; p = 0.73; I² = 0%) and IL-6 (SMD = -0.06; 95% CI: -0.64 to 0.53; p = 0.84; I² = 0%) were also no significant. The certainty of evidence was rated as moderate for all outcomes. Risk of bias varied, with missing outcome data being the most frequent limitation. **Conclusion:** Creatine supplementation does not significantly reduce inflammatory biomarkers in humans based on current evidence. Although certain benefits were observed under intense endurance conditions, results remain inconsistent across populations. Future well-powered trials with standardized protocols are needed to clarify creatine’s role in modulating inflammation.

**Keywords:** Creatine, Inflammation, Cytokines, IL-6, CRP, Meta-analysis, Supplementation

**INTRODUCTION**

Inflammation plays a pivotal role in both physiological adaptation and pathological processes. Inflammation is the response of living vascularized tissue to injury and can be triggered by microbial infections, physical agents, chemical substances, necrotic tissue, or immunological reactions. The goal of inflammation is to contain and isolate the injury, destroy invading microorganisms, and inactivate toxins, as well as prepare the tissue for healing and repair (Ferreo-Miliani et al, 2007).

Conversely, chronic low-grade inflammation is defined as a two to four-fold elevation in circulating pro-inflammatory markers, including C-reactive protein (CRP), tumor necrosis factor-α (TNF-α), and interleukin-6 (IL-6). This persistent inflammatory state is strongly associated with the aging process and contributes mechanistically to sarcopenia—the age-related reduction in muscle mass and strength (Ferrucci et al., 2005). Beyond aging, inflammation drives the progression of several chronic diseases: elevated CRP and TNF-α concentrations are associated with increased total knee pain in osteoarthritis (OA), and chronic inflammation can lead to metabolic disorders such as Type 2 Diabetes Mellitus (T2D) and cardiovascular diseases. However, the increase in IL-6 following exercise may play a beneficial role by mobilizing substrates for energy and enhancing insulin sensitivity, potentially protecting against disorders like T2D by inhibiting TNF-α production (Stannus et al., 2013). The clinical and performance implications of managing inflammation are critical, as exercise-induced muscle trauma results in pain, delayed onset muscle soreness (DOMS), reduced range of motion, and prolonged muscle strength loss, negatively impacting subsequent athletic performance (Bassit et al., 2008; Rawson et al., 2007).

In this context, creatine is a widely popular dietary supplement utilized as an ergogenic aid. Its well-established performance-enhancing effects are rooted in its fundamental role as a temporal and spatial energy buffer. Supplementation reliably increases total muscle creatine concentration, enhancing phosphocreatine (PCr) availability to facilitate ATP resynthesis during high-intensity exercise (Kreider et al., 2017). When combined with resistance training, Creatine reliably promotes strength and fat-free mass gains in diverse populations, including older adults (Devries et al., 2014). While creatine is widely recognized for its performance-enhancing properties, recent studies suggest it may also modulate inflammatory responses, especially following intense physical activity. Creatine is reported to be anti-inflammatory in nature, helping to maintain muscle integrity and attenuating inflammatory markers after strenuous exercise sessions (Bassit et al., 2008; Santos et al., 2004).

The precise mechanisms underlying creatine’s anti-inflammatory and cytoprotective effects remain to be definitively determined. However, several mechanisms have been proposed. One theory involves osmotic effects and cellular swelling; creatine increases intracellular water content (Sestili et al., 2006).

Current evidence regarding creatine’s potential anti-inflammatory properties remains inconsistent, highlighting significant knowledge gaps across different populations and protocols. Positive effects in athletes subject to high physiological stress have been frequently reported. For instance, creatine supplementation for five days prior to a half-ironman competition significantly reduced the exercise-induced increase in plasma levels of pro-inflammatory cytokines, including TNF-α, IFN-α, and IL-1β, as well as Prostaglandin E2 (PGE2), 24 and 48 hours post-competition (Bassit et al., 2008; Santos et al., 2004). Similarly, creatine supplementation attenuated the post-race increase in plasma TNF-α (by 33.7%) and PGE2 (by 60.9%), and abolished the increase in lactate dehydrogenase (LDH) activity following a strenuous 30 km race in marathon runners (Santos et al., 2004; Bassit et al., 2008).

However, null findings have limited the generalization of these effects to other populations or exercise types. In studies focused on chronic, low-grade inflammation, 12 weeks of creatine supplementation yielded no effect on inflammatory biomarkers (CRP, IL-1β, IL-6, TNF-α) in patients with mild to moderate knee osteoarthritis (OA) (Cornish & Peeler, 2018). Moreover, combining creatine supplementation (5 g/day for 12 weeks) with resistance training in community-dwelling older adults failed to provide additional benefits on systemic inflammation markers such as IL-6, IL-10, and CRP, compared to training with placebo (Oliveira et al., 2020). Creatine also failed to reduce muscle damage (assessed via strength, range of motion, soreness, and elevated creatine kinase activity) or enhance recovery following a resistance exercise challenge designed to be hypoxic in trained men. Several methodological limitations contribute to these discrepancies, as many studies are small-scale; for example, the study investigating creatine in OA included only 18 participants (Cornish & Peeler, 2018) and the half-ironman study included only 11 triathletes (Bassit et al., 2008; Rawson et al., 2007). Furthermore, the inflammatory markers assessed vary widely across trials, ranging from cytokines (IL-6, TNF-α, IL-1β, IFN-α) and pain mediators (PGE2) to muscle damage proxies (CK, LDH, CRP). Despite growing interest, current evidence on creatine’s impact on inflammation remains fragmented, with no consensus across populations or protocols.

The inconsistencies observed between trials that investigated acute exercise-induced inflammation (Bassit et al., 2008; Santos et al., 2004) and those addressing chronic inflammation (Cornish & Peeler, 2018; Oliveira et al., 2020) highlight a critical need for synthesizing the accumulated data (Cornish & Peeler, 2018). A systematic synthesis and meta-analysis is therefore warranted to rigorously aggregate findings from randomized controlled trials. Such an approach will permit a detailed evaluation of acute versus chronic creatine effects across different physiological states and population subsets (Rawson et al., 2007). Crucially, this effort must focus on objective inflammatory markers measured in human participants to clarify the clinical and physiological relevance of creatine's purported anti-inflammatory effects. A systematic review with meta-analysis is warranted to clarify whether creatine exerts clinically meaningful anti-inflammatory effects in humans, particularly in the context of exercise-induced and chronic inflammation.

Therefore, the present study aimed to systematically review and meta-analyze randomized controlled trials investigating the effects of creatine supplementation on inflammatory biomarkers in humans. We hypothesized that creatine would reduce levels of key inflammatory markers, particularly in response to acute exercise-induced stress (Bassit et al., 2008; Santos et al., 2004).

**METHODS**

**Protocol and Registration**

The review followed the guidelines outlined in the Preferred Reporting Items for Systematic Reviews and Meta-Analyses (PRISMA) (Page et al., 2021) and has been formally registered in the PROSPERO database (CRD420251027784).

**Eligibility Criteria**

The selected studies were sourced from peer-reviewed journals and were published from the inception of each database up to November 2025. The eligibility criteria were established based on the PICOS framework (Population, Intervention, Comparison, Outcomes, and Study Design), encompassing:

1. (P) Studies involving human participants of any age, sex, or health status (e.g., healthy individuals, athletes, or patients with clinical conditions). Exclusion criteria: Studies involving animals or in vitro models;
2. (I) Studies that administered creatine supplementation, regardless of dosage, duration, or form (e.g., creatine monohydrate, creatine ethyl ester), either alone or combined with exercise or other interventions. Exclusion criteria: Studies using multi-ingredient supplements where the independent effect of creatine cannot be determined;
3. (C) For comparison groups, we included studies that evaluated subjects that received placebo;
4. (O) Primary outcomes: Studies that assessed inflammatory markers (e.g., CRP, interleukins such as IL-6, IL-1β, TNF-α, etc.). Secondary outcomes: Blood glucose, cholesterol and tryglicerides. Exclusion criteria: Studies without available data on inflammatory markers;
5. (S) We included studies with single or double-blind randomized controlled trials and crossover designs. This review is restricted to articles published in peer-reviewed journals, master’s theses and doctoral dissertations. We excluded conference abstracts, descriptive studies, case reports, editorials, and reviews.

**Information Source, Search Strategy and Study Selection**

The literature search was conducted in the EMBASE, LILACS, CINAHL, MEDLINE/PubMed (via the National Library of Medicine), Cochrane Library, Scopus, and Web of Science databases. The search strategy included the following terms: "Creatine Supplement" OR "Creatine monohydrate supplementation" OR "Creatine supplementation" AND "Inflammation" OR "Cytokine" OR "Interleukin" (full strategies available in the Supplementary File).

All retrieved records were exported to Rayyan QCRI (Qatar Computing Research Institute, Qatar) for automatic duplicate removal. Title and abstract screening was carried out in Rayyan by at least two independent reviewers, followed by full-text screening. In cases of disagreement, a third reviewer adjudicated the final decision. After selecting the eligible studies, the research team collectively evaluated whether a meta-analysis was feasible.

**Data Collection and Data Extraction**

Information on authorship, study design, participant characteristics, intervention details, and exercise protocols was extracted and summarized in a structured table. Missing information was requested directly from corresponding authors. When no response was received, numerical data presented only in figures were extracted using WebPlotDigitizer®. Data were expressed as means and standard deviations (SD). When studies reported standard error (SE) or confidence intervals (CI), these values were converted to SD.

**Data Items**

We extracted data related to inflammatory biomarkers to compare outcomes between intervention and control groups. Additional information regarding participant characteristics, intervention protocols, and funding sources were obtained from the included studies. Variables that were unclear or not reported were excluded from further analysis.

**Assessment of the Risk of Bias in Individual Studies and Across Studies**

Risk of bias was assessed using the Cochrane Risk of Bias 2.0 tool (Sterne et al., 2019) in Review Manager (RevMan 5.4.1). The tool evaluates six domains:

1. Randomization process
2. Deviations from intended interventions
3. Missing outcome data
4. Outcome measurement
5. Selection of reported results
6. Overall bias

Each domain was rated as “low risk,” “some concerns,” or “high risk.” Two independent reviewers completed the assessment, and disagreements were resolved by consulting a third reviewer. All assessors completed prior training in risk-of-bias evaluation. Potential sources of bias at the study and review level, such as publication bias and selective reporting, were also considered.

**Certainty Assessment (Levels of Evidence)**

The certainty of evidence was appraised using the GRADE (Grading of Recommendations, Assessment, Development and Evaluation) approach (GRADE Working Group, 2004). Factors considered included study design, methodological quality, precision of estimates, and consistency across studies (Meader et al., 2014). The GRADEpro GDT v4® software (McMaster University, Canada) was used to generate the Summary of Findings table.

**Qualitative Analysis (Systematic Review)**

Study characteristics and findings were described in text and tables, with emphasis on cardiovascular and inflammatory outcomes in both intervention and control conditions.

**Synthesis of Results and Summary Measures**

When at least two studies provided comparable data, a meta-analysis was conducted. Only post-intervention values were included. Heterogeneity was quantified using the I² statistic, interpreted as follows:

- 0–29%: negligible heterogeneity
- 30–49%: moderate
- 50–74%: substantial
- 75–100%: considerable (Higgins et al., 2002; 2003)

If dispersion metrics (e.g., SD, 95% CI, SE, p-value) were not reported, SD of change scores was calculated when possible. Pooled effects were expressed as weighted mean difference (MD) with 95% confidence intervals. Statistical significance was set at p < 0.05. A random-effects model was applied due to expected methodological and clinical heterogeneity (Deeks et al., 2023). All analyses were conducted using RevMan 5.4.1.

**RESULTS**

**Study Selection**

A total of 789 records were identified through database searches. After removing 212 duplicates, 577 unique records were screened according to the inclusion criteria. Following the screening of titles and abstracts, 562 records were excluded. Fifteen studies were then selected for full-text retrieval, one of which could not be retrieved. The remaining 14 studies were assessed for eligibility through full-text reading. Six studies were excluded for the following reasons: data not published (n=1), population under 18 years of age (n=1), article type was a review (n=1), study had no placebo group (n=1), and no outcome of interest was reported (n=2). Consequently, eight studies were included in the final review. The search methods and study selection process were conducted in accordance with the PRISMA statement, as illustrated in Figure 1.

**Results of Individual Studies**

This analysis focused on the impact of creatine supplementation on inflammatory markers. The reviewed studies demonstrated mixed effects depending on the population and experimental conditions.

Santos et al. (2004) reported that short-term creatine supplementation (20 g/day for 5 days) significantly attenuated inflammatory responses following a 30-kilometer race in male athletes. Specifically, creatine reduced post-race increases in prostaglandin E₂ (PGE₂) by 60.9% and tumor necrosis factor-α (TNF-α) by 33.7%, suggesting a protective effect against exercise-induced muscle damage and systemic inflammation. Similarly, Bassit et al. (2008), using a similar protocol (20 g/day for 5 days), found that creatine supplementation in male triathletes led to significantly lower levels of TNF-α, interleukin-1β (IL-1β), and PGE₂ after a half-Ironman triathlon. These findings reinforce the anti-inflammatory potential of creatine during prolonged and intense endurance activities, potentially via modulation of cytokine responses to physiological stress.

In contrast, several studies failed to observe such benefits in other contexts. Cornish and Peeler (2018) administered creatine supplementation (20 g/day for 1 week followed by 5 g/day for 11 weeks) to patients with mild to moderate knee osteoarthritis and found no significant changes in inflammatory markers, including CRP, IL-1β, IL-6, s100 A8/A9, and TNF-α. This suggests that creatine may not confer anti-inflammatory effects in chronic low-grade inflammatory conditions. Similarly, Oliveira et al. (2020) conducted a 12-week randomized trial in older adults (mean age 67 years) using a daily dose of 5 g of creatine monohydrate combined with resistance training. The results showed no significant differences between the creatine and placebo groups in key inflammatory markers such as IL-6, IL-10, adiponectin, leptin, or CRP, although both groups experienced reductions in MCP-1, indicating a potential effect of training itself rather than creatine.

Deldicque et al. (2008) investigated the molecular effects of creatine at the gene expression level in young healthy men after just 5 days of supplementation (21 g/day) combined with acute resistance exercise. Although they observed increases in gene expression of muscle-related targets such as collagen-1, GLUT-4, and myosin heavy chains, no modulatory effect of creatine was found on IL-6 mRNA expression, indicating that creatine did not influence local inflammatory gene responses in muscle tissue under these conditions. Likewise, Rawson et al. (2007) evaluated creatine's effects on recovery from hypoxic resistance exercise in trained men over a 10-day supplementation protocol and reported no reduction in markers of muscle damage or inflammation. Notably, neither lactate dehydrogenase nor CRP increased following the exercise protocol, and creatine had no measurable effect on recovery outcomes.

Tarnopolsky et al. (2007) further extended these findings to an older population undergoing 6 months of resistance training. In this trial, creatine (5 g/day) combined with conjugated linoleic acid did not result in any significant changes in IL-6 or CRP levels when compared to placebo, although improvements were observed in body composition and strength parameters. This highlights a potential disconnect between functional improvements and systemic inflammatory markers.

**Synthesis of Results**

In relation to the acute effects of creatine on CRP, the overall pooled results showed a standardized mean difference (SMD) of 0.32 (95% CI: -0.29, 0.94), indicating no statistically significant difference between the creatine and placebo groups (Z = 1.03, P = 0.30). The analysis exhibited low heterogeneity (I² = 28%) (Figure 2), suggesting consistency in the direction and magnitude of the effect across the two included studies.

Examining the individual studies, the effect size for Rawson et al., 2007 was an SMD of -0.05 (95% CI: -0.69, 0.78), showing no significant benefit of creatine. In contrast, the study by Tarnopolsky et al., 2007 showed a larger, though still non-significant, positive effect in favor of creatine, with an SMD of 0.56 (95% CI: -0.08, 1.23) (Figure 2). In conclusion, based on the available evidence, creatine supplementation does not demonstrate a statistically significant acute effect compared to placebo.

The meta-analysis also assessed the chronic effect of creatine supplementation on inflammatory biomarkers, including creatine kinase (CK), CRP, and IL-6, across two studies (Figure 3).

For CRP, the meta-analysis included 45 participants from the two studies (Cornish & Peeler, 2018; Oliveira et al., 2020). The results showed a standardized mean difference (SMD) of -0.11 (95% CI: -0.69, 0.48), indicating no statistically significant difference between the creatine and placebo groups (Z = 0.35, P = 0.73). No heterogeneity was observed (I² = 0%) (Figure 3), suggesting consistent results across the studies.

In the case of IL-6, the analysis also included 45 participants. The SMD was -0.06 (95% CI: -0.64, 0.53), indicating no significant effect of creatine supplementation on CRP levels (Z = 0.20, P = 0.84). The analysis again exhibited no heterogeneity (I² = 0%) (Figure 3).

**Risk of Bias**

The risk of bias varied across the included studies, with concerns identified in several domains, including randomization, deviations from intended interventions, missing outcome data, outcome measurement, and selection of reported results. Overall, the studies demonstrated a mixture of low risk, some concerns, and high risk of bias (Figure 4).

*Randomization Process*
 All studies adequately described the randomization process and were judged as low risk for this domain. Randomization procedures appeared to have been properly implemented, with no evidence of selection bias or systematic differences between intervention groups at baseline.

*Deviations from Intended Interventions*

Across all studies, the risk of bias due to deviations from intended interventions was judged to be low. Most studies employed double-blind or placebo-controlled designs, minimizing the likelihood that participants’ or researchers’ awareness of the assigned interventions influenced the outcomes.

*Missing Outcome Data*

High risk were identified in Cornisha and Peeler 2018, Oliveira et al, 2020 and Santos et al 2004, where participant losses or incomplete reporting were not fully explained. The remaining studies either reported complete datasets or provided adequate justification for missing data, suggesting that attrition was unlikely to have affected the results.

*Measurement of Outcomes*

All studies (100%) were rated as low risk for this domain. Outcome measures were obtained using validated and standardized procedures appropriate for the interventions. Although blinding of assessors was not explicitly described in all studies, measurement bias was considered minimal.

*Selection of Reported Results*

Most studies (Bassit et al, 2008; Cornisha and Peeler 2018; Deldicque et al, 2008; Santos et al, 2004 and Taes et al, 2004) raised some concerns regarding selective reporting, as pre-specified protocols or analysis plans were not always clearly available. Despite this, reported outcomes were generally consistent with study aims and expected endpoints.

*Overall Risk of Bias*

**GRADE Assessment**

The GRADE assessment indicated that the overall quality of evidence regarding the effects of creatine supplementation on inflammatory markers was moderate, though limited by concerns related to risk of bias (Table 2). Specifically, the domains of inconsistency, indirectness, and imprecision were not considered serious, suggesting reasonable consistency and directness across studies. However, the presence of missing outcome data led to a classification of *very serious risk of bias* for all evaluated outcomes.

• CRP (acute effects): Moderate certainty

• CRP (chronic effects): Moderate certainty

• IL-6 (chronic effects): Moderate certainty

Detailed explanations for these GRADE ratings, including considerations of data completeness and study design, are provided in the supplementary material.

**Heterogeneity** The moderate heterogeneity observed among studies assessing inflammatory outcomes may be attributed to methodological and clinical differences. These include variations in supplementation duration (ranging from short-term to several weeks), participant characteristics (e.g., trained athletes versus older adults), and exercise protocols (endurance versus resistance training). Moreover, some trials did not provide detailed information about blinding or adherence to supplementation, potentially influencing the magnitude of observed effects. Despite these discrepancies, the overall direction of the results was consistent, indicating a potential anti-inflammatory effect of creatine supplementation under different experimental conditions.

**DISCUSSION**

**Summary of Key Findings**

Our systematic review with meta-analysis aimed to evaluate the effects of creatine (Cr) supplementation on inflammatory markers in human populations. As key findings, we observed that: Creatine supplementation did not consistently reduce biomarkers associated with chronic low-grade inflammation, specifically CRP or IL-6, across diverse clinical or elderly populations. For instance, plasma IL-6 concentrations were not significantly affected by creatine supplementation after an exhaustive competition (Bassit et al., 2008), nor did creatine plus resistance training (RT) provide additional benefits on IL-6 or CRP in older adults compared to RT alone. While some individual trials showed benefit in athletes under high physiological stress, results were inconsistent or absent in older adults and clinical populations. In fact, short-term creatine failed to reduce muscle damage markers like creatine kinase (CK) or muscle soreness following hypoxic resistance exercise in trained men. Overall certainty of evidence for specific chronic markers in specific populations appears limited, and risk of bias concerns were present in several small-scale studies.

**Physiological Mechanisms of Creatine and Inflammation**

The anti-inflammatory effects observed in specific contexts suggest several underlying mechanisms, although the precise mechanism remains undetermined (Bassit et al., 2008; Cornish & Peeler, 2018). One of the primary proposed mechanisms is related to cytoprotective effects on muscle cells post-exercise. Creatine loading increases muscle intracellular water content, promoting cell swelling. This may increase muscle cell resistance to mechanical injury, thereby reducing cell death and mitigating the ensuing inflammatory process as a whole (Doma et al 2022; Cella et al 2020). Supporting this, studies demonstrating efficacy noted that creatine supplementation abolished the increase in lactate dehydrogenase (LDH), a marker of cell death/lysis, following high-intensity running (Santos et al., 2004).

Creatine is also suggested to modulate the release of inflammatory mediators through downregulation of pro-inflammatory cytokine production (Bassit et al., 2008; Santos et al., 2004). Creatine effectively reduced plasma levels of TNF-α, IFN-α, and IL-1β, in addition to reducing Prostaglandin E2 (PGE2) following acute strenuous exercise (Bassit et al., 2008; Santos et al., 2004). Furthermore, *in vitro* research has indicated that creatine reduces neutrophil adhesion by downregulating adhesion molecules (Nomura et al., 2003), potentially through the activation of adenosine A2A receptors resulting from altered ATP and phosphocreatine concentrations (Cornish & Peeler, 2018).

Finally, reduced oxidative stress via its demonstrated direct antioxidant properties may contribute to anti-inflammatory benefits (Lawler et al., 2002). However, these anti-inflammatory properties appear to be highly context-dependent (Oliveira et al., 2020). The failure to observe reductions in inflammatory biomarkers during chronic low-grade inflammation (e.g., in knee osteoarthritis (OA) or aging) suggests that creatine's protective capacity may primarily target the acute, high-magnitude tissue damage inherent in intensive endurance performance, rather than complex systemic inflammation (Cornish & Peeler, 2018; Oliveira et al., 2020).

**Contextualization with Prior Literature**

Our findings partially align with studies reporting anti-inflammatory effects in athletes subject to extreme physiological exertion. Specifically, creatine consumption attenuated the increase in plasma TNF-α and PGE2 after a 30 km race (Santos et al., 2004) and reduced plasma levels of pro-inflammatory cytokines (TNF-α, IFN-α, IL-1β) after a half-ironman competition (Bassit et al., 2008). These trials support the view that creatine is effective against robust exercise-induced inflammation (Bassit et al., 2008).

However, our findings diverge sharply when considering chronic low-grade inflammation or specific resistance exercise contexts. For example, 12 weeks of creatine supplementation showed no significant impact on inflammatory biomarkers (CRP, IL-6, TNF-α, IL-1β, s100 A8/A9) or cartilage degradation markers (sCOMP) in patients with mild to moderate knee OA (Cornish & Peeler, 2018). Similarly, trials involving older adults reported that 12 weeks of creatine supplementation combined with resistance training did not provide additional improvement on markers of inflammation (IL-6, IL-10, CRP) or insulin resistance compared to placebo plus training (Oliveira et al., 2020). Furthermore, the lack of benefit noted after a hypoxic resistance challenge (Rawson et al., 2007) suggests that creatine's protective effects seen in running protocols (Santos et al., 2004) do not generalize to resistance exercise protocols designed to induce muscle damage (Rawson et al., 2007). This highlights that the efficacy of creatine as an anti-inflammatory agent relies heavily on the type and intensity of the physiological stressor (Rawson et al., 2007).

### Contrast of Results Across Subgroups

The overall impact of creatine (Cr) supplementation on inflammatory biomarkers appears highly context-dependent, demonstrating contrasting results across different study populations, exercise modalities, and intervention durations.

Population Type: Positive anti-inflammatory effects were consistently reported in trained endurance athletes subjected to extreme physiological stress. For example, Creatine supplementation significantly reduced pro-inflammatory cytokines (TNF-α, IFN-α, IL1-β) and PGE2 following a half-ironman competition (Bassit et al., 2008) and attenuated TNF-α and PGE2 after a 30 km race (Santos et al., 2004). However, studies involving clinical patients or older adults with chronic low-grade inflammation typically reported null findings. Twelve weeks of supplementation had no effect on inflammatory biomarkers (CRP, IL-6, TNF-α, IL-1β, s100 A8/A9) in patients with mild to moderate knee osteoarthritis (Cornish & Peeler, 2018). Similarly, 12 weeks of Creatine combined with resistance training did not provide additional benefits on chronic inflammatory markers (IL-6, IL-10, CRP) in community-dwelling older adults, although Monocyte Chemoattractant Protein-1 (MCP-1) was reduced by the exercise intervention alone (Oliveira et al., 2020).

In relation to exercise modality, the greatest benefits were observed in endurance or high-volume eccentric exercise protocols (Bassit et al., 2008; Santos et al., 2004). In contrast, studies using resistance exercise protocols designed to induce muscle damage (e.g., squat exercise under hypoxic conditions or high-force eccentric exercise) generally found that Creatine supplementation failed to reduce markers of muscle damage or inflammation (e.g., CK, soreness, CRP) compared to placebo (Rawson et al., 2007).

Supplementation Duration and Dose: Acute loading phases (typically 5 days of 20 g/day) prior to a single bout of strenuous exercise demonstrated efficacy in attenuating the inflammatory response (Bassit et al., 2008; Santos et al., 2004). Conversely, chronic supplementation protocols (e.g., 12 weeks of 5 g/day maintenance dose) showed no significant reduction in chronic systemic inflammation markers like CRP and IL-6 (Cornish & Peeler, 2018; Oliveira et al., 2020). This suggests that Creatine may be primarily effective as a cytoprotective agent against acute, high-magnitude tissue trauma rather than modulating sustained chronic low-grade inflammation (Cornish & Peeler, 2018).

### Meta-Analysis Interpretation

Assuming a meta-analysis focused on key chronic markers (CRP and IL-6), the pooled effect size would likely be interpreted as statistically insignificant.

SMDs, CIs, and Heterogeneity (I²): In a meta-analysis, the standardized mean difference (SMD) represents the magnitude of the intervention effect across studies using different measurement scales. Confidence intervals (CIs) define the range within which the true pooled effect likely lies. Heterogeneity, often quantified by the I² statistic, describes the proportion of total variation in study estimates that is due to genuine differences in the true effects between studies, rather than just sampling error (Higgins & Thompson, 2002). An I² value is considered more useful than the basic test for heterogeneity, as I² does not intrinsically depend on the number of studies (Higgins & Thompson, 2002).

Acute CRP and Chronic CRP/IL-6: Meta-analysis focusing on CRP and IL-6 across chronic (long-term) studies would reveal no significant effect of creatine supplementation (Cornish & Peeler, 2018; Oliveira et al., 2020). Given the consistent null findings for these specific markers in chronic, low-grade inflammatory conditions (Cornish & Peeler, 2018; Oliveira et al., 2020), the I² for these outcomes would likely be low, suggesting consistent results.

Divergence from Individual Studies: The pooled estimates showing a lack of chronic effect may differ from promising individual studies focused on acute outcomes (Santos et al., 2004; Bassit et al., 2008). This divergence arises because meta-analysis pools heterogenous populations and outcomes. For instance, Creatine significantly reduced TNF-α, IL-1β, IFN-α, and PGE2 in healthy triathletes (Bassit et al., 2008; Santos et al., 2004), effects that are highly relevant to muscle trauma (Bassit et al., 2008). However, when these effects are pooled with data from older adults whose inflammation is linked to metabolic disease rather than eccentric exercise (Oliveira et al., 2020), the overall effect on systemic markers like IL-6 and CRP might become negligible.

### GRADE and Risk of Bias Interpretation

The certainty of the overall evidence, potentially assessed using tools like GRADE (The GRADE Working Group, 2017 cited in PRISMA 2020), would likely be rated as moderate to low. This lower certainty is partly due to missing outcome data and the inconsistent protocols used across trials. Many included studies were small-scale (e.g., N=5 in one group in the half-ironman study (Bassit et al., 2008), N=9 in the OA study (Cornish & Peeler, 2018)), limiting statistical power (Rawson et al., 2007).

While most studies were double-blinded and employed appropriate randomization methods (Cornish & Peeler, 2018; Bassit et al., 2008; Rawson et al., 2007), selective outcome reporting and attrition (loss of subjects) were notable limitations (Cornish & Peeler, 2018). For example, the study on knee OA patients was limited by its small sample size (Cornish & Peeler, 2018), which may have influenced the ability to detect significant differences (Cornish & Peeler, 2018). High variability in biomarker results also limited the findings (Cornish & Peeler, 2018).

### Clinical Implications

The practical application of creatine as an anti-inflammatory agent is currently compartmentalized by context:

With respect to athletes (especially in Endurance Events), creatine supplementation has demonstrable benefits in reducing acute exercise-induced inflammation, muscle damage (LDH), and pain mediators (PGE2) following exhaustive, long-distance events (Santos et al., 2004; Bassit et al., 2008). Thus, creatine is a valuable tool for maintaining muscle integrity and accelerating recovery in this population (Bassit et al., 2008; Santos et al., 2004; Rawson et al., 2007).

In relation to older adults or those with chronic inflammation: Creatine alone is not supported as an intervention to reduce established chronic inflammatory markers (CRP, IL-6, TNF-α) in conditions like knee osteoarthritis or general aging (Cornish & Peeler, 2018; Oliveira et al., 2020; Tarnopolsky et al., 2007). While creatine combined with resistance training offers significant benefits in improving fat-free mass (FFM) and strength in older adults (Tarnopolsky et al., 2007), the hypothesized anti-inflammatory mechanism does not appear to operate reliably in chronic disease settings (Cornish & Peeler, 2018).

Caution must be exercised to avoid overgeneralizing the positive results observed in specific acute exercise models to chronic disease management (Rawson et al., 2007; Cornish & Peeler, 2018).

### Study Limitations

A major limitation of the overall evidence base stems from high heterogeneity in study designs, encompassing vastly different populations, physiological states, and dosing protocols (Cornish & Peeler, 2018).

Small Sample Sizes in Individual RCTs: Many foundational trials suffered from small subject numbers, limiting statistical power to detect meaningful differences (e.g., the half-ironman study used N=11 total triathletes (Bassit et al., 2008)).

Few Studies Evaluated Multiple Inflammatory Markers Simultaneously: While some studies examined comprehensive cytokine panels (Bassit et al., 2008), many focused only on one or two specific markers (e.g., CK or CRP), potentially missing broader effects (Rawson et al., 2007).

Lack of Long-Term Trials in Clinical Populations: The absence of extended trials (e.g., 6 months to 1 year) in clinical groups makes it difficult to assess the long-term impact of creatine on disease progression (Cornish & Peeler, 2018).

Absence of Standardized Outcome Reporting Protocols: The lack of consistent reporting methods hinders comparative analysis across studies.

**Sources of Heterogeneity impacting meta-analysis frequently include:**

The following addresses the heterogeneity present across the randomized controlled trials included in the analysis, based exclusively on the descriptive information provided in the sources.

## Sources of Heterogeneity

The synthesis of evidence is significantly impacted by profound heterogeneity in study populations and activity levels. Participants ranged widely from young, highly trained male athletes, such as triathletes (mean age: 40.3 ± 2.18 years) (Bassit et al, 2008) and marathon runners (mean age: 25.5 ± 3.2 years) (Santos et al, 2004), to older adults (mean age: 67 ± 5 years) (Oliveira et al, 2020) and clinical populations with pre-existing conditions like mild to moderate knee osteoarthritis (mean age: 57.1 ± 7.4 years) (Cornisha & Peeler, 2018) or hemodialysis patients (Marini et al, 2019; Taes et al, 2004; Marini et al, 2024). The median age across these studies spans from young men (21.7 ± 0.55 years) (Deldicque et al, 2008) up to 70 ± 10 years in hemodialysis patients (Taes et al, 2004).

Furthermore, the exercise protocols varied fundamentally by intensity and type, targeting either acute trauma or chronic adaptation. Studies induced extreme endurance stress (e.g., half-ironman competition or a 30 km race) (Bassit et al, 2008; Santos et al, 2004), tested recovery following high-volume resistance exercise protocols designed to be hypoxic (Rawson et al, 2007), or involved long-term supervised resistance training (Tarnopolsky et al, 2007; Oliveira et al, 2020). Conversely, some trials involved patients with no specific exercise training added to the creatine intervention (Cornisha & Peeler, 2018; Taes et al, 2004; Marini et al, 2019; Marini et al, 2024).

Dosage and Duration varied from short acute loading phases (e.g., 5 consecutive days of 20 grams/day) prior to a competition (Bassit et al, 2008; Santos et al, 2004) or a short study duration of 5 days (Deldicque et al, 2008), to chronic supplementation lasting 12 weeks (Cornisha & Peeler, 2018; Oliveira et al, 2020) and up to 6 months (Tarnopolsky et al, 2007) or 12 months (Marini et al, 2024). Daily doses varied dramatically, including low fixed maintenance doses of 2 grams/day (Taes et al, 2004) or 5 grams/day (Oliveira et al, 2020; Tarnopolsky et al, 2007; Marini et al, 2024), intermediate fixed doses of 20 grams/day (Bassit et al, 2008; Santos et al, 2004), and very high doses of 60 grams/day used in a 3-day acute loading phase (Irandoust et al, 2022). Furthermore, dosing was sometimes adjusted based on body weight, such as 0.3 g/kg body weight/day during a 5-day loading phase (Rawson et al, 2007).

Timing and Co-Intervention also differed. Creatine was typically administered in multiple equal doses daily (Bassit et al, 2008; Rawson et al, 2007; Santos et al, 2004). In exercise trials, supplements were sometimes consumed immediately after training sessions (Oliveira et al, 2020), while in others, they were administered daily in the evening (Taes et al, 2004). Notably, one major study combined creatine with conjugated linoleic acid (CLA) (Tarnopolsky et al, 2007), introducing a confounding factor when evaluating the independent effect of creatine.

The outcomes of inflammatory marker measurement showed extensive variability, hindering direct comparison of the anti-inflammatory effects of creatine. Studies focused on different physiological aspects of inflammation:

Acute Cytokines and Mediators: Several studies quantified immediate inflammatory and muscle damage responses in plasma, reporting markers like Tumor Necrosis Factor-α (TNF-α), IL-1β, IL-6, Prostaglandin E2 (PGE2), and acute muscle damage indicators such as creatine kinase (CK) and lactate dehydrogenase (LDH) (Bassit et al, 2008; Santos et al, 2004; Rawson et al, 2007; Cornisha & Peeler, 2018).

Chronic Systemic Markers: Other studies focused on markers typically associated with chronic low-grade inflammation, such as CRP, IL-6, IL-10, and Monocyte Chemoattractant Protein-1 (MCP-1)(Cornisha & Peeler, 2018; Oliveira et al, 2020; Tarnopolsky et al, 2007).

Unique and Molecular Outcomes: Specialized markers were also assessed, including serum cartilage oligomeric matrix protein (sCOMP) (a cartilage degradation marker) (Cornisha & Peeler, 2018), and markers of systemic stress like the Malnutrition-Inflammation Score (MIS) (Marini et al, 2019). Crucially, one study investigated molecular mechanisms by measuring gene expression (e.g., MHC I, MHC IIA, IL-6 mRNA) and signaling pathways in muscle tissue via biopsiesand real-time PCR, rather than just plasma concentrations (Deldicque et al, 2008).

### Suggestions for Future Research

Future research must prioritize methodological rigor to overcome current limitations:

There is a crucial need for large-scale, double-blind RCTs employing standardized dosing and standardized outcome reporting (Cornish & Peeler, 2018).

Studies should assess comprehensive biomarker panels (e.g., TNF-α, CRP, IL1-β, IL-6) rather than relying on single markers to capture the full scope of anti-inflammatory activity (Cornish & Peeler, 2018; Bassit et al., 2008).

**Analogy for Context-Dependent Effects**

Creatine's effect on inflammation is like a specialized fire extinguisher. It is highly effective at immediately putting out intense, acute fires caused by extreme physiological stress (strenuous endurance exercise), protecting the cells from immediate damage (Santos et al., 2004; Bassit et al., 2008). However, it appears much less effective when dealing with the slow, smoldering structural decay of chronic, low-grade inflammation associated with aging or chronic diseases, where different underlying mechanisms are driving the problem (Cornish & Peeler, 2018; Oliveira et al., 2020).

**CONCLUSION**

In summary, the current body of evidence does not support a consistent anti-inflammatory effect of creatine supplementation in humans, particularly regarding chronic low-grade inflammation markers such as CRP and IL-6. While short-term loading protocols in endurance athletes demonstrate a reduction in exercise-induced cytokine release and muscle damage mediators, these effects do not translate to older adults, clinical populations, or long-term supplementation settings. The certainty of evidence is constrained by small sample sizes, heterogeneous biomarker panels, variability in dosing regimens, and inconsistent reporting of inflammatory outcomes. Future randomized controlled trials should prioritize larger cohorts, standardized inflammatory endpoints, harmonized supplementation protocols, and population-specific hypotheses (e.g., acute vs. chronic inflammation, endurance vs. resistance stress). Such methodological refinement is essential to clarify whether creatine acts as a targeted modulator of acute inflammatory stress or if its potential benefits extend to chronic inflammatory states with clinical relevance.

**ACKNOWLEDGEMENTS**

We sincerely thank São Paulo State University (UNESP) for providing essential infrastructure and financial support, which were instrumental in conducting this study. Their commitment to fostering scientific research has been invaluable to our work. We acknowledge that Artificial Intelligence tools were used to assist in the development of this work. Specifically, AI was utilized to help generate ideas, refine the structure of the text, and review the English language for clarity and accuracy. All content was critically reviewed and finalized by the authors to ensure its academic integrity and originality.

**FUNDING**

Dr. Vitor E. Valenti receives financial support from the National Council for Scientific and Technological Development, an entity linked to the Ministry of Science, Technology, Innovations and Communications from Brazil (Process number 302574/2021-2). The study received financial support from FAPESP (Process number 2025/06769-1).

**DATA AVAILABILITY**

The data from this study are available at: https://drive.google.com/drive/folders/1mvYpAUJRDS13FwibtT580t-ZEj-Nis__?

**REFERENCES**

1. Ferrero-Miliani L, Nielsen OH, Andersen PS, Girardin SE. Chronic inflammation: importance of NOD2 and NALP3 in interleukin-1beta generation. Clin Exp Immunol. 2007 Feb;147(2):227-35. doi: 10.1111/j.1365-2249.2006.03261.x.
2. Ferrucci L, Corsi A, Lauretani F, Bandinelli S, Bartali B, Taub DD, Guralnik JM, Longo DL. The origins of age-related proinflammatory state. Blood. 2005 Mar 15;105(6):2294-9. doi: 10.1182/blood-2004-07-2599. Epub 2004 Nov 30.
3. Stannus OP, Jones G, Blizzard L, Cicuttini FM, Ding C. Associations between serum levels of inflammatory markers and change in knee pain over 5 years in older adults: a prospective cohort study. Ann Rheum Dis. 2013 Apr;72(4):535-40. doi: 10.1136/annrheumdis-2011-201047.
4. Kreider RB, Kalman DS, Antonio J, Ziegenfuss TN, Wildman R, Collins R, Candow DG, Kleiner SM, Almada AL, Lopez HL. International Society of Sports Nutrition position stand: safety and efficacy of creatine supplementation in exercise, sport, and medicine. J Int Soc Sports Nutr. 2017 Jun 13;14:18. doi: 10.1186/s12970-017-0173-z.
5. Devries MC, Phillips SM. Creatine supplementation during resistance training in older adults-a meta-analysis. Med Sci Sports Exerc. 2014 Jun;46(6):1194-203. doi: 10.1249/MSS.0000000000000220.
6. Sestili P, Martinelli C, Bravi G, Piccoli G, Curci R, Battistelli M, Falcieri E, Agostini D, Gioacchini AM, Stocchi V. Creatine supplementation affords cytoprotection in oxidatively injured cultured mammalian cells via direct antioxidant activity. Free Radic Biol Med. 2006 Mar 1;40(5):837-49. doi: 10.1016/j.freeradbiomed.2005.10.035.
7. Page MJ, McKenzie JE, Bossuyt PM, Boutron I, Hoffmann TC, Mulrow CD, et al. The PRISMA 2020 statement: an updated guideline for reporting systematic reviews. BMJ [Internet]. 2021 Mar 29 [cited 2022 Oct 5];372. Available from: <https://pubmed.ncbi.nlm.nih.gov/33782057/>
8. Sterne JAC, Savović J, Page MJ, Elbers RG, Blencowe NS, Boutron I, et al. RoB 2: a revised tool for assessing risk of bias in randomised trials. BMJ. 2019 Aug 28;366:l4898. doi: 10.1136/bmj.l4898.
9. Meader N, King K, Llewellyn A, Norman G, Brown J, Rodgers M, Moe-Byrne T, Higgins JP, Sowden A, Stewart G. A checklist designed to aid consistency and reproducibility of GRADE assessments: development and pilot validation. Syst Rev. 2014 Jul 24;3:82. doi: 10.1186/2046-4053-3-82. PMID: 25056145; PMCID: PMC4124503.
10. Higgins JPT, Thompson SG. Quantifying heterogeneity in a meta-analysis. Stat Med [Internet]. 2002 Jun 15 [cited 2022 Oct 5];21(11):1539–58. Available from: <https://pubmed.ncbi.nlm.nih.gov/12111919/>
11. Higgins, J.P.; Thompson, S.G.; Deeks, J.J.; Altman, D.G. Measuring inconsistency in meta-analyses. BMJ 2003, 327, 557–560.
12. Deeks JJ, Higgins JPT, Altman DG (editors). Chapter 10: Analysing data and undertaking meta-analyses. In: Higgins JPT, Thomas J, Chandler J, Cumpston M, Li T, Page MJ, Welch VA (editors). Cochrane Handbook for Systematic Reviews of Interventions version 6.4 (updated August 2023). Cochrane, 2023. Available from [www.training.cochrane.org/handbook](http://www.training.cochrane.org/handbook)
13. Santos RV, Bassit RA, Caperuto ÉC, Costa Rosa LF. The effect of creatine supplementation upon inflammatory and muscle soreness markers after a 30km race. Life Sci. 2004;75(16):1917–24. doi: 10.1016/j.lfs.2003.11.038
14. Bassit RA, Curi R, Costa Rosa LF. Creatine supplementation reduces plasma levels of pro-inflammatory cytokines and PGE2 after a half-ironman competition. Amino Acids. 2008;35(2):425–31. doi: 10.1007/s00726-007-0582-4
15. Rawson ES, Conti MP, Miles MP. Creatine supplementation does not reduce muscle damage or enhance recovery from resistance exercise. J Strength Cond Res. 2007;21(4):1208–13. doi: 10.1519/00124278-200711000-00039
16. Tarnopolsky MA, Chilibeck PD, Tonson A, et al. Combined creatine monohydrate and conjugated linoleic acid supplementation improves strength and body composition following resistance exercise in older adults. Appl Physiol Nutr Metab. 2007;32(6):1225–35. doi: 10.1139/H07-103
17. Oliveira AS, da Silva RP, Cayres SU, et al. Effects of creatine supplementation associated with resistance training on inflammatory and metabolic markers in older adults: A randomized, double-blind, placebo-controlled trial. J Nutr Health Aging. 2020;24(5):514–20. doi: 10.1007/s12603-020-1356-0
18. Cornish SM, Peeler JD. No effect of creatine monohydrate supplementation on inflammatory and cartilage degradation biomarkers in individuals with knee osteoarthritis. Nutr Res. 2018;51:57–65. doi: 10.1016/j.nutres.2017.12.010
19. Deldicque L, Atherton P, Patel R, et al. Effects of resistance exercise with and without creatine supplementation on gene expression and cell signaling in human skeletal muscle. J Appl Physiol (1985). 2008;104(2):371–8. doi: 10.1152/japplphysiol.00873.2007
20. Taes YE, Delanghe JR, De Vriese AS, et al. Creatine supplementation does not influence plasma homocysteine levels in chronic hemodialysis patients. Nephron Clin Pract. 2004;98(3):c95–c101. doi: 10.1159/000080677
21. Doma K, Ramachandran AK, Boullosa D, Connor J. The Paradoxical Effect of Creatine Monohydrate on Muscle Damage Markers: A Systematic Review and Meta-Analysis. Sports Med. 2022 Jul;52(7):1623-1645. doi: 10.1007/s40279-022-01640-z.
22. Cella PS, Marinello PC, Borges FH, Ribeiro DF, Chimin P, Testa MTJ, Guirro PB, Duarte JA, Cecchini R, Guarnier FA, Deminice R. Creatine supplementation in Walker-256 tumor-bearing rats prevents skeletal muscle atrophy by attenuating systemic inflammation and protein degradation signaling. Eur J Nutr. 2020 Mar;59(2):661-669. doi: 10.1007/s00394-019-01933-6.
23. Mitchell, RN, Kumar, V, Abbas, AK. Robbins & Cotran Fundamentos de Patologia**.** 9. ed. Rio de Janeiro: GEN Guanabara Koogan, 2017. *E-book.* p.61. ISBN 9788595151796. Disponível em: https://app.minhabiblioteca.com.br/reader/books/9788595151796/.
24. Nomura A, Zhang M, Sakamoto T, Ishii Y, Morishima Y, Mochizuki M, Kimura T, Uchida Y, Sekizawa K. Anti-inflammatory activity of creatine supplementation in endothelial cells in vitro. Br J Pharmacol. 2003 Jun;139(4):715-20. doi: 10.1038/sj.bjp.0705316
25. Lawler JM, Barnes WS, Wu G, Song W, Demaree S. Direct antioxidant properties of creatine. Biochem Biophys Res Commun. 2002 Jan 11;290(1):47-52. doi: 10.1006/bbrc.2001.6164.

**TABLES LEGEND**

**Table 1.** Description of the characteristics of the study population of articles by author and year, sample, age (years), intervention, control and outcomes.

**Table 2.** Levels of evidence analysis via (GRADE Working Group, 2004).

**FIGURE LEGENDS**

**Figure 1.** PRISMA 2020 flow diagram for new systematic reviews which included searches of databases and registers only.

**Figure 2.** Meta-analysis for overall acute effects of creatine on C-reactive protein.

**Figure 3.** Meta-analysis for overall chronic effects of creatine on C-reactive protein (CRP) and interleukin-6 (IL-6).

**Figure 4.** Cochrane risk of bias tool.
