# Supplementary material for: Impact of creatine supplementation on inflammation: evidence from a systematic review and meta-analysis of randomized double-blind placebo trials
Source: Front Immunol. 2026 Feb 19;17:1743603. doi: 10.3389/fimmu.2026.1743603 (PMC12961398; doi:10.3389/fimmu.2026.1743603)
Supplement: Supplementary file 2 [file SupplementaryFile1.zip › SR Creatine inflammatory markers (Kell Doutorado). /Para submeter/Table 2 GRADE.docx]

**Table 2.** Levels of evidence analysis via (GRADE Working Group, 2004).

| **Outcome** | **No. of Studies** | **Risk of Bias** | **Inconsistency** | **Indirectness** | **Imprecision** | **Certainty of Evidence** |
| --- | --- | --- | --- | --- | --- | --- |
| **CRP acute effects** | 2 | Very Serious^a^ | Not serious | Not serious | Not serious | Moderate |
| **CRP chronic effects** | 2 | Very Serious^a^ | Not serious | Not serious | Not serious | Moderate |
| **IL-6 chronic effects** | 2 | Very Serious^a^ | Not serious | Not serious | Not serious | Moderate |

^a^. Missing outcome data
