# Supplementary material for: Impact of creatine supplementation on inflammation: evidence from a systematic review and meta-analysis of randomized double-blind placebo trials
Source: Front Immunol. 2026 Feb 19;17:1743603. doi: 10.3389/fimmu.2026.1743603 (PMC12961398; doi:10.3389/fimmu.2026.1743603)
Supplement: Supplementary file 2 [file SupplementaryFile1.zip › SR Creatine inflammatory markers (Kell Doutorado). /Supplementary Files/Review Protocol.docx]

**Impact of Creatine Supplementation on Inflammation: Evidence from a Systematic Review and Meta-Analysis**

Kell M. R. de Camargo^1^, Alejandro Bruna-Mejías^2^, Luana A. Gonzaga^1^, Sandra Maria Barbalho^3, 4, 5^, Lucas Fornari Laurindo^2,6,7^, Alexandre L. Barroca^1^, Andrey A. Porto^1^, David M. Garner^8^, Rodrigo D. Raimundo^9^, Vitor E. Valenti^1^

^1^Systematic Reviews Center for Cardiovascular and Metabolic Health, São Paulo State University, School of Philosophy and Sciences, Marília, SP, Brazil. ^2^Departamento de Ciencias y Geografía, Facultad de Ciencias Naturales y Exactas, Universidad de Playa Ancha, Valparaíso 2360072, Chile. ^3^Postgraduate Program in Structural and Functional Interactions in Rehabilitation, School of Medicine, Universidade de Marília (UNIMAR), Marília 17525-902, São Paulo, Brazil. ^4^Department of Biochemistry and Nutrition, School of Food and Technology of Marília (FATEC), Marília 17500-000, São Paulo, Brazil. ^5^Research Coordinator, UNIMAR Charity Hospital, Universidade de Marília (UNIMAR), Marília 17525-902, São Paulo, Brazil. ^6^Department of Biochemistry and Pharmacology, School of Medicine, Faculdade de Medicina de Marília. (FAMEMA), Marília 17519-030, São Paulo, Brazil. ^7^Department of Administration, Associate Degree in Hospital Management, Universidade de Marília (UNIMAR), Marília 17525-902, São Paulo, Brazil. ^8^Cardiorespiratory Research Group, School of Biological and Medical Sciences, Faculty of Health and Life Sciences, Oxford Brookes University, Headington Campus, Gipsy Lane, Oxford OX3 0BP, UK. ^9^Laboratório de Delineamento de Estudos e Escrita Científica, Centro Universitário FMABC, Santo André, SP, Brazil

***Corresponding author:** Vitor E. Valenti

Sao Paulo State University, UNESP.

Av. HyginoMuzzi Filho, 737

Marília, SP, Brazil - 17.525-000

E-mail: vitor.valenti@unesp.br

**METHODS**

**Protocol and Registration**

The review was reported according to the recommendations of the Preferred Reporting Items for Systematic Reviews and Meta-Analyzes (PRISMA) (Page et al, 2021) and is registered in the PROSPERO database (XXXXXXX).

**Eligibility Criteria**

The studies originated from peer-reviewed journals, published from the beginning of the database until June 2025. The inclusion and exclusion criteria were in agreement with the PICOS (Population, Intervention, Comparison, Outcomes and Study Design) elements, including:

1. (P) Studies involving human participants of any age, sex, or health status (e.g., healthy individuals, athletes, or patients with clinical conditions). Exclusion criteria: Studies involving animals or in vitro models;
2. (I) Studies that administered creatine supplementation, regardless of dosage, duration, or form (e.g., creatine monohydrate, creatine ethyl ester), either alone or combined with exercise or other interventions. Exclusion criteria: Studies using multi-ingredient supplements where the independent effect of creatine cannot be determined;
3. (C) For comparison groups, we will include studies that evaluated subjects that received placebo;
4. (O) Studies that assessed inflammatory markers (e.g., C-reactive protein [CRP], interleukins such as IL-6, IL-1β, TNF-α, etc.) as primary or secondary outcomes. Exclusion criteria: Studies without available data on inflammatory markers;
5. (S) We will include studies with single or double-blind randomized controlled trials and crossover designs. This review is restricted to articles published in peer-reviewed journals, master’s theses and doctoral dissertations. We will exclude conference abstracts, descriptive studies, case reports, editorials, and reviews.

**Information Source, Search Strategy and Study Selection**

The searches will be performed via EMBASE, Lilacs, CINAHL, MEDLINE/PubMed (via National Library of Medicine), Cochrane, Scopus, and Web of Science databases with the submission of the keywords "Creatine Supplement" OR "Creatine monohydrate supplementation" OR "Creatine supplementation" AND "Inflammation" OR "Cytokine" OR "Interleukin" (Supplementary file: Search strategy).

All articles acknowledged will be exported to the Rayyan QCRI program (Qatar Computing Research Institute, Qatar) to eliminate duplicates. The studies will be screened in the Rayyan program by reading the title and abstract. The suitability stage will be completed by at least two four independent reviewers by reading their entire articles. Another reviewer will be invited to give a decision if there is a disagreement concerning a study. After including the final references we will evaluate in group the possibility of meta-analysis.

**Data Collection and Data Extraction**

Data concerning the author, study design, features of the study participants, intervention, and exercise protocols of the respective studies will be extracted from primary studies and presented in Table. Missing data will be requested by contacting the corresponding study authors. This stage will be finished independently by at least two reviewers. When the author’s correspondent did not respond, the Web Plot Digitizer^®^ we will apply to extract the data presented in graphs. The data will be charted as mean and standard deviations (SD). Values presenting with “standard error” or “confidence intervals'' (CI) in the primary studies will be converted to SD.

**Data Items**

We collected data regarding inflammatory markers in order to compare outcomes after intervention. Data related to participant and intervention profile and funding sources were obtained from the selected references. Missing or unclear information was discarded.

**Assessment of the Risk of Bias in Individual Studies and Across Studies**

The analysis of bias will be produced by Risk of Bias tools originated in the Cochrane organization (Sterne et al., 2019) via the Review Manager program (RevMan 5.4.1). Risk of bias is a tool based on the domains. Its evaluation is split into six areas: "Randomisation process”, "Deviations from intended interventions", "Missing outcome data", "Measuring of the outcome", "Selection of the reported results" and "Overall bias". The cataloging will be split into three retorts: low risk, some concerns and, high risk. Our assumptions will consider on the table developed by (Sterne et al., 2019), "Reviewers' judgment and criteria for judgment." Two independent authors will complete the risk of bias analysis. A further researcher will be referred to if there are any inconsistencies in their verdicts.

We will identify variables that could influence the cumulative evidence, i.e. publication bias, selective reporting within studies, etc.

The assessors of the Risk of Bias were trained with appropriate sessions.

**Certainty Assessment (Levels of Evidence)**

We used the Grades of Recommendation, Assessment, Development, and Evaluation (GRADE) Working Group (GRADE Working Group, 2004) to gauge the certainty of the evidence. This analysis comprises the study design of randomized trials (strong evidence). We likewise considered study quality (detailed study methods and execution) and limitations in the strength of evidence analysis (Meader et al, 2014). We performed the GRADEpro GDT v4^®^ (McMaster University, Ontario, Canada) to elaborate the summary of the findings table.

**Qualitative Analysis (Systematic Review)**

A narrative synthesis was implemented to describe detailed data on how each study was completed. The details for each study were introduced in texts and tables. The results of the individual qualitative analysis per study were completed by analyzing cardiovascular parameters for the intervention or control protocols.

**Synthesis of Results and Summary Measures**

After selecting all references, we will evaluate the possibility of meta-analysis. In a positive situation, we will introduce the outcome values. The information required to construct the meta-analysis is the post-intervention period. We will adopt the criterion of extracting all data offered between groups in post intervention.

Heterogeneity will be calculated via the I² statistic. We will interpret 0%-29%: not be important; 30%-49%: moderate heterogeneity; 50%-74%: substantial heterogeneity; 75%-100%: considerable heterogeneity (Higgins et al, 2002; Higgins et al., 2003). For the "95% CI" and "Test for overall effect size" values, significant differences will be assumed for p<0.05 (or, <5%). If the studies did not provide dispersion values of change such as SD, 95% CI, standard errors, or p-values, the missing SDs of the changes (SDchanges) will be calculated. Outcomes of the meta-analysis will be reported in weighted MD, 95% CI and p-value. p < 0.05 will be considered statistically significant for the overall MD of the intervention group compared to the control group. The results will be presented in forest plots. We will impose a random-effect model as this is a more conservative method that permits the study heterogeneity to deviate beyond chance, providing further generalizable results (Deeks et al, 2023). All data will be formed using the Review Manager Program (RevMan 5.4.1).

**FUNDING**

Dr. Vitor E. Valenti receives financial support from the National Council for Scientific and Technological Development, an entity linked to the Ministry of Science, Technology, Innovations and Communications from Brazil (Process number 302574/2021-2).

**REFERENCES**

1. Page MJ, McKenzie JE, Bossuyt PM, Boutron I, Hoffmann TC, Mulrow CD, et al. The PRISMA 2020 statement: an updated guideline for reporting systematic reviews. BMJ [Internet]. 2021 Mar 29 [cited 2022 Oct 5];372. Available from: <https://pubmed.ncbi.nlm.nih.gov/33782057/>
2. Sterne JAC, Savović J, Page MJ, Elbers RG, Blencowe NS, Boutron I, et al. RoB 2: a revised tool for assessing risk of bias in randomised trials. BMJ. 2019 Aug 28;366:l4898. doi: 10.1136/bmj.l4898.
3. Meader N, King K, Llewellyn A, Norman G, Brown J, Rodgers M, Moe-Byrne T, Higgins JP, Sowden A, Stewart G. A checklist designed to aid consistency and reproducibility of GRADE assessments: development and pilot validation. Syst Rev. 2014 Jul 24;3:82. doi: 10.1186/2046-4053-3-82. PMID: 25056145; PMCID: PMC4124503.
4. Higgins JPT, Thompson SG. Quantifying heterogeneity in a meta-analysis. Stat Med [Internet]. 2002 Jun 15 [cited 2022 Oct 5];21(11):1539–58. Available from: <https://pubmed.ncbi.nlm.nih.gov/12111919/>
5. Higgins, J.P.; Thompson, S.G.; Deeks, J.J.; Altman, D.G. Measuring inconsistency in meta-analyses. BMJ 2003, 327, 557–560.
6. Deeks JJ, Higgins JPT, Altman DG (editors). Chapter 10: Analysing data and undertaking meta-analyses. In: Higgins JPT, Thomas J, Chandler J, Cumpston M, Li T, Page MJ, Welch VA (editors). Cochrane Handbook for Systematic Reviews of Interventions version 6.4 (updated August 2023). Cochrane, 2023. Available from www.training.cochrane.org/handbook
