# Supplementary material for: Impact of creatine supplementation on inflammation: evidence from a systematic review and meta-analysis of randomized double-blind placebo trials
Source: Front Immunol. 2026 Feb 19;17:1743603. doi: 10.3389/fimmu.2026.1743603 (PMC12961398; doi:10.3389/fimmu.2026.1743603)
Supplement: Supplementary file 2 [file SupplementaryFile1.zip › SR Creatine inflammatory markers (Kell Doutorado). /Supplementary Files/Ficha de Triagem modelo.docx]

**FICHA DE ELEGIBILIDADE (REVISÃO SISTEMÁTICA)**

Etapa leitura da metodologia/resultados dos artigos na íntegra

**Motivo (s) de exclusão (se houver):**

**P)** Os participantes possuem mais de 18 anos de idade?

( ) Sim ( ) Não [*Excluir*]

**I)** A intervenção utilizada no estudo é creatina? Vale qualquer formato de administração

( ) Sim ( ) Não [*Excluir*]

**C)** Teve grupo controle?

( ) Sim ( ) Não [*Excluir*]

**O)** O estudo apresenta valores de marcadores inflamatórios (citocinas e outros marcadores) antes e após as intervenções de creatina?

( ) Sim ( ) Não [*Excluir*]

**S)** O estudo é um ensaio clínico randomizado e controlado?

( ) Sim ( ) Não [*Excluir*]
