# Supplementary material for: Impact of creatine supplementation on inflammation: evidence from a systematic review and meta-analysis of randomized double-blind placebo trials
Source: Front Immunol. 2026 Feb 19;17:1743603. doi: 10.3389/fimmu.2026.1743603 (PMC12961398; doi:10.3389/fimmu.2026.1743603)
Supplement: Supplementary file 2 [file SupplementaryFile1.zip › SR Creatine inflammatory markers (Kell Doutorado). /Supplementary Files/Data for meta-analysis.docx]

**OBJETIVO: VERIFICAR OS EFEITOS DA CREATINA SOBRE PARÂMETROS INFLAMATÓRIOS**

**INCLUÍDOS**

**Completar dose, tempo de intervenção e N amostral dos próximos estudos**

**EFEITOS CRÔNICOS**

**Cornisha & Peeler 2018 (20 g/day for 1 week and then 5g/day por 11 weeks) X 12 weeks intervention. Intervention group: n=9. Control group: n=9**

Comparar Creatine Post vs Placebo post

CRP: Table 2: Intervention: 20.8±18.4 / Control: 21.7±18.4

IL-6: Table 2: Intervention: 1.1±0.6 / Control: 1.1±0.6

**Oliveira et al 2020 (5g/day) X 12 weeks intervention. Intervention group: n=13. Control group: n=14**

**Comparar CR + RT post vs. PL + RT post**

IL-6: Table 2: Intervention: 4.1±2.0 / Control: 4.3±1.9

CRP: Table 2: Intervention: 2.9±2.0 / Control: 3.3±3.2

**Taes et al 2004 (2 g/day) X 4 weeks intervention. Intervention group: n=25. Control group: n=20**

**Comparar Creatine Period 2 vs Placebo Period 2**

CRP: Table 3: Intervention: 6 (1-10) / Control: 3 (1-9)

**EFEITOS AGUDOS**

Rawson et al 2007 **(0.3g/kg de peso corporal/dia durante 5 dias, seguido de 0.03g/kg de peso corporal/dia durante 5 dias) X 10 days intervention. Intervention group: n=11. Control group: n=11**

**Comparar Placebo 24 vs Creatine 24**

CRP: Figure 7: Intervention: -0.19+0.59 / Placebo: -0.15+0.82

Tarnopolsky et al 2007 **(5 g/day) X 24 weeks intervention. Intervention group: n=21. Control group: n=18**

**Comparar Placebo Men post vs Supplement Placebo Men post**

CRP: Table 5: Intervention: 3.79±4.33 / Control: 1.85±1.05

EXCLUÍDOS DA META-ANÁLISE

Bassit et al 2008: Não tem desvio-padrão

Deldicque et al 2008: MArcadores em expressão genética, não no plasma.

Santos et al 2004: Apenas 1 desfecho
