# Supplementary material for: Impact of creatine supplementation on inflammation: evidence from a systematic review and meta-analysis of randomized double-blind placebo trials
Source: Front Immunol. 2026 Feb 19;17:1743603. doi: 10.3389/fimmu.2026.1743603 (PMC12961398; doi:10.3389/fimmu.2026.1743603)
Supplement: Supplementary file 2 [file SupplementaryFile1.zip › SR Creatine inflammatory markers (Kell Doutorado). /Supplementary Files/Motivos da exclusão da referência.docx]

**Pesquisa:** Efeitos do exercício no risco cardiovascular de pacientes com câncer.

***Artigo 1 -* Effectiveness of physical exercise on the cardiovascular system in breast cancer patients: a systematic review and meta-analysis of randomized controlled trials.**

Autores: Shurui Wang, Ting Yang, Wanmin Qiang*, Aomei Shen, Zihan Zhao, Xing Chen, Chenxi Xi, Huan Liu, Fengli Guo

**P)** Os participantes passaram são portadores de câncer e possuem mais de 18 anos de idade?

( X ) Sim ( ) Não [*Excluir*]

- “Studies that included participants of any age with histologically confirmed BC and had completed their primary treatment (surgery, radiotherapy, hormonotherapy, or chemotherapy) were eligible for the study.”

**I)** A intervenção utilizada no estudo é qualquer tipo de exercício?

( X ) Sim ( ) Não [*Excluir*]

- “Physical exercise is defined as any bodily movement produced by skeletal muscles that require energy expenditure, includes but is not limited to one, or a combination of the following: aerobic, flexible or endurance exercise, resistance training, yoga, stretching activities, dancing programs, in the context of daily, family and community activities[26,27]. Therefore, the interventions included in this meta-analysis involving one or more exercise as mentioned above modes and were not limited by the frequency, intensity, duration, or training status of exercise.”

**C)** Teve grupo controle ou placebo ou os voluntários foram controles deles mesmos?

( ) Sim (X) Não [*Excluir*]

Trecho do artigo que mostra isso:

- “No control group was evaluated.”

**O)** O estudo apresenta valores de variáveis cardiovasculares?

( X ) Sim ( ) Não [*Excluir*]

- “Moreover, while lowered resting heart rate, BP, BMI, and breathing conditions are known phenotypic responses to exercise, less is known about how such changes may influence cardiovascular function and outcomes after BC treatment.”

**S)** O estudo é uma revisão sistemática com ou sem meta-análise?

( X ) Sim ( ) Não [*Excluir*]

- “The systematic review was registered with PROSPERO (CRD42021226319), and the Preferred Reporting Items for Systematic Reviews and Meta-Analyses guidelines for systematic review reporting were followed[25].”
