# Supplementary material for: Impact of creatine supplementation on inflammation: evidence from a systematic review and meta-analysis of randomized double-blind placebo trials
Source: Front Immunol. 2026 Feb 19;17:1743603. doi: 10.3389/fimmu.2026.1743603 (PMC12961398; doi:10.3389/fimmu.2026.1743603)
Supplement: Supplementary file 2 [file SupplementaryFile1.zip › SR Creatine inflammatory markers (Kell Doutorado). /Supplementary Files/GRADE Should Creatine vs. Placebo be used for inflammation-5.pdf]

Author(s):
Question: Creatine compared to Placebo for inflammation
Setting:
Bibliography:

Certainty assessment							№ of patients		Effect		Certainty	Importance
№ of studies	Study design	Risk of bias	Inconsistency	Indirectness	Imprecision	Other considerations	Creatine	Placebo	Relative (95% CI)	Absolute (95% CI)		
**CRP - acute effects**												
2	randomised trials	very serious[a]	not serious	not serious	not serious[b]	all plausible residual confounding would suggest spurious effect, while no effect was observed	32	29	-	SMD **0.32 SD higher** (0.29 lower to 0.94 higher)	⊕⊕⊕◯ Moderate[a,b]	CRITICAL
**CRP - chronic effects**												
2	randomised trials	very serious[a]	not serious	not serious	not serious	all plausible residual confounding would suggest spurious effect, while no effect was observed	22	23	-	SMD **0.11 SD lower** (0.69 lower to 0.48 higher)	⊕⊕⊕◯ Moderate[a]	CRITICAL
**IL-6 - chronic effects**												
2	randomised trials	very serious[a]	not serious	not serious	not serious	all plausible residual confounding would suggest spurious effect, while no effect was observed	22	23	-	SMD **0.06 SD lower** (0.64 lower to 0.53 higher)	⊕⊕⊕◯ Moderate[a]	CRITICAL

CI: confidence interval; **SMD:** standardised mean difference

Explanations

a. Missing outcome data
b. 95%CI: -0,29; 0.94
