# Supplementary material for: Impact of creatine supplementation on inflammation: evidence from a systematic review and meta-analysis of randomized double-blind placebo trials
Source: Front Immunol. 2026 Feb 19;17:1743603. doi: 10.3389/fimmu.2026.1743603 (PMC12961398; doi:10.3389/fimmu.2026.1743603)
Supplement: Supplementary file 2 [file SupplementaryFile1.zip › SR Creatine inflammatory markers (Kell Doutorado). /Supplementary Files/Final References/Final/Tarnopolsky et al 2007.pdf]

Creatine Monohydrate and Conjugated Linoleic Acid Improve Strength and Body Composition Following Resistance Exercise in Older Adults

Mark Tarnopolsky[1]*, Andrew Zimmer[1], Jeremy Paikin[1], Adeel Safdar[1], Alissa Aboud[1], Erin Pearce[1], Brian Roy[2], Timothy Doherty[3,4]

1 Department of Pediatrics and Medicine, McMaster University, Hamilton, Ontario, Canada, 2 Department of Physical Education and Kinesiology, Brock University, St. Catharines, Ontario, Canada, 3 Department of Clinical Neurological Sciences, The University of Western Ontario, London, Ontario, Canada, 4 Department of Physical Medicine and Rehabilitation, The University of Western Ontario, London, Ontario, Canada

Aging is associated with lower muscle mass and an increase in body fat. We examined whether creatine monohydrate (CrM) and conjugated linoleic acid (CLA) could enhance strength gains and improve body composition (i.e., increase fat-free mass (FFM); decrease body fat) following resistance exercise training in older adults (>65 y). Men (N = 19) and women (N = 20) completed six months of resistance exercise training with CrM (5g/d)+CLA (6g/d) or placebo with randomized, double blind, allocation. Outcomes included: strength and muscular endurance, functional tasks, body composition (DEXA scan), blood tests (lipids, liver function, CK, glucose, systemic inflammation markers (IL-6, C-reactive protein)), urinary markers of compliance (creatine/creatinine), oxidative stress (8-OH-2dG, 8-isoP) and bone resorption (*N*-telopeptides). Exercise training improved all measurements of functional capacity ($P<0.05$) and strength ($P<0.001$), with greater improvement for the CrM+CLA group in most measurements of muscular endurance, isokinetic knee extension strength, FFM, and lower fat mass ($P<0.05$). Plasma creatinine ($P<0.05$), but not creatinine clearance, increased for CrM+CLA, with no changes in serum CK activity or liver function tests. Together, this data confirms that supervised resistance exercise training is safe and effective for increasing strength in older adults and that a combination of CrM and CLA can enhance some of the beneficial effects of training over a six-month period. ***Trial Registration.*** **ClinicalTrials.gov NCT00473902**

Citation: Tarnopolsky M, Zimmer A, Paikin J, Safdar A, Aboud A, et al (2007) Creatine Monohydrate and Conjugated Linoleic Acid Improve Strength and Body Composition Following Resistance Exercise in Older Adults. PLoS ONE 2(10): e991. doi:10.1371/journal.pone.0000991

INTRODUCTION

Human aging is associated with a reduction in muscle mass and strength that can lead to functional impairment in activities of daily living. Sarcopenia refers to a reduction in fat free mass (FFM, >2 SD below normal) that can occur with aging and this leads to functional impairments in up to 25% of women and men over the age of 75 y [1–3]. Resistance exercise training is an important countermeasure for sarcopenia with numerous studies demonstrating improvements in strength, function, and fat free mass in older adults [4–8]. Several strategies have been evaluated to augment the gains in FFM and strength during the course of resistance exercise training including sex hormone administration [9] and nutritional supplements [6]. While there is no question that resistance exercise is the most potent stimulus for the promotion of strength and FFM gains, adjunctive strategies to augment these effects may enhance the overall efficacy of strength training interventions. An increase in body fat occurs with the aging process. This increase in percentage body fat compounds the negative effects of the accompanying sarcopenia [10].

Creatine monohydrate (CrM) is a guanidino compound that is produced endogenously and is naturally occurring in meat containing products. In addition to its role as a temporal and spatial energy buffer, CrM supplementation can enhance increases in fat-free mass (FFM) when given in association with a several month resistance exercise training program [11–15]. There are several effects of CrM administration that may enhance resistance exercise-induced strength gains including activation of myogenic determination factors [16], enhancement of satellite cell activation and recruitment [17–19], reduction of amino acid oxidation and protein breakdown [20], and an increase in myofibrillar mRNA and protein content [21]. Our group [15], and others [13], have found that CrM supplementation during a resistance exercise training program increased FFM gains and some measures of strength in older adults. By contrast, a few other groups have not confirmed such results [22,23]. However, in one case there was no increase in strength following one year of low intensity resistance exercise training [23], and the other study was cross-sectional in design [22]. Given some discrepancy in the literature, one aim of the current study was to evaluate the efficacy of CrM supplementation as an adjunct to six months of moderate intensity resistance exercise training on measures of body composition, functional tasks, and strength in a cohort of older adult men and women. Given that body fat mass gain is another feature of aging, and this was not affected by resistance training, with or without creatine, in our last study [15], we also wanted to evaluate whether other nutritional compounds could enhance body fat mass losses.

Conjugated linoleic acid (CLA) refers to two naturally occurring isomers of linoleic acid which are found predominately in dairy

Academic Editor: Chenxi Wang, University of Louisville, United States of America

Received April 27, 2007; **Accepted** September 5, 2007; **Published** October 3, 2007

Copyright: © 2007 Tarnopolsky et al. This is an open-access article distributed under the terms of the Creative Commons Attribution License, which permits unrestricted use, distribution, and reproduction in any medium, provided the original author and source are credited.

Funding: This study was funded entirely by the Canadian Institute of Health Research.

Competing Interests: Dr. Tarnopolsky has received an investigator initiated grant to evaluate the absorption characteristics of a new form of creatine (creatine ascorbate) as compared with creatine monohydrate from Avicena (2006). The current submitted study was completed in 2005 and Avicena did not contribute any money to the current study, but did supply product. Dr. Tarnopolsky has not received any personal money or sponsored talks for either Avicena or Pharmanutrients.

* **To whom correspondence should be addressed.** E-mail: tarnopol@mcmaster.ca

products and plant oils such as flax seed [24,25]. Studies in animals have found a reduction in intra-abdominal fat and enhanced FFM gains with CLA supplementation [24,26–28]. Other work has reported that CLA-mediated whole body fat loss in overweight men and women [29–31]; however, the efficacy of CLA in combination with resistance exercise training in normal weight young men is equivocal [32]. There have been some concerns that CLA can promote oxidative stress (8-isoprostanes, 8-isoP) [33,34], and induce hepatic lipid accumulation [35]. The latter effect appears to be isomer specific and is not seen with commercially available mixtures containing equal proportions of each of the two major isomers [26,36]. Therefore, a second aim of the study was to determine whether CLA leads to a reduction in body fat during resistance exercise training and to evaluate safety issues related to oxidative stress and hepatic dysfunction in older adults.

In addition to the biomechanical disadvantage resulting from obesity, body fat accumulation is associated with negative health outcome indicators including an increase in markers of inflammation, oxidative stress, glucose dysregulation, and dyslipidemia [37]. The influence of resistance exercise training on markers of inflammation, blood lipids, and adipokines has rarely been studied in older adults [38]. Consequently, the final aim of the study was to evaluate whether resistance exercise training with or without a CrM+CLA supplementation would favorably alter markers of inflammation, oxidative stress, blood lipids or adipokines in older adults.

MATERIALS AND METHODS

Participants

Thirty-nine community dwelling, older adult (65–85 y), men (n = 19) and women (n = 20) were recruited from within a 30 km radius of our research centre (McMaster University) to participate in a six month resistance exercise training study. None of the subjects participated in sports training programs or performed vigorous physical activity more than 3 times per wk, >30 min/d, in the year prior to the commencement of the study. None of the subjects had participated in resistance exercise activities in the preceding two years and most had not done such training. No participants required the use of assistive devices for mobility. Recruitment strategies included local newspaper ads, radio announcements, and flyers distributed to surrounding areas. Each subject underwent a thorough screening, which included a telephone interview and a medical evaluation. Exclusion criteria included: evidence of coronary heart disease; congestive heart disease; uncontrolled hypertension; chronic obstructive pulmonary disease; diabetes mellitus; renal failure; major orthopedic disability; and smoking. All the women were post-menopausal and were not taking hormone replacement therapy. The study was approved by the Hamilton Health Sciences Research Ethics Board and the study has been registered in ClinicalTrials.gov under # NCT00473902. The protocol for this trial is available as supporting information; see Protocol S1. Information sessions were conducted to inform the subjects of the risks and benefits of participating in a resistance exercise training program and they provided written consent.

After the initial screening, the subjects were required to perform a cycle ergometry test to 5 METS (metabolic equivalents) on a mechanically braked cycle ergometer (Monarch, Varberg, Sweden). A 12-lead electrocardiogram (ECG, Burdick, Kone Instruments Corp., Espoo, Finland) was taken prior to and upon completion of the ergometry test, as part of exclusion criteria to participate in the resistance exercise training program. Any participants with abnormal resting ECG's or a blood pressure of >160 mmHg systolic and/or 95 mmHg diastolic were not allowed to complete the screening tests. One subject with questionable post-exercise exercise ECG changes was referred to a cardiologist for assessment and was cleared to participate in the study. A summary of the recruitment strategy and allocation is presented in Figure 1.

Nutritional Supplementation

After completion of pre-training testing, subjects were then randomized to either a combination of CrM and CLA (CrM+CLA) (5 g CrM (Neotine®)+2 g dextrose/d, Palo Alta, CA); and 6 g of CLA (CLA-ONE® 45% c9, t11; 45% t10, c12, Pharmanutrients, Gurnee, IL) or placebo (PL) (7 g dextrose/d+6 g of safflower oil/d) for 24 weeks. The CrM+CLA group consisted of 11 men and 10 women, while the PL group consisted of 8 men and 10 women (Table 1). Randomization was done by having recruited men and women randomly draw from two blocks (one male and one female) each containing twenty-four shuffled/sealed envelopes with each block having equal allocation to treatment (N = 12/block) or placebo (N = 12/block). After six months of recruitment and a lack of drop-outs, we closed recruitment with a total of 19 men and 20 women randomized to the trial. The flavor and appearance of the supplements were indistinguishable by the subjects and investigators. Subjects were instructed to consume their supplements daily and return unused supplements every month to ensure compliance.

Strength Training

Subjects followed a resistance exercise training program twice per week for six months, while being supervised in a group environment for each training session. All participants recorded each contraction in a log book. Sessions were held on Mondays and Wednesdays (1200 h–1400 h) or Tuesdays and Thursdays (1900 h–2100 h) for 24 weeks. Each training session was preceded by a 5-min aerobic warm-up of spinning on a cycle ergometer or brisk walking, followed by a brief stretching session. Twelve exercises were used to train the major muscle groups of the upper and lower body in a circuit-set system using weight training machines (Universal Gym Equipment Inc., Cedar Rapids, Iowa). Subjects performed 12 repetitions of each exercise including; leg press, chest press, leg extension, leg flexion, shoulder press, lat pull-down seated row, calf raise, abdominal crunch, and back extension and 10 repetitions for arm flexion and arm extension. The training protocol progressed from performing 1 set of each exercise at 50% of their 1RM (repetition maximum) values up to 3 sets of 75% of their 1RM values over the course of the training period. The 1RM's were re-tested every 4 weeks to accommodate any increases in strength by the subjects and re-adjusted to the new 1RM value.

Testing

Once recruited, each subject was put through three separate visits for pre- and post-testing. Testing included analyses of functional and strength measures, body composition, diet records and urine and blood collection (see below). The same individual completed all of the strength testing and DEXA scanning to ensure operative consistency.

Functional Testing

A total of five functional tasks were performed once using a stopwatch that recorded times to an accuracy 1/100 of a second. These tasks included; 1. 30 metre walking-subjects walked as fast and safely as possible for a 30m distance that was marked off with clear start and stop points; 2. Balance-subjects were timed during a 'heel to toe' tandem gait on a pre-marked 9.14-meter tape line

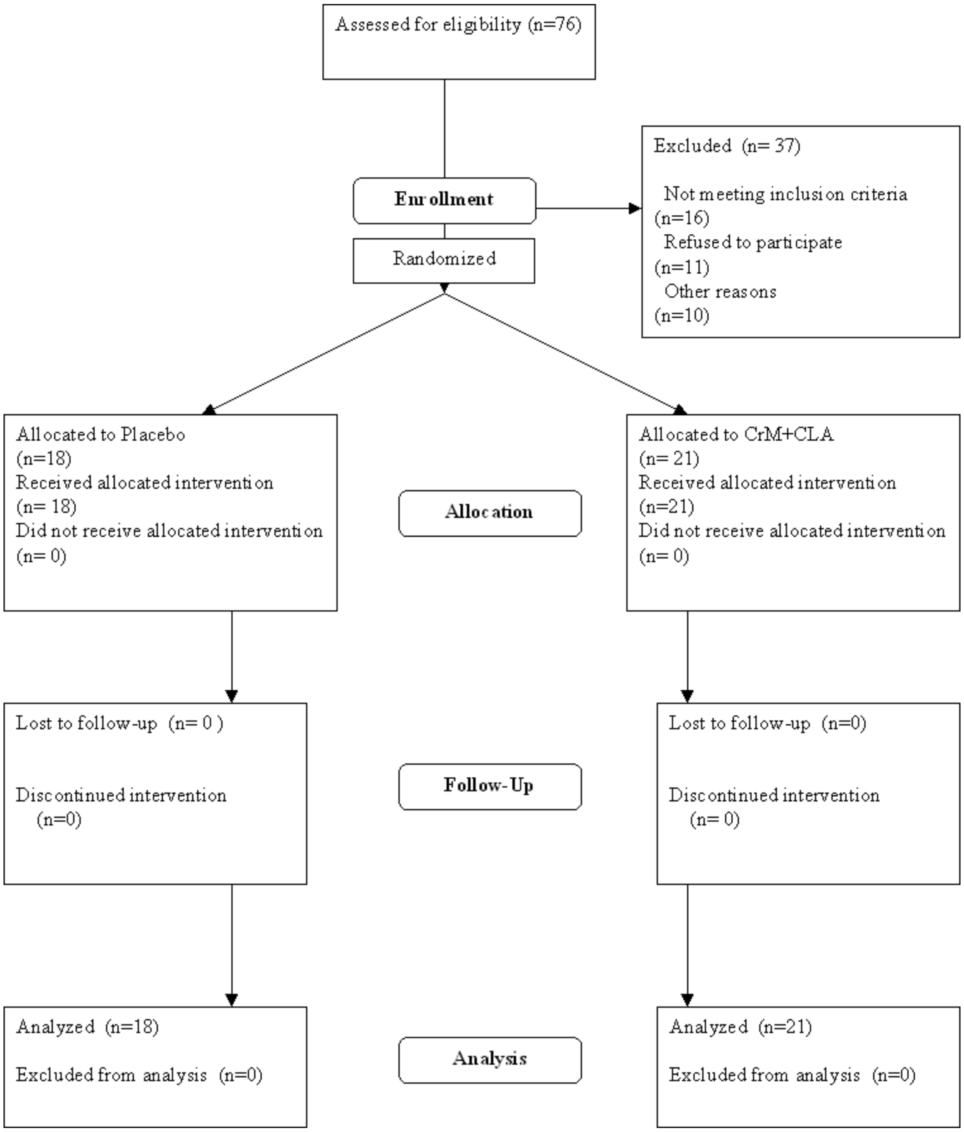

Figure 1. Summary of the recruitment strategy and allocation.
doi:10.1371/journal.pone.0000991.g001

on the floor. Subjects could keep their arms out to aid in balance and for safety. Any step off the line resulted in a one second addition to the total task time; 3. Sit to stand-subjects performed a series of consecutive rising and sitting from a sturdy, armless plastic chair secured against a wall. Starting from a seated position, the numbers of completed repetitions were recorded over 30 s, with their arms at their sides or across their chests while performing the task; 4. Chair rise and walk–starting from a seated position, subjects stood up and walked as quickly as possible in a predetermined straight line to a pylon 9.14 m, go around the pylon, and return to their original seated position; 5. Stair climb–subjects climbed two flights of stairs (N = 14), starting with both feet on the bottom platform and ascending one step at a time, using a handrail only if insecure.

Strength testing

Isometric ankle dorsiflexion Maximal ankle dorsiflexion was taken as the highest of three trials of 5 s maximal contractions using a custom-made isometric device with the ankle joint at 90°, as previously described [39].

Hand grip strength Hand grip strength was measured using an isometric dynamometer (JAMAR®, Sammons, Bolingbrook, IL). The grip width was adjusted to hand size and subjects performed three×5 s with a one min rest between each trial.

Isometric and isokinetic knee extension Isometric and isokinetic (120°/sec) knee extension was measured by using a dynamometer (Biodex System 3, Biodex Medical Systems, Shirley, NY). Subjects were positioned into the machine with the knee flexed at 90° and performed three×5 s maximal voluntary contractions with 30 s rest between each trial at each speed.

1RM and endurance testing One repetition maximum (1 RM) testing occurred prior to training commencement and post-training for use as an outcome measurement and to set individual weight assignments for the training sessions (as above). Subjects were tested for training intensity adjustments for all 12 exercises described above, and for outcome variables using four machines: leg press, chest press, arm flexion, and leg extension (Universal Gym Equipment Inc., Cedar Rapids, Iowa). Muscle endurance was also completed at the end of the study using the weight from

Table 1. Subject Characteristics, Body Composition and Bone Density.

	Placebo				Supplement					
	MEN (N = 8)		WOMEN (N = 10)		MEN (N = 11)		WOMEN (N = 10)		Training Effect	Sex Effect
	Pre	Post	Pre	Post	Pre	Post	Pre	Post	P value	P value
Age (y)	74.8±6.6	NC	68.3±4.4	NC	71.8±5.2	NC	69.5±3.8	NC	NC	0.01
Height (cm)	172.1±7.4	NC	160.8±6.8	NC	171.0±7.4	NC	161.1±5.2	NC	NC	0.00
Weight (kg)	76.7±8.2	77.2±7.8	65.3±10.5	65.1±10.2	81.6±11	81.8±11.6	66.2±8.4	65.7±8	0.98	0.0002
FFM (kg)	55.8±5.5	57.2±5.7†	41.1±5.1	41.5±5.1	58.1±8.6	60.8±8.5*,†	41.3±4.4	42.9±4.7*	0.0001	0.0001
FM (kg)	18.8±4.4	18.2±4.2	22.8±5.7	22.6±5.9	21.4±4.9	19.9±5.9*	23.5±5.3	21.5±5.6*	0.0001	0.08
B.M.I.	25.9±2.7	26.1±2.5	25.2±3.0	25.1±2.9	27.8±2.1	27.8±2.1	25.5±3.1	25.3±3.0	0.07	0.86
BMD-total	1.21±0.09	1.25±0.11	1.13±0.12	1.15±0.13	1.20±0.13	1.23±0.13	1.02±0.09	1.04±0.08	0.34	0.0005
BMD-hip	1.01±0.09	0.98±0.08†	0.86±0.14	0.84±0.13	0.94±0.10	0.92±0.10†	0.83±0.10	0.81±0.11	0.0001	0.001
BMD-L-spine	1.12±0.06	1.12±0.07	1.12±0.15	1.11±0.18	1.19±0.14	1.19±0.15	0.94±0.11	0.94±0.09	0.66	0.004

Values are means±standard deviation. NC = no change; FFM = fat free mass; FM = fat mass; BMI = body mass index.
*indicates a significant interaction between training and supplement (see text).
†indicates a significant interaction between men and women in response to training (see text).
doi:10.1371/journal.pone.0000991.t001

the initial 1RM by determining the number of repetitions done post-training using the original 1RM weight.

Body composition assessment

Body mass and height were measured to the nearest 0.1 kg and 0.5 cm, respectively, using a calibrated electronic scale (Health-O-meter Pro Series Electronic Scale, Bridgeview, IL) (Table 1). Body composition was assessed using dual energy x-ray absorptiometry (DEXA) scan (Hologic QDR 1000W, Waltham, MA) and a software program for adults (Hologic, V.8.26a). Fat free mass (FFM), fat mass (FM) and bone mineral density (BMD) of the entire body, spine, and femur were recorded.

Diet records

To determine dietary composition and consistency during the study, two prospective 3d diet records were collected from the subjects 1 wk prior to the start and during the final week of exercise training. Dietary intake was analyzed using computerized diet analysis software (Diet Analysis Plus Version 7.0, Thompson Wadsworth, Canada) for the determination of total energy, % protein (PRO), carbohydrate (CHO), and fat, alcohol, and calcium intake.

Urine and blood collections

Twenty-four hour urine samples were collected into 4 L containers before training began, and once again in last week of training. The samples were kept at 4°C, and delivered to the laboratory within 24 h where the volume was measured and samples were aliquoted into 5 mL polyethylene tubes and stored at −80°C until subsequent analysis (see below). All subjects arrived between 0800 h and 0930 h in an overnight post-absorptive state. Blood was taken from the antecubital vein and drawn into 10 mL evacuated tubes with heparin used for plasma collection and non-treated tubes were used to collect serum Samples were centrifuged at 1200 rpm for 10 min and the serum was aliquoted into 1.5 mL polyethylene microcentrifuge tubes and stored at −80°C for subsequent analysis.

Blood and Urine Analyses

Serum glucose, creatine kinase (CK), bilirubin, gamma glutamyl transferase (GGT), low density lipoprotein (LDL), high density lipoprotein (HDL), and total cholesterol were each analyzed by the core laboratory at Hamilton Health Sciences Centre in batches containing samples from men and women, pre-post training, and from each of the treatment groups. All other metabolites were analyzed according to the manufacturer's guidelines using ELISA-based assays for leptin (R&D Systems, DLP00, Minneapolis, MN), C-reactive protein (CRP) (Alpha Diagnostic, U54401841, San Antonio, TX), adiponectin (R&D Systems, DY1065, Minneapolis, MN), and interleukin-6 (I-L6) (R&D Systems, HS600B, Minneapolis, MN). Insulin concentration was determined using a commercially available radio-immunoassay (Coat-A-Count, TK1N2, Diagnostics Products, Los Angeles, CA). The HOMA-IR index was calculated using the equation ([fasting serum insulin (uU/mL)×fasting plasma glucose (mmol/L)]/22.5).

Urine creatine (Cr) and creatinine (Crn) were analyzed using high-performance liquid chromatography as described previously by our group[40]. The creatinine clearance rate was calculated from the measured serum and urinary creatinine values using the equation (urine creatinine (mg/dL)×urine vol. (mL/24 h)/[1440×serum creatinine (mg/dL)]). Urinary 8-isoprostanes (8-isoP, Cayman Chemical, # 516351, Ann Arbour, MI Cayman Chemical, # 516351, Ann Arbour, MI), 8-hydroxy-2-deoxy-guanosine (8-OH-2dG, KOG-200SE, Baltimore, MD), and *N*-telopeptides (Osteomark, NTx # 504837, Princeton, NJ) were analyzed by ELISA according to manufacturer's instructions.

Statistical Analysis

The subject characteristics were analyzed using an unpaired t-test (age and height). In accordance with NIH guidelines, and given that sex differences in sarcopenia have been reported [1–3], we planned our statistical analysis to determine whether or not sex differences existed. For the main outcome variables of body composition (FFM and body fat) and strength (knee extension and handgrip) we first ran a Kolmogorov-Smirnov test and reviewed the distribution plots to determine whether the data was normally distributed. In all of these cases the criteria for a normal distribution were met and our outcome variables were analyzed using a three-way analysis of variance (ANOVA) with sex as a between variable (men vs. women), and training (pre vs. post) and supplement (CrM+CLA vs. placebo) as the repeated measures variable. A Tukey's HSD *post-hoc* analysis for unequal sample sizes

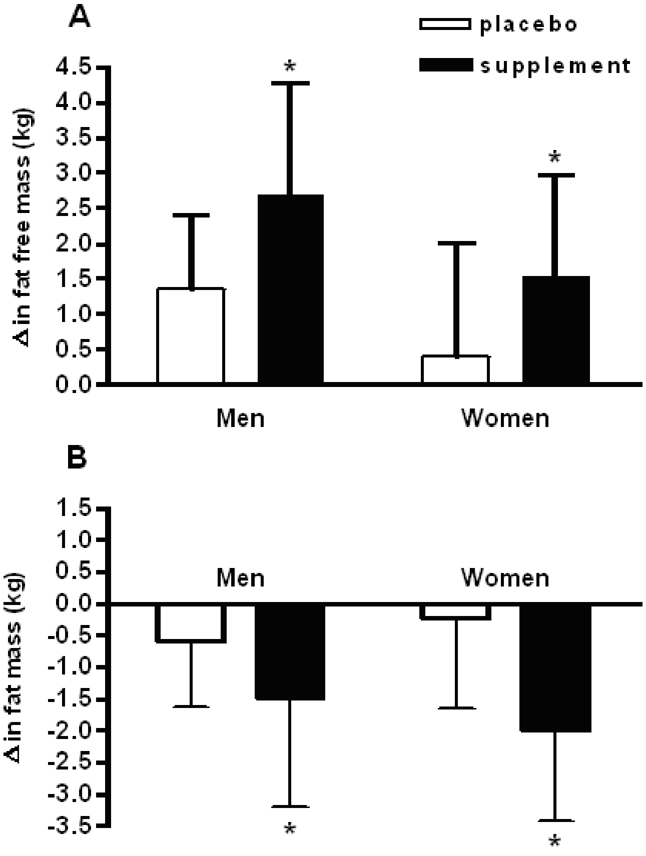

Figure 2. (A) Changes in fat free mass between supplement groups and sex in response to training. (B) Changes in fat mass between supplement and sex in response to training. Values are means±standard deviation. *-indicates a significant difference with a $p<0.05$ (see text).
doi:10.1371/journal.pone.0000991.g002

was used to locate pair-wise differences when statistical significance was observed. In addition, body composition measures were further analyzed to blood and urine markers using Pearson R correlation. Significance was set at $P \leq 0.05$ (two-tailed). All data was analyzed using a computerized program (Statistica V5, Statsoft, Tulsa, OK). Values are reported as mean±standard deviation (SD).

RESULTS

Subject Characteristics and Body Composition

The men were slightly older, taller, heavier, and had higher FFM compared to the women (Table 1). Resistance exercise training increased FFM more for men (+2.0 kg, $P<0.001$) compared to women (+1.0 kg, $P<0.05$), with a significantly greater increase in FFM for the CrM+CLA group (+2.1 kg, $P=0.02$) compared to placebo (+0.9 kg, $P=0.06$, NS) (Table 1, Figure 2). There was a significantly greater reduction in fat mass in the CrM+CLA group following training (−1.9 kg, $P<0.001$) as compared to placebo (−0.4 kg, NS)(Table 1, Figure 2). Total bone mineral density (BMD) was lower for women and did not change after training. Hip BMD was lower for women and decreased slightly, but significantly for men only after training (interaction, $P<0.05$). Lumbar BMD was lower for women but did not change in response to exercise training with or without CrM+CLA (Table 1).

Diet Analysis

Men consumed more energy compared to women ($P<0.02$), but there was no effect of training on energy intake, and men on placebo had lower % fat intake vs. all other groups. There were no other sex, training, or supplement effects on dietary composition (Table 2).

Muscle Strength Testing

All measures of strength were higher for men compared to women (Table 3). Every measure of muscle strength ($P<0.05$ to $P<0.001$) increased following exercise training (Table 4). The CrM+CLA supplement did not further enhance isometric strength measurements after training; however, isokinetic strength increased more after training for those on supplement vs. placebo ($P<0.05$). Women on CrM+CLA showed a greater increase in knee extension 1RM strength after training as compared to all other groups ($P<0.05$). Muscle endurance increased significantly ($P<0.0001$) following training (# reps. post-training @ pre-training 1RM) with the improvements in chest press and arm flexion showing a greater improvement ($P<0.05$), with a trend towards a greater improvement in leg press ($P=0.077$), for the CrM+CLA group. There was a three-way interaction ($P<0.05$) with women showing a greater improvement in knee extension endurance after training for the CrM+CLA group (Table 3). Correlation analysis showed that those with the lowest initial knee extension strength had the greatest percentage increase in strength after training ($r=-0.61$, $r^2=0.37$, $P<0.05$).

Table 2. Dietary Analysis.

	Placebo				Supplement					
	MEN (N = 5)		WOMEN (N = 7)		MEN (N = 9)		WOMEN (N = 8)		Training Effect	Sex Effect
	Pre	Post	Pre	Post	Pre	Post	Pre	Post	P value	P value
Intake (kcal/d)	2812±914	2777±689	1989±178	1886±356	2435±1108	2409±1110	1916±466	1974±681	0.81	0.02
% PRO	16±3	16±2	16±3	17±3	15±3	16±3	17±4	17±2	0.67	0.26
% CHO	60±4	61±10	49±10	52±11	50±6	48±5	49±10	51±6	0.48	0.14
% FAT	26±4	23±4	35±8	33±5	34±6	34±4	35±6	34±4	0.18	0.007
PRO (g/kg)/d	1.3±0.6	1.4±0.3	1.4±0.4	1.2±0.3	1.1±0.4	1.1±0.3	1.2±0.2	1.3±0.3	0.81	0.93
% C_2H_5OH	3.7±3.3	6.1±2.4	3.2±2.9	3.3±4.1	4.1±4.6	5.1±4	3.8±3.7	3.1±3.4	0.21	0.27
Ca^{++}	1204±387	1162±368	718±209	758±158	913±413	773±335	987±462	951±409	0.52	0.17

Values are means±standard deviation. PRO = protein; CHO = carbohydrate; C_2H_5OH = ethanol; Ca^{++} = calcium.
doi:10.1371/journal.pone.0000991.t002

Table 3. Muscle Strength Tests.

	Placebo				Supplement					
	MEN (N = 8)		WOMEN (N = 10)		MEN (N = 10)		WOMEN (N = 10)		Training Effect	Sex Effect
	Pre	Post	Pre	Post	Pre	Post	Pre	Post	P value	P value
Isometric										
Knee Ext.(Nm)	133.9±37.3	152.0±44.7	78.6±12.6	96.0±20.2	135.8±37.4	151.4±52.4	86.0±14.3	105.6±17.8	0.0001	0.0001
Dorsiflexion(Nm)	56.7±14.9	55.9±14.4	35.5±4.6	37.3±5.2	48.8±10.9	54.7±12.7	30.4±5.2	34.1±7.6	0.03	0.0001
Jamar (kg)	39±6	42±6	25±4	27±4	39±7	43±7	27±5	30±4	0.0001	0.0001
Isokinetic										
Knee 120 deg/sec	93.1±26.7	101.3±23.4	53.2±20.2	64.7±9.2	88.2±27.2	108.2±26.2*	57.7±16.4	68.0±15. 5*	0.0001	0.0001
1 Repetition Maximum (lb)										
Chest Press	98±23	136±16	60±12	91±9	104±35	146±38	53±7	89±15	0.0001	0.0001
Arm Flexion	64±17	104±23	32±9	57±11	76±35	115±36	29±7	61±16	0.0001	0.0001
Leg Press	205±55	269±52	137±33	187±54	215±59	295±74	123±22	200±31	0.0002	0.0001
Knee Extension	106±20	158±31	81±27	109±13	117±39	163±44	64±16	113±18‡	0.0001	0.0001
Endurance (lb)										
Chest Press	1±0	17±11	1±0	18±6	1±0	22±10*	1±0	32±11*	0.0001	0.12
Arm Flexion	1±0	16±7	1±0	15±9	1±0	21±12*	1±0	25±9*	0.0001	0.56
Leg Press	1±0	21±16	1±0	27±12	1±0	27±13	1±0	46±27	0.0001	0.07
Knee Extension	1±0	17±8	1±0	10±2	1±0	14±4	1±0	17±6‡	0.0001	0.38

Values are means±standard deviation. RM = repetition maximum.
*indicates a significant interaction between training and supplement (see text).
‡indicates a significant interaction between training, supplement and gender (see text).
doi:10.1371/journal.pone.0000991.t003

Functional Tests

Women had slightly worse balance walk scores and sit to stand performance compared to men (Table 4). Exercise training improved all five measures of functional capacity for men and women with no differential effect of CrM+CLA (Table 4).

Blood Analyses

There were a number of sex differences in the blood measurements with higher leptin, adiponectin, and HDL-C concentrations in women ($P<0.001$). Furthermore, women had lower creatinine and bilirubin concentrations as compared with men. Following resistance exercise training, there was an increase in CK activity, and an increase in total and LDL cholesterol ($P<0.05$), without a change in LDL/HDL ratio (Table 5). As expected[15], an increase in serum creatinine was seen following training only in the CrM+CLA group ($P<0.05$) (Table 5).

Urine Analyses

Women had higher 8-OH-2dG/creatinine and Cr/Crn ratios and lower 24 h creatinine compared to men (Table 6). Urinary creatine, creatinine and Cr/Crn ratios were all higher following training only in the Cr+CLA supplemented group (Table 6). Neither resistance training, nor the CrM+CLA supplement, influenced the measured plasma creatinine clearance. The 8-OH2-dG/creatinine ratio was lower for both groups following training (Table 6). In contrast, the 8-isoP/creatinine ratio was higher following training only for the females in the CrM+CLA group (Table 6). There were no effects of sex, exercise training or CrM+CLA supplementation on urinary N-telopeptide excretion (Table 6).

Side Effects

Subjects tolerated the supplementation protocol well, with only one report of gastrointestinal distress (stomach cramps) on

Table 4. Functional Tests.

	Placebo				Supplement					
	MEN (N = 8)		WOMEN (N = 10)		MEN (N = 11)		WOMEN (N = 10)		Training Effect	Sex Effect
	Pre	Post	Pre	Post	Pre	Post	Pre	Post	P value	P value
Sit and Stand (#)	13±2	16±1	10±2	14±4	12±2	15±3	11±3	13±2	0.0001	0.0005
Timed Stairs (sec)	6.8±0.6	6.9±1.5	8.7±1.7	8.1±1.6	8.1±1.3	7.7±1.5	7.8±0.9	6.9±0.9	0.004	0.24
Walk Test (sec)	14.8±2.7	13.7±2.1	17.5±2.5	16.1±2.4	17.3±2.6	16.6±3.5	16.8±2.0	15.1±1.2	0.0001	0.34
Chair and Walk (sec)	13.4±1.7	11.4±2.3	15.0±2.4	13.3±2.2	14.5±2.4	13.7±3.1	14.3±2.1	12.6±1.3	0.0001	0.08
Balance Walk (sec)	23.4±8.5	22.3±9.0	33±6.1	28.1±7.6	32.6±11.4	27.4±8.1	31.9±8	25.3±7.8	0.04	0.04

Values are means±standard deviation. Sec = seconds; # = number completed.
doi:10.1371/journal.pone.0000991.t004

Table 5. Blood Analysis.

	Placebo				Supplement					
	MEN (N = 8)		WOMEN (N = 10)		MEN (N = 11)		WOMEN (N = 10)		Training Effect	Sex Effect
	Pre	Post	Pre	Post	Pre	Post	Pre	Post	P value	P value
Glucose (mmol/L)	4.8±0.5	4.8± 0.5	4.6±0.5	4.8±0.8	5.0±0.6	4.9± 1.70	4.9±0.5	4.7±0.6	0.89	0.63
HOMA-IR	1.53±1.01	1.36±0.66	0.76±0.59	1.28±1.46	1.54±1.36	1.23±0.99	1.43±1.01	0.94±0.35	0.50	0.34
CK (μmol/L)	90.1±26.9	113.8±29.8	103.3±31.7	120.3±38.6	114.8±50.1	180.3±120.5	146.0±153.3	187.7±181.7	0.0001	0.78
Bili (μmol/L)	9.3±2.9	10.6±2.3	7.5±2.2	7.5±1.5	10.5±3.9	11.6±6.0	7.5±3.0	7.8±2.4	0.17	0.02
GGT (μmol/L)	26.4±7.9	25.5±4.9	27.3±17.0	26.6±12.4	30.5±22.5	27.8±22.3	21.2±12.8	22.6±5.9	0.90	0.90
Crn (μmol/L)	87±10	86±6	71±10	70±14	90±19	114±25*	71±11	80±17*	0.0001	0.0001
LDL (mmol/L)	3.03±0.56	3.33±0.75	3.27±1.08	3.46±1.19	3.14±0.45	3.33±0.54	3.20±0.96	3.54±0.85	0.002	0.83
HDL (mmol/L)	1.57±0.32	1.63±0.39	2.07±0.45	2.22±0.59	1.32±0.28	1.34±0.21	1.82±0.31	1.83±0.37	0.09	0.0001
Chol (mmol/L)	5.35±0.57	5.68±0.85	5.96±1.16	6.32±1.38	5.37±0.51	5.54±0.72	5.68±1.09	6.13±1.00	0.0007	0.23
LDL/HDL (mmol/L)	2.02±0.59	2.19±0.81	1.65±0.68	1.62±0.62	2.47±0.62	2.54±0.59	1.84±0.79	2.02±0.72	0.097	0.018
Leptin (ng/mL)	4.54±1.87	4.84±2.26	16.0±10.26	15.33±9.61	4.84±2.47	4.38±3.70	16.21±12.74	16.95±15.97	0.97	0.0003
CRP (mg/L)	2.98±3.24	1.85±1.05	3.65±3.62	3.80±4.25	2.56±3.12	3.79±4.33	3.31±3.5	5.12±3.47	0.44	0.23
Adipon. (mg/L)	9.65±4.33	10.32±3.96	17.12±7.54	16.47±6.33	7.18±4.39	6.48±3.19	12.89±5.84	13.72±7.1	0.96	0.0003
IL-6 (pg/mL)	2.38±3.2	2.33±2.21	3.91±3.03	4.06±3.05	5.48±3.08	7.3±4.47	1.81±1.46	2.04±.82	0.10	0.61

Values are means±standard deviation. HOMA-IR = homeostasis model assessment of insulin resistance; CK = creatine kinase; billi = bilirubin; GGT = gamma glutamyl transferase; LDL = low density lipoprotein; HDL = high density lipoprotein; Chol = total cholesterol; CRP = C-reactive protein; Adipon. = adiponectin; IL-6 = interleukin -6.
*indicates a significant interaction between training and supplement (see text).
doi:10.1371/journal.pone.0000991.t005

CrM+CLA but this did not lead to discontinuance of the study. Furthermore, there were no reports of muscular cramping or any other subjective symptoms during the study. Subjects variably reported delayed onset muscle soreness yet none developed any musculoskeletal injuries that required them to discontinue the study. Two individuals complained of anterior knee pain during the knee extension exercises that required a slower increase in the intensity of the training sessions. No individual dropped out due to musculoskeletal injuries.

DISCUSSION

We found that six months of twice weekly, supervised, resistance exercise training robustly improved muscle strength and functional capacity in community dwelling older adults. Significant improvements in body composition (increased FFM and lower fat mass) were seen following resistance exercise training only for the CrM+CLA intervention group. There were several measurements of strength that were higher after training on the CrM+CLA arm of the study, particularly muscular endurance. Other than a reduction in a marker of DNA oxidative stress (8-OH-2-dG) there were no other blood or urine markers that showed changes that would be consistent with a reduction in risk for the metabolic syndrome following resistance exercise training. No adverse affects were noted clinically or serologically, and markers of inflammation (CRP and IL-6), adipocytokines (leptin and adiponectin), and glucose homeostasis (fasting glucose and HOMA-IR index) showed no effect from training or supplementation. Total cholesterol and LDL increased following training for all groups, but the LDL/HDL ratio did not change.

Previous findings of an augmented increase in FFM following resistance exercise training with CrM administration in older adults [13,15], were confirmed in the current study. We have also previously reported that older adults show a significant increase in total muscle creatine following four months of CrM supplementation at the same dose as used in the current study [15]. An increase in satellite cell number occurs in older adults in response to resistance exercise training [41,42], and CrM has recently been shown to enhance satellite cell activation during resistance exercise training in younger men [17]. Together, the aforementioned data suggest that CrM may enhance the resistance exercise induced activation of satellite cells and contribute to the increase in FFM seen in the current study. It is not possible to determine what proportion of the increase in FFM was due to the CrM and what may have been due to the CLA component of the supplement. It is likely that much of the increase in FFM was due to CrM given that the potentiation of the FFM gains seen in the current study were quantitatively similar to those previously reported by our group [15], and others [13], using CrM only. Furthermore, CLA supplementation alone does not appear to enhance FFM gains in young men completing a resistance exercise program [32]. The molecular mechanism(s) behind the observed enhancement of the FFM with CrM were not evaluated in the current study and may have included an increase in satellite cell recruitment [17,19], lower protein breakdown and oxidation [20], or increased myofibrillar protein accretion [21]. Given that the progression of the exercise program was set as a parameter for each participant, that we recorded every repetition and weight lifted during the entire six months of training, and that the final 1 RM values were the same for all groups (aside from slightly greater increases in 2 of 10 exercises for the women on supplement), the total "volume" of training over the six months was virtually identical between supplemented and non-supplemented groups (especially for men). Consequently, some of the improvements in mass and muscular endurance must have been due to molecular or physiological alterations induced by the supplement *per se* and not due to a higher total volume of exercise over the six months.

In addition to a greater increase in FFM after training with the CrM+CLA supplement, we found that several measurements of strength increased to a greater degree in the supplemented groups. In a previous study we found that isometric knee extension strength increased for men and women, and dorsiflexion strength increased only for men following four months of resistance exercise

Table 6. Urine Analysis.

	Placebo				Supplement					
	MEN		WOMEN		MEN		WOMEN		Training Effect	Sex Effect
	Pre	Post	Pre	Post	Pre	Post	Pre	Post	P value	P value
Cr (mg/24 hr)	43.7±51.8	31.9±39.7	150±128.1	181.3±145.5	66±62.6	1851.1± 933.5*	171.4±153.9	2393.7± 1656*	0.0002	0.11
Crn (mg/24 hr)	1265.6±284.0	1293.2±217.0	978.3±258.2	979.0±123.8	1432.1±363.2	1808.0±544.0*	935.9±158.8	1034.1± 31.6*	0.002	0.0001
Crn Cl. (mL/sec)	1.51±0.36	1.54±0.32	1.44±0.44	1.49±0.36	1.72±0.65	1.70±0.71	1.39±0.33	1.40±0.45	0.73	0.21
Cr/Crn ratio	0.04±0.05	0.02±0.03	0.16±0.14	0.19±0.17	0.05±0.06	1.11±1.24*	0.18±0.16	2.24±1.52*	0.0003	0.009
8-OH/Crn (ng/g Crn)	8329±3032	7394±1921	11622±4379	10713±3190	7245±2703	6130±1260	7942±3071	7088±2582	0.01	0.03
8-iso/Crn (pg/mg Crn)	2.9±1.3	3.8±1.0	3.8±1.3	4.1±2.1	2.5±2.2	2.3±1.4	5.2±2.3	9.9±6.2‡	0.03	0.005
N-telo (BCE/mmol Crn)	380.6±335.7	378.1±275.6	354.7±231.3	235.9±146.5	510±353.3	712.4±714.4	257.7±79.7	272.1±154.2	0.67	0.09

Values are means±standard deviation. 8-OH = 8-hydroxy-2-deoxyguanosine; 8-Iso. = 8-isoprostane per 24 hour urine; Cr = Creatine; Crn = creatinine; Cl. = Clearance; *N*-telo = *N*-telopeptides; BCE = bone collagen equivalent.
*indicates a significant interaction between training and supplement (see text).
‡indicates a significant interaction between training, supplement and gender (see text).
doi:10.1371/journal.pone.0000991.t006

training (3 x/wk) [15]. In spite of men showing higher strength values for every outcome measurement in the current study, most measurements of strength improved similarly for men and women. Knee extension strength was the only strength outcome that showed a sex difference with greater increases for women on CrM+CLA after training compared to all other groups. Since we found that those with the lowest initial strength showed the greatest percentage improvements in strength ($r = -0.61$), it may be that this latter finding was reflective of the lower initial strength in the women randomized to CrM+CLA and not a true effect of the supplement *per se*. Nevertheless, this latter finding is of significant clinical relevance because older adults with very low levels of strength are at the greatest risk of falling [43,44], and therefore, it is this group that would stand to gain the most from a resistance exercise training program.

A novel finding in the current study was the robust reduction in the fat mass seen after resistance training in the CrM+CLA group. We feel that this effect was primarily due to the CLA component for a number of reasons. Firstly, almost every study looking at the effects of CrM alone during resistance exercise training did not find any effect on fat mass [12,13,15]. Secondly, studies in overweight humans have found a lower fat mass after CLA supplementation at about the same dose as used in the current study [29–31,45]. Finally, there is strong biological rationale for a reduction in body fat secondary to CLA supplementation. CLA has consistently been shown to lower body fat in animals, likely by increasing PPARα, and SREBP1 in adipocytes and inducing transcription of components of β-oxidation [26,46,47]. CLA has also recently demonstrated an ability to normalize impaired glucose tolerance and improve hyperinsulinemia in a pre-diabetic animal model. It is not clear what the distribution of body fat was in the current study because CT or MRI scanning was not used to quantify intra-abdominal fat stores. Given that abdominal obesity and intra abdominal fat are greater metabolic risk factors for the metabolic syndrome complications [48,49], it will be important in future studies to evaluate these variables in response to training and CLA.

A large number of studies have looked at the beneficial effects of endurance exercise training on serum markers of metabolic risk including; inflammation (CRP, IL-6, TNF), dysglycemia (HOMA-IR, euglycemic clamps, fasting glucose, hemoglobin A1C), oxidative stress (8-isoP, oxidized LDL), or adipocytokines (leptin, adiponectin) [38,50–56]. Fewer studies have examined the potential for resistance exercise to favorably alter serum variables associated with higher risk of metabolic disease complications [38,57,58]. Two studies have found that resistance exercise reduced hemoglobin A1C in older adults with type 2 diabetes [57,58]. We did not find any evidence of a beneficial influence of training or CrM+CLA on HOMA-IR or fasting blood glucose in the current study. However, this could be due to the fact that none of our older adults had type 2 diabetes, and therefore, the potential for improvement was less. There has been some concern that CLA administration (either the c9, t11 or t10, c12 isomer) could lead to an increase in markers of oxidative stress and that this was responsible for an increase in insulin resistance [33,59]. In contrast, CLA supplements containing combinations of the two major isomers taken together did not show the same negative effects on oxidative stress or systemic inflammation [33]. We found that the women who took the CrM+CLA supplement had higher concentrations of 8-isoprostanes as compared with all other groups. Given that neither this group nor the others showed any evidence of oxidative stress using the DNA marker, renders the biological significance unclear. Our data suggests that a combination of CLA isomers taken during a resistance exercise training program does not negatively influence markers of inflammation

(CRP, IL-6) or dysglycemia (HOMA-IR, fasting glucose) or markers of DNA oxidative damage (8-OH-2dG).

The results of the current study confirmed our earlier finding that resistance exercise training was associated with a reduction in a marker of DNA oxidative stress (8-OH-2dG) [60]. Our previous work using a similar training stimulus indicated that this effect was due to a training induced induction of the anti-oxidant enzymes, catalase and Cu/Zn superoxide dismutase [61]. In contrast, there was no beneficial effect consequent to the training program with respect to oxidative stress measured using the 8-isoprostanes, and the women on CrM+CLA showed an increase in 8-isoprostanes after training. One study found that acute resistance exercise did not alter 8-isoprostanes in blood [62], and another found no difference in 8-isoprostanes in trained athletes compared to sedentary controls [63]. Our data is in general agreement with the latter two studies in that three of our groups showed no significant influence of training on 24 h 8-isoprostane excretion. The biological significance of the increase in 8-isoprostanes for women on CrM+CLA is unclear as is the discrepancy between the 8-OH2dG and the 8-isoprostane results. Our data showing that resistance exercise training leads to an increase in anti-oxidant enzyme activity in older adults renders the 8-OH-2dG results more biologically plausible.

Several studies have found that endurance exercise can favorably improve blood lipid profiles in a direction associated with a lower risk of cardiovascular disease risk (↓ LDL, ↑ HDL) [64], while others have found that up to six months of endurance training had no effect on LDL or total cholesterol values [65,66]. The evidence for resistance exercise to improve blood lipid profiles is less consistent or robust [67]. Other factors such as hormonal status in women can influence the response of LDL to an endurance exercise stimulus with increases only seen for women taking hormone replacement [68]. Another group found that genotype influenced the HDL response to 6 months of endurance exercise training where increases were only seen for a specific genotype in the cholesteryl ester transfer protein allele [65]. In the current study there were significant increases seen for both LDL and total cholesterol in both sexes and in both groups after six months of training, yet the ratio of LDL/HDL was not altered. Given that the trial was six months in duration and we did not have a non-exercised control group, it is possible that this minor alteration represented a normal aging phenomenon. Whether or not longer term supplementation with CLA [29], and/or a longer duration of training would have beneficial effects on lipid profiles is unclear.

Resistance training has been shown to have variable and inconsistent effect upon bone turnover and bone density. Some have shown improvements in markers of bone turnover within 16 weeks of high intensity weight training [69], while others have observed improvements in bone mineral density and osteocalcin after 6 months of high intensity weight training [70], in older adults. In contrast, others have reported no changes in markers of bone turnover following 12 weeks of resistance training in older women [71]. The lack of consistency in the response of bone to resistance training likely results from a number of different factors including differences in training intensity and duration and subject population factors such as aging. Given that the osteogenic response to weight training appears to be blunted in older adults [72], and that changes in bone mineral density in response to any intervention take from 6 months to a year to detect [70], it is possible that a higher intensity or a longer duration may have favorably influenced bone mass and markers in the current study. Creatine monohydrate has been shown to stimulate metabolic activity, differentiation and mineralization in osteoblast-like cell cultures [73]. Two groups have found lower *N*-telopeptide excretion (a marker of bone catabolism) in boys and young men with muscular dystrophy following CrM supplementation [74,75]. In contrast, we did not find any effect of four months of resistance exercise training with or without CrM supplementation on bone mineral density or osteocalcin in older men and women [15]. In the current study we did not find any beneficial effects of either the CrM+CLA supplement or resistance training on markers of bone turnover or bone density measurements.

No participants experienced side effects from the training or the supplementation that led to discontinuance of the study. These findings concur with other studies using progressive weight training in older adults [6,7,13,76], and those with obesity and type 2 diabetes [38,57,58]. We did start with a fairly modest initial intensity of 50% of the 1 RM, which we have found to be safe in the training of older adults in the past [15]. There was a slight increase in serum CK activity with resistance training; however, this was not associated with any pigmenturia, muscle cramps and in no case was the CK elevation greater than 400 U/L (N<220 U/L), and there were no differential increased in CK activity with the CrM+CLA supplement. Given that creatine is converted non-enzymatically to creatinine, we did find the expected and previously reported [15], increase in serum creatinine for the groups taking the supplement containing CrM. As with our previous study [15], we did not find any influence on measured creatinine clearance, which we took to indicate that there were no negative effects on renal function. Furthermore, others have not found any evidence of renal dysfunction with short and long-term CrM administration [15,77–79].

In conclusion, we have confirmed the beneficial effects of resistance exercise training on increasing FFM, strength, and function in older adults and have added novel data showing that a combination of CrM+CLA lead to greater increases in FFM and losses of fat mass. Furthermore, those on the supplement showed greater muscular endurance and no evidence of clinical or serological side effects. Whether such changes are maintained in the longer term is unclear at this point.

SUPPORTING INFORMATION

Protocol S1

Found at: doi:10.1371/journal.pone.0000991.s001 (0.14 MB DOC)

ACKNOWLEDGMENTS

We would like to thank Susie Rothman and Julie Hall for assistance with data collection. The creatine monohydrate was kindly donated by Avicena, Palo, Alta, CA and the CLA was kindly donated by Pharmanutrients, Gurnee, IL. Neither of aforementioned companies donated money or other benefits towards the study or any of the authors up to the time of submission of the current article.

Author Contributions

Conceived and designed the experiments: MT AZ. Performed the experiments: AZ JP AS AA EP BR. Analyzed the data: MT AZ JP AS AA EP BR. Wrote the paper: MT AA EP TD.

REFERENCES

1. Castillo EM, Goodman-Gruen D, Kritz-Silverstein D, Morton DJ, Wingard DL, et al. (2003) Sarcopenia in elderly men and women: the Rancho Bernardo study. Am J Prev Med 25: 226–231.
2. Gillette-Guyonnet S, Nourhashemi F, Andrieu S, Cantet C, Albarede JL, et al. (2003) Body composition in French women 75+years of age: the EPIDOS study. Mech Ageing Dev 124: 311–316.
3. Lau EM, Lynn HS, Woo JW, Kwok TC, Melton LJ, 3rd (2005) Prevalence of and risk factors for sarcopenia in elderly Chinese men and women. J Gerontol A Biol Sci Med Sci 60: 213–216.
4. Charette SL, McEvoy L, Pyka G, Snow-Harter C, Guido D, et al. (1991) Muscle hypertrophy response to resistance training in older women. J Appl Physiol 70: 1912–1916.
5. Taaffe DR, Duret C, Wheeler S, Marcus R (1999) Once-weekly resistance exercise improves muscle strength and neuromuscular performance in older adults. J Am Geriatr Soc 47: 1208–1214.
6. Fiatarone MA, O'Neill EF, Ryan ND, Clements KM, Solares GR, et al. (1994) Exercise training and nutritional supplementation for physical frailty in very elderly people. N Engl J Med 330: 1769–1775.
7. Skelton DA, Young A, Greig CA, Malbut KE (1995) Effects of resistance training on strength, power, and selected functional abilities of women aged 75 and older. J Am Geriatr Soc 43: 1081–1087.
8. Borst SE (2004) Interventions for sarcopenia and muscle weakness in older people. Age Ageing 33: 548–555.
9. Lambert CP, Sullivan DH, Freeling SA, Lindquist DM, Evans WJ (2002) Effects of testosterone replacement and/or resistance exercise on the composition of megestrol acetate stimulated weight gain in elderly men: a randomized controlled trial. J Clin Endocrinol Metab 87: 2100–2106.
10. Newman AB, Kupelian V, Visser M, Simonsick E, Goodpaster B, et al. (2003) Sarcopenia: alternative definitions and associations with lower extremity function. J Am Geriatr Soc 51: 1602–1609.
11. Kreider RB, Ferreira M, Wilson M, Grindstaff P, Plisk S, et al. (1998) Effects of creatine supplementation on body composition, strength, and sprint performance. Med Sci Sports Exerc 30: 73–82.
12. Volek JS, Duncan ND, Mazzetti SA, Staron RS, Putukian M, et al. (1999) Performance and muscle fiber adaptations to creatine supplementation and heavy resistance training. Med Sci Sports Exerc 31: 1147–1156.
13. Chrusch MJ, Chilibeck PD, Chad KE, Davison KS, Burke DG (2001) Creatine supplementation combined with resistance training in older men. Med Sci Sports Exerc 33: 2111–2117.
14. Tarnopolsky MA, Parise G, Yardley NJ, Ballantyne CS, Olatinji S, et al. (2001) Creatine-dextrose and protein-dextrose induce similar strength gains during training. Med Sci Sports Exerc 33: 2044–2052.
15. Brose A, Parise G, Tarnopolsky MA (2003) Creatine supplementation enhances isometric strength and body composition improvements following strength exercise training in older adults. J Gerontol A Biol Sci Med Sci 58: 11–19.
16. Hespel P, Op't Eijnde B, Van Leemputte M, Urso B, Greenhaff PL, et al. (2001) Oral creatine supplementation facilitates the rehabilitation of disuse atrophy and alters the expression of muscle myogenic factors in humans. J Physiol 536: 625–633.
17. Olsen S, Aagaard P, Kadi F, Tufekovic G, Verney J, et al. (2006) Creatine supplementation augments the increase in satellite cell and myonuclei number in human skeletal muscle induced by strength training. J Physiol 573: 525–534.
18. Vierck JL, Icenoggle DL, Bucci L, Dodson MV (2003) The effects of ergogenic compounds on myogenic satellite cells. Med Sci Sports Exerc 35: 769–776.
19. Dangott B, Schultz E, Mozdziak PE (2000) Dietary creatine monohydrate supplementation increases satellite cell mitotic activity during compensatory hypertrophy. Int J Sports Med 21: 13–16.
20. Parise G, Mihic S, MacLennan D, Yarasheski KE, Tarnopolsky MA (2001) Effects of acute creatine monohydrate supplementation on leucine kinetics and mixed-muscle protein synthesis. J Appl Physiol 91: 1041–1047.
21. Willoughby DS, Rosene J (2001) Effects of oral creatine and resistance training on myosin heavy chain expression. Med Sci Sports Exerc 33: 1674–1681.
22. Bermon S, Venembre P, Sachet C, Valour S, Dolisi C (1998) Effects of creatine monohydrate ingestion in sedentary and weight-trained older adults. Acta Physiol Scand 164: 147–155.
23. Eijnde BO, Van Leemputte M, Goris M, Labarque V, Taes Y, et al. (2003) Effects of creatine supplementation and exercise training on fitness in men 55-75 yr old. J Appl Physiol 95: 818–828.
24. Tricon S, Burdge GC, Williams CM, Calder PC, Yaqoob P (2005) The effects of conjugated linoleic acid on human health-related outcomes. Proc Nutr Soc 64: 171–182.
25. Belury MA (2002) Dietary conjugated linoleic acid in health: physiological effects and mechanisms of action. Annu Rev Nutr 22: 505–531.
26. Wendel AA, Belury MA (2006) Effects of conjugated linoleic acid and troglitazone on lipid accumulation and composition in lean and Zucker diabetic fatty (fa/fa) rats. Lipids 41: 241–247.
27. Xu X, Storkson J, Kim S, Sugimoto K, Park Y, et al. (2003) Short-term intake of conjugated linoleic acid inhibits lipoprotein lipase and glucose metabolism but does not enhance lipolysis in mouse adipose tissue. J Nutr 133: 663–667.
28. Sebedio JL, Angioni E, Chardigny JM, Gregoire S, Juaneda P, et al. (2001) The effect of conjugated linoleic acid isomers on fatty acid profiles of liver and adipose tissues and their conversion to isomers of 16:2 and 18:3 conjugated fatty acids in rats. Lipids 36: 575–582.
29. Gaullier JM, Halse J, Hoye K, Kristiansen K, Fagertun H, et al. (2005) Supplementation with conjugated linoleic acid for 24 months is well tolerated by and reduces body fat mass in healthy, overweight humans. J Nutr 135: 778–784.
30. Blankson H, Stakkestad JA, Fagertun H, Thom E, Wadstein J, et al. (2000) Conjugated linoleic acid reduces body fat mass in overweight and obese humans. J Nutr 130: 2943–2948.
31. Gaullier JM, Halse J, Hoye K, Kristiansen K, Fagertun H, et al. (2004) Conjugated linoleic acid supplementation for 1 y reduces body fat mass in healthy overweight humans. Am J Clin Nutr 79: 1118–1125.
32. Kreider RB, Ferreira MP, Greenwood M, Wilson M, Almada AL (2002) Effects of conjugated linoleic acid supplementation during resistance training on body composition, bone density, strength, and selected hematological markers. J Strength Cond Res 16: 325–334.
33. Riserus U, Basu S, Jovinge S, Fredrikson GN, Arnlov J, et al. (2002) Supplementation with conjugated linoleic acid causes isomer-dependent oxidative stress and elevated C-reactive protein: a potential link to fatty acid-induced insulin resistance. Circulation 106: 1925–1929.
34. Basu S, Riserus U, Turpeinen A, Vessby B (2000) Conjugated linoleic acid induces lipid peroxidation in men with abdominal obesity. Clin Sci (Lond) 99: 511–516.
35. Kelley DS, Bartolini GL, Warren JM, Simon VA, Mackey BE, et al. (2004) Contrasting effects of t10,c12- and c9,t11-conjugated linoleic acid isomers on the fatty acid profiles of mouse liver lipids. Lipids 39: 135–141.
36. Kim HK, Kim SR, Ahn JY, Cho IJ, Yoon CS, et al. (2005) Dietary conjugated linoleic acid reduces lipid peroxidation by increasing oxidative stability in rats. J Nutr Sci Vitaminol (Tokyo) 51: 8–15.
37. Singh B, Mallika V, Goswami B (2006) Metabolic syndrome: Diagnosis, potential markers and management-an update. Clin Chim Acta.
38. Fatouros IG, Tournis S, Leontsini D, Jamurtas AZ, Sxina M, et al. (2005) Leptin and adiponectin responses in overweight inactive elderly following resistance training and detraining are intensity related. J Clin Endocrinol Metab 90: 5970–5977.
39. Tarnopolsky MA, Roy BD, MacDonald JR (1997) A randomized, controlled trial of creatine monohydrate in patients with mitochondrial cytopathies. Muscle Nerve 20: 1502–1509.
40. Rodriguez MC, Macdonald JR, Mahoney DJ, Parise G, Beal MF, et al. (2006) Beneficial effects of creatine, CoQ(10), and lipoic acid in mitochondrial disorders. Muscle Nerve.
41. Mackey AL, Esmarck B, Kadi F, Koskinen SO, Kongsgaard M, et al. (2007) Enhanced satellite cell proliferation with resistance training in elderly men and women. Scand J Med Sci Sports 17: 34–42.
42. Dreyer HC, Blanco CE, Sattler FR, Schroeder ET, Wiswell RA (2006) Satellite cell numbers in young and older men 24 hours after eccentric exercise. Muscle Nerve 33: 242–253.
43. Luukinen H, Koski K, Laippala P, Kivela SL (1997) Factors predicting fractures during falling impacts among home-dwelling older adults. J Am Geriatr Soc 45: 1302–1309.
44. Sturnieks DL, Tiedemann A, Chapman K, Munro B, Murray SM, et al. (2004) Physiological risk factors for falls in older people with lower limb arthritis. J Rheumatol 31: 2272–2279.
45. Whigham LD, O'Shea M, Mohede IC, Walaski HP, Atkinson RL (2004) Safety profile of conjugated linoleic acid in a 12-month trial in obese humans. Food Chem Toxicol 42: 1701–1709.
46. Takahashi Y, Kushiro M, Shinohara K, Ide T (2003) Activity and mRNA levels of enzymes involved in hepatic fatty acid synthesis and oxidation in mice fed conjugated linoleic acid. Biochim Biophys Acta 1631: 265–273.
47. Wang YW, Jones PJ (2004) Conjugated linoleic acid and obesity control: efficacy and mechanisms. Int J Obes Relat Metab Disord 28: 941–955.
48. Onat A, Avci GS, Barlan MM, Uyarel H, Uzunlar B, et al. (2004) Measures of abdominal obesity assessed for visceral adiposity and relation to coronary risk. Int J Obes Relat Metab Disord 28: 1018–1025.
49. Busetto L (2001) Visceral obesity and the metabolic syndrome: effects of weight loss. Nutr Metab Cardiovasc Dis 11: 195–204.
50. Marcell TJ, McAuley KA, Traustadottir T, Reaven PD (2005) Exercise training is not associated with improved levels of C-reactive protein or adiponectin. Metabolism 54: 533–541.
51. Short KR, Vittone JL, Bigelow ML, Proctor DN, Rizza RA, et al. (2003) Impact of aerobic exercise training on age-related changes in insulin sensitivity and muscle oxidative capacity. Diabetes 52: 1888–1896.
52. O'Leary VB, Marchetti CM, Krishnan RK, Stetzer BP, Gonzalez F, et al. (2006) Exercise-induced reversal of insulin resistance in obese elderly is associated with reduced visceral fat. J Appl Physiol 100: 1584–1589.
53. Shadid S, Stehouwer CD, Jensen MD (2006) Diet/Exercise versus pioglitazone: effects of insulin sensitization with decreasing or increasing fat mass on adipokines and inflammatory markers. J Clin Endocrinol Metab 91: 3418–3425.
54. Monzillo LU, Hamdy O, Horton ES, Ledbury S, Mullooly C, et al. (2003) Effect of lifestyle modification on adipokine levels in obese subjects with insulin resistance. Obes Res 11: 1048–1054.
55. Bruun JM, Helge JW, Richelsen B, Stallknecht B (2006) Diet and exercise reduce low-grade inflammation and macrophage infiltration in adipose tissue but

not in skeletal muscle in severely obese subjects. Am J Physiol Endocrinol Metab 290: E961–967.

56. Bluher M, Bullen JW Jr, Lee JH, Kralisch S, Fasshauer M, et al. (2006) Circulating adiponectin and expression of adiponectin receptors in human skeletal muscle: associations with metabolic parameters and insulin resistance and regulation by physical training. J Clin Endocrinol Metab 91: 2310–2316.
57. Dunstan DW, Daly RM, Owen N, Jolley D, De Courten M, et al. (2002) High-intensity resistance training improves glycemic control in older patients with type 2 diabetes. Diabetes Care 25: 1729–1736.
58. Castaneda C, Layne JE, Munoz-Orians L, Gordon PL, Walsmith J, et al. (2002) A randomized controlled trial of resistance exercise training to improve glycemic control in older adults with type 2 diabetes. Diabetes Care 25: 2335–2341.
59. Riserus U, Vessby B, Arnlov J, Basu S (2004) Effects of cis-9,trans-11 conjugated linoleic acid supplementation on insulin sensitivity, lipid peroxidation, and proinflammatory markers in obese men. Am J Clin Nutr 80: 279–283.
60. Parise G, Brose AN, Tarnopolsky MA (2005) Resistance exercise training decreases oxidative damage to DNA and increases cytochrome oxidase activity in older adults. Exp Gerontol 40: 173–180.
61. Parise G, Phillips SM, Kaczor JJ, Tarnopolsky MA (2005) Antioxidant enzyme activity is up-regulated after unilateral resistance exercise training in older adults. Free Radic Biol Med 39: 289–295.
62. McAnulty SR, McAnulty LS, Nieman DC, Morrow JD, Utter AC, et al. (2005) Effect of resistance exercise and carbohydrate ingestion on oxidative stress. Free Radic Res 39: 1219–1224.
63. Watson TA, MacDonald-Wicks LK, Garg ML (2005) Oxidative stress and antioxidants in athletes undertaking regular exercise training. Int J Sport Nutr Exerc Metab 15: 131–146.
64. Halbert JA, Silagy CA, Finucane P, Withers RT, Hamdorf PA (1999) Exercise training and blood lipids in hyperlipidemic and normolipidemic adults: a meta-analysis of randomized, controlled trials. Eur J Clin Nutr 53: 514–522.
65. Wilund KR, Ferrell RE, Phares DA, Goldberg AP, Hagberg JM (2002) Changes in high-density lipoprotein-cholesterol subfractions with exercise training may be dependent on cholesteryl ester transfer protein (CETP) genotype. Metabolism 51: 774–778.
66. Banz WJ, Maher MA, Thompson WG, Bassett DR, Moore W, et al. (2003) Effects of resistance versus aerobic training on coronary artery disease risk factors. Exp Biol Med (Maywood) 228: 434–440.
67. Boyden TW, Pamenter RW, Going SB, Lohman TG, Hall MC, et al. (1993) Resistance exercise training is associated with decreases in serum low-density lipoprotein cholesterol levels in premenopausal women. Arch Intern Med 153: 97–100.
68. Green JS, Stanforth PR, Rankinen T, Leon AS, Rao Dc D, et al. (2004) The effects of exercise training on abdominal visceral fat, body composition, and indicators of the metabolic syndrome in postmenopausal women with and without estrogen replacement therapy: the HERITAGE family study. Metabolism 53: 1192–1196.
69. Sartorio A, Lafortuna C, Capodaglio P, Vangeli V, Narici MV, et al. (2001) Effects of a 16-week progressive high-intensity strength training (HIST) on indexes of bone turnover in men over 65 years: a randomized controlled study. J Endocrinol Invest 24: 882–886.
70. Vincent KR, Braith RW (2002) Resistance exercise and bone turnover in elderly men and women. Med Sci Sports Exerc 34: 17–23.
71. Mullins NM, Sinning WE (2005) Effects of resistance training and protein supplementation on bone turnover in young adult women. Nutr Metab (Lond) 2: 19.
72. Kohrt WM (2001) Aging and the osteogenic response to mechanical loading. Int J Sport Nutr Exerc Metab 11 Suppl: S137–142.
73. Gerber I, ap Gwynn I, Alini M, Wallimann T (2005) Stimulatory effects of creatine on metabolic activity, differentiation and mineralization of primary osteoblast-like cells in monolayer and micromass cell cultures. Eur Cell Mater 10: 8–22.
74. Tarnopolsky MA, Mahoney DJ, Vajsar J, Rodriguez C, Doherty TJ, et al. (2004) Creatine monohydrate enhances strength and body composition in Duchenne muscular dystrophy. Neurology 62: 1771–1777.
75. Louis M, Lebacq J, Poortmans JR, Belpaire-Dethiou MC, Devogelaer JP, et al. (2003) Beneficial effects of creatine supplementation in dystrophic patients. Muscle Nerve 27: 604–610.
76. Chilibeck PD, Davison KS, Whiting SJ, Suzuki Y, Janzen CL, et al. (2002) The effect of strength training combined with bisphosphonate (etidronate) therapy on bone mineral, lean tissue, and fat mass in postmenopausal women. Can J Physiol Pharmacol 80: 941–950.
77. Mihic S, MacDonald JR, McKenzie S, Tarnopolsky MA (2000) Acute creatine loading increases fat-free mass, but does not affect blood pressure, plasma creatinine, or CK activity in men and women. Med Sci Sports Exerc 32: 291–296.
78. Poortmans JR, Auquier H, Renaut V, Durussel A, Saugy M, et al. (1997) Effect of short-term creatine supplementation on renal responses in men. Eur J Appl Physiol Occup Physiol 76: 566–567.
79. Poortmans JR, Francaux M (1999) Long-term oral creatine supplementation does not impair renal function in healthy athletes. Med Sci Sports Exerc 31: 1108–1110.
