# Supplementary material for: Impact of creatine supplementation on inflammation: evidence from a systematic review and meta-analysis of randomized double-blind placebo trials
Source: Front Immunol. 2026 Feb 19;17:1743603. doi: 10.3389/fimmu.2026.1743603 (PMC12961398; doi:10.3389/fimmu.2026.1743603)
Supplement: Supplementary file 2 [file SupplementaryFile1.zip › SR Creatine inflammatory markers (Kell Doutorado). /Supplementary Files/Final References/Final/Taes et al 2004.pdf]

Kidney International, Vol. 66 (2004), pp. 2422–2428

Creatine supplementation does not decrease total plasma homocysteine in chronic hemodialysis patients

YOURI E.C. TAES, JORIS R. DELANGHE, DIRK DE BACQUER, MICHEL LANGLOIS, LUT STEVENS, INGE GEEROLF, NORBERT H. LAMEIRE, and AN S. DE VRIESE

Laboratory Clinical Chemistry, University Hospital Ghent, Belgium; Renal Unit, Department of Internal Medicine, University Hospital Ghent, Belgium; Department of Public Health, Ghent University, Ghent, Belgium; Laboratory Clinical Chemistry, Sint-Jan AV, Brugge, Belgium; and Renal Unit, Sint-Jan AV, Brugge, Belgium

Creatine supplementation does not decrease total plasma homocysteine in chronic hemodialysis patients.

Background. Hyperhomocysteinemia is present in the majority of chronic hemodialysis patients. Treatment with folic acid, vitamin B12, and vitamin B6 cannot fully normalize plasma homocysteine concentrations (tHcy). Previously we have demonstrated the tHcy-lowering effect of creatine supplementation in an animal model of uremia (*Kidney Int* 64:1331–1337, 2003). The present study investigates the effects of creatine supplementation on tHcy in a vitamin-repleted chronic hemodialysis population.

Methods. Forty-five hemodialysis patients receiving folic acid and vitamin B6 and B12 were included. Patients were treated with creatine (2 g/day) or placebo during 2 treatment periods of 4 weeks, separated by a washout of 4 weeks. Plasma tHcy, creatine, Kt/V_{urea}, folic acid, vitamin B12, and routine biochemistry were determined, as well as the prognostic inflammatory and nutritional index.

Results. All patients had elevated tHcy concentrations (21.2 ± 5.6 μmol/L). Creatine treatment resulted in increased plasma and red blood cell creatine levels, documenting uptake of creatine. Creatine did not affect tHcy concentrations. There was no relationship between plasma creatine concentrations and tHcy concentrations. No changes in body weight, routine biochemistry, nutritional status, folic acid, or vitamin B12 were observed during the study.

Conclusion. Creatine supplementation at a rate of 2 g/day does not further decrease tHcy concentrations in chronic dialysis patients already treated with high dose folic acid, vitamin B6, and B12 supplementation.

Hyperhomocysteinemia, a raised plasma concentration of the sulfur amino acid homocysteine, is observed in several genetic and acquired disorders. The majority of patients with end-stage renal disease (ESRD) have a moderate degree of hyperhomocysteinemia [1]. Treatment of hyperhomocysteinemia consists of folic acid and vitamin B6, B12 supplements. Folic acid supplementation can reduce plasma tHcy about 30% in uremic patients, whereas the effect of vitamin B6 and B12 is in general much less pronounced [2, 3]. However, the majority of ESRD patients maintain elevated plasma tHcy concentrations when treated with high dose multivitamin supplementation. Betaine can further decrease post-methionine loading tHcy concentrations by 18%, in addition to folic acid supplementation [4]. Additional tHcy-lowering therapies are necessary in these patients in order to attain normal tHcy concentrations.

Creatine synthesis and homocysteine (Hcy)-formation are metabolically connected (Fig. 1). Hcy [1, 5] is derived from methionine in a multiple step metabolic cycle (Fig. 1). Methionine can intracellularly be converted to S-adenosyl methionine, a sulfonium compound with a highly reactive methyl group, which acts as a universal methyl donor in numerous transmethylation reactions in vivo. The demethylated product S-adenosylhomocysteine, a thioether, is readily hydrolyzed to Hcy and adenosine by S-adenosyl-homocysteine hydrolase. Transsulfuration of homocysteine to yield cystathionine is favored in conditions with methionine excess. Remethylation of homocysteine to methionine occurs in conditions of low methionine intake through methyl-donation from 5-methyltetrahydrofolate (5-CH_3THF) by means of methionine synthase. Creatine is synthesized in humans by two successive metabolic steps (Fig. 1). Guanidinoacetate is synthesized from glycine and arginine by arginine: glycine aminidinotransferase (AGAT; EC 2.1.4.1), mainly in the kidney. Second, guanidinoacetate is methylated in the liver to creatine by guanidinoacetate-methyltransferase (GAMT) (EC 2.1.1.2) with S-adenosylmethionine as methyl-donor [6, 7]. Dietary intake and endogenous synthesis in liver compensate for a daily loss as creatinine of about 2%. Creatine supplementation represses AGAT biosynthesis and,

Key words: homocysteine, creatine, folic acid, end-stage renal disease.

Received for publication March 17, 2004
and in revised form May 6, 2004, and June 14, 2004
Accepted for publication June 22, 2004

© 2004 by the International Society of Nephrology

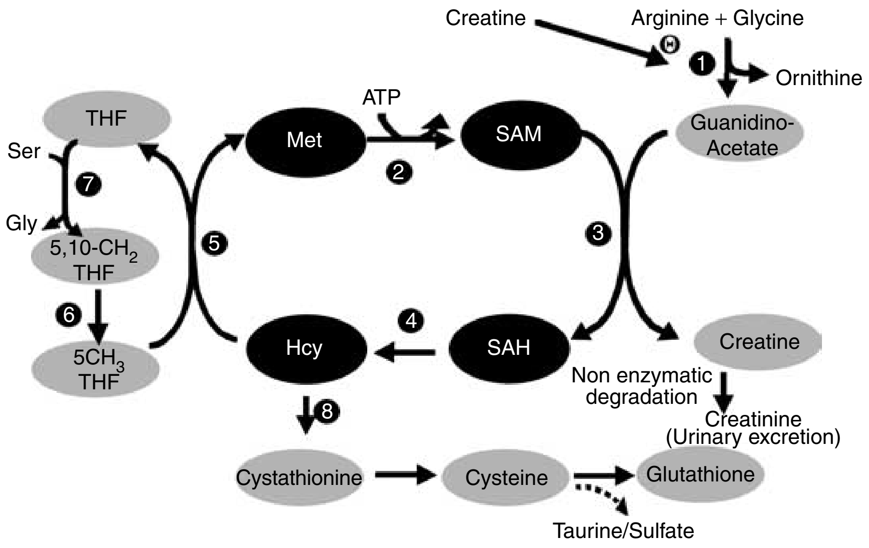

Fig. 1. Overview of homocysteine and creatine metabolism. THF: tetrahydrofolate; $5CH_3$-THF: 5-methyltetrahydrofolate; 5,10-CH_2-THF: 5,10 methylene tetrahydrofolate; Ser: serine; Gly: glycine; SAM: S-adenosyl methionine; SAH: S-adenosyl homocysteine; Hcy: homocysteine; Met: methionine. Enzymes are (1) arginine:glycine amidinotransferase (AGAT); (2) methionine adenosyltransferase; (3) guanidinoacetate methyltransferase (GAMT); (4) S-adenosyl homocysteine hydrolase; (5) methionine synthase; (6) 5,10-CH2-THF reductase; (7) serine-hydroxy methyltransferase; (8) cystathionine-β–synthase.

consequently, guanidinoacetate and creatine formation (Fig. 1). Methylation of guanidinoacetate during creatine biosynthesis has been estimated to account for up to 70% of the transmethylation reactions in the body with formation of Hcy [8, 9]. Exogenous creatine supplementation can, thus, be expected to decrease endogenous Hcy synthesis. Inhibition of the methyltransferase reactions has been described in uremia due to a reduced S-adenosylmethionine (SAM)/S-adenosylhomocysteine (SAH) ratio, and protein hypomethylation and reduced protein repair are observed in uremic subjects [10, 11]. The influence of creatine on tHcy concentrations, SAM/SAH ratios, and the methyltransferase reactions in humans is currently unknown. Creatine supplementation could reduce the guanidinoacetate-methyltransferase reaction and consequent Hcy-formation. In an animal model of chronic renal failure we have recently demonstrated a pronounced beneficial effect of creatine supplementation on plasma tHcy concentrations. tHcy concentrations were lowered by 22% in creatine-treated animals compared to control diet–fed uremic animals [12]. Whether creatine can lower tHcy in humans as observed in our animal model is currently unknown.

The present study investigates the effect of creatine supplementation on plasma tHcy concentrations in chronic hemodialysis patients already treated with high-dose multivitamin supplementation.

METHODS

Study design and population

Forty-nine hemodialysis patients were recruited. Dialysis was performed with a low-flux triacetate dialyzer (Sureflux-L; Nipro, Osaka, Japan) for 4 to 5 hours 3 times weekly for at least 3 months. Exclusion criteria were: acute illness, life expectancy <3 months, low compliance

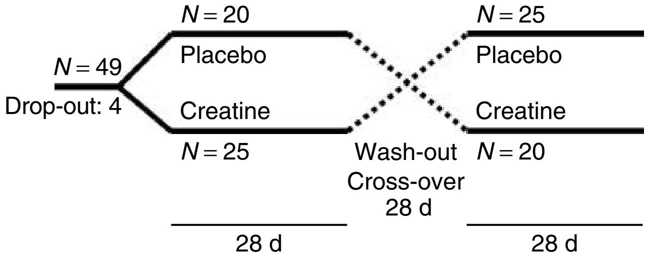

Fig. 2. Treatment schedule.

due to cognitive, social, or psychiatric problems, and inability to provide informed consent. All patients were treated with folic acid 5 mg, pyridoxine 50 mg, and vitamin B12 12 μg orally 3 times a week for at least 3 months. Written informed consent was obtained from all participants. The study protocol was approved by the Ethical Committee of AZ Sint-Jan AV. Sample size estimation (type I error = 0.05; type II error = 0.20), based on the Hcy lowering in our animal study (Δ tHcy = 20%) and tHcy concentrations in dialysis patients, revealed sufficient statistical power of the present study.

The study followed a double-blind, placebo-controlled, crossover design (Fig. 2). Patients received 2 g creatine or placebo daily in the evening during 2 treatment periods of 4 weeks, in random order, and separated by a washout period of 4 weeks. Creatine monohydrate (CreaPure®) was obtained from Degussa Bioactives (Freising, Germany). Placebo tablets contained Fast Flo lactose (Foremost Farms, Baraboo, WI, USA). An exact number of tablets was supplied, and pills were counted to ascertain compliance. Treatment duration, blood flow, dialysate flow, and membrane surface area were determined by the attending nephrologists, but no changes were made during the study period. Single pool Kt/V_{urea} was calculated at the start and end of the study, where K =

dialyzer urea clearance, T = duration of dialysis, and V = urea distribution volume at the end of dialysis.

Predialysis blood samples were obtained at baseline and after 4 weeks in both treatment periods. EDTA-samples for tHcy determinations were transported on ice, centrifuged immediately, and stored at −20°C until testing.

Biochemical determinations

Plasma creatinine concentrations were determined using a compensated rate-blanked Jaffé based method (Roche Diagnostics, Mannheim, Germany) on a Modular P analyzer (Roche Diagnostics) according to the manufacturer's procedure. Plasma urea concentrations and aspartate-aminotransferase (AST), alanine-aminotransferase (ALT), alkaline phosphatase, creatine kinase, and γ-glutamyl transferase (GGT) activities were determined on a Modular P analyzer using commercial reagents (Roche Diagnostics).

Total plasma homocysteine concentrations were determined using a fluorescence polarization immunoassay on an Axsym analyzer (Abbott Laboratories, Abbott Park, IL, USA).

Plasma folate and vitamin B12 concentrations were determined using an electrochemiluminescence assay (Roche Diagnostics) on an Elecsys 2010 analyzer (Roche Diagnostics). Plasma and erythrocyte creatine concentrations were determined enzymatically as described before [13]. Plasma creatine was determined without deproteinization, whereas erythrocyte creatine was determined after deproteinization with 5-sulfosalicylic acid. No analytical interference of creatine on tHcy was observed. The prognostic inflammatory and nutritional index (PINI) was calculated as PINI = [(α_1-acid glycoprotein (mg/L) × CRP (mg/L)]/[(albumin (g/L) × prealbumin (mg/L)] [14, 15]. α_1-acid glycoprotein, albumin, and prealbumin were determined nephelometrically using commercial reagents (Dade Behring, Marburg, Germany) on a BN II nephelometer. CRP was determined turbidimetrically on a Modular P analyzer (Roche Diagnostics).

Statistics

Data are expressed as mean ± SD, unless parameters were not normally distributed [median (interquartile range)]. Student *t* test was used to compare separate groups when appropriate; otherwise nonparametric group comparison was performed using Mann-Whitney *U* test. Associations between continuous variables were examined using Spearman rank correlation analysis. In order to estimate the effect of creatine treatment on the tHcy concentrations in this crossover framework, a mixed effect model was fitted treating patient as a random effect while the treatment mode, period, and carry-over were modeled as fixed effects [16]. Model fitting was performed using SAS software (PROC MIXED) (release 8.1, SAS Institute, Inc., Cary, NC, USA). Differences were considered significant at P less than 0.05.

Table 1. Baseline patient characteristics

	Placebo	Creatine
N	20	25
Male/Female	10/10	14/11
Age *years*	69 ± 12	71 ± 8
Body mass index *kg/m*^{2}	24 ± 4	25 ± 3
Plasma creatinine *μmol/L*	751 ± 203	698 ± 221
Plasma urea *mmol/L*	22.3 ± 5.7	22.5 ± 6.5
Kt/V_{urea}	1.4 ± 0.4	1.3 ± 0.2

RESULTS

Baseline patient characteristics

Forty-five hemodialysis patients (24 males, 21 females) with a mean age of 70 ± 10 years (range 35–88) were included in this study. Four patients were excluded from the study (2 patients were transplanted during the study, 1 died, and 1 quit the study). Twenty-five patients received creatine, and 20 patients received placebo in the first treatment period. No baseline differences in age, gender distribution, body mass index, tHcy concentrations, or dialysis parameters were observed between placebo and creatine groups for both treatment periods (Table 1).

Plasma tHCY and creatine concentrations

No significant changes in plasma tHcy concentrations were observed during treatment with creatine or placebo. Pre- and post-treatment tHcy concentrations were comparable in both creatine or placebo groups during the 2 treatment periods (Fig. 3). Plasma and red cell creatine concentrations were significantly elevated in the creatine treated groups, whereas comparable concentrations were observed before and after placebo treatment, documenting the uptake of creatine (Fig. 3 and Table 3).

No difference in baseline creatine concentrations was observed between the first and second treatment period, documenting the washout effect (Fig. 3). Male patients had significantly higher tHcy concentrations (23.3 ± 3.7 μmol/L) compared to female patients (18.7 ± 6.1 μmol/L; $P = 0.005$). No effect of creatine supplementation on tHcy concentrations was observed in either male or female patients (data not shown).

The results of the mixed effects model fit for tHcy are shown in Table 2. No treatment, period, or carryover effects were detected. No correlation between plasma creatine and tHcy concentrations was observed ($r = -0.08$; $P = 0.76$).

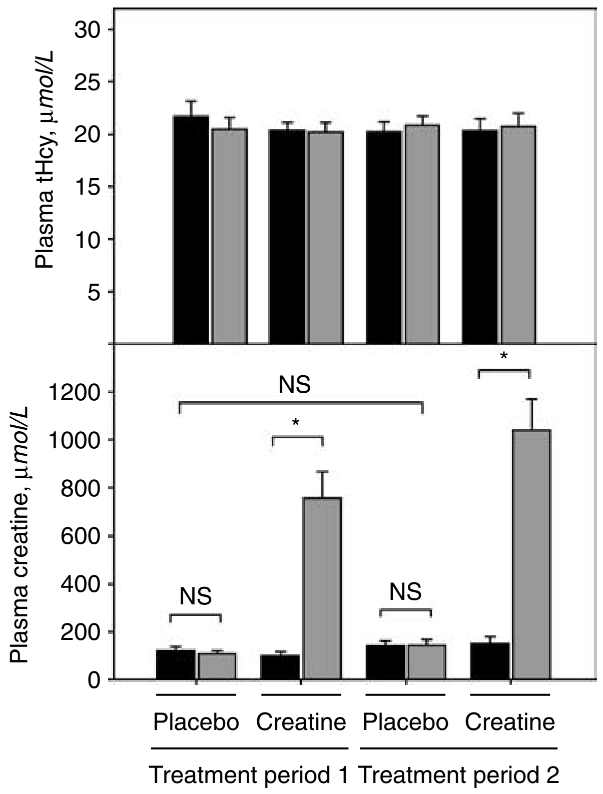

Fig. 3. Plasma homocysteine (tHcy) and creatine concentrations in both treatment periods. Black bars represent concentrations before treatment, whereas gray bars represent concentrations after treatment. Creatine concentrations were significantly higher in the creatine groups compared to the placebo groups ($*P < 0.0001$). No difference in tHcy concentrations was observed.

Table 2. Results of the mixed effects model fit for tHcy

	β (SE)	t value	Significance
Treatment (creatine vs. placebo)	−0.253 (0.634)	−0.40	$P = 0.69$
Carryover effect	−0.259 (1.409)	−0.18	$P = 0.86$
Period (2nd vs. 1st)	−0.393 (0.634)	−0.62	$P = 0.54$

Plasma folate and vitamin B12 concentrations

All patients had elevated plasma concentrations of folate and vitamin B12 (Table 3). No difference in plasma folate or vitamin B12 was observed between creatine- or placebo-treated groups. No significant relationship between tHcy and plasma folate or vitamin B12 concentrations was observed.

Nutritional status and dialysis

Nutritional status remained constant during the study period. No difference over time in total protein, albumin, prealbumin concentrations, and PINI scores were observed in any of the groups. A significant relationship between total protein concentration and tHcy was observed ($r = 0.20$; $P = 0.006$). No relationship between albumin ($r = 0.103$; $P = 0.17$), prealbumin ($r = 0.10$; $P = 0.20$) concentrations, or PINI scores ($r = 0.01$; $P = 0.87$) and tHcy was observed in this population. Body weight and Kt/V_{urea} remained constant during the study period. No difference in Kt/V_{urea} between creatine- and placebo-treated groups [1.3 ± 0.3 vs. 1.4 ± 0.3 (post-treatment)] was observed. Baseline tHcy concentrations correlated with Kt/V_{urea} ($r = -0.35$; $P = 0.01$) and plasma creatinine concentrations ($r = 0.40$; $P = 0.004$) (Fig. 4). No relationship after treatment was observed between Kt/V_{urea} and tHcy concentrations.

DISCUSSION

Hcy metabolism is tightly regulated under normal conditions. However, in situations with compromised Hcy homeostasis such as dialysis, creatine supplementation could have a more prominent tHcy-lowering effect [17]. In our dialysis patients treated with folic acid, vitamin B6, and B12, plasma tHcy concentrations remained moderately elevated, an observation in agreement with the well-known resistance to therapy in uremia. In contrast to the effects in experimental animals [12, 18], oral creatine supplementation at a rate of 2 g/day did not lower plasma tHcy concentrations in our dialysis population. tHcy concentrations were comparable after treatment with creatine and placebo. This observation is in line with the absence of an effect of creatine supplementation on tHcy in young healthy volunteers [19].

Plasma creatine and creatinine concentrations were higher in creatine-supplemented subjects, documenting uptake and metabolization of creatine. The absence of effect could, thus, not be attributed to a limited uptake of creatine. In addition, the sample size was sufficiently large to demonstrate a decrease in plasma tHcy as observed previously in our animal study [12]. The dose of 2g creatine daily was chosen on the basis of existing treatment schedules. In healthy subjects, creatine at a maintenance dose of 2 g is used as a performance enhancing nutritional supplement, with documented uptake of creatine in the skeletal muscle [20]. Loading doses up to 20 g/day are used by athletes. We have chosen not to use a loading dose to avoid extremely high plasma concentrations with potential side effects.

In contrast to our experimental animals, our patient population was treated with a chronic high-dose multivitamin regimen. High-dose folic acid supplementation could mask the Hcy-lowering effect of creatine in this population. A clinically relevant tHcy-lowering treatment should be able to decrease tHcy levels in vitamin-replete patients. Theoretically, lowering methylation demand could be synergistic with folic acid supplementation, which enhances the remethylation pathway. In contrast to these theoretic expectations, a decreased methylation demand by exogenous creatine supplementation did not lower tHcy concentrations, despite documented uptake and metabolization of creatine.

Table 3. Biochemical variables in both treatment groups

	Ref. range	Period 1 Placebo	Period 1 Creatine	Period 2 Placebo	Period 2 Creatine
Body weight *kg*		68 ± 17	69 ± 10	69 ± 11	68 ± 17
Red cell creatine *mmol/L*		0.30 ± 0.06	0.42 ± 0.11[b]	0.25 ± 0.06	0.39 ± 0.06[b]
Plasma creatinine *μmol/L*	49–103	751 ± 194	884 ± 292[c]	769 ± 230	840 ± 239
Plasma urea *mmol/L*	2.8–8.0	23.3 ± 6.7	25.0 ± 6.7	26.7 ± 5.0	25.0 ± 6.7
AST *U/L*[a]	0–37	17 (15–21)	16 (14–23)	16 (14–22)	16 (15–19)
ALT *U/L*	13–45	15 (11–19)	15 (12–19)	13 (12–19)	14 (11–17)
Alkaline phosphatase *U/L*[a]	56–119	81 (60–103)	92 (75–122)	92 (75–119)	92 (62–120)
GGT *U/L*[a]	11–49	22 (18–26)	21 (14–38)	20 (14–44)	18 (17–27)
Creatine kinase *U/L*[a]	10–195	67 (42–78)	71 (37–99)	60 (33–87)	67 (52–78)
CRP *mg/L*[a]	<5	5 (2–10)	3 (1–8)	3 (1–9)	6 (1–10)
α_1-acid glycoprotein *g/L*	0.5–1.3	1.00 ± 0.25	0.98 ± 0.28	1.01 ± 0.27	1.01 ± 0.26
Albumin *g/L*	34–48	40.1 ± 4.7	39.5 ± 5.6	39.9 ± 4.8	40.0 ± 4.0
Prealbumin *g/L*	0.2–0.4	0.26 ± 0.07	0.28 ± 0.10	0.29 ± 0.08	0.25 ± 0.06
PINI[a]	<1.0	0.47 (0.13–1.0)	0.25 (0.07–0.76)	0.25 (0.09–0.91)	0.42 (0.14–1.3)
Total protein *g/L*	66–83	67 ± 3.9	66 ± 4.5	66 ± 4.4	66 ± 35
Folate *nmol/L*	4.5–20.3	69.9 ± 13.4	63.2 ± 15.6	66.1 ± 19.1	73.0 ± 14.9
Vitamin B12 *pmol/L*	145–639	579 ± 192	483 ± 199	581 ± 190	636 ± 157

Abbreviations are: PINI, prognostic inflammatory and nutritional index; AST, aspartate-aminotransferase; ALT, alanine-aminotransferase; GGT, γ-glutamyl transferase.
[a]Non-Gaussian distribution, data expressed as median (interquartile range).
[b]$P < 0.0001$.
[c]$P < 0.01$, creatine treatment compared to placebo treatment in both treatment periods.

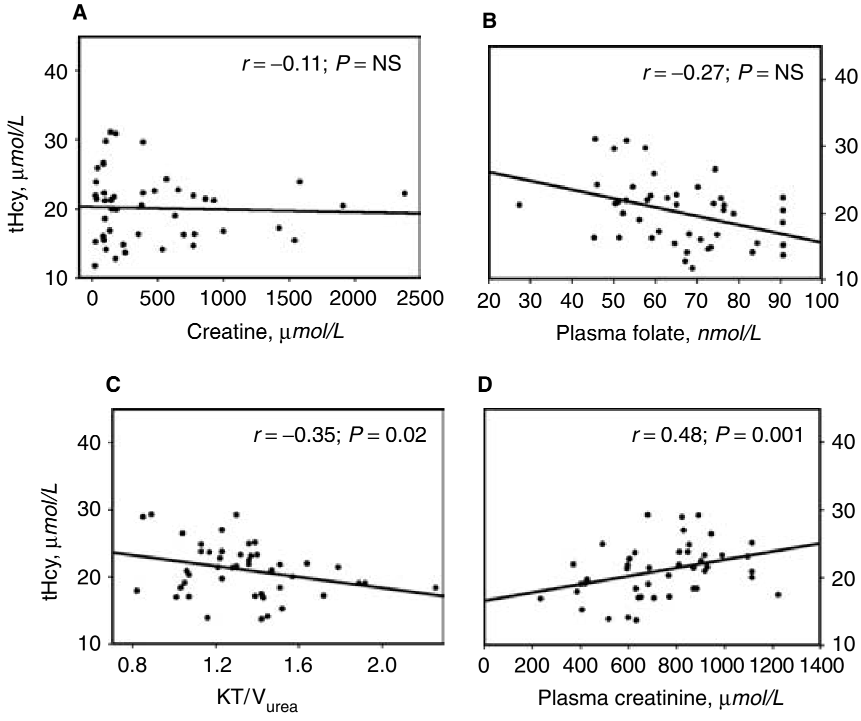

Fig. 4. Relationship between plasma homocysteine (tHcy) and plasma creatine (*A*), plasma folate (*B*), Kt/V_{urea} (*C*), and plasma creatinine concentrations (*D*). (A) and (B) represent values (homocysteine, creatine, folate) after treatment; (C) and (D) represent baseline values (homocysteine, creatinine, Kt/V_{urea}).

In our patient population, creatine supplementation did not alter plasma folate concentrations. High-dose folic acid supplementation could have masked the folate sparing action of creatine that we have observed in our animal model. Lowering methyl group consumption by creatine supplementation apparently no longer influences tHcy or folate concentrations in conditions of excess folate [12].

Species-related differences in metabolic pathways could perhaps explain the difference in effect between humans and rodents. In rats, about 75% of plasma tHcy was found to be free, whereas in humans, about 65% to 75% of tHcy is bound to protein by a disulfide bond [21, 22]. In dialysis patients, protein binding of Hcy was even found to be higher in comparison to nondialyzed chronic uremic patients [23]. These differences in protein binding

could be reflected in differences found in renal extraction of Hcy in humans and rats. In rats, significant clearance of Hcy by the kidneys was observed [22], whereas in humans, no arteriovenous difference in Hcy concentrations was observed [24]. Differences in renal handling and in general Hcy metabolism could account for the absence of effect of creatine supplementation in our study. Aside from dissimilarities in Hcy-metabolism, creatine could be metabolized differently in rats than in humans. Compartmentalization and possibly regulation of the different enzymes necessary for creatine biosynthesis differs substantially between species [6, 25].

In uremia, a disturbed SAM/SAH ratio inhibits the methyltransferase reactions. Creatine supplementation has been described to reduce the guanidinoacetate-methyltransferase reaction by lowering guanidinoacetate concentrations. The influence of creatine on other methyltransferase reactions (e.g., DNA-, protein methyltransferases) is currently unknown. Because creatine supplementation fails to lower tHcy concentrations in our dialysis population, the effect on other methyltransferase reactions will probably be limited in humans.

The safety of creatine supplementation and the absence of adverse effects on renal function were previously assessed by our group in an animal model of chronic renal failure [26]. No adverse effects on glomerular filtration or renal protein handling were observed. Although adverse effects of creatine have been suggested, none of the controlled studies in humans on the effects of creatine supplementation have revealed any [27]. In our hemodialysis population we did not observe any major adverse effect of creatine administration. Plasma creatinine concentrations were slightly elevated due to increased creatine load in the creatine groups compared to the placebo groups. No effect on body weight or liver enzymes was noted.

Protein malnutrition is common in patients on maintenance dialysis. Malnutrition and hypoalbuminemia are risk factors for increased morbidity and mortality in patients with ESRD [28, 29]. Nutritional status influences plasma tHcy concentrations by the strong protein binding of Hcy to albumin, and by the increased Hcy formation upon methionine uptake from the diet. The PINI is a formula to evaluate nutritional status and prognosis in critically ill patients [14, 15]. The PINI score has been measured in several settings, and has been found to be a reliable indicator of both nutritional status and prognosis. The PINI score was determined in this study in order to take into account the effect of protein malnutrition on tHcy concentrations. In our population we evaluated the relationship between nutritional status by means of the PINI score and tHcy concentrations, as tHcy depend greatly on protein intake [30, 31]. During the study no changes in nutritional status were observed in either creatine- or placebo-treated group. We observed no correlation between PINI scores, albumin or prealbumin concentrations, and tHcy concentrations. In conditions of excess folate, nutritional status could be of less importance in determining plasma tHcy concentrations.

Creatine supplementation has been shown to exert ergogenic effects in several populations. In dialysis patients, creatine supplementation was shown to decrease muscle cramps during dialysis [32]. In other conditions associated with low muscular mass and performance [33], creatine was shown to increase muscular performance and quality of life. Further research is necessary to evaluate the potential use and mechanisms of creatine as an ergogenic substance in dialysis patients.

CONCLUSION

The present study demonstrated that inhibition of endogenous methylation demand with dietary creatine supplementation does not further decrease tHcy concentrations in chronic dialysis patients treated with high-dose folic acid, vitamin B6, and B12 supplementation.

ACKNOWLEDGMENTS

This study is supported by a grant from the Fund for Scientific Research-Flanders (FWO-Vlaanderen grant #G.0424.04). Y.E. Taes is Research Assistant of the Fund for Scientific Research-Flanders. Creatine monohydrate was kindly provided by Degussa AG (Trostberg, Germany). The authors wish to thank Ervé Matthys (M.D.), Mario Schurgers (M.D.), Johan Boelaert (M.D.), the dialysis patients, and nursing staff of the Renal Unit, AZ Sint-Jan AV, Brugge, for their cooperation and support.

Reprint requests to Dr. Y. Taes, M.D., Laboratory Clinical Chemistry 2P8, University Hospital Ghent, De Pintelaan 185, 9000 Ghent/Belgium. E-mail: youri.taes@Ugent.be

REFERENCES

1. Selhub J: Homocysteine metabolism. *Annu Rev Nutr* 19:217–246, 1999
2. De Vriese AS, Verbeke F, Schrijvers BF, Lameire NH: Is folate a promising agent in the prevention and treatment of cardiovascular disease in patients with renal failure? *Kidney Int* 61:1199–1209, 2002
3. Bostom AG, Gohh RY, Beaulieu AJ, *et al*: Treatment of hyperhomocysteinemia in renal transplant recipients. A randomized, placebo-controlled trial. *Ann Intern Med* 127:1089–1092, 1997
4. McGregor DO, Dellow WJ, Robson RA, *et al*: Betaine supplementation decreases post-methionine hyperhomocysteinemia in chronic renal failure. *Kidney Int* 61:1040–1046, 2002
5. Refsum H, Ueland PM, Nygård O, Vollset SE: Homocysteine and cardiovascular disease. *Annu Rev Med* 49:31–62, 1998
6. Wyss M, Kaddurah Daouk R: Creatine and creatinine metabolism. *Physiol Rev* 80:1107–213, 2000
7. Walker JB: Creatine: Biosynthesis, regulation and function. *Adv Enzymol Relat Areas Mol Biol* 50:177–242, 1979
8. Mudd SH, Poole JR: Labile methyl balance for normal humans on various dietary regimes. *Metabolism* 24:721–735, 1975
9. Mudd SH, Ebert MH, Scriver CR: Labile methyl group balances in the human: The role of sarcosine. *Metabolism* 29:707–739, 1980
10. Perna AF, Ingrosso D, Lombardi C, *et al*: Homocysteine in uremia. *Am J Kidney Dis* 41:S123–S126, 2003
11. Perna AF, Ingrosso D, Castaldo P, *et al*: Homocysteine and transmethylations in uremia. *Kidney Int* 59:S230–S233, 2001

12. Taes YE, Delanghe JR, De Vriese AS, *et al*: Creatine supplementation decreases homocysteine in an animal model of uremia. *Kidney Int* 64:1331–1337, 2003
13. Delanghe J, De Slypere JP, De Buyzere M, *et al*: Normal reference values for creatine, creatinine, and carnitine are lower in vegetarians. *Clin Chem* 35:1802–1803, 1989
14. Vehe KL, Brown RO, Kuhl DA, *et al*: The prognostic inflammatory and nutritional index in traumatized patients receiving enteral nutrition support. *J Am Coll Nutr* 10:355–363, 1991
15. Nelson KA, Walsh D: The cancer anorexia-cachexia syndrome: A survey of the Prognostic Inflammatory and Nutritional Index (PINI) in advanced disease. *J Pain Symptom Manage* 24:424–428, 2002
16. Senn SJ (editor): *Crossover Trials in Clinical Research*, Chichester, John Wiley, 1993
17. Wyss M, Schulze A: Health implications of creatine: Can oral creatine supplementation protect against neurological and atherosclerotic disease? *Neuroscience* 112:243–260, 2002
18. Stead LM, Au KP, Jacobs RL, *et al*: Methylation demand and homocysteine metabolism: Effects of dietary provision of creatine and guanidinoacetate. *Am J Physiol Endocrinol Metab* 281:E1095–E1100, 2001
19. Steenge GR, Verhoef P, Greenhaff PL: The effect of creatine and resistance training on plasma homocysteine concentrations in healthy volunteers. *Arch Intern Med* 161:1455–1456, 2001
20. Hultman E, Söderlund K, Timmons JA, *et al*: Muscle creatine loading in men. *J Appl Physiol* 81:232–237, 1996
21. Friedman AN, Bostom AG, Selhub J: The kidney and homocysteine metabolism. *J Am Soc Nephrol* 12:2181–2189, 2001
22. House JD, Brosnan ME, Brosnan JT: Renal uptake and excretion of homocysteine in rats with acute hyperhomocysteinemia. *Kidney Int* 54:1601–1607, 1998
23. Suliman M, Anderstam B, Lindholm B, Bergstrom J: Total, free, and protein-bound sulphur amino acids in uraemic patients. *Nephrol Dial Transplant* 12:2332–2338, 1997
24. Van Guldener C, Donker AJM, Jakobs C, *et al*: No net renal extraction of homocysteine in fasting humans. *Kidney Int* 54:166–169, 1998
25. Kreider RB: Species-specific responses to creatine supplementation. *Am J Physiol Regul Integr Comp Physiol* 285:R725–R726, 2003
26. Taes YE, Delanghe JR, Wuyts B, *et al*: Creatine supplementation does not affect kidney function in an animal model with pre-existing renal failure. *Nephrol Dial Transplant* 18:258–264, 2003
27. Poortmans JR, Francaux M: Adverse effects of creatine supplementation. Fact or fiction? *Sports Med* 30:155–170, 2000
28. Kalantar-Zadeh K, Ikizler TA, Block G, *et al*: Malnutrition-inflammation complex syndrome in dialysis patients: Causes and consequences. *Am J Kidney Dis* 42:864–881, 2003
29. Suliman ME, Qureshi AR, Barany P, *et al*: Hyperhomocysteinemia, nutritional status, and cardiovascular disease in hemodialysis patients. *Kidney Int* 57:1727–1735, 2000
30. Suliman ME, Stenvinkel P, Barany P, *et al*: Hyperhomocysteinemia and its relationship to cardiovascular disease in ESRD: Influence of hypoalbuminemia, malnutrition, inflammation, and diabetes mellitus. *Am J Kidney Dis* 41:S89–S95, 2003
31. Suliman ME, Stenvinkel P, Heimburger O, *et al*: Plasma sulfur amino acids in relation to cardiovascular disease, nutritional status, and diabetes mellitus in patients with chronic renal failure at start of dialysis therapy. *Am J Kidney Dis* 40:480–488, 2002
32. Chang CT, Wu CH, Yang JY, *et al*: Creatine monohydrate treatment alleviates muscle cramps associated with haemodialysis. *Nephrol Dial Transplant* 17:1978–1981, 2002
33. Derave W, Eijnde BO, Hespel P: Creatine supplementation in health and disease: Where is the evidence for long-term efficacy? *Moll Cell Biochem* 244:49–55, 2003
