# Supplementary material for: Impact of creatine supplementation on inflammation: evidence from a systematic review and meta-analysis of randomized double-blind placebo trials
Source: Front Immunol. 2026 Feb 19;17:1743603. doi: 10.3389/fimmu.2026.1743603 (PMC12961398; doi:10.3389/fimmu.2026.1743603)
Supplement: Supplementary file 2 [file SupplementaryFile1.zip › SR Creatine inflammatory markers (Kell Doutorado). /Supplementary Files/Final References/Final/Santos et al 2004.pdf]

Available online at www.sciencedirect.com

SCIENCE @ DIRECT®

Life Sciences 75 (2004) 1917–1924

Life Sciences

www.elsevier.com/locate/lifescie

The effect of creatine supplementation upon inflammatory and muscle soreness markers after a 30km race

R.V.T. Santos, R.A. Bassit, E.C. Caperuto, L.F.B.P. Costa Rosa*

Laboratory of Metabolism, Institute of Biomedical Sciences, University of São Paulo, Brazil

Received 8 October 2003; accepted 14 November 2003

Abstract

We have evaluated the effect of a creatine supplementation protocol upon inflammatory and muscle soreness markers: creatine kinase (CK), lactate dehydrogenase (LDH), prostaglandin E_2 (PGE_2) and tumor necrosis factor-alpha (TNF-α) after running 30km. Runners with previously experience in running marathons, with their personal best between 2.5–3h were supplemented for 5 days prior to the 30km race with 4 doses of 5g of creatine and 15g of maltodextrine per day while the control group received the same amount of maltodextrine. Pre-race blood samples were collected immediately before running the 30km, and 24h after the end of the test (the post-race samples). After the test, athletes from the control group presented an increase in plasma CK (4.4-fold), LDH (43%), PGE_2 6.6-fold) and TNF-α (2.34-fold) concentrations, indicating a high level of cell injury and inflammation. Creatine supplementation attenuated the changes observed for CK (by 19%), PGE_2 and TNF-α (by 60.9% and 33.7%, respectively, $p < 0.05$) and abolished the increase in LDH plasma concentration observed after running 30km, The athletes did not present any side effects such as cramping, dehydration or diarrhea, neither during the period of supplementation, nor during the 30km race. All the athletes finished the race in a time equivalent to their personal best ± 5.8%. These results indicate that creatine supplementation reduced cell damage and inflammation after an exhaustive intense race.
© 2004 Elsevier Inc. All rights reserved.

Keywords: Creatine supplementation; Muscle soreness; Inflammation; Long distance running; PGE2

* Corresponding author. Departamento de Histologia e Embriologia, Instituto de Ciênciasncias Biomédicas I, Universidade de São Paulo, Av. Lineu Prestes, 1524, sala 302, 05508-900, Butantan, São Paulo, Brazil. Tel./fax: +55-11-3091-7227.
E-mail address: ggrosa@icb.usp.br (L.F.B.P. Costa Rosa).

0024-3205/$ - see front matter © 2004 Elsevier Inc. All rights reserved.
doi:10.1016/j.lfs.2003.11.036

Introduction

Long distance endurance runners, as athletes in general, aim to train in a way that allows improvement in performance without any harmful effects. Among the leading causes of trauma during and after endurance exercise are eccentric contraction, the impact of the extremities against the ground and the total number of repetitions of the same movement, as in long distance running (Malloch and Taunton, 2000).

Muscle injury leads to cellular damage with membrane disruption and leakage to the extra cellular fluid and plasma of enzymes used as markers of cell death, such as creatine phosphokinase (CK), lactate dehydrogenase (LDH) and carbonic anhydrase III (Moran and Schellmann, 1996; Nuviala et al., 1992; Janssen et al., 1989; Smith and Miles, 2000).

Exercise also induces an increase in prostaglandin (PG) production, as part of an inflammatory response triggered by the micro trauma that occur (Bansil et al., 1985; Bomalaski et al., 1983). There is an enhancement of PGE_2 synthesis, produced by infiltrating macrophages exposed to the inflammatory environment, that is related to type III and IV pain afferent nerve sensitization and, therefore, to pain reported 24–48h after the exercise bout (Thompson et al., 1997).

Within the muscle, the passive elements (connective tissue and muscle fibers) absorb strain. This ability increases by 100% after muscle activation (Malloch and Taunton, 2000). Thus, if muscle activation is in any way compromised, as during fatigue, the ability to absorb strain is reduced, increasing muscle vulnerability to trauma (Malloch and Taunton, 2000). One of the most widely used supplements for ergogenic purposes is creatine (Lawler et al., 2002). Creatine, when supplemented for short-time periods, improves performance during repeated bouts of high intensity exercise (review in Mujika and Padilla, 1997; Kreider, 2003), while there is a lack of an ergogenic effect of creatine supplementation in a variety of endurance exercise protocols (van Loon et al., 2004; Van Schuylenbergh et al., 2003; Izquierdo et al., 2002).

Creatine also causes weight gain through an increase in intracellular water content, leading to an increase in cell volume (Ingwall, 1976; Demant and Rhodes, 1999; Lawler et al., 2002) as well as by stimulating muscle hypertrophy through changes in MRF4 and myogenin expression (Hespel et al., 2001; Willoughby). In fact, both mechanisms could be correlated, as proposed by van Loon and colleagues (2003), since cell swelling stimulates protein synthesis and net protein deposition (Lang et al., 1998). Considering the effects of creatine upon muscle cell volume and protein metabolism, and the impact that this effect could have upon cell injury, we evaluated the effect of creatine supplementation upon the release of markers of inflammation and cell death after a 30km race.

Material and methods

Subjects and protocol

The experimental protocol was approved by the Ethical Committee of the Biomedical Sciences Institute, University of Sao Paulo. After signing an Informed Consent form, thirty four male athletes received, for 5 days, 20g of creatine monohydrate divided in 4 doses of 5g, with 60g of carbohydrate (maltodextrine, n = 18) or the same amount of carbohydrate (n = 16)—placebo group, and ran 30km in a double-blind trial. The athletes were instructed to dilute the powder offered, creatine plus carbohydrate and carbohydrate alone, in the same volume of water. The carbohydrate offered for both groups had the same flavour and color to avoid identification of the samples. The subjects were selected among a group

of non-smoking athletes training for a marathon to be held in one month, who did not consume forbidden drugs or medications and did not have a prior history of ingesting creatine as a supplement. The self-reported personal best in the marathon was in the range of 2.5–3h, and all had the experience of running at least 3 marathons. The athletes, whose mean age was of 25.5 $\pm$ 3.2 years, ranging from 21.4 to 30.1 years, were allowed to drink and eat normally, and received creatine or placebo during the 5 days that preceded the tests. The race started at 7:30 am, under 18 °C and 96% humidity. At the end of the race the temperature decreased to 15 °C and humidity increased to 98%. All the athletes finished the race close to their best personal time for that distance (in a 5.8% range).

Blood sampling

Blood samples were collected (20 ml) from the antecubital vein 15 min before the event and 24h after the race, in sterile tubes containing heparin. Blood samples were centrifuged at 650 $\times$ g, for 15 minutes. Samples were kept at -80 °C and analyzed within one week. From each sample, plasma concentration of creatine kinase, lactate dehydrogenase, prostaglandin E_2 (PGE_2) tumour necrosis factor-α (TNF-α) and creatinine were measured.

Metabolite measurements

Plasma TNF-α and PGE_2 concentrations were assessed using commercially available ELISA-kits (Amershan-Life Science). LDH (EC 1.1.1.27) was spectrophotometrically measured at 37 °C by following the oxidation of NADH in 1ml of a medium containing 0.3M mannitol, 10mM KCl, 5mM MgCl2, 10mM KH2PO4 (pH 7.8) and 0.1% Triton-X-100. The reaction was started by adding pyruvate (Bergmeyer, 1963). CK activity was determined by using a commercial kit (Labtest, São Paulo Brazil) that evaluates the kinetics of CK through changes in the total content of NADH in the essay medium, in a Beckman DU 640 spectrophotometer. Creatinine plasma concentration was also measured with a commercial kit (Labtest São Paulo, Brazil) that evaluates the total amount of creatinine by its conjugation to picric acid. For the LDH assay, the samples were centrifuged at 1174 $\times$ g for 10 minutes to provide a platelet-free plasma sample (Liu et al., 2000).

Statistical analysis

The data obtained were compared using a 2 $\times$ 2 repeated measures ANOVA and the post-hoc test of Tukey and the level of significance of at least $p < 0.05$ was chosen for all statistical comparisons. The data are presented as mean $\pm$ SEM.

All data were analyzed using GraphPad Prism program and graph package (V3.0, GraphPad, Inc., San Diego, Calif., USA). Groups were compared by analysis of variance (ANOVA) and the Turkey post test.

Results

The physical characteristics and the degree of training of the runners are illustrated in Table 1, that shows that the group of athletes was homogeneous in terms of running experience and performance in marathons.

Table 1
Physical characteristics and degree of training (marathon runners, n = 34)

	Mean ± SEM	Range
Age (yrs)	25.5 ± 3.2	21–30
Weight (kg)	62.5 ± 7.9	54.4–77.6
Height (cm)	167.4 ± 5.4	161.4–175.3
Training (Km/wk)	95.6 ± 11.6	80–115
Regular running (yrs)	6.4 ± 1.1	3–10
Personal best (min)	161.4 ± 15.3	150–178

The athletes presented, when consuming only carbohydrate as placebo, an increase of cell death and of the concentration of and inflammation markers, 24h after running 30km. As shown on Table 2, there was an increase of 4.4-fold in plasma CK concentration, of 42.9% in that of LDH and an enhancement of 7.7- and 2.34-fold in the concentrations of the markers of inflammation, PGE_2 and TNFα, respectively, after the 30km race.

Athletes that received creatine supplementation also presented an increase in plasma concentration of CK (5.3-fold), PGE_2 (2.34-fold) and TNFα (1.4-fold) after running the 30km, as shown in Table 2. The increase in LDH concentration was abolished in the supplemented group. When comparing the changes observed after the 30km in the placebo and supplemented group, we noted that the increase observed in LDH, PGE_2 and TNFα plasma concentration was reduced after creatine consumption, from 42.9% to 6% for LDH, from 7.7-fold to 2.34-fold for PGE_2 and from 2.33-fold to 1.44-fold for TNFα. Creatine supplementation also decreased the total amount of LDH (37.9%), PGE_2 (66.4%) and TNFα (33.7%) found in the plasma of athletes after the 30km, compared with the values observed for the placebo group (Table 2).

There was no significant difference between the time that the athletes from both groups, placebo and creatine supplemented, took to finish the 30km.

Discussion

Regular physical exercise is known to be beneficial to health, reducing the risk of a number of pathological disorders and extending the life span of humans and laboratory animals (Radak et al.,

Table 2
Creatine kinase (CK), lactate dehydrogenase (LDH), prostaglandin E_2 (PGE_2), tumour necrosis factor-alpha (TNFα) and creatinine plasma concentrations measured immediately before and 24h after a 30km running, in the plasma obtained from athletes subjected to a placebo (Con, n = 16) or a creatine supplementation protocol (CR, n = 18)

	Con Before	Con After	Cr Before	Cr After
CK (U/l)	48.26 ± 27.28	213.19 ± 113.60*	32.17 ± 16.42	170.95 ± 61.82*
LDH (U/l)	208.9 ± 17.6	298.7 ± 23.6*	198.7 ± 13.5	185.3 ± 21.5#
PGE2 (pg/ml)	42.76 ± 4.95	329.35 ± 17.96*	47.12 ± 8.76	110.42 ± 12.38*#
TNFα (pg/ml)	91.18 ± 7.55	213.76 ± 15.05*	97.77 ± 9.24	141.6 ± 3.32*#
Creatinine (mg/dl)	0.22 ± 0.03	0.34 ± 0.09	0.31 ± 0.05	0.41 ± 0.07

The results are expressed as mean ± SEM of 23 samples.

* $p < 0.05$ for comparison with the values obtained before the exercise bout.

# $p < 0.05$ for comparison with the values obtained for the control group. The observed power was 1,00 for LDH, PGE_2 and TNFα.

2000). However, it has been claimed that some types of exercise, such as high intensity prolonged exercise, are detrimental to health, increasing reactive oxygen species production, and thus leading to the oxidative damage of cells (Radak et al., 2000; Palazzetti et al., 2003), still high intensity exercise may increase musculoskeletal injury, as observed in basic military training (Popovich et al., 2000) and during prolonged exhaustive exercise, when the muscles are exposed to loads to which they are not accustomed to (Hikida et al., 1983; Sorichter et al., 1997). Another cause of muscle injury is the impact against the surface during running. To counteract that effect, the passive elements absorb strain in a very efficient way when the skeletal muscle is fully activated (Malloch and Taunton, 2000). Long distance running, such as marathon, produces muscle fibre necrosis and inflammation, conditions prevalent at 1 and 3 days after the race, accompanied by post-exercise soreness (Hikida et al., 1983).

Considering that muscle cell resistance to injury during running is related to cell volume integrity and its metabolic activation, and that creatine supplementation increases cell volume through an increase in cell water content, glycogen stores and/or myofibrillar content (Ingwall, 1976; Demant and Rhodes, 1999; Lawler et al., 2002; van Loon et al., 2004) as well as having been utilized to attenuate cortical damage after traumatic brain injury in rodents (Rabchevsky et al., 2003); we evaluated the effect of creatine supplementation upon cell injury and inflammatory markers after a 30km run.

The athletes from both groups, placebo and creatine, finished the race close to their best personal time to the 30km. This result is in agreement with the findings of van Loon and colleagues (2003), who failed to show differences in whole-body oxidative capacity in cyclists supplemented with creatine. The design of our study did not allow us to observe any possible effect of increased glycogen content upon performance, as postulated by van Loon and colleagues (2004). In fact, if the athletes were allowed to run until exhaustion, an increase in muscle glycogen content would interfere in performance. During the period of the study the athletes did not report any side effects, such as cramp or intestinal dysfunction.

As expected, running 30km provoked an increase in plasma CK, LDH, PGE_2 and TNF-α concentrations, indicating an increase in cell injury associated with an inflammatory response (Noakes, 1987; Clarcson and Hubal, 2002). In fact, Suman and colleagues (2003) demonstrated that during the early events of the muscle injury/repair process there is an up regulation of genes associated with inflammation, oxidative stress and cell proliferation, which is associated with an increased consumption of non steroidal anti-inflammatory drugs by athletes to avoid muscle pain after long distance running (Baldwin, 2003). In our study we choose to collect blood sample 24 hours after the end of the 30km , considering that pain reports occur 24–48h after exercise, as well as the peak of CK in plasma (Thompson et al., 1997; Noakes, 1987). When searching the literature, however, we found reports about a plasma peak of LDH 8h after exercise, and 48h after a heart stroke (Noakes, 1987; Kaman et al., 1977). Therefore, considering that we found increased plasma concentration of LDH 24h after exercise, it seems quite important to better evaluate the effect of creatine supplementation upon the time profile of plasma enzymes concentration after long distance running.

The creatine loading protocol abolished the increase in LDH plasma concentration and reduced the changes observed for PGE_2 and TNF-α. There was, however, no effect upon CK plasma concentration. In fact, CK plasma concentration is known to vary strongly among individual athletes, and also in relation to gender, racial differences, training status, as well as it does not correlate precisely with changes in other markers such as skeletal troponin I, a regulatory protein of the thin filament (Janssen et al., 1989; Sorichter et al., 1997; Lev et al., 1999). All these could be contributing to the lack of decrease in CK concentration after creatine supplementation.

The reduction in LDH plasma concentration is an important indicative of reduced cell death, since the LDH assay has proved to be one of the best methods for determining the relative amount of cellular death/lysis (Moran and Schellmann, 1996). LDH reduction after exercise clearly indicates decreased skeletal cell injury. Another aspect related to the muscle injury/repair process is the presence of an inflammatory response a few hours after the last exercise bout (Hikida et al., 1983; Komulainen and Vihko, 1994; Baldwin, 2003), accompanied by muscle soreness, which seems to have a profound detrimental effect on motor function (Svensson and Arendt-Nielsen, 1995). In fact, prostaglandin E_2, synthesized by macrophages upon exposure to the inflammatory environment, has been implicated in the sensation of pain following exertion (Thompson et al., 1997). Interestingly, creatine supplementation significantly reduced the increase in plasma PGE_2 after the exercise bout. Another marker of inflammation, involved in muscle soreness, which is altered after exercise is TNF-α (Schafers et al., 2003; Gielen et al., 2003). Our results showed a reduction in the increase of plasma TNF-α levels after the 30km race in the supplemented group. A reduction in TNF-α could be beneficial for reducing muscle soreness (Schafers et al., 2003), as well as for minimizing muscle proteolysis, since TNF-α is associated with an increase in both ubiquitin conjugates and ubiquitin mRNA expression in the skeletal muscle (Argilés and López-Soriano, 1996).

Taken together these observations show that creatine supplementation before running 30km reduces the effort-induced increment observed in markers of cell death, muscle soreness and proteolysis, what suggests a positive effect of the supplementation strategy in maintaining muscle integrity after intense prolonged exercise.

Acknowledgements

We are very thankful to the athletes who made this study possible and who endured the inconvenience of this investigation, as well as to Patrícia Rogeri for blood sampling. Santos, R.V.T and Caperuto, E.C. are recipients of FAPESP scholarships.

References

Argilés, J.M., López-Soriano, F.J., 1996. The ubiquitin-dependent proteolytic pathway in skeletal muscle: its role in pathological states. TiPS 17, 223–226.

Baldwin, L.A., 2003. Use of nonsteroidal anti-inflammatory drugs following exercise-induced muscle injury. Sports Medicine 33, 177–185.

Bansil, C.K., Wilson, G.D., Stone, M.H., 1985. Role of prostaglandins E and F2a in exercise-induced delayed muscle soreness. Medicine Science in Sports and Exercise 17, 276–280.

Bergmeyer, H.U., 1963. Methods of enzymatic analysis, 1–1063.

Bomalaski, J.S., Williamson, P.K., Lurier, R.B., 1983. Prostaglandins and the inflammatory response. Clin. Lab. Med. 3, 695–710.

Clarcson, P.M., Hubal, M.J., 2002. Exercise-induced muscle damage in humans. American Journal of Physical and Medical Rehabilitation 81, S52–S69.

Demant, T.W., Rhodes, E.C., 1999. Effects of creatine supplementation on exercise performance. Sports Medicine 28, 49–60.

Gielen, S., Adams, V., Mobius-Winkler, S., Linke, A., Erbs, S., Yu, J., Kempf, W., Schubert, A., Schuler, G., Hambrecht, R., 2003. Anti-inflammatory effects of exercise training in the skeletal muscle of patients with chronic heart failure. Journal of the American College of Cardiology 42, 861–868.

Hespel, P., Op't Eijnde, B., Van Leemputte, M., Urso, B., Greenhaff, P.L., Labarque, V., Dymarkowski, S., Van Hecke, P., Richter, E.A., 2001. Oral creatine supplementation facilitates the rehabilitation of disuse atrophy and alters the expression of muscle myogenic factors in humans. Journal of Physiology 536, 625–633.
Hikida, R.S., Staron, R.S., Hagerman, F.C., Sherman, W.M., Costill, D.L., 1983. Muscle fiber necrosis associated with human marathon runners. Journal of Neurological Science 59, 185–203.
Ingwall, J.S., 1976. Creatine and control of muscle-specific protein synthesis in cardiac and skeletal muscle. Circulation Research 38, 1115–1123.
Izquierdo, M., Ibanez, J., Gonzalez-Badillo, J.J., Gorostiaga, E.M., 2002. Effects of creatine supplementation on muscle power, endurance, and sprint performance. Med. Sci. Sports Exerc. 34, 332–343.
Janssen, G.M.E., Kuipers, H., Willems, G.M., Does, R.J.M.M., Janssen, M.P.E., Geurten, P., 1989. Plasma activity of muscle enzymes: quantification of skeletal muscle damage and relationship with metabolic variables. International Journal of Sports Medicine 10, S160–S168.
Kaman, R.L., Goheen, B., Patton, R., Raven, P., 1977. The effect of near maximum exercise of serum enzymes: the exercise profile versus the cardiac profile. Clin. Chim. Acta 81, 145–152.
Komulainen, J., Vihko, V., 1994. Exercise-induced necrotic muscle damage and enzyme release in the four days following prolonged submaximal running in rats. Pflugers Archives 428, 346–351.
Kreider, R.B., 2003. Effects of creatine supplementation on performance and training adaptations. Mol. Cell. Biochem. 244, 89–94.
Lang, F., Busch, G.L., Ritter, M., Volkl, H., Waldegger, S., Gulbins, E., Haussinger, D., 1998. Functional significance of cell volume regulatory mechanisms. Physiol. Rev. 78, 247–306.
Lawler, J.M., Barnes, W.S., Wu, G., Song, W., Demaree, S., 2002. Direct antioxidant properties of creatine. Biochemistry and Biophysics Research Communications 290, 47–52.
Lev, E.I., Tur-Kaspa, I., Askhenazy, I., Reiner, A., Faraggi, D., Shemer, J., Argov, Z., 1999. Distribution of serum creatine kinase activity in young healthy persons. Clin. and Chim. Acta 279, 107–115.
Liu, Z.-J., Wang, W., He, C.-S., 2000. Comparison of serum and plasma lactate dehydrogenase in postburn patients. Burns 26, 46–48.
Malloch, A.J., Taunton, J.E., 2000. Overuse syndrome. In: Shepard, R.J., Astrand, P.-O. (Eds.), Endurance in Sport, pp. 766–799.
Moran, J.H., Schellmann, R.G., 1996. A rapid β-NADH-linked fluorescence assay for lactate dehydrogenase in cellular death. Journal of Pharmacology and Toxicology Methods 36, 41–44.
Mujika, I., Padilla, S., 1997. Creatine supplementation as an ergogenic acid for sports performance in highly trained athletes: a critical review. Int. J. Sports Med. 18, 491–496.
Noakes, T.D., 1987. Effect of exercise on serum enzyme activities in humans. Sports Medicine 4, 245–267.
Nuviala, R.J., Roda, L., Lapieza, M.G., Boned, B., Giner, A., 1992. Serum enzymes activities at rest and after a marathon race. Journal of Sports Medicine and Physical Fitness 32, 180–186.
Palazzetti, S., Richard, M.J., Favier, A., Margaritis, I., 2003. Overload training increases exercise-induced oxidative stress and damage. Canadian Journal of Applied Physiology 28, 588–604.
Popovich, R.M., Gardner, J.W., Potter, R., Knapik, J.J., Jones, B.H., 2000. Effect of rest from running on overuse injuries in army basic training. American Journal of Preventive Medicine 18, 147–155.
Rabchevsky, A.G., Sullivan, P.G., Fugaccia, I., Scheff, S.W., 2003. Creatine diet supplement for spinal cord injury: influences on functional recovery and tissue sparing in rats. Journal of Neurotrauma 20, 659–669.
Radak, Z., Pucsuk, J., Boros, S., Josfai, L., Taylor, A.W., 2000. Changes in urine 8-hydroxydeoxyguanosine levels of super-marathon runners during a four day race period. Life Sciences 66, 1763–1767.
Schafers, M., Sorkin, L.S., Sommer, C., 2003. Intramuscular injection of tumor necrosis factor-alpha induces muscle hyperalgesia in rats. Pain 104, 579–588.
Smith, L.L., Miles, M.P., 2000. Exercise Induced Muscle Injury and Inflammation. In: Garret, W.E., Kirkendall, D.T. (Eds.), Exercise and Sport Science, 401–413.
Sorichter, S., Mair, J., Koller, A., Muller, E., Kremser, C., Judmaier, W., Haid, C., Rama, D., Calzolari, C., Puschendorf, B., 1997. Skeletal muscle troponin I release and magnetic resonance imaging signal intensity changes after eccentric exercise-induced skeletal muscle injury. Clin. Chim. Acta 262, 139–146.
Suman, M., McKinstry, M., Warren, G.L., Hulderman, T., Mishra, D., Brumbaugh, K., Luster, M.I., Simeonova, P.P., 2003. Inflammatory mediators and skeletal muscle injury: a DNA microarray analysis. Journal of Interferon and Cytokine Research 23, 237–245.

Svensson, P., Arendt-Nielsen, L., 1995. Induction and assessment of experimental muscle pain. Journal of Electromyography and Kinesiology 5, 131–140.

Thompson, H.S., Hyatt, J.-P., De Souza, M.J., Clarkson, P.M., 1997. The effects of oral contraceptives on delayed onset muscle soreness following exercise. Contraception 56, 59–65.

Van Loon, L.J.C., Murphy, R., Oostelaar, A.M., Cameron-Smith, D., Hargreaves, M., Wagenmakers, A.J.M., Snow, R., 2004. Creatine supplementation increases glycogen storage but not GLUT-4 expression in human skeletal muscle. Clin. Sci. 106, 99–106.

Van Loon, L.J.C., Oostelaar, A.M., Hartgens, F., Hesselink, M.K.C., Snows, R.J., Wagenmakers, A.J.M., 2003. Effects of creatine loading and prolonged creatine supplementation on body composition, fuel selection, sprint and endurance performance in humans. Clin. Sci. 104, 153–162.

Van Schuylenbergh, R., Van Leemputte, M., Hespel, P., 2003. Effects of oral creatine-pyruvate supplementation in cycling performance. Int. J. Sports Med. 24, 144–150.
