# Supplementary material for: Impact of creatine supplementation on inflammation: evidence from a systematic review and meta-analysis of randomized double-blind placebo trials
Source: Front Immunol. 2026 Feb 19;17:1743603. doi: 10.3389/fimmu.2026.1743603 (PMC12961398; doi:10.3389/fimmu.2026.1743603)
Supplement: Supplementary file 2 [file SupplementaryFile1.zip › SR Creatine inflammatory markers (Kell Doutorado). /Supplementary Files/Final References/Final/Deldicque et al 2008.pdf]

J Appl Physiol 104: 371–378, 2008.
First published November 29, 2007; doi:10.1152/japplphysiol.00873.2007.

Effects of resistance exercise with and without creatine supplementation on gene expression and cell signaling in human skeletal muscle

Louise Deldicque,[1] Philip Atherton,[2] Rekha Patel,[2] Daniel Theisen,[1] Henri Nielens,[1] Michael J. Rennie,[2] and Marc Francaux[1]

[1]*Department of Physical Education and Rehabilitation, Université catholique de Louvain, Louvain-la-Neuve, Belgium; and* [2]*University of Nottingham, School of Graduate Entry Medicine and Health, City Hospital, Derby, United Kingdom*

Submitted 14 August 2007; accepted in final form 23 November 2007

Deldicque L, Atherton P, Patel R, Theisen D, Nielens H, Rennie MJ, Francaux M. Effects of resistance exercise with and without creatine supplementation on gene expression and cell signaling in human skeletal muscle. *J Appl Physiol* 104: 371–378, 2008. First published November 29, 2007; doi:10.1152/japplphysiol.00873.2007.—To test the hypothesis that creatine supplementation would enhance the anabolic responses of muscle cell signaling and gene expression to exercise, we studied nine subjects who received either creatine or a placebo (maltodextrin) for 5 days in a double-blind fashion before undergoing muscle biopsies: at rest, immediately after exercise (10 × 10 repetitions of one-leg extension at 80% 1 repetition maximum), and 24 and 72 h later (all in the morning after fasting overnight). Creatine supplementation decreased the phosphorylation state of protein kinase B (PKB) on Thr308 at rest by 60% ($P < 0.05$) and that of eukaryotic initiation factor 4E-binding protein on Thr37/46 (4E-BP1) by 30% 24 h postexercise ($P < 0.05$). Creatine increased mRNA for collagen 1 (α_1), glucose transporter-4 (GLUT-4), and myosin heavy chain I at rest by 250%, 45%, and 80%, respectively, and myosin heavy chain IIA (MHCIIA) mRNA immediately after exercise by 70% (all $P < 0.05$). Immediately after exercise, and independent of creatine, mRNA for muscle atrophy F-box (MAFbx), MHCIIA, peroxisome proliferator-activated receptor γ coactivator-1α, and interleukin-6 were upregulated (60–350%; $P < 0.05$); the phosphorylation state of p38 both in the sarcoplasm and nucleus were increased (12- and 25-fold, respectively; both $P < 0.05$). Concurrently, the phosphorylation states of PKB (Thr308) and 4E-BP1 (Thr37/46) were decreased by 50% and 75%, respectively ($P < 0.05$). Twenty-four hours postexercise, MAFbx, myostatin, and GLUT-4 mRNA expression decreased below preexercise values (−35 to −50%; $P < 0.05$); calpain 1 mRNA increased 70% 72 h postexercise ($P < 0.05$) and at no other time. In conclusion, 5 days of creatine supplementation do not enhance anabolic signaling but increase the expression of certain targeted genes.

mitogen-activated protein kinase; protein kinase B; protein synthesis

CREATINE SUPPLEMENTATION HAS BEEN used in various contexts, not only among athletes as an ergogenic aid for improving muscle power output during high-intensity exercise (16) but also as a potential therapeutic agent for patients suffering from general muscle wasting and myopathies (24, 37, 40). Creatine supplementation leads to an increase in muscle fiber area when associated with resistance exercise training (39) or in the case of muscle regeneration after atrophy (18). More recently, creatine supplementation has been shown to amplify the increase in satellite cell number and myonuclei concentration in human skeletal muscle fibers during 4–16 wk of resistance training. These creatine-induced adaptations were associated with an enhanced muscle fiber growth in response to strength training (32).

Evidence accumulated over the past 15 years suggests that the primary mechanism by which creatine exerts its anabolic effect in healthy subjects who are weight training is by allowing them to work at a higher proportion of their maximal voluntary contraction force and thus increase the training stimulus (16). However, this mechanism may not explain the higher force production observed in patients suffering from myopathy (24, 37). Therefore, the search for alternative mechanisms for the beneficial effects of creatine has been under scrutiny. Several studies suggest that creatine could be more effective in muscle submitted to degeneration/regeneration process induced by exercise (32), immobilization (18), or disease (24, 37). Therefore, we also hypothesized that creatine would slow down the degeneration phase and accelerate the regeneration phase after high-intensity exercise, which usually occur 1 and 3 days postexercise, respectively (15).

In two previous studies, we were unable to detect any difference in the myofibrillar or sarcoplasmic protein synthetic rates or the breakdown rate of human muscle after creatine supplementation whether at rest (26) or postexercise (25). This lack of effect of creatine after exercise might have been explained by the timing of the measurements made. For example, we measured the protein turnover rates immediately after exercise following 5 days of supplementation, and it might have been that the creatine-plus-exercise effects were present at a later time point. Indeed, exercise-induced increases in protein synthesis can last for up to 72 h (29). Furthermore, there may be intricate changes in translational signaling attributable to creatine that are undetectable by direct measures of muscle protein synthesis. Indeed, many recent studies in human muscle have implicated changes in such candidate pathways with resistance exercise, including the PKB (protein kinase B)-mTOR and the MAPK (mitogen-activated protein kinase) pathways, key cascades in the regulation of skeletal muscle protein synthesis, and remodelling by resistance exercise (10, 13, 14, 20, 42).

Furthermore, in the past studies, we did not examine the possibility of a "priming" of muscle gene expression that could be important for forthcoming synthetic responses to resistance exercise or indeed other adaptive processes. In support of the notion for the modulation of gene expression by creatine, there has been evidence of increased myosin heavy chain (MHC) and IGF-I and II in human subjects after creatine ingestion (11,

Address for reprint requests and other correspondence: M. Francaux, Place Pierre de Coubertin 1, B-1348 Louvain-la-Neuve, Belgium (e-mail: marc.francaux@uclouvain.be).

The costs of publication of this article were defrayed in part by the payment of page charges. The article must therefore be hereby marked "*advertisement*" in accordance with 18 U.S.C. Section 1734 solely to indicate this fact.

 8750-7587/08 $8.00 Copyright © 2008 the American Physiological Society

Downloaded from journals.physiology.org/journal/jappl (2804:07F0:7502:2C1D:3467:2D37:5744:F794) on June 21, 2025.

43). Furthermore, such gene expression changes, either in the satellite cells or in the myofiber after each exercise session, could be one mechanism by which the induction of satellite cell nuclei into the myofiber occurs after several weeks of resistance training, when coupled with creatine supplementation (32).

The aims of the present study were to test whether 5 days of creatine supplementation induce short-term changes in gene expression and cellular signaling at rest and after a single bout of exercise. We hypothesized that short-term creatine supplementation would increase expression of genes associated with the control of muscle mass, phenotype, and metabolism and augment anabolic signal transduction activity through the MAPK and the PKB pathways.

METHODS

Subjects. Nine healthy young men (21.7 ± 0.55 y, BMI 24 ± 0.9 kg/m^2) who did not partake in any formal resistance exercise regime, were recruited for this double-blind crossover study. All subjects were given an oral and written account of the study before signing a consent form. This study was approved by the Ethics Committee of the Université catholique de Louvain, and the investigation was performed according to the principles outlined in the Declaration of Helsinki.

Experimental protocol. Before the experiment, subjects participated in a pretest to determine one repetition maximum (1-RM) for each leg on a leg-extension apparatus. The exercise consisted of a one-leg knee extension movement from an angle of 90° to 160°. After a warm-up comprised of three sets of 10 repetitions at 5 kg, the load was progressively increased until the subject could not perform more than one single repetition. Subjects were allowed 2 min of rest between each set and reached 1-RM within five to six trials.

Subjects were instructed to refrain from vigorous physical activity 2 days before and during the experimental phase. Food intake on the evening preceding each muscle biopsy was controlled by administering a standardized dinner (22% protein, 48% carbohydrate, and 30% fat). Subjects were randomly divided into two groups: one group (n = 5) received 21 g (3 × 7 g) of oral creatine monohydrate per day for 5 days before the beginning of the experiment and during the 3 days of the experiment, whereas the second group (n = 4) received a placebo (maltodextrin 3 × 7 g/day) during the same period (the protocol is summarized in Fig. 1). During the experimental days, the subjects were asked to ingest creatine during the breakfast after biopsy sampling. After a washout period of 6 wk, the treatments were crossed over for the second trial.

On the first morning of the study, participants reported to the laboratory after a 10-h overnight fast, and a first biopsy was taken at rest in the control leg chosen at random. The procedure involved the administration of local anaesthesia (1% lidocaine) and sample extraction from the mid portion of the vastus lateralis muscle with a 4-mm Bergström biopsy needle. Blood, macroscopically visible fat, and connective tissue were quickly removed, and the sample was immediately frozen in liquid nitrogen and stored at −80°C.

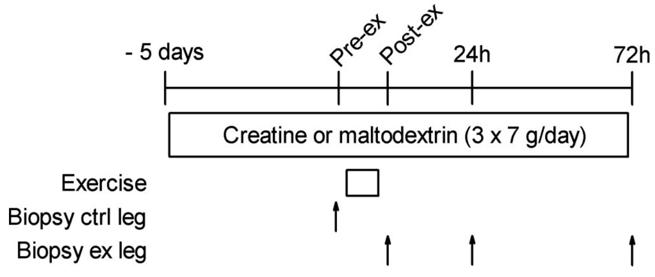

Fig. 1. Experimental protocol.

The exercise was then performed with the other leg after a warm-up of three sets of 10 repetitions at 5 kg. The main exercise session consisted of 10 sets of 10 repetitions at 80% of the 1-RM of the exercising leg, which corresponds to a mean value of positive work of 19,471 ± 1,403.5 J for the placebo trial and 19,902 ± 1,504.1 J for the creatine one. The positive work of each repetition was calculated by multiplying the moved mass by g (9.81 m/s^2) and by the distance (height to which the mass was raised). In this calculation, we neglected the friction due to the pulleys. The 1-RM was not exactly the same for both legs in each subject resulting in different mean work between placebo and creatine trials. These values were not statistically different. All subjects performed the same number of repetitions during both trials. A second biopsy was taken from the exercising leg within 30 s following the completion of the last repetition. A standardized breakfast was given after the exercise session (475 kcal; 7% protein, 74% carbohydrate and 19% fat). Each participant received a standardized dinner in the evening. Additional biopsies were taken 24 and 72 h later from the exercising leg, each after a 10-h overnight fast.

Protein extraction and cell fractionation. About 20–30 mg of frozen muscle were ground in a mortar and homogenized in ice-cold hypotonic buffer [20 mM Hepes, 5 mM sodium fluoride, 1 mM sodium molybdate, 0.1 mM EDTA, 0.5% NP-40, protease inhibitor cocktail (Roche Applied Science)] for 5 min on ice. The homogenates were then centrifuged for 30 s at 10,000 g. The supernatant, containing the sarcoplasmic proteins, was stored at −80°C. The pellet was resuspended in a buffer containing 20 mM Hepes, 5 mM sodium fluoride, 1 mM sodium molybdate, 0.1 mM EDTA, 20% glycerol, a protease inhibitor cocktail, and the same volume of a saline buffer containing 20 mM Hepes, 5 mM sodium fluoride, 1 mM sodium molybdate, 0.1 mM EDTA, 20% glycerol, 0.8 M NaCl, and a protease inhibitor cocktail. The solution was then homogenized on a rotary mixer for 30 min at 4°C and centrifuged for 10 min at 10,000 g. The supernatant, containing the nuclear proteins, was stored at −80°C. Sarcoplasmic and nuclear protein concentrations were determined using a protein assay kit (Bio-Rad Laboratories) with BSA as a standard. Fraction purity was verified and confirmed by immunoblotting for nuclear histone 1 (anti-histone 1, 1:1,000, Santa Cruz).

SDS/PAGE and immunoblotting. Cell lysates (70 μg for sarcoplasmic proteins and 30 μg for nuclear proteins) were combined with Laemmli sample buffer and separated by SDS/PAGE. After electrophoretic separation at 40 mA, the proteins were transferred to a PVDF membrane at 80 V for 4 h for a Western blot analysis. Membranes were then incubated in a 5% Blotto solution. Subsequently, membranes were incubated with the following antibodies (1:500) overnight at 4°C: phospho-PKB Ser 473 (Cell Signaling), phospho-PKB Thr 308 (Cell Signaling), total PKB (Cell Signaling), phospho-p70^{s6k} Thr 389 (Santa Cruz), total p70^{s6k} (Santa Cruz), phospho-p38 Thr 180/Tyr 182 (Cell Signaling), total p38 (Cell Signaling), phospho-ERK1/2 Thr 202/Tyr 204 (Cell Signaling), total ERK (Cell Signaling), phospho-4E-BP1 Thr 37/46 (Cell Signaling), total 4E-BP1 (Cell Signaling), and myocyte enhancer factor-2 (MEF-2) (Santa Cruz). Antibodies from Cell Signaling were diluted in TBS-Tween containing 1% BSA, and antibodies from Santa Cruz were diluted in a 5% Blotto solution. PKB phosphorylated on Thr 308 and Ser 473 was analyzed because the phosphorylation of both sites is required to achieve a high level of kinase activity (3).

Membranes were washed in TBS-Tween and incubated for 1 h at room temperature in a secondary antibody conjugated to horseradish peroxidase (1:10,000, Cell Signaling). After an additional three washes, chemiluminescence detection was carried out using an enhanced chemiluminescent Western blotting kit (ECL Plus, Amersham Biosciences) and hyperfilms (Hyperfilm ECL, Amersham Biosciences). Then, the membranes were stripped and reprobed with a total antibody to verify the relative amount of the analyzed proteins

Downloaded from journals.physiology.org/journal/jappl (2804:07F0:7502:2C1D:3467:2D37:5744:F794) on June 21, 2025.

through the whole experiment. The films were scanned with an image scanner using the Labscan software and quantified with the Image Master 1D Image Analysis Software (Amersham Biosciences). The results represent the phosphorylated form of the protein. A value of 1 was arbitrarily assigned to the control conditions (placebo preexercise) to which the postexercise and the creatine values were reported. The choice of this baseline enables depiction of the effects of creatine supplementation in addition to the effects of acute exercise.

RNA extraction and quantitative real-time PCR. Frozen tissue samples (~30 mg) were homogenized in TRIZOL using a Polytron. Total RNA was extracted according to the instructions provided by the manufacturer. RNA was quantified by spectrophotometry (260 nm), and its concentration was adjusted to 1 μg/μl using RNase-free water. A RNA agarose gel was run to verify the integrity of the RNA. Reverse transcription (RT) was performed using the iScript synthesis kit (Bio-Rad) on a iQ5 real-time PCR detection system (Bio-Rad), with 1 μg of total RNA in a reaction volume of 20 μl (4 μl iScript reaction mix ×5, 1 μl iScript reverse transcriptase, 1 μl RNA template, 14 μl RNase-free water). The final RT product was adjusted to 140 μl using RNase free water. Real-time RT-PCR primers were designed (Table 1) for human calpain 1, collagen 1(α_1), C2 subunit of proteasome, GLUT-4, IL-6, muscle atrophy F-box (MAFbx), myosin heavy chain (MHC) I, MHC IIA, MyoD, myostatin, proliferating cell nuclear antigen (PCNA), peroxisome proliferator-activated receptor gamma coactivator (PGC)-1α, and β_2-microglobulin. The latter was used as the "house-keeping" gene, because preliminary experiments as well as a previous study (30) had revealed that it was not affected by either exercise or creatine supplementation. Sybr Green real-time RT-PCR analyses were carried out on the iQ5 real-time PCR detection system (Bio-Rad) using the following cycle conditions: 10 min at 95°C, followed by 40 cycles of 1 min at 60°C, and 15 s at 95°C. For each gene, real-time RT-PCR was conducted in duplicate with 25-μl reaction volume containing 12.5 μl of Platinium Sybr Green qPCR SuperMix UDG (Invitrogen), 0.75 μl of each primer (10 pmol/μl), 9 μl of RNAse-free water, and 2 μl of 1:5 diluted cDNA. A melt analysis was run to verify the amplified DNA product. A value of 1 was arbitrarily assigned to the control conditions (placebo preexercise) to which the postexercise and the creatine values were reported.

Muscle creatine concentration. Muscle creatine concentration was measured on the preexercise biopsy as previously described (11). Briefly, a fraction of the muscle biopsy (~15 mg) was used for spectrophotometric determination of total creatine (i.e., creatine phosphate + free creatine). Ground muscle was extracted in 0.25 M $HClO_4$ and neutralized with 1 M KOH. A fraction of the supernatant was used to determine free creatine, the remaining serving to determine total creatine content. To hydrolyze creatine phosphate, 2 M HCl was added to the supernatant, which was then heated for 15 min at 60°C. The reaction was stopped on ice, and the supernatant was neutralized with 2 M NaOH. Total creatine was immediately determined enzymatically using a spectrophotometric method described by Guder et al. (17) (creatinine PAP, Boerhinger Mannheim, Germany, from which the creatininase was omitted). Phosphorylcreatine concentration was calculated by subtracting free creatine from total creatine concentrations.

Statistical analysis. The difference in muscle creatine content between placebo and creatine conditions was tested for significance using a paired *t*-test. Treatment by time interactions were evaluated using a two-way ANOVA for repeated measures. When appropriate, Student-Newman-Keuls post hoc tests were applied. The significance threshold was set to $P < 0.05$. The results are presented as means ± SE.

RESULTS

Muscle creatine concentration. After 5 days of supplementation muscle total creatine concentration increased by ~20% ($P < 0.01$) (Table 2).

Transcriptional regulation by exercise and creatine. Immediately after exercise, increases in PCNA mRNA (+150%; $P < 0.05$), MAFbx (+70%; $P < 0.05$), MHC IIA (+60%; $P < 0.05$), PGC-1α (+80%; $P < 0.05$), and IL-6 (+350%; $P < 0.05$) were observed (Fig. 2). The expression of MHC IIA mRNA, PGC-1α, and IL-6 all returned to preexercise values by 24 or 72 h postexercise ($P < 0.05$). At 24 h postexercise, PCNA mRNA nearly returned to preexercise values before increasing again at 72 h postexercise (+130%; $P < 0.05$). MAFbx mRNA decreased below preexercise values at 24 h postexercise (−50%; $P < 0.05$) but had returned to basal values 2 days later. As for MAFbx, mRNA for myostatin (−35%; $P < 0.01$) and GLUT-4 (−45%; $P < 0.01$) was also decreased at 24 h postexercise compared with preexercise values but had returned to baseline by 72 h postexercise. Calpain 1 mRNA was increased 72 h postexercise compared with preexercise (+70%), postexercise (+20%), and 24 h postexercise (+40%) ($P < 0.05$).

Creatine increased the expression of collagen 1(α_1) (+250%; $P < 0.05$), GLUT-4 (+45%; $P < 0.05$), and MHC I mRNA (+80%; $P < 0.05$) at rest and the expression of MHC IIA mRNA immediately after exercise (+70%; $P < 0.05$).

Activation of the MAPK pathway by exercise. Immediately after exercise, the phosphorylation state of p38 was increased

Table 1. *Sequences of primers used for mRNA quantification by real-time RT-PCR*

	Forward	Reverse	Accession Number
Calpain 1	GCC AAG CAG GTG AAC TAC	TGA AGT CTC GGA ATG ACA TC	NM_005186
Collagen 1(α_1)	GTG CTA AAG GTG CCA ATG GT	CTC CTC GCT TTC CTT CCT CT	NM_000088
C2	CAT TGA AAA GGG CGC AAT C	GCC ATA TCG TTG TGT TGG TA	NM_148976
GLUT-4	CAG TAT GTT GCG GAG GCT AT	CCT CGA GTT TCA GGT ACT CTT	NM_001042
IL-6	TGG ATT CAA TGA GGA GAC TTG	GAT TCT TTG CCT TTT TCT GC	BT_019749
MAFbx	CGA CCT CAG CAG TTA CTG CAA C	TTT GCT ATC AGC TCC AAC AGC C	NM_058229
MHC I	ACA AGC TGC AGC TAA AGG TC	TCA AGA TGT GGC AAA GCT AC	NM_000257
MHC IIA	AGG CTT CAA GAT TTG GTA GA	TTC CTT TGC AAC AGG GTA GA	NM_017534
MyoD	CCG CCT GAG CAA AGT AAA TG	GCC CTC GAT ATA GCG GAT G	NM_002478
Myostatin	CTA CAA CGG AAA CAA TCA TTA CCA	GTT TCA GAG ATC GGA TTC CAG TAT	NM_005259
PCNA	AGG AGG AAG CTG TTA CCA TAG AG	AAG TGT CCC ATA TCC GCA AT	NM_002592
PGC-1α	GAT GAT GGA GAC AGC TAT GGT	GAG TCA TAC TTG CTC TTG GTG	NM_013261
β_2-Microglobulin	ATG AGT ATG CCT GCC GTG TGA	GGC ATC TTC AAA CCT CCA TG	NM_004048

Primer sequences: C2, C2 subunit of proteasome; GLUT-4, glucose transporter 4; IL-6, interleukin-6; MAFbx, muscle atrophy F-box; MHC I, myosin heavy chain type I; MHC IIA, myosin heavy chain type II A; PCNA, proliferating cell nuclear antigen; PGC-1α, peroxisome proliferator-activated receptor gamma coactivator-1α.

Downloaded from journals.physiology.org/journal/jappl (2804:07F0:7502:2C1D:3467:2D37:5744:F794) on June 21, 2025.

Table 2. *Creatine, phosphorylcreatine, and total creatine concentrations in muscle*

	Placebo (n = 9)	Creatine (n = 9)	Placebo *Trial 1* (n = 5)	Creatine *Trial 2* (n = 5)	Creatine *Trial 1* (n = 4)	Placebo *Trial 2* (n = 4)
Cr	9.6±0.64	12.6±0.67*	9.2±0.82	12.9±1.10*	12.1±0.78	10.0±1.11*
PCr	16.3±1.17	17.6±0.63	15.7±1.37	17.3±0.79	18.1±1.12	16.9±2.20
Total Cr	25.8±1.18	30.2±0.71†	24.9±1.18	30.2±0.78*	30.2±1.40	26.9±1.89*

Values are means ± SE (mmol/kg wet wt). Free creatine (Cr), phosphorylcreatine (PCr), and total Cr levels after Cr supplementation for 5 days (3 × 7 g/day). Results are presented for the nine subjects, for the subgroup of subjects (n = 5) receiving placebo during the first trial and Cr during the second trial and for the subgroup of subjects (n = 4) receiving creatine during the first trial and placebo during the second trial. Significnat difference: *$P < 0.05$; †$P < 0.01$.

more than 10-fold in the sarcoplasm ($P < 0.05$) and more than 20-fold in the nucleus ($P < 0.01$) (Figs. 3 and 5). It returned to preexercise values 24 and 72 h postexercise in both fractions. The phosphorylation state of ERK1/2 in the nucleus tended to be increased by exercise but did not reach the statistical threshold ($P = 0.065$). Immediately postexercise, the expression of MEF-2 was doubled in the nucleus ($P < 0.05$). Creatine had no effect on the phosphorylation state of p38 and ERK1/2 or on the expression of MEF-2 in the nucleus.

Alteration of the PKB pathway by exercise. Immediately after exercise, the phosphorylation state of PKB on Thr 308 ($P < 0.05$) and 4E-BP1 on Thr 37/46 ($P < 0.01$) decreased by 50 and 75%, respectively, and returned to preexercise values at 24 and 72 h postexercise (Figs. 4 and 5). The same trend to inhibition was observed immediately postexercise on both PKB on Ser 473 and on p70^{s6k} on Thr 389, but the statistical significance was not reached. Twenty-four hours postexercise, two of the nine subjects showed a markedly elevated phos-

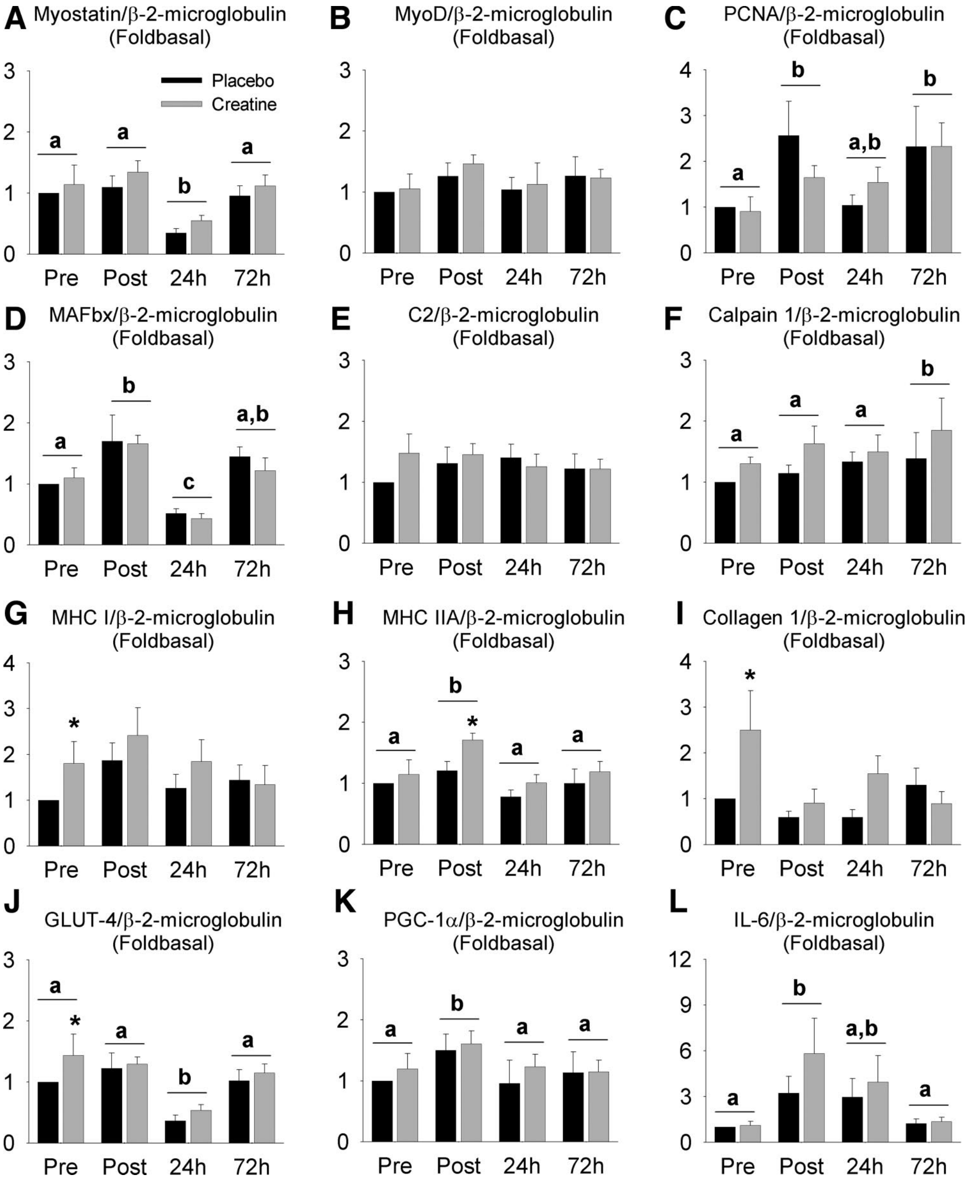

Fig. 2. Effect of creatine supplementation on the mRNA for myostatin (*A*), MyoD (*B*), proliferating cell nuclear antigen (PCNA; *C*), muscle atrophy F-box (MAFbx; *D*), C2 subunit of proteasome (*E*), calpain 1 (*F*), myosin heavy chain (MHC) I (*G*), MHC IIA (*H*), collagen 1 (α_1) (*I*), GLUT-4 (*J*), peroxisome proliferator-activated receptor gamma coactivator-1α (PGC-1α; *K*), and IL-6 (*L*). Values are means ± SE (n = 9) and relative to the placebo condition before exercise. *Significant difference ($P < 0.05$) of creatine vs. placebo at the same time point. Two different letters above the histograms indicate a significant difference ($P < 0.05$) between the different time points.

Downloaded from journals.physiology.org/journal/jappl (2804:07F0:7502:2C1D:3467:2D37:5744:F794) on June 21, 2025.

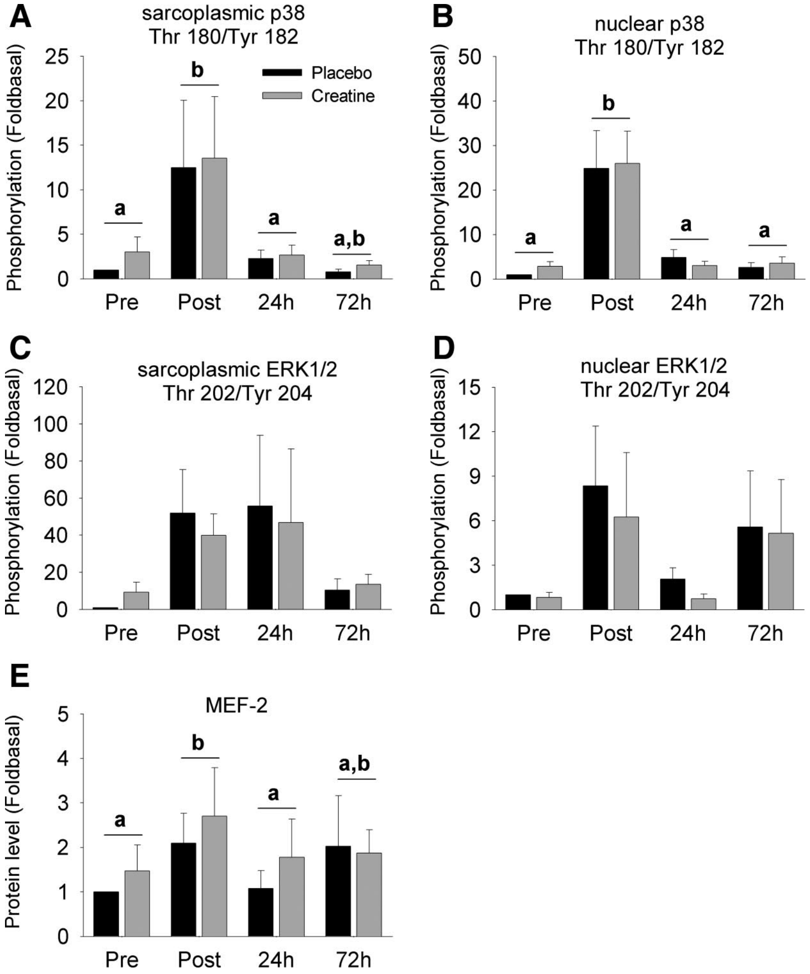

Fig. 3. Effect of creatine supplementation on the phosphorylation state of sarcoplasmic p38 on Thr 180/Tyr 182 (*A*), nuclear p38 on Thr 180/Tyr 182 (*B*), sarcoplasmic ERK1/2 on Thr 202/Tyr 204 (*C*), nuclear ERK1/2 on Thr 202/Tyr 204 (*D*), and expression of MEF-2 in the nucleus (*E*). Results are means ± SE (n = 9) and relative to the placebo condition before exercise. Two different letters above the histograms indicate a significant difference ($P < 0.05$) between the different time points.

phorylation state of p70^{s6k} on Thr 389, whereas it remained unchanged in the other subjects. Creatine decreased the phosphorylation state of PKB on Thr 308 at rest by 60% ($P < 0.05$) and the phosphorylation state of 4E-BP1 on Thr 37/46 24 h postexercise by 30% ($P < 0.05$).

DISCUSSION

We designed the studies described above to determine principally whether the combination of acute exercise with 5 days of creatine supplementation would cause a greater than normal increase in the expression of genes and in signal transduction likely to be involved in adaptation of muscle size or biochemical properties. Indeed, exercise is known to activate multiple signal transduction pathways and to modulate transcriptional and translational processes.

The only significant gene alterations we observed in response to creatine supplementation were increases in the expression of GLUT-4, collagen 1(α_1), and MHC I mRNA at rest and increased expression of MHC IIA mRNA immediately postexercise. Most of the changes in mRNA levels observed in the present study and elsewhere (11) occurred at rest before the resistance exercise session. This suggests that, contrary to our hypotheses, creatine per se is able to modify the expression of several genes and that exercise training and/or a process of muscle degeneration/regeneration are not an essential prerequisite.

In addition to the modulation of gene expression, creatine supplementation has been shown to augment the increase in satellite cells and myonuclei number induced by several weeks of strength training (32). Therefore, the activation, proliferation, and differentiation of satellite cells are other mechanisms by which creatine might increase muscle mass after several weeks of supplementation combined with training. In the current study, we found that, although exercise did acutely increase a marker of satellite cell proliferation (PCNA, a protein involved in DNA replication maximally expressed in S phase), there was no prolonged effect over the next 24 h but a rebound at 72 h and no additional effect of creatine. MyoD is another key regulator of muscle remodeling associated with cell proliferation. We did not observe any change in MyoD mRNA by creatine nor by exercise, whereas others found a doubling of MyoD expression immediately postexercise (36) or a peak expression at 8–12 h postexercise (45).

Although there is good evidence that the MAPK and the PKB pathways are involved in the regulation of skeletal muscle protein synthesis and remodeling by resistance exercise (4, 10, 31, 41), short-term creatine supplementation does not seem to act via these two cascades to increase muscle mass, contrary to our hypothesis. Creatine supplementation had no additional effect on the phosphorylation of p38 and ERK1/2 or on MEF-2 protein expression, nor did creatine increase signaling through

Downloaded from journals.physiology.org/journal/jappl (2804:07F0:7502:2C1D:3467:2D37:5744:F794) on June 21, 2025.

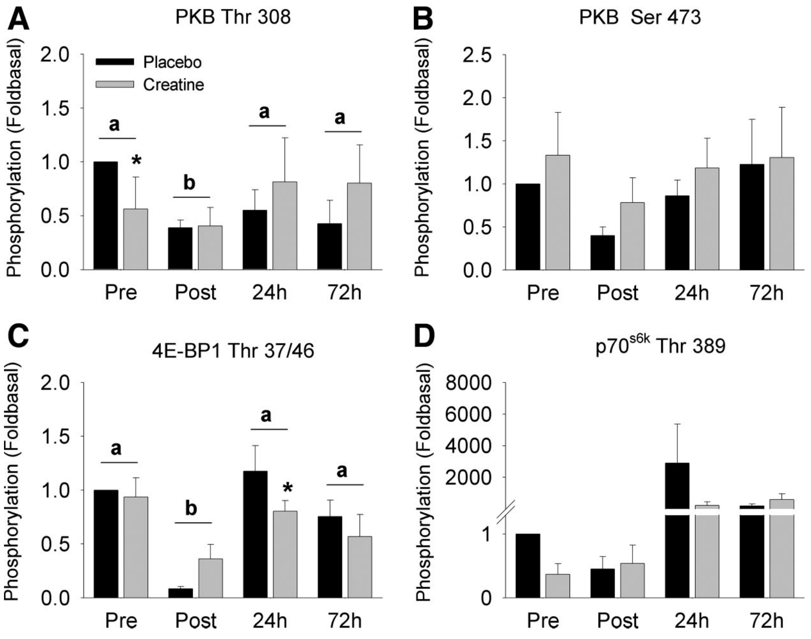

Fig. 4. Effect of creatine supplementation on the phosphorylation state of PKB on Thr 308 (*A*), PKB on Ser 473 (*B*), 4E-BP1 on Thr 37/46 (*C*), and $p70^{s6k}$ on Thr 389 (*D*). Results are means ± SE ($n = 9$) and relative to the placebo condition before exercise. *Significant difference ($P < 0.05$) of creatine vs. placebo at the same time point. Two different letters above the histograms indicate a significant difference ($P < 0.05$) between the different time points.

the PKB pathway. The results on the MAPK and the PKB pathways are in agreement with our previous studies showing no effect on protein synthesis (25, 26).

Taken together, our results indicate that, although the most remarkable effects of creatine are seen after several weeks of supplementation, some of them could be initiated at the transcription level as soon as after 5 days of supplementation. Nevertheless, the extent of these increases is rather small, and we have no evidence to relate them to activation of satellite cells and to muscle mass accumulation observed when creatine is combined with resistance training (32).

This study also produced novel results in the context of resistance exercise. These can be summarized as follows: *1*) an acute rise in the expression of MAFbx mRNA seen immediately at the end of exercise followed by a subsequent fall at 24 h before the return to basal values by 72 h; *2*) a fall in myostatin mRNA at 24 h; *3*) PCNA, a marker of cell proliferation, was activated in a biphasic way, increasing immediately postexercise and after 3 days of recovery; *4*) the increases in both the sarcoplasmic and nuclear complements of p38 and ERK1/2; *5*) the immediate rise after exercise of MEF-2 protein. Therefore, acute resistance exercise induced very rapid, pronounced alterations in gene transcription and cellular signaling, indicative of the early modifications of some specific mRNAs seen at the end of exercise. Additional effects of creatine were much less pronounced than the effects of exercise alone. However, since no control group undertook muscle biopsies without resistance exercise and considering the potential influ-

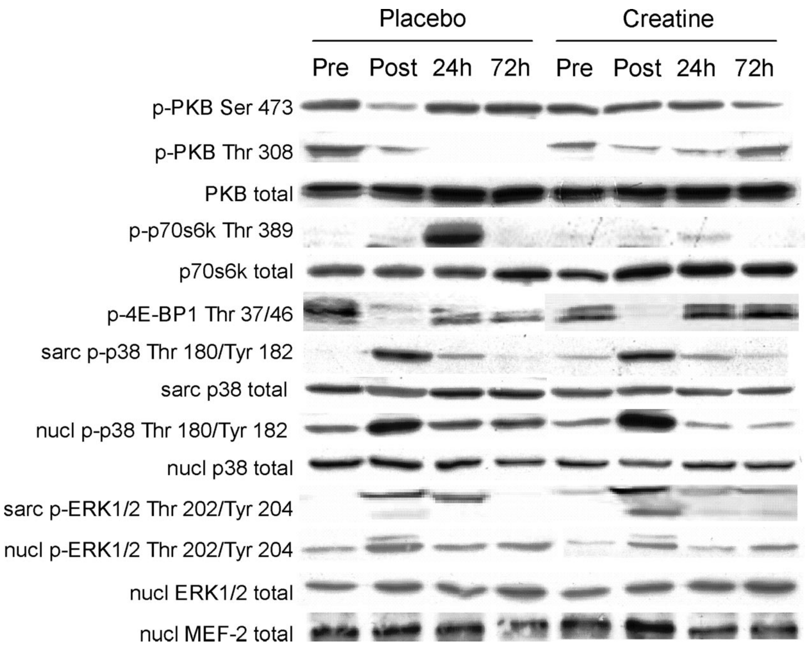

Fig. 5. Typical Western blot bands. All bands in one row have been obtained from one Western blot. $p70^{s6k}$, p70 ribosomal protein S6 kinase; 4E-BP1, eukaryotic initiation factor 4E-binding protein; MEF-2, myocyte enhancer factor 2; sarc, sarcoplasmic; nucl, nuclear; pre, preexercise; post, postexercise; 24 h, 24 h postexercise; 72 h, 72 h postexercise.

Downloaded from journals.physiology.org/journal/jappl (2804:07F0:7502:2C1D:3467:2D37:5744:F794) on June 21, 2025.

ence of biopsies sampling on gene expression (38), the effects of exercise should be analyzed with care.

A large number of studies have shown modulation of multiple gene clusters in response to resistance exercise stimulus (5, 36, 45). Indeed, a recent microarray study highlights the degree of such regulation (22). Furthermore, some gene changes may be associated with the known effects of resistance exercise on protein metabolism. It has been suggested that, during contractile activity, protein synthesis is depressed and protein breakdown is stimulated. The observed changes in the component of the ubiquitin/proteasome pathway, MAFbx, fits in well with this scenario, although we did not assess MAFbx protein level. The current results are the first to show an acute upregulation of MAFbx mRNA in human muscle immediately after exercise, likely to reflect increased protein breakdown at this time. At 24 h postexercise, MAFbx mRNA expression was reversed and depressed, suggesting a reduction in protein degradation.

Myostatin is a negative regulator of muscle mass and reportedly regulates the expression of MAFbx to modulate ubiquitin-dependent proteolysis (27). Similar to MAFbx, 24 h postexercise, mRNA for myostatin was depressed. The decrease in MAFbx and myostatin mRNA observed 24 h after exercise suggests that the ubiquitin/proteasome pathway is suppressed at this time after exercise.

Strength training in humans has been shown to result in MHC IIB-to-IIA transitions without affecting MHC I percentage (1). Since the changes in the amounts of the different MHC mRNA isoforms precede the corresponding changes at the protein level (19), our data suggest that one bout of exercise already stimulates the expression of MHC IIA observed after resistance training (1).

We observed changes in metabolic genes after resistance exercise. Immediately after exercise, IL-6 mRNA more than tripled, confirming that skeletal muscle is a major site of IL-6 production (33). GLUT-4 mRNA abundance decreased by 24 h after exercise. GLUT-4 mRNA seems to be regulated by the type of exercise. Endurance exercise increased GLUT-4 mRNA (23), whereas the opposite effect was observed after resistance exercise (6). PGC-1α mRNA has been reported to increase after resistance exercise, and the maximal expression level is reached about 3 h after exercise (7, 35, 38). We observed a significant increase immediately postexercise, suggesting that this coactivator takes part in the early response to exercise. This suggestion is in agreement with the observation that p38 is activated and MEF-2 is more abundant in the nucleus immediately after exercise since the transcriptional regulation of PGC-1α partially depends on the activation of both p38 and MEF-2 (2, 44). An increase in the expression of MEF-2 has already been reported after endurance exercise (28), but, to the best of our knowledge, this is the first study to show an increase in MEF-2 after a bout of resistance exercise.

ERK1/2 is another member of the MAPK pathways. Like p38, ERK1/2 is known to be regulated by exercise (41). ERK1/2 phosphorylation increased by more than 50-fold in the sarcoplasm and by about 8-fold in the nucleus, but the statistical significance was not reached due to the large intersubject variability.

Some studies have reported that resistance exercise activates the PKB cascade (9, 13); others have observed no changes (8, 9, 12, 14). It was thus surprising to observe a decrease in the phosphorylation state of PKB on Thr 308 and the downstream target 4E-BP1 on Thr 37/46. The same trend was found on PKB on Ser 473 and on $p70^{s6k}$ on Thr 389, but without reaching the statistical threshold. Recently, there have been few reports of an inhibition of 4E-BP1 immediately after exercise in humans (13, 21). The decrease in the PKB phosphorylation state fits well with the findings that exercise in the fasted state decreases protein synthesis and increases protein breakdown (34). Moreover, the PKB pathway is very sensitive to nutrients, but since all biopsies were taken in the fasted state this could have blunted the activation of the PKB pathway generally observed during the recovery period (10). It is likely that the results would have been different if the subjects were in a fed state.

In summary, it is generally accepted that the effects of exercise training are the results of incremental addition of repeated bouts of exercise. We found evidence to suggest that creatine induces changes in the expression of certain targeted genes as early as after 5 days of supplementation and in association with a single session of resistance exercise. The increase in collagen 1(α1) and MHC I-IIA mRNA by creatine might improve muscle framework, providing a favorable environment for muscle mass accretion after a few weeks of training. However, we have no evidence of a modulation of translational signaling when resistance exercise is coupled to creatine supplementation.

GRANTS

This work was supported by grants to M. Rennie from UK Biotechnology and Biological Sciences Research Council (BB/X510697/1 and BB/C516779/1), National Institute of Arthritis and Musculoskeletal and Skin Diseases AR-49869, and the EC EXEGENESIS program and to M. Francaux from Fonds de la Recherche Scientifique Médicale (3.4574.03).

REFERENCES

1. **Adams GR, Hather BM, Baldwin KM, Dudley GA.** Skeletal muscle myosin heavy chain composition and resistance training. *J Appl Physiol* 74: 911–915, 1993.
2. **Akimoto T, Pohnert SC, Li P, Zhang M, Gumbs C, Rosenberg PB, Williams RS, Yan Z.** Exercise stimulates Pgc-1alpha transcription in skeletal muscle through activation of the p38 MAPK pathway. *J Biol Chem* 280: 19587–19593, 2005.
3. **Alessi DR, Andjelkovic M, Caudwell B, Cron P, Morrice N, Cohen P, Hemmings BA.** Mechanism of activation of protein kinase B by insulin and IGF-1. *EMBO J* 15: 6541–6551, 1996.
4. **Baar K, Esser K.** Phosphorylation of p70(S6k) correlates with increased skeletal muscle mass following resistance exercise. *Am J Physiol Cell Physiol* 276: C120–C127, 1999.
5. **Bickel CS, Slade J, Mahoney E, Haddad F, Dudley GA, Adams GR.** Time course of molecular responses of human skeletal muscle to acute bouts of resistance exercise. *J Appl Physiol* 98: 482–488, 2005.
6. **Churchley EG, Coffey VG, Pedersen DJ, Shield A, Carey KA, Cameron-Smith D, Hawley JA.** Influence of pre-exercise muscle glycogen content on transcriptional activity of metabolic and myogenic genes in well-trained humans. *J Appl Physiol* 102: 1604–1611, 2007.
7. **Coffey VG, Shield A, Canny BJ, Carey KA, Cameron-Smith D, Hawley JA.** Interaction of contractile activity and training history on mRNA abundance in skeletal muscle from trained athletes. *Am J Physiol Endocrinol Metab* 290: E849–E855, 2006.
8. **Coffey VG, Zhong Z, Shield A, Canny BJ, Chibalin AV, Zierath JR, Hawley JA.** Early signaling responses to divergent exercise stimuli in skeletal muscle from well-trained humans. *FASEB J* 20: 190–192, 2006.
9. **Creer A, Gallagher P, Slivka D, Jemiolo B, Fink W, Trappe S.** Influence of muscle glycogen availability on ERK1/2 and Akt signaling after resistance exercise in human skeletal muscle. *J Appl Physiol* 99: 950–956, 2005.
10. **Cuthbertson DJ, Babraj J, Smith K, Wilkes E, Fedele MJ, Esser K, Rennie M.** Anabolic signaling and protein synthesis in human skeletal

Downloaded from journals.physiology.org/journal/jappl (2804:07F0:7502:2C1D:3467:2D37:5744:F794) on June 21, 2025.

muscle after dynamic shortening or lengthening exercise. *Am J Physiol Endocrinol Metab* 290: E731–E738, 2006.
11. **Deldicque L, Louis M, Theisen D, Nielens H, Dehoux M, Thissen JP, Rennie MJ, Francaux M.** Increased IGF mRNA in human skeletal muscle after creatine supplementation. *Med Sci Sports Exerc* 37: 731–736, 2005.
12. **Deshmukh A, Coffey VG, Zhong Z, Chibalin AV, Hawley JA, Zierath JR.** Exercise-induced phosphorylation of the novel Akt substrates AS160 and filamin A in human skeletal muscle. *Diabetes* 55: 1776–1782, 2006.
13. **Dreyer HC, Fujita S, Cadenas JG, Chinkes DL, Volpi E, Rasmussen BB.** Resistance exercise increases AMPK activity and reduces 4E-BP1 phosphorylation and protein synthesis in human skeletal muscle. *J Physiol* 576: 613–624, 2006.
14. **Eliasson J, Elfegoun T, Nilsson J, Kohnke R, Ekblom B, Blomstrand E.** Maximal lengthening contractions increase p70 S6 kinase phosphorylation in human skeletal muscle in the absence of nutritional supply. *Am J Physiol Endocrinol Metab* 291: E1197–E1205, 2006.
15. **Faulkner JA, Brooks SV, Opiteck JA.** Injury to skeletal muscle fibers during contractions: conditions of occurrence and prevention. *Phys Ther* 73: 911–921, 1993.
16. **Greenhaff PL, Casey A, Short AH, Harris R, Soderlund K, Hultman E.** Influence of oral creatine supplementation of muscle torque during repeated bouts of maximal voluntary exercise in man. *Clin Sci (Lond)* 84: 565–571, 1993.
17. **Guder WG, Hoffmann GE, Hubbuch A, Poppe WA, Siedel J, Price CP.** Multicentre evaluation of an enzymatic method for creatinine determination using a sensitive colour reagent. *J Clin Chem Clin Biochem* 24: 889–902, 1986.
18. **Hespel P, Op't Eijnde B, Van Leemputte M, Urso B, Greenhaff PL, Labarque V, Dymarkowski S, Van Hecke P, Richter EA.** Oral creatine supplementation facilitates the rehabilitation of disuse atrophy and alters the expression of muscle myogenic factors in humans. *J Physiol* 536: 625–633, 2001.
19. **Jaschinski F, Schuler M, Peuker H, Pette D.** Changes in myosin heavy chain mRNA and protein isoforms of rat muscle during forced contractile activity. *Am J Physiol Cell Physiol* 274: C365–C370, 1998.
20. **Karlsson HK, Nilsson PA, Nilsson J, Chibalin AV, Zierath JR, Blomstrand E.** Branched-chain amino acids increase p70S6k phosphorylation in human skeletal muscle after resistance exercise. *Am J Physiol Endocrinol Metab* 287: E1–E7, 2004.
21. **Koopman R, Zorenc AH, Gransier RJ, Cameron-Smith D, van Loon LJ.** Increase in S6K1 phosphorylation in human skeletal muscle following resistance exercise occurs mainly in type II muscle fibers. *Am J Physiol Endocrinol Metab* 290: E1245–E1252, 2006.
22. **Kostek MC, Chen YW, Cuthbertson DJ, Shi R, Fedele MJ, Esser KA, Rennie MJ.** Gene expression responses over 24h to lengthening and shortening contractions in human muscle: major changes in CSRP3, MUSTN1, SIX1 and FBXO32. *Physiol Genomics* 31: 42–52, 2007.
23. **Kraniou Y, Cameron-Smith D, Misso M, Collier G, Hargreaves M.** Effects of exercise on GLUT-4 and glycogenin gene expression in human skeletal muscle. *J Appl Physiol* 88: 794–796, 2000.
24. **Louis M, Lebacq J, Poortmans JR, Belpaire-Dethiou MC, Devogelaer JP, Van Hecke P, Goubel F, Francaux M.** Beneficial effects of creatine supplementation in dystrophic patients. *Muscle Nerve* 27: 604–610, 2003.
25. **Louis M, Poortmans JR, Francaux M, Berre J, Boisseau N, Brassine E, Cuthbertson DJ, Smith K, Babraj JA, Waddell T, Rennie MJ.** No effect of creatine supplementation on human myofibrillar and sarcoplasmic protein synthesis after resistance exercise. *Am J Physiol Endocrinol Metab* 285: E1089–E1094, 2003.
26. **Louis M, Poortmans JR, Francaux M, Hultman E, Berre J, Boisseau N, Young VR, Smith K, Meier-Augenstein W, Babraj JA, Waddell T, Rennie MJ.** Creatine supplementation has no effect on human muscle protein turnover at rest in the postabsorptive or fed states. *Am J Physiol Endocrinol Metab* 284: E764–E770, 2003.
27. **McFarlane C, Plummer E, Thomas M, Hennebry A, Ashby M, Ling N, Smith H, Sharma M, Kambadur R.** Myostatin induces cachexia by activating the ubiquitin proteolytic system through an NF-kappaB-independent, FoxO1-dependent mechanism. *J Cell Physiol* 209: 501–514, 2006.
28. **McGee SL, Sparling D, Olson AL, Hargreaves M.** Exercise increases MEF2- and GEF DNA-binding activity in human skeletal muscle. *FASEB J* 20: 348–349, 2006.
29. **Miller BF, Olesen JL, Hansen M, Dossing S, Crameri RM, Welling RJ, Langberg H, Flyvbjerg A, Kjaer M, Babraj JA, Smith K, Rennie MJ.** Coordinated collagen and muscle protein synthesis in human patella tendon and quadriceps muscle after exercise. *J Physiol* 567: 1021–1033, 2005.
30. **Murphy RM, Watt KK, Cameron-Smith D, Gibbons CJ, Snow RJ.** Effects of creatine supplementation on housekeeping genes in human skeletal muscle using real-time RT-PCR. *Physiol Genomics* 12: 163–174, 2003.
31. **Nader GA, Esser KA.** Intracellular signaling specificity in skeletal muscle in response to different modes of exercise. *J Appl Physiol* 90: 1936–1942, 2001.
32. **Olsen S, Aagaard P, Kadi F, Tufekovic G, Verney J, Olesen JL, Suetta C, Kjaer M.** Creatine supplementation augments the increase in satellite cell and myonuclei number in human skeletal muscle induced by strength training. *J Physiol* 573: 525–534, 2006.
33. **Pedersen BK, Ostrowski K, Rohde T, Bruunsgaard H.** The cytokine response to strenuous exercise. *Can J Physiol Pharmacol* 76: 505–511, 1998.
34. **Phillips SM, Tipton KD, Aarsland A, Wolf SE, Wolfe RR.** Mixed muscle protein synthesis and breakdown after resistance exercise in humans. *Am J Physiol Endocrinol Metab* 273: E99–E107, 1997.
35. **Pilegaard H, Saltin B, Neufer PD.** Exercise induces transient transcriptional activation of the PGC-1alpha gene in human skeletal muscle. *J Physiol* 546: 851–858, 2003.
36. **Psilander N, Damsgaard R, Pilegaard H.** Resistance exercise alters MRF and IGF-I mRNA content in human skeletal muscle. *J Appl Physiol* 95: 1038–1044, 2003.
37. **Tarnopolsky M, Martin J.** Creatine monohydrate increases strength in patients with neuromuscular disease. *Neurology* 52: 854–857, 1999.
38. **Vissing K, Andersen JL, Schjerling P.** Are exercise-induced genes induced by exercise? *FASEB J* 19: 94–96, 2005.
39. **Volek JS, Duncan ND, Mazzetti SA, Staron RS, Putukian M, Gomez AL, Pearson DR, Fink WJ, Kraemer WJ.** Performance and muscle fiber adaptations to creatine supplementation and heavy resistance training. *Med Sci Sports Exerc* 31: 1147–1156, 1999.
40. **Vorgerd M, Grehl T, Jager M, Muller K, Freitag G, Patzold T, Bruns N, Fabian K, Tegenthoff M, Mortier W, Luttmann A, Zange J, Malin JP.** Creatine therapy in myophosphorylase deficiency (McArdle disease): a placebo-controlled crossover trial. *Arch Neurol* 57: 956–963, 2000.
41. **Widegren U, Ryder JW, Zierath JR.** Mitogen-activated protein kinase signal transduction in skeletal muscle: effects of exercise and muscle contraction. *Acta Physiol Scand* 172: 227–238, 2001.
42. **Williamson D, Gallagher P, Harber M, Hollon C, Trappe S.** Mitogen-activated protein kinase (MAPK) pathway activation: effects of age and acute exercise on human skeletal muscle. *J Physiol* 547: 977–987, 2003.
43. **Willoughby DS, Rosene J.** Effects of oral creatine and resistance training on myosin heavy chain expression. *Med Sci Sports Exerc* 33: 1674–1681, 2001.
44. **Wright DC, Han DH, Garcia-Roves PM, Geiger PC, Jones TE, Holloszy JO.** Exercise-induced mitochondrial biogenesis begins before the increase in muscle PGC-1alpha expression. *J Biol Chem* 282: 194–199, 2007.
45. **Yang Y, Creer A, Jemiolo B, Trappe S.** Time course of myogenic and metabolic gene expression in response to acute exercise in human skeletal muscle. *J Appl Physiol* 98: 1745–1752, 2005.

Downloaded from journals.physiology.org/journal/jappl (2804:07F0:7502:2C1D:3467:2D37:5744:F794) on June 21, 2025.
