# Supplementary material for: Impact of creatine supplementation on inflammation: evidence from a systematic review and meta-analysis of randomized double-blind placebo trials
Source: Front Immunol. 2026 Feb 19;17:1743603. doi: 10.3389/fimmu.2026.1743603 (PMC12961398; doi:10.3389/fimmu.2026.1743603)
Supplement: Supplementary file 2 [file SupplementaryFile1.zip › SR Creatine inflammatory markers (Kell Doutorado). /Supplementary Files/Final References/Final/Bassit et al 2008.pdf]

Amino Acids (2008) 35: 425–431
DOI 10.1007/s00726-007-0582-4
Printed in The Netherlands

Amino Acids

Creatine supplementation reduces plasma levels of pro-inflammatory cytokines and PGE_2 after a half-ironman competition

R. A. Bassit[1], **R. Curi**[2], and **L. F. B. P. Costa Rosa**[1,†]

[1] Department of Cellular Biology, Institute of Biomedical Sciences, University of São Paulo, São Paulo, Sp, Brazil
[2] Department of Physiology and Biophysics, Institute of Biomedical Sciences, University of São Paulo, São Paulo, Sp, Brazil

Received May 3, 2007
Accepted July 5, 2007
Published online October 4, 2007; © Springer-Verlag 2007

Summary. *Objective.* The effect of creatine supplementation upon plasma levels of pro-inflammatory cytokines: Interleukin (IL) 1β and IL-6, Tumor Necrosis Factor α (TNFα), and Interferon α (INFα) and Prostaglandin E_2 (PGE_2) after a half-ironman competition were investigated.

Methods. Eleven triathletes, each with at least three years experience of participation in this sport were randomly divided between the control and experimental groups. During 5 days prior to competition, the control group ($n = 6$) was supplemented with carbohydrate ($20\,g \cdot d^{-1}$) whereas the experimental group ($n = 5$) received creatine ($20\,g \cdot d^{-1}$) in a double-blind trial. Blood samples were collected 48 h before and 24 and 48 h after competition and were used for the measurement of cytokines and PGE_2.

Results. Forty-eight hours prior to competition there was no difference between groups in the plasma concentrations ($pg \cdot ml^{-1}$, mean ± SEM) of IL-6 (7.08 ± 0.63), TNFα (76.50 ± 5.60), INFα (18.32 ± 1.20), IL-1β (23.42 ± 5.52), and PGE_2 (39.71 ± 3.8). Twenty-four and 48 h after competition plasma levels of TNFα, INFα, IL-1β and PGE_2 were significantly increased ($P < 0.05$) in both groups. However, the increases in these were markedly reduced following creatine supplementation. An increase in plasma IL-6 was observed only after 24 h and, in this case, there was no difference between the two groups.

Conclusion. Creatine supplementation before a long distance triathlon competition may reduce the inflammatory response induced by this form of strenuous of exercise.

Keywords: Long distance triathlon – Muscle inflammation – Muscle damage – Eccentric contraction – Pro-inflammatory cytokines

Introduction

The growing interest in triathlon is based on the original structure of the sport itself and in the wide variety of distances offered to participants, ranging from sprint to ironman distance, and lasting from 1 to 10 h, respectively. The triathlon comprises sequentially of swimming, cycling, and running. The leading causes of trauma during and after endurance exercise are a) eccentric muscle contractions, as in the long distance triathlon, b) the impact of the extremities against the ground and c) the number of repetitions of the same movement (Bansil et al., 1985). Delayed onset muscle soreness (DOMS) develops 24–48 h after as a consequence of prolonged eccentric muscle contraction and the strenuous nature of events such as long distance triathlon. (Bansil et al., 1985; Milles and Clarkson, 1994; Egermann, et al., 2003; Volek and Rawson, 2004). The discomfort experienced by athletes is generally accompanied by prolonged muscle-strength loss, a reduced range of motion, and high levels of creatine kinase activity in the blood (Milles and Clarkson, 1994). Muscle swelling and the sensation of DOMS suggests that physical exercise causes muscle inflammation, especially if the exercise is strenuous and/or involves eccentric contractions as in marathon or long distance triathlon (Malloch and Taunton, 2000; Malm, 2001; Clarkson and Hubal, 2002). The passive elements (connective tissue and muscle fibers) can absorb strain and this ability increases by as much as 100% after muscle activation (Malloch and Taunton, 2000). However, when muscle activation is compromised, as during fatigue caused by endurance exercise, the ability to absorb strain is reduced, increasing muscle vulnerability to trauma (Malloch and Taunton, 2000).

The plasma levels of IL-1β, IL-6, TNFα, INFα, and C reactive protein are known to increase during strenuous exercise (Malm, 2001; Clarkson and Hubal, 2002; Nosaka and Newton, 2002). Exercise also induces an increase in prostaglandin E_2 (PGE_2) production, as part of the inflammatory response triggered by micro trauma occurring

[†] In memoriam.

in the skeletal muscles (Bansil et al., 1985; Smith and Miles, 2000). There is an increase of PGE_2 synthesis by infiltrating macrophages in the inflamed muscle, and this is a key mediator of pain 24–48 h after an exercise session (Volek and Rawson, 2004).

Creatine improves performance during repeated bouts of high intense exercise when supplemented for a short period of time (Mujika and Padilla, 1997; Lawler et al., 2002). The typical programme of creatine intake consists of 20 g per day for 5–7 days followed by a maintenance load of 3–5 g per day (Harris et al., 1992; Milles and Clarkson, 1994; Bemben and Lamont, 2005). The greatest uptake of creatine by the muscle occurs during the initial stages of the loading regime (Harris et al., 1992). Exercise seems to enhance the uptake of creatine (Harris et al., 1992), especially if ingested with a carbohydrate drink after exertion (Terjung et al., 2000). Oral intake of creatine is known to increase glycogen concentrations in human skeletal muscle by up to 40% (Op T'Eijnde et al., 2001). Creatine supplementation also causes weight gain by increasing the retention of intracellular water, increasing muscle cell volume (Demant and Rhodes, 1999; Terjung et al., 2000). Moreover, creatine supplementation has been reported to maintain muscle integrity, reducing muscle damage and inflammatory responses and attenuating the increase in plasma PGE_2 levels (Santos et al., 2004).

In the present report we investigated the effects of creatine supplementation upon plasma levels of pro-inflammatory cytokines (IL-1β, IL-6, TNFα and INFα) and PGE_2 after a half-ironman competition consisting of 1.9 km of swimming, 90 km of cycling and 21 km of running. In summary, creatine supplementation for five days prior to the competition was shown to reduce the plasma levels of the most cytokines evaluated, except for IL-6, and PGE_2 even after 48 h of the competition.

Materials and methods

Subjects and experimental

The experimental protocol was approved by the Ethics Committee of the Institute of Biomedical Sciences, University of Sao Paulo, São Paulo, Brazil. The subjects were selected from a group of non-smoking athletes who were in training for a half-ironman triathlon (Long Distance Triathlon Brazilian Championship). None of the athletes had a history of consuming forbidden drugs or anti-inflammatory medications and did not take creatine as a supplement. The athletes average age was 40.3 years, range 34–56 years. The range in self-reported personal best performance in a half-ironman competition was 266–311 min. In the present study the athletes completed the half-ironman triathlon competition in the range of 280–329 min (4 h 40 min–5 h 29 min – Table 1). All athletes had previously participated in at least three half-ironman triathlon competitions.

After signing an informed consent form, eleven male athletes were randomly divided into the control (Pl – $n = 6$) and experimental (Cr – $n = 5$) groups. During five days prior to the competition, athletes from the experimental group received 20 g per day of creatine monohydrate divided in two equal doses. These were ingested at 10 am and 4 pm. Athletes from the control group similarly received 20 g per day of carbohydrate in place of creatine. The creatine or carbohydrate supplementation was supplied to athletes as a ready-to-consume powder mixture containing maltodextrin 50 g. The athletes from both groups were instructed to dilute the powder with the same volume of water just before the ingestion. The mixture offered for both groups had similar volume, flavour and color to avoid identification of the supplements. The athletes were allowed to drink and eat normally during the five days that preceded the competition. All experiments were conducted as a double-blind trial. The race started at 8:30 am with 30 °C and 90% relative-humidity. Conditions at the end of the competition were 38 °C and 80% RH. All athletes finished the race within 5% of their best personal time for that distance.

Table 1. Physical data and degree of training (triathletes, $n = 11$)

	Mean ± SEM	Range
Age (yrs)	40.3 ± 2.18	34–56
Weight (kg)	76.3 ± 2.29	63.6–85.0
Height (cm)	178.2 ± 2.04	170–190
Personal best (min)	290.5 ± 5.05	266–311
Regular triathlon (yrs)	5.3 ± 0.80	3–10
Half-ironman time (min)	302.2 ± 4.52	280–329
Training (time/wk)	177.0 ± 12.8	120–240
VO_2 max. $(ml/kg \cdot min^{-1})$	52.3 ± 1.34	45.3–58.0

Physical data and the degree of training showed that the triathletes were homogeneous in terms of running experience and performance in a half-ironman competition (Table 1).

Blood sampling

Blood samples (20 ml) were collected from an antecubital vein 48 h before the start, and 24 and 48 h after the end of the competition, into sterile heparinized glass tubes. Blood samples were centrifuged at $650 \times g$ for 15 min and plasma was kept at −70 °C for one week until analysed for cytokines (IL-1, IL-6, TNFα and INFα) and prostaglandin E_2 (PGE_2).

Measurements of the cytokines and PGE_2

Plasma IL-1β, IL-6, TNFα, INFα and PGE_2 concentrations were determined using commercially available ELISA-kits (Biotrak – cellular communication assays, Amersham Pharmacia biotech, Little Chalfont Buckinghamshire, UK).

Statistical analysis

The results were compared using two-way ANOVA. Group means were further compared using the post-hoc test of Bonferroni. A level of significance of at least $p < 0.05$ was chosen for all comparisons. All data were analyzed using Graph Pad Prism program and graph package (V4.0, Graph Pad Inc., San Diego, CA, USA). The results are presented as mean ± SEM.

Results

Forty-eight hours before the half-ironman competition plasma concentrations (mean ± SEM) of IL-6 ($7.08 \pm 0.63\,pg \cdot ml^{-1}$), TNFα ($76.50 \pm 5.60\,pg \cdot ml^{-1}$), INFα ($18.32 \pm 1.20\,pg \cdot ml^{-1}$), IL-1β ($23.42 \pm 3.52\,pg \cdot ml^{-1}$), and PGE_2 ($39.71 \pm 3.8\,pg \cdot ml^{-1}$) were within the normal

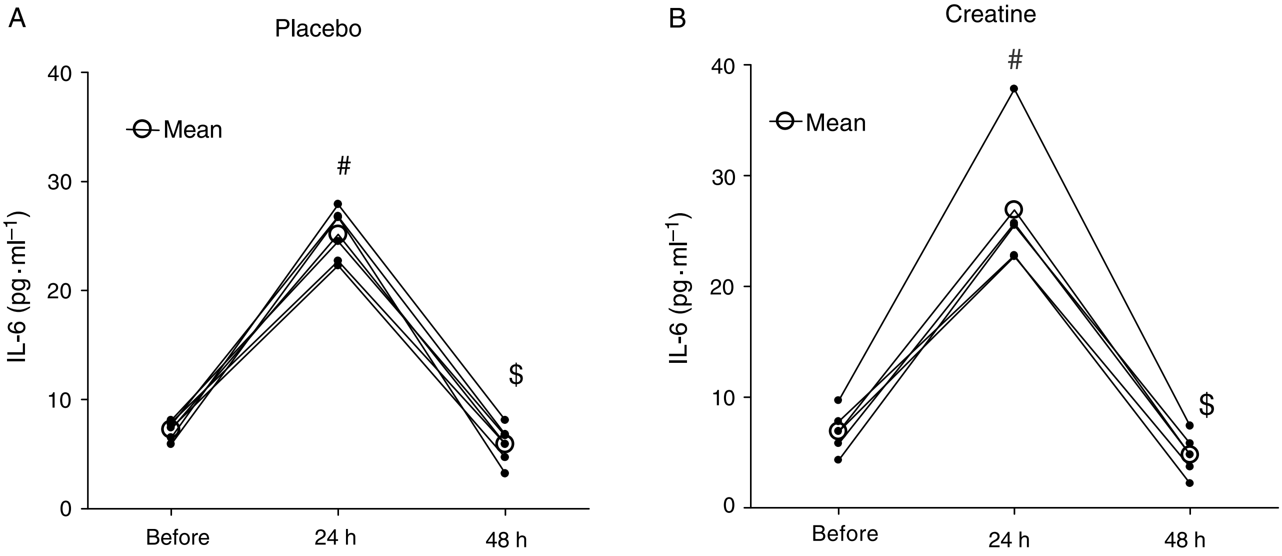

Fig. 1. Interleukin-6 (IL-6) plasma concentration. IL-6 levels was determined 48 h before, and 24 and 48 h after the end of the competition in plasma collected from triathletes that received either placebo (Pl, $n = 6$) or creatine supplementation (Cr, $n = 5$). The results are expressed as mean. Standard errors of the means were always lower of the 20% of the mean value. $^{*}\,p < 0.05$ for comparison with the creatine supplemented group. $^{\#}\,p < 0.05$ for comparison with the data before the competition in both groups. $^{\$}p < 0.05$ for comparison between 48 and 24 h in both groups

expected range (Santos et al., 2004). There were no significant differences between the control and experimental groups.

Following competition plasma IL-6 at 24 h was increased 3.5-fold (7.25 ± 0.35 vs. 25.15 ± 0.88; $p < 0.05$) and 3.9-fold (6.90 ± 0.91 vs. 29.90 ± 2.80; $p < 0.05$) in the control and experimental groups, respectively (Fig. 1). However, by 48 h IL-6 had fallen back to 76.5% (5.90 ± 0.71; $p < 0.05$) and 82.2% (4.78 ± 0.89; $p < 0.05$) of the pre-competition concentration in the control and experimental groups, respectively. There was no effect of creatine supplementation on the response of plasma IL-6 to exercise under the conditions of this study.

Plasma TNFα was increased in the control group 3.5-fold at 24 h to 287.48 ± 8.50 ($p < 0.05$) and 4-fold at 48 h to 324.40 ± 9.94 ($p < 0.05$) compared to the concentration

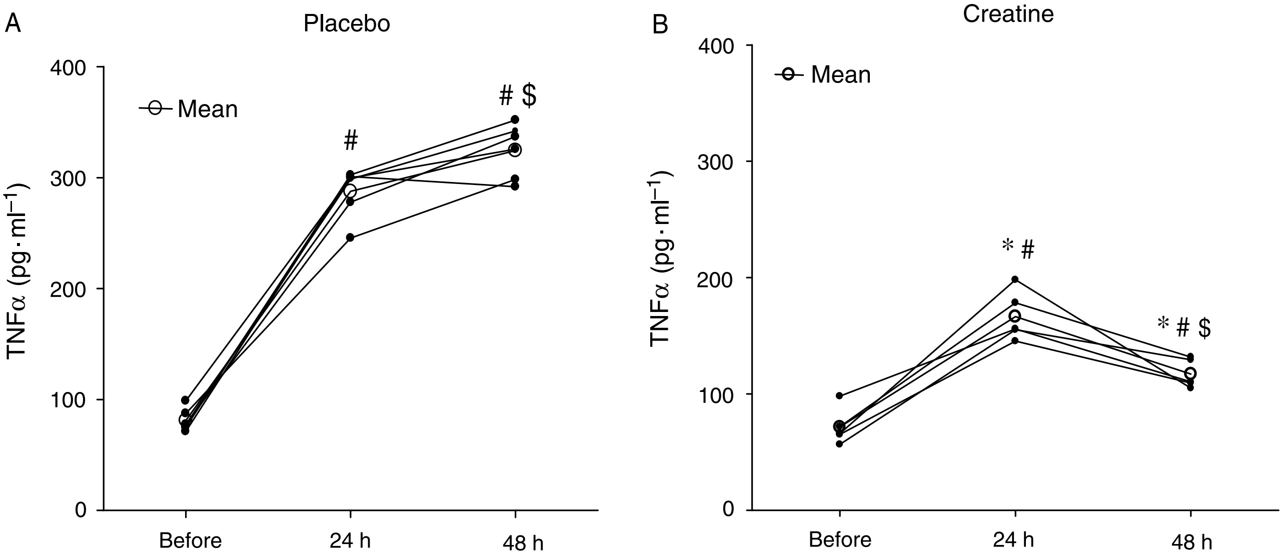

Fig. 2. Tumor Necrosis Factor α (TNFα) plasma concentration. TNFα levels was determined 48 h before, and 24 and 48 h after the end of the competition in plasma collected from triathletes that received either placebo (Pl, $n = 6$) or creatine supplementation (Cr, $n = 5$). The results are expressed as mean. Standard errors of the means were always lower of the 10% of the mean value. $^{*}\,p < 0.05$ for comparison with the creatine supplemented group. $^{\#}p < 0.05$ for comparison with the data before the competition in both groups. $^{\$}p < 0.05$ for comparison between 48 and 24 h in both groups

before competition (81.37 ± 4.15) (Fig. 2). The further increase from 24 to 48 h was significant. Creatine supplementation significantly reduced the increases in TNFα at 24 and 48 h by 42 and 64%. Concentrations at these times (166.64 ± 9.54 and 117.22 ± 5.55) were significantly lower ($p < 0.05$) than those in the control group (Fig. 2). The decrease from 24 to 48 h was significant.

Plasma INFα was increased in the control group 16.5-fold at 24 h to 296.93 ± 8.29 ($p < 0.05$) and 16.3-fold at 48 h to 293.33 ± 5.39 ($p < 0.05$) compared to the concentration before competition (18.05 ± 1.30) (Fig. 3). The change from 24 to 48 h was not significant. Creatine supplementation significantly reduced ($p < 0.05$) the increase in INFα at 24 and 48 h by 50.5 and 80.1%. Concentrations at these times (147.08 ± 2.46 and 58.24 ± 6.10) were significantly lower than the corresponding concentrations in the control group (Fig. 3). The decrease from 24 to 48 h was significant ($P < 0.05$) by 60.4%.

Plasma IL1-β was increased in the control group 6.9-fold at 24 h to 157.50 ± 7.74 ($p < 0.05$) and 6.9-fold at

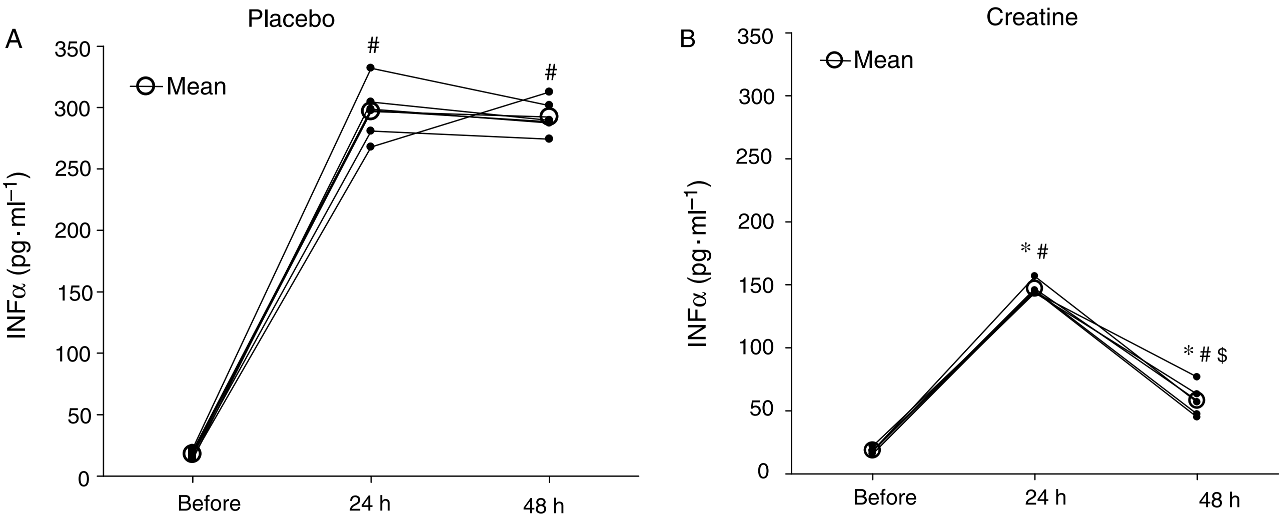

Fig. 3. Interferonα (INFα) plasma concentration. INFα levels was determined 48 h before, and 24 and 48 h after the end of the competition in plasma collected from triathletes that received either placebo (**A**, Pl, $n = 6$) or creatine supplementation (**B**, Cr, $n = 5$). The results are expressed as mean. Standard errors of the means were always lower of the 15% of the mean value. $^{*}p < 0.05$ for comparison with the creatine supplemented group. $^{\#}p < 0.05$ for comparison with the data before the competition in both groups. $^{\$}p < 0.05$ for comparison between 48 and 24 h in both groups

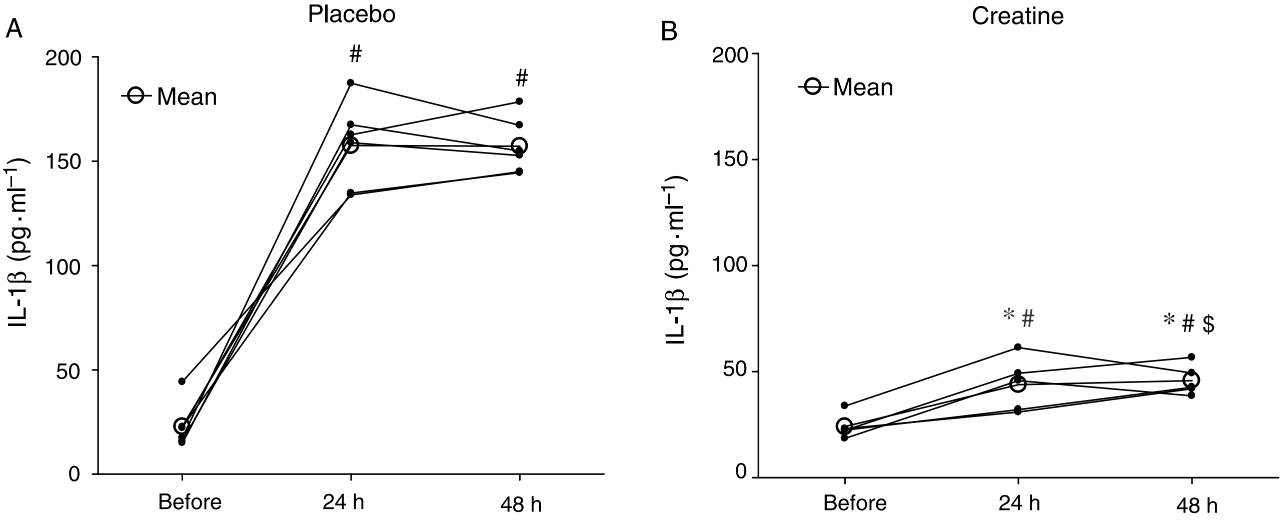

Fig. 4. Interleukin-1 β (IL-1β) plasma concentration. IL-1β levels was determined 48 h before, and 24 and 48 h after the end of the competition in plasma collected from triathletes that received either placebo (**A**, Pl, $n = 6$) or creatine supplementation (**B**, Cr, $n = 5$). The results are expressed as mean. Standard errors of the means were always lower of the 20% of the mean value. $^{*}p < 0.05$ for comparison with the creatine supplemented group. $^{\#}p < 0.05$ for comparison with the data before the competition in both groups. $^{\$}p < 0.05$ for comparison between 48 and 24 h in both groups

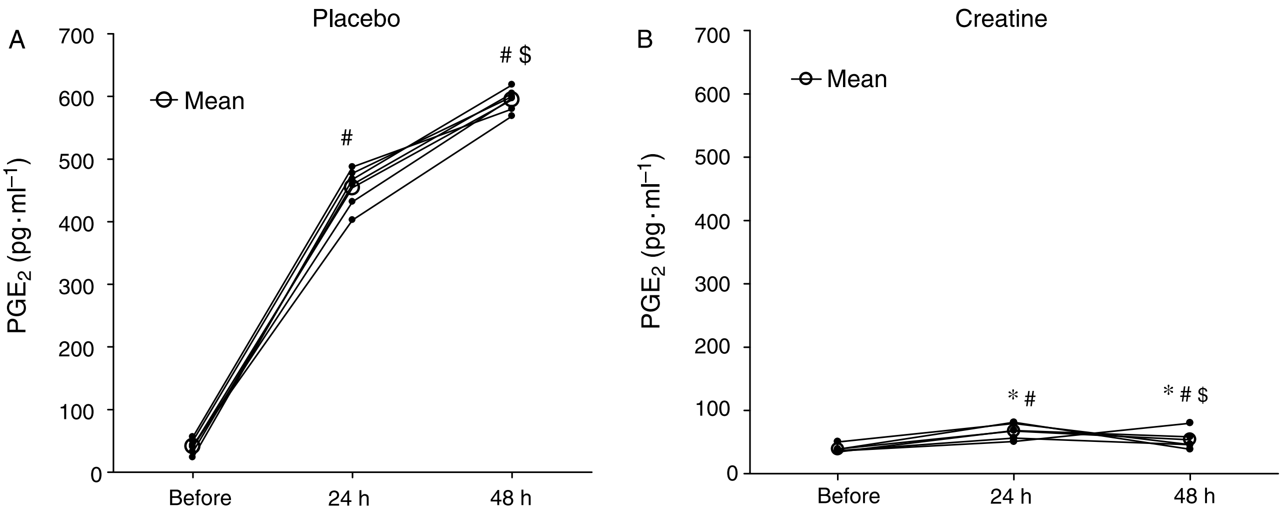

Fig. 5. Prostaglandin E_2 (PGE_2) plasma concentration. PGE_2 levels was determined 48 h before, and 24 and 48 h after the end of the competition in plasma collected from triathletes that received either placebo (**A**, Pl, $n=6$) or creatine supplementation (**B**, Cr, $n=5$). The results are expressed as mean. Standard errors of the means were always lower of the 15% of the mean value. $^{*}p<0.05$ for comparison with the creatine supplemented group. $^{\#}p<0.05$ for comparison with the data before the competition in both groups. $^{\$}p<0.05$ for comparison between 48 and 24 h in both groups

48 h to 157.15 ± 5.44 ($p<0.05$) compared to the concentration before competition (22.82 ± 4.47) (Fig. 4). The change from 24 to 48 h was not significant. Creatine supplementation significantly ($p<0.05$) reduced the increases in IL1-β at 24 and 48 h by 72 and 71%. Concentrations at these times (43.86 ± 5.67 and 45.82 ± 3.23) were significantly lower than the corresponding concentrations in the control group ($p<0.05$) (Fig. 4).

Plasma PGE_2 was increased in the control group 11-fold at 24 h to 454.37 ± 11.96 ($p<0.05$) and 14.4-fold at 48 h to 594.7 ± 7.3 ($p<0.05$) compared to the concentration before competition (41.22 ± 4.79) (Fig. 5). The change from 24 to 48 h was significant. Creatine supplementation significantly reduced ($p<0.05$) the increase in PGE_2 at 24 and 48 h by 85.5 and 91%. Concentrations at these times (65.70 ± 5.85 and 52.56 ± 7.10) were significantly lower than the corresponding concentrations in the control group ($p<0.05$) (Fig. 5).

Discussion

The major finding of the present report is that athletes receiving creatine supplementation during the five day period that precedes the half-ironman shows significantly lower levels of pro-inflammatory mediators, such as TNFα, INFα, IL1-β and PGE_2, at 24 and 48 h after the competition.

In response to an infectious agent or a nonspecific form of tissue injury, the host shows an acute inflammatory reaction in the affected tissue. Polymorphonuclear neutrophils (PMNs), mast cells, and macrophages are important cellular components of this inflammatory process. Pro-inflammatory mediators such as prostaglandins are released from PMNs and/or macrophages and various cytokines are released from macrophages and/or lymphocytes. The pro-inflammatory cytokines (IL-1β, IL-6, and TNFα) evaluated herein are derived mainly from macrophages and have many systemic and metabolic effects. Of the major importance is the impact of these cytokines on the liver, leading to production of a number of proteins generally named *acute phase proteins* (APP). These events are collectively called as the acute phase response and are characterized by fever, leukocytosis, decreased appetite, altered sleep patterns, and malaise, also referred to as sickness behavior. The early or alarm cytokines are IL-1β and TNFα, which act locally on fibroblasts and endothelial cells to induce the production of other cytokines such as IL-6 acting centrally through the induction of prostaglandins to cause fever and the sickness behavior (Baumann and Glaudie, 1994). Both primary and secondary cytokines initiate other cytokine cascades that make up the acute phase response. For instance, the APP produced by the liver have a wide range of activities, such as neutralizing inflammatory agents to minimize the extent of the damage or participating in the repair of tissues that contribute to the host defense (Steel and Witehead, 1994).

The production of pro-inflammatory cytokines, such as IL1β, IL-6, TNFα, and INFα, is increased during intense and prolonged exercise (Mackinnon, 1999). These type of exercise may cause damage to and inflammation within skeletal muscle (Malm, 2001). The increase in IL-1β, and

TNFα levels may be a result of high plasma levels of stress hormones such as catecholamines and corticosteroids (Cupps and Fauci, 1982; Nieman and Nehlsen-Cannarella, 1991); the plasma levels of these hormones rise dramatically during physical exercise (Farrel et al., 1983; Mackinnon, 1999). The overall damage in tissues is characterized by movement of fluid and plasma proteins. Neutrophils represent the first wave of infiltrating cells, followed by monocytes (Smith and Miles, 2000). In addition, the overt signs and symptoms of the inflamed tissue include swelling, redness, hest, pain, and loss of or reduced function (Smith and Miles, 2000). Skeletal muscle injury manifests within few hours, increasing as exercise progresses, and it is still apparent after completion of the exercise session (Farber et al., 1991). Thus, considering that *delay onset muscle soreness* (DOMS) develops 24–48 h after strenuous exercise as a consequence of eccentric muscle contraction or strenuous endurance events, blood samples collected within this period should reflect the plasma levels of pro-inflammatory mediators (Thompson et al., 1997; Egermann et al., 2003).

As observed herein, in the half-ironman triathlon competition, the plasma levels of pro-inflammatory cytokines markedly increased after the competition. Another aspect related to the muscle inflammation process is the increase in plasma levels of PGE_2 observed 48 h after 30 km race in marathon runners (Santos et al., 2004). PGE_2 is actively synthesized by macrophages upon exposure to an inflammatory environment and it has been implicated in the pain following exertion (Thompson et al., 1997). This fact is in agreement with the increase in PGE_2 plasma concentrations observed in the present study.

Data from muscle biopsies indicate that IL-1β and IL-6 are produced within skeletal muscle during and after exercise being associated with muscle damage (Canonn et al., 1989; Rohde et al., 1997). Inflammatory activity within skeletal muscle can also be driven by the local endothelial cells. IL-1β has been implicated in muscle proteolysis and repair following injury/inflammation (Mackinnon, 1999). However, muscle contraction, even in the absence of markers of muscle damage, rapidly increases IL-6 mRNA expression as observed in skeletal muscle biopsy samples (Smith and Miles, 2000; Pedersen et al., 2003; Bemben and Lamont, 2005). IL-6 production by skeletal muscle varies with exercise intensity, duration, the mass of muscle recruited, and endurance capacity (Pedersen et al., 2003, 2004; Febbraio et al., 2004). The release of this cytokine by skeletal muscle may play a role to mobilize substrates for energy production (O'Toole et al., 1989; Op T'Eijnde et al., 2001). Recent study has shown that IL-6 is released from skeletal muscle during exercise and that carbohydrate ingestion attenuates the increase in the production of this cytokine during both running and cycling (Pedersen and Febbraio, 2005).

Skeletal muscle contraction is a powerful stimulus for glucose disposal and uptake leading to hypoglycemia if the endogenous glucose production and output from the liver are not stimulated at the same extent during exercise (Pedersen and Febbraio, 2005). IL-6 influences glucose homeostasis during exercise and provides potential new insights into factors that mediate glucose production and disposal. There is strong evidence that IL-6 may also affect lipid metabolism in humans resulting in lypolysis and fatty acid oxidation (Pedersen and Febbraio, 2005).

The increase in IL-6 after 24 h may be important to maintain blood glucose levels, to inhibit TNFα production and to increase insulin sensitivity protecting against certain disorders, such as type 2 diabetes (Pedersen and Hoffman-Goetz, 2000). Usually IL-6 is referred as an "inflammation-responsive" cytokine rather than a pro-inflammatory cytokine as IL-6 directly do not induce inflammation. Additionally, proinflammatory cytokines such as IL-1β and TNFα have been shown to induce PGE_2 synthesis in endothelial cells, smooth muscle cells, and skeletal muscle. IL-6 production and release plasma seems to precede neutrophil and macrophage accumulation in the muscle, as a high level of IL-6 is found immediately after an exhaustive exercise bout (Pedersen and Hoffman-Goetz, 2000).

The plasma levels of TNF-α is also elevated in the inflammation process, however, chronic muscular activity down regulates this cytokine expression in contracting skeletal muscle of elderly humans (Pedersen et al., 2004; Pedersen and Febbraio, 2005).

The creatine supplementation tested in the present study was able to reduce the increase in plasma levels of the pro-inflammatory cytokines (IL1β, TNFα, and INFα) in addition to PGE_2, when compared with the placebo group, in both situations (24 and 48 h after competition). In spite of this, however, the mechanism involved remains to be determined. There is possible that creatine supplementation may reduce muscle cell death and as consequence the inflammatory process as whole. The absence of effect of creatine supplementation on IL-6 plasma may reflect on adjustment of the changes induced in the remaining cytokines.

In conclusion, the results presented herein suggest that creatine supplementation during a short period of five days before the half-ironman triathlon may attenuate the increase in plasma levels of pro-inflammatory cytokines and PGE_2. Further experiments are now required to investigate if this anti-inflammatory response is due to systemic

and/or local effect of creatine on leukocytes (macrophages) and/or the exercised muscle.

Acknowledgements

The authors are thankful to the athletes who made this study possible, to Roberto Rivelino for blood sampling and to Dr. Mayana Zatz, from the Human Genome Institute, University of São Paulo, for making the facilities available for blood collection, and to Dr. Emer S. Ferro, University of São Paulo, for critical reading of this manuscript. The authors are also grateful to Fundação de Amparo a Pesquisa do Estado de São Paulo (FAPESP) for financial support. RAB was a fellowship recipient from FAPESP (04/00573-1).

References

Bansil CK, Wilson GD, Stone MH (1985) Role of prostaglandins E and F2a in exercise-induced delayed muscle soreness. Med Sci Sports Exerc 17: 276–280

Baumann H, Glaudie J (1994) The acute phase response. Immunol Today 15: 74

Bemben MG, Lamont HS (2005) Creatine supplementation and exercise performance. Sports Med 35: 107–125

Canonn JG, Fielding RA, Fiatore MA, Orencole SJ, Dinarello CA, Evans WJ (1989) Increased interkeukin 1β in human skeletal muscle after exercise. Am J Physiol 257: R451–R455

Clarkson PM, Hubal MJ (2002) Exercise-induced muscle damage in humans. Am J Phys Med Rehabil 81: S52–S69

Cupps TR, Fauci AS (1982) Corticosteroid-mediated immunoregulation in man. Immunol Rev 65: 133–155

Demant TW, Rhodes EC (1999) Effects of creatine supplementation on exercise performance. Sports Med 28: 49–60

Egermann M, Brocai D, Lill CA, Schmitt H (2003) Analysis of injuries in long-distance triathletes. Int J Sports Med 24: 271–276

Farber HW, Schaefer EJ, Franey R, Grimaldi R, Hill HS (1991) The endurance triathlon: metabolic changes after each event and during recovery. Med Sci Sports Exerc 23: 959–965

Farell PA, Garthwait TL, Gustafson AB (1983) Plasma adrenocorticotrofin and cortisol responses to submaximal and exhaustive exercise. J Appl Physiol 55: 1441–1444

Febbraio MA, Hiscock N, Sacchetti M, Fische RCP, Pedersen BK (2004) Interleukin-6 is novel factor mediating glucose homeostasis during skeletal contraction. Diabetes 53: 1643–1648

Harris RC, Soderlund K, Hultman E (1992) Elevation of creatine in resting and exercise muscle of normal subjects of creatine supplementation. Clin Sci 83: 367–374

Lawler JM, Barnes WS, Wu G, Song W, Demaree S (2002) Direct antioxidant properties of creatine. Biochem Biophys Res Commun 290: 47–52

Mackinnon LT (1999) Advanced in exercise immunology. Human Kinetics, Champaign, IL

Malloch AJ, Taunton JE (2000) Overuse syndrome. In: Shepard P-O, Astrand RJ (eds) Endurance in sport. Blackwell Science, Oxford, pp 766–799

Malm C (2001) Exercise-induced muscle damage and inflammation: fact or fiction? Acta Physiol Scand 171: 233–239

Milles MP, Clarkson PM (1994) Exercise-induced muscle pain, soreness, and cramps. J Sports Med Phys Fitness 34: 203–206

Mujika I, Padilla S (1997) Creatine supplementation as an ergogenic acid for sports performance in highly trained athletes: a critical review. Int J Sports Med 18: 491–496

Nieman DC, Nehlsen-Cannarella SL (1992) Effects of endurance exercise on immune response. In: Shephard RJ, Astrand PO (eds) Endurance in sport. Blackwell, Oxford, pp 487–504

Nosaka K, Newton M (2002) Concentric or eccentric training effect on eccentric exercise-induced muscle damage. Med Sci Sports Exerc 31: 63–69

Op T'Eijnde B, Ritcher EA, Henquin JC, Kiens B, Hespel P (2001) Effect of creatine supplementation on creatine and glycogen content in rat skeletal muscle. Acta Physiol Scand 171: 169–176

O'Toole ML, Douglas PS, Hiller WD (1989) Lactate, oxygen uptake, and cycling performance in triathletes. Int J Sports Med 10: 413–418

Pedersen BK, Febbraio M (2005) Muscle-derived intereukin-6 – a possible link between skeletal muscle, adipose tissue, liver, and brain. Brain Behav Immun 19: 371–376

Pedersen BK, Steensberg A, Fischer C, Keller C, Keller P, Promgaard P, Febbraio M, Saltin B (2003) Searching for the exercise factor: is IL-6 a candidate? J Muscle Res Cell Motil 24: 113–119

Pedersen M, Steensberg A, Keller C, Osaka T, Zacho M, Saltin B, Febbraio MA, Pedersen BK (2004) Does the aging skeletal muscle maintain its endocrine function? Exerc Immunol Rev 10: 42–55

Pedersen BP, Hoffman-Goetz L (2000) Exercise and the immune system: regulation, integration, and adaptation. Physiol Rev 80: 1055–1081

Rohde T, Maclean DA, Ritcher EA, Kiens B, Pedersen BK (1997) Prolonged submaximal eccentric exercise is associated with increased levels of plasma IL-6. Am J Physiol 273: E85–E91

Santos RVT, Bassit RA, Caperuto EC, Costa Rosa LFBP (2004) The effect of creatine supplementation upon inflammatory and muscle soreness markers after a 30 km race. Life Sci 75: 1917–1924

Smith L, Miles MP (2000) Exercise induced muscle injury and inflammation. In: Garret WE, Kirkendall DT (eds) Exercise and sport science. Lippincott Williams & Willkins, Philadelphia, pp 401–413

Steel DM, Witehead AS (1994) The major acute phase reactant: C-reactive protein, serum amyloid P component and serum amyloid A protein. Immunol Today 15: 8

Terjung RL, Clarkson P, Eichner ER, et al (2000) The physiological and health effect of oral creatine supplementation. Med Sci Sport Exerc 32: 706–716

Thompson HS, Hyatt J-P, De Souza MJ, Clarkson PM (1997) The effects of oral contraceptives on delayed onset muscle soreness following exercise. Contraception 56: 59–65

Volek JS, Rawson ES (2004) Scientific basis and practical aspects of creatine supplementation for athletes. Nutrition 20: 609–614

Authors' address: Dr. Reinaldo Abunasser Bassit, Departamento de Biologia Celular, Instituto de Ciências Biomédicas I, Universidade de São Paulo, Av. Lineu Prestes 1524, Sala 105, 05508-900 Butantã, São Paulo, SP, Brasil,
Fax: 55-011-30917245, E-mail: tubaraousp@sti.com.br
