# Supplementary material for: Impact of creatine supplementation on inflammation: evidence from a systematic review and meta-analysis of randomized double-blind placebo trials
Source: Front Immunol. 2026 Feb 19;17:1743603. doi: 10.3389/fimmu.2026.1743603 (PMC12961398; doi:10.3389/fimmu.2026.1743603)
Supplement: Supplementary file 2 [file SupplementaryFile1.zip › SR Creatine inflammatory markers (Kell Doutorado). /Supplementary Files/Final References/Final/Rawson et al 2007.pdf]

MONTANA STATE UNIVERSITY LIBRARY

Creatine supplementation does not reduce muscle damage or enhance recovery from resistance exercise.

Authors: E.S. Rawson, M.P. Conti, and M.P. Miles

This is a postprint of an article that originally appeared in Journal of Strength and Conditioning Research on November 2007.

Rawson, ES, MP Conti, and MP Miles. Creatine supplementation does not reduce muscle damage or enhance recovery from resistance exercise. Journal of Strength and Conditioning Research, 21(4):1208-1213, 2007.
http://dx.doi.org/10.1519/00124278-200711000-00039

Made available through Montana State University's ScholarWorks
scholarworks.montana.edu

Creatine Supplementation Does not Reduce Muscle Damage or Enhance Recovery From Resistance Exercise

Eric S. Rawson,[1] Michael P. Conti,[1] and Mary P. Miles[2]

[1]*Department of Exercise Science and Athletics, Bloomsburg University, Bloomsburg, Pennsylvania 17815;*
[2]*Department of Health and Human Development, Montana State University, Bozeman, Montana 59717.*

Abstract. Rawson, E.S., M.P. Conti, and M.P. Miles. Creatine supplementation does not reduce muscle damage or enhance recovery from resistance exercise. *J. Strength Cond. Res. 21(4): 1208–1213. 2007.*—Previous studies have shown that creatine supplementation reduces muscle damage and inflammation following running but not following high-force, eccentric exercise. Although the mechanical strain placed on muscle fibers during high-force, eccentric exercise may be too overwhelming for creatine to exert any protective effect, creatine supplementation may protect skeletal muscle stressed by a resistance training challenge that is more hypoxic in nature. The purpose of this study was to examine the effects of short-term creatine supplementation on markers of muscle damage (i.e., strength, range of motion, muscle soreness, muscle serum protein activity, C-reactive protein) to determine whether creatine supplementation offers protective effects on skeletal muscle following a hypoxic resistance exercise test. Twenty-two healthy, weight-trained men (19–27 years) ingested either creatine or a placebo for 10 days. Following 5 days of supplementation, subjects performed a squat exercise protocol (5 sets of 15–20 repetitions at 50% of 1 repetition maximum [1RM]). Assessments of creatine kinase (CK) and lactate dehydrogenase activity, high-sensitivity C-reactive protein, maximal strength, range of motion (ROM), and muscle soreness (SOR) with movement and palpation were conducted pre-exercise and during a 5-day follow up. Following the exercise test, maximal strength and ROM decreased, whereas SOR and CK increased. Creatine and placebo-supplemented subjects experienced significant decreases in maximal strength (creatine: 13.4 kg, placebo: 17.5 kg) and ROM (creatine: 2.4°, placebo: 3.0°) immediately postexercise, with no difference between groups. Following the exercise test, there were significant increases in SOR with movement and palpation ($p < 0.05$ at 24, 48, and 72 hours postexercise), and CK activity ($p < 0.05$ at 24 and 48 hours postexercise), with no differences between groups at any time. These data suggest that oral creatine supplementation does not reduce skeletal muscle damage or enhance recovery following a hypoxic resistance exercise challenge.

Introduction

Anecdotal reports of increased muscle dysfunction (i.e., cramps, muscle strains, etc.) in creatine users have been discussed, but only 1 published case study has reported severe muscle dysfunction (compartment syndrome and rhabdomyolysis) in a creatine user (27). Muscle cramping in creatine users has been reported, but these studies did not include placebo or noncreatine user groups for comparison (14, 18). In contrast to the anec-dotal reports, Greenwood and colleagues reported either similar (16, 17) or fewer (15) instances of muscle dysfunction (cramping, muscle tightness, strains, injuries, etc.) between creatine and noncreatine users. Additionally, survey data (15, 32) and anecdotal reports (7) indicate that exogenous creatine and phosphocreatine decrease muscle soreness and increase recovery between workouts. Thus, the effects of creatine on muscle function are unclear.

In theory, increased muscle phosphocreatine levels resulting from creatine supplementation may reduce muscle dysfunction because it is known that exogenous phosphocreatine reduces muscle damage in cardiac tissue (28, 29). Phosphocreatine binds to the polar phospholipid heads of the cardiac muscle membrane, stabilizes the membrane phospholipid bilayer, decreases membrane fluidity, and turns the membrane into a more ordered state (29). This subsequently decreases the loss of cardiac muscle proteins, indicating less cytoplasmic leakage and potentially less muscle tissue damage (29). In fact, phosphocreatine is used as a cardio-protective agent during heart surgery and to reduce infarct size after myocardial infarction (28, 29).

Previously, Rawson et al. (25) examined the effects of creatine supplementation on exercise-induced muscle damage. Twenty-three untrained individuals were supplemented with creatine (20 g per day) or placebo for 5 days. Following the supplementation period, subjects performed 50 maximal eccentric contractions of the elbow flexors, and criterion measures (force production, range of motion, circumference, soreness, and muscle serum proteins) were assessed pre-exercise and for 5 days after exercise. Eccentric exercise damages skeletal muscle and sarcoplasmic reticulum membranes (19, 38), resulting in calcium dysregulation and muscle dysfunction (decreased strength, decreased range of motion, increased muscle soreness, and increased levels of muscle serum proteins) (9, 10). Although both creatine and placebo groups experienced a significant loss of maximal force production and range of motion, a significant increase in circumference of the biceps, soreness, and serum creatine kinase and lactate dehydrogenase, there were no differences between groups at any time. These findings are supported by Warren et al. (36) who examined the effects of creatine supplementation on exercise-induced muscle damage using an animal model. In contrast to data from Rawson et al. (25), Santos and colleagues (30) reported that creatine supplementation attenuated the postexercise increase in creatine kinase (by 19%), prostaglandin E_2 (by 61%), and tumor necrosis factor-α (by 34%) and eliminated the increase in lactate dehydrogenase in trained runners following a 30-km run. The authors concluded that creatine supplementation reduced muscle damage and inflammation following a stressful running task.

It is unclear why the results of Rawson et al. (25) and

TABLE 1. Descriptive characteristics (mean ± *SD*) for subjects randomized to receive either creatine or placebo supplementation. There were no differences between creatine- and placebo-supplemented subjects before supplementation.

	Creatine	Placebo	*p* value
Age (y)	22.2 ± 1.3	22.1 ± 2.5	$p = 0.94$
Height (cm)	176.4 ± 6.3	177.2 ± 5.7	$p = 0.75$
Body mass (kg)	88.4 ± 11.5	83.4 ± 8.2	$p = 0.23$
Training experience (y)	6.4 ± 3.3	5.8 ± 3.1	$p = 0.66$
1RM* squat strength (kg)	137.8 ± 24.7	126.1 ± 30.2	$p = 0.22$
Range of motion (°)	52.6 ± 6.3	52.2 ± 5.2	$p = 0.88$
Creatine kinase ($IU \cdot L^{-1}$)	297.6 ± 211.7	286.0 ± 258.6	$p = 0.86$
Lactate dehydrogenase ($IU \cdot L^{-1}$)	147.5 ± 19.0	155.9 ± 24.3	$p = 0.37$
C-Reactive Protein ($mg \cdot L^{-1}$)	1.2 ± 1.3	1.9 ± 3.6	$p = 0.80$

* 1RM = 1 repetition maximum.

Santos et al. (30) are discrepant or why creatine supplementation appears to reduce muscle damage and inflammation following running (30) but not following high-force, eccentric exercise (25, 36). Potentially, the mechanical strain placed on muscle fibers from a high-force, eccentric laboratory exercise test is too overwhelming for creatine to exert any protective effect (23). However, creatine may preserve energy metabolism under hypoxic conditions, which may also reduce tissue damage. For instance, Wilken et al. (37) and Adcock et al. (1) reported that exogenous creatine maintained higher adenosine triphosphate (ATP) and phosphocreatine levels in nervous tissue after 30 and 100 minutes of hypoxia, respectively, when compared with controls. Additionally, creatine-supplemented animals show improved cerebral reperfusion and reduced infarct size following stroke (22).

Because there are data to suggest that athletes ingesting creatine have fewer instance of muscle dysfunction (15), it is possible that creatine supplementation exerts protective effects on skeletal muscle when stressed by an unaccustomed hypoxic exercise training test. The purpose of this study was to examine the effects of short-term creatine supplementation on indirect markers of muscle damage (i.e., strength, range of motion, muscle soreness, muscle serum protein activity, C-reactive protein) to determine whether creatine supplementation offers protective effects on skeletal muscle following a hypoxic resistance exercise challenge.

METHODS

Experimental Approach to the Problem

This study was conducted in the Department of Exercise Science and Athletics at Bloomsburg University. On visit 1, a blood sample was drawn, and maximal strength, range of motion, and muscle soreness were assessed. Participants then ingested high doses of either creatine or placebo supplements for 5 days (loading phase) followed by a 5-day low-dose supplementation phase (maintenance phase). Following the loading phase, participants completed a standardized exercise test designed to induce muscle tissue disruption without severe muscle damage (35). A reassessment of maximal strength and range of motion was conducted immediately postexercise. Assessments of range of motion, soreness, and a blood draw were taken once per day on each of the following 5 days. Maximal strength was reassessed 5 days postexercise.

Subjects

Twenty-two resistance-trained men recruited from the Bloomsburg, PA, area completed the study. All participants completed a physical activity readiness questionnaire (PAR-Q) and signed an informed consent document approved by the Bloomsburg University Institutional Review Board before participation in this study. Subjects were instructed to maintain similar habitual physical activity and dietary behaviors but to refrain from resistance exercise for the duration of the study. Individuals who had previously ingested creatine supplements were permitted to participate in this investigation if they had not ingested creatine within the previous 6 weeks. Descriptive characteristics of the subjects are presented in Table 1.

Because of gender differences in resting muscle creatine levels (13), potential differences in the response of females to creatine supplementation (21, 33), gender differences in the response to stressful exercise (8), and in an effort to study the most homogenous population, only male subjects were studied. Subjects were recreationally trained and participated in a resistance exercise program that included the squat exercise. Although weight training experience is contradictory for participation in an exercise-induced muscle damage study because of the repeated bout effect, Volek et al. (35) demonstrated increased muscle serum proteins and muscle soreness in this population following the exercise stress protocol outlined in the current study.

Supplementation

Participants were randomly placed into either a creatine or placebo group, and supplements were administered in a double-blind, placebo-controlled manner. During the loading phase, each participant ingested 0.3 $g \cdot kg^{-1}$ of body weight per day of encapsulated creatine (NutraSense Company, Shawnee Mission, KS) or placebo for 5 days. Participants were instructed to ingest the supplements with food in 3 equal doses per day. This supplementation protocol has been previously shown to significantly increase muscle creatine levels (34). Following the loading phase, participants ingested 0.03 $g \cdot kg^{-1}$ of body weight per day of creatine or placebo for 5 days to maintain increased muscle creatine levels.

Muscle Function and Soreness Tests

Each participant's 1 repetition maximum (1RM) in a squat exercise performed on a Smith Machine (Body Masters Sports Industries Inc., Rayne, LA) was determined using standard procedures (5). Range of motion about the knee was assessed with a goniometer (Lafayette Instrument, Lafayette, IN) while the subjects lay prone and attempted to fully flex the knee. The malleolus, lateral epicondyle, and greater trochanter were marked with semipermanent marker to ensure consistent measurements.

Perception of muscle soreness was assessed using a 100-mm-linear visual analog scale, where 0 mm represents no pain, and 100 mm represents very painful. Soreness was evaluated during movement (body weight squat) and palpation.

Blood Samples

Blood samples were taken before supplementation and every 24 hours postexercise. Venous blood samples were taken from the antecubital vein, and serum creatine kinase and lactate dehydrogenase were measured colorimetrically on an automated analyzer (Roche Modular Triple P, Roche, Indianapolis, IN) at Geisinger Medical Center in Danville, PA. Serum C-reactive protein concentrations were determined using a high-sensitivity enzyme immunoassay (EIA) kit (MP Biomedicals, Irvine, CA) with a sensitivity of 0.1 mg·L^{-1}. Spectrophotometric measurements were made using a μQuant Universal microplate spectrophotometer (Bio-Tek Instruments, Winooski, VT).

Exercise Protocol

Subjects completed a standardized warm-up (5 minutes of cycling) and then performed 5 sets of 15–20 repetitions of a Smith Machine squat exercise at 50% of their previously determined 1RM squat, with 2 minutes recovery between each set. Volek and colleagues (35) used this exercise test to induce muscle tissue disruption, but not severe damage, in recreationally weight-trained men. This exercise test results in transiently increased blood creatine kinase and muscle soreness (35) that are lower than values reported following high-force, eccentric exercise tests (25).

Statistical Analyses

Sample size estimation was conducted using the methods of Cohen (11), assuming a power (1-β) of 0.80, $\alpha = 0.05$, and based on the increase in creatine kinase reported by Volek et al. (35). It was determined that 10 subjects in each group would be necessary to locate a difference in creatine kinase between creatine- and placebo-supplemented groups. Data were tested for normality using a Kolmogorov-Smirnov test, and variables that deviated from normality (creatine kinase and C-reactive protein) were log-transformed before analysis. A repeated-measures analysis of variance (ANOVA) with a grouping factor was used to assess the pattern of change between groups from pre- to postsupplementation (group × time interaction term). Tukey's post hoc tests were used to locate differences when ANOVA revealed a significant interaction. Significance was set at $p \leq 0.05$.

Results

Descriptive characteristics and baseline measures of outcome variables are described in Table 1. There were no differences in age, height, body mass, years of training experience, maximal squat strength, range of motion, creatine kinase, lactate dehydrogenase, or C-reactive protein between creatine and placebo groups (Table 1).

Muscle Function and Soreness

Squat strength significantly decreased in both groups immediately following the exercise test ($p < 0.001$) (creatine group: −9.7%; placebo group: −13.9%) but was not different from presupplementation values 5 days after the exercise test. There was no difference in squat strength between creatine and placebo groups at any time (group × time interaction, $p = 0.64$) (Figure 1). Knee angle significantly increased immediately postexercise test ($p < 0.001$) (creatine group: +4.6% placebo group +5.7%), indicating that range of motion about the knee decreased. Range of motion was not different from presupplementation values from 24 to 120 hours postexercise, and there were no differences in range of motion between creatine and placebo groups at any time (group × time interaction, $p = 0.14$) (Figure 2). Muscle soreness during the body weight–only squat significantly increased from pre-exercise values at 24, 48, and 72 hours postexercise (all $p < 0.001$), with no difference between creatine and placebo groups at any time (group × time interaction, $p = 0.20$) (Figure 3). Muscle soreness with palpation significantly

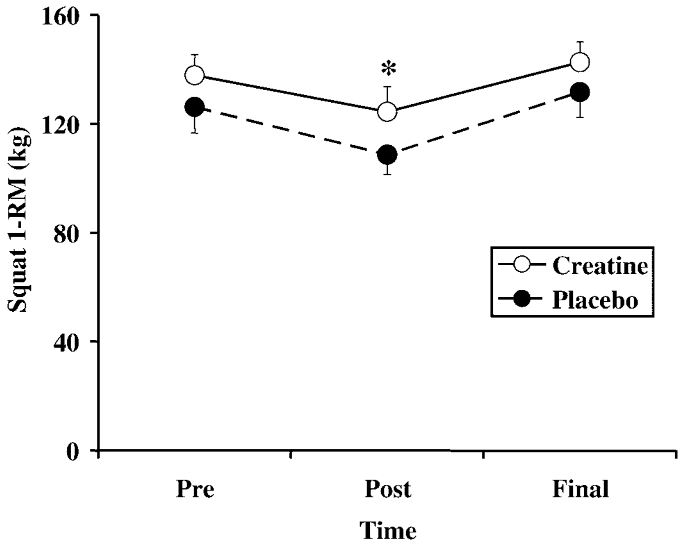

Figure 1. One repetition maximum (1RM) in the squat. Pre = presupplementation; post = immediately postexercise test and following 5 days of creatine (0.3 g·kg^{-1}·d^{-1}) or placebo supplementation; final = 120 hours postexercise test and following 5 days of creatine (0.03 g·kg^{-1}·d^{-1}) or placebo supplementation; * Significantly different from pre value (main effect, $p < 0.001$; group × time, $p = 0.64$).

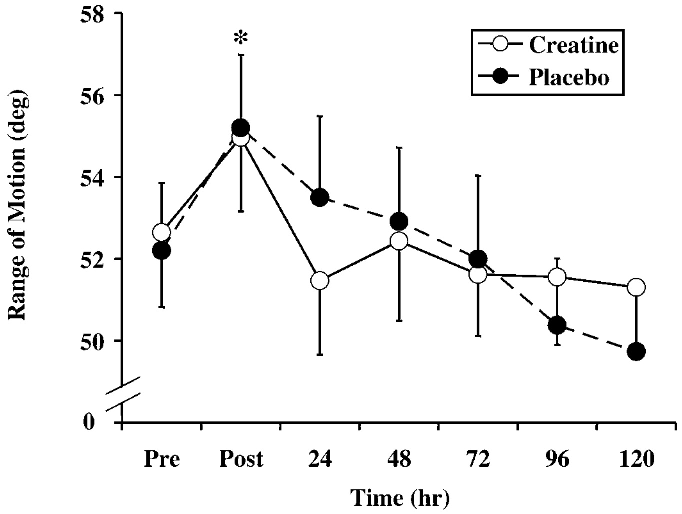

Figure 2. Range of motion. Pre = presupplementation; post = immediately postexercise; 24, 48, 72, 96, and 120 denote time in hours postexercise test. * Significantly different from pre value (main effect, $p < 0.001$; group × time, $p = 0.14$).

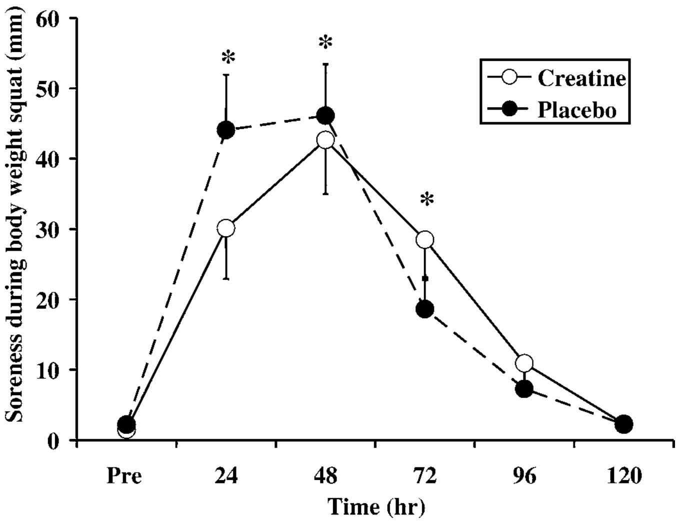

FIGURE 3. Soreness during a body weight squat. Pre = presupplementation; 24, 48, 72, 96, and 120 denote time in hours postexercise test. * Significantly different from pre value (main effect, $p < 0.001$; group × time, $p = 0.20$).

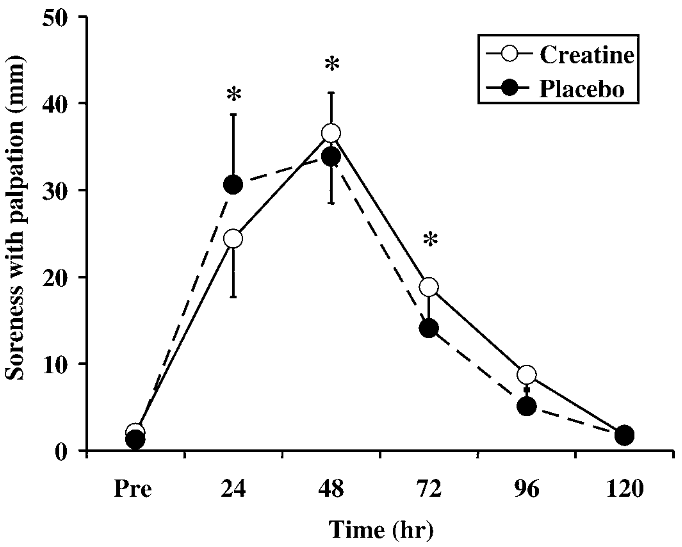

FIGURE 4. Soreness with palpation. Pre = presupplementation; 24, 48, 72, 96, and 120 denote time in hours postexercise test. * Significantly different from pre value (main effect, $p < 0.001$; group × time, $p = 0.77$).

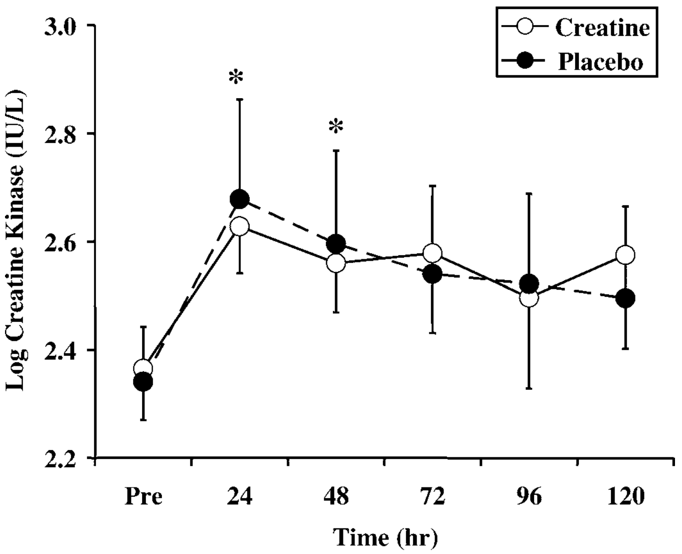

FIGURE 5. Log creatine kinase. Pre = presupplementation; 24, 48, 72, 96, and 120 denote time in hours postexercise test. * Significantly different from pre value (main effect, $p < 0.01$; group × time, $p = 0.95$).

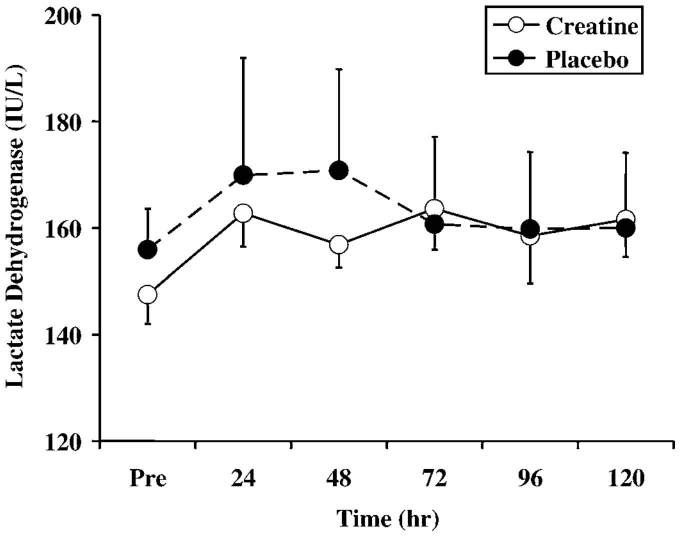

FIGURE 6. Lactate dehydrogenase. Pre = presupplementation; 24, 48, 72, 96, and 120 denote time in hours postexercise test.

increased from pre-exercise at 24 ($p < 0.001$), 48 ($p < 0.001$), and 72 hours ($p < 0.01$) postexercise. There was no difference in muscle soreness with palpation between groups at any time (group × time interaction, $p = 0.77$) (Figure 4).

Blood Assessments

Creatine kinase was significantly increased from baseline at 24 ($p < 0.01$) and 48 hours ($p = 0.05$) postexercise, but there were no differences in creatine kinase between creatine or placebo groups at any time (Figure 5) (group × time interaction, $p = 0.95$). Lactate dehydrogenase and C-reactive protein did not increase following the exercise test (Figures 6 and 7).

DISCUSSION

Creatine monohydrate is one of the most popular dietary supplements used by athletes, with annual sales exceeding several hundred million dollars and a high prevalence of use across many sports. Survey research supports that the prevalence of creatine use is as high as 17% in high school student–athletes, 30% in high school football players, 41% in National Collegiate Athletic Association student–athletes, 57% in health club members, and 74% in power sport athletes (reviewed in 24, 26).

Muscle damage/dysfunction caused by strenuous exercise may negatively affect exercise performance by decreasing economy, impairing glycogen repletion, altering biomechanical execution, and decreasing strength (31), which may increase risk of injury (12). Although anecdotal reports and a single case study (27) imply that creatine supplementation may cause muscle dysfunction, the results of open label studies (15–17), clinical trials (25, 30), and animal research (36) do not support this contention. In fact, evidence continues to accumulate that creatine does not promote, and may even reduce, muscle dys-

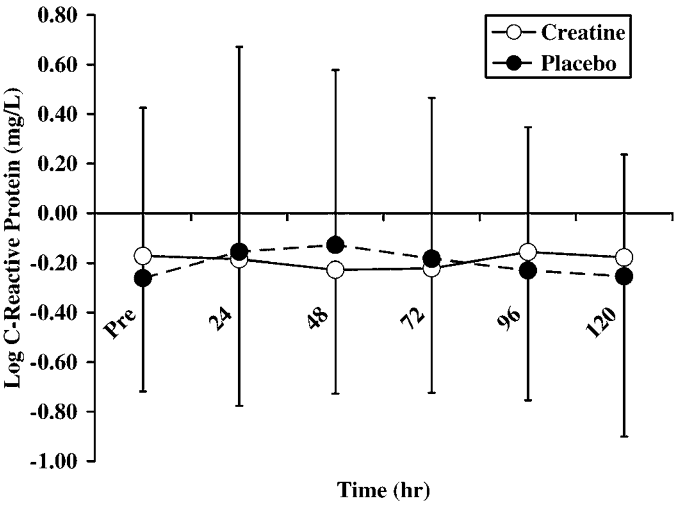

FIGURE 7. Log high-sensitivity C-reactive protein. Pre = presupplementation; 24, 48, 72, 96, and 120 denote time in hours postexercise test.

function. With that in mind, and given the large number of athletes ingesting creatine, the purpose of this study was to further investigate the interactions between creatine supplementation and muscle function.

We examined the effects of short-term creatine supplementation on indirect markers of exercise-induced muscle damage (i.e., strength, range of motion, muscle soreness, muscle serum protein activity, C-reactive protein) to determine whether creatine supplementation offers protective effects on skeletal muscle following resistance exercise. Athletes report decreased muscle soreness (7, 32) and faster recovery between workouts (15) following exogenous creatine or phosphocreatine administration, indicating that creatine may be useful as a recuperative aid. Reportedly, creatine supplementation attenuates muscle damage following stressful running (30) but not following high-force, eccentric exercise (25, 36); thus, the data are discrepant. Potentially, creatine may protect skeletal muscle tissue from hypoxic, but not mechanically induced, damage. Previous studies demonstrated higher ATP and phosphocreatine tissue levels (1, 37) improved cerebral reperfusion and reduced infarct size (22) following a hypoxic challenge in creatine-treated animals vs. controls. Also, Arciero et al. (4) demonstrated improved limb blood flow following creatine supplementation and resistance training compared with resistance training alone. Thus, it is possible that creatine preserves energy metabolism or improve blood flow during a hypoxic exercise challenge and subsequently reduce tissue damage.

In this study, contrary to our hypothesis, creatine supplementation did not reduce muscle damage or enhance recovery from a hypoxic resistance exercise challenge. Subjects in both creatine- and placebo-supplemented groups experienced significant decreases in muscle strength and range of motion and increases in muscle soreness and muscle serum proteins, with no differences between groups. Rawson et al. (25) previously hypothesized that high-force, eccentric exercise produces such severe muscle damage that any protective effects of creatine would be overwhelmed. Thus, a goal of this study was to expose participants to a hypoxic exercise test that induced muscle tissue disruption but not severe muscle damage. In the current study, the postexercise reduction in force production (≈12 vs. 47%) and range of motion (≈5 vs. 20%) and peak increase in creatine kinase (1,173 vs. 3,816 $IU \cdot L^{-1}$), muscle soreness with movement (≈44 vs. 50 mm), and muscle soreness with palpation (≈35 vs. 48 mm) were smaller than in the previous study that used high-force, eccentric actions. Thus, we believe we were successful in causing less muscle damage with the hypoxic squat protocol relative to high-force, eccentric exercise.

In contrast to data from the current study are the data presented by Santos et al. (30), which demonstrate a protective effect of creatine on skeletal muscle. Following a 30-km run, creatine-supplemented subjects experienced less muscle damage, as indicated by a blunted increase in creatine kinase (19% < placebo), prostaglandin E_2 (61% < placebo), and tumor necrosis factor-α (34% < placebo) and no increase in lactate dehydrogenase. Data from Santos et al. (30) and Rawson et al. (25) are difficult to compare because different populations (endurance vs. resistance trained), different exercise tests (running vs. resistance exercise), different time courses of postexercise blood measurements (24 hours postexercise vs. 24–120 hours postexercise), and different inflammatory markers (prostaglandin E_2 and tumor necrosis factor-α vs. C-reactive protein) were measured and studied. Additionally, Rawson et al. (25) assessed changes in strength, range of motion, and muscle soreness, whereas Santos et al. (30) did not.

It is possible that the resistance exercise used in the current study induces muscle damage through a different mechanism than a running protocol. For instance, Bloomer et al. (6) compared oxidative stress following 30 minutes of cycling (70% of $\dot{V}O_2max$) or dumbbell squatting (70% 1RM). In this study, which attempted to match for muscle group, exercise time, and intensity, protein carbonyl values following the resistance exercise was 1.8 times higher 24 hours postexercise than following the cycling test. Previous studies comparing the effects of aerobic and anaerobic exercise have demonstrated greater lipid peroxidation following anaerobic exercise tests (2, 3, 20), suggesting that aerobic and resistance exercises may damage muscles through different mechanisms.

In summary, these data suggest that oral creatine supplementation does not reduce skeletal muscle damage or enhance recovery following low-intensity, high-repetition resistance exercise. Collectively, the available studies show a protective effect of creatine on muscle function following stressful running (30) but not following high-repetition resistance (current study) or high-force, eccentric exercise (25); thus, the data are discrepant. Further studies of the interactive effects of creatine supplementation and exercise on muscle function are needed.

Practical Applications

Data from the current study can be applied to resistance-trained athletes undergoing high-repetition resistance training. Creatine supplementation did not decrease muscle damage or enhance recovery from a stressful bout of resistance exercise. The current study, combined with the previous investigations of the interactions between creatine supplementation, extreme exercise stress, and muscle function (25, 30, 36), do not clearly indicate that creatine exerts a protective effect on skeletal muscle. However, these studies do indicate that, even under extreme exercise conditions, creatine supplementation does not exacerbate muscle dysfunction. Clinicians, researchers, strength and conditioning professionals, and athletes should recognize that few studies have the statistical

power to detect severe adverse events (i.e., severe rhabdomyolysis), which may occur at a rate of 1 in 10,000 exposures. The results of the current study cannot be generalized to athletes ingesting creatine for extended periods, to those ingesting creatine in doses above what is recommended, or to athletes engaged in resistance training with an exaggerated eccentric component or plyometrics. The results of the current study suggest that creatine supplementation neither decreases muscle damage nor enhances recovery from a hypoxic resistance exercise challenge.

References

1. Adcock, K.H., J. Nedelcu, T. Loenneker, E. Martin, T. Wallimann, and B.P. Wagner. Neuroprotection of creatine supplementation in neonatal rats with transient cerebral hypoxia-ischemia. *Dev. Neurosci.* 24: 382–388. 2002.
2. Alessio, H.M., A.H. Goldfarb, and R.G. Cutler. MDA content increases in fast- and slow-twitch skeletal muscle with intensity of exercise in a rat. *Am. J. Physiol.* 255:C874–877. 1988.
3. Alessio, H.M., A.E. Hagerman, B.K. Fulkerson, J. Ambrose, R.E. Rice, and R. L. Wiley. Generation of reactive oxygen species after exhaustive aerobic and isometric exercise. *Med. Sci. Sports Exerc.* 32:1576–1581. 2000.
4. Arciero, P.J., N.S. Hannibal, 3rd, B.C. Nindl, C.L. Gentile, J. Hamed, and M.D. Vukovich. Comparison of creatine ingestion and resistance training on energy expenditure and limb blood flow. *Metabolism* 50: 1429–1434. 2001.
5. Baechle, T.R., and R.W. Earle, eds. *Essentials of Strength Training and Conditioning* (2nd ed.). Champaign, IL: Human Kinetics, 2000.
6. Bloomer, R.J., A.H. Goldfarb, L. Wideman, M.J. McKenzie, and L.A. Consitt. Effects of acute aerobic and anaerobic exercise on blood markers of oxidative stress. *J. Strength Cond. Res.* 19:276–285. 2005.
7. Clark, J.F. Uses of creatine phosphate and creatine supplementation for the athlete. In: *Creatine and Creatine Phosphate: Scientific and Clinical Perspectives.* M.A. Conway and J.F. Clark, eds. San Diego: Academic Press, 1996.
8. Clarkson, P.M., and M.J. Hubal. Are women less susceptible to exercise-induced muscle damage? *Curr. Opin. Clin. Nutr. Metab. Care* 4:527–531. 2001.
9. Clarkson, P.M., K. Nosaka, and B. Braun. Muscle function after exercise-induced muscle damage and rapid adaptation. *Med. Sci. Sports Exerc.* 24:512–520. 1992.
10. Clarkson, P.M., and I. Tremblay. Exercise-induced muscle damage, repair, and adaptation in humans. *J. Appl. Physiol.* 65:1–6. 1988.
11. Cohen, J. *Statistical Power Analysis for the Behavioral Sciences* (2nd ed.). Hillsdale: Lawrence Erlbaum Associates, 1988.
12. Ebbeling, C.B., and P.M. Clarkson. Exercise-induced muscle damage and adaptation. *Sports Med.* 7:207–234. 1989.
13. Forsberg, A.M., E. Nilsson, J. Werneman, J. Bergström, and E. Hultman. Muscle composition in relation to age and sex. *Clin. Sci.* 81: 249–256. 1991.
14. Greenwood, M., J. Farris, R. Kreider, L. Greenwood, and A. Byars. Creatine supplementation patterns and perceived effects in select division I collegiate athletes. *Clin. J. Sport Med.* 10:191–194. 2000.
15. Greenwood, M., R.B. Kreider, L. Greenwood, and A. Byars. Cramping and injury incidence in collegiate football players are reduced by creatine supplementation. *J. Athletic Train.* 38:216–219. 2003.
16. Greenwood, M., R.B. Kreider, L. Greenwood, D.S. Willoughby, and A. Byars. The effects of creatine supplementation on cramping and injury occurrence during college baseball training and competition. *J. Ex. Physiol. Online* 6:16–23. 2003.
17. Greenwood, M., R.B. Kreider, C. Melton, C. Rasmussen, S. Lancaster, E. Cantler, P. Milnor, and A. Almada. Creatine supplementation during college football training does not increase the incidence of cramping or injury. *Mol. Cell Biochem.* 244:83–88. 2003.
18. Juhn, M.S., J.W. O'Kane, and D.M. Vinci. Oral creatine supplementation in male collegiate athletes: a survey of dosing habits and side effects. *J. Am. Diet Assoc.* 99:593–595. 1999.
19. Lieber, R.L., L. Thornell, and J. Friden. Muscle cytoskeletal disruption occurs within the first 15 min of cyclic eccentric contractions. *J. Appl. Physiol.* 80:278–284. 1996.
20. Marzatico, F., O. Pansarasa, L. Bertorelli, L. Somenzini, and G. Della Valle. Blood free radical antioxidant enzymes and lipid peroxides following long-distance and lactacidemic performances in highly trained aerobic and sprint athletes. *J. Sports Med. Phys. Fitness* 37:235–239. 1997.
21. Parise, G., S. Mihic, D. MacLennan, K.E. Yarasheski, and M.A. Tarnopolsky. Effects of acute creatine monohydrate supplementation on leucine kinetics and mixed-muscle protein synthesis. *J. Appl Physiol.* 91: 1041–1047. 2001.
22. Prass, K., G. Royl, U. Lindauer, D. Freyer, D. Megow, U. Dirnagl, G. Stockler-Ipsiroglu, T. Wallimann, and J. Priller. Improved reperfusion and neuroprotection by creatine in a mouse model of stroke. *J. Cereb. Blood Flow Metab.* 2006.
23. Proske, U. and T.J. Allen. Damage to skeletal muscle from eccentric exercise. *Exerc. Sport Sci. Rev.* 33:98–104. 2005.
24. Rawson, E.S., and P.M. Clarkson. Scientifically debatable: Is creatine worth its weight? *Gatorade Sports Sci. Exchange* 16:1–6. 2004.
25. Rawson, E.S., B. Gunn, and P.M. Clarkson. The effects of creatine supplementation on exercise-induced muscle damage. *J. Strength Cond. Res.* 15:178–184. 2001.
26. Rawson, E.S., and J.S. Volek. Effects of creatine supplementation and resistance training on muscle strength and weightlifting performance. *J. Strength Cond. Res.* 17:822–831. 2003.
27. Robinson, S.J. Acute quadriceps compartment syndrome and rhabdomyolysis in a weight lifter using high-dose creatine supplementation. *J. Am. Board Fam. Pract.* 13:134–137. 2000.
28. Saks, V.A., V. Stepanov, I.V. Jaliashvili, E.A. Konorev, S.A. Kryzkanovsky, and E. Strumia. Molecular and cellular mechanisms of action for the cardioprotective and therapeutic role of creatine phosphate. In: *Creatine and Creatine Phosphate: Scientific and Clinical Perspectives.* M.A. Conway and J.F. Clark, eds. San Diego: Academic Press, 1996.
29. Saks, V.A., and E. Strumia. Phosphocreatine: Molecular and cellular aspects of the mechanism of cardioprotective action. *Curr. Ther. Res.* 53: 565–598. 1993.
30. Santos, R.V., R.A. Bassit, E.C. Caperuto, and L.F. Costa Rosa. The effect of creatine supplementation upon inflammatory and muscle soreness markers after a 30 km race. *Life Sci.* 75:1917–1924. 2004.
31. Smith, L.L. Causes of delayed onset muscle soreness and the impact on athletic performance: A review. *J. Appl. Sport Sci. Res.* 6:135–141. 1992.
32. Stanton, R., and G.A. Abt. Creatine monohydrate use among elite Australian power lifters. *J. Strength Cond. Res.* 14:322–327. 2000.
33. Tarnopolsky, M.A. Gender differences in metabolism; nutrition and supplements. *J. Sci. Med. Sport* 3:287–298. 2000.
34. Volek, J.S., N.D. Duncan, S.A. Mazzetti, R.S. Staron, M. Putukian, A.L. Gomez, D.R. Pearson, W.J. Fink, and W.J. Kraemer. Performance and muscle fiber adaptations to creatine supplementation and heavy resistance training. *Med. Sci. Sports Exerc.* 31:1147–1156. 1999.
35. Volek, J.S., W.J. Kraemer, M.R. Rubin, A.L. Gomez, N.A. Ratamess, and P. Gaynor. l-Carnitine l-tartrate supplementation favorably affects markers of recovery from exercise stress. *Am. J. Physiol. Endocrinol. Metab.* 282:E474–482. 2002.
36. Warren, G.L., J.M. Fennessy, and M.L. Millard-Stafford. Strength loss after eccentric contractions is unaffected by creatine supplementation. *J. Appl. Physiol.* 89:557–562. 2000.
37. Wilken, B., J.M. Ramirez, I. Probst, D.W. Richter, and F. Hanefeld. Anoxic ATP depletion in neonatal mice brainstem is prevented by creatine supplementation. *Arch. Dis. Child. Fetal Neonatal Ed.* 82:F224–227. 2000.
38. Yasuda, T., K. Sakamoto, K. Nosaka, M. Wada, and S. Katsuta. Loss of sarcoplasmic reticulum membrane integrity after eccentric exercise. *Acta Physiol. Scand.* 161:581–582. 1997.

Acknowledgments

The authors thank the research participants for their dedication. This study was supported in part by a Bloomsburg University Research and Disciplinary Grant (to E.S.R.), a National Strength and Conditioning Association (NSCA) Graduate Student Research Grant (to M.P.C.), a Gatorade Sports Science Institute Student Research Grant (to M.P.C.), a Bloomsburg University Graduate Student Thesis Award (to M.P.C.), and an American Heart Association Pacific Mountain Affiliate Grant (to M.P.M.). Creatine monohydrate was generously donated by the NutraSense Company (Shawnee Mission, KS). The results of the present study do not constitute an endorsement by the authors or the NSCA.
