# Supplementary material for: Impact of creatine supplementation on inflammation: evidence from a systematic review and meta-analysis of randomized double-blind placebo trials
Source: Front Immunol. 2026 Feb 19;17:1743603. doi: 10.3389/fimmu.2026.1743603 (PMC12961398; doi:10.3389/fimmu.2026.1743603)
Supplement: Supplementary file 2 [file SupplementaryFile1.zip › SR Creatine inflammatory markers (Kell Doutorado). /Supplementary Files/ROB 2/Fig ROB 2 Creatine inflammatory markers.pdf]

Intention-to-treat

Unique ID	Study ID	Weight	D1	D2	D3	D4	D5	Overall
Bassit et al 2008	NA	1	+	+	+	+	!	!
Cornisha & Peeler 2018	NA	1	+	+	-	+	!	-
Deldicque et al 2008	NA	1	+	+	+	+	!	!
Oliveira et al 2020	NA	1	+	+	-	+	+	-
Rawson et al 2007	NA	1	+	+	+	+	+	+
Santos et al 2004	NA	1	+	+	-	+	!	-
Taes et al 2004	NA	1	+	+	+	+	!	!
Tarnopolsky et al 2007	NA	1	+	+	+	+	+	+

+ Low risk
! Some concerns
- High risk

D1 Randomisation process
D2 Deviations from the intended interventions
D3 Missing outcome data
D4 Measurement of the outcome
D5 Selection of the reported result
