# Supplementary material for: Impact of creatine supplementation on inflammation: evidence from a systematic review and meta-analysis of randomized double-blind placebo trials
Source: Front Immunol. 2026 Feb 19;17:1743603. doi: 10.3389/fimmu.2026.1743603 (PMC12961398; doi:10.3389/fimmu.2026.1743603)
Supplement: Supplementary file 2 [file SupplementaryFile1.zip › SR Creatine inflammatory markers (Kell Doutorado). /Supplementary Files/Final References/Final/Oliveira et al 2020.pdf]

Clinical Nutrition ESPEN 38 (2020) 94–98

Contents lists available at ScienceDirect

Clinical Nutrition ESPEN

journal homepage: http://www.clinicalnutritionespen.com

Original article

Creatine supplementation does not promote additional effects on inflammation and insulin resistance in older adults: A pilot randomized, double-blind, placebo-controlled trial

Camila L.P. Oliveira [a], Barbara de Moura Mello Antunes [b], Aline Corado Gomes [c], Fábio Santos Lira [b], Gustavo Duarte Pimentel [c], Normand G. Boulé [d], João Felipe Mota [c, *]

[a] Human Nutrition Research Unit, Department of Agricultural, Food & Nutritional Science, University of Alberta, Edmonton, AB, T6G 2E1, Canada
[b] Exercise and Immunometabolism Research Group, Department of Physical Education, Sao Paulo State University, Presidente Prudente, SP, 19060-900, Brazil
[c] Clinical and Sports Nutrition Research Laboratory, Faculty of Nutrition, Goiás Federal University, 227 Street, Block 68, Setor Leste Universitario, Goiania, GO, 74.605-080, Brazil
[d] Faculty of Kinesiology, Sport, and Recreation, University of Alberta, Edmonton, AB, T6G 2H9, Canada

ARTICLE INFO

Article history:
Received 8 November 2019
Accepted 29 May 2020

Keywords:
Creatine
exercise
older adults
inflammation

SUMMARY

Background: A chronic, low-grade inflammation is commonly present in older adults and has been associated with the onset of age-related chronic diseases. Resistance training (RT) and creatine (CR) supplementation emerged as promising strategies to reduce circulating pro-inflammatory cytokines. This study aimed to investigate the effects of CR supplementation combined with RT on markers of inflammation and insulin resistance in community-dwelling older adults.
Methods: In a pilot randomized, double-blind, placebo-controlled trial, participants were allocated to one of the following groups: 1) Creatine supplementation and resistance training (CR + RT, n = 13); 2) Placebo and resistance training (PL + RT, n = 14). While engaged in a 12-week RT program, participants from CR + RT group received 5 g/day of CR monohydrate and participants from PL + RT group received the same dose of maltodextrin. At baseline and at week 12, blood samples were collected for glucose, insulin, adiponectin, leptin, interleukin 6, interleukin 10, monocyte chemo-attractant protein-1 and C-reactive protein analysis.
Results: After 12 weeks of intervention, there were no differences between groups in any of the variables analyzed. Monocyte chemoattractant protein-1 was reduced in both groups (CR + RT: −55.66 ± 48.93 pg/mL, $p < 0.01$, $dz = 1.13$; PL + RT: −46.52 ± 55.21 pg/mL, $p < 0.01$, $dz = 0.84$).
Conclusion: Resistance training, regardless of CR supplementation, decreased MCP-1 concentration in older adults.

© 2020 European Society for Clinical Nutrition and Metabolism. Published by Elsevier Ltd. All rights reserved.

Introduction

The world population has been experiencing a transformation in its age structure due to increased longevity and decreased birth rates [1]. For this reason, age-related consequences and its possible treatments have been the focus of researches worldwide. Recent evidence demonstrated that chronic low-grade inflammation is commonly present in older adults and is strongly associated with insulin resistance [2]. As a consequence, this metabolic milieu can lead to the development of chronic diseases, such as type 2 diabetes mellitus (T2D) and cardiovascular diseases [3]. Considering human's skeletal muscle is able to modulate inflammation and the major site of insulin-mediated glucose disposal, this body compartment plays an essential role in the metabolic alterations resultant from aging [4]. Consequently, strategies able to counteract the age-related loss of skeletal muscle mass and strength might

* Corresponding author. Clinical and Sports Nutrition Research Laboratory, Faculty of Nutrition, Federal University of Goias, Goiania, Goias, Brazil. 227 Street, block 68, Setor Leste Universitario, Goiania, GO, 74.605-080, Brazil. Fax: +55 (62) 3209 6270.
E-mail address: jfemota@gmail.com (J.F. Mota).

https://doi.org/10.1016/j.clnesp.2020.05.024
2405-4577/© 2020 European Society for Clinical Nutrition and Metabolism. Published by Elsevier Ltd. All rights reserved.

positively affect older adult's inflammatory status and insulin sensitivity.

Resistance training (RT) and creatine (CR) supplementation recently emerged as promising strategies to counteract the effects of aging on skeletal muscle [5]. In older adults, RT in isolation appears to have a systemic anti-inflammatory effect, increasing circulating levels of anti-inflammatory cytokines, such as interleukin (IL) 10, and reducing pro-inflammatory molecules, such as C-reactive protein (CRP), IL-6, and tumor necrosis factor alpha (TNF-α) [6,7]. Similarly, CR supplementation has been shown to reduce circulating pro-inflammatory cytokines resultant from exercise-induced muscle damage in adults [8] and from laboratory-induced acute and chronic inflammation in animals [9,10]. In addition to its anti-inflammatory effects, when combined, RT and CR supplementation have been shown to improve insulin sensitivity in diverse population groups [11]. In humans, it appears that these strategies improved participants' metabolic control possibly via an increase in glucose transporter type 4 (GLUT-4) recruitment to the sarcolemma [12].

To our knowledge, the therapeutic role of CR supplementation combined with RT on improving systemic biomarkers of inflammation and insulin resistance in older adults has not been explored. Therefore, this pilot randomized, double-blind, placebo-controlled trial aimed to investigate the effects of CR supplementation combined with RT on pro- and anti-inflammatory biomarkers and insulin resistance in community-dwelling older adults.

Materials and methods

Experimental design, participants' information and intervention protocol

This study was a part of a randomized, double-blind, placebo-controlled, parallel-group clinical trial, registered at ensaiosclinicos.gov.br (RBR-2shfhj) and approved by the Human Research Ethics Committee of the Federal University of Goias (840.317). This clinical trial was performed in accordance with the ethical standards as laid down in the 1964 Declaration of Helsinki and its later amendments or comparable ethical standards. Before participating in the study, all participants were informed of the procedures and potential risks involved in the investigation and provided written informed consent. Men and women aged 60 and 80 years were recruited. Major exclusion criteria included the use of any ergogenic supplement in the 6 months prior to study initiation; use of any medication that affect muscle growth or the ability to perform moderate to vigorous exercise during the study (e.g., statins, muscle relaxants, and anti-inflammatory drugs); presence of kidney, liver, and/or heart disease; adherence to a vegetarian, vegan, or restrictive dietary pattern; engagement in structured physical activity. Specific details regarding this study have been reported elsewhere [13].

Thirty-two healthy, non-athletic older adults were randomly assigned following a stratified randomization based on sex to one of the following groups: 1) CR supplementation combined with RT (CR + RT), or 2) Placebo combined with RT (PL+ RT). Participants were asked to consume CR (5 g/day) or maltodextrin (5 g/day), while engaged in a 12-week supervised RT program. Considering the population group enrolled in this clinical trial, a more conservative supplementation protocol was chosen (i.e., 5 g/day of CR). This dosage was shown to saturate skeletal muscles with CR [14] and improve glycemic control in older adults diagnosed with T2D [12]. On training days, participants consumed the supplement immediately after the training sessions dissolved in a beverage comprised of 100 g of lemon-flavored maltodextrin. On non-training days, participants consumed the supplement immediately after lunch dissolved in one glass of liquid of their preference. The supplement packages were coded so neither the investigators nor the participants were aware of the contents until completion of the analysis. Participant's dietary intake was assessed at baseline and after 12 weeks of intervention with the use of a 3-day food record. Fat and lean masses were assessed by dual-energy X-ray absorptiometry (DXA), using a Lunar DPX NT densitometry (General Electric Medical Systems Lunar, Madison, EUA) with the enCORE 2011 software (version 13.60).

Both groups performed a 12-week supervised resistance training program, 3 times per week with 60 minutes of duration each session. Before the start of the training program, all participants underwent a familiarization period of 1 week. The volume of training progressed individually each session with increased weight × sets × repetitions. Each training session comprised of a 1) warm-up phase: 5–7 min of a light run on a stationary bicycle or 12 repetitions with 30% of the repetition maximum (RM) of the first exercise; 2) main phase: 3 sets of 13–15 of RM for the major muscle groups with 60 seconds of rest between sets, exercises for abdominal and lumbar regions, and 3 sets of 10–13 of RM for the other muscle groups with 60 seconds of rest between sets; and 3) final phase: stretching exercises.

Biochemical analysis

All biochemical analyses were done at baseline and after 12 weeks of intervention. Two days after the last day of intervention, participants were instructed to take their medications and attend the research unit in the morning after a 12-hour overnight fast. Blood samples were obtained from the antecubital vein. Immediately after collection, venous blood glucose was measured using a portable glucose meter (Accu-check, F. Hoffmann-La Roche Ltd, Switzerland). The remaining blood was inserted into an EDTA vacutainer and placed on ice before being centrifuged for 10 min at 4 °C and 3.500 rpm. Plasma was then pipetted into aliquot tubes and stored at −80 °C until analysis. Monocyte chemoattractant protein-1 (MCP-1), IL-6, IL-10, leptin and adiponectin were analyzed using the Human Quantikine ELISA kits (R&D Systems©), according to manufacturer's instructions and were determined in duplicates. C-reactive protein was analyzed by turbidimetric assay. Insulin concentration was determined using Insulin AccuBind ELISA Kits (Monobind Inc.) according to manufacturer's instructions. Homeostatic model assessment (HOMA) of β-cell function (%B) and insulin resistance (IR) were calculated using the HOMA2 Calculator (©Diabetes Trials Unit, University of Oxford, version 2.2.3). Intra- and inter-assay coefficients of variation (CV) were as follows: MCP-1:4.7 and 4.6, IL-6: 1.7 and 2.0, IL-10: 1.7 and 5.9, leptin: 3.0 and 4.2, adiponectin: 2.5 and 6.8, and insulin: 5.1 and 7.2, respectively.

Statistical analyses

A post hoc power analysis was conducted on MCP-1 from the CR + RT group (n = 11) using a two-tailed Wilcoxon matched pairs signed-rank test. With an effect size of 1.13 and type I error probability of 0.05, the estimated achieved power was 90%.

Data distribution was evaluated by Shapiro–Wilk W test. Variables that were not normally distributed were log-transformed for statistical analysis. Within-group differences (i.e., before and after intervention) were assessed by a paired samples t-test. Differences between groups were assessed by analysis of covariance (ANCOVA) with the baseline value as covariate and the change from baseline as the dependent variable. Potential confounders (covariates) that could affect biochemical measures and body composition were examined in the groups. Significant covariates were identified using multiple linear regression with backward elimination of those that were not significant. Effect sizes were calculated using Cohen's

dz formula and classified as small ($d = 0.2$), medium ($d = 0.5$), and large ($d = 0.8$) [15]. STATA version 12 (StataCorp, College Station, TX) and G*Power version 3.1.9.2, (Universität Düsseldorf, Germany) were used to perform all statistical analyses. Differences were regarded as statistically significant if $p < 0.05$.

Results

Fourteen participants from the PL + RT group (8 females and 6 males; age: 67 ± 6 years; body mass index: 27.3 ± 5.0 kg/m^2) and 13 from the CR + RT group (8 females and 5 males; age: 67 ± 5 years; body mass index: 27.1 ± 4.8 kg/m^2) completed the trial and were included in the analyses. Participants' baseline characteristics (Table 1) and dietary intake (i.e., energy, macronutrients, and calcium) have been reported elsewhere [13] and no differences between groups were observed.

Glycemic and inflammatory markers at baseline and after 12 weeks of intervention are presented in Table 2. There were no differences between groups in any of the variables analyzed. After 12 weeks of intervention, RT reduced MCP-1 concentration in both groups (CR + RT: −55.66 ± 48.93 pg/mL, $p < 0.01$, $dz = 1.13$; PL + RT: −46.52 ± 5 5.21 pg/mL, $p < 0.01$, $dz = 0.84$) (Fig. 1).

Discussion

The main finding of this pilot randomized, double-blind, placebo-controlled trial was that the addition of CR supplementation to 12 weeks of RT did not improve biomarkers of inflammation and insulin resistance in community-dwelling older adults. On the other hand, 12 weeks of RT, regardless of dietary intervention, decreased MCP-1 concentration in this population group.

In a randomized, double-blind, placebo-controlled design, Gualano, Novaes [16] observed no effect on fasting blood glucose, insulin and HOMA values in 22 young adults who underwent 12 weeks of aerobic exercise training combined with CR supplementation. Similarly, in this clinical trial, 12 weeks of CR supplementation combined with RT did not improve fasting blood glucose, insulin, and HOMA values in a cohort of community-dwelling older adults. In these two studies, the majority of participants had normal concentrations of blood glucose, which might have hindered a further improvement in glycemic control. On the other hand, the same group of researchers demonstrated a positive impact of 12 weeks of CR supplementation on glycemic control in 13 individuals diagnosed with T2D undergoing aerobic and resistance exercise training [12].

Monocyte chemoattractant protein-1 is a potent chemotactic factor for monocytes produced by several cell types in response to stimuli (e.g., oxidative stress, cytokines, or growth factors) or in a constitutive manner [17]. This chemokine has been associated with a state of low-grade chronic inflammation [17–19] and several age-related diseases, such as frailty [20], sarcopenic obesity [21], and cardiovascular diseases [22]. Jin, Lee [23] demonstrated that senescent cells secrete MCP-1, which is supported by increased levels of this chemokine with age [24–27]. A recent study reported that adults (21–50 years of age, n = 60) had mean MCP-1 concentration of 60 ± 0.6 pg/mL and older adults (70–92 years of age, n = 33) of 130 ± 1.5 pg/mL [27]. Considering the mean age of participants enrolled in this study was 67 years, the baseline values were in line with these studies. After 12 weeks of RT, participant's MCP-1 concentration dropped significantly and reached values similar to those observed in young adults (n = 10, 23 years of age; 27.6 ± 2.4 pg/mL) [24]. To our knowledge, only one study assessed the chronic effects of RT on blood MCP-1 concentration in 21 older adults and no difference was observed after 12 weeks of training [28]. Differences in the population group (i.e., women, 85 ± 4 years of age, diagnosed with diverse chronic diseases), exercise intensity (i.e., low intensity) and frequency (i.e., ~1 session/week) may explain the contradictory findings. Besides from MCP-1, this study failed to demonstrate a change in other systemic biomarkers of inflammation in older adults. There are several potential mechanisms of exercise-induced reduction in inflammation which might have influenced this study's results [6,7] and the most discussed one is the reduction in adipose tissue [7]. This body compartment is known to secrete pro-inflammatory cytokines that contribute to a state of low-grade chronic inflammation [29]. Considering that participants of this study did not experience a reduction in fat mass [13], it is possible that it might have influenced the absence of change in others systemic biomarkers of inflammation.

Creatine supplementation has been shown to improve inflammation induced by a half-ironman competition [30], a 30-km race [31], and an acute repeated-sprint exercise [32] in highly-trained adults. However, in this study, CR supplementation combined with RT did not improve IL-6, IL-10 and CRP in sedentary older adults. The mechanisms by which this compound exerts its anti-inflammatory properties is not fully understood [9] and might be distinct in different population groups and exercise modalities, which may explain, in part, the contradictory findings. Moreover, adiponectin and leptin analysis revealed no difference between groups and over time, which might be related to the absence of change in fat mass reported in the companion study to this paper [13,33]. Although the supplement dose utilized in this study has been shown to saturate skeletal muscles with CR (i.e., 5 g/day) [14], higher doses and longer periods of intervention might be needed to improve inflammation in older adults. Therefore, further research should be conducted to better understand the anti-inflammatory effects of different doses of CR supplementation combined or not with exercise training in older adults.

The placebo treatment chosen in this clinical trial (i.e., maltodextrin) is commonly used in studies assessing the effects of CR supplementation in humans [12,34,35], as it is very similar to CR in taste, texture, color, and appearance. Moreover, maltodextrin has been shown to exert little to no influence on the variables assessed [36,37], especially if ingested in low doses (i.e., 5 g/day). Therefore,

Table 1
Baseline characteristics of the study participants.

	CR + RT		PL + RT	
	Females (n = 8)	Males (n = 5)	Females (n = 8)	Males (n = 6)
Age (year)	64.4 ± 2.9	72.1 ± 2.4	66.3 ± 6.2	68.1 ± 7.0
Height (cm)	154.5 ± 6.8	164.4 ± 4.5	155.3 ± 5.5	165.2 ± 9.8
Body weight (kg)	66.4 ± 14.3	70.8 ± 14.0	68.7 ± 17.2	71.9 ± 18.8
Body mass index (kg/m^2)	27.8 ± 5.3	26.2 ± 4.8	28.2 ± 5.3	26.5 ± 9.0
Lean mass (kg)	33.8 ± 5.2	45.4 ± 6.6	36.4 ± 6.9	45.8 ± 7.9
Fat mass (kg)	28.5 ± 9.3	21.7 ± 9.7	29.5 ± 11.0	23.7 ± 10.5

A version of this table has been published elsewhere [13]. Data are expressed as mean ± standard deviation. No significant between-group differences were observed. Sex-differences were not evaluated due to low sample size. Abbreviations: CR + RT: creatine combined with resistance training; PL + RT: placebo combined with resistance training.

Table 2
Markers of inflammation and insulin resistance at baseline (Pre) and after 12 weeks of intervention (Post).

	CR + RT		P[1]	PL + RT		P[1]	P[2]
	Pre	Post		Pre	Post		
Glucose (mmol/L)	5.5 ± 1.0	5.1 ± 1.0	0.09	6.1 ± 1.4	5.8 ± 1.6	0.09	0.29
Insulin (uU/mL)[3]	9.2 ± 4.8	8.7 ± 5.7	0.42	12.3 ± 12.8	10.8 ± 9.6	0.45	0.74
HOMA %β[3]	88.0 ± 25.3	113.7 ± 72.7	0.46	94.9 ± 78.2	97.9 ± 78.8	0.62	0.63
HOMA IR[3]	1.2 ± 0.7	1.2 ± 0.7	0.40	1.6 ± 1.5	1.4 ± 1.2	0.39	0.81
Adiponectin (pg/mL)	11132.4 ± 4193.9	9453.7 ± 4967.2	0.37	8681.9 ± 4275.1	7563.8 ± 4664.6	0.35	0.92
Leptin (ng/mL)	525.4 ± 411.6	467.9 ± 418.3	0.67	684.0 ± 431.8	557.7 ± 401.9	0.10	0.67
IL-6 (pg/mL)	4.7 ± 2.9	4.1 ± 2.0	0.09	4.1 ± 1.3	4.3 ± 1.9	0.74	0.47
IL-10 (pg/mL)[3]	2.3 ± 1.3	1.9 ± 0.8	0.49	2.5 ± 1.3	2.8 ± 2.2	0.67	0.38
MCP-1 (pg/mL)[3]	78.8 ± 43.7	23.1 ± 18.6	<0.01	72.1 ± 68.4	25.6 ± 17.5	<0.01	0.43
CRP (mg/L)[3]	4.9 ± 4.9	2.9 ± 2.0	0.61	6.6 ± 7.4	3.3 ± 3.2	0.07	0.85

Data are expressed as mean ± standard deviation. [1]P values represent within-group differences from baseline to 12 weeks and were obtained with the use of paired-samples t-test. [2]P values represent between-group differences from baseline to 12 weeks and were obtained with the use of ANCOVA adjusted for baseline values. [3]Variables not normally distributed, according to Shapiro–Wilk W test, and log-transformed for statistical analysis. Abbreviations: CR + RT: creatine combined with resistance training; CRP: C-reactive protein; HOMA %β: homeostasis model assessment of β-cell function; HOMA IR: homeostasis model assessment of insulin resistance; IL-6: interleukin-6; IL-10: interleukin-10; MCP-1: monocyte chemoattractant protein-1; PL + RT: placebo combined with resistance training.

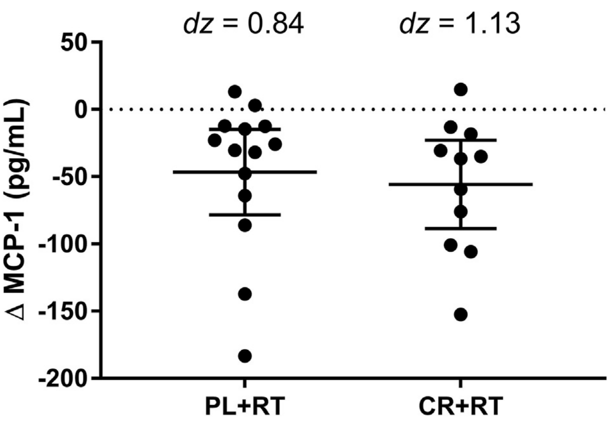

Fig. 1. Effects of resistance training combined with creatine or placebo on the change in MCP-1 in older adults. Data are mean ± standard deviation. Abbreviation: CR + RT: creatine combined with resistance training; *dz*: effect size; MCP-1: monocyte chemoattractant protein-1; PL + RT: placebo combined with resistance training.

results from this clinical trial are unlikely to have been influenced by the choice of placebo treatment. This study presents limitations as it is a secondary study, with a small sample size. Furthermore, more inflammatory biomarkers, markers of muscle biochemistry, and muscle damage could have been analyzed to assess the effects of RT. On the other hand, it was a well-controlled study regarding supplementation, diet, and exercise protocol.

In conclusion, CR supplementation combined with RT did not improve markers of inflammation and insulin resistance in community-dwelling older adults. On the other hand, 12 weeks of RT, regardless of dietary intervention, reduced MCP-1 concentration in this population group. Further research is needed to elucidate the potential mechanisms by which CR supplementation combined or not with RT act on markers of inflammation and insulin resistance in healthy and diseased older adults.

Funding

This research did not receive any specific grant from funding agencies in the public, commercial, or not-for-profit sectors.

Author contributions

The study was conceptualized by CLPO and JFM; data were collected and analyzed by CLPO, BMA, and ACG; data interpretation and manuscript preparation were undertaken by CLPO, FSL, GDP, NGB and JFM. All authors approved the final version of the paper.

Declaration of Competing Interest

None of the authors has any conflict in relation to the study.

Acknowledgements

We would like to thank MedNutrition for providing the supplements and the participants involved in this trial. JFM has been financially supported by the National Council for Scientific and Technological Development (CNPq, number 305082/2019-1).

References

[1] Fehlings MG, Tetreault L, Nater A, Choma T, Harrop J, Mroz T, et al. The aging of the global population: the changing epidemiology of disease and spinal disorders. Neurosurgery 2015;77(Suppl 4):S1–5.
[2] Cleasby ME, Jamieson PM, Atherton PJ. Insulin resistance and sarcopenia: mechanistic links between common co-morbidities. J Endocrinol 2016;229:R67–81.
[3] Shoelson SE, Lee J, Goldfine AB. Inflammation and insulin resistance. J Clin Invest 2006;116:1793–801.
[4] Dalle S, Rossmeislova L, Koppo K. The role of inflammation in age-related sarcopenia. Front Physiol 2017;8:1045.
[5] Chilibeck PD, Kaviani M, Candow DG, Zello GA. Effect of creatine supplementation during resistance training on lean tissue mass and muscular strength in older adults: a meta-analysis. Open Access J Sports Med 2017;8:213–26.
[6] Gleeson M, Bishop NC, Stensel DJ, Lindley MR, Mastana SS, Nimmo MA. The anti-inflammatory effects of exercise: mechanisms and implications for the prevention and treatment of disease. Nat Rev Immunol 2011;11:607.
[7] Woods JA, Wilund KR, Martin SA, Kistler BM. Exercise, inflammation and aging. Aging Dis 2011;3:130–40.
[8] Kim J, Lee J, Kim S, Yoon D, Kim J, Sung DJ. Role of creatine supplementation in exercise-induced muscle damage: a mini review. J Exer Rehab 2015;11:244–50.
[9] Riesberg LA, Weed SA, McDonald TL, Eckerson JM, Drescher KM. Beyond muscles: the untapped potential of creatine. Int Immunopharm 2016;37:31–42.
[10] Campos-Ferraz PL, Gualano B, das Neves W, Andrade IT, Hangai I, Pereira RT, et al. Exploratory studies of the potential anti-cancer effects of creatine. Amino acids; 2016.
[11] Pinto CL, Botelho PB, Pimentel GD, Campos-Ferraz PL, Mota JF. Creatine supplementation and glycemic control: a systematic review. Amino Acids 2016;48:2103–29.
[12] Gualano B, De Salles Painneli V, Roschel H, Artioli GG, Neves Jr M, De Sa Pinto AL, et al. Creatine in type 2 diabetes: a randomized, double-blind, placebo-controlled trial. Med Sci Sports Exerc 2011;43:770–8.
[13] Pinto CL, Botelho PB, Carneiro JA, Mota JF. Impact of creatine supplementation in combination with resistance training on lean mass in the elderly. J Cachexia Sarcopenia Muscle 2016;7:413–21.
[14] Hultman E, Soderlund K, Timmons JA, Cederblad G, Greenhaff PL. Muscle creatine loading in men. J Appl Physiol 1996;81:232–7.

[15] Cohen J. Statistical power analysis for the behavioral sciences. 2 ed. Hillsdale: Eribaum; 1988.
[16] Gualano B, Novaes RB, Artioli GG, Freire TO, Coelho DF, Scagliusi FB, et al. Effects of creatine supplementation on glucose tolerance and insulin sensitivity in sedentary healthy males undergoing aerobic training. Amino Acids 2008;34:245–50.
[17] Deshmane SL, Kremlev S, Amini S, Sawaya BE. Monocyte chemoattractant protein-1 (MCP-1): an overview. J Interferon Cytokine Res 2009;29:313–26.
[18] Conti P, DiGioacchino M. MCP-1 and RANTES are mediators of acute and chronic inflammation. Allergy Asthma Proc 2001;22:133–7.
[19] Franceschi C, Campisi J. Chronic inflammation (inflammaging) and its potential contribution to age-associated diseases. J Gerontol: Series A 2014;69:S4–9.
[20] Lu Y, Tan CT, Nyunt MS, Mok EW, Camous X, Kared H, et al. Inflammatory and immune markers associated with physical frailty syndrome: findings from Singapore longitudinal aging studies. Oncotarget 2016;7:28783–95.
[21] Lim JP, Leung BP, Ding YY, Tay L, Ismail NH, Yeo A, et al. Monocyte chemoattractant protein-1: a proinflammatory cytokine elevated in sarcopenic obesity. Clin Interv Aging 2015;10:605–9.
[22] Niu J, Kolattukudy PE. Role of MCP-1 in cardiovascular disease: molecular mechanisms and clinical implications. Clin Sci (Lond) 2009;117:95–109.
[23] Jin HJ, Lee HJ, Heo J, Lim J, Kim M, Kim MK, et al. Senescence-associated MCP-1 secretion is dependent on a decline in BMI1 in human mesenchymal stromal cells. Antioxidants Redox Signal 2016;24:471–85.
[24] Bartlett DB, Fox O, McNulty CL, Greenwood HL, Murphy L, Sapey E, et al. Habitual physical activity is associated with the maintenance of neutrophil migratory dynamics in healthy older adults. Brain Behav Immun 2016;56:12–20.
[25] Inadera H, Egashira K, Takemoto M, Ouchi Y, Matsushima K. Increase in circulating levels of monocyte chemoattractant protein-1 with aging. J Interferon Cytokine Res : Off J Int Soc Interferon Cytokine Res 1999;19:1179–82.
[26] Mansfield AS, Nevala WK, Dronca RS, Leontovich AA, Shuster L, Markovic SN. Normal ageing is associated with an increase in Th2 cells, MCP-1 (CCL1) and RANTES (CCL5), with differences in sCD40L and PDGF-AA between sexes. Clin Exp Immunol 2012;170:186–93.
[27] Valiathan R, Ashman M, Asthana D. Effects of ageing on the immune system: infants to elderly. Scand J Immunol 2016;83:255–66.
[28] Ogawa K, Sanada K, Machida S, Okutsu M, Suzuki K. Resistance exercise training-induced muscle hypertrophy was associated with reduction of inflammatory markers in elderly women. Mediat Inflamm 2010;2010:171023.
[29] Mraz M, Haluzik M. The role of adipose tissue immune cells in obesity and low-grade inflammation. J Endocrinol 2014;222:R113–27.
[30] Bassit RA, Curi R, Costa Rosa LF. Creatine supplementation reduces plasma levels of pro-inflammatory cytokines and PGE2 after a half-ironman competition. Amino Acids 2008;35:425–31.
[31] Santos RV, Bassit RA, Caperuto EC, Costa Rosa LF. The effect of creatine supplementation upon inflammatory and muscle soreness markers after a 30km race. Life Sci 2004;75:1917–24.
[32] Deminice R, Rosa FT, Franco GS, Jordao AA, de Freitas EC. Effects of creatine supplementation on oxidative stress and inflammatory markers after repeated-sprint exercise in humans. Nutrition 2013;29:1127–32.
[33] Blüher M, Mantzoros CS. From leptin to other adipokines in health and disease: facts and expectations at the beginning of the 21st century. Metabolism 2015;64:131–45.
[34] Sales LP, Pinto AJ, Rodrigues SF, Alvarenga JC, Goncalves N, Sampaio-Barros MM, et al. Creatine supplementation (3 g/d) and bone health in older women: a 2-year, randomized, placebo-controlled trial. J Geron Series A Biol Sci Med Sci 2020;75:931–8.
[35] Candow DG, Vogt E, Johannsmeyer S, Forbes SC, Farthing JP. Strategic creatine supplementation and resistance training in healthy older adults. App Phy Nutr Metab 2015;40:689–94.
[36] Staples AW, Burd NA, West DWD, Currie KD, Atherton PJ, Moore DR, et al. Carbohydrate does not augment exercise-induced protein accretion versus protein alone. Med Sci Sports Exerc 2011;43:1154–61.
[37] Svanberg E, Jefferson LS, Lundholm K, Kimball SR. Postprandial stimulation of muscle protein synthesis is independent of changes in insulin. Am J Physiol 1997;272:E841–7.
