# Supplementary material for: Impact of creatine supplementation on inflammation: evidence from a systematic review and meta-analysis of randomized double-blind placebo trials
Source: Front Immunol. 2026 Feb 19;17:1743603. doi: 10.3389/fimmu.2026.1743603 (PMC12961398; doi:10.3389/fimmu.2026.1743603)
Supplement: Supplementary file 2 [file SupplementaryFile1.zip › SR Creatine inflammatory markers (Kell Doutorado). /Supplementary Files/ROB 2/ (revisado). Rob 2 no WORD.docx]

Taes et al 2004

1.1: Y – “The study followed a double-blind, placebo-controlled, crossover design (Fig. 2). Patients received 2 g creatine or placebo daily in the evening during 2 treatment periods of 4 weeks, in random order, and separated by a washout period of 4 weeks.

1.2: PY: Comment: In a double-blind study, neither the participants nor the researchers administering the treatment know which intervention is being given, which inherently requires the allocation sequence to be concealed to maintain the blinding.

1.3: “No baseline differences in age, gender distribution, body mass index, tHcy concentrations, or dialysis parameters were observed between placebo and creatine groups for both treatment periods (Table 1).”

Algorithm result: LOW

Assessor`s judgement: LOW


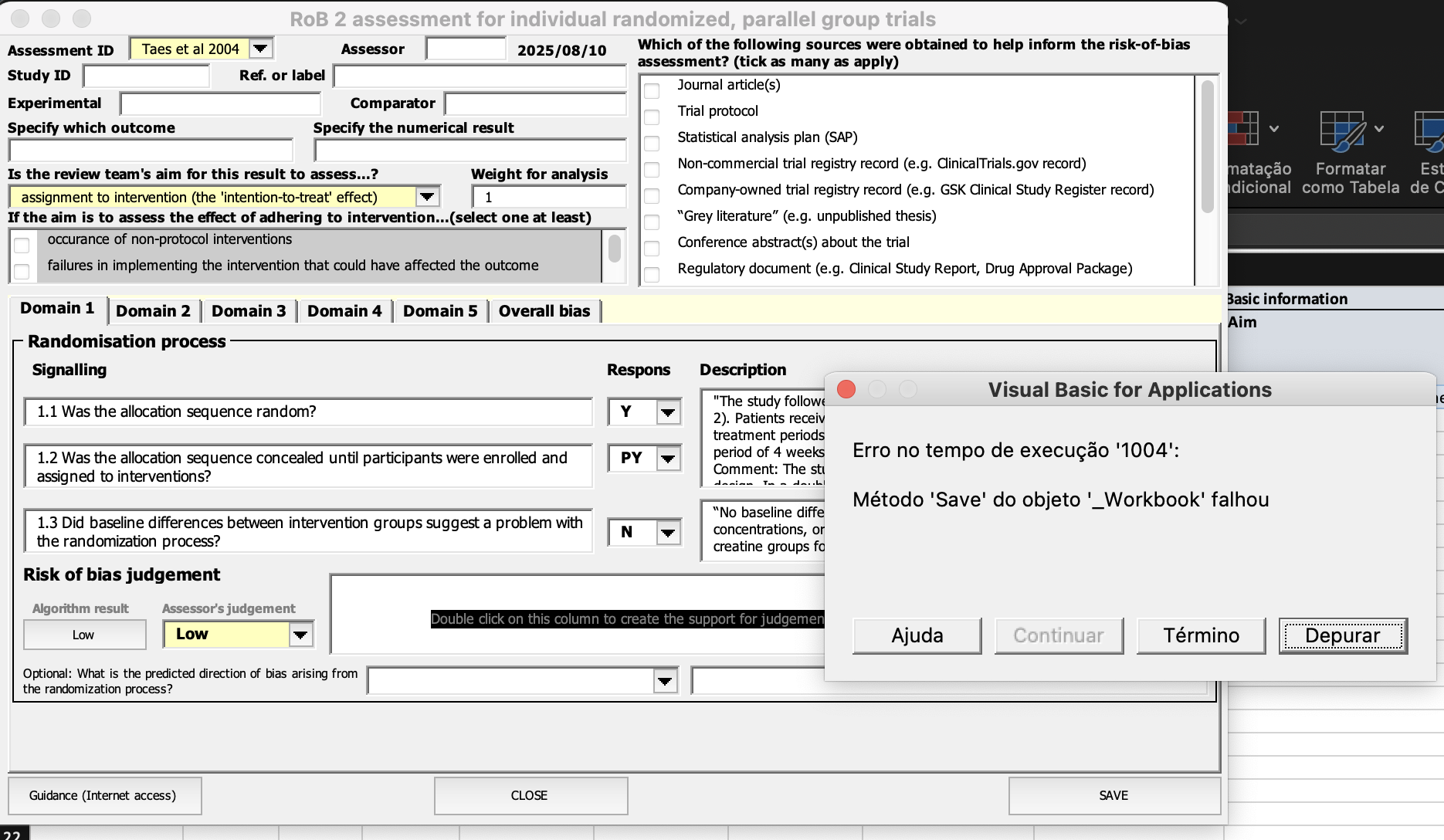


2.1: N: “The study followed a double-blind, placebo-controlled, crossover design (Fig. 2). Patients received 2 g creatine or placebo daily in the evening during 2 treatment periods of 4 weeks, in random order, and separated by a washout period of 4 weeks.”

2.2: N: Comment: As the study used a double-blind design, it implies that neither the participants nor the researchers or carers involved in administering the intervention knew the assigned treatment.

2.3: N: “The study implemented a double-blind design, preventing deviations due to awareness. While four patients were excluded from the study due to transplantation, death, or quitting, and not completing the full protocol, the remaining patients' compliance was ascertained by counting pills. There is no information suggesting deviations from the intended intervention for those who continued in the study.”

2.6: “The study employed a mixed effect model for analysis, treating the patient as a random effect and modeling the treatment mode, period, and carry-over as fixed effects. This statistical approach is appropriate for a crossover trial design, as used in this study, to estimate the effect of assigned intervention.”

3.1: N: “Forty-five hemodialysis patients (24 males, 21 females) with a mean age of 70 ± 10 years (range 35–88) were included in this study. Four patients were excluded from the study (2 patients were transplanted during the study, 1 died, and 1 quit the study). Twenty-five patients received creatine, and 20 patients received placebo in the first treatment period. No baseline differences in age, gender distribution, body mass index, tHcy concentrations, or dialysis parameters were observed between placebo and creatine groups for both treatment periods (Table 1).”

Y: Comment: 45/49 participants completed the study; 4 dropped out (due to transplantation, death, or withdrawal). Dropout was unrelated to outcome and small in proportion

3.2: PY: Comment: The reasons for patient exclusion (transplantation, death, or quitting the study) are generally considered unrelated to the expected effect of creatine supplementation on tHcy concentrations, thus making systematic bias from these specific dropouts less likely.

4.1: N: “Study design and population Forty-nine hemodialysis patients were recruited. Dialysis was performed with a low-flux triacetate dialyzer (Sureflux-L; Nipro, Osaka, Japan) for 4 to 5 hours 3 times weekly for at least 3 months. Exclusion criteria were: acute illness, life expectancy <3 months, low compliance due to cognitive, social, or psychiatric problems, and in-ability to provide informed consent. All patients were treated with folic acid 5 mg, pyridoxine 50 mg, and vita-

min B12 12 lg orally 3 times a week for at least 3 months. Written informed consent was obtained from all participants. The study protocol was approved by the Ethical Committee of AZ Sint-Jan AV. Sample size estimation (type I error= 0.05; type II error= 0.20), based on the Hcy lowering in our animal study ( tHcy= 20%) and tHcy concentrations in dialysis patients, revealed sufficient statistical power of the present study. The study followed a double-blind, placebo-controlled, crossover design (Fig. 2). Patients received 2 g creatine or placebo daily in the evening during 2 treatment periods of 4 weeks, in random order, and separated by a washout period of 4 weeks. Creatine monohydrate (CreaPure ) was obtained from Degussa Bioactives (Freising, Germany). Placebo tablets contained Fast Flo lactose (Foremost Farms, Baraboo, WI, USA). An exact number of tablets was supplied, and pills were counted to ascertain compliance. Treatment duration, blood flow,dialysate flow, and membrane surface area were determined by the attending nephrologists, but no changes were made during the study period. Single pool Kt/Vurea was calculated at the start and end of the study, where K=2424 Taes et al: Creatine supplementation and homocysteine in hemodialysis dialyzer urea clearance, T= duration of dialysis, and V= urea distribution volume at the end of dialysis. Predialysis blood samples were obtained at baseline and after 4 weeks in both treatment periods. EDTA-samples for tHcy determinations were transported on ice, centrifuged immediately, and stored at−20◦C until testing.

Biochemical determinations

Plasma creatinine concentrations were determined using a compensated rate-blanked Jaff´e based method (Roche Diagnostics, Mannheim, Germany) on a Modular P analyzer (Roche Diagnostics) according to the manufacturer’s procedure. Plasma urea concentrations and aspartate-aminotransferase (AST), alanine-aminotransferase (ALT), alkaline phosphatase, creatine kinase, and c-glutamyl transferase (GGT) activities were determined on a Modular P analyzer using comercial reagents (Roche Diagnostics).

Total plasma homocysteine concentrations were determined using a fluorescence polarization immunoassay on an Axsym analyzer (Abbott Laboratories, Abbott Park, IL, USA). Plasma folate and vitamin B12 concentrations were determined using an electrochemiluminescence assay

(Roche Diagnostics) on an Elecsys 2010 analyzer (Roche Diagnostics). Plasma and erythrocyte creatine concentrations were determined enzymatically as described before [13]. Plasma creatine was determined without de- proteinization, whereas erythrocyte creatine was deter- mined after deproteinization with 5-sulfosalicylic acid. No analytical interference of creatine on tHcy was ob- served. The prognostic inflammatory and nutritional index (PINI) was calculated as PINI= [(a1-acid glyco- protein (mg/L) × CRP (mg/L)]/[(albumin (g/L) × prealbumin (mg/L)] [14, 15]. a1-acid glycoprotein, albumin, and prealbumin were determined nephelometrically using commercial reagents (Dade Behring, Marburg, Germany) on a BN II nephelometer. CRP was determined turbidimetrically on a Modular P analyzer (Roche Diagnostics).

Statistics Data are expressed as mean ± SD, unless parameters were not normally distributed [median (interquartile range)]. Student t test was used to compare separate groups when appropriate; otherwise nonparametric group comparison was performed using Mann-Whitney U test. Associations between continuous variables were examined using Spearman rank correlation analysis. In order to estimate the effect of creatine treatment on the tHcy concentrations in this crossover framework, a mixed effect model was fitted treating patient as a random effect while the treatment mode, period, and carry-over were

Table 1.

Model fitting was performed using SAS software (PROC MIXED) (release 8.1, SAS Institute, Inc., Cary, NC, USA). Differences were considered significant at P less than 0.05.”

Comment: “The study was conducted using a double-blind, placebo-controlled, crossover design. This design inherently minimizes the potential for measurement or ascertainment of outcomes to differ between groups, as neither the participants nor the researchers involved in conducting the measurements were aware of the assigned intervention. The biochemical determinations were performed on automated analyzers using commercial reagents, which further reduces human bias in the measurement process.”

4.2: N: Comment: Given the double-blind nature of the study, the outcome assessors (those performing the biochemical analyses and interpreting the results) would have been unaware of whether participants received creatine or placebo.

4.3 N: Comment: Outcome assessors were blinded

5.1: PY: “The study employed a double-blind, placebo-controlled, crossover design, which means the unblinding of outcome data would occur after data collection. The methods section details a specific statistical approach for the primary outcome: "a mixed effect model was fitted treating patient as a random effect while the treatment mode, period, and carry-over were modeled as fixed effects". The sample size estimation was also described, "based on the Hcy lowering in our animal study ( tHcy = 20%) and tHcy concentrations in dialysis patients, revealed sufficient statistical power of the present study". This level of detail and the use of an appropriate statistical model for the design strongly suggest a pre-specified analysis plan, even if the exact timing of its finalization relative to unblinding is not explicitly stated.”

NI: Comment: a pre-specified analysis plan not mentioned

5.2: N: Comment: The study clearly states its focus on "total plasma homocysteine (tHcy) concentrations" as the primary outcome related to the effect of creatine supplementation. The method for determining tHcy concentrations is consistently described. While other biochemical parameters were measured, tHcy was the specific outcome investigated for the effect of creatine, and there is no indication that different measures or definitions of homocysteine were explored or selectively reported. (PN)

5.3: N: Comment: he study explicitly describes the single statistical method used to analyze the effect of creatine treatment on tHcy concentrations: a mixed effect model. This model is appropriate for the crossover design used. The results section then presents the findings from this specific model (Table 2), indicating "No treatment, period, or carryover effects were detected". There is no mention of or suggestion that multiple analytical approaches were attempted or that results were chosen from various analyses. (PN)

Algorithm result: Some concerns

Assessor’s judgement: Some concerns

Santos et al 2004.

- 1. – PY – “After signing an Informed Consent form, thirty four male athletes received, for 5 days, 20g of creatine monohydrate divided in 4 doses of 5g, with 60g of carbohydrate (maltodextrine, n = 18) or the same amount of carbohydrate (n = 16)—placebo group, and ran 30km in a double-blind trial.”

     1.1 NI - Comment: The study is described as double-blind, but it does not specify the method used to generate the random sequence.

1.2 – Y – “The carbohydrate offered for both groups had the same flavour and color to avoid identification of the samples.”

1.3 – N – “There was no significant difference between the time that the athletes from both groups, placebo and creatine supplemented, took to finish the 30km.”

Algorithm result: LOW

Assessor’s judgement: LOW

2.1 – N: ““The carbohydrate offered for both groups had the same flavour and color to avoid identification of the samples.”

2.2 N – Comment: as a double-blind trial, both carers and people delivering weren’t aware of intervention.

2.6 – Y – “The data obtained were compared using a 2 2 repeated measures ANOVA and the post-hoc test of Tukey and the level of significance of at least p < 0.05 was chosen for all statistical comparisons. The data are presented as mean F SEM.“

Algorithm result: LOW

Assessor’s judgement: LOW

3.1 – N - The study initially included thirty-four male athletes (18 in the creatine group and 16 in the placebo group). However, the results presented in Table 2 are based on "23 samples". This indicates that data for the outcomes were not available for all, or nearly all, participants randomized, as approximately 32% of the initial participants' data are not accounted for in the results.

3.2 – N - The study does not provide any explanation for why only 23 samples were used in the analysis when 34 athletes were initially randomized. There is no discussion of participant dropouts, withdrawals, or issues with sample collection/analysis that would explain the missing 11 samples. Furthermore, the study does not mention any statistical methods used to account for missing data, such as intention-to-treat analysis or sensitivity analysis, which could help mitigate potential bias.

3.3 – PY - Given that a significant portion of the outcome data (approximately 32%) is missing without explanation, and these blood samples were collected 24 hours after the 30km race, it is plausible that the reasons for the missing data could be related to the true physiological outcome values. For instance, athletes who experienced more severe muscle soreness or inflammatory responses might have been less able or willing to provide the follow-up blood sample, which could introduce bias into the results. While the study states "All the athletes finished the race" and "did not present any side effects such as cramping, dehydration or diarrhea", these statements primarily refer to the period during and immediately after the race, not necessarily to the 24-hour post-race blood collection point where the missing data occurred.

Comment: Due to the substantial proportion of unexplained missing outcome data (11 out of 34 participants) and the fact that these data points are from blood samples collected 24 hours post-race, it is reasonable to consider that the missingness could be related to the unobserved outcome values. Without any information from the authors on the reasons for these missing samples or any analysis to address potential bias, the possibility that the missingness was not random and was instead dependent on the athletes' actual post-race physiological status cannot be dismissed.

Algorithm result: HIGH

Assessor’s judgement: HIGH

4.1 – N - The study utilized standard and commercially available methods for measuring the outcomes. Plasma TNF-a and PGE2 concentrations were assessed using commercially available ELISA-kits. LDH was measured spectrophotometrically, and CK and creatinine plasma concentrations were determined using commercial kits. Blood samples were stored at -80°C and analyzed within one week, which is appropriate for sample stability. These methods are generally accepted in biochemical research for these markers.

4.2 – N - The study describes standardized procedures for blood sample collection ("15 min before the event and 24h after the race, in sterile tubes containing heparin") and analysis methods (e.g., specific kits and spectrophotometry) that were applied to all samples, regardless of the intervention group. There is no information suggesting that the measurement or ascertainment process differed between the creatine and placebo groups.

4.3 – N - The study was conducted as a "double-blind trial". This means that both the participants and the researchers (including those involved in sample handling and analysis, who would be the outcome assessors) were unaware of which treatment each participant received. The specific measures taken, such as the carbohydrate for both groups having the "same flavour and color to avoid identification of the samples", support the effectiveness of this blinding for all involved personnel.

Algorithm result: LOW

Assessor’s judgement: LOW

5.1 – NI - The details the statistical methods used, specifically a "2 x 2 repeated measures ANOVA and the post-hoc test of Tukey" with a significance level of "p < 0.05". However, it does not explicitly state whether this analysis plan was finalized before the unblinded outcome data became available or if it was pre-specified.

5.2 – N – (PN) The study clearly specifies the outcome markers measured: creatine kinase (CK), lactate dehydrogenase (LDH), prostaglandin E2 (PGE2), and tumor necrosis factor-alpha (TNF-a). Blood samples were collected at two specific time points: "15 min before the event and 24h after the race". The results consistently report these specific markers at these predetermined time points, with no indication of other potential measurements, scales, definitions, or time points being considered or selected from.

5.3 – N – (PN)The study explicitly states the statistical analysis method used: "2 x 2 repeated measures ANOVA and the post-hoc test of Tukey". It also mentions the software used for analysis. There is no information provided to suggest that multiple different analytical approaches were explored or that the reported results were selected from several eligible analyses.

Algorithm result: Some concerns

Assessor’s judgement: Some concerns

Rawson et al 2007

1.1 – Y – “Participants were randomly placed into either a creatine or placebo group, and supplements were administered in a double-blind, placebo-controlled manner.” NI – no information about the randomization process

1.2 – PY – Comment: The supplements were administered in a double-blind, placebo-controlled manner. This methodology implies that neither the participants nor the administrators knew which group each participant was assigned to, thereby concealing the allocation sequence at the point of enrollment and assignment.

1.3 – N - The study explicitly state that there were no differences between creatine- and placebo-supplemented subjects before supplementation. Specifically, there were no significant differences in age, height, body mass, years of training experience, maximal squat strength, range of motion, creatine kinase, lactate dehydrogenase, or C-reactive protein between the two groups at baseline.

Algorithm result: LOW

Assessor’s judgement: LOW

2.1 – N - The supplements were administered in a double-blind, placebo-controlled manner, meaning participants were unaware of whether they received creatine or a placebo.

2.2 – N - The study explicitly states that supplements were administered in a double-blind, placebo-controlled manner. This indicates that the people delivering the interventions were also unaware of the participants' assignments to maintain the integrity of the blinding.

2.6 – Y - The study conducted sample size estimation and used a repeated-measures analysis of variance (ANOVA) with a grouping factor to assess changes between groups over time. Tukey's post hoc tests were used when significant interactions were found, and variables deviating from normality (creatine kinase and C-reactive protein) were log-transformed before analysis. Significance was set at p ≤ 0.05. This methodology aligns with appropriate statistical practices for a study of this design.

Algorithm result: LOW

Assessor’s judgement: LOW

3.1 – Y - The study states that "Twenty-two resistance-trained men... completed the study". The results section then presents data for all measured outcomes (muscle strength, range of motion, muscle soreness, creatine kinase, lactate dehydrogenase, and C-reactive protein) for both the creatine and placebo groups across all pre- and post-exercise time points without any indication of missing data or participant dropouts. This suggests that data were collected from all or nearly all randomized participants.

Algorithm result: LOW

Assessor’s judgement: LOW

4.1 – N - “Descriptive characteristics and baseline measures of outcome variables are described in Table 1. There were no differences in age, height, body mass, years of training experience, maximal squat strength, range of motion, creatine kinase, lactate dehydrogenase, or C-reactive protein between creatine and placebo groups (Table 1).”

Comment: The study utilized standard and widely accepted methods for measuring outcomes.

4.2 – PN - The study was conducted in a double-blind, placebo-controlled manner. This implies that not only the participants and supplement administrators but also the personnel involved in assessing the outcomes were unaware of the group assignments. For objective measures like strength, ROM (using a goniometer), and blood markers (CK, LDH, CRP measured by automated analyzers or EIA kits), the methods are standardized and less prone to differential measurement bias. For subjective measures like muscle soreness, the double-blind design is crucial in minimizing potential bias in ascertainment between groups.

4.3 – N - The overall design of the study was double-blind. In a double-blind study, typically all parties involved in the study's execution, including outcome assessors, are unaware of the participants' assigned interventions to prevent bias.

Algorithm result: LOW

Assessor’s judgement: LOW

5.1 – PY - The study describes a detailed statistical analysis plan, including sample size estimation conducted prior to the study to determine the necessary number of subjects (10 subjects per group to detect a difference in creatine kinase). It also specifies the statistical tests to be used (Kolmogorov-Smirnov for normality, log-transformation for non-normal variables, repeated-measures ANOVA with a grouping factor, and Tukey's post hoc tests), and the significance level (p ≤ 0.05). While it doesn't explicitly state that this plan was finalized before unblinding, the level of detail provided and the fact that it's a double-blind, placebo-controlled study, suggests that the analysis plan would have been established beforehand to maintain research integrity and prevent bias.

5.2 – N - The study examined multiple indirect markers of muscle damage: strength, range of motion, muscle soreness (both with movement and palpation), creatine kinase, lactate dehydrogenase, and C-reactive protein. For each of these, specific measurement methods were used (e.g., 1RM for strength, goniometer for ROM, visual analog scale for soreness, specific assays for blood markers). All measured outcomes were assessed at pre-defined time points (pre-exercise, immediately post-exercise, and during a 5-day follow-up period). The results section presents data and statistical outcomes for all of these variables and time points, with no indication that only a subset of eligible measurements or time points were selected for reporting based on their outcomes.

5.3 – N - The "Statistical Analyses" section clearly outlines the specific analytical approach used, including normality testing, log-transformation for specific variables, and repeated-measures ANOVA with Tukey's post hoc tests. There is no information in the study to suggest that different analytical approaches were attempted or that results were selectively reported from multiple eligible analyses. The stated methods represent a single, consistent analysis plan.

Algorithm result: LOW

Assessor’s judgement: LOW

Oliveira et al 2020

1.1 – Y – “This study was a part of a randomized, double-blind, placebo- controlled, parallel-group clinical trial, registered at ensaio-sclinicos.gov.br (RBR-2shfhj) and approved by the Human Research Ethics Committee of the Federal University of Goias (840.317).”

1.2 – Y – Comment: The study was conducted as a "double-blind" trial.

PY – Comment: The study claims to be randomised and mentions “stratification by sex,” which suggests some random procedure, although the method of sequence generation is not detailed. The packages were coded and kept blinded to participants and investigators until the end of the analysis, indicating adequate concealment.

1.3 – N – “After 12 weeks of intervention, there were no differences between groups in any of the variables analyzed..”

Algorithm result: LOW

Assessor’s judgement: LOW

2.1 – N - The study was a "double-blind" trial, meaning that neither the investigators nor the participants were aware of the contents of the supplement packages until the analysis was completed.

2.2 – N - The trial design was "double-blind", which specifically states that investigators were unaware of the supplement contents until the completion of the analysis. This implies that those administering the intervention were also unaware.

2.6 – Y - “Data distribution was evaluated by ShapiroeWilk W test. Variables that were not normally distributed were log-transformed for statistical analysis. Within-group differences (i.e., before and after intervention) were assessed by a paired samples t-test. Differences between groups were assessed by analysis of covariance (ANCOVA) with the baseline value as covariate and the change from baseline as the dependent variable. Potential confounders (covariates) that could affect biochemical measures and body composition were examined in the groups. Signi cant covariates were identi ed using multiple linear regression with backward elimination of those that were not signi cant. Effect sizes were calculated using Cohen's dz formula and classi ed as small (d¼ 0.2), medium (d¼ 0.5), and large (d¼ 0.8) [15]. STATA version 12 (StataCorp, College Station, TX) and G*Power version 3.1.9.2, (Universit€ at Düsseldorf, Germany) were used to perform all statistical analyses. Differences were regarded as statistically signi cant if p < 0.05.”

Comment: Differences between groups were assessed using analysis of covariance (ANCOVA), with the baseline value as a covariate and the change from baseline as the dependent variable. Potential confounders that could affect biochemical measures and body composition were also examined. Given that no significant between-group differences were observed at baseline for participants' characteristics and dietary intake, ANCOVA adjusted for baseline values is an appropriate statistical method for comparing intervention effects in this randomized, parallel-group trial.

Algorithm result: LOW

Assessor’s judgement: LOW

3.1 – N - Thirty-two healthy older adults were randomly assigned to the study groups. However, only 14 participants from the Placebo with Resistance Training (PL + RT) group and 13 from the Creatine supplementation with Resistance Training (CR + RT) group completed the trial and were included in the analyses. This means 27 out of 32 participants completed, indicating that 5 participants (approximately 15.6%) did not complete the trial and their outcome data would be missing. This does not qualify as "nearly all" participants.

3.2 – N - The study state the number of participants who completed the trial but do not provide any information regarding the reasons for participant dropout or any methods used to handle the missing data (e.g., imputation, sensitivity analyses) to ensure the results were not biased.

3.3 – PY - Since the reasons for the 5 participant dropouts are not provided, it is possible that participants might have discontinued their involvement for reasons related to the intervention's effects or lack thereof (e.g., experiencing side effects, perceiving no benefit), which could directly relate to the true value of their outcomes.

3.4 – NI - While it is possible that the missingness could depend on the true value due to the absence of information on dropout reasons (as per 3.3), the study do not provide enough details or context about the dropouts to determine whether it is likely that missingness depended on the true value. We only know that participants did not complete the trial.

Algorithm result: HIGH

Assessor’s judgement: HIGH

4.1 – N – “All biochemical analyses were done at baseline and after 12 weeks of intervention. Two days after the last day of intervention, participants were instructed to take their medications and attend the research unit in the morning after a 12-hour overnight fast. Blood samples were obtained from the antecubital vein. Immediately after collection, venous blood glucose was measured using a portable glucose meter (Accu-check, F. Hoffmann-La Roche Ltd, Switzerland). The remaining blood was inserted into an EDTA vacutainer and placed on ice before being centrifuged for 10 min at 4 C and 3.500 rpm. Plasma was then pipetted into aliquot tubes and stored at 80 C until analysis. Monocyte chemoattractant protein-1 (MCP-1), IL-6, IL-10, leptin and adiponectin were analyzed using the Human Quantikine ELISA kits (R&D Systems©), according to manufacturer's instructions and were determined in duplicates. C-reactive protein was analyzed by turbidimetric assay. Insulin concentration was determined using Insulin AccuBind ELISA Kits (Monobind Inc.) according to manufacturer's instructions. Homeostatic model assessment (HOMA) of b-cell function (%B) and insulin resistance (IR) were calculated using the HOMA2 Calculator (©Diabetes Trials Unit, University of Oxford, version 2.2.3). Intra- and inter-assay coef cients of variation (CV) were as follows: MCP- 1:4.7 and 4.6, IL-6: 1.7 and 2.0, IL-10: 1.7 and 5.9, leptin: 3.0 and 4.2, adiponectin: 2.5 and 6.8, and insulin: 5.1 and 7.2, respectively.

Statistical analyses

A post hoc power analysis was conducted on MCP-1 from the CR þ RT group (n¼ 11) using a two-tailed Wilcoxon matched pairs signed-rank test. With an effect size of 1.13 and type I error probability of 0.05, the estimated achieved power was 90%. Data distribution was evaluated by ShapiroeWilk W test. Vari-

ables that were not normally distributed were log-transformed for statistical analysis. Within-group differences (i.e., before and after intervention) were assessed by a paired samples t-test. Differences between groups were assessed by analysis of covariance (ANCOVA) with the baseline value as covariate and the change from baseline as the dependent variable. Potential confounders (covariates) that could affect biochemical measures and body composition were examined in the groups. Signi cant covariates were identi ed using multiple linear regression with backward elimination of those that were not signi cant. Effect sizes were calculated using Cohen's dz formula and classi ed as small (d¼ 0.2), medium (d¼ 0.5), and large (d¼ 0.8) [15]. STATA version 12 (StataCorp, College Station, TX) and G*Power version 3.1.9.2, (Universit€ at Düsseldorf, Germany) were used to perform all statistical analyses. Differences were regarded as statistically signi cant if p < 0.05.”

4.2 – N - The study was conducted as a "double-blind" trial. This means that neither the investigators nor the participants were aware of the contents of the supplement packages until the completion of the analysis. This strong blinding procedure ensures that the measurement or ascertainment of outcomes would not differ systematically between the creatine and placebo groups due to knowledge of the assigned intervention.

4.3 – N - Given that the trial was "double-blind," the investigators, who would be responsible for assessing the outcomes, were not aware of the contents of the supplement packages or which participants received the active intervention versus the placebo until the analysis was completed.

Algorithm result: LOW

Assessor’s judgement: LOW

5.1 – Y - The study was registered at ensaio-sclinicos.gov.br (RBR-2shfhj), which indicates a pre-specified plan. Furthermore, the trial was designed as a "double-blind" study, meaning that neither the investigators nor the participants were aware of the contents of the supplement packages until the completion of the analysis. This blinding procedure strongly supports that the analysis plan would have been finalized before unblinding occurred and before outcome data were available for analysis in an unblinded manner. The "Statistical analyses" section also details specific methods used for assessing data distribution, within-group differences, and between-group differences.

5.2 – N - The study explicitly measured and reported on a comprehensive list of outcomes, including glucose, insulin, HOMA %b, HOMA IR, adiponectin, leptin, IL-6, IL-10, MCP-1, and CRP. All of these measurements were taken at baseline and after 12 weeks of intervention. Table 2 presents the data for all these markers at both time points, along with their respective within-group and between-group p-values. While the discussion highlights the within-group reduction of MCP-1 as a key finding, this does not imply that other eligible outcome measurements were selectively omitted from reporting. All measured outcomes appear to have been consistently presented.

5.3 – N - The "Statistical analyses" section clearly outlines the specific methods used for data analysis, including Shapiro-Wilk W test for distribution, log-transformation for non-normally distributed variables, paired samples t-test for within-group differences, and analysis of covariance (ANCOVA) with baseline values as covariates for between-group differences. Table 2 comprehensively presents the results of both within-group (P1) and between-group (P2) analyses for all measured outcomes. The paper directly states "there were no differences between groups in any of the variables analyzed", which aligns with reporting the ANCOVA results directly. The significant finding regarding MCP-1 reduction is a within-group change, analyzed by the pre-specified paired t-test. There is no indication that different analytical approaches were explored or selected based on the resulting numerical outcomes.

Algorithm result: LOW

Assessor’s judgement: LOW

Overall bias:

Algorithm result: HIGH

Assessor’s judgement: HIGH

Marini et al 2024

1.1 – N – “After the inclusion of the patients in the study, they were allocated by gender and age (Figure 1).”

1.2 – Y – Comment: The study utilized a double-blind design, and the interventions were performed with blinded sachets standardized to prevent patients from identifying their contents. This indicates that neither the participants nor the researchers involved in the administration knew the group assignments, ensuring concealment.

1.3 – N - The baseline characteristics of both the creatine and placebo groups were similar in terms of sex, age, BMI, hemodialysis time, Kt/v, and life habits. Table 1 shows that all comparisons of baseline variables between the groups had p-values greater than 0.05, indicating no statistically significant differences. This suggests that the allocation process, which aimed to balance the groups, did not result in problematic baseline imbalances.

Algorithm result: Some concerns

Assessor’s judgement: Some concerns

2.1 – N – The study was conducted using a double-blind design. The interventions were performed with blinded sachets that were standardized to prevent patients from identifying their contents.

2.2 – N - The study utilized a double-blind design. This design implies that neither the participants nor the individuals administering the interventions (carers/researchers) were aware of the assigned group to maintain objectivity. The use of blinded and standardized sachets further supports that the content was concealed from all parties involved in the administration.

2.6 – Y - The study utilized an Intention to Treat (ITT) analysis, which is considered an appropriate statistical approach for randomized controlled trials to estimate the effect of assignment to intervention, as it preserves the integrity of the initial randomization. For missing data, the ITT analysis used the Last Observed Carried Forward (LOCF) method for imputation.

Algorithm result: LOW

Assessor’s judgement: LOW

3.1 – N - The study explicitly states that "During the intervention there were sample losses due to several factors as shown in Figure 1, thus causing missing data or dropouts". This indicates that data were not available for all participants who were initially randomized.

3.2 – Y - The authors acknowledge that withdrawals (dropouts/missing data) "cause a reduction in the sample and promote a false estimate of the treatment effect". To address this potential bias, they state that "the sample was evaluated with the imputation of data (by intention to treat—ITT), a method in which the value of the last result of the individual at the moment that is missing was replicated (last observed carried forward-LOCF)". The use of ITT analysis with LOCF imputation is evidence of their attempt to mitigate bias from missing outcome data.

Algorithm result: LOW

Assessor’s judgement: LOW

4.1 – N - The study utilized standardized and widely accepted methods for measuring outcomes. Body composition was assessed using a bioimpedance medical device (Model mBCA 525, Seca, Hamburg, Deutschland, Germany), and anthropometric data (weight, height, calf circumference) were collected following established recommendations. The Malnutrition-Inflammation Score (MIS) is a recognized instrument for assessing nutritional status. Biochemical analyses (creatinine, urea, phosphorus) were performed using Abbott methods. The authors themselves highlighted the use of BIA to assess body composition and hydration status as a "positive point" of the study.

4.2 – N – The study explicitly states it was an "exploratory 1-year, balanced, placebo-controlled, and double-blind design". Furthermore, the intervention was performed with "blinded sachets" that were "standardized to avoid any identification of the content by the patients". This double-blind approach and standardization indicate that the procedures for measurement and ascertainment of outcomes would have been applied identically to both groups, without knowledge of the assigned intervention.

4.3 – N - Given that the study employed a double-blind design, it implies that both the participants and the researchers involved in the administration and assessment of interventions were unaware of the group assignments. This inherent aspect of a double-blind study means that outcome assessors would also have been blinded to the intervention received by participants.

5.1 – PY - The study describes its statistical methods in detail in section 2.8, including the software used (SPSS 21 and G*Power 3.1), tests for normality (Shapiro-Wilk Test), methods for categorical variables (Chi-square and Fisher’s exact test), and methods for continuous variables (Mann–Whitney test, Friedman test, Wilcoxon test). It also specifies the level of statistical significance (p < 0.05) and how effect size was estimated (Cohen d test). Importantly, it states that the sample size was calculated using Gpower® 3.1 software beforehand. The use of a "double-blind design" means that the outcome data would have been unblinded only after the data collection was complete, aligning with the idea that the analytical plan was in place before the results were known. While the text doesn't explicitly state "pre-specified analysis plan was finalized before unblinded outcome data," the comprehensive description of statistical methods and prior sample size calculation strongly suggests this was the case.

5.2 – N - The study clearly defines the primary outcome domains and their measurements: Malnutrition-Inflammation Score (MIS) using the Kalantar-Zadeh et al. (2001) instrument, and body composition assessed by a bioimpedance medical device (Model mBCA 525, Seca). Specific measures within body composition such as fat-free mass (FFM), skeletal muscle mass index (SMMI), total body water (TBW), intracellular water (ICW), and extracellular water (ECW) were pre-defined and measured. The analyses were performed at three pre-defined time points: pre, intermediate (after 6 months), and post (after 12 months). There is no indication that multiple unstated measurements were taken and then results were selectively chosen.

5.3 – N - The study describes its statistical analysis methods explicitly. It also clearly states that "the sample was evaluated with the imputation of data (by intention to treat—ITT), a method in which the value of the last result of the individual at the moment that is missing was replicated (last observed carried forward-LOCF)" to account for missing data.

Algorithm result: LOW

Assessor’s judgement: LOW

Marini et al 2019

1.1 – Y – “This randomized, placebo-controlled, and double-blind clinical trial was conducted with patients of both sexes who were diagnosed with CKD, undergoing HD, and aged between 18 and 59 years. The overall study lasted 6 weeks, and the intervention with creatinewas 4weeks.”

1.2 – PY - The study was conducted as a double-blind clinical trial, meaning neither the participants nor the researchers knew the group assignments. The intervention itself was blinded, and the sachets containing creatine or placebo were standardized to prevent patients from identifying the content. This strong emphasis on blinding implies that the allocation sequence would have been concealed to maintain the integrity of the blinding process.

1.3 – N - The study state that the baseline characteristics of both groups were similar for sex, age, body mass index (BMI), and previous comorbidities. Additionally, food intake at baseline was also similar between the groups. This indicates that the randomization process successfully created comparable groups without significant baseline differences.

Algorithm result: LOW

Assessor’s judgement: LOW

2.1 – N - The study was designed as a double-blind clinical trial, and the intervention was blinded. The sachets containing creatine or placebo were standardized to avoid any identification of the content by the patients.

2.2 – N - As a double-blind clinical trial, it implies that neither the participants nor the researchers (including carers and those delivering the interventions) knew the group assignments.

2.6 – Y - The study used standard and appropriate statistical methods for analyzing a randomized controlled trial, including descriptive statistics, Shapiro-Wilk Test for normality, $\chi^2$ test for categorical variables, Wilcoxon test or Mann-Whitney U test and Student t-test for various comparisons, and two-way ANOVA followed by post hoc Tukey test to evaluate the interaction between supplements and intervention time. The level of statistical significance was set at 5% (P < .05).

Algorithm result: LOW

Assessor’s judgement: LOW

3.1 – PY - The study initially included 30 patients, with 15 randomly allocated to the Placebo Group (PG) and 15 to the Creatine Group (CG). However, during the intervention period, one patient in each group was excluded due to nonadherence to the creatine supplementation (less than 90% of the recommended dose). This resulted in 14 patients analyzed per group for the outcomes. Therefore, data were available for 28 out of the 30 randomized participants. The exclusion of 2 out of 30 (approximately 6.7%) participants, especially when balanced across groups and due to non-adherence, generally means that data for "nearly all" participants were available for analysis in this pilot study.

Algorithm result: LOW

Assessor’s judgement: LOW

4.1 – N - The study utilized appropriate and commonly accepted methods for measuring outcomes. For instance, body composition (lean body mass and fat mass) was assessed by Dual-Energy X-ray Absorptiometry (DXA), which the study explicitly states "allows greater veracity in the results". The Malnutrition-Inflammation Score (MIS) is a validated tool for assessing nutritional status in hemodialysis patients. Quality of Life (QoL) was assessed using the SF-36 questionnaire, a "well-documented health-related instrument" validated in a Brazilian population. While the study acknowledged a limitation in not quantifying hydration status, which "may have altered the measured LBM" due to DXA not separating intracellular from extracellular water, this does not render the overall measurement method inappropriate, but rather highlights a specific nuance in interpreting LBM results.

4.2 – N - The study was conducted as a double-blind clinical trial. This means that neither the participants nor the researchers who were involved in measuring and ascertaining outcomes knew whether a participant received creatine or placebo. Furthermore, for anthropometric data and food intake, assessments were performed by trained nutritionists, which helps ensure standardization of data collection procedures across both groups. Biochemical analyses largely relied on objective laboratory methods. This double-blind, standardized approach minimizes the potential for differential measurement or ascertainment bias between the groups.

4.3 – N - The study was a double-blind trial. This design ensures that outcome assessors were unaware of which intervention each participant received.

Algorithm result: LOW

Assessor’s judgement: LOW

5.1 – PY - The study was a randomized, placebo-controlled, double-blind clinical trial. The study is also explicitly stated as part of a larger trial previously registered in the Brazilian Registry of Clinical Trials under the code RBR-98wzgn. Trial registration typically involves submitting a pre-specified protocol including the analysis plan. The description of statistical methods used is comprehensive and aligns with standard practices for such a trial. The double-blind nature of the study also indicates that the researchers who conducted the analyses would not have been aware of the group assignments during the data processing phase, thus minimizing the potential for analysis choices to be influenced by preliminary unblinded results.

5.2 – N - The study clearly defined its primary objectives as evaluating the attenuation of lean body mass (LBM) loss and malnutrition-inflammation score (MIS). For these outcomes, specific and validated measurement tools and methodologies were used: DXA for body composition, including LBM, and the Malnutrition-Inflammation Score (MIS) itself. The time points for evaluation (pre and post-intervention) were also clearly defined. There is no indication that the researchers chose from multiple different scales or definitions for LBM or MIS, or that results from various time points were selectively reported.

5.3 – N - The "Statistical Analyses" section explicitly details the methods used: Shapiro-Wilk Test for normality, $\chi^2$ test for categorical variables, Wilcoxon test or Mann-Whitney U test and Student t-test for various comparisons, and two-way ANOVA followed by post hoc Tukey test to evaluate the interaction between supplements and intervention time. The reported results for LBM and MIS changes, which involve supplement × time interaction, were specifically analyzed using two-way ANOVA with post hoc Tukey test. This indicates a consistent application of pre-defined and appropriate statistical methods, rather than the selection of a favorable analysis from multiple options.

Algorithm result: LOW

Assessor’s judgement: LOW

Overall bias:

Algorithm result: LOW

Assessor’s judgement: LOW

Deldicque et al 2008

1.1 – Y – “Subjects. Nine healthy young men (21.7 0.55 y, BMI 24 0.9 kg/m2) who did not partake in any formal resistance exercise regime, were recruited for this double-blind crossover study. All subjects were given an oral and written account of the study before signing a consente form. This study was approved by the Ethics Committee of the Universite ´ catholique de Louvain, and the investigatio was performed according to the principles outlined in the Declaration of Helsinki.”
NI – no information about the randomization process

“Subjects were randomly divided into two groups: one group (n 5) received 21 g (3 7 g) of oral creatine monohydrate per day for 5 days before the beginning of the experi- ment and during the 3 days of the experiment, whereas the second group (n 4) received a placebo (maltodextrin 3 7 g/day) during the same period (the protocol is summarized in Fig. 1).”

1.2 – Y - The study was conducted in a double-blind fashion and is described as a double-blind crossover study. This means that both the participants and the researchers were unaware of which treatment (creatine or placebo) was being administered at any given time, implying concealment of the allocation sequence.

1.3 – N - The study utilized a double-blind crossover design, where each subject served as their own control, receiving both creatine and placebo treatments separated by a washout period. This design inherently controls for individual baseline differences between participants. While the mean work performed during the exercise was not exactly the same for both legs, the study explicitly states that these values were not statistically different between the placebo and creatine trials, which is an important aspect of comparability for the exercise stimulus itself. No other reported baseline differences among the initial randomly assigned groups (n=5 vs n=4) suggest an issue with the randomization process.

Algorithm result: LOW

Assessor’s judgement: LOW

2.1 – N - The study was conducted in a double-blind fashion. This means that the participants were unaware of whether they were receiving creatine or a placebo.

2.2 – N - The study was conducted as a double-blind crossover study. In a double-blind study, neither the participants nor the researchers/personnel delivering the interventions are aware of the treatment assignments.

2.6 – Y - The study used a double-blind crossover design, which means each participant served as their own control, receiving both the creatine and placebo treatments. This design is excellent for controlling individual variability. For statistical analysis, they used a paired t-test to compare muscle creatine content between placebo and creatine conditions, which is appropriate for within-subject comparisons in a crossover study.Two-way ANOVA for repeated measures was used to evaluate treatment by time interactions, which is also appropriate for analyzing data from a crossover design with multiple time points. Student-Newman-Keuls post hoc tests were applied when necessary. The significance threshold was set to P < 0.05.

Algorithm result: LOW

Assessor’s judgement: LOW

3.1 – Y - The study recruited nine healthy young men. The results sections consistently present data for all nine subjects (n = 9) for key outcomes such as muscle creatine concentration, gene expression (mRNA levels), and protein phosphorylation states. There is no mention of participants dropping out or missing data for any of the measured outcomes.

Algorithm result: LOW

Assessor’s judgement: LOW

4.1 – N - The study utilized standard and widely accepted laboratory techniques for measuring the various outcomes: Muscle creatine concentration was determined spectrophotometrically, a method previously described and common in research; Protein extraction, cell fractionation, SDS/PAGE, and immunoblotting (Western blot) were used to assess protein phosphorylation states. These are standard biochemical techniques, and the purity of fractions was verified. Results were quantified using specialized software; RNA extraction and quantitative real-time PCR (RT-PCR) were used for gene expression analysis, following manufacturer instructions. A housekeeping gene (2-microglobulin) was used, which was verified not to be affected by exercise or creatine supplementation. These are also standard molecular biology techniques.

4.2 – N - The study was designed as a double-blind crossover study. This means that each subject received both the creatine and placebo treatments, serving as their own control. The methods for sample collection (muscle biopsies), processing (immediate freezing), and analysis (protein extraction, SDS/PAGE, RNA extraction, RT-PCR, creatine measurement) were standardized across all samples. The arbitrary assignment of a value of 1 to the "placebo preexercise" condition for reporting results further indicates a standardized measurement approach independent of the specific intervention.

4.3 – PN - The study was conducted in a double-blind fashion. This blinding typically extends to the researchers and personnel involved in handling samples and performing analyses to prevent bias, implying that outcome assessors would also be blinded to the intervention received by participants.

Algorithm result: LOW

Assessor’s judgement: LOW

5.1 NI - The study describe the statistical methods used (paired t-test, two-way ANOVA for repeated measures, Student-Newman-Keuls post hoc tests, significance threshold of P < 0.05). However, they do not explicitly state that these analyses were pre-specified or finalized before the unblinded outcome data became available. The study's double-blind crossover design suggests a rigorous methodology, but this does not directly address the timing of the analysis plan finalization.

5.2 – N - he study reports a comprehensive range of measurements including muscle creatine concentration, various gene expressions (mRNA for collagen 1, GLUT-4, MHC I, MHC IIA, MAFbx, myostatin, PCNA, PGC-1α, IL-6, calpain 1, MyoD, C2 subunit), and phosphorylation states of multiple proteins (PKB, 4E-BP1, p38, ERK1/2, p70s6k, MEF-2). The researchers present results for all defined time points (rest, immediately after exercise, 24h, and 72h post-exercise) for these outcomes. Furthermore, the paper reports on findings that did not reach statistical significance (e.g., ERK1/2 phosphorylation in the nucleus, PKB on Ser 473, and p70s6k on Thr 389). This transparency in reporting both significant and non-significant results for all measured outcomes suggests that results were not selectively chosen.

5.3 – N - The study specifies the statistical tests used for different types of comparisons: paired t-test for muscle creatine content (a within-subject comparison) and two-way ANOVA for repeated measures (appropriate for a crossover design with multiple time points). These are standard and appropriate statistical methods for the double-blind crossover study design. There is no indication in the study that multiple other eligible analyses were performed and that a specific one was selected post-hoc based on the results. The consistent application of these methods across different outcome types reinforces that the analyses were not selectively chosen.

Algorithm result: Some concerns

Assessor’s judgement: Some concerns

Overall bias:

Algorithm result: Some concerns

Assessor’s judgement: Some concerns

Cornisha & Peeler 2018

1.1 – Y – “Following baseline evaluation, participants were randomly assigned to 1 of 2 groups in a 1:1 blocked fashion using online software available at http://www.randomizer.org/: (Group 1) Participants supplemented their regular diet with creatine monohydrate (Creapure®, AlzChem AG, Trostberg, Germany) for 12 consecutive weeks. In week #1, participants ingested 5 grams of creatine monohydrate 4× per day for a total ingestion of 20 g/d. For the remaining 11 weeks, participants consumed 5 g of creatine monohydrate per day (Group 2).”

1.2 – Y - Comment: The study was conducted in a double-blind fashion, meaning that neither the participants nor the investigators knew which supplement (creatine or placebo) was being taken. This implies that the allocation sequence was concealed.

1.3 – N - The demographic data at baseline for both the creatine and placebo groups showed no statistically significant differences for age, weight, body mass index (BMI), or duration of symptoms (all P > .05). This indicates that the randomization process was effective in creating comparable groups at baseline.

Algorithm result: LOW

Assessor’s judgement: LOW

2.1 – N - The study was conducted in a double-blind fashion, meaning that neither the participants nor the investigators knew which supplement (creatine or placebo) was being taken.

2.2 – N - The study was conducted in a double-blind fashion, meaning that neither the participants nor the investigators knew who was taking what supplement.

2.6 – Y - A 2 group (creatine vs. placebo) × 2 time (baseline and 12 weeks) repeated measures analysis of variance (ANOVA) was used to analyze all dependent variable blood biomarkers, KOOS outcome scores, and isometric strength scores, which is an appropriate statistical method for this study design.

Algorithm result: LOW

Assessor’s judgement: LOW

3.1 – N - While data for the Knee Osteoarthritis Outcome Score (KOOS) was available for all randomized participants in both groups (N=9 for creatine, N=9 for placebo), and blood biomarker data was available for nearly all (N=8 for creatine, N=9 for placebo), a substantial amount of data for isometric strength measurements was missing. For strength, only 6 out of 9 participants in the creatine group and 4 out of 9 participants in the placebo group had post-intervention data. This represents 33% and 56% missing data for these respective groups and outcomes, which is not "nearly all."

3.2 – N - The study do not provide any discussion or analysis regarding the missing outcome data, particularly for the isometric strength measurements, nor do they explain how these missing data points were handled to prevent potential bias in the results. The study does acknowledge that the "small number of study participants" might have "influenced our ability to detect significant differences", but this refers to statistical power rather than specific bias from missing data.

3.3 – PY - Although the study do not explicitly state the reasons for missing data, especially the significant number for isometric strength measurements, it is plausible in a study of knee osteoarthritis that participants experiencing increased pain or greater functional limitations might be less able or willing to complete demanding strength tests. If this were the case, the missingness would directly depend on the true value of their condition (e.g., higher pain leading to lower or missing strength measurements).

3.4 – PY - Given the nature of knee osteoarthritis, where pain can inhibit maximal effort contraction, and the significant amount of missing data for the strength outcome, it is likely that participants who experienced worsening pain or greater functional decline might have been the ones for whom strength data was not obtained. This would mean the missingness in the outcome data was dependent on the true value of the participants' condition.

Algorithm result: HIGH

Assessor’s judgement: HIGH

4.1 – N - Blood biomarkers (inflammatory and cartilage degradation) were measured using ELISA kits, with blood drawn by a certified phlebotomist, analyzed in triplicate, and samples from the same participant analyzed on the same microplate to reduce variation. The intra-assay coefficient of variation for the biomarkers was low, ranging from 1.9% to 8.3%. These methods are standard for biomarker analysis; The Knee Osteoarthritis Outcome Score (KOOS) questionnaire is a self-administered, knee-specific questionnaire that is described as a valid, highly reliable, and responsive measurement tool for evaluating changes after OA interventions; Isometric thigh muscle strength was measured using a Biodex System 3 dynamometer, following a detailed protocol for participant positioning, stabilization, warm-up, maximal exertion contractions, and rest periods. These are recognized and appropriate methods for strength assessment.

4.2 – N - The study was conducted in a double-blind fashion, meaning that neither the participants nor the investigators knew which supplement (creatine or placebo) was being taken. This blinding would ensure that the methods of measurement and ascertainment of outcomes were applied consistently across both groups, without bias related to the assigned intervention. Furthermore, specific steps were taken to reduce variation in blood analyses (e.g., analyzing baseline and post-intervention samples on the same microplate).

4.3 – N - The study was double-blind, which means investigators (who would serve as outcome assessors) were not aware of the participants' assigned intervention.

Algorithm result: LOW

Assessor’s judgement: LOW

5.1 – NI - The study details the statistical methods used, stating that "A 2 group (creatine vs. placebo) × 2 time (baseline and 12 weeks) repeated measures analysis of variance was used to analyze all the dependent variable blood biomarkers, KOOS outcome scores, and isometric strength scores" and specifies the significance level and post-hoc tests. While a specific analysis plan is described, the study do not explicitly state that this plan was pre-specified or finalized before unblinded outcome data were available for analysis.

5.2 – N - The primary and secondary objectives of the study clearly listed all the outcomes measured: inflammatory and cartilage degradation biomarkers, the Knee Osteoarthritis Outcome Score (KOOS) questionnaire, and isometric thigh muscle strength. The results section then presents data for all these outcomes, including all the specific biomarkers (CRP, IL-1β, IL-6, s100 A8/A9, TNF-α, sCOMP), all KOOS subscales (Symptoms, Pain, ADL, Sport/Rec, QOL, Total Score), and isometric strength at all three tested angles for both flexion and extension. There is no indication that results were selectively reported from multiple eligible outcome measurements.

5.3 – The study explicitly states the statistical analysis approach used: "A 2 group (creatine vs. placebo) × 2 time (baseline and 12 weeks) repeated measures analysis of variance was used to analyze all the dependent variable blood biomarkers, KOOS outcome scores, and isometric strength scores". The significance level and post-hoc test (Fisher’s LSD) are also specified. This indicates a single, consistent analytical approach applied to all outcomes. There is no information in the study to suggest that multiple different analytical approaches were explored and one was selected based on the results. While there was a significant amount of missing data for isometric strength measurements, the study do not mention any specific methods for handling missing data or whether different handling methods were considered as alternative "eligible analyses."

Algorithm result: Some concerns

Assessor’s judgement: Some concerns

Overall bias:

Algorithm result: HIGH

Assessor’s judgement: HIGH

Bassit et al 2008

1.1 – Y – “Eleven triathletes, each with at least three years experience of participation in this sport were randomly divided between the control and experimental groups.” NI - Comment: The only information about the randomisation methods is a statement that the study is randomised.

1.2 – PY – Comment: The study was conducted as a double-blind trial, and the supplements were prepared to have similar volume, flavor, and color to prevent identification. This strongly suggests that neither the participants nor the researchers involved in the allocation or administration knew which group received which intervention, implying allocation concealment.

1.3 – N - Forty-eight hours before the competition, there were no significant differences between the control and experimental groups in the plasma concentrations of IL-6, TNFa, INFa, IL-1b, and PGE2. Additionally, the triathletes were homogeneous in terms of running experience and performance in a half-ironman competition based on physical data and training degree.

Algorithm result: LOW

Assessor’s judgement: LOW

2.1 – N - The study was conducted as a double-blind trial. Furthermore, the supplements (creatine or carbohydrate) were prepared with similar volume, flavor, and color to prevent identification by the athletes.

2.2 – N - As a double-blind trial, both the participants and the researchers administering the interventions would have been unaware of the assignments. The identical appearance of the supplements also supports this.

2.6 – Y - The study utilized two-way ANOVA for comparisons, followed by a Bonferroni post-hoc test for group means. A significance level of p<0.05 was chosen, and all data were analyzed using the Graph Pad Prism program. These are appropriate statistical methods for this type of experimental design.

Algorithm result: LOW

Assessor’s judgement: LOW

3.1 – Y - The study started with 11 triathletes who were randomly divided into a control group (n=6) and an experimental group (n=5). All results presented for the plasma concentrations of IL-6, TNFa, INFa, IL-1b, and PGE2 consistently show data for all 6 participants in the placebo group and all 5 participants in the creatine group at all measured time points (48 h before, 24 h after, and 48 h after competition). There is no indication of any missing data or dropouts for these outcome measures.

Algorithm result: LOW

Assessor’s judgement: LOW

4.1 – N - Plasma concentrations of IL-1b, IL-6, TNFa, INFa, and PGE2 were determined using commercially available ELISA-kits (Biotrak – cellular communication assays, Amersham Pharmacia biotech, Little Chalfont Buckinghamshire, UK). ELISA (Enzyme-Linked Immunosorbent Assay) is a standard and appropriate method for quantifying these biological markers.

4.2 – N - The study was conducted as a double-blind trial. This means that neither the participants nor the researchers involved in administering the supplements or collecting the samples were aware of the assigned intervention. The identical appearance (volume, flavor, color) of the creatine and carbohydrate supplements further prevented identification. Given that the outcomes were measured objectively using standardized ELISA kits, and the blinding was maintained, it is highly unlikely that the measurement or ascertainment of the outcome differed in a biased way between the groups.

4.3 – PN - As the study was a double-blind trial, it is implicit that those assessing the outcomes (i.e., performing the ELISA assays) were also blinded to the participants' group assignments. This is a fundamental aspect of a double-blind design to prevent bias in outcome assessment. The use of standardized commercial kits also reduces the potential for assessor influence.

Algorithm result: LOW

Assessor’s judgement: LOW

5.1 – PY - The study explicitly details the statistical methods used, including two-way ANOVA, Bonferroni post-hoc test, and a significance level of p<0.05, all analyzed using Graph Pad Prism. This comprehensive description of the analytical approach suggests a pre-specified plan. As a double-blind trial, it is standard and expected practice for the analysis plan to be finalized before the unblinding of outcome data to prevent bias, although the exact timing of this finalization is not explicitly stated. – NI Comment: They do not mention protocol registration

5.2 – N - The study clearly states its objective to investigate specific pro-inflammatory cytokines (IL-1b, IL-6, TNFa, INFa) and Prostaglandin E2 (PGE2). Blood samples were collected at three defined time points: 48 hours before, and 24 and 48 hours after competition. The results for all these pre-specified outcomes at all specified time points are presented and discussed consistently throughout the paper, with findings for each marker (including IL-6, for which no significant difference was found between groups) clearly reported. There is no indication that results were selectively reported based on their significance. -PN

5.3 – N - The "Statistical analysis" section precisely outlines the methods used: two-way ANOVA followed by a Bonferroni post-hoc test, with a p<0.05 significance level. This indicates a single, pre-determined analytical approach rather than the selection of results from multiple different analyses run after the data were collected and observed. - PN

Algorithm result: LOW – SOME CONCERNS

Assessor’s judgement: LOW - SOME CONCERNS

Overall bias:

Algorithm result: LOW

Assessor’s judgement: LOW
