# Supplementary material for: Impact of creatine supplementation on inflammation: evidence from a systematic review and meta-analysis of randomized double-blind placebo trials
Source: Front Immunol. 2026 Feb 19;17:1743603. doi: 10.3389/fimmu.2026.1743603 (PMC12961398; doi:10.3389/fimmu.2026.1743603)
Supplement: Supplementary file 2 [file SupplementaryFile1.zip › SR Creatine inflammatory markers (Kell Doutorado). /Para submeter/Frontiers in Immunology (5.9 IF)_/Table 1. Description of studies.docx]

**Table 1.** Description of the characteristics of the study population of articles by author and year, sample, age (years), intervention, control, outcomes and funding.

| **Author/**  **Years** | **Study Design** | **Sample** | **Age (years)** | **Intervention** | **Control** | **Outcomes** | **Funding** |
| --- | --- | --- | --- | --- | --- | --- | --- |
| Bassit et al 2008 | Randomized, double-blind, placebo-controlled trial. | 11 male triathletes.  Intervention group: n=5.  Control group: n=6. | Mean age: 40.3 ± 2.18 years.  Range: 34 to 56 years. | Dose: 20 grams of creatine monohydrate per day.  Duration: 5 consecutive days prior to the competition.  Form of administration: Two equal daily doses (10 a.m. and 4 p.m.), mixed with 50 g of maltodextrin powder and diluted in water. | 20 grams of carbohydrate (maltodextrin) daily, prepared and flavored identically to the creatine solution. | Creatine supplementation significantly reduced Tumor Necrosis Factor-α (TNF-α), Interleukin-1β and Prostaglandin E2 (PGE2). | Yes. |
| Cornisha & Peeler 2018 | Randomized, double-blind, placebo-controlled trial. | 18 patients with mild to moderate knee osteoarthritis.  Intervention group: n=9.  Control group: n=9 | Mean age: 57.1 ± 7.4 years.  Range: 46.7 to 65.9 years. | Dose: 20 grams per day of creatine monohydrate for the first week (loading phase). This was administered as 5 grams, 4 times per day. For the remaining 11 weeks, participants consumed 5 grams per day of creatine monohydrate (maintenance phase).  Duration: 12 consecutives weeks.  Form of administration: oral supplementation. Creatine monohydrate (Creapure®) was supplemented to participants' regular diet. | The Placebo Group (n=9) received 20 grams per day of maltodextrin for the first week (4 x 5g/day), followed by 5 grams per day of maltodextrin for the remaining 11 weeks. The study was double-blind, ensuring neither participants nor investigators knew the supplement content. | No significant differences were found in inflammatory biomarkers (C-reactive protein, interleukin-1β, interleukin-6, s100 A8/A9, tumor necrosis factor-α) between the creatine and placebo groups after 12 weeks | Yes. |
| Deldicque et al 2008 | Double-blind crossover study. | 9 health young man.  Intervention group: n=5.  Control group: n=4. | Mean age: 21.7 ± 0.55 years.  Range: Not reported. | Dose: 21 grams per day of oral creatine monohydrate, divided into three 7 grams doses.  Duration: 5 days.  Forms of administration: oral ingestion. | Maltodextrin, 21 grams per day (3 x 7g/day), administered orally during the same period as the creatine supplementation. | Creatine showed no modulatory effect on IL-6 expression. | Yes. |
| Oliveira et al 2020 | Pilot randomized, double-blind, placebo-controlled trial. Also described as a randomized, double-blind, placebo-controlled, parallel-group clinical trial. | 27 community-dwelling older adults completed the trial.  Intervention group: n=13  Control group: n=14 | Mean age: 67 ± 5 years (intervention group) and 67 ± 6 years (control group).  Range: 60 to 80 years. | Dose: 5 grams per day of creatine monohydrate.  Duration: 12 weeks.  Forms of administration: oral ingestion. On training days, consumed immediately after sessions dissolved in a beverage with 100 g of lemon-flavored maltodextrin. On non-training days, consumed immediately after lunch dissolved in a liquid of their preference. | 5 grams per day of maltodextrin, administered orally following the same protocol as the creatine group. | After 12 weeks, there were no differences between groups in any of the analyzed variables, including adiponectin, leptin, IL-6, IL-10, and CRP. | No. |
| Rawson et al 2007 | Randomized, placebo-controlled, double-blind trial. | 22 healthy, weight-trained men.  Intervention group: n=11.  Control group: n=11. | Mean age: 22.2 ± 1.3 years (Intervention group) and 22.1 ± 2.5 years (Control group).  Range: 19 to 27 years. | Dose: Loading phase: 0.3 grams per kilogram body weight per day for 5 days. Maintenance phase: 0.03 g/kg body weight/day for 5 days.  Duration: 10 days total (5 days loading + 5 days maintenance).  Form of administration: oral, encapsulated, ingested with food in 3 equal doses per day. | Placebo, administered in encapsulated form following the same dosage and frequency protocol as the creatine group. | Creatine supplementation did not reduce muscle damage or enhance recovery following a hypoxic resistance exercise challenge.  Lactate dehydrogenase and C-reactive protein did not increase following the exercise test. | Yes. |
| Santos et al 2004 | Randomized, double-blind, placebo-controlled trial. | 34 male athletes.  Intervention group: n=18  Control group: n=16. | Mean age: 25.5 ± 3.2 years.  Rage: 21.4 to 30.1 years. | Dose: 20 grams per day of creatine monohydrate, divided into 4 doses of 5g each, along with 15g of maltodextrin per dose.  Duration: 5 days prior to the 30km race.  Form of administration: oral supplementation, by diluting the powder in water. | The control group received the same amount of maltodextrin (60g total, or 15g per dose), prepared with the same flavor and color as the creatine solution to maintain blinding. | Creatine supplementation attenuated increases in prostaglandin E2 (PGE2) by 60.9%, and tumor necrosis factor-alpha (TNF-α) by 33.7% after the 30km race. | Yes. |
| Taes et al 2004 | Randomized, double-blind, placebo-controlled, crossover design. | 45 chronic hemodialysis patients completed the trial (out of 49 recruited).  Intervention group: n=45.  Control group: n=45 | Mean age: 70 ± 10 years.  Range: 35 to 88 years. | Dose: 2 grams per day of creatine monohydrate (CreaPure®).  Duration: 4 weeks per treatment period. Patients received creatine in one of two 4-week periods.  Form of administration: oral ingestion (tablets), taken daily in the evening. | Patients received placebo tablets (Fast Flo lactose) daily in the evening, following the same protocol as the creatine group, during one of the two 4-week treatment periods. | Creatine supplementation did not decrease total plasma homocysteine (tHcy) concentrations in chronic hemodialysis patients who were already receiving folic acid and vitamins B6 and B12. Plasma and red blood cell creatine levels significantly increased in the creatine-treated groups, confirming uptake. | Yes. |
| Tarnopolsky et al 2007 | Randomized, double-blind, placebo-controlled trial. | 39 community-dwelling older adults (19 men and 20 women).  Intervention group: n=21.  Control group: n=18. | Mean age: Approximately 70.9 years (calculated average from group means: Control Men 74.8 ± 6.6, Control Women 68.3 ± 4.4, Intervention Men 71.8 ± 5.2, Intervention Women 69.5 ± 3.8).  Range: 65 to 85 years. | Dose: 5 grams per day of creatine monohydrate (Neotine®) plus 6 grams per day of conjugated linoleic acid (CLA-ONE®), along with 2 grams per day of dextrose.  Duration: 6 months (24 weeks) of resistance exercise training.  Form of administration: Oral supplementation, consumed daily. | The placebo group received 7 grams per day of dextrose plus 6 grams per day of safflower oil. The supplements were indistinguishable in flavor and appearance. | There were no significant changes in IL-6 and C-reactive protein. | Yes. |

**Legend:** CLA – Conjugated Linoleic Acid; CRP – C-reactive Protein; IL-1β – Interleukin-1 beta; IL-6 – Interleukin-6; IL-10 – Interleukin-10; LDH – Lactate Dehydrogenase; PGE₂ – Prostaglandin E2; s100 A8/A9 – S100 Calcium-Binding Protein A8/A9 (Calprotectin); TNF-α – Tumor Necrosis Factor alpha; tHcy – Total Plasma Homocysteine.
