# Supplementary material for: Impact of creatine supplementation on inflammation: evidence from a systematic review and meta-analysis of randomized double-blind placebo trials
Source: Front Immunol. 2026 Feb 19;17:1743603. doi: 10.3389/fimmu.2026.1743603 (PMC12961398; doi:10.3389/fimmu.2026.1743603)
Supplement: Supplementary file 2 [file SupplementaryFile1.zip › SR Creatine inflammatory markers (Kell Doutorado). /Supplementary Files/Final References/Alexandre/Justificativa excluídos.docx]

**Pesquisa:** Impact of Creatine Supplementation on Inflammation: Evidence from a Systematic Review and Meta-Analysis

***Artigo 1 - Efeitos da terapia de ventosaterapia seca e suplementação de creatina nas respostas inflamatórias e cardiovasculares ao teste de Wingate em jogadores de handebol***

Autores: Khadijeh Irandoust, Helmi Ben Saad, Faezeh Mohammadgholiha, Morteza Taheri, Ismail Dergaa.

**P)** Os participantes possuem mais de 18 anos de idade?

( X ) Sim ( ) Não

- “The inclusion criteria were i) competitive level of at least regional level in Iran; ii) age between 18 and 25 years.”

**I)** A intervenção utilizada no estudo é creatina? Vale qualquer formato de administração?

( X ) Sim ( ) Não

- “In players with CS condition, 60 g of creatine was consumed per day in three consecutive days prior to the study (3 meals of 20 g in morning, noon, and night).”

**C)** Teve grupo controle?

(X) Sim ( ) Não

- “for multiple comparisons was used to compare the four conditions (control-condition, DCT, CS, DCT+CS).”

**O)** O estudo apresenta valores de marcadores inflamatórios (citocinas e outros marcadores) antes e após as intervenções de creatina?

( ) Sim (X) Não

- “based on the findings of our study, DCT and CS may have a positive impact on cardiovascular function, including changes in heart-rate as well as blood biomarkers, following short-term maximal test.”

**S)** O estudo é um ensaio clínico randomizado e controlado?

( ) Sim (X) Não

- “A non-randomized quasi-experimental cross-over study design was used.”

***Artigo 2 - Effects of creatine supplementation on oxidative stress and inflammatory markers after repeated-sprint exercise in humans***

Autores: Rafael Deminice, Flávia Troncon Rosa, Gabriel Silveira Franco, Alceu Afonso Jordão, Ellen Cristini de Freitas

**P)** Os participantes possuem mais de 18 anos de idade?

( ) Sim (X) Não

Placebo Creatine

- “Age (y) 17.4 ± 1.2 17.1 ± 1.4”

**I)** A intervenção utilizada no estudo é creatina? Vale qualquer formato de administração

(X) Sim ( ) Não

- “using Cr (0.3 g/kg−1)”

**C)** Teve grupo controle?

(X) Sim ( ) Não

-”the participants were divided randomly into two groups: placebo (Pla, n = 12) or Cr supplemented (Cr, n = 13).”

**O)** O estudo apresenta valores de marcadores inflamatórios (citocinas e outros marcadores) antes e após as intervenções de creatina?

(X) Sim ( ) Não

- “Cr supplementation inhibited increases in TNF-α and CRP levels and LDH activity induced by acute exercise.”

**S)** O estudo é um ensaio clínico randomizado e controlado?

(X) Sim ( ) Não

- “Cr supplementation was performed in a double-blind, randomized controlled manner.”

***Artigo 3 - Effectiveness of Creatine Supplementation on Aging Muscle and Bone: Focus on Falls Prevention and Inflammation***

Autores: Darren G Candow, Scott C Forbes, Philip D Chilibeck, Stephen M Cornish, Jose Antonio, Richard B Kreider.

**P)** Os participantes possuem mais de 18 anos de idade?

( ) Sim ( ) Não

**I)** A intervenção utilizada no estudo é creatina? Vale qualquer formato de administração

( ) Sim ( ) Não

**C)** Teve grupo controle?

( ) Sim ( ) Não

**O)** O estudo apresenta valores de marcadores inflamatórios (citocinas e outros marcadores) antes e após as intervenções de creatina?

( ) Sim ( ) Não

**S)** O estudo é um ensaio clínico randomizado e controlado?

( ) Sim ( ) Não
