# Supplementary material for: Impact of creatine supplementation on inflammation: evidence from a systematic review and meta-analysis of randomized double-blind placebo trials
Source: Front Immunol. 2026 Feb 19;17:1743603. doi: 10.3389/fimmu.2026.1743603 (PMC12961398; doi:10.3389/fimmu.2026.1743603)
Supplement: Supplementary file 2 [file SupplementaryFile1.zip › SR Creatine inflammatory markers (Kell Doutorado). /Supplementary Files/Final References/Final/Cornisha & Peeler 2018.pdf]

NUTRITION RESEARCH 51 (2018) 57–65

Available online at www.sciencedirect.com

ScienceDirect

www.nrjournal.com

No effect of creatine monohydrate supplementation on inflammatory and cartilage degradation biomarkers in individuals with knee osteoarthritis

Stephen M. Cornish [a,b,d,*], *Jason D. Peeler* [c,d]

[a] *Faculty of Kinesiology and Recreation Management, University of Manitoba, Winnipeg, Canada*
[b] *Health, Leisure, and Human Performance Research Institute, University of Manitoba, Winnipeg, Canada*
[c] *Department of Human Anatomy and Cell Sciences, University of Manitoba, Winnipeg, Canada*
[d] *Pan Am Clinic, Winnipeg, Canada*

ARTICLE INFO

Article history:
Received 30 August 2017
Revised 21 December 2017
Accepted 22 December 2017

Keywords:
Cytokines
Knee osteoarthritis
Creatine monohydrate
Inflammation
Double-blind

ABSTRACT

The study purpose was to evaluate the effectiveness of creatine monohydrate supplementation (20 grams/day for 1 week and then 5 grams/day for 11 weeks) on inflammation (C-reactive protein, interleukin-1β, interleukin-6, s100 A8/A9, and tumor necrosis factor-α) and cartilage degradation (serum cartilage oligomeric matrix protein) in patients with knee osteoarthritis. We hypothesized that supplementing with creatine monohydrate for 12 weeks would lower biomarkers of inflammation and cartilage degradation in patients with knee osteoarthritis when compared to placebo. A total of 18 patients with mild to moderate knee osteoarthritis were recruited and randomized in a double blind fashion to either a creatine supplementation group (N = 9) or a placebo (N = 9). At baseline and after 12 weeks of supplementation patients had inflammatory and cartilage degradation biomarkers measured in the systemic blood. Further, patients completed the Knee injury and Osteoarthritis Outcome (KOOS) questionnaire as well as had their isometric thigh strength evaluated using an isokinetic dynamometer at both time points. Results indicated that there was no difference between the creatine and placebo groups at 12 week follow up in the inflammatory biomarkers measured nor was there any difference between the groups for cartilage degradation (all $P > .05$). No statistical differences were noted for the KOOS questionnaire subscales or total score (all $P > .05$). Muscle strength testing indicated a main effect of time for both groups where isometric thigh strength at 0° of knee flexion was lowered significantly ($P = .047$). No other significant differences were found in the strength data. We conclude that 12 weeks of supplementation with creatine monohydrate does not affect inflammatory biomarkers, cartilage degradation, KOOS scores, or muscle strength in patients with mild to moderate knee osteoarthritis.

© 2017 Elsevier Inc. All rights reserved.

Abbreviation: ADLs, Activities of daily living; CRP, C-reactive protein; ESR, Erythrocyte sedimentation rate; IL-1β, Interleukin-1 beta; IL-6, Interleukin-6; KOOS, Knee Osteoarthritis Outcome Score; OA, Osteoarthritis; s100 A8/A9, Calprotectin; sCOMP, Serum cartilage oligomeric protein; WOMAC, Western Ontario and McMaster Universities Osteoarthritis Index.

* *Corresponding author at:* 117 Frank Kennedy Centre, Faculty of Kinesiology and Recreation Management, University of Manitoba, Winnipeg, MB, Canada, R3T 2N2. Tel.: +1 204 474 9981; fax: +1 204 474 7634.
E-mail address: Stephen.Cornish@umanitoba.ca (S.M. Cornish).

https://doi.org/10.1016/j.nutres.2017.12.010
0271-5317/© 2017 Elsevier Inc. All rights reserved.

1. Introduction

Osteoarthritis (OA) is a progressive joint degenerative condition that is estimated to affect more than 27 million North Americans and for which there is currently no known cure [1]. Chronic low-grade inflammation is highly associated with OA and has been hypothesized to be one of the driving forces behind OA progression [2]. The World Health Organization reports knee OA as the 4th and 8th most common cause of disability in women and men, respectively [3], with it occurring at a rate as high as that of cardiac disease [4]. Data suggest that knee OA is the most frequent form of OA [5], and that it has a lifetime risk of nearly one in two (46%), with overweight individuals being most susceptible [6].

Knee OA results in significant and progressively debilitating inflammation, joint pain and stiffness, muscle weakness, and decreased range of joint motion which substantially impairs one's ability to perform essential activities of daily living [7,8]. These changes severely limit mobility, adversely impact work productivity, and significantly diminish overall health and quality of life [9].

Chronic low-grade inflammation is defined as a two to four fold increase in circulating levels of pro-inflammatory markers such as C-reactive protein (CRP), tumor necrosis factor-α (TNF-α), and interleukin-6 (IL-6) [10]. In older adults with OA, it has been demonstrated that a change over 2.7 years in concentrations of CRP and TNF-α is associated with an increase in total knee pain, and TNF-α and IL-6 levels are predictive for change in knee pain at 5-year follow-up [11]. Furthermore, de Boer et al [12] indicated that some adipokines (cytokines released from adipose tissue) were associated with synovial joint inflammation in patients diagnosed with knee OA suggesting a possible role of systemic inflammation in progression of the disease while severity of pain in patients with OA was associated with an elevated level of CRP [13]. These results suggest that a low level systemic inflammatory response is a potential mechanism that results in progressive OA disease.

Recently, there has been increasing interest in creatine monohydrate supplementation in a variety of health conditions [14]. Previous work has found that creatine is anti-inflammatory in nature [15,16] and this may be one mechanism whereby it promotes health effects. In an *in vitro* model using endothelial cells, creatine has been shown to exert anti-inflammatory activities by reducing neutrophil adhesion via down regulation of intercellular adhesion molecule-1 and E-selectin [16]. Further *in vivo* work in the area of creatine supplementation has evaluated its effects on blood markers of inflammation in models of exercise designed to elicit an inflammatory response [17-19] with research indicating that creatine is able to attenuate inflammatory markers after a strenuous exercise session. Also, a study done in postmenopausal women diagnosed with knee OA found that creatine supplementation combined with lower body resistance exercise improved physical function and leg lean tissue mass more so than a placebo while at the same time decreasing joint stiffness and improving quality of life [20]. Creatine supplementation may also be beneficial for enhancing cartilage formation due to the fact that phosphocreatine can be used as an energy source in the synthesis of endochondral growth cartilage [21,22]. This demonstrates the possible benefits of using creatine supplementation to improve disease outcomes in patients with knee OA.

To our knowledge, the therapeutic role of creatine supplementation in lowering systemic biomarkers of inflammation and cartilage degradation in patients diagnosed with knee OA has not been explored. Therefore, the primary research objective of this randomized, placebo controlled trial was to compare the resting blood inflammatory biomarkers (C-reactive protein, interleukin-1β, interleukin-6, s100 A8/A9, and tumor necrosis factor-α) and cartilage degradation biomarker (serum cartilage oligomeric matrix protein) concentrations from before to after 12 weeks of creatine monohydrate supplementation in patients with knee OA. Secondary research objectives were to evaluate the effects of creatine supplementation on: (1) subjective knee function using the Knee injury and Osteoarthritis Outcome Score (KOOS) questionnaire; and (2) isometric thigh muscle strength using an isokinetic dynamometer. To accomplish these objectives, inflammatory and cartilage degradation biomarkers, KOOS scores, and isometric thigh muscle strength were measured at baseline and after 12 weeks to determine possible changes in these variables in patients with knee OA. The primary hypothesis was that creatine monohydrate supplementation would decrease the systemic biomarkers of inflammation and joint degradation when compared to a placebo supplement in individuals with mild to moderate knee OA.

2. Methods and materials

2.1. Participants

A total of 18 participants (n = 9 creatine group and n = 9 placebo group) were recruited from poster and newspaper advertisements from the general population in Winnipeg, Manitoba, Canada (see Fig. 1). Inclusion criteria included: (1) ages 45–65; (2) body mass index (BMI) over 25 kg/m^2 (defined as overweight); (3) knee pain when performing normal activities of daily living (walking, squatting, or kneeling); (4) Kellgren & Lawrence grades II & III radiographic evidence of mild to moderate knee osteoarthritis in one or both knees. Exclusion criteria included: (1) radiographic evidence of severe knee OA (Kellgren & Lawrence grade IV); (2) history of traumatic hip, knee, or ankle injury or surgery; (3) use of crutches or a walking aid during ambulation; (3) history of medical conditions that prevent physical activity; (4) Unable to provide consent due to language barrier or mental status; (5) history of diabetes, cardiovascular disease, or screen positive for ankylosing spondylitis, psoriatic arthritis, chronic reactive arthritis, or renal problems requiring peritoneal dialysis or hemodialysis; (6) unwillingness or inability to return for follow-up appointments.

Participants provided informed consent, completed participant information and knee demographic forms, and underwent radiographic evaluation to confirm eligibility. Baseline evaluation of (1) systemic inflammation and cartilage degradation; and (2) knee joint pain and function (using the KOOS, and isometric strength testing) was completed.

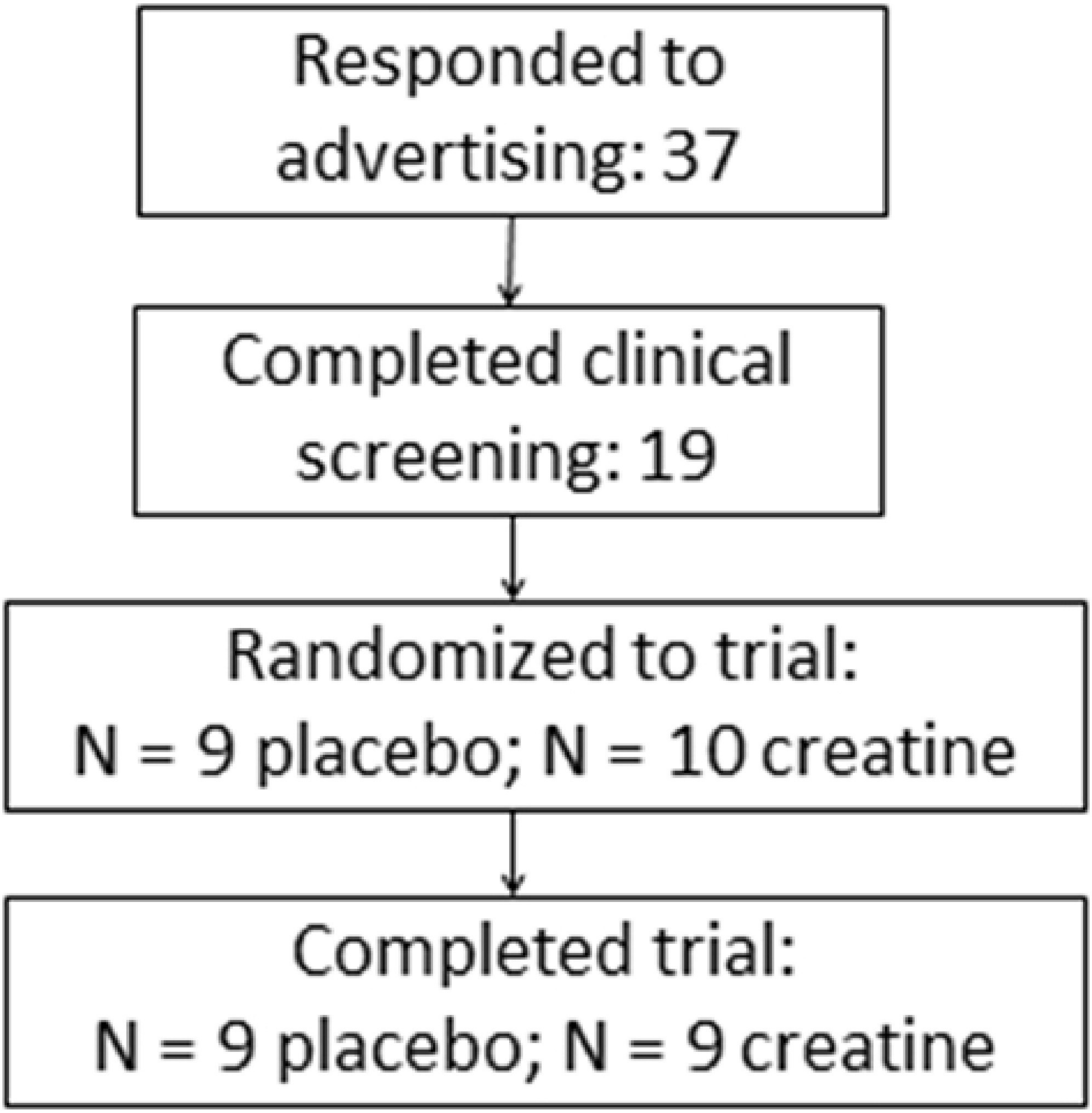

Fig. 1 – Subject recruitment flow diagram.

Following baseline evaluation, participants were randomly assigned to 1 of 2 groups in a 1:1 blocked fashion using online software available at http://www.randomizer.org/: (Group 1) Participants supplemented their regular diet with creatine monohydrate (Creapure®, AlzChem AG, Trostberg, Germany) for 12 consecutive weeks. In week #1, participants ingested 5 grams of creatine monohydrate 4× per day for a total ingestion of 20 g/d. For the remaining 11 weeks, participants consumed 5 g of creatine monohydrate per day (Group 2). Participants in the placebo group supplemented their regular diet with maltodextrin (a sugar based molecule) for 12 consecutive weeks. In week #1, participants ingested 5 g of maltodextrin 4× per day for a total ingestion of 20 g/d. They then ingested 5 g of maltodextrin per day for the remaining 11 weeks. The study was double blind where neither the participants nor the investigators knew who was taking what supplement. The study was approved by the University of Manitoba Research Ethics Board.

Table 1 – Demographic information for knee osteoarthritis patients at baseline.

	Creatine (N=9)	Placebo (N=9)	*p*-values
Gender (male/female)	5/4	5/4	-
Age (y)	57.5 ± 8.0 (46.7-65.9)	56.7 ± 6.8 (46.8-65.9)	0.824
Weight (kg)	89.1 ± 18.6 (60.5-121.5)	99.6 ± 19.0 (80.5-145.5)	0.255
BMI (kg/m^2)	29.6 ± 4.1 (25-35.5)	32.7 ± 4.4 (26.6-40.7)	0.135
Leg alignment (varus/normal/valgus)	1/5/3	1/8/0	-
Affected knee (unilateral/bilateral)	3/6	5/4	-
Duration of symptoms (mo)	155.3 ± 190.8 (18-576)	164.0 ± 130.0 (12-372)	0.913

Values are means ± SD (range). Participants were randomized to either a creatine (n = 9) supplementation group or placebo (n = 9) group. Demographic data was analyzed using an independent *t* test.

2.2. *Blood assays*

At baseline and after 12 weeks of the supplementation protocol approximately 20 mL of blood was drawn from the antecubital vein under sterile conditions by a certified phlebotomist into EDTA vacutainer blood collection tubes. The blood was centrifuged at 2500 rpm for 15 minutes to separate the plasma. Approximately 1 mL of plasma was then aliquoted into microtubes (8 × 1 mL tubes) and labeled appropriately and then frozen at –80°C until analysis. Also, approximately 5 mL of blood was drawn under the same sterile conditions by a certified phlebotomist into a serum separator vacutainer blood tube. The serum tube was allowed to clot for 30 minutes before it was centrifuged at 2500 rpm for 15 minutes to separate the serum. Serum was aliquoted into 2 × 1 mL microtubes and stored at –80°C for later analysis.

All blood was analyzed in triplicate and participants baseline and 12 week post samples were analyzed in the same microplate to reduce variation. Plasma was analyzed for a variety of cytokines including: interleukin-1 beta (IL-1β), IL-6, tumor necrosis factor alpha (TNF-α), and s100 A8/A9 using enzyme linked immunosorbent assay (ELISA) kits (R&D Systems, Minneapolis MN, USA) according to the manufacturer's instructions. Also, plasma was analyzed for the acute phase protein C-reactive protein using an ELISA kit (R&D Systems, Minneapolis MN, USA). Serum was analyzed for serum cartilage oligomeric matrix protein (sCOMP) using a commercially available ELISA kit (R&D Systems, Minneapolis, MN, USA). The "sandwich" technique was used to analyze the molecules of interest in the 96 well ELISA microplate. Capture antibodies are used to coat the 96 wells of the microplate to isolate the molecule of interest. Plasma or serum samples and standards were pipetted into each microplate well and incubated to allow antibody-sample interaction. The interaction between the molecule of interest and the capture antibodies permits the binding of the sample and antibody. Once this is complete, the microplate is washed several times to remove any debris or unattached sample. Then, each well has a secondary antibody that is conjugated to an enzyme introduced into each of the wells. During the second incubation time, the second antibody binds to the molecule of interest. Once this incubation is complete, a chromogenic substrate was added to each well which is specific to the conjugated enzyme of the second antibody; the substrate-enzyme interaction generates a color change in the well plate. The intensity of the color change indicates the quantity of the molecule of interest. The quantification of the color change is read using wavelength absorbance on a microplate reader. The sCOMP, CRP, and s100 A8/A9 assays were measured for absorbance at a wavelength of 450 nm and corrected at 540 nm while the IL-1β, IL-6, and TNF-α assays were read at a wavelength of 490 nm and corrected at 650 nm on a microplate reader (BioTek Instruments EPOCH, Winooski,

Table 2 – Baseline (pre) and 12-week (post) concentrations for inflammatory and cartilage degradation biomarkers in individuals diagnosed with mild to moderate knee osteoarthritis.

Variable	Creatine (n = 8)		Placebo (n = 9)		*P*	
	Pre	Post	Pre	Post	Time	Time x Group
CRP (ng/mL)	18.7±16.9	20.8±18.4	27.1±18.8	21.7±18.4	.544	.173
IL-6 (pg/mL)	0.9±0.6	1.1±0.6	1.9±2.2	1.1±0.6	.498	.253
sCOMP (ng/mL)	217.0±34.5	238.2±39.0	195.4±61.2	203.6±42.7	.109	.464
IL-1β (pg/mL)	0.1±0.05	0.1±0.09	0.1±0.07	0.1±0.07	.507	.941
TNF-α (pg/mL)	0.8±0.6	0.9±0.6	0.8±0.3	0.7±0.3	.963	.464
s100 A8/A9 (ng/mL)	332.9±184.4	386.9±223.8	523.8±407.8	549.7±504.8	.409	.769

Values are means ± SD. Participants were randomized to either a creatine (n = 8) supplementation group or placebo (n = 9) group. Inflammatory and cartilage degradation biomarkers were measured at two time-points: pre – before the start of the supplementation time period; and post – 12 weeks after the supplementation time period. Data were analyzed using a 2 (group) × 2 (time) repeated measures ANOVA (STATISTICA version 13.3). CRP: C-reactive protein; IL-6: interleukin-6; sCOMP: serum cartilage oligomeric matrix protein; IL-1β: interleukin-1 beta; TNF-α: tumor necrosis factor-alpha; s100 A8/A9: calprotectin

Table 3 – Baseline (pre) and 12-week (post) values for the Knees injury and Osteoarthritis Outcome Score (KOOS) questionnaire in individuals diagnosed with mild to moderate knee osteoarthritis.

Subscale	Creatine (n = 9)		Placebo (n = 9)		P	
	Pre	Post	Pre	Post	Time	Time × Group
Symptoms	52 ± 16 (32-82)	56 ± 12 (43-82)	56 ± 9 (46-75)	58 ± 23 (21-93)	.470	.822
Pain	62 ± 18 (33-89)	62 ± 15 (39-84)	66 ± 15 (44-89)	64 ± 18 (36-86)	.736	.784
ADL	68 ± 16 (38-88)	69 ± 15 (50-90)	72 ± 16 (47-94)	71 ± 22 (38-94)	.992	.648
Sport/Rec	43 ± 22 (15-75)	42 ± 26 (0-80)	50 ± 25 (25-90)	47 ± 20 (19-75)	.757	.951
QOL	43 ± 13 (25-63)	47 ± 21 (13-75)	52 ± 14 (31-75)	62 ± 20 (31-85)	.787	.272
Total Score	58 ± 14	61 ± 16	64 ± 12	62 ± 20	.860	.524

Values are means ± SD (Range). Participants were randomized to either a creatine (n = 9) supplementation group or placebo (n = 9) group. The KOOS questionnaire measured knee osteoarthritis symptoms, pain, activities of daily living, sport/recreation, and quality of life at two time-points: pre – before the start of the supplementation time period; and post – 12 weeks after the supplementation time period. Data were analyzed using a 2 (group) × 2 (time) repeated measures ANOVA (STATISTICA version 13.3). ADL: Activities of Daily Living; QOL: Quality of Life

VT). The intra-assay coefficient of variation ranged from the following for each individual biomarker: CRP: 3.1%-7.8%; sCOMP: 1.9%-2.1%; IL-1β: 3.0%-3.6%; IL-6: 5.3%-8.3%; s100 A8/A9: 2.7%-4.4%; and TNF-α: 3.3%-7.1%.

2.3. *Knee injury and Osteoarthritis Outcome Score*

Chronic knee pain, symptoms and joint function during activities of daily living (ADLs) were evaluated using the Knee injury and Osteoarthritis Outcome Score (KOOS) questionnaire. The KOOS is a self-administered, knee-specific questionnaire that is designed to measure OA patient's knee pain over the previous seven-day period [23,24]. Testing indicates that it is a valid, highly reliable and responsive measurement tool for evaluating changes after different OA interventions [23-25]. Scoring on the KOOS can range from 0 (complete disability) to 100 (no disability) and is evaluated using 5 categories: (1) Pain; (2) Symptoms; (3) Function in Daily Living; (4) Sports/Recreation; and (5) Quality of Life [23,24].

2.4. *Isometric strength*

Isometric thigh muscle strength was measured using the Biodex System 3 system (Biodex Corporation, Shirley, NY, USA). The testing protocol was performed at baseline and immediately following completion of the 12 weeks of supplementation. During testing, participants were placed in a seated position and the isometric strength of their quadriceps and hamstring muscle groups was assessed with the knee positioned in 90°, 45° and 0° degrees of flexion. Participants sat in the chair of the dynamometer and were secured to it by stabilizing straps placed over the waist, chest, and distal third of the thigh muscle. The participant's hip flexors were at an

Table 4 – Baseline (pre) and 12 week (post) thigh isometric strength values for knee flexion in individuals diagnosed with mild to moderate knee osteoarthritis

Isometric knee position	Creatine (n = 6)		Placebo (n = 4)		P	
	Pre	Post	Pre	Post	Time	Time × Group
0° Flexion (N·m)	119.8 ± 26.0 (75.0-151.6)	129.6 ± 31.5 (102.3-171.3)	153.3 ± 38.2 (111.3-200.3)	175.1 ± 51.6 (144.6-252.3)	.072	.456
45° Flexion (N·m)	165.1 ± 66.1 (104.3-294.6)	169.5 ± 44.3 (140.0-255.0)	162.7 ± 31.8 (124.3-190.3)	206.5 ± 54.3 (168.0-287.0)	.070	.125
90° Flexion (N·m)	182.9 ± 78.8 (119.0-336.3)	176.9 ± 49.2 (107.6-248.0)	161.2 ± 29.7 (132.0-190.0)	207.6 ± 43.1 (169.0-268.6)	.147	.071

Values are means ± SD (Range). Participants were randomized to either a creatine (n = 6) supplementation group or placebo (n = 4) group. The thigh muscle strength for knee flexion was measured at two time-points: pre – before the start of the supplementation time period; and post – 12 weeks after the supplementation time period. Data were analyzed using a 2 (group) × 2 (time) repeated measures ANOVA (STATISTICA version 13.3).

Table 5 – Baseline (pre) and 12 week (post) thigh isometric muscle strength for knee extension in individuals diagnosed with mild to moderate knee osteoarthritis

Isometric Knee Position	Creatine (n = 6)		Placebo (n = 4)		P	
	Pre	Post	Pre	Post	Time	Time × Group
0° Extension (N·m)	161.5 ± 51.1 (75.0-225.0)	112.0 ± 35.4 (53.3-149.3)	170.3 ± 65.1 (124.6-264.3)	151.3 ± 37.1 (115.0-202.6)	*0.047	0.327
45° Extension (N·m)	213.2 ± 41.0 (149.3-270.0)	222.0 ± 75.4 (147.6-342.3)	239.2 ± 59.8 (151.6-279.0)	258.7 ± 70.3 (157.6-319.0)	0.478	0.785
90° Extension (N·m)	239.8 ± 55.6 (163.0-307.0)	255.7 ± 88.8 (182.6-415.6)	248.2 ± 39.1 (202.6-297.3)	325.6 ± 88.6 (200.3-403.6)	0.101	0.257

Values are means ± SD (Range). Participants were randomized to either a creatine (n = 6) supplementation group or placebo (n = 4) group. The thigh muscle strength for knee extension was measured at two time-points: pre – before the start of the supplementation time period; and post – 12 weeks after the supplementation time period. Data were analyzed using a 2 (group) x 2 (time) repeated measures ANOVA (STATISTICA version 13.3). *Time main effect where pooled data revealed that isometric knee extension strength was significantly reduced in both groups at 0° of extension.

angel of approximately 80° and they sat with their backs against the back support of the chair on the dynamometer. The lateral femoral epicondyle was lined up with the rotational axis of the lever arm on the dynamometer and the lever arm was secured to the distal shin by a strap. Prior to the maximal strength testing, participants were instructed to perform the protocol at 50% effort as a warm-up, and to familiarize themselves with the equipment and testing protocol [26]. Participants were then asked to perform four maximal exertion (5 seconds in duration) contractions at each knee flexion and extension angle, with a 30-second rest period between each contraction. Both limbs were tested using the same protocol. The peak torque, measured in Newton-meters (N-m), achieved over the four repetitions was the recorded score.

2.5. *Statistical analyses*

A 2 group (creatine vs. placebo) × 2 time (baseline and 12 weeks) repeated measures analysis of variance was used to analyze all the dependent variable blood biomarkers, KOOS outcome scores, and isometric strength scores. The significance level for the study was set at $P \leq .05$. If significant interactions or main effects were found the Fisher's LSD post-hoc test was used to determine where the differences were located. All statistical analyses were performed using STATISTICA 13.3 (Tibco Software Inc, Palo Alto, CA, USA).

3. Results

Demographic information for the participants is presented in Table 1. There were no main effects for group or time or their interaction for any of the inflammatory blood biomarkers analyzed ($P > 0.05$; see Table 2). Further, there were no main effects for group or time or their interaction for the sCOMP protein ($P > .05$; see Table 2). Knee flexion and extension strength at 0°, 45°, and 90° of knee flexion or extension respectively was not significantly different ($P > 0.05$; see Tables 4 and 5) between creatine and placebo groups but there was a main effect for time where both groups decreased significantly from baseline to 12 weeks post testing for knee extension strength at 0 degrees of extension ($P = .047$; see Table 5). There were no other main effects for time ($P > .05$; see Tables 4 and 5) in the isometric muscle strength measures. All 5 parameters of the KOOS (ie, pain, symptoms, activities of daily living function, sport and recreation function, and knee related quality of life) were analyzed independently and there were no main effects for group, time or their interaction for any of the parameters analyzed (all $P > .05$; see Table 3).

4. Discussion

Our research was based on the hypothesis that creatine monohydrate supplementation could act in an anti-inflammatory manner in individuals diagnosed with mild to moderate knee OA. There were no statistically significant effects of the supplementation protocol on the biomarkers of inflammation that we measured thus, our hypothesis was not supported and thus, is rejected. Also, we analyzed isometric thigh muscle strength (of the quadriceps and hamstrings) as well as the KOOS questionnaire both before and after the intervention and found no significant effects of creatine supplementation on either of these parameters. Our results indicate that creatine supplementation by itself has no appreciable effect on inflammatory biomarkers, thigh muscle strength, or osteoarthritis outcome scores as assessed by the KOOS questionnaire in a group of individuals diagnosed with mild to moderate knee osteoarthritis.

Previous research in patients with juvenile rheumatoid arthritis supported the use of a creatine and an esterified fatty acid propriety blend for reducing indicators of inflammation including CRP and erythrocyte sedimentation rate (ESR); however, this was an open label pilot study and it was not indicated what part of the supplement was composed of creatine nor what part or type of fatty acids were included in the supplement [27]. Our results in a knee OA population

showed no effect of creatine supplementation on biomarkers of inflammation over a 12 week supplementation time period thus, not supporting the effects that Golini and Jones [27] noted. Our trial was double blinded and thus more rigorous than the open labeled trial done by Golini and Jones [27] therefore, it seems likely that creatine supplementation does not have an appreciable effect on biomarkers of inflammation in patients with knee OA.

Even though our trial did not show any effect of creatine supplementation on biomarkers of inflammation in a knee OA population, this does not negate the fact that creatine supplementation may be beneficial for patients with knee OA. A previous study performed found that 12 weeks of creatine supplementation combined with resistance training exercise improved health outcomes in post-menopausal women with knee OA [20]. In this previous study, the Western Ontario and McMaster Universities Osteoarthritis Index (WOMAC) was used before and after 12 weeks of creatine supplementation and training and showed that the creatine group significantly improved in the physical function subscale as well as decreased the scores in the stiffness subscale. Further, this study indicated that lower limb lean mass was improved more so in the creatine supplemented group when compared to the placebo and that quality of life was improved significantly more in the creatine supplemented group when compared to the placebo group [20]. These results are indicative of a positive effect of creatine supplementation on the health outcomes of patients diagnosed with knee OA. Our study evaluated if a mechanism of improvement in health outcomes might be due to the potential anti-inflammatory effects of creatine supplementation, but this does not seem to be the case. In a group of patients diagnosed with rheumatoid arthritis, creatine supplementation for 24 weeks by itself was shown to statistically improve appendicular lean tissue mass but did not improve any measures of strength or functional ability [28]. Further research in rheumatoid arthritis patients demonstrated that muscular strength was increased with creatine supplementation alone but this did not transfer to improved functional abilities; however, it did decrease disease activity in this cohort [29]. A study performed in heart failure patients randomized individuals to a creatine supplementation (5 g/d) group plus aerobic exercise 3 times per week versus a group that received no intervention [30]. In this study the results indicated a lowering of the inflammatory markers CRP and IL-6 after 8 weeks of the intervention; however, this study was confounded by the fact that the intervention consisted of exercise and creatine supplementation so it is difficult to determine if the lowering of the inflammatory biomarkers is evidence of creatine's effectiveness or the effectiveness of aerobic exercise or a combination of both. Our study only utilized creatine supplementation, albeit in a different population group, but did not demonstrate any effectiveness in the supplementation protocol in lowering systemic biomarkers of inflammation which suggests that the lowering of inflammatory biomarkers in the Hemati et al. [30] study was likely due to the exercise intervention.

The other biomarker we evaluated in this trial was serum cartilage oligomeric matrix protein (sCOMP) which is a marker of cartilage degradation. Previous research has suggested that creatine may be useful as an energy substrate for cartilage and bone formation [21]. Creatine may be effective at providing an energy substrate for developing cartilage; however, we saw no evidence of any statistical changes in sCOMP over the course of the 12-week intervention which suggests to us that during this time there were no further changes in cartilage degradation which may indicate that creatine supplementation has no effect on the loss of cartilage associated with knee OA. It is possible that a longer intervention, beyond our 12 week creatine supplementation regime, could demonstrate an attenuation of cartilage degradation or an increase in formation in the knee OA patient group but this would require further research.

Some research in athletic populations has demonstrated that creatine supplementation may be effective in attenuating the rise in inflammatory variables after an acute bout of exercise. Bassit et al. [17] found that creatine supplementation for 5 days before a triathlon race significantly attenuated the inflammatory cytokines interferon-α and IL-1β while having no effect on IL-6 24 hours after the race. Creatine supplementation for 5 days before a 30 km running race also attenuated the rise in TNF-α and prostaglandin-E2 when compared to the placebo response [19]. A study that evaluated 7 days of creatine supplementation on TNF-α and CRP response after repeated sprinting exercises found an attenuation of the inflammatory response in the creatine supplemented individuals when compared to the placebo group [18]. The attenuation in the rise of these inflammatory markers suggests that creatine may be anti-inflammatory in nature. The mechanism whereby creatine supplementation may be anti-inflammatory is unknown but it has been suggested that creatine supplementation may reduce cell death and thus the inflammatory process as a whole [17]. Some support for this idea comes from the evidence which suggests lactate dehydrogenase (LDH), as a measure of cell damage, is attenuated with creatine supplementation when using exercise to stimulate an inflammatory reaction [17,19]. Research done *in vitro* suggests that creatine may be anti-inflammatory through activation of the adenosine A2A receptors via changes in phosphocreatine concentration and ATP activation [16]. Whatever the mechanism may be, we found no evidence for a reduction of pro-inflammatory biomarkers in individuals with knee osteoarthritis. This suggests that, at least in OA of the knee, there is no appreciable effect of creatine supplementation on inflammatory biomarkers we evaluated in this study.

Other studies have evaluated the effects of creatine supplementation without combined exercise training in a variety of pathological conditions to determine changes in muscle strength and quality of life. In colorectal cancer patients creatine supplementation did not improve muscle function or quality of life over an 8 week intervention compared to placebo [31]; however, patients with chronic obstructive pulmonary disease saw an increase in muscle strength and endurance as well as health status with creatine supplementation when compared to placebo but there was no change in exercise capacity (aerobic fitness level) for this cohort [32]. Our results in knee osteoarthritis patients agree with the Norman et al. [31] study results but are contrary to the results obtained by Fuld et al. [32]. It may

be that certain diseased populations respond more readily to creatine supplementation by itself in improving muscle strength and quality of life. Additionally, patients with congestive heart failure supplementing with creatine saw no difference in VO_{2peak} (a measure of aerobic fitness level) or quality of life but did see an improvement in muscle strength [33]. Further research in fibromyalgia patients observed an increase in muscular strength in a group supplemented with creatine versus a placebo but saw no improvements in pain, cognitive function, quality of sleep, or quality of life when comparing the creatine to placebo group [34]. Also, two studies out of the same laboratory demonstrated that there was no effect on creatine supplementation at a lower dose (0.1 g/kg per day for 12 weeks) in children diagnosed with systemic lupus erythematoses on muscle function or quality of life [35] nor was there any effect in juvenile dermatomyositis patients on muscle function, body composition, or health related quality of life [36]. The results of the present study agree with the last two studies mentioned where we saw no improvement in knee related quality of life (as assessed by the KOOS questionnaire) or isometric knee flexor and extensor muscle strength in knee OA patients. We observed a significant main effect for time where both the placebo and creatine supplemented groups decreased knee extension isometric strength at 0° of knee extension. We are unsure why this main effect of time occurred but it may suggest that pain in the affected knee joint may have become worse over the 12 weeks intervention and thus could have inhibited maximal effort contraction of the knee joint using the quadriceps muscle group.

It may be that creatine supplementation by itself does little to enhance quality of life or muscle strength. A review paper indicates that in combination with resistance training, creatine supplementation seems to enhance skeletal muscle strength and hypertrophy in older adults resulting in improved quality of life in older adults [37]. A more recent review states that there is potential of creatine supplementation by itself to delay muscle atrophy, improve muscle endurance and strength, and decrease the loss of physical function in older adults [38]. However, in our study we utilized middle aged adults with mild to moderate knee osteoarthritis and we did not use resistance training combined with creatine supplementation as we wanted to evaluate the independent effects of creatine supplementation on inflammatory biomarkers, muscle strength, and the KOOS questionnaire parameters; thus, further research is warranted in the area of creatine supplementation combined with resistance training to evaluate the effects this type of intervention may have on knee osteoarthritis patients.

Finally, it is important to acknowledge the limitations of the present study. Interpretation of the data was limited by the small number of study participants. This may have influenced our ability to detect significant differences in the cohort, and future investigations should attempt to recruit a larger number of participants to further confirm whether creatine supplementation has any influence on inflammation within an osteoarthritic patient population. Beyond this, while the focus of this study was limited to specific systemic biomarkers of inflammation and cartilage degradation (and each one of these biomarkers could be influenced by a number of systemic factors), it may be more appropriate to evaluate the effect of this intervention more directly on the knee joint. While joint aspiration may be painful and invasive, the direct evaluation of biomarkers for inflammation and cartilage degradation from knee joint synovial fluid could allow the effects of a creatine supplementation protocol on knee joint health to be more accurately evaluated. Another limitation was that there was a high degree of variability in our blood biomarkers results which likely influenced our results and thus, using methods that do not have the same degree of variability may be necessary to ensure that a true effect of the supplementation protocol is found.

In conclusion, 12 weeks of creatine supplementation did not affect inflammation, sCOMP levels, isometric thigh strength, or pain and function associated with normal activities of daily living in our group of mild to moderate knee osteoarthritis patients. Future work should evaluate the potential of exercise interventions combined with creatine supplementation to improve inflammatory biomarkers and sCOMP in patients with mild to moderate knee osteoarthritis to determine if exercise is able to alter inflammation favorably in this patient population.

Acknowledgment

SMC and JDP designed, conducted, and analyzed the data in relation to this research. SMC wrote the manuscript with editorial assistance from JDP; SMC had the primary responsibility for final content of the manuscript. This study was funded by the Dr. Paul H.T. Thorlakson Research Fund from the University of Manitoba. This funding source had no role in the study design, data collection, data analysis and interpretation or in the writing of this manuscript or the decision to submit the manuscript for publication. The authors acknowledge the kind contribution of AlzChem AG for the creatine monohydrate supplements used in this study. AlzChem AG had no role in the study design, data collection, data analysis and interpretation or in the writing of this manuscript or the decision to submit the manuscript for publication. The authors wish to acknowledge the contributions of the following individuals for their participation in the data collection process: Jeremie Chase, Alison Longo, David Turczyn, and Bennett Zhang. SMC and JDP have no conflicts of interest.

REFERENCES

[1] Lawrence RC, Felson DT, Helmick CG, et al. Estimates of the prevalence of arthritis and other rheumatic conditions in the United States. Part II. Arthritis Rheum 2008;58:26–35.

[2] Stannus O, Jones G, Cicuttini F, Parameswaran V, Quinn S, Burgess J, et al. Circulating levels of IL-6 and TNF-α are associated with knee radiographic osteoarthritis and knee cartilage loss in older adults. Osteoarthritis Cartilage 2010;18:1441–7.

[3] Jordan KM, Arden NK, Doherty M, et al. EULAR Recommendations 2003: an evidence based approach to the management of knee osteoarthritis: Report of a Task Force of the

Standing Committee for International Clinical Studies Including Therapeutic Trials (ESCISIT). Ann Rheum Dis 2003;62: 1145–55.
[4] Guccione AA, Felson DT, Anderson JJ, et al. The effects of specific medical conditions on the functional limitations of elders in the Framingham Study. Am J Public Health 1994;84: 351–8.
[5] Oliveria SA, Felson DT, Reed JI, Cirillo PA, Walker AM. Incidence of symptomatic hand, hip, and knee osteoarthritis among patients in a health maintenance organization. Arthritis Rheum 1995;38:1134–41.
[6] Murphy L, Schwartz TA, Helmick CG, et al. Lifetime risk of symptomatic knee osteoarthritis. Arthritis Rheum 2008;59: 1207–13.
[7] American Academy of Orthopaedic Surgeons. Treatment of Osteoarthritis of the Knee - Evidence Based Guideline. Rosemont, Il: American Academy of Orthopaedic Surgeons; 2013 May 18. Report No.: 2nd ed.
[8] Hunter DJ, McDougall JJ, Keefe FJ. The symptoms of osteoarthritis and the genesis of pain. Rheum Dis Clin North Am 2008;34:623–43.
[9] Jinks C, Jordan K, Croft P. Osteoarthritis as a public health problem: the impact of developing knee pain on physical function in adults living in the community: (KNEST 3). Rheumatology (Oxford) 2007;46:877–81.
[10] Zanchi NE, Almeida FN, Lira FS, Rosa Neto JC, Nicastro H, da Luz CR, et al. Renewed avenues through exercise muscle contractility and inflammatory status. ScientificWorldJournal 2012;584205. https://doi.org/10.1100/2012/584205 [Epub 2012 May 3].
[11] Stannus OP, Jones G, Blizzard L, Cicuttini FM, Ding C. Associations between serum levels of inflammatory markers and change in knee pain over 5 years in older adults: a prospective cohort study. Ann Rheum Dis 2013;72:535–40.
[12] de Boer TN, van Spil WE, Huisman AM, Polak AA, Bijlsma JW, Lafeber FP, et al. Serum adipokines in osteoarthritis; comparison with controls and relationship with local parameters of synovial inflammation and cartilage damage. Osteoarthritis Cartilage 2012;20:846–53.
[13] Stürmer T, Brenner H, Koenig W, Günther KP. Severity and extent of osteoarthritis and low grade systemic inflammation as assessed by high sensitivity C reactive protein. Ann Rheum Dis 2004;63(2):200–5.
[14] Gualano B, Roschel H, Lancha-Jr AH, Brightbill CE, Rawson ES. In sickness and in health: the widespread application of creatine supplementation. Amino Acids 2012;43:519–29. https://doi.org/10.1007/s00726-011-1132-7 [Epub 2011 Nov 19].
[15] Khanna NK, Madan BR. Studies on the anti-inflammatory activity of creatine. Arch Int Pharmacodyn Ther 1978;231: 340–50.
[16] Nomura A, Zhang M, Sakamoto T, Ishii Y, Morishima Y, Mochizuki M, et al. Anti-inflammatory activity of creatine supplementation in endothelial cells in vitro. Br J Pharmacol 2003;139:715–20.
[17] Bassit RA, Curi R, Costa Rosa LFBP. Creatine supplementation reduces plasma levels of pro-inflammatory cytokines and PGE2 after a half-ironman competition. Amino Acids 2008;35: 425–31.
[18] Deminice R, Troncon Rosa F, Silveira Franco G, Afonso Jordao A, Crisinti de Freitas E. Effects of creatine supplementation on oxidative stress and inflammatory markers after repeated-sprint exercise in humans. Nutrition 2013;29: 1127–32.
[19] Santos RVT, Bassit RA, Caperuto EC, Costa Rosa LFBP. The effect of creatine supplementation upon inflammatory and muscle soreness markers after a 30km race. Life Sci 2004;75: 1917–24.
[20] Neves Jr M, Gualano B, Roschel H, Fuller R, Benatti FB, Pinto AL, et al. Beneficial effect of creatine supplementation in knee osteoarthritis. Med Sci Sports Exerc 2011;43:1538–43. https://doi.org/10.1249/MSS.0b013e3182118592.
[21] Funanage VL, Carango P, Shapiro IM, Tokuoka T, Tuan RS. Creatine kinase activity is required for mineral deposition and matrix synthesis in endochondral growth cartilage. Bone Miner 1992;17:228–36.
[22] Wallimann T, Hemmer W. Creatine kinase in non-muscle tissues and cells. Mol Cell Biochem 1994;133-134:193–220.
[23] Roos EM, Lohmander LS. The Knee injury and Osteoarthritis Outcome Score (KOOS): from joint injury to osteoarthritis. Health Qual Life Outcomes 2003;1:64.
[24] Roos EM, Roos HP, Lohmander LS, Ekdahl C, Beynnon BD. Knee injury and Osteoarthritis Outcome Score (KOOS)—development of a sefl-adminsitered outcome measure. J Orthop Sports Phys Ther 1998;28:88–96.
[25] Roos EM, Roos HP, Lohmander LS. WOMAC Osteoarthritis Index—additional dimensions for use in subjects with post-traumatic osteoarthritis of the knee. Western Ontario and MacMaster Universities. Osteoarthritis Cartilage 1999;7: 216–21.
[26] Segal NA, Glass NA, Felson DT, Hurley M, Yang M, Nevitt M, et al. Effect of quadriceps strength and proprioception on risk for knee osteoarthritis. Med Sci Sports Exerc 2010;42:2081–8.
[27] Golini J, Jones WL. Kre-Celazine(®) as a viable treatment for juvenile rheumatoid arthritis/juvenile idiopathic arthritis – a pilot study. J Med Food 2014;17(9):1022–6. https://doi.org/10.1089/jmf.2013.0169 [Epub 2014 Jun 4].
[28] Wilkinson TJ, Lemmey AB, Jones JG, Sheikh F, Ahmad YA, Chitale S, et al. Can creatine supplementation improve body composition and objective physical function in rheumatoid arthritis patients? A randomized controlled trial. Arthritis Care Res (Hoboken) 2016;68:729–37. https://doi.org/10.1002/acr.22747.
[29] Willer B, Stucki G, Hoppeler H, Brühlmann P, Krähenbühl S. Effects of creatine supplementation on muscle weakness in patients with rheumatoid arthritis. Rheumatology (Oxford) 2000;39:293–8.
[30] Hemati F, Rahmani A, Asadollahi K, Soleimannejad K, Khalighi Z. Effects of complementary creatine monohydrate and physical training on inflammatory and endothelial dysfunction markers among heart failure patients. Asian J Sports Med 2016;5(7(1)):e28578. https://doi.org/10.5812/asjsm.28578 [eCollection 2016 Mar].
[31] Norman K, Stübler D, Baier P, Schütz T, Ocran K, Holm E, et al. Effects of creatine supplementation on nutritional status, muscle function and quality of life in patients with colorectal cancer—a double blind randomised controlled trial. Clin Nutr 2006;25(4):596–605 [Epub 2006 May 15].
[32] Fuld JP, Kilduff LP, Neder JA, Pitsiladis Y, Lean ME, Ward SA, et al. Creatine supplementation during pulmonary rehabilitation in chronic obstructive pulmonary disease. Thorax 2005;60:531–7.
[33] Kuethe F, Krack A, Richartz BM, Figulla HR. Creatine supplementation improves muscle strength in patients with congestive heart failure. Pharmazie 2006;61:218–22.
[34] Alves CR, Santiago BM, Lima FR, Otaduy MC, Calich AL, Tritto AC, et al. Creatine supplementation in fibromyalgia: a randomized, double-blind, placebo-controlled trial. Arthritis Care Res (Hoboken) 2013;65:1449–59. https://doi.org/10.1002/acr.22020.
[35] Hayashi AP, Solis MY, Sapienza MT, Otaduy MC, de Sá Pinto AL, Silva CA, et al. Efficacy and safety of creatine supplementation in childhood-onset systemic lupus erythematosus: a randomized, double-blind, placebo-controlled, crossover trial. Lupus 2014;23:1500–11. https://doi.org/10.1177/0961203314546017 [Epub 2014 Aug 18].
[36] Solis MY, Hayashi AP, Artioli GG, Roschel H, Sapienza MT, Otaduy MC, et al. Efficacy and safety of creatine supplementation in juvenile dermatomyositis: A

randomized, double-blind, placebo-controlled crossover trial. Muscle Nerve 2016;53:58–66. https://doi.org/10.1002/mus.24681 [Epub 2015 Nov 23].

[37] Dalbo VJ, Roberts MD, Lockwood CM, Tucker PS, Kreider RB, and Kerksick CM. The effects of age on skeletal muscle and the phosphocreatine energy system: can creatine supplementation help older adults. Dyn Med 2009; 24;8:6. doi: https://doi.org/10.1186/1476-5918-8-6.

[38] Moon A, Heywood L, Rutherford S, Cobbold C. Creatine supplementation: can it improve quality of life in the elderly without associated resistance training? Curr Aging Sci 2013;6:251–7.
