# Supplementary material for: Impact of creatine supplementation on inflammation: evidence from a systematic review and meta-analysis of randomized double-blind placebo trials
Source: Front Immunol. 2026 Feb 19;17:1743603. doi: 10.3389/fimmu.2026.1743603 (PMC12961398; doi:10.3389/fimmu.2026.1743603)
Supplement: Supplementary file 2 [file SupplementaryFile1.zip › SR Creatine inflammatory markers (Kell Doutorado). /Supplementary Files/Final References/Kell/Irandoust et al 2022.pdf]

Effects of dry cupping therapy and creatine supplementation on inflammatory and cardiovascular responses to the Wingate test in handball players

Effets de la thérapie par ventouses sèches et de la supplémentation en créatine sur les réponses inflammatoires et cardiovasculaires après le test Wingate chez des handballeurs

Khadijeh Irandoust1, Helmi Ben Saad2, Faezeh Mohammadgholiha1, Morteza Taheri1, Ismail Dergaa3

1. *Imam Khomeini International University, Qazvin, Iran*
2. *University of Sousse, Faculty of Medicine of Sousse, Hospital Farhat HACHED, Research laboratory "Heart failure, LR12SP09", Sousse, Tunisia.*
3. *Primary Health Care Corporation (PHCC), Doha, Qatar*

Abstract

Aim: To evaluate the effects of dry cupping therapy (DCT) and creatine supplementation (CS) on cardiovascular and inflammatory responses to the Wingate test.

Methods: In this quasi-experimental study, 12 male handball young players were selected in a crossover design. Players were studied in four conditions: DCT; CS; CS+DCT, control. In all conditions, blood pressure, heart-rate, and body composition were measured pre- and post- Wingate test. Players were assessed by the Wingate test in two 30-second phases with a 1-minute break between the phases. Blood [lactate-dehydrogenase (LDH), creatine phosphokinase (CK)] was drawn pre- and immediately post- the Wingate test. In players with CS condition, 60 g of creatine was consumed per day in three consecutive days prior to the study (3 meals of 20 g in morning, noon, and night). The DCT was performed after Wingate test to consider its possible effects for alleviating the muscle injury markers. Data were evaluated using analysis of covariance followed by a post-hoc Bonferoni test.

Results: The heart-rate' means in DCT, CS and CS+DCT conditions were lower compared to the control-condition ($p<0.05$). The CK' means in DCT and CS+DCT conditions were lower compared to the control-condition ($p<0.05$). The mean systolic and diastolic blood pressure and LDH in the four conditions were similar ($p>0.05$).

Conclusion: DCT and CS lead to beneficial effects on cardiovascular function, including changes in heart-rate as well as blood biomarkers among handball players following the Wingate test.

Key words : Anaerobic Test; Al-hijama; Creatine Complement; Lactic Acid; Traditional Medicine; Alternative Medicine; Complementary Medicine.

Résumé

Objectif: Évaluer les effets de la thérapie par ventouses sèches (TVS) et la supplémentation en créatine (SC) sur les réponses cardiovasculaires et inflammatoires au test de Wingate.

Méthodes: Dans cette étude quasi-expérimentale, 12 jeunes handballeurs masculins étaient sélectionnés selon un plan croisé. Les joueurs étaient étudiés dans quatre conditions: TVS; SC; SC+TVS, contrôle. Dans toutes les conditions, la pression artérielle, la fréquence cardiaque et la composition corporelle étaient mesurées avant et après le test de Wingate. Les handballeurs étaient évalués par le test Wingate en deux phases de 30 secondes avec une pause d'une minute entre les phases. Le sang [lactate-déshydrogénase (LDH) et créatine phosphokinase (CPK)] était prélevé avant- et immédiatement après- le Wingate test. Dans la condition SC, 60 g de créatine étaient consommés par jour pendant trois jours consécutifs avant l'étude (3 repas de 20 g matin, midi, et soir). La TVS était réalisée après le Wingate test pour examiner si elle atténue les marqueurs de lésions musculaires. Une analyse de covariance suivie d'un test de Bonferoni post-hoc étaient réalisées.

Résultats: Les moyennes de la fréquence cardiaque des conditions TVS, SC et SC+TVS étaient significativement plus basses comparativement à la condition-contrôle ($p<0,05$). Les moyennes de la CPK dans les conditions TVS et SC+TVS étaient significativement plus basses comparativement à la condition-contrôle ($p<0,05$). Les moyennes des pressions artérielles systolique et diastolique et de la LDH des quatre conditions étaient similaires ($p>0,05$).

Conclusion: La TVS et la SC entraînent des effets bénéfiques sur les fonctions cardiovasculaires, notamment des modifications de la fréquence cardiaque ainsi que les marqueurs biochimiques du sang chez les jeunes handballeurs après le test de Wingate.

Mots clés : Acide lactique; Al-hijama; Complément de créatine; Médecine Alternative, Médecine traditionnelle; Test anaérobie.

Correspondance

Khadijeh Irandoust
Imam Khomeini International University, Qazvin, Iran
Email: Irandoust@soc.ikiu.ac.ir

This article is distributed under the terms of the Creative Commons Attribution-NonCommercial-NoDerivatives 4.0 International License (CC BY-NC-ND 4.0) which permits non-commercial use production, reproduction and distribution of the work without further permission, provided the original author and source are credited.

INTRODUCTION

Athletic performance enhancement has long been of interest to athletes and coaches (1-4). Studies reported that sports nutrition and recovery are becoming increasingly important to the high-performing athlete in a bid to reduce fatigue and enhance athlete performance (5-7). Given the role of the recovery phase in various physiological adaptations in the neuromuscular, cardiovascular and respiratory systems of the human body, a great deal of research has been conducted to find out how each athlete should go through this phase at the end of each specific exercise (8, 9). Several studies highlighted that insufficient recovery would result in complications such as chronic fatigue, illness, and overtraining in athletes, which often have an impact on both the athlete's health and mental state (10, 11). Since skeletal muscle is the main tissue involved in physical activity, performing studies about changes and injuries resulting from physical activity has always been of interest (12).
The positive role of supplementation on athletic performance is well documented (13, 14). For instance, a great deal of research has shown that creatin supplementation (CS) would improve athletic performance (13, 14). Creatine monohydrate is perhaps one of the most widely used supplements taken in an attempt to improve athletic performance. According to various studies (15-18), muscle damage can lead to impaired plasma enzyme activities. Several studies (19, 20) have confirmed the relationship between exercise-induced histological muscle damage and muscle enzyme release. Muscle damage may occur in response to various stimuli resulting from prolonged strenuous exercise (21). Studies identified that active recovery is more effective than passive one in reducing blood lactate levels and improving other physiological conditions of the body (22). Creatine phosphokinase (CK) and lactate dehydrogenase (LDH) indices are considered as indicators of cell damage assessment (23, 24). Muscle injury and pain are common experiences after intense physical activity (25-27). Indicators of cell damage include morphological changes in tissues, decreased levels of function, inflammation, delayed contusion, and the activities of CK and LDH (28). Hence, it is necessary to measure recovery-related physiological factors during training or competition in athletes. On the one hand, CK is one of those enzymes, which increase following heavy exercises and causes cell damage (29). CK is an important enzyme for muscle cell metabolism (30). On the other hand, LDH is an enzyme that converts lactate to pyruvate by which the coenzyme nicotinamide adenine dinucleotide (NAD) is converted to NAD-hydrogen (NADH) (31). This enzyme is highly distributed in the cytoplasm of all body tissues in various concentrations as well as in red blood cells (32). Increasing the production of LDH and lactate acid during exercise in non-trained individuals compared to trained ones is an important point (33). The use of sports supplements has become widespread in today's society (4, 5, 34-36). Many supplements have a psychological effect on people, and some supplements, such as CS, which is used in training and competitions would probably improve athletic performance by delaying fatigue and increasing lactate tolerance (37). One of the most commonly used supplements among athletes is CS, which is used for energy purposes (38). On the one hand, CS increases creatine, which plays an important role in the rapid accumulation of ADP produced by ATP hydrolysis, especially in type II fibers (39). On the other hand, CS increases phosphocreatine regeneration during recovery and after high-intensity exercise (40).
Despite the existence of modern and up-to-date methods for athletes' recovery, it is observed that athletes are also inclined to traditional methods such as dry cupping therapy (DCT). The latter is a traditional way to reduce muscle pain and fatigue in athletes (23). DCT is also used to keep hemostasis in body (41). The results of some studies identified that exercise should be performed in such a way to maximize the performance beside reduction of lactate production (42). In this regard, the relationship between performance improvement and some cardiovascular factors such as heart-rate and blood pressure regulation is also crucial (43-45).
Since the therapeutic effects of DCT as well as CS in athletes have been well proven (41, 46), the aim of this study was to investigate the effect of these two interventions as well as their combination on physiological factors (ie; inflammatory and cardiovascular responses) associated with muscle injury. Therefore, faster heart-rate recovery may lead to faster recovery of blood lactate. One of these ways is DCT that is used to reduce muscle pain and relieve excessive fatigue in athletes (23). In addition, CS has been common in recent years to increase performance, improve health, and improve recovery (40).

METHODS

Study design and participants

A non-randomized quasi-experimental cross-over study design was used. Twelve participants from the males' handball team of Qazvin province (Iran) between the ages of 18 to 25 years voluntarily participated. The participants were given all information about the study procedure, and they signed the consent form. The research was approved by the local ethics committee Imam Khomeini International University (reference number 17628).

Inclusion, non-inclusion and exclusion criteria

The inclusion criteria were i) competitive level of at least regional level in Iran; ii) age between 18 and 25 years; iii) minimum of 12 hours of total training volume per week on average. Players weren't included if they had any infectious disease during the last three-month (due to the coronavirus disease-19 (COVID-19) pandemic). Being absent at any level of the whole study protocol was considered as an exclusion criterion.

Procedures

Figure 1 illustrates the study procedures. Body composition analyzer (Model, In Body 320) was used to measure percent body fat and weight. The resting systolic and diastolic blood pressure (SBP, DBP, respectively) and heart-rate were measured in a sitting position, after a minimum five-minute rest, by a blood pressure device (ALPK2, model 300-V-EU,

Japan) and a Polar V800 HR monitor (47), respectively. Levels of LDH and CK were respectively measured by DGKC and CK-NAC kits. Blood sampling was determined by a specific Enzyme-Linked ImmunoSorbent Assay (ELISA). A Monark cycle was used to estimate anaerobic power and capacity by Wingate test (2, 48). Three-day food recall questionnaire (49) was used to monitor the food programs. All measures were taken prior to the study (height, body weight, body mass index, body circumference, and body composition). Then, the intervention period began, which was performed according to the research plan in the three conditions of interventions (DCT, CS, DCT+CS). After the interventions, immediately post-test measurements were performed again for all aforementioned parameters. All testing sessions were performed indoors at the same time of the day (ie; between 7.00 and 8.30) to minimize the effects of diurnal variations in the aerobic and anaerobic contribution to physical performance (26, 50).

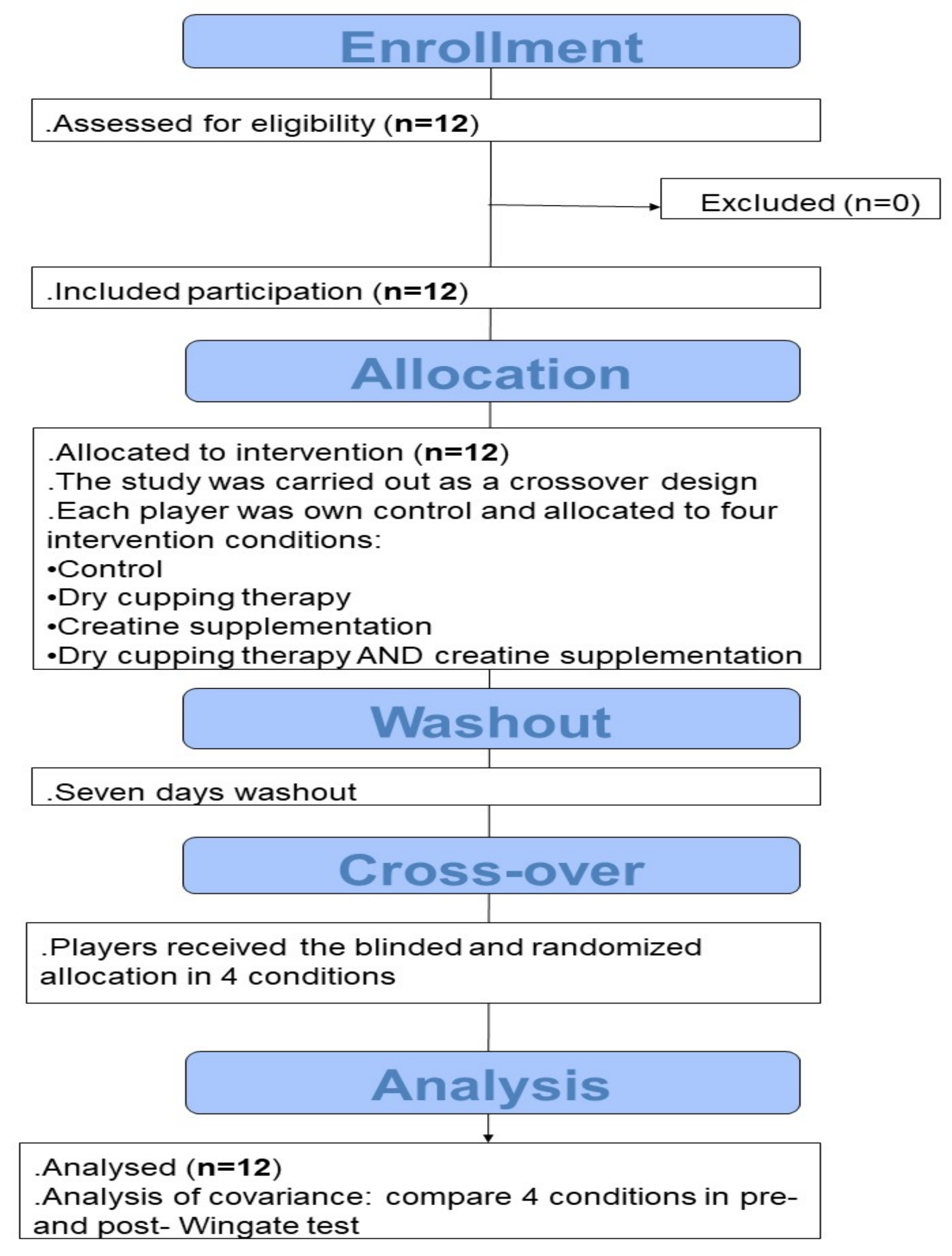

Figure 1. Study procedures

Data analysis

Quantitative data were normally distributed, and therefore were expressed as mean±standard deviation (SD). The analysis of covariance (ANCOVA) with Bonferroni's correction for multiple comparisons was used to compare the four conditions (control-condition, DCT, CS, DCT+CS). Statistical significance was set at P <0.05

RÉSULTATS

A total sample of 12 participants was retained. The 12 participants were divided into four conditions: control, DCT, SC, and SC+DCT. The total sample mean±SD age, height, weight, body mass index, lean body mass, and body fat, were respectively, 20±2 years, 186±3 cm, 91±9 kg, 26.3±2.6 (kg/m2), 70.3±4.1 kg, and 23.3±4.2 kg.

Table 1 exposes the results of ANCOVA for the four test conditions for the inflammatory and cardiovascular data. As it can be seen in table 1 and figure 2, means of SBP and DBP were similar in the four conditions (p>0.05). ANCOVA test suggested a significant difference in heart-rate for the four conditions (p<0.05) (Table 1). According to the Bonferoni test (Figure 3), means heart-rate in DCT, CS and CS+DCT conditions had significant decreases compared to the control-condition (p<0.05). As a result, DCT, CS, and DCT+CS lead to a decrease in heart-rate of participants following the Wingate test. There was a significant difference in CK in the four conditions (Table 1). According to figure 4, means of CK in the DCT and the CS+DCT conditions had significant decreases compared to the control-condition (p<0.05). Therefore, DCT have a positive effect on CK reduction in players following the Wingate test. However, mean CK was not significantly different in CS and control-condition (p>0.05), and mean CK in the DCT and CS+DCT conditions were not significantly different (p≥0.05). LDH' means were similar in the four conditions (p>0.05) (Table 1). Therefore, DCT and CS have no effect on LDH in players following the Wingate test (Figure 5).

Table 1. Results of analysis of covariance for four test conditions for systolic and diastolic blood pressure, heart-rate, creatinine phosphokinase, and lactate dehydrogenase (n=12).

Subscales	Source	Sum of squares	Degrees of freedom	Mean squares	Fisher-tests	p-value	Partial regression coefficient	Power
Systolic blood pressure (mmHg)	Pre-test	16.11	1	16.11	8.31	0.006	0.16	0.81
	Condition	5.47	3	1.82	0.94	0.430	0.06	0.24
	Error	83.39	43	1.94				
Diastolic blood pressure (mmHg)	Pre-test	0.02	1	0.02	0.06	0.813	0.01	0.06
	Condition	0.54	3	0.18	0.50	0.684	0.03	0.14
	Error	15.75	43	0.37				
Heart-rate (bpm)	Pre-test	9.08	1	9.08	0.02	0.883	0.01	0.05
	Condition	12766.43	3	4255.48	10.23	<0.001	0.42	0.99
	Error	17880.14	43	415.82				
Creatinine phosphokinase (U/L)	Pre-test	4100.80	1	41005.80	20.83	<0.001	0.33	0.99
	Condition	26494.17	3	8831.39	4.49	0.005	0.24	0.85
	Error	84667.02	43	1969.01				
Lactate dehydrogenase (U/L)	Pre-test	9252.93	1	95527.93	704.7	<0.001	0.62	0.99
	Condition	3030.77	3	1010.59	0.77	0.517	0.05	0.20
	Error	5645.96	43	1313.02				

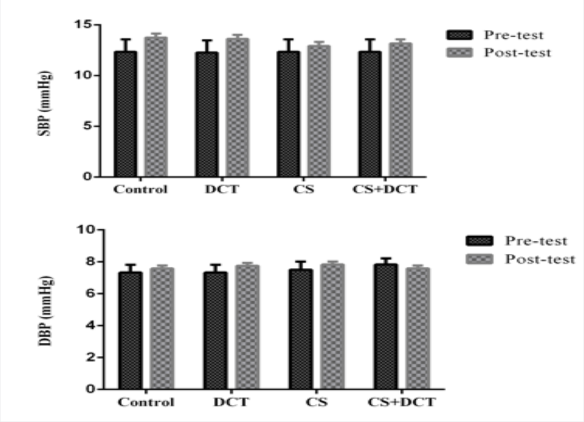

Figure 2. Bonferroni test results for systolic (SBP) and dyastolic (DBP) blood pressure (n=12).
CS: creatine supplementation. DCT: dry cupping therapy)

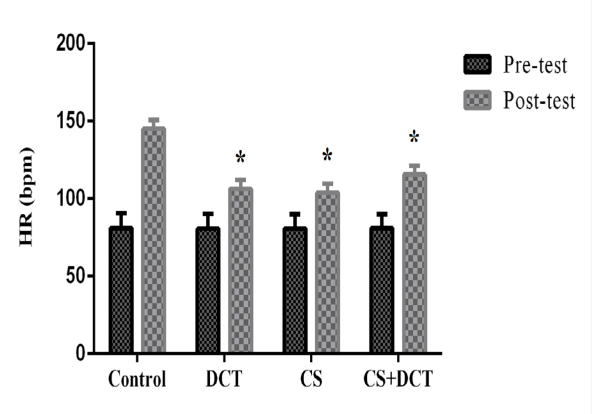

Figure 3. Bonferroni test results for heart-rate (HR) (n=12).
CS: creatine supplementation. DCT: dry cupping therapy.*p<0.05: Pre-test vs. Post-test

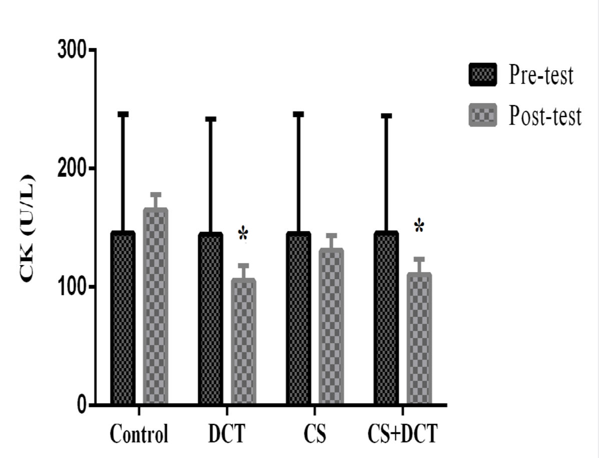

Figure 4. Bonferroni test results for creatinine phosphokinase (CK) (n=12).
CS: creatine supplementation. DCT: dry cupping therapy. *p<0.05: Pre-test vs. Post-test

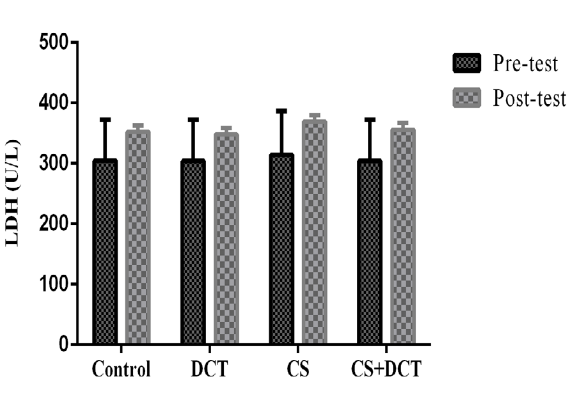

Figure 5. Bonferroni test results for lactate dehydrogenase (LDH) (n=12).
CS: creatine supplementation. DCT: dry cupping therapy

DISCUSSION

The main results of the present study including 12 male handball players evaluated in four conditions (control-condition, DCT, CS, and CS+DCT) were that compared to the control-condition, there was a significant decrease of mean heart-rate in DCT, CS and CS+DCT conditions, and mean CK in DCT and CS+DCT conditions.

Many athletes use contributing factors, such as different training protocols, nutrition plan and food supplements to enhance and improve athletic performance during competition (5, 51). The use of sports supplements has gained so much interest in today's society (46). Despite the fact that supplements have a psychological effect on athletes, some supplements can possibly delay fatigue, and increase lactate tolerance and improve athletic performance (48). Creatine is a protein compound consisting of the three amino acids methionine, arginine and glycine (46). This substance is formed in the body as a phosphate compound (creatine phosphate) and is used as a source of energy storage, especially in activities and sports of speed and explosions (52). Most creatine storage is located in skeletal muscles (46). This compound has been used as a supplement in various sports for many years, and studies in recent years show that CS can increase the amount and content of creatine in the muscles (53). With the onset of oxidant stress, the body's antioxidant activity becomes more active, and the use of some antioxidant supplements reduces the body's antioxidant activity (54-58). The findings of our study generally showed that DCT and CS had an effect on the heart-rate of young male handball players following the Wingate test (Table 1). Furthermore, DCT also had an effect on CK in young male handball players following the Wingate test (Table 1). There are conflicting opinions about the effects of creatine monohydrate supplementation on human health indicators (46, 53). For example, Banerjee et al. (59) reported that creatine monohydrate supplementation significantly reduced CK enzyme levels, while in contrast Roseno et al. (60) suggested that CS did not increase any of the serum markers of cell damage (ie;

CK and LDH). While one study reported that CS had no significant effect on cell damage markers (ie; CK and LDH) (61), Atashk et al. (62) identified no significant effects by examining and comparing untrained and trained females (running on a treadmill with 70% of maximal oxygen consumption). Finally, Lin et al. (63) reported a significant increase in CK, LDH, lactate and uric-acid levels in trained participants. Since creatine inhibits the release of enzymes out of the cell membrane by increasing membrane stability, it can be concluded that creatine intake may inhibit the increase in CK activity (62). Regarding the effects of cupping therapy, in most sports, the volume and intensity of pressure on athletes is so great that it causes musculoskeletal injuries, waste accumulation, depletion of energy, and also disrupts the mechanism of the immune system (64). Therefore, recovery-related tasks are as important as physical activity, because insufficient recovery of the body's functional capacities during training or competition activities reduces the athlete's ability to work (65).

DCT is a treatment method, which is performed by creating suction in certain parts of the body and drawing blood from them (41). Cupping therapy removes excess fluid, transmits blood flow to the skin and muscles, and stimulates the peripheral nervous system, as well as reducing pain (41). In a study of 30 gymnasts, it was reported that 30 minutes of cupping therapy resulted in a faster return to elevated CK levels (66). In general, according to the findings of our study regarding the simultaneous use of DCT and CS, it is clear that this method works for athletes. One of the possible mechanisms of cupping effect from a medical point of view could be the endocrine regulation and its effect on sympathetic and parasympathetic systems (66, 67). Similarly, in inflammation, different cytokines of skin keratinocytes are hidden as well (66). These cytokines can cause changes in cell surface receptors, which can facilitate the healing process (67). It is hypothesized that creatine can act though a number of possible mechanisms as a potential ergogenic aid, but it appears to be most effective for activities that involve repeated short bouts of high-intensity physical activity.

Considering all aspects of the issue, research has not yet found side effects for the combined use of DCT and CS. This study has some limitations. The first one is the small number of participants as well as the cross-sectional design of the research due to the lack of access to more participants due to COVID-19 constraints. It is recommended to use more participants in future research to increase the generalizability of our results. The second limitation is the lack of monitoring of nutrition, emotions and other psychological factors, and limiting the age range are other effective factors that should be taken into consideration in future studies. The third limitation is that the cardiovascular outcome measures were sparse (heart-rate and blood pressure). The fourth limitation is the lack of a serial timed biomarker measuring point during recovery. The latter technique enables access to the biomarker kinetics during the recovery phase. The fifth limitation is the lack of data for plasma catecholamine concentrations. It has been reported that increased sympathetic stimulation (ie; tachycardia) is shown by the increase in plasma catecholamine concentrations (17). The sixth limitation was that all participants were male, and therefore it is not possible to generalize our results. Finally, our study only looked at acute responses, although the literature does not support acute hormonal effects on long-term alterations. Our findings should be taken with caution, and further longitudinal monitoring-based research is needed to better understand how DCT and CS can affect the inflammatory and cardiovascular responses to intense physical exercise.

To conclude, based on the findings of our study, DCT and CS may have a positive impact on cardiovascular function, including changes in heart-rate as well as blood biomarkers, following short-term maximal test.

Conflict of interests

The authors declare that there is no conflict of interests.

Acknowledgments

Thanks to all the athletes who participated in the study.

REFERENCES

1. Taheri M, Irandoust K, Noorian F, Bagherpour F. The effect of aerobic exercise program on cholesterol, blood lipids and cigarette withdrawal behavior of smokers. Acta Medica Mediterranea. 2017;33(4):597-600.
2. Paryab N, Taheri M, H'Mida C, Irandoust K, Mirmoezzi M, Trabelsi K, et al. Melatonin supplementation improves psychomotor and physical performance in collegiate student-athletes following a sleep deprivation night. Chronobiol Int. 2021;38(5):753-61.
3. Irandoust K, Taheri M, Chtourou H, Nikolaidis PT, Rosemann T, Knechtle B. Effect of time-of-day-exercise in group settings on level of mood and depression of former elite male athletes. Int J Environ Res Public Health. 2019;16(19):3541.
4. Taheri M, Irandoost K, Yousefi S, Jamali A. Effect of 8-week lower extremity weight-bearing exercise protocol and acute caffeine consumption on reaction time in postmenopausal women. Salmand. 2017;12(1):16-27.
5. Amirsasan R, Nabilpour M, Pourraze H, Curby D. Effect of 8-week resistance training with creatine supplementation on body composition and physical fitness indexes in male futsal players. Int J Sport Stud Hlth. 2018;1(3):e8381.
6. Jahani Golbar S, Gharekhanlu R, Kordi MR, Khazani A. Effect of Endurance exercise training on kinesin - 5 and dynein motor proteins in sciatic nerves of male Wistar rats with diabetic neuropathy. Int J Sport Stud Hlth. 2018;1(1):e67758.
7. Asadi A, Ghasemi M, Zarandi E, Khanjari MM, Bayat S, Malekmohammadi S. Effects of combined resistance-aerobic training and milk consumption on the weight loss of overweight female students. Int J Sport Stud Hlth. 2019;2(2).
8. Harris NK, Woulfe CJ, Wood MR, Dulson DK, Gluchowski AK, Keogh JB. Acute physiological responses to strongman training compared to traditional strength training. J Strength Cond Res. 2016;30(5):1397-408.
9. Harbili S. The Effect of different recovery duration on repeated anaerobic performance in elite cyclists. J Hum Kinet. 2015;49(1):171-8.

10. Souter G, Lewis R, Serrant L. Men, mental health and elite sport: a narrative review. Sports Med Open. 2018;4(1):57.

11. Gleeson M. Biochemical and immunological markers of over-training. J Sports Sci Med. 2002;1(2):31-41.

12. Nader GA, Esser KA. Intracellular signaling specificity in skeletal muscle in response to different modes of exercise. J Appl Physiol (1985). 2001;90(5):1936-42.

13. Buford TW, Kreider RB, Stout JR, Greenwood M, Campbell B, Spano M, et al. International society of sports nutrition position stand: creatine supplementation and exercise. J Int Soc Sports Nutr. 2007;4(1):1-8.

14. Burke LM. Caffeine and sports performance. Appl Physiol Nutr Metab. 2008;33(6):1319-34.

15. Kuipers H. Exercise-induced muscle damage. Int J Sports Med. 1994;15(3):132-5.

16. Tee JC, Bosch AN, Lambert MI. Metabolic consequences of exercise-induced muscle damage. Sports Med. 2007;37(10):827-36.

17. Souissi A, Haddad M, Dergaa I, Saad HB, Chamari, K. A new perspective on cardiovascular drift during prolonged exercise. Life Sci. 2021;287:120109.

18. Souissi A, Souissi N, Dabboubi R, Souissi N. Effect of melatonin on inflammatory response to prolonged exercise. Biol Rhythm Res. 2020;51:560-5.

19. Pyne DB. Exercise-induced muscle damage and inflammation: a review. Aust J Sci Med Sport. 1994;26(3-4):49-58.

20. Romero-Parra N, Cupeiro R, Alfaro-Magallanes VM, Rael B, Rubio-Arias JA, Peinado AB, et al. Exercise-induced muscle damage during the menstrual cycle: A systematic review and meta-analysis. J Strength Cond Res. 2021;35(2):549-61.

21. Martin-Rincon M, Gelabert-Rebato M, Galvan-Alvarez V, Gallego-Selles A, Martinez-Canton M, Lopez-Rios L, et al. Supplementation with a mango leaf extract (Zynamite®) in combination with quercetin attenuates muscle damage and pain and accelerates recovery after strenuous damaging exercise. Nutrients. 2020;12(3):614.

22. Nalbandian HM, Radak Z, Takeda M. Active recovery between interval bouts reduces blood lactate while improving subsequent exercise performance in trained men. Sports. 2017;5(2):40.

23. Kargar-Shoragi M-K, Ghofrani M, Bagheri L, Emamdoost S, Otadi K. The effect of cupping and one exercise session on levels of creatine kinase and lactate dehydrogenase among the members of a handball team. Trad Integr Med. 2016:115-21.

24. Barros NDA, Aidar FJ, DE MATOS DG, DE SOUZA RF, Neves EB, Cabral BGDAT, et al. Evaluation of muscle damage, body temperature, peak torque, and fatigue index in three different methods of strength gain. Int J Exerc Sci. 2020;13(3):1352.

25. Wan JJ, Qin Z, Wang PY, Sun Y, Liu X. Muscle fatigue: general understanding and treatment. Exp Mol Med. 2017;49(10):e384.

26. Dergaa I, Varma A, Musa S, Chaabane M, Salem AB, Fessi MS. Diurnal variation: Does it affect short-term maximal performance and biological parameters in police officers. Int J Sport Stud Hlth. 2020;3:e111424.

27. Dergaa I, Fessi MS, Chaabane M, Souissi N, Hammouda O. The effects of lunar cycle on the diurnal variations of short-term maximal performance, mood state, and perceived exertion. Chronobiol Int. 2019;36:1249-57.

28. Ebbeling CB, Clarkson PM. Exercise-induced muscle damage and adaptation. Sports Med. 1989;7(4):207-34.

29. Shi M, Wang X, Yamanaka T, Ogita F, Nakatani K, Takeuchi T. Effects of anaerobic exercise and aerobic exercise on biomarkers of oxidative stress. Environ Health Prev Med. 2007;12(5):202-8.

30. Brancaccio P, Maffulli N, Limongelli FM. Creatine kinase monitoring in sport medicine. Br Med Bull. 2007;81-82(1):209-30.

31. Spriet LL, Howlett RA, Heigenhauser GJ. An enzymatic approach to lactate production in human skeletal muscle during exercise. Med Sci Sports Exerc. 2000;32(4):756-63.

32. Soleimani N, Faridnouri H, Dayer M. The Effect of Dusts on Liver Enzymes and Kidney Parameters of Serum in Male Rats in Khuzestan, Iran. J Chem Health Risks. 2020;10(4):315-26.

33. Behboudi L, Eizadi M, Masrour H. The Effect of whey protein supplementation after eccentric resistance exercise on glutathione peroxidase and lactate dehydrogenase in non-trained young men. Int J Health Studies. 2019;5(2).

34. Taheri M, Irandost K, Mirmoezzi M, Ramshini M. Effect of aerobic exercise and omega-3 supplementation on psychological aspects and sleep quality in prediabetes elderly women. Sleep Hyp. 2019;21(2):170-4.

35. Souissi A, Yousfi N, Dabboubi R, Aloui G, Haddad M, Souissi N. Effect of acute melatonin administration on physiological response to prolonged exercise. Biol Rhythm Res. 2020;51:980-7.

36. Souissi A, Dergaa I, Chtourou H, Ben Saad H. The effect of daytime ingestion of melatonin on thyroid hormones responses to acute submaximal exercise in healthy active males: A pilot study. Am J Mens Health. 2022;16:15579883211070383.

37. Koo GH, Woo J, Kang S, Shin KO. Effects of supplementation with BCAA and L-glutamine on blood fatigue factors and cytokines in juvenile athletes submitted to maximal intensity rowing performance. J Phys Ther Sci. 2014;26(8):1241-6.

38. Petróczi A, Naughton DP, Pearce G, Bailey R, Bloodworth A, McNamee M. Nutritional supplement use by elite young UK athletes: fallacies of advice regarding efficacy. Int J Sport Nutr. 2008;5(1):1-8.

39. Brosnan JT, Brosnan ME. Creatine: endogenous metabolite, dietary, and therapeutic supplement. Annu Rev Nutr. 2007;27:241-61.

40. E. Creatine supplementation and dynamic high-intensity intermittent exercise. Scand J Med Sci Sports. 1993;3(3):143-9.

41. Hanan S, Eman S. Cupping therapy (al-hijama): It's impact on persistent non-specific lower back pain and client disability. Life Sci J. 2013;10(4s):631-42.

42. Fontana P, Boutellier U, Knöpfli-Lenzin C. Time to exhaustion at maximal lactate steady state is similar for cycling and running in moderately trained subjects. Eur J Appl Physiol. 2009;107(2):187-92.

43. Jennings G, Nelson L, Nestel P, Esler M, Korner P, Burton D, et al. The effects of changes in physical activity on major cardiovascular risk factors, hemodynamics, sympathetic

function, and glucose utilization in man: a controlled study of four levels of activity. Circulation. 1986;73(1):30-40.

44. Jafari M, Pouryamehr E, Fathi M. The Effect of eight weeks high intensity interval training (HIIT) on e-selection and p-selection in young obese females. Int J Sport Stud Hlth. 2017;1(1):e64336.
45. Irandoust K, Taheri M. Effect of peripheral heart action training and yoga exercise training on respiratory functions and C-reactive protein of postmenopausal women. WHB. 2019; 6(2): 1-7.
46. Hall M, Trojian TH. Creatine supplementation. Curr Sports Med Rep. 2013;12(4):240-4.
47. Hernández-Vicente A, Hernando D, Marín-Puyalto J, Vicente-Rodríguez G, Garatachea N, Pueyo E, Bailón R. Validity of the polar h7 heart-rate sensor for heart-rate variability analysis during exercise in different age, body composition and fitness level groups. Sensors. 2021;21:902.
48. Paryab N, Taheri M, Irandoust K, Mirmoezzi M. Effects of melatonin on neurological function and maintenance of physical and motor fitness in collegiate student-athletes following sleep deprivation. Int J Sport Stud Hlth. 2020;3(2).
49. Schröder H, Covas M, Marrugat J, Vila J, Pena A, Alcantara M, et al. Use of a three-day estimated food record, a 72-hour recall and a food-frequency questionnaire for dietary assessment in a Mediterranean Spanish population. Clin Nutr. 2001;20(5):429-37.
50. Souissi A, Yousfi N, Souissi N, Haddad M, Driss T. The effect of diurnal variation on the performance of exhaustive continuous and alternated-intensity cycling exercises. PLoS One. 2020;15:e0244191.
51. Yousef Abadi HA, Mirzaei B, Habibi H, Barbas I. Prevalence of Rapid Weight Loss and Its Effects on Elite Cadet Wrestlers Participated in the Final Stage of National Championships. Int J Sport Stud Hlth. 2017;1(1):e64316.
52. Ambrose PJ. Drug use in sports: a veritable arena for pharmacists. J Am Pharm Assoc. 2004;44(4):501-16.
53. Barcelos RP, Stefanello ST, Mauriz JL, Gonzalez-Gallego J, Soares FA. Creatine and the Liver: Metabolism and Possible Interactions. Mini Rev Med Chem. 2016;16(1):12-8.
54. Crichton GE, Bryan J, Murphy KJ. Dietary antioxidants, cognitive function and dementia--a systematic review. Plant Foods Hum Nutr. 2013;68(3):279-92.
55. Souissi A, Dergaa I, Musa S, Saad HB, Souissi N. Effects of daytime ingestion of melatonin on heart rate response during prolonged exercise. Mov Sport Sci/Sci Mot. 2022;115:25-32.
56. Romdhani M, Dergaa I, Moussa-Chamari I, Souissi N, Chaabouni Y, Mahdouani K, Abene O, Driss T, Chamari K, Hammouda O. The effect of post-lunch napping on mood, reaction time, and antioxidant defense during repeated sprint exercice. Biol Sport. 2021;38:629-38.
57. Romdhani M, Souissi N, Dergaa I, Moussa-Chamari I, Chaabouni Y, Mahdouani K, Hammouda O. The effect of caffeine, nap opportunity and their combination on biomarkers of muscle damage and antioxidant defence during repeated sprint exercise. Biol Sport. 2022;39:1033-42.
58. Souissi A, Dergaa I. An overview of the potential effects of melatonin supplementation on athletic performance. Int J Sport Stud Hlth. 2021;4:e121714.
59. Banerjee B, Sharma U, Balasubramanian K, Kalaivani M, Kalra V, Jagannathan NR. Effect of creatine monohydrate in improving cellular energetics and muscle strength in ambulatory Duchenne muscular dystrophy patients: a randomized, placebo-controlled 31P MRS study. Magn Reson Imaging. 2010;28(5):698-707.
60. Rosenbaum M, Leibel RL. The physiology of body weight regulation: relevance to the etiology of obesity in children. Pediatrics. 1998;101(3 Pt 2):525-39.
61. Poprzecki S, Zajac A, Czuba M, Waskiewicz Z. The effects of terminating creatine supplementation and resistance training on anaerobic power and chosen biochemical variables in male subjects. J Hum Kinet. 2008;20(1):99-110.
62. Atashak S, Jafari A. Effect of short-term creatine monohydrate supplementation on indirect markers of cellular damage in young soccer players. Sci Sports. 2012;27(2):88-93.
63. Lin WT, Yang SC, Tsai SC, Huang CC, Lee NY. L-Arginine attenuates xanthine oxidase and myeloperoxidase activities in hearts of rats during exhaustive exercise. Br J Nutr. 2006;95(1):67-75.
64. Moraska A. Sports massage. A comprehensive review. J Sports Med Phys Fitness. 2005;45(3):370-80.
65. Goodwin J. A comparison of massage and sub-maximal exercise as warm-up protocols combined with a stretch for vertical jump performance. J Sports Sci. 2002;20(1):48-9.
66. Sun D-l, Zhang Y, Chen D-l, Zhang A-b, Xu M, Li Z-j, et al. Effect of moxibustion therapy plus cupping on exercise-induced fatigue in athletes. J Acupunct Tuina Sci. 2012;10(5):281-6.
67. Lowe DT. Cupping therapy: An analysis of the effects of suction on skin and the possible influence on human health. Complement Ther Clin Pract. 2017;29:162-8.
