# Supplementary material for: Impact of creatine supplementation on inflammation: evidence from a systematic review and meta-analysis of randomized double-blind placebo trials
Source: Front Immunol. 2026 Feb 19;17:1743603. doi: 10.3389/fimmu.2026.1743603 (PMC12961398; doi:10.3389/fimmu.2026.1743603)
Supplement: Supplementary file 2 [file SupplementaryFile1.zip › SR Creatine inflammatory markers (Kell Doutorado). /Supplementary Files/Final References/Kell/Marini et al 2019.pdf]

ResearchGate

See discussions, stats, and author profiles for this publication at: https://www.researchgate.net/publication/335900308

Short-Term Creatine Supplementation May Alleviate the Malnutrition-Inflammation Score and Lean Body Mass Loss in Hemodialysis Patients: A Pilot Randomized Placebo-Controlled Trial

Article *in* Journal of Parenteral and Enteral Nutrition · September 2019

DOI: 10.1002/jpen.1707

CITATIONS
37

READS
225

8 authors, including:

Ana Clara Barreto Marini
Federal University of Goiás
24 PUBLICATIONS **203** CITATIONS

SEE PROFILE

João Felipe Mota
Federal University of Goiás
133 PUBLICATIONS **4,103** CITATIONS

SEE PROFILE

Benjamin T Wall
University of Exeter
115 PUBLICATIONS **5,103** CITATIONS

SEE PROFILE

Claude Pichard
Hôpitaux Universitaires de Genève
179 PUBLICATIONS **1,599** CITATIONS

SEE PROFILE

All content following this page was uploaded by Gustavo Duarte Pimentel on 03 March 2020.

The user has requested enhancement of the downloaded file.

Journal of Parenteral and Enteral Nutrition
Volume 00 Number 0
xxx 2019 1–8
© 2019 American Society for Parenteral and Enteral Nutrition
DOI: 10.1002/jpen.1707
wileyonlinelibrary.com
WILEY

Short-Term Creatine Supplementation May Alleviate the Malnutrition-Inflammation Score and Lean Body Mass Loss in Hemodialysis Patients: A Pilot Randomized Placebo-Controlled Trial

Ana Clara B. Marini, MSc[1]; Reika D. Motobu, BSc[1]; Ana T. V. Freitas, PhD[1]; João F. Mota, PhD[1]; Benjamin T. Wall, PhD[2]; Claude Pichard, MD, PhD[3]; Alessandro Laviano, MD, PhD[4]; and Gustavo Duarte Pimentel, PhD[1]

Abstract

Background: Creatine supplementation has been proposed to alleviate muscle loss in various populations, but has not been investigated in hemodialysis (HD) patients. Thus, our objective was to evaluate whether creatine supplementation could attenuate the loss of lean body mass (LBM) and malnutrition-inflammation score (MIS) in HD patients. *Methods:* A randomized, placebo-controlled, double blind, parallel-design study included HD patients, of both sexes, aged 18–59 years. The patients were allocated to a Placebo Group (PG; n = 15; received maltodextrin, 1st week: 40 g/day and 2nd–4th weeks: 10 g/day) and a Creatine Group (CG; n = 15; received creatine plus maltodextrin, 1st week: 20 g/day of creatine plus 20 g/day of maltodextrin and 2nd–4th weeks: 5 g/day of creatine plus 5 g/day of maltodextrin). Pre and post the intervention, patients were evaluated for food intake, MIS, body composition and biochemical parameters. *Results:* CG group attenuated the MIS (Pre: 5.57 ± 0.72 *vs.* Post: 3.85 ± 0.47 score, $P = 0.003$) compared with PG (Pre: 5.71 ± 0.97 *vs.* Post: 5.36 ± 0.95 score, $P = 0.317$) (supplement × time $P = 0.017$, effect size: 0.964). The change of LBM was greater in CG than in PG (CG: Δ0.95 vs PG: Δ0.13 kg). At post-intervention, 28.6% of PG patients presented LBM loss and 71.4% remain stable. In contrast, 14.4% of CG patients had LBM loss, 42.8% remain stable and 42.8% gained. Food intake and quality of life did not change. CG increased the BMI and gait speed in post-compared to pre-moment, but no difference among the groups. *Conclusion:* In HD patients, four weeks of creatine supplementation may alleviate the MIS as well as attenuate the LBM loss compared to placebo. (*JPEN J Parenter Enteral Nutr.* 2019;00:1–8)

Keywords

creatine; hemodialysis; inflammation; lean body mass

Clinical Relevancy Statement

Hemodialysis induces an imbalance between muscle protein synthesis and breakdown, leading to loss of muscle mass and function in patients. This study found that short-term creatine supplementation attenuates the malnutrition-inflammation score and lean body mass when compared with placebo.

Background

The malnutrition-inflammatory score (MIS) is commonly associated with morbidity and mortality in chronic kidney disease (CKD) patients undergoing hemodialysis (HD)[1] and nondialyzed patients.[2] Additionally, a Brazilian study revealed that the MIS is a useful tool to evaluate protein-energy wasting (PEW) in CKD patients.[2] Considering that PEW is a condition of reduced body protein and energy stores[3] and that reduced lean body mass (LBM) is negatively associated with MIS,[4] a new therapeutic strategy to attenuate LBM loss and MIS values may improve the clinical outcome and the quality of life (QoL) of HD patients.

From the [1]Clinical and Sports Nutrition Research Laboratory (Labince), Faculty of Nutrition, Federal University of Goias, Goiânia, GO, Brazil; [2]Department of Sport and Health Sciences, College of Life and Environmental Sciences, University of Exeter, Exeter, UK; [3]Clinical Nutrition, Geneva University Hospital, Geneva, Switzerland; and the [4]Department of Clinical Medicine, Sapienza University, Viale dell'Università 37, 00185 Rome, Italy.

Financial disclosure: A. C. B. M. was supported by Capes, Brazil.

Conflicts of interest: None declared.

Received for publication March 26, 2019; accepted for publication August 21, 2019.

This article originally appeared online on xxxx 0, 2019.

Corresponding Author:
Gustavo Duarte Pimentel, Faculdade de Nutrição, Universidade Federal de Goiás, Rua 227, Quadra 68 s/n°, Setor Leste Universitário, CEP: 74605080, Goiânia, GO, Brazil.
Email: gupimentel@yahoo.com.br

Creatine supplementation has been proposed to alleviate muscle loss in various populations.[5] Kley et al[6] in a meta-analysis found that creatine supplementation in patients with muscular myopathies was well tolerated and may lead to an increase in muscle strength and LBM. However, the impact of creatine supplementation on LBM, MIS, and QoL has not been investigated in HD patients.

Creatine supplementation may enhance muscular phosphor creatine stores and stimulate rapid recovery of adenosine triphosphate levels.[7,8] In addition, water retention due to Cr-induced reduction in ionic strength may contribute to the gain of body weight, LBM, and muscle strength.[9] Considering that creatine supplementation is safe and inexpensive and appears to positively modulate body composition in patients with wasting diseases or in those with chronic dialysis,[8,10,11] we hypothesized that 4 weeks of creatine monohydrate supplementation would lower the MIS and the LBM loss in HD patients. Thus, our objective was to evaluate whether creatine supplementation could attenuate the loss of LBM and MIS in CKD patients undergoing HD.

Materials and Methods

Design of Study

This randomized, placebo-controlled, and double-blind clinical trial was conducted with patients of both sexes who were diagnosed with CKD, undergoing HD, and aged between 18 and 59 years. The overall study lasted 6 weeks, and the intervention with creatine was 4 weeks.

After inclusion of the patients in the study, they were randomly allocated by gender, age, and LBM content. The patients signed the informed consent form approved by the Research Ethics Committee of the Federal University of Goiás, number 1.470.351, and this study is part of a larger trial looking at various interventions that was previously registered in the Brazilian Registry of Clinical Trials under the code RBR-98wzgn.

Recruitment and Sample Selection

The sample and criteria of inclusion were composed of patients who were diagnosed with CKD and undergoing HD treatment for >3 months at the 3 HD outpatient clinics in Goiânia, GO, Brazil. The Gpower 3.1 software was used to calculate the sample size,[12] in which a significance level of 5% with statistical power of 80%, effect size 0.50, 2 groups, and 2 measurements (LBM and MIS) were considered, so the study population should be 12 patients per group.

Exclusion criteria included patients presenting with neurological disease, severe cardiovascular diseases, or physical disability (amputations, deep vein thrombosis) and patients who underwent structured physical training 3 months prior to the date of inclusion in the study or those already taking supplements such as creatine.

Experimental Groups

The study was performed with 30 patients divided into 2 groups randomized by gender, age, and LBM content (Figure 1): (1) placebo group (PG), composed of 15 patients which received maltodextrin; and (2) creatine group (CG), composed of 15 patients who received creatine monohydrate. During the intervention period, 1 patient in each group was excluded because of nonadherence to the creatine supplementation (90% of the recommended dose was accepted as the limit of adhesion).

The intervention was separated into 3 steps after the randomization and division of the groups (Supplementary Figure S1): (1) During week 1 of the study, the initial evaluations were performed, including food intake assessment, MIS (see below for details), blood tests, anthropometric, and body composition (dual-energy x-ray absorptiometry [DXA]). (2) From week 2 to 5, the intervention with the creatine and the placebo was performed (see below). And (3) during week 6 of the study, the same parameters were reassessed within 48 hours after the last intake of the creatine and the placebo.

Protocol Supplementation

The blinded intervention was performed as described in Table 1. The sachets containing either creatine or placebo were standardized to avoid any identification of the content by the patients. Creatine-loading phase induces a rapid increase of intramuscular creatine phosphate, which allows for a short intervention period.[13,14] Because creatine powder had no taste, whereas maltodextrin had lemon flavor, all doses of creatine contained maltodextrin. Fortunately, the addition of maltodextrin to creatine favors absorption by the gastrointestinal tract and the uptake by muscle tissue.[15] Both creatine and maltodextrin were donated by Maxtitanium, Supley Laboratório de Alimentos e Suplementos Nutricionais, Matão, SP, Brazil.

Evaluation of Food Intake

Food intake assessments (24-hour food recall) were conducted by trained nutritionists at the beginning of the intervention (week 1), during the intervention (week 3), and at the end of the intervention (last week). The data were calculated in the Dietpro software (5.8 version; Agromídia Software, Viçosa, MG, Brazil), and the macronutrients and micronutrients consumption of the patients were quantified.

Malnutrition-Inflammation Score

Malnutrition-inflammation score (MIS) is a tool based on Subjective Global Assessment (SGA), which includes 3 other items: body mass index (BMI), serum albumin

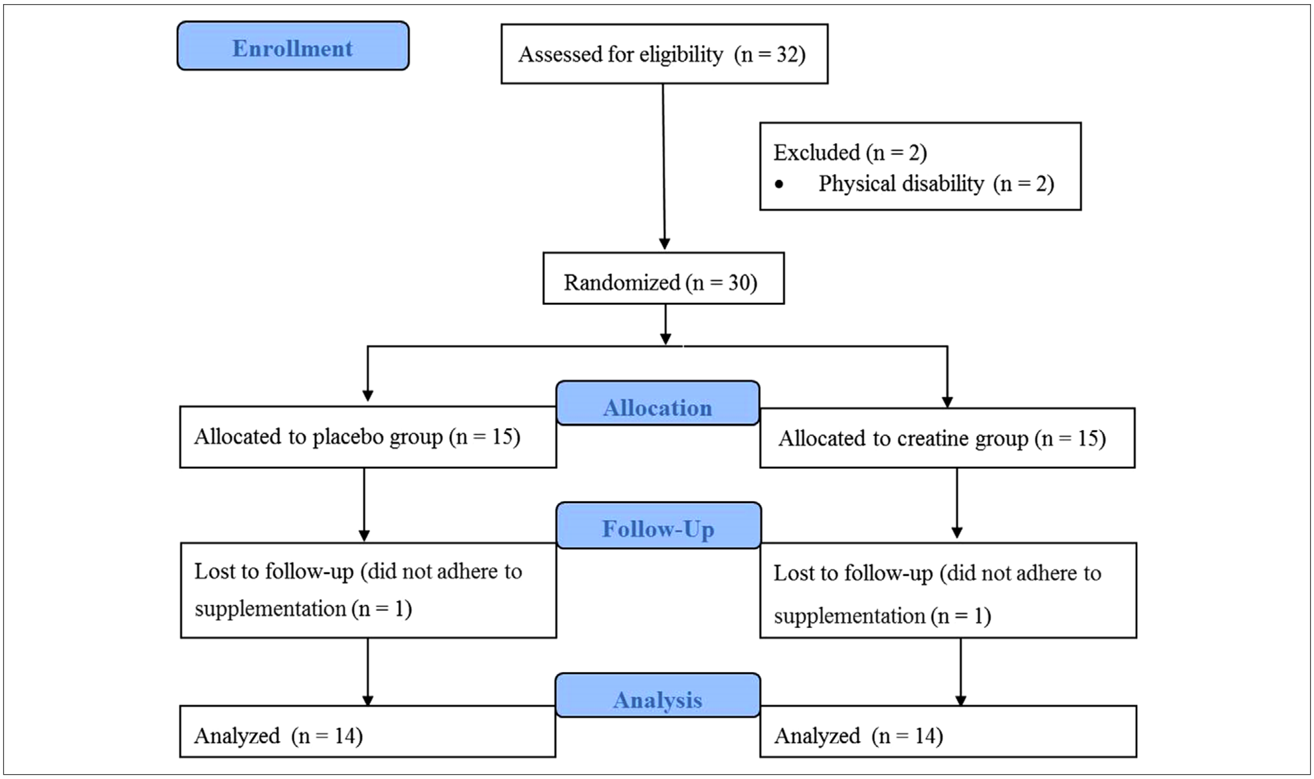

Figure 1. Participant flowchart (CONSORT). First stage: Evaluations (week 1); second stage: Intervention (weeks 2–5); and third stage: Reevaluations (week 6). CONSORT, Consolidated Standards of Reporting Trials; LBM, lean body mass; MIS, malnutrition-inflammation score.

Table 1. Blinded Intervention Protocol.

	Week 1 (Loading Phase)		Weeks 2–4	
Groups	4 Times per Day[a]	Total per Day	1 Time per Day[b]	Total per Day
Placebo (malto)	10 g	40 g	10 g	10 g
Creatine (malto + creatine)	5 g (creatine) + 5 g (malto)	20 g + 20 g	5 g (creatine) + 5 g (malto)	5 g + 5 g

[a]Breakfast, lunch, snack, and dinner.
[b]Lunch or dinner; maltodextrin (malto) with lemon flavor.

concentrations, and total iron-binding capacity (TIBC).[1,16,17] MIS presents clinical history and physical and biochemical analysis of the patient. The clinical history consists of addressing aspects such as weight reduction in the last 6 months, changes in dietary intake, presence of gastrointestinal symptoms, and functional capacity related to nutrition status. Physical examination includes aspects such as subcutaneous fat loss, muscle loss, the presence of edema resulting from malnutrition, and ascites, which have been defined as normal, mild, moderate, or severe. Analysis of biochemical parameters includes serum albumin level and TIBC exams. After completion of the clinical, physical, and biochemical examinations (the results of which could range from 1 to 30), then the classification of the nutrition status was performed. A score of ≤6 presents normality, and a score of >6 presents classification for malnutrition and high MIS.[1,16,17]

Anthropometric and Body Composition Assessment

Anthropometric data were collected in the intermediate session of the week of HD (second session). Weight and height were evaluated by an anthropometric digital scale (Filizola) for later calculation of BMI. In addition, arm, calf, and thigh circumferences were measured using a flexible tape measure. The data were collected in duplicate by trained nutritionists.

Body composition was assessed by DXA (Lunar DPX NT, GE Medical Systems Lunar, Wisconsin, Madison,

Table 2. Baseline Characteristics.

Variables	Placebo (n = 14) Mean ± SEM	Creatine (n = 14) Mean ± SEM	*P*-Value
Sex, n[a]			
Female	5	4	.68
Male	9	10	
Age, y	41.79 ± 2.72	41.86 ± 3.32	.98
Body mass index, kg/m²	21.93 ± 1.28	22.76 ± 1.41	.60
Comorbidities, n[a]			
Hypertension	10	6	.15
Diabetes	1	1	
Hypertension + diabetes	0	1	
Glomerulonephritis	2	0	
Others	1	3	
Unknown	0	3	

[a] χ^2.

USA). This equipment collected the total fat mass and LBM.

Quality of Life

QoL was assessed by Short Form Health Survey 36 (SF-36), which is a well-documented health-related instrument consisting of 36 questions and divided into 8 dimensions: physical functioning, physical role functioning, pain, general health, vitality, social role functioning, emotional role functioning, and mental health. The data of this form vary from 0 to 100 (worse to best status) and have been validated in a Brazilian population.[18]

Biochemical Analysis

Data from the monthly biochemical analysis performed by the clinics (pre-hemodialysis urea and post-hemodialysis urea, phosphorus, serum albumin level, and TIBC) were collected in the patients' medical records before and after the intervention. For the exams not performed periodically, the serum was collected by nursing and stored at −80°C for subsequent quantification of serum creatinine by the chemiluminescence method in the Roche Architect 8000 equipment.

Statistical Analyses

The data were deposited in Microsoft Excel and transcribed into the programs SPSS version 18.0 and e R Studio version 3.4.3. Descriptive statistics (absolute and relative frequencies and SE of the mean) were used. The continuous variables were tested for normality by the Shapiro-Wilk Test. χ^2 test was used to evaluate categorical variables. Differences in food intake and Δ of variables among the PG and CG were tested by Wilcoxon test or Mann-Whitney *U* test and Student *t*-test, respectively. To evaluate the interaction between supplements and intervention time, two-way analysis of variance (ANOVA) test, followed by post hoc Tukey test, was used. The level of statistical significance was set at 5% ($P < .05$).

Results

Baseline Characteristics and Food Intake

The baseline characteristics of the patients are shown in Table 1. Both groups were similar for sex, age, BMI, and previous comorbidities (Table 2), as well as food intake (Table 3).

Malnutrition-Inflammation Score

The MIS showed a significant reduction in CG (Δ: −1.71) compared with PG (Δ: −0.36) ($P = .01$, with high effect size) (Table 4).

Anthropometry and Body Composition

Although no difference among the groups was observed ($P = .43$), both enhanced the body weight (PG Δ: 0.51 kg vs CG Δ: 0.77 kg) and the BMI postintervention compared with preintervention. In addition, no change in arm, thigh, and calf circumferences was found between the groups ($P > .05$) (Table 4). By contrast, the gait speed was higher in the CG (Δ: 0.05 m/s) than in the PG (Δ: −0.03 m/s), with high effect size, but no difference among the groups.

LBM was higher in CG (Δ: 0.95 kg) than in PG (Δ: 0.13 kg) (ANOVA supplement × time $P = .03$ and high effect size), and there was higher fat body mass in PG (Δ: 0.39 kg) than in CG (Δ: −0.17) (ANOVA supplement × time $P = .02$ and high effect size) (Table 4). Additionally, at the end of the intervention, 28.6% and 71.4% of patients of

Table 3. Food Intake Among the Groups.

Variables	Placebo (n = 14) Mean ± SEM	Creatine (n = 14) Mean ± SEM	*P*-Value
Energy, kcal	1629.57 ± 265.52	1553.46 ± 157.42	.80
Carbohydrate, g	180.35 ± 26.93	177.91 ± 21.54	.94
Total fat, g	66.90 ± 12.21	64.67 ± 6.64	.57
Monounsaturated fat, g	18.64 ± 2.35	18.30 ± 2.30	.80
Polyunsaturated fat, g	15.81 ± 2.21	15.21 ± 1.81	1
Saturated fat, g	16.16 ± 2.25	16.17 ± 2.26	.98
Cholesterol, mg	255.75 ± 74.67	272.12 ± 36.83	.21
Protein, g	76.47 ± 14.27	65.96 ± 8.17	.63
Protein, g/kg b.w.	1.31 ± 0.23	1.14 ± 0.16	.54
Calcium, mg	352.75 ± 90.62	367.63 ± 65.16	.37
Iron, mg	8.26 ± 1.15	6.85 ± 0.70	.35
Phosphorus, mg	948.06 ± 184.36	825.70 ± 80.32	1
Magnesium, mg	177.82 ± 22.17	168.30 ± 20.57	.70
Potassium, mg	1880.31 ± 254.44	1847.39 ± 257.60	.98
Sodium, mg	3860.10 ± 644.30	3551.40 ± 381.10	.66
Dietary fiber, g	16.75 ± 1.96	14.52 ± 1.78	.35

Mann-Whitney *U* test.
b.w., body weight.

PG presented an LBM loss and remained stable, respectively (Figure 2A). In contrast, in the CG, 14.4% of patients presented LBM loss, 42.8% remained stable, and 42.8% gained (Figure 2B). Moreover, at the end of the study, CG presented a reduction of Δ mean fat body mass ($P = .011$, Figure 3A) and an increase of Δ mean LBM ($P = .011$, Figure 3B).

Biochemical Analysis

Although the serum creatinine concentrations were increased in CG (Δ: 1.90 mg/dL) compared with PG (Δ: −0.82 mg/dL) (ANOVA supplement × time $P = .001$ and high effect size), serum urea pre-HD and post-HD concentrations and phosphorus did not alter with the treatment ($P > .05$) (Table 4).

Quality of Life

QoL did not change in any of the 8 domains assessed (Supplementary Table S1).

Discussion

The present study is the first to investigate the effects of 4 weeks of creatine supplementation in patients undergoing HD. We showed that supplementation was able to alleviate the MIS and LBM loss. In addition, 43% of the CG patients increased LBM, whereas no gain was seen in patients administered the placebo. These results corroborate with the meta-analysis of Candow et al,[19] who suggest that creatine supplementation may lead to physiological benefits and improved body composition across various populations.

Regarding MIS, there was a significant reduction in CG after the intervention. Of note, 3 patients previously classified as malnourished improved to normal values. MIS is an important predictor of mortality among CKD patients on HD.[17] Likewise, a Brazilian observational retrospective cohort study conducted with 171 patients revealed that the instrument has 53% sensitivity and 82% specificity for mortality in patients with >24 months of HD treatment. Thus, we can observe the importance of reducing the number of previously malnourished patients, and we can infer that the reduction in the score in the group supplemented with creatine likely decreases the chances of death.[20]

In the CG, there was a significant increase in body weight, BMI, gait speed, and LBM. These findings corroborate with a 2009 study by Johnston et al, who observed that when immobilizing the arm of healthy young people and supplementing them with creatine, there was a preservation of lean arm mass (+0.9%), observed by DXA, whereas in the PG there was a reduction (−3.7%).[21] Likewise, previous studies showed that creatine supplementation leads to enhanced LBM as well as body weight in young and older adults.[22,23] Similar to our study, Gotshalk et al[24] showed in older adults and elderly patients that 7 days of creatine supplementation was able to increase body mass and LBM (likely in part because of water retention) as well as improve the time in gait test. Thus, these data reinforce the initial hypothesis that short-term creatine supplementation can raise the LBM and improve muscle function in older people[23] and also in adults with chronic disease, as observed in the present study.

Indeed, treatment with maltodextrin and creatine favors greater input of creatine into the muscle[15] and higher availability of nutrients to improve nutrition status.

Table 4. Comparison of MIS, Body Composition, and Biochemical Parameters Among the Groups.

Variables	Placebo (n = 14) Mean ± SEM			Creatine (n = 14) Mean ± SEM			ΔP	Effect size	ANOVA P
	Preintervention	Postintervention	Δ	Preintervention	Postintervention	Δ			
MIS	5.71 ± 0.97[a]	5.36 ± 0.95[a]	−0.36 ± 0.39	5.57 ± 0.72[a]	3.85 ± 0.47[b#]	−1.71 ± 0.37	0.01*	0.964	.01*
Body composition									
Body weight, kg	58.91 ± 3.67	59.42 ± 3.69[#]	0.51 ± 0.21	62.07 ± 4.83	62.84 ± 4.81[#]	0.77 ± 0.24	0.43	0.301	.43
Body mass index, kg/m²	21.93 ± 1.28	22.13 ± 1.30	0.19 ± 0.09	22.76 ± 1.41	23.04 ± 1.39[#]	0.27 ± 0.08	0.52	0.245	.33
Arm circumference, cm	27.68 ± 1.16	27.81 ± 1.13	0.12 ± 0.23	28.59 ± 1.60	28.38 ± 1.35	−0.20 ± 0.71	0.65	0.160	.66
Thigh circumference, cm	45.46 ± 1.86	45.30 ± 1.76	−0.15 ± 0.58	45.61 ± 1.94	45.96 ± 1.93	0.35 ± 0.14	0.41	0.312	.41
Calf circumference, cm	33.51 ± 1.17	32.55 ± 0.82	−0.96 ± 0.74	33.77 ± 1.42	34.04 ± 1.40	0.27 ± 0.16	0.11	0.608	.11
Gait speed, m/s	0.81 ± 0.03	0.78 ± 0.03	−0.03 ± 0.04	0.72 ± 0.03	0.78 ± 0.03[#]	0.05 ± 0.02	0.09	0.647	.22
Lean body mass, kg	41.33 ± 2.28[a]	41.46 ± 2.36[a]	0.13 ± 0.21	42.96 ± 2.74[b]	43.92 ± 2.71[a#]	0.95 ± 0.30	0.03*	0.832	.03*
Fat body mass, kg	15.23 ± 2.51[a]	15.63 ± 2.52[b#]	0.39 ± 0.12	16.77 ± 2.93[b]	16.60 ± 2.95[b]	−0.17 ± 0.01	0.02*	0.903	.02*
Biochemical parameters									
Creatinine, mg/dL	5.86 ± 0.60[a]	5.03 ± 0.45[a]	−0.82 ± 1.94	4.04 ± 0.49[b]	5.95 ± 0.84[a#]	1.90 ± 0.76	0.00*	1.113	.00*
Pre-hemodialysis urea, mg/dL	136.92 ± 8.05	150.42 ± 11.89	13.50 ± 8.41	133.42 ± 10.76	131.92 ± 8.22	−1.50 ± 7.77	0.20	0.495	.20
Post-hemodialysis urea, mg/dL	36.79 ± 6.55	30.07 ± 6.76	−6.71 ± 4.21	40.71 ± 7.38	44.86 ± 5.64	4.14 ± 8.16	0.24	0.446	.24
Phosphorus, mg/dL	5.32 ± 0.65	5.50 ± 0.89	0.17 ± 0.40	5.71 ± 0.44	5.72 ± 0.46	0.01 ± 0.32	0.76	0.115	.76

ANOVA, analysis of variance; MIS, malnutrition-inflammatory score.
* $P < .05$ was considered as significant.
# Difference vs preintervention. a ≠ b: difference in two-way ANOVA followed by post hoc Tukey test.

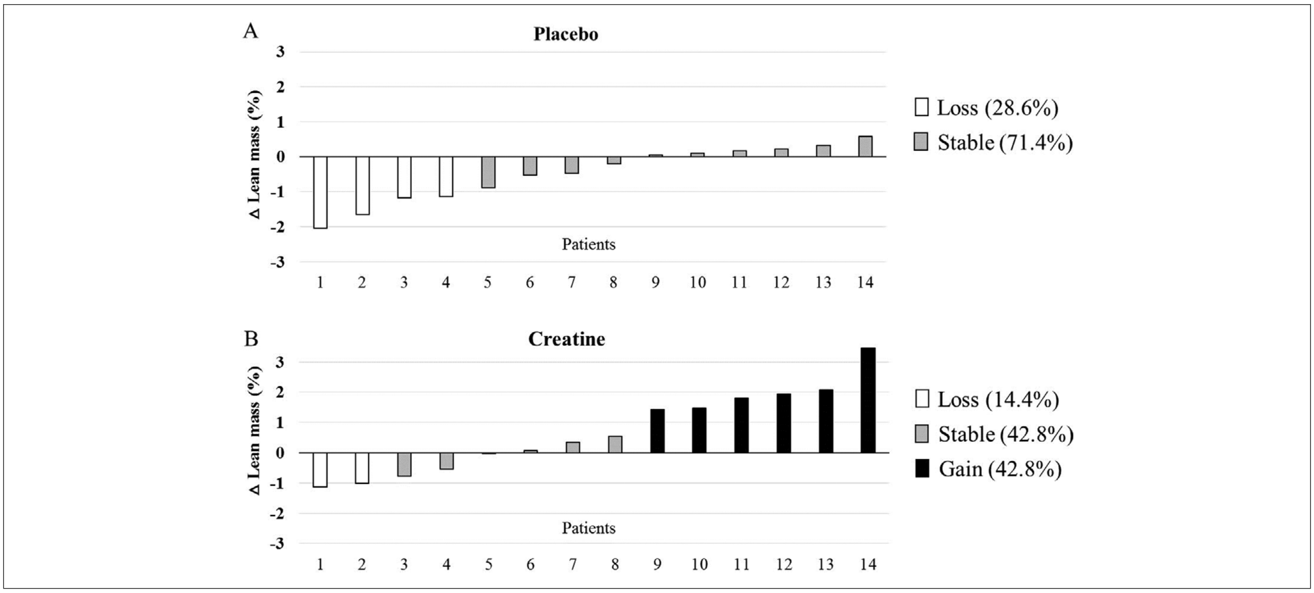

Figure 2. Δ of individual change in lean body mass (%).

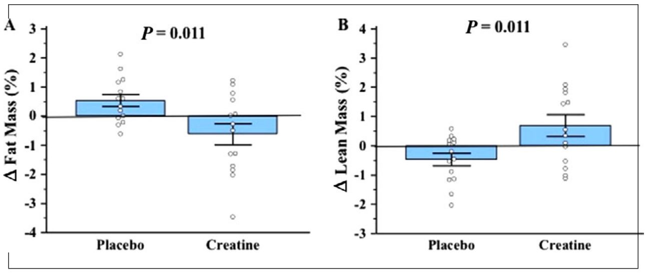

Figure 3. Δ of % change in body fat mass (A) and in lean body mass (B), measured by dual x-ray absorptiometry in the treated and control groups.

The addition of maltodextrin to creatine favors absorption by the gastrointestinal tract and uptake by muscle tissue.[15]

Regarding biochemical analyses, there was a significant increase in serum creatinine concentrations in CG when compared with PG. The elevated serum creatinine levels are related to the fact that approximately 2% of daily creatine is converted into the cyclic degradation product and can leave the cells through the permeable cell membrane and enter the blood stream without provoking toxic effects on the body.[11] Additionally, low serum creatinine concentrations (<10 [6–10] mg/dL), which is a good marker of nutrition status in HD patients is associated with increased mortality and reduced muscle mass[25,26]; thus, we should study how poor dietary consumption impairs the loss of LBM.

According to Wallimann et al,[11] intradialytic creatine supplementation is safe and may improve the QoL of HD patients; however, in the present study, we did not observe alteration in any domains of the SF-36 questionnaire. We believe the present study may encourage further research with creatine supplementation in CKD patients on HD, as we observed that creatine generated clinically relevant results, with good compliance by the patients, with no complaints of ingestion difficulties or side effects.

The present study presented positive points: (1) the use of DXA to evaluate the body composition, once it allows greater veracity in the results; (2) food intake and protein intake assessment, since we can affirm that attenuation of LBM and MIS loss were independent of food consumption, once no changes from the beginning to the end of the study were found.

The main study limitations are that (1) the hydration status was not quantified. This may have altered the measured LBM because of an accumulation of intramuscular water. DXA was used for LBM determination, and this method does not separation of intracellular from extracellular water contained in the LBM. Bioelectrical impedance analysis could estimate the amount of intramuscular water,[27] but we have been unable to use bioelectrical impedance that determines the hydration status. And (2) no physical activity test was applied, and the impact of physical activity on LBM remains unknown.

Conclusion

In HD patients, 4 weeks of creatine supplementation may alleviate the MIS as well as attenuate LBM loss, compared with placebo. However, more comprehensive follow-up studies are needed to recommend the routine use of creatine supplementation.

Statement of Authorship

A. C. B. Marini, B. T. Wall, and G. D. Pimentel contributed to the design of the research; A. C. B. Marini and R. D. Motobu contributed to the acquisition and analysis of the data; A. C. B. Marini, A. T. V. Freitas, J. F. Mota, B. T. Wall, C. Pichard, A. Laviano, and G. D. Pimentel contributed to the interpretation of the data; and A. C. B. Marini and G. D. Pimentel drafted the manuscript. All authors critically revised the manuscript, agree to be fully accountable for ensuring the integrity and accuracy of the work, and read and approved the final manuscript.

Supplementary Information

Additional supporting information may be found online in the Supporting Information section at the end of the article.

References

1. Kalantar-Zadeh K, Kopple JD, Block G, Humphreys MH. A malnutrition-inflammation score is correlated with morbidity and mortality in maintenance hemodialysis patients. *Am J Kidney Dis*. 2001;38(6):1251-1263.
2. Amparo FC, Kamimura MA, Molnar MZ, et al. Diagnostic validation and prognostic significance of the malnutrition-inflammation score in nondialyzed chronic kidney disease patients. *Nephrol Dial Transpl*. 2015;30(5):821-828.
3. Fouque D, Kalantar-Zadeh K, Kopple J, et al. A proposed nomenclature and diagnostic criteria for protein-energy wasting in acute and chronic kidney disease. *Kidney Int*. 2008;73(4):391-398.
4. Rambod M, Bross R, Zitterkoph J, et al. Association of malnutrition-inflammation score with quality of life and mortality in hemodialysis patients: a 5-year prospective cohort study. *Am J Kidney Dis*. 2009;53(2):298-309.
5. Moon A, Heywood L, Rutherford S, Cobbold C. Creatine supplementation: can it improve quality of life in the elderly without associated resistance training? *Curr Aging Sci*. 2013;6(3):251-257.
6. Kley RA, Tarnopolsky MA, Vorgerd M. Creatine treatment in muscle disorders: a meta-analysis of randomised controlled trials. *J Neurol Neurosurg Psychiatry*. 2008;79(4):366-367.
7. Greenhaff PL, Bodin K, Soderlund K, Hultman E. Effect of oral creatine supplementation on skeletal muscle phosphocreatine resynthesis. *Am J Physiol*. 1994;266(5 pt 1):E725-E730.
8. Gualano B, Roschel H, Lancha AH, Jr., Brightbill CE, Rawson ES. In sickness and in health: the widespread application of creatine supplementation. *Amino Acids*. 2012;43(2):519-529.
9. Murphy RM, Stephenson DG, Lamb GD. Effect of creatine on contractile force and sensitivity in mechanically skinned single fibers from rat skeletal muscle. *Am J Physiol Cell Physiol*. 2004;287(6):C1589-C1595.
10. Sakkas GK, Schambelan M, Mulligan K. Can the use of creatine supplementation attenuate muscle loss in cachexia and wasting? *Curr Opin Clin Nutr Metab Care*. 2009;12(6):623-627.
11. Wallimann T, Riek U, Moddel M. Intradialytic creatine supplementation: a scientific rationale for improving the health and quality of life of dialysis patients. *Med Hypotheses*. 2017;99:1-14.
12. Faul F, Erdfelder E, Lang AG, Buchner A. G*Power 3: a flexible statistical power analysis program for the social, behavioral, and biomedical sciences. *Behav Res Methods*. 2007;39(2):175-191.
13. McKenna MJ, Morton J, Selig SE, Snow RJ. Creatine supplementation increases muscle total creatine but not maximal intermittent exercise performance. *J Appl Physiol*. 1999;87(6):2244-2252.
14. Gualano B, Ferreira DC, Sapienza MT, Seguro AC, Lancha AH, Jr. Effect of short-term high-dose creatine supplementation on measured GFR in a young man with a single kidney. *Am J Kidney Dis*. 2010;55(3):e7-e9.
15. Green AL, Simpson EJ, Littlewood JJ, Macdonald IA, Greenhaff PL. Carbohydrate ingestion augments creatine retention during creatine feeding in humans. *Acta Physiol Scand*. 1996;158(2):195-202.
16. Enia G, Sicuso C, Alati G, Zoccali C. Subjective global assessment of nutrition in dialysis patients. *Nephrol Dial transpl*. 1993;8(10):1094-1098.
17. Steiber AL, Kalantar-Zadeh K, Secker D, McCarthy M, Sehgal A, McCann L. Subjective global assessment in chronic kidney disease: a review. *J Ren Nutr*. 2004;14(4):191-200.
18. Neto JF, Ferraz MB, Cendoroglo M, Draibe S, Yu L, Sesso R. Quality of life at the initiation of maintenance dialysis treatment–a comparison between the SF-36 and the KDQ questionnaires. *Qual Life Res*. 2000;9(1):101-107.
19. Candow DG, Chilibeck PD, Forbes SC. Creatine supplementation and aging musculoskeletal health. *Endocrine*. 2014;45(3):354-361.
20. Borges MC, Vogt BP, Martin LC, Caramori JC. Malnutrition inflammation score cut-off predicting mortality in maintenance hemodialysis patients. *Clin Nutr ESPEN*. 2017;17:63-67.
21. Johnston AP, Burke DG, MacNeil LG, Candow DG. Effect of creatine supplementation during cast-induced immobilization on the preservation of muscle mass, strength, and endurance. *J Strength Cond Res*. 2009;23(1):116-120.
22. Branch JD. Effect of creatine supplementation on body composition and performance: a meta-analysis. *Int J Sport Nutr Exerc Metab*. 2003;13(2):198-226.
23. Gualano B, Rawson ES, Candow DG, Chilibeck PD. Creatine supplementation in the aging population: effects on skeletal muscle, bone and brain. *Amino Acids*. 2016;48(8):1793-805.
24. Gotshalk LA, Kraemer WJ, Mendonca MA, et al. Creatine supplementation improves muscular performance in older women. *Eur J Appl Physiol*. 2008;102(2):223-231.
25. Pifer TB, McCullough KP, Port FK, et al. Mortality risk in hemodialysis patients and changes in nutritional indicators: DOPPS. *Kidney Int*. 2002;62(6):2238-2245.
26. Kalantar-Zadeh K, Streja E, Kovesdy CP, et al. The obesity paradox and mortality associated with surrogates of body size and muscle mass in patients receiving hemodialysis. *Mayo Clin Proc*. 2010;85(11):991-1001.
27. Cruz-Jentoft AJ, Bahat G, Bauer J, et al. Sarcopenia: revised European consensus on definition and diagnosis. *Age Ageing*. 2019;48(1):16-31.

View publication stats
