# Supplementary material for: Impact of creatine supplementation on inflammation: evidence from a systematic review and meta-analysis of randomized double-blind placebo trials
Source: Front Immunol. 2026 Feb 19;17:1743603. doi: 10.3389/fimmu.2026.1743603 (PMC12961398; doi:10.3389/fimmu.2026.1743603)
Supplement: Supplementary file 2 [file SupplementaryFile1.zip › SR Creatine inflammatory markers (Kell Doutorado). /Supplementary Files/Final References/Kell/Ficha de Triagem completa (1).docx]

**FICHA DE ELEGIBILIDADE (REVISÃO SISTEMÁTICA)**

Etapa leitura da metodologia/resultados dos artigos na íntegra

**Artigo 1**: **Evaluation of Creatine Monohydrate Supplementation on the Gastrocnemius Muscle of**Mice**with Muscular Dystrophy: A Preliminary Study**

Fernandes, V.A.R., dos Santos, G.P., Iatecola, A., Buchaim, D.V., Garcia, I.J.F., Reis, C.H.B., Bueno, L.M.M., Pagani, B.T., Buchaim, R.L., da Cunha, M.R.

**P)** Os participantes possuem mais de 18 anos de idade?

( ) Sim (X) Não

**Justificativa:** Os participantes do estudo foram um total de **20 ratos machos, com 16 semanas de idade**, incluindo 10 ratos MDX (distróficos) e 10 ratos C57BL/10 (saudáveis). Portanto, não são humanos com mais de 18 anos.

**I)** A intervenção utilizada no estudo é creatina? Vale qualquer formato de administração

( ) Sim ( ) Não

**C)** Teve grupo controle?

( ) Sim ( ) Não

**O)** O estudo apresenta valores de marcadores inflamatórios (citocinas e outros marcadores) antes e após as intervenções de creatina?

( ) Sim ( ) Não

**S)** O estudo é um ensaio clínico randomizado e controlado?

( ) Sim ( ) Não

**Artigo 2: Creatine Supplementation Beyond Athletics: Benefits of Different Types of Creatine for Women, Vegans, and Clinical Populations—A Narrative Review**

Gutiérrez-Hellín, J., Del Coso, J., Franco-Andrés, A., Gamonales, J.M., Espada, M.C., González-García, J., López-Moreno, M., Varillas-Delgado, D.

**P)** Os participantes possuem mais de 18 anos de idade?

( ) Sim (X) Não

**Justificativa:** O "Artigo 2" é uma **revisão narrativa** ("A Narrative Review") da literatura existente sobre os benefícios da suplementação de creatina. Como uma revisão, **o estudo não envolveu participantes diretos** em um experimento. Em vez disso, ele explora e resume a evidência científica de estudos anteriores conduzidos por outros pesquisadores. A revisão discute os benefícios da creatina em diversas populações, incluindo **mulheres, veganos e populações clínicas**, o que indiretamente se refere a indivíduos de várias faixas etárias, incluindo adultos com mais de 18 anos. No entanto, a pergunta refere-se aos "participantes" do *presente estudo*, e este estudo, por ser uma revisão, não possui participantes próprios no sentido de sujeitos experimentais.

**I)** A intervenção utilizada no estudo é creatina? Vale qualquer formato de administração

( ) Sim ( ) Não

**C)** Teve grupo controle?

( ) Sim ( ) Não

**O)** O estudo apresenta valores de marcadores inflamatórios (citocinas e outros marcadores) antes e após as intervenções de creatina?

( ) Sim ( ) Não

**S)** O estudo é um ensaio clínico randomizado e controlado?

( ) Sim ( ) Não

**Artigo 3:** **Vitamin D Toxicity from an Unusual and Unexpected Source: A Report of 2 Cases**

Silva, C., Fung, A.W.S., Masson, V., Assen, K., Ward, V., McKenzie, J., Blydt-Hansen, T.D., Cosme, J., Van Der Gugten, G., Barakauskas, V.E., Fox, D.A.

**P)** Os participantes possuem mais de 18 anos de idade?

( ) Sim ( ) Não [*Excluir*]

**I)** A intervenção utilizada no estudo é creatina? Vale qualquer formato de administração

( ) Sim ( ) Não [*Excluir*]

**C)** Teve grupo controle?

( ) Sim ( ) Não [*Excluir*]

**O)** O estudo apresenta valores de marcadores inflamatórios (citocinas e outros marcadores) antes e após as intervenções de creatina?

( ) Sim ( ) Não [*Excluir*]

**S)** O estudo é um ensaio clínico randomizado e controlado?

( ) Sim ( ) Não [*Excluir*]

# Artigo 4: A case of pulmonary thromboembolism possibly associated with the use of creatine supplements

Lee, S.H., Seo, J.A., Park, J.E., Kim, C.H., Lee, J.

**P)** Os participantes possuem mais de 18 anos de idade?

(X) Sim ( ) Não

O estudo é um **relato de caso** que descreve um **homem de 24 anos.**

“A 24-year-old non-smoker male presented to the emergency department with sudden-onset dypnoea and chest discomfort.”

**I)** A intervenção utilizada no estudo é creatina? Vale qualquer formato de administração

(X) Sim ( ) Não

O paciente em questão estava a tomar **suplementos contendo creatina monohidratada** diariamente para aumentar a sua massa muscular. Ele tinha recentemente aumentado a dose para **mais de 20 g/dia de creatina monohidratada**

**C)** Teve grupo controle?

( ) Sim (X) Não

Conforme indicado no título e na descrição, este é um **"relato de caso",** tendo o estudo sido conduzido somente perante um indivíduo.

**O)** O estudo apresenta valores de marcadores inflamatórios (citocinas e outros marcadores) antes e após as intervenções de creatina?

( ) Sim ( ) Não

**S)** O estudo é um ensaio clínico randomizado e controlado?

( ) Sim ( ) Não

**Artigo 5:** **Role of Creatine Supplementation in Alleviating Hepatotoxicity Caused by Doxorubicin**

Aljobaily, N.A., Viereckl, M., Aljobaily, H.A., Albertson, J.A., Han, Y., Hydock, D.

**P)** Os participantes possuem mais de 18 anos de idade?

( ) Sim (X) Não

“Sprague-Dawley rats (Rattus norvegicus) were used as a model organism"

**I)** A intervenção utilizada no estudo é creatina? Vale qualquer formato de administração

( ) Sim ( ) Não

**C)** Teve grupo controle?

( ) Sim ( ) Não

**O)** O estudo apresenta valores de marcadores inflamatórios (citocinas e outros marcadores) antes e após as intervenções de creatina?

( ) Sim ( ) Não

**S)** O estudo é um ensaio clínico randomizado e controlado?

( ) Sim ( ) Não

Artigo 6: **No effect of creatine monohydrate supplementation on inflammatory and cartilage degradation biomarkers in individuals with knee osteoarthritis**

Cornish, S.M., Peeler, J.D.

**P)** Os participantes possuem mais de 18 anos de idade?

(X) Sim ( ) Não

“Inclusion criteria included: (1) ages 45–65”

**I)** A intervenção utilizada no estudo é creatina? Vale qualquer formato de administração

(X) Sim ( ) Não

The study purpose was to evaluate the effectiveness of creatine monohydrate supplementation (20 grams/day for 1 week and then 5 grams/day for 11 weeks)".

Transcrição: "(Group 1) Participants supplemented their regular diet with creatine monohydrate (Creapure®, AlzChem AG, Trostberg, Germany) for 12 consecutive weeks"

**C)** Teve grupo controle?

(X) Sim ( ) Não

"A total of 18 patients with mild to moderate knee osteoarthritis were recruited and randomized in a double blind fashion to either a creatine supplementation group (N = 9) or a placebo (N = 9)".

"Participants in the placebo group supplemented their regular diet with maltodextrin (a sugar based molecule) for 12 consecutive weeks".

**O)** O estudo apresenta valores de marcadores inflamatórios (citocinas e outros marcadores) antes e após as intervenções de creatina?

(X) Sim ( ) Não

"At **baseline and after 12 weeks** of supplementation patients had **inflammatory and cartilage degradation biomarkers measured** in the systemic blood".

"Table 2 – **Baseline (pre) and 12-week (post) concentrations for inflammatory and cartilage degradation biomarkers** in individuals diagnosed with mild to moderate knee osteoarthritis".

"Plasma was analyzed for a variety of **cytokines including: interleukin-1 beta (IL-1β), IL-6, tumor necrosis factor alpha (TNF-α), and s100 A8/A9** using enzyme linked immunosorbent assay (ELISA) kits... Also, plasma was analyzed for the acute phase protein **C-reactive protein** using an ELISA kit... Serum was analyzed for **serum cartilage oligomeric matrix protein (sCOMP)**".

**S)** O estudo é um ensaio clínico randomizado e controlado?

(X) Sim ( ) Não

"Therefore, the primary research objective of this **randomized, placebo controlled trial** was to compare the resting blood inflammatory biomarkers...".

"A total of 18 patients with mild to moderate knee osteoarthritis were recruited and **randomized in a double blind fashion** to either a creatine supplementation group (N = 9) or a placebo (N = 9)"

**Artigo 7: Exercise induced fatigue: McArdle disease in an adult patient with myopathy**

Aydogdu, S., Uskudar Cansu, D., Kilic Yildirim, G., Erdogan, B

**P)** Os participantes possuem mais de 18 anos de idade?

( ) Sim ( ) Não

**I)** A intervenção utilizada no estudo é creatina? Vale qualquer formato de administração

( ) Sim ( ) Não

**C)** Teve grupo controle?

( ) Sim ( ) Não

**O)** O estudo apresenta valores de marcadores inflamatórios (citocinas e outros marcadores) antes e após as intervenções de creatina?

( ) Sim ( ) Não

**S)** O estudo é um ensaio clínico randomizado e controlado?

( ) Sim ( ) Não

**Artigo 8: The role of dietary creatine**

Brosnan, M.E., Brosnan, J.T.

**P)** Os participantes possuem mais de 18 anos de idade?

( ) Sim (X) Não

Este artigo é uma **revisão narrativa** da literatura existente sobre a creatina. Como tal, **o estudo não envolveu participantes diretos em um experimento próprio**. Ele discute a ingestão e os efeitos da creatina em várias populações, incluindo "infants" (bebês), "rodent model" (modelo de roedores), "rats" (ratos), "mice" (camundongos), e também "adults" (adultos). A pergunta refere-se aos "participantes" do *presente estudo*, e este estudo, por ser uma revisão, não possui participantes no sentido de sujeitos experimentais.

"**MINIREVIEW ARTICLE**".

"Infants receive creatine in mother’s milk or in milk-based formulas. Vegans and infants fed on soy-based formulas receive no dietary creatine.".

"Creatine supplementation prevents the accumulation of fat in the livers of **rats** fed a high-fat diet.".

"Creatine supplementation in **mouse** colitis models has been shown to attenuate the inflammatory response".

**I)** A intervenção utilizada no estudo é creatina? Vale qualquer formato de administração

( ) Sim ( ) Não

**C)** Teve grupo controle?

( ) Sim ( ) Não

**O)** O estudo apresenta valores de marcadores inflamatórios (citocinas e outros marcadores) antes e após as intervenções de creatina?

( ) Sim ( ) Não

**S)** O estudo é um ensaio clínico randomizado e controlado?

( ) Sim ( ) Não

**Artigo 9: Effect of creatine monohydrate supplementation on relative serum level of IL-6 and IL-18 following neonatal hypoxia ischemia in male albino**mouse

Iqbal, S., Ali, M., Iqbal, F.

**P)** Os participantes possuem mais de 18 anos de idade?

( ) Sim ( ) Não

**I)** A intervenção utilizada no estudo é creatina? Vale qualquer formato de administração

( ) Sim ( ) Não

**C)** Teve grupo controle?

( ) Sim ( ) Não

**O)** O estudo apresenta valores de marcadores inflamatórios (citocinas e outros marcadores) antes e após as intervenções de creatina?

( ) Sim ( ) Não

**S)** O estudo é um ensaio clínico randomizado e controlado?

( ) Sim ( ) Não

**Artigo 10: Toxic hepatitis in a group of 20 male body-builders taking dietary supplements**

Timcheh-Hariri, A., Balali-Mood, M., Aryan, E., Sadeghi, M., Riahi-Zanjani, B.

**P)** Os participantes possuem mais de 18 anos de idade?

(X) Sim ( ) Não

"The patients’ ages ranged from **24 to 32** with a mean of 28 years".

**I)** A intervenção utilizada no estudo é creatina? Vale qualquer formato de administração

( ) Sim (X) Não

"We investigated and reported toxic hepatitis in 20 male athletes following **self-prescribing of a number of dietary supplements** which are lesser known. The patients’ ages ranged from 24 to 32 with a mean of 28 years. They had taken three kinds of supplements for 1 year including testosterone optimizer agent T Bomb II, **a creatine supplement Phosphagen** and an amino acid based supplement Cell-Tech".

"In this study, we documented the cases of 20 male body-builders with no past medical history, who developed hepatitis secondary to **chronic ingestion of Phosphagen, a creatine supplement** (Fig. 1); T- Bomb II, an optimizer of testosterone production (Fig. 2) and Cell-Tech, an amino acid and creatine-based supplement (Fig. 3) for 1 year"

**C)** Teve grupo controle?

( ) Sim ( ) Não

**O)** O estudo apresenta valores de marcadores inflamatórios (citocinas e outros marcadores) antes e após as intervenções de creatina?

( ) Sim ( ) Não

**S)** O estudo é um ensaio clínico randomizado e controlado?

( ) Sim ( ) Não

**Artigo 11:** **Creatine as a therapeutic strategy for myopathies**

Tarnopolsky, M.A.

**P)** Os participantes possuem mais de 18 anos de idade?

( ) Sim (X) Não

O artigo de revisão cita um estudo de Escolar et al. (2005) onde os participantes tinham "idade **acima de 7 anos** no grupo creatina", o que indica que nem todos possuíam mais de 18 anos.

**I)** A intervenção utilizada no estudo é creatina? Vale qualquer formato de administração

( ) Sim ( ) Não

**C)** Teve grupo controle?

( ) Sim ( ) Não

**O)** O estudo apresenta valores de marcadores inflamatórios (citocinas e outros marcadores) antes e após as intervenções de creatina?

( ) Sim ( ) Não

**S)** O estudo é um ensaio clínico randomizado e controlado?

( ) Sim ( ) Não

**Artigo 12: Resistance training and dietary supplements as intervention for regaining muscle mass following radiotherapy in head and neck cancer patients**

Lønbro, S., Overgaard, K., Primdahl, H., Johansen, J., Overgaard, J.

**P)** Os participantes possuem mais de 18 anos de idade?

( ) Sim ( ) Não

**I)** A intervenção utilizada no estudo é creatina? Vale qualquer formato de administração

( ) Sim ( ) Não

**C)** Teve grupo controle?

( ) Sim ( ) Não

**O)** O estudo apresenta valores de marcadores inflamatórios (citocinas e outros marcadores) antes e após as intervenções de creatina?

( ) Sim ( ) Não

**S)** O estudo é um ensaio clínico randomizado e controlado?

( ) Sim ( ) Não

**Artigo 13:** **When “creatine” increases creatinine**

Barlapudi, S., Soltani, Z.

**P)** Os participantes possuem mais de 18 anos de idade?

( ) Sim ( ) Não

**I)** A intervenção utilizada no estudo é creatina? Vale qualquer formato de administração

( ) Sim ( ) Não

**C)** Teve grupo controle?

( ) Sim ( ) Não

**O)** O estudo apresenta valores de marcadores inflamatórios (citocinas e outros marcadores) antes e após as intervenções de creatina?

( ) Sim ( ) Não

**S)** O estudo é um ensaio clínico randomizado e controlado?

( ) Sim ( ) Não

**Artigo 14:** Severe intrahepatic cholestasis and renal failure due to an anabolic androgenic dietary supplement

Raines, Alissa PharmD; Dick, Travis PharmD; Seaman, James MD; Harmston, Gordon MD

**P)** Os participantes possuem mais de 18 anos de idade?

(X) Sim ( ) Não

“We report two cases of otherwise healthy males, age 49 years (patient 1) and 21 years (patient 2)”

**I)** A intervenção utilizada no estudo é creatina? Vale qualquer formato de administração

(X) Sim ( ) Não

“Neither patient took prescription medications prior to symptom onset, but patient 1 took a creatine supplement.”

**C)** Teve grupo controle?

( ) Sim (X) Não

**O)** O estudo apresenta valores de marcadores inflamatórios (citocinas e outros marcadores) antes e após as intervenções de creatina?

( ) Sim ( ) Não

**S)** O estudo é um ensaio clínico randomizado e controlado?

( ) Sim ( ) Não

**Artigo 15:** Creatine supplementation exacerbates allergic lung inflammation and airway remodeling in mice

[Rodolfo P Vieira](https://pubmed.ncbi.nlm.nih.gov/?sort=date&term=Vieira+RP&cauthor_id=17641295)[1](https://pubmed.ncbi.nlm.nih.gov/17641295/#full-view-affiliation-1), [Anna Cecília S Duarte](https://pubmed.ncbi.nlm.nih.gov/?sort=date&term=Duarte+AC&cauthor_id=17641295), [Renata C Claudino](https://pubmed.ncbi.nlm.nih.gov/?sort=date&term=Claudino+RC&cauthor_id=17641295), [Adenir Perini](https://pubmed.ncbi.nlm.nih.gov/?sort=date&term=Perini+A&cauthor_id=17641295), [Angela B G Santos](https://pubmed.ncbi.nlm.nih.gov/?sort=date&term=Santos+AB&cauthor_id=17641295), [Henrique T Moriya](https://pubmed.ncbi.nlm.nih.gov/?sort=date&term=Moriya+HT&cauthor_id=17641295), [Fernanda M Arantes-Costa](https://pubmed.ncbi.nlm.nih.gov/?sort=date&term=Arantes-Costa+FM&cauthor_id=17641295), [Mílton A Martins](https://pubmed.ncbi.nlm.nih.gov/?sort=date&term=Martins+MA&cauthor_id=17641295), [Celso R F Carvalho](https://pubmed.ncbi.nlm.nih.gov/?sort=date&term=Carvalho+CR&cauthor_id=17641295), [Marisa Dolhnikoff](https://pubmed.ncbi.nlm.nih.gov/?sort=date&term=Dolhnikoff+M&cauthor_id=17641295)

**P)** Os participantes possuem mais de 18 anos de idade?

( ) Sim (X) Não

“Thirty-one male Balb/c mice (20-25 g)”

**I)** A intervenção utilizada no estudo é creatina? Vale qualquer formato de administração

( ) Sim ( ) Não

**C)** Teve grupo controle?

( ) Sim ( ) Não

**O)** O estudo apresenta valores de marcadores inflamatórios (citocinas e outros marcadores) antes e após as intervenções de creatina?

( ) Sim ( ) Não

**S)** O estudo é um ensaio clínico randomizado e controlado?

( ) Sim ( ) Não

**Artigo 16:** Creatine monohydrate supplementation does not improve functional recovery after total knee arthroplasty

[Brian D Roy](https://pubmed.ncbi.nlm.nih.gov/?sort=date&term=Roy+BD&cauthor_id=16003653)[1](https://pubmed.ncbi.nlm.nih.gov/16003653/#full-view-affiliation-1), [Justin de Beer](https://pubmed.ncbi.nlm.nih.gov/?sort=date&term=de+Beer+J&cauthor_id=16003653), [David Harvey](https://pubmed.ncbi.nlm.nih.gov/?sort=date&term=Harvey+D&cauthor_id=16003653), [Mark A Tarnopolsky](https://pubmed.ncbi.nlm.nih.gov/?sort=date&term=Tarnopolsky+MA&cauthor_id=16003653)

**P)** Os participantes possuem mais de 18 anos de idade?

(X) Sim ( ) Não

“The study participants were 37 individuals, 17 men and 20 women.”

**I)** A intervenção utilizada no estudo é creatina? Vale qualquer formato de administração

(X) Sim ( ) Não

“The intervention involved participants randomized to receive 7g creatine monohydrate + 4g dextrose + flavoring.”

**C)** Teve grupo controle?

(X) Sim ( ) Não

“The study included a control group that received identical satchels (7g dextrose + flavoring) as a placebo”

**O)** O estudo apresenta valores de marcadores inflamatórios (citocinas e outros marcadores) antes e após as intervenções de creatina?

( ) Sim (X) Não

The provided excerpts do not mention the measurement of inflammatory markers (cytokines or other markers) before and after the interventions.

**S)** O estudo é um ensaio clínico randomizado e controlado?

( ) Sim ( ) Não

**Artigo 17:** Histological assessment of intermediate- and long-term creatine monohydrate supplementation in mice and rats

Tarnopolsky, M.A., Bourgeois, J.M., Snow, R., Keys, S., Roy, B.D., Kwiecien, J.M., Turnbull, J.

**P)** Os participantes possuem mais de 18 anos de idade?

( ) Sim (X) Não

“The participants in this study included “CD-1 female mice (...) at 15 wk of age”

“Young male Sprague-Dawley animals”

**I)** A intervenção utilizada no estudo é creatina? Vale qualquer formato de administração

( ) Sim ( ) Não

**C)** Teve grupo controle?

( ) Sim ( ) Não

**O)** O estudo apresenta valores de marcadores inflamatórios (citocinas e outros marcadores) antes e após as intervenções de creatina?

( ) Sim ( ) Não

**S)** O estudo é um ensaio clínico randomizado e controlado?

( ) Sim ( ) Não

**Artigo 18:** Creatine transporter and mitochondrial creatine kinase protein content in myopathies

[M A Tarnopolsky](https://pubmed.ncbi.nlm.nih.gov/?sort=date&term=Tarnopolsky+MA&cauthor_id=11317279)[1](https://pubmed.ncbi.nlm.nih.gov/11317279/#full-view-affiliation-1), [A Parshad](https://pubmed.ncbi.nlm.nih.gov/?sort=date&term=Parshad+A&cauthor_id=11317279), [B Walzel](https://pubmed.ncbi.nlm.nih.gov/?sort=date&term=Walzel+B&cauthor_id=11317279), [U Schlattner](https://pubmed.ncbi.nlm.nih.gov/?sort=date&term=Schlattner+U&cauthor_id=11317279), [T Wallimann](https://pubmed.ncbi.nlm.nih.gov/?sort=date&term=Wallimann+T&cauthor_id=11317279)

**P)** Os participantes possuem mais de 18 anos de idade?

(X) Sim ( ) Não

"The subjects in the study, across different groups like Control, Mitochondrial myopathy, Inflammatory myopathy, Muscular dystrophy, and Congenital myopathy, had mean ages ranging from "35.7 ± 16.2" to "59.3 ± 11.8" years, indicating they were adults."

**I)** A intervenção utilizada no estudo é creatina? Vale qualquer formato de administração

( ) Sim (X) Não

The study focuses on evaluating "Transporter and mitochondrial creatine kinase protein content in myopathies" from muscle biopsies and does not describe creatine supplementation as an intervention administered to the participants within this specific study."

**C)** Teve grupo controle?

( ) Sim ( ) Não

**O)** O estudo apresenta valores de marcadores inflamatórios (citocinas e outros marcadores) antes e após as intervenções de creatina?

( ) Sim ( ) Não

**S)** O estudo é um ensaio clínico randomizado e controlado?

( ) Sim ( ) Não

**Artigo 19: Creatine Supplementation Mitigates Doxorubicin-Induced Skeletal Muscle Dysfunction but Not Cardiotoxicity.**

Cella, Paola Sanches, de Matos, Ricardo Luís Nascimento, Marinello, Poliana Camila, Guimarães, T A S, Nunes, J H C, Moura, Felipe Arruda, Bracarense, Ana Paula Frederico Rodrigues Loureiro, Chimin, Patrícia, Deminice, Rafael

**P)** Os participantes possuem mais de 18 anos de idade?

( ) Sim ( ) Não [*Excluir*]

**I)** A intervenção utilizada no estudo é creatina? Vale qualquer formato de administração

( ) Sim ( ) Não [*Excluir*]

**C)** Teve grupo controle?

( ) Sim ( ) Não [*Excluir*]

**O)** O estudo apresenta valores de marcadores inflamatórios (citocinas e outros marcadores) antes e após as intervenções de creatina?

( ) Sim ( ) Não [*Excluir*]

**S)** O estudo é um ensaio clínico randomizado e controlado?

( ) Sim ( ) Não [*Excluir*]

**Artigo 20:** Influence of CReatine Supplementation on mUScle Mass and Strength After Stroke (ICaRUS Stroke Trial): A Randomized Controlled Trial.

[Juli Thomaz de Souza](https://pubmed.ncbi.nlm.nih.gov/?sort=date&term=de+Souza+JT&cauthor_id=36949443)[1](https://pubmed.ncbi.nlm.nih.gov/36949443/#full-view-affiliation-1), [Marcos F Minicucci](https://pubmed.ncbi.nlm.nih.gov/?sort=date&term=Minicucci+MF&cauthor_id=36949443)[2](https://pubmed.ncbi.nlm.nih.gov/36949443/#full-view-affiliation-2), [Natália C Ferreira](https://pubmed.ncbi.nlm.nih.gov/?sort=date&term=Ferreira+NC&cauthor_id=36949443)[2](https://pubmed.ncbi.nlm.nih.gov/36949443/#full-view-affiliation-2), [Bertha F Polegato](https://pubmed.ncbi.nlm.nih.gov/?sort=date&term=Polegato+BF&cauthor_id=36949443)[2](https://pubmed.ncbi.nlm.nih.gov/36949443/#full-view-affiliation-2), [Marina Politi Okoshi](https://pubmed.ncbi.nlm.nih.gov/?sort=date&term=Okoshi+MP&cauthor_id=36949443)[2](https://pubmed.ncbi.nlm.nih.gov/36949443/#full-view-affiliation-2), [Gabriel P Modolo](https://pubmed.ncbi.nlm.nih.gov/?sort=date&term=Modolo+GP&cauthor_id=36949443)[3](https://pubmed.ncbi.nlm.nih.gov/36949443/#full-view-affiliation-3), [Bethan E Phillips](https://pubmed.ncbi.nlm.nih.gov/?sort=date&term=Phillips+BE&cauthor_id=36949443)[4](https://pubmed.ncbi.nlm.nih.gov/36949443/#full-view-affiliation-4), [Philip J Atherton](https://pubmed.ncbi.nlm.nih.gov/?sort=date&term=Atherton+PJ&cauthor_id=36949443)[4](https://pubmed.ncbi.nlm.nih.gov/36949443/#full-view-affiliation-4), [Kenneth Smith](https://pubmed.ncbi.nlm.nih.gov/?sort=date&term=Smith+K&cauthor_id=36949443)[4](https://pubmed.ncbi.nlm.nih.gov/36949443/#full-view-affiliation-4), [Daniel Wilkinson](https://pubmed.ncbi.nlm.nih.gov/?sort=date&term=Wilkinson+D&cauthor_id=36949443)[4](https://pubmed.ncbi.nlm.nih.gov/36949443/#full-view-affiliation-4), [Adam Gordon](https://pubmed.ncbi.nlm.nih.gov/?sort=date&term=Gordon+A&cauthor_id=36949443)[4](https://pubmed.ncbi.nlm.nih.gov/36949443/#full-view-affiliation-4), [Suzana E Tanni](https://pubmed.ncbi.nlm.nih.gov/?sort=date&term=Tanni+SE&cauthor_id=36949443)[2](https://pubmed.ncbi.nlm.nih.gov/36949443/#full-view-affiliation-2), [Vladimir Eliodoro Costa](https://pubmed.ncbi.nlm.nih.gov/?sort=date&term=Costa+VE&cauthor_id=36949443)[5](https://pubmed.ncbi.nlm.nih.gov/36949443/#full-view-affiliation-5), [Maria Fernanda P Fernandes](https://pubmed.ncbi.nlm.nih.gov/?sort=date&term=Fernandes+MFP&cauthor_id=36949443)[2](https://pubmed.ncbi.nlm.nih.gov/36949443/#full-view-affiliation-2), [Silméia G Zanati Bazan](https://pubmed.ncbi.nlm.nih.gov/?sort=date&term=Bazan+SGZ&cauthor_id=36949443)[2](https://pubmed.ncbi.nlm.nih.gov/36949443/#full-view-affiliation-2), [Leonardo A M Zornoff](https://pubmed.ncbi.nlm.nih.gov/?sort=date&term=Zornoff+LAM&cauthor_id=36949443)[2](https://pubmed.ncbi.nlm.nih.gov/36949443/#full-view-affiliation-2), [Rodrigo Bazan](https://pubmed.ncbi.nlm.nih.gov/?sort=date&term=Bazan+R&cauthor_id=36949443)[3](https://pubmed.ncbi.nlm.nih.gov/36949443/#full-view-affiliation-3), [Sérgio A Rupp de Paiva](https://pubmed.ncbi.nlm.nih.gov/?sort=date&term=de+Paiva+SAR&cauthor_id=36949443)[2](https://pubmed.ncbi.nlm.nih.gov/36949443/#full-view-affiliation-2), [Paula Schmidt Azevedo](https://pubmed.ncbi.nlm.nih.gov/?sort=date&term=Azevedo+PS&cauthor_id=36949443)[2](https://pubmed.ncbi.nlm.nih.gov/36949443/#full-view-affiliation-2)

**P)** Os participantes possuem mais de 18 anos de idade?

(X) Sim ( ) Não

"The study protocol states that "Men and women aged 60 years and older who were diagnosed with ischemic stroke" will be recruited."

**I)** A intervenção utilizada no estudo é creatina? Vale qualquer formato de administração

(X) Sim ( ) Não

"The intervention group was designed for patients to "intake one sachet containing 10g of creatine twice a day"

**C)** Teve grupo controle?

(X) Sim ( ) Não

"The study includes a control group where patients "will intake one sachet containing 10g of placebo [maltodextrin] twice a day"

**O)** O estudo apresenta valores de marcadores inflamatórios (citocinas e outros marcadores) antes e após as intervenções de creatina?

( ) Sim (X) Não

"The primary outcomes listed are "functional capacity, strength, and changes in muscle mass after the intervention as assessed by (...) and identification of muscle degradation markers by D3-methylhistidine". There is no mention of inflammatory markers being assessed.

**S)** O estudo é um ensaio clínico randomizado e controlado?

( ) Sim ( ) Não

**Artigo 21: Effect of Creatine Supplementation on Body Composition and Malnutrition-Inflammation Score in Hemodialysis Patients: An Exploratory 1-Year, Balanced, Double-Blind Design.**

Marini, Ana Clara B, Schincaglia, Raquel M, Candow, Darren G, Pimentel, Gustavo D

**P)** Os participantes possuem mais de 18 anos de idade?

(X) Sim ( ) Não

"An exploratory 1-year balanced, placebo-controlled, and double-blind design was conducted with hemodialysis patients **(≥18 years)**".

"This exploratory 1-year, balanced, placebo-controlled, and double-blind design was conducted with patients of both sexes diagnosed with CKD undergoing HD, and **older than 18 years**"

**I)** A intervenção utilizada no estudo é creatina? Vale qualquer formato de administração

(X) Sim ( ) Não

"to evaluate the effect of **creatine supplementation** on the body composition and Malnutrition-Inflammation Score (MIS) in patients with chronic kidney disease (CKD) undergoing hemodialysis". O grupo de creatina (CG) recebeu "**5 g of creatine monohydrate** and 5 g of maltodextrin per day". A intervenção foi administrada através de "sachets containing **either creatine or placebo**"

**C)** Teve grupo controle?

(X) Sim ( ) Não

O design do estudo é explicitamente descrito como um "**placebo-controlled**, and double-blind design". O estudo incluiu um "Placebo Group (PG) composed of 19 patients who received maltodextrin", servindo como grupo controle em comparação ao "Creatine Group (CG) composed of 21 patients who received creatine"

**O)** O estudo apresenta valores de marcadores inflamatórios (citocinas e outros marcadores) antes e após as intervenções de creatina?

(X) Sim ( ) Não

"**Malnutrition-Inflammation Score (MIS)**" em "three time points: pre, intermediate (after 6 months), and post (after 12 months)"

**S)** O estudo é um ensaio clínico randomizado e controlado?

(X) Sim ( ) Não

"**exploratory 1-year, balanced, placebo-controlled, and double-blind design**"

**Artigo 22:** **Creatine supplementation increases postnatal growth and strength and prevents overexpression of pro-inflammatory interleukin 6 in the hippocampus in an experimental model of cerebral palsy.**

Gouveia, Henrique J C B, Manhães-de-Castro, Raul, Costa-de-Santana, Bárbara J R, Vasconcelos, Emanuel Ewerton M, Silva, Eliesly Roberto, Roque, Angélica, Torner, Luz, Guzmán-Quevedo, Omar, Toscano, Ana E

**P)** Os participantes possuem mais de 18 anos de idade?

( ) Sim ( ) Não [*Excluir*]

**I)** A intervenção utilizada no estudo é creatina? Vale qualquer formato de administração

( ) Sim ( ) Não [*Excluir*]

**C)** Teve grupo controle?

( ) Sim ( ) Não [*Excluir*]

**O)** O estudo apresenta valores de marcadores inflamatórios (citocinas e outros marcadores) antes e após as intervenções de creatina?

( ) Sim ( ) Não [*Excluir*]

**S)** O estudo é um ensaio clínico randomizado e controlado?

( ) Sim ( ) Não [*Excluir*]

**Artigo 23:** Creatine Supplementation Potentiates Exercise Protective Effects against Doxorubicin-Induced Hepatotoxicity in Mice.

[Loriane R L Costa Godinho](https://pubmed.ncbi.nlm.nih.gov/?sort=date&term=Costa+Godinho+LRL&cauthor_id=37107198)[1](https://pubmed.ncbi.nlm.nih.gov/37107198/#full-view-affiliation-1), [Paola S Cella](https://pubmed.ncbi.nlm.nih.gov/?sort=date&term=Cella+PS&cauthor_id=37107198)[1](https://pubmed.ncbi.nlm.nih.gov/37107198/#full-view-affiliation-1), [Tatiana A S Guimarães](https://pubmed.ncbi.nlm.nih.gov/?sort=date&term=Guimar%C3%A3es+TAS&cauthor_id=37107198)[1](https://pubmed.ncbi.nlm.nih.gov/37107198/#full-view-affiliation-1), [Guilherme H Dantas Palma](https://pubmed.ncbi.nlm.nih.gov/?sort=date&term=Palma+GHD&cauthor_id=37107198)[1](https://pubmed.ncbi.nlm.nih.gov/37107198/#full-view-affiliation-1), [Jonathan H C Nunes](https://pubmed.ncbi.nlm.nih.gov/?sort=date&term=Nunes+JHC&cauthor_id=37107198)[1](https://pubmed.ncbi.nlm.nih.gov/37107198/#full-view-affiliation-1), [Rafael Deminice](https://pubmed.ncbi.nlm.nih.gov/?sort=date&term=Deminice+R&cauthor_id=37107198)[1](https://pubmed.ncbi.nlm.nih.gov/37107198/#full-view-affiliation-1)

**P)** Os participantes possuem mais de 18 anos de idade?

( ) Sim (X) Não

"The study was approved by the "Ethics Committee for Animal Use" and the participants are referred to as "Mice"."

**I)** A intervenção utilizada no estudo é creatina? Vale qualquer formato de administração

( ) Sim ( ) Não

**C)** Teve grupo controle?

( ) Sim ( ) Não

**O)** O estudo apresenta valores de marcadores inflamatórios (citocinas e outros marcadores) antes e após as intervenções de creatina?

( ) Sim ( ) Não

**S)** O estudo é um ensaio clínico randomizado e controlado?

( ) Sim ( ) Não

**Artigo 24:** Creatine supplementation to improve the peripheral and central inflammatory profile in cerebral palsy.

[Henrique J C B Gouveia](https://pubmed.ncbi.nlm.nih.gov/?sort=date&term=Gouveia+HJCB&cauthor_id=36513462)[1](https://pubmed.ncbi.nlm.nih.gov/36513462/#full-view-affiliation-1), [Raul Manhães-de-Castro](https://pubmed.ncbi.nlm.nih.gov/?sort=date&term=Manh%C3%A3es-de-Castro+R&cauthor_id=36513462)[1](https://pubmed.ncbi.nlm.nih.gov/36513462/#full-view-affiliation-1), [Diego Cabral Lacerda](https://pubmed.ncbi.nlm.nih.gov/?sort=date&term=Lacerda+DC&cauthor_id=36513462)[2](https://pubmed.ncbi.nlm.nih.gov/36513462/#full-view-affiliation-2), [Ana Elisa Toscano](https://pubmed.ncbi.nlm.nih.gov/?sort=date&term=Toscano+AE&cauthor_id=36513462)[3](https://pubmed.ncbi.nlm.nih.gov/36513462/#full-view-affiliation-3)

**P)** Os participantes possuem mais de 18 anos de idade?

( ) Sim (X) Não

“Results obtained in these preclinical studies may contribute to the quality of therapeutic strategies offered to children suffering from CP, the most common cause of chronic motor disability in childhood.”

**I)** A intervenção utilizada no estudo é creatina? Vale qualquer formato de administração

( ) Sim ( ) Não

**C)** Teve grupo controle?

( ) Sim ( ) Não

**O)** O estudo apresenta valores de marcadores inflamatórios (citocinas e outros marcadores) antes e após as intervenções de creatina?

( ) Sim ( ) Não

**S)** O estudo é um ensaio clínico randomizado e controlado?

( ) Sim ( ) Não

**Artigo 25:** Creatine supplementation protects against diet-induced non-alcoholic fatty liver but exacerbates alcoholic fatty liver.

[Poliana C Marinello](https://pubmed.ncbi.nlm.nih.gov/?sort=date&term=Marinello+PC&cauthor_id=36220368)[1](https://pubmed.ncbi.nlm.nih.gov/36220368/#full-view-affiliation-1), [Paola S Cella](https://pubmed.ncbi.nlm.nih.gov/?sort=date&term=Cella+PS&cauthor_id=36220368)[2](https://pubmed.ncbi.nlm.nih.gov/36220368/#full-view-affiliation-2), [Mayra T J Testa](https://pubmed.ncbi.nlm.nih.gov/?sort=date&term=Testa+MTJ&cauthor_id=36220368)[2](https://pubmed.ncbi.nlm.nih.gov/36220368/#full-view-affiliation-2), [Phillipe B Guirro](https://pubmed.ncbi.nlm.nih.gov/?sort=date&term=Guirro+PB&cauthor_id=36220368)[2](https://pubmed.ncbi.nlm.nih.gov/36220368/#full-view-affiliation-2), [Walison Augusto da Silva Brito](https://pubmed.ncbi.nlm.nih.gov/?sort=date&term=da+Silva+Brito+WA&cauthor_id=36220368)[3](https://pubmed.ncbi.nlm.nih.gov/36220368/#full-view-affiliation-3), [Camila S Padilha](https://pubmed.ncbi.nlm.nih.gov/?sort=date&term=Padilha+CS&cauthor_id=36220368)[2](https://pubmed.ncbi.nlm.nih.gov/36220368/#full-view-affiliation-2), [Alessandra L Cecchini](https://pubmed.ncbi.nlm.nih.gov/?sort=date&term=Cecchini+AL&cauthor_id=36220368)[3](https://pubmed.ncbi.nlm.nih.gov/36220368/#full-view-affiliation-3), [Robin P da Silva](https://pubmed.ncbi.nlm.nih.gov/?sort=date&term=da+Silva+RP&cauthor_id=36220368)[4](https://pubmed.ncbi.nlm.nih.gov/36220368/#full-view-affiliation-4), [José Alberto R Duarte](https://pubmed.ncbi.nlm.nih.gov/?sort=date&term=Duarte+JAR&cauthor_id=36220368)[5](https://pubmed.ncbi.nlm.nih.gov/36220368/#full-view-affiliation-5), [Rafael Deminice](https://pubmed.ncbi.nlm.nih.gov/?sort=date&term=Deminice+R&cauthor_id=36220368)[6](https://pubmed.ncbi.nlm.nih.gov/36220368/#full-view-affiliation-6)

**P)** Os participantes possuem mais de 18 anos de idade?

( ) Sim (X) Não

"The study describes the participants as "male Swiss mice" and states that "All animal experiments used in this study are strictly adhered to the international standards for the care and use of laboratory animals"."

**I)** A intervenção utilizada no estudo é creatina? Vale qualquer formato de administração

( ) Sim ( ) Não

**C)** Teve grupo controle?

( ) Sim ( ) Não

**O)** O estudo apresenta valores de marcadores inflamatórios (citocinas e outros marcadores) antes e após as intervenções de creatina?

( ) Sim ( ) Não

**S)** O estudo é um ensaio clínico randomizado e controlado?

( ) Sim ( ) Não

**Artigo 26:** **Short-Term Creatine Supplementation May Alleviate the Malnutrition-Inflammation Score and Lean Body Mass Loss in Hemodialysis Patients: A Pilot**RandomizedPlacebo**-**Controlled Trial**.**

Marini, Ana Clara B, Motobu, Reika D, Freitas, Ana T V, Mota, João F, Wall, Benjamin T, Pichard, Claude, Laviano, Alessandro, Pimentel, Gustavo Duarte

**P)** Os participantes possuem mais de 18 anos de idade?

(X) Sim ( ) Não

"A randomized, placebo-controlled, double blind, parallel-design study included HD patients, of both sexes, **aged 18–59 years**"

**I)** A intervenção utilizada no estudo é creatina? Vale qualquer formato de administração

(X) Sim ( ) Não

"our objective was to evaluate whether **creatine supplementation** could attenuate the loss of lean body mass (LBM) and malnutrition-inflammation score (MIS) in HD patients"

"**creatine plus maltodextrin, 1st week: 20 g/day of creatine plus 20 g/day of maltodextrin and 2nd–4th weeks: 5 g/day of creatine plus 5 g/day of maltodextrin**"

**C)** Teve grupo controle?

(X) Sim ( ) Não

"**randomized, placebo-controlled, double blind**, parallel-design study".

"**Placebo Group (PG)**, composed of 15 patients which received maltodextrin"

**O)** O estudo apresenta valores de marcadores inflamatórios (citocinas e outros marcadores) antes e após as intervenções de creatina?

(X) Sim ( ) Não

"**Malnutrition-Inflammation Score (MIS)**"

A Tabela 4 do artigo apresenta os valores de **MIS** "**Pre intervention**" e "**Post intervention**" para ambos os grupos, mostrando uma "significant reduction in CG (: −1.71) compared with PG (:−0.36) (P= .01, with high effect size)"

**S)** O estudo é um ensaio clínico randomizado e controlado?

(X) Sim ( ) Não

"**A randomized, placebo-controlled, double blind, parallel-design study**".

"This **randomized, placebo-controlled, and double-blind clinical trial** was conducted...".

"**randomly allocated** by gender, age, and LBM content"

**Artigo 27:** Creatine supplementation in Walker-256 tumor-bearing rats prevents skeletal muscle atrophy by attenuating systemic inflammation and protein degradation signaling.

[Paola S Cella](https://pubmed.ncbi.nlm.nih.gov/?sort=date&term=Cella+PS&cauthor_id=30806774)[1](https://pubmed.ncbi.nlm.nih.gov/30806774/#full-view-affiliation-1), [Poliana C Marinello](https://pubmed.ncbi.nlm.nih.gov/?sort=date&term=Marinello+PC&cauthor_id=30806774)[1](https://pubmed.ncbi.nlm.nih.gov/30806774/#full-view-affiliation-1)[2](https://pubmed.ncbi.nlm.nih.gov/30806774/#full-view-affiliation-2), [Fernando H Borges](https://pubmed.ncbi.nlm.nih.gov/?sort=date&term=Borges+FH&cauthor_id=30806774)[2](https://pubmed.ncbi.nlm.nih.gov/30806774/#full-view-affiliation-2), [Diogo F Ribeiro](https://pubmed.ncbi.nlm.nih.gov/?sort=date&term=Ribeiro+DF&cauthor_id=30806774)[1](https://pubmed.ncbi.nlm.nih.gov/30806774/#full-view-affiliation-1), [Patrícia Chimin](https://pubmed.ncbi.nlm.nih.gov/?sort=date&term=Chimin+P&cauthor_id=30806774)[1](https://pubmed.ncbi.nlm.nih.gov/30806774/#full-view-affiliation-1), [Mayra T J Testa](https://pubmed.ncbi.nlm.nih.gov/?sort=date&term=Testa+MTJ&cauthor_id=30806774)[1](https://pubmed.ncbi.nlm.nih.gov/30806774/#full-view-affiliation-1), [Philippe B Guirro](https://pubmed.ncbi.nlm.nih.gov/?sort=date&term=Guirro+PB&cauthor_id=30806774)[1](https://pubmed.ncbi.nlm.nih.gov/30806774/#full-view-affiliation-1), [José A Duarte](https://pubmed.ncbi.nlm.nih.gov/?sort=date&term=Duarte+JA&cauthor_id=30806774)[3](https://pubmed.ncbi.nlm.nih.gov/30806774/#full-view-affiliation-3), [Rubens Cecchini](https://pubmed.ncbi.nlm.nih.gov/?sort=date&term=Cecchini+R&cauthor_id=30806774)[2](https://pubmed.ncbi.nlm.nih.gov/30806774/#full-view-affiliation-2), [Flávia A Guarnier](https://pubmed.ncbi.nlm.nih.gov/?sort=date&term=Guarnier+FA&cauthor_id=30806774)[2](https://pubmed.ncbi.nlm.nih.gov/30806774/#full-view-affiliation-2), [Rafael Deminice](https://pubmed.ncbi.nlm.nih.gov/?sort=date&term=Deminice+R&cauthor_id=30806774)[4](https://pubmed.ncbi.nlm.nih.gov/30806774/#full-view-affiliation-4)

**P)** Os participantes possuem mais de 18 anos de idade?

( ) Sim (X) Não

Os trechos fornecidos deste artigo detalham métodos para análise de expressão gênica e listam referências, mas não descrevem os participantes ou a metodologia de um estudo específico conduzido pelos autores.

**I)** A intervenção utilizada no estudo é creatina? Vale qualquer formato de administração

( ) Sim ( ) Não

**C)** Teve grupo controle?

( ) Sim ( ) Não

**O)** O estudo apresenta valores de marcadores inflamatórios (citocinas e outros marcadores) antes e após as intervenções de creatina?

( ) Sim ( ) Não

**S)** O estudo é um ensaio clínico randomizado e controlado?

( ) Sim ( ) Não

**Artigo 28:** Creatine supplementation impairs airway inflammation in an experimental model of asthma involving P2 × 7 receptor.

[Monique Garcia](https://pubmed.ncbi.nlm.nih.gov/?sort=date&term=Garcia+M&cauthor_id=30888047)[1](https://pubmed.ncbi.nlm.nih.gov/30888047/#full-view-affiliation-1)[2](https://pubmed.ncbi.nlm.nih.gov/30888047/#full-view-affiliation-2), [Alana Santos-Dias](https://pubmed.ncbi.nlm.nih.gov/?sort=date&term=Santos-Dias+A&cauthor_id=30888047)[1](https://pubmed.ncbi.nlm.nih.gov/30888047/#full-view-affiliation-1), [André Luis Lacerda Bachi](https://pubmed.ncbi.nlm.nih.gov/?sort=date&term=Bachi+ALL&cauthor_id=30888047)[1](https://pubmed.ncbi.nlm.nih.gov/30888047/#full-view-affiliation-1)[3](https://pubmed.ncbi.nlm.nih.gov/30888047/#full-view-affiliation-3), [Manoel Carneiro Oliveira-Junior](https://pubmed.ncbi.nlm.nih.gov/?sort=date&term=Oliveira-Junior+MC&cauthor_id=30888047)[1](https://pubmed.ncbi.nlm.nih.gov/30888047/#full-view-affiliation-1), [Adilson Santos Andrade-Souza](https://pubmed.ncbi.nlm.nih.gov/?sort=date&term=Andrade-Souza+AS&cauthor_id=30888047)[1](https://pubmed.ncbi.nlm.nih.gov/30888047/#full-view-affiliation-1), [Sérgio César Ferreira](https://pubmed.ncbi.nlm.nih.gov/?sort=date&term=Ferreira+SC&cauthor_id=30888047)[1](https://pubmed.ncbi.nlm.nih.gov/30888047/#full-view-affiliation-1), [Jefferson Comin Jonco Aquino-Junior](https://pubmed.ncbi.nlm.nih.gov/?sort=date&term=Aquino-Junior+JCJ&cauthor_id=30888047)[1](https://pubmed.ncbi.nlm.nih.gov/30888047/#full-view-affiliation-1), [Francine Maria Almeida](https://pubmed.ncbi.nlm.nih.gov/?sort=date&term=Almeida+FM&cauthor_id=30888047)[1](https://pubmed.ncbi.nlm.nih.gov/30888047/#full-view-affiliation-1), [Nicole Cristine Rigonato-Oliveira](https://pubmed.ncbi.nlm.nih.gov/?sort=date&term=Rigonato-Oliveira+NC&cauthor_id=30888047)[1](https://pubmed.ncbi.nlm.nih.gov/30888047/#full-view-affiliation-1), [Ana Paula Ligeiro Oliveira](https://pubmed.ncbi.nlm.nih.gov/?sort=date&term=Oliveira+APL&cauthor_id=30888047)[4](https://pubmed.ncbi.nlm.nih.gov/30888047/#full-view-affiliation-4), [Luiz Eduardo Baggio Savio](https://pubmed.ncbi.nlm.nih.gov/?sort=date&term=Savio+LEB&cauthor_id=30888047)[5](https://pubmed.ncbi.nlm.nih.gov/30888047/#full-view-affiliation-5), [Robson Coutinho-Silva](https://pubmed.ncbi.nlm.nih.gov/?sort=date&term=Coutinho-Silva+R&cauthor_id=30888047)[5](https://pubmed.ncbi.nlm.nih.gov/30888047/#full-view-affiliation-5), [Tobias Müller](https://pubmed.ncbi.nlm.nih.gov/?sort=date&term=M%C3%BCller+T&cauthor_id=30888047)[6](https://pubmed.ncbi.nlm.nih.gov/30888047/#full-view-affiliation-6), [Marco Idzko](https://pubmed.ncbi.nlm.nih.gov/?sort=date&term=Idzko+M&cauthor_id=30888047)[7](https://pubmed.ncbi.nlm.nih.gov/30888047/#full-view-affiliation-7), [Timo Siepmann](https://pubmed.ncbi.nlm.nih.gov/?sort=date&term=Siepmann+T&cauthor_id=30888047)[2](https://pubmed.ncbi.nlm.nih.gov/30888047/#full-view-affiliation-2)[8](https://pubmed.ncbi.nlm.nih.gov/30888047/#full-view-affiliation-8), [Rodolfo Paula Vieira](https://pubmed.ncbi.nlm.nih.gov/?sort=date&term=Vieira+RP&cauthor_id=30888047)[1](https://pubmed.ncbi.nlm.nih.gov/30888047/#full-view-affiliation-1)[9](https://pubmed.ncbi.nlm.nih.gov/30888047/#full-view-affiliation-9)[10](https://pubmed.ncbi.nlm.nih.gov/30888047/#full-view-affiliation-10)[11](https://pubmed.ncbi.nlm.nih.gov/30888047/#full-view-affiliation-11)

**P)** Os participantes possuem mais de 18 anos de idade?

( ) Sim (X) Não

"The participants of this study were "Forty-eight male BALB/c mice (20–25 g)"."

**I)** A intervenção utilizada no estudo é creatina? Vale qualquer formato de administração

( ) Sim ( ) Não

**C)** Teve grupo controle?

( ) Sim ( ) Não

**O)** O estudo apresenta valores de marcadores inflamatórios (citocinas e outros marcadores) antes e após as intervenções de creatina?

( ) Sim ( ) Não

**S)** O estudo é um ensaio clínico randomizado e controlado?

( ) Sim ( ) Não

**Artigo 29:** High-fat diet suppresses the positive effect of creatine supplementation on skeletal muscle function by reducing protein expression of IGF-PI3K-AKT-mTOR pathway.

[Renato Ferretti](https://pubmed.ncbi.nlm.nih.gov/?sort=date&term=Ferretti+R&cauthor_id=30286093)[1](https://pubmed.ncbi.nlm.nih.gov/30286093/#full-view-affiliation-1), [Eliezer Guimarães Moura](https://pubmed.ncbi.nlm.nih.gov/?sort=date&term=Moura+EG&cauthor_id=30286093)[2](https://pubmed.ncbi.nlm.nih.gov/30286093/#full-view-affiliation-2), [Veridiana Carvalho Dos Santos](https://pubmed.ncbi.nlm.nih.gov/?sort=date&term=Dos+Santos+VC&cauthor_id=30286093)[1](https://pubmed.ncbi.nlm.nih.gov/30286093/#full-view-affiliation-1), [Eduardo José Caldeira](https://pubmed.ncbi.nlm.nih.gov/?sort=date&term=Caldeira+EJ&cauthor_id=30286093)[3](https://pubmed.ncbi.nlm.nih.gov/30286093/#full-view-affiliation-3), [Marcelo Conte](https://pubmed.ncbi.nlm.nih.gov/?sort=date&term=Conte+M&cauthor_id=30286093)[4](https://pubmed.ncbi.nlm.nih.gov/30286093/#full-view-affiliation-4), [Cintia Yuri Matsumura](https://pubmed.ncbi.nlm.nih.gov/?sort=date&term=Matsumura+CY&cauthor_id=30286093)[1](https://pubmed.ncbi.nlm.nih.gov/30286093/#full-view-affiliation-1), [Adriana Pertille](https://pubmed.ncbi.nlm.nih.gov/?sort=date&term=Pertille+A&cauthor_id=30286093)[5](https://pubmed.ncbi.nlm.nih.gov/30286093/#full-view-affiliation-5), [Matias Mosqueira](https://pubmed.ncbi.nlm.nih.gov/?sort=date&term=Mosqueira+M&cauthor_id=30286093)[6](https://pubmed.ncbi.nlm.nih.gov/30286093/#full-view-affiliation-6)

**P)** Os participantes possuem mais de 18 anos de idade?

( ) Sim (X) Não

"The excerpt refers to an 'Animal model for progressive resistance exercise', indicating that the participants are not humans over 18 years old."

**I)** A intervenção utilizada no estudo é creatina? Vale qualquer formato de administração

( ) Sim ( ) Não

**C)** Teve grupo controle?

( ) Sim ( ) Não

**O)** O estudo apresenta valores de marcadores inflamatórios (citocinas e outros marcadores) antes e após as intervenções de creatina?

( ) Sim ( ) Não

**S)** O estudo é um ensaio clínico randomizado e controlado?

( ) Sim ( ) Não

**Artigo 30:** Exploratory studies of the potential anti-cancer effects of creatine.

[P L Campos-Ferraz](https://pubmed.ncbi.nlm.nih.gov/?sort=date&term=Campos-Ferraz+PL&cauthor_id=26872655)[1](https://pubmed.ncbi.nlm.nih.gov/26872655/#full-view-affiliation-1)[2](https://pubmed.ncbi.nlm.nih.gov/26872655/#full-view-affiliation-2), [B Gualano](https://pubmed.ncbi.nlm.nih.gov/?sort=date&term=Gualano+B&cauthor_id=26872655)[3](https://pubmed.ncbi.nlm.nih.gov/26872655/#full-view-affiliation-3), [W das Neves](https://pubmed.ncbi.nlm.nih.gov/?sort=date&term=das+Neves+W&cauthor_id=26872655)[3](https://pubmed.ncbi.nlm.nih.gov/26872655/#full-view-affiliation-3), [I T Andrade](https://pubmed.ncbi.nlm.nih.gov/?sort=date&term=Andrade+IT&cauthor_id=26872655)[3](https://pubmed.ncbi.nlm.nih.gov/26872655/#full-view-affiliation-3)[4](https://pubmed.ncbi.nlm.nih.gov/26872655/#full-view-affiliation-4), [I Hangai](https://pubmed.ncbi.nlm.nih.gov/?sort=date&term=Hangai+I&cauthor_id=26872655)[3](https://pubmed.ncbi.nlm.nih.gov/26872655/#full-view-affiliation-3)[5](https://pubmed.ncbi.nlm.nih.gov/26872655/#full-view-affiliation-5), [R T S Pereira](https://pubmed.ncbi.nlm.nih.gov/?sort=date&term=Pereira+RT&cauthor_id=26872655)[3](https://pubmed.ncbi.nlm.nih.gov/26872655/#full-view-affiliation-3), [R N Bezerra](https://pubmed.ncbi.nlm.nih.gov/?sort=date&term=Bezerra+RN&cauthor_id=26872655)[4](https://pubmed.ncbi.nlm.nih.gov/26872655/#full-view-affiliation-4), [R Deminice](https://pubmed.ncbi.nlm.nih.gov/?sort=date&term=Deminice+R&cauthor_id=26872655)[6](https://pubmed.ncbi.nlm.nih.gov/26872655/#full-view-affiliation-6), [M Seelaender](https://pubmed.ncbi.nlm.nih.gov/?sort=date&term=Seelaender+M&cauthor_id=26872655)[7](https://pubmed.ncbi.nlm.nih.gov/26872655/#full-view-affiliation-7)[8](https://pubmed.ncbi.nlm.nih.gov/26872655/#full-view-affiliation-8), [A H Lancha](https://pubmed.ncbi.nlm.nih.gov/?sort=date&term=Lancha+AH&cauthor_id=26872655)[3](https://pubmed.ncbi.nlm.nih.gov/26872655/#full-view-affiliation-3)

**P)** Os participantes possuem mais de 18 anos de idade?

( ) Sim (X) Não

"The participants in this study were "Fifty 8-week-old male Wistar rats"."

**I)** A intervenção utilizada no estudo é creatina? Vale qualquer formato de administração

( ) Sim ( ) Não

**C)** Teve grupo controle?

( ) Sim ( ) Não

**O)** O estudo apresenta valores de marcadores inflamatórios (citocinas e outros marcadores) antes e após as intervenções de creatina?

( ) Sim ( ) Não

**S)** O estudo é um ensaio clínico randomizado e controlado?

( ) Sim ( ) Não

**Artigo 31:** Creatine supplementation attenuates pulmonary and systemic effects of lung ischemia and reperfusion injury.

[Francine Maria Almeida](https://pubmed.ncbi.nlm.nih.gov/?sort=date&term=Almeida+FM&cauthor_id=26215332)[1](https://pubmed.ncbi.nlm.nih.gov/26215332/#full-view-affiliation-1), [Manoel Carneiro Oliveira-Junior](https://pubmed.ncbi.nlm.nih.gov/?sort=date&term=Oliveira-Junior+MC&cauthor_id=26215332)[2](https://pubmed.ncbi.nlm.nih.gov/26215332/#full-view-affiliation-2), [Renato Aparecido Souza](https://pubmed.ncbi.nlm.nih.gov/?sort=date&term=Souza+RA&cauthor_id=26215332)[3](https://pubmed.ncbi.nlm.nih.gov/26215332/#full-view-affiliation-3), [Ricardo Costa Petroni](https://pubmed.ncbi.nlm.nih.gov/?sort=date&term=Petroni+RC&cauthor_id=26215332)[4](https://pubmed.ncbi.nlm.nih.gov/26215332/#full-view-affiliation-4), [Sonia Fatima Soto](https://pubmed.ncbi.nlm.nih.gov/?sort=date&term=Soto+SF&cauthor_id=26215332)[1](https://pubmed.ncbi.nlm.nih.gov/26215332/#full-view-affiliation-1), [Francisco Garcia Soriano](https://pubmed.ncbi.nlm.nih.gov/?sort=date&term=Soriano+FG&cauthor_id=26215332)[4](https://pubmed.ncbi.nlm.nih.gov/26215332/#full-view-affiliation-4), [Paulo Tarso Camillo de Carvalho](https://pubmed.ncbi.nlm.nih.gov/?sort=date&term=Carvalho+PT&cauthor_id=26215332)[2](https://pubmed.ncbi.nlm.nih.gov/26215332/#full-view-affiliation-2), [Regiane Albertini](https://pubmed.ncbi.nlm.nih.gov/?sort=date&term=Albertini+R&cauthor_id=26215332)[2](https://pubmed.ncbi.nlm.nih.gov/26215332/#full-view-affiliation-2), [Nilsa Regina Damaceno-Rodrigues](https://pubmed.ncbi.nlm.nih.gov/?sort=date&term=Damaceno-Rodrigues+NR&cauthor_id=26215332)[5](https://pubmed.ncbi.nlm.nih.gov/26215332/#full-view-affiliation-5), [Fernanda Degobbi Tenorio Quirino Santos Lopes](https://pubmed.ncbi.nlm.nih.gov/?sort=date&term=Lopes+FD&cauthor_id=26215332)[1](https://pubmed.ncbi.nlm.nih.gov/26215332/#full-view-affiliation-1), [Hugo Caire Castro-Faria-Neto](https://pubmed.ncbi.nlm.nih.gov/?sort=date&term=Castro-Faria-Neto+HC&cauthor_id=26215332)[6](https://pubmed.ncbi.nlm.nih.gov/26215332/#full-view-affiliation-6), [Milton Arruda Martins](https://pubmed.ncbi.nlm.nih.gov/?sort=date&term=Martins+MA&cauthor_id=26215332)[1](https://pubmed.ncbi.nlm.nih.gov/26215332/#full-view-affiliation-1), [Marisa Dolhnikoff](https://pubmed.ncbi.nlm.nih.gov/?sort=date&term=Dolhnikoff+M&cauthor_id=26215332)[7](https://pubmed.ncbi.nlm.nih.gov/26215332/#full-view-affiliation-7), [Rogerio Pazetti](https://pubmed.ncbi.nlm.nih.gov/?sort=date&term=Pazetti+R&cauthor_id=26215332)[8](https://pubmed.ncbi.nlm.nih.gov/26215332/#full-view-affiliation-8), [Rodolfo Paula Vieira](https://pubmed.ncbi.nlm.nih.gov/?sort=date&term=Vieira+RP&cauthor_id=26215332)[9](https://pubmed.ncbi.nlm.nih.gov/26215332/#full-view-affiliation-9)

**P)** Os participantes possuem mais de 18 anos de idade?

( ) Sim (X) Não

"The study's methodologies, such as 'The left lungs were homogenized' and the general context of authors' previous work (animal models), indicate that the participants are not humans over 18 years old."

**I)** A intervenção utilizada no estudo é creatina? Vale qualquer formato de administração

( ) Sim ( ) Não

**C)** Teve grupo controle?

( ) Sim ( ) Não

**O)** O estudo apresenta valores de marcadores inflamatórios (citocinas e outros marcadores) antes e após as intervenções de creatina?

( ) Sim ( ) Não

**S)** O estudo é um ensaio clínico randomizado e controlado?

( ) Sim ( ) Não

**Artigo 32: Efficacy and safety of creatine supplementation in juvenile dermatomyositis: A**randomized**, double-blind,**placebo**-controlled**crossovertrial**.**

Solis, Marina Yazigi, Hayashi, Ana Paula, Artioli, Guilherme Giannini, Roschel, Hamilton, Sapienza, Marcelo Tatit, Otaduy, Maria Concepción, De Sã Pinto, Ana Lucia, Silva, Clovis Artur, Sallum, Adriana Maluf Elias, Pereira, Rosa Maria R, Gualano, Bruno

**P)** Os participantes possuem mais de 18 anos de idade?

( ) Sim ( ) Não

**I)** A intervenção utilizada no estudo é creatina? Vale qualquer formato de administração

( ) Sim ( ) Não

**C)** Teve grupo controle?

( ) Sim ( ) Não

**O)** O estudo apresenta valores de marcadores inflamatórios (citocinas e outros marcadores) antes e após as intervenções de creatina?

( ) Sim ( ) Não

**S)** O estudo é um ensaio clínico randomizado e controlado?

( ) Sim ( ) Não

**Artigo 33:** Effects of creatine supplementation on oxidative stress and inflammatory markers after repeated-sprint exercise in humans.

[Rafael Deminice](https://pubmed.ncbi.nlm.nih.gov/?sort=date&term=Deminice+R&cauthor_id=23800565)[1](https://pubmed.ncbi.nlm.nih.gov/23800565/#full-view-affiliation-1), [Flávia Troncon Rosa](https://pubmed.ncbi.nlm.nih.gov/?sort=date&term=Rosa+FT&cauthor_id=23800565), [Gabriel Silveira Franco](https://pubmed.ncbi.nlm.nih.gov/?sort=date&term=Franco+GS&cauthor_id=23800565), [Alceu Afonso Jordao](https://pubmed.ncbi.nlm.nih.gov/?sort=date&term=Jordao+AA&cauthor_id=23800565), [Ellen Cristini de Freitas](https://pubmed.ncbi.nlm.nih.gov/?sort=date&term=de+Freitas+EC&cauthor_id=23800565)

**P)** Os participantes possuem mais de 18 anos de idade?

( ) Sim (X) Não

"The participants were 25 healthy and well-trained men from an "Under-20 y soccer team", with a mean age of "17.4 1.2" years and "17.1 1.4" years for the respective groups."

**I)** A intervenção utilizada no estudo é creatina? Vale qualquer formato de administração

( ) Sim ( ) Não

**C)** Teve grupo controle?

( ) Sim ( ) Não

**O)** O estudo apresenta valores de marcadores inflamatórios (citocinas e outros marcadores) antes e após as intervenções de creatina?

( ) Sim ( ) Não

**S)** O estudo é um ensaio clínico randomizado e controlado?

( ) Sim ( ) Não

**Artigo 34: Creatine supplementation does not decrease oxidative stress and inflammation in skeletal muscle after eccentric exercise.**

Silva, Luciano A, Tromm, Camila B, Da Rosa, Guilherme, Bom, Karoliny, Luciano, Thais F, Tuon, Talita, De Souza, Cláudio T, Pinho, Ricardo A

**P)** Os participantes possuem mais de 18 anos de idade?

( ) Sim ( ) Não

**I)** A intervenção utilizada no estudo é creatina? Vale qualquer formato de administração

( ) Sim ( ) Não

**C)** Teve grupo controle?

( ) Sim ( ) Não

**O)** O estudo apresenta valores de marcadores inflamatórios (citocinas e outros marcadores) antes e após as intervenções de creatina?

( ) Sim ( ) Não

**S)** O estudo é um ensaio clínico randomizado e controlado?

( ) Sim ( ) Não

**Artigo 35:** Creatine activates airway epithelium in asthma.

[S C Ferreira](https://pubmed.ncbi.nlm.nih.gov/?sort=date&term=Ferreira+SC&cauthor_id=21072743)[1](https://pubmed.ncbi.nlm.nih.gov/21072743/#full-view-affiliation-1), [A C Toledo](https://pubmed.ncbi.nlm.nih.gov/?sort=date&term=Toledo+AC&cauthor_id=21072743), [M Hage](https://pubmed.ncbi.nlm.nih.gov/?sort=date&term=Hage+M&cauthor_id=21072743), [A B G Santos](https://pubmed.ncbi.nlm.nih.gov/?sort=date&term=Santos+AB&cauthor_id=21072743), [M C R Medeiros](https://pubmed.ncbi.nlm.nih.gov/?sort=date&term=Medeiros+MC&cauthor_id=21072743), [M A Martins](https://pubmed.ncbi.nlm.nih.gov/?sort=date&term=Martins+MA&cauthor_id=21072743), [C R F Carvalho](https://pubmed.ncbi.nlm.nih.gov/?sort=date&term=Carvalho+CR&cauthor_id=21072743), [M Dolhnikoff](https://pubmed.ncbi.nlm.nih.gov/?sort=date&term=Dolhnikoff+M&cauthor_id=21072743), [R P Vieira](https://pubmed.ncbi.nlm.nih.gov/?sort=date&term=Vieira+RP&cauthor_id=21072743)

**P)** Os participantes possuem mais de 18 anos de idade?

( ) Sim (X) Não

"The study participants were "32 male BALB / c mice (20 – 25 g)"."

**I)** A intervenção utilizada no estudo é creatina? Vale qualquer formato de administração

( ) Sim ( ) Não

**C)** Teve grupo controle?

( ) Sim ( ) Não

**O)** O estudo apresenta valores de marcadores inflamatórios (citocinas e outros marcadores) antes e após as intervenções de creatina?

( ) Sim ( ) Não

**S)** O estudo é um ensaio clínico randomizado e controlado?

( ) Sim ( ) Não

**Artigo 36:** Can Creatine Supplementation Interfere with Muscle Strength and Fatigue in Brazilian National Level Paralympic Powerlifting?

[Carlos Rodrigo Soares Freitas Sampaio](https://pubmed.ncbi.nlm.nih.gov/?sort=date&term=Soares+Freitas+Sampaio+CR&cauthor_id=32824920)[1](https://pubmed.ncbi.nlm.nih.gov/32824920/#full-view-affiliation-1), [Felipe J Aidar](https://pubmed.ncbi.nlm.nih.gov/?sort=date&term=Aidar+FJ&cauthor_id=32824920)[1](https://pubmed.ncbi.nlm.nih.gov/32824920/#full-view-affiliation-1)[2](https://pubmed.ncbi.nlm.nih.gov/32824920/#full-view-affiliation-2)[3](https://pubmed.ncbi.nlm.nih.gov/32824920/#full-view-affiliation-3)[4](https://pubmed.ncbi.nlm.nih.gov/32824920/#full-view-affiliation-4), [Alexandre R P Ferreira](https://pubmed.ncbi.nlm.nih.gov/?sort=date&term=Ferreira+ARP&cauthor_id=32824920)[5](https://pubmed.ncbi.nlm.nih.gov/32824920/#full-view-affiliation-5), [Jymmys Lopes Dos Santos](https://pubmed.ncbi.nlm.nih.gov/?sort=date&term=Santos+JLD&cauthor_id=32824920)[6](https://pubmed.ncbi.nlm.nih.gov/32824920/#full-view-affiliation-6), [Anderson Carlos Marçal](https://pubmed.ncbi.nlm.nih.gov/?sort=date&term=Mar%C3%A7al+AC&cauthor_id=32824920)[1](https://pubmed.ncbi.nlm.nih.gov/32824920/#full-view-affiliation-1)[3](https://pubmed.ncbi.nlm.nih.gov/32824920/#full-view-affiliation-3), [Dihogo Gama de Matos](https://pubmed.ncbi.nlm.nih.gov/?sort=date&term=Matos+DG&cauthor_id=32824920)[1](https://pubmed.ncbi.nlm.nih.gov/32824920/#full-view-affiliation-1), [Raphael Fabrício de Souza](https://pubmed.ncbi.nlm.nih.gov/?sort=date&term=Souza+RF&cauthor_id=32824920)[1](https://pubmed.ncbi.nlm.nih.gov/32824920/#full-view-affiliation-1)[2](https://pubmed.ncbi.nlm.nih.gov/32824920/#full-view-affiliation-2), [Osvaldo Costa Moreira](https://pubmed.ncbi.nlm.nih.gov/?sort=date&term=Moreira+OC&cauthor_id=32824920)[7](https://pubmed.ncbi.nlm.nih.gov/32824920/#full-view-affiliation-7), [Ialuska Guerra](https://pubmed.ncbi.nlm.nih.gov/?sort=date&term=Guerra+I&cauthor_id=32824920)[8](https://pubmed.ncbi.nlm.nih.gov/32824920/#full-view-affiliation-8), [José Fernandes Filho](https://pubmed.ncbi.nlm.nih.gov/?sort=date&term=Fernandes+Filho+J&cauthor_id=32824920)[9](https://pubmed.ncbi.nlm.nih.gov/32824920/#full-view-affiliation-9), [Lucas Soares Marcucci-Barbosa](https://pubmed.ncbi.nlm.nih.gov/?sort=date&term=Marcucci-Barbosa+LS&cauthor_id=32824920)[10](https://pubmed.ncbi.nlm.nih.gov/32824920/#full-view-affiliation-10), [Albená Nunes-Silva](https://pubmed.ncbi.nlm.nih.gov/?sort=date&term=Nunes-Silva+A&cauthor_id=32824920)[10](https://pubmed.ncbi.nlm.nih.gov/32824920/#full-view-affiliation-10), [Paulo Francisco de Almeida-Neto](https://pubmed.ncbi.nlm.nih.gov/?sort=date&term=Almeida-Neto+PF&cauthor_id=32824920)[11](https://pubmed.ncbi.nlm.nih.gov/32824920/#full-view-affiliation-11), [Breno Guilherme Araújo Tinoco Cabral](https://pubmed.ncbi.nlm.nih.gov/?sort=date&term=Cabral+BGAT&cauthor_id=32824920)[11](https://pubmed.ncbi.nlm.nih.gov/32824920/#full-view-affiliation-11), [Victor Machado Reis](https://pubmed.ncbi.nlm.nih.gov/?sort=date&term=Reis+VM&cauthor_id=32824920)[12](https://pubmed.ncbi.nlm.nih.gov/32824920/#full-view-affiliation-12)

**P)** Os participantes possuem mais de 18 anos de idade?

(X) Sim ( ) Não

"The study involved "Eight Paralympic powerlifting athletes" with an age of "25.40 ± 3.30 years"

**I)** A intervenção utilizada no estudo é creatina? Vale qualquer formato de administração

(X) Sim ( ) Não

"The study's aim was to analyze "the effect of creatine (Cr) supplementation" and involved subjects receiving "creatine supplementation for 7 days"

**C)** Teve grupo controle?

(X) Sim ( ) Não

"The study was performed in a "single-blind manner, with subjects conducting the experiments first with placebo supplementation", serving as a control condition."

**O)** O estudo apresenta valores de marcadores inflamatórios (citocinas e outros marcadores) antes e após as intervenções de creatina?

( ) Sim (X) Não

"The outcomes measured in this study included "muscle strength, fatigue index (FI), peak torque (PT), force (kgf), force (N), rate of force development (RFD), and time to maximum isometric force". No inflammatory markers were mentioned."

**S)** O estudo é um ensaio clínico randomizado e controlado?

( ) Sim ( ) Não

**Artigo 37:** **POST-OLYMPIA RECUPERATION.**

Autores???

**P)** Os participantes possuem mais de 18 anos de idade?

( ) Sim ( ) Não

**I)** A intervenção utilizada no estudo é creatina? Vale qualquer formato de administração

( ) Sim ( ) Não

**C)** Teve grupo controle?

( ) Sim ( ) Não

**O)** O estudo apresenta valores de marcadores inflamatórios (citocinas e outros marcadores) antes e após as intervenções de creatina?

( ) Sim ( ) Não

**S)** O estudo é um ensaio clínico randomizado e controlado?

( ) Sim ( ) Não

**Artigo 38:** Creatine kinase in ischemic and inflammatory disorders.

[David Kitzenberg](https://pubmed.ncbi.nlm.nih.gov/?term=%22Kitzenberg%20D%22%5BAuthor%5D) 1,2, [Sean P Colgan](https://pubmed.ncbi.nlm.nih.gov/?term=%22Colgan%20SP%22%5BAuthor%5D) 1,2, [Louise E Glover](https://pubmed.ncbi.nlm.nih.gov/?term=%22Glover%20LE%22%5BAuthor%5D) 1,2,✉

**P)** Os participantes possuem mais de 18 anos de idade?

( ) Sim (X) Não

Este é um artigo de revisão ("writing of this review paper") e não descreve um estudo primário com participantes conduzido pelos autores.

**I)** A intervenção utilizada no estudo é creatina? Vale qualquer formato de administração

( ) Sim ( ) Não

**C)** Teve grupo controle?

( ) Sim ( ) Não

**O)** O estudo apresenta valores de marcadores inflamatórios (citocinas e outros marcadores) antes e após as intervenções de creatina?

( ) Sim ( ) Não

**S)** O estudo é um ensaio clínico randomizado e controlado?

( ) Sim ( ) Não

**Artigo 39:** Creatine Supplementation for Patients with Inflammatory Bowel Diseases: A Scientific Rationale for a Clinical Trial

[Theo Wallimann](https://pubmed.ncbi.nlm.nih.gov/?sort=date&term=Wallimann+T&cauthor_id=33922654)[1](https://pubmed.ncbi.nlm.nih.gov/33922654/#full-view-affiliation-1), [Caroline H T Hall](https://pubmed.ncbi.nlm.nih.gov/?sort=date&term=Hall+CHT&cauthor_id=33922654)[2](https://pubmed.ncbi.nlm.nih.gov/33922654/#full-view-affiliation-2), [Sean P Colgan](https://pubmed.ncbi.nlm.nih.gov/?sort=date&term=Colgan+SP&cauthor_id=33922654)[3](https://pubmed.ncbi.nlm.nih.gov/33922654/#full-view-affiliation-3), [Louise E Glover](https://pubmed.ncbi.nlm.nih.gov/?sort=date&term=Glover+LE&cauthor_id=33922654)[4](https://pubmed.ncbi.nlm.nih.gov/33922654/#full-view-affiliation-4)

**P)** Os participantes possuem mais de 18 anos de idade?

(X) Sim ( ) Não

"The ongoing pilot clinical trial described involves "12 patients, aged 18–70, with mild to moderate ulcerative colitis"."

**I)** A intervenção utilizada no estudo é creatina? Vale qualquer formato de administração

(X) Sim ( ) Não

"The trial "incorporates the use of oral Cr at a dose of 2 × 7 g per day"."

**C)** Teve grupo controle?

( ) Sim (X) Não

"For the "current pilot clinical trial" involving 12 patients, the provided excerpts do not explicitly state the inclusion of a control (placebo) group; a control group is mentioned only for a "proposed larger, more long-term Cr supplementation study"."

**O)** O estudo apresenta valores de marcadores inflamatórios (citocinas e outros marcadores) antes e após as intervenções de creatina?

( ) Sim ( ) Não

**S)** O estudo é um ensaio clínico randomizado e controlado?

( ) Sim ( ) Não

**Artigo 40:** Nutrition and Polymyositis and Dermatomyositis

Ingela Loell and Ingrid Lundberg

**P)** Os participantes possuem mais de 18 anos de idade?

( ) Sim (X) Não

Este é um capítulo de revisão ("solely a review of the field of research") e não descreve um estudo primário com participantes conduzido pelos autores.

**I)** A intervenção utilizada no estudo é creatina? Vale qualquer formato de administração

( ) Sim ( ) Não

**C)** Teve grupo controle?

( ) Sim ( ) Não

**O)** O estudo apresenta valores de marcadores inflamatórios (citocinas e outros marcadores) antes e após as intervenções de creatina?

( ) Sim ( ) Não

**S)** O estudo é um ensaio clínico randomizado e controlado?

( ) Sim ( ) Não

**Artigo 41:** Anti-Inflammatory and Anti-Catabolic Effects of Creatine Supplementation: A Brief Review

[Dean M Cordingley](https://pubmed.ncbi.nlm.nih.gov/?sort=date&term=Cordingley+DM&cauthor_id=35276903)[1](https://pubmed.ncbi.nlm.nih.gov/35276903/#full-view-affiliation-1)[2](https://pubmed.ncbi.nlm.nih.gov/35276903/#full-view-affiliation-2), [Stephen M Cornish](https://pubmed.ncbi.nlm.nih.gov/?sort=date&term=Cornish+SM&cauthor_id=35276903)[1](https://pubmed.ncbi.nlm.nih.gov/35276903/#full-view-affiliation-1)[3](https://pubmed.ncbi.nlm.nih.gov/35276903/#full-view-affiliation-3)[4](https://pubmed.ncbi.nlm.nih.gov/35276903/#full-view-affiliation-4), [Darren G Candow](https://pubmed.ncbi.nlm.nih.gov/?sort=date&term=Candow+DG&cauthor_id=35276903)[5](https://pubmed.ncbi.nlm.nih.gov/35276903/#full-view-affiliation-5)

**P)** Os participantes possuem mais de 18 anos de idade?

( ) Sim (X) Não

Este é um artigo de revisão e destaca descobertas de outros estudos ("studies have evaluated the efficacy of a combined nutritional supplement beverage intake"), mas não descreve um estudo primário com participantes conduzido pelos autores.

**I)** A intervenção utilizada no estudo é creatina? Vale qualquer formato de administração

( ) Sim ( ) Não

**C)** Teve grupo controle?

( ) Sim ( ) Não

**O)** O estudo apresenta valores de marcadores inflamatórios (citocinas e outros marcadores) antes e após as intervenções de creatina?

( ) Sim ( ) Não

**S)** O estudo é um ensaio clínico randomizado e controlado?

( ) Sim ( ) Não

**Artigo 42:** **Creatine supplementation attenuates inflammation in collateral lung after left lung transplantation**

de Almeida, FM, Battochio, AS, Napoli, JP, Alves, KA, Balbin, GS, Oliveira, M, Moriya, HT, Pego-Fernandes, P, Vieira, RP, Pazetti, R

**P)** Os participantes possuem mais de 18 anos de idade?

( ) Sim ( ) Não

**I)** A intervenção utilizada no estudo é creatina? Vale qualquer formato de administração

( ) Sim ( ) Não

**C)** Teve grupo controle?

( ) Sim ( ) Não

**O)** O estudo apresenta valores de marcadores inflamatórios (citocinas e outros marcadores) antes e após as intervenções de creatina?

( ) Sim ( ) Não

**S)** O estudo é um ensaio clínico randomizado e controlado?

( ) Sim ( ) Não

**Artigo 43:** Creatine supplementation attenuates pathogen-induced airway inflammation in pediatric primary airway epithelial cells

**P)** Os participantes possuem mais de 18 anos de idade?

( ) Sim (X) Não

"The human primary airway epithelial cells used in the study were obtained from pediatric donors with mean ages of "3.7 ± 2.9 years" and "2.8 ± 0.6 years", indicating they were not individuals over 18 years of age."

**I)** A intervenção utilizada no estudo é creatina? Vale qualquer formato de administração

( ) Sim ( ) Não

**C)** Teve grupo controle?

( ) Sim ( ) Não

**O)** O estudo apresenta valores de marcadores inflamatórios (citocinas e outros marcadores) antes e após as intervenções de creatina?

( ) Sim ( ) Não

**S)** O estudo é um ensaio clínico randomizado e controlado?

( ) Sim ( ) Não

**Artigo 44:** Effectiveness of Creatine Supplementation on Aging Muscle and Bone: Focus on Falls Prevention and Inflammation

[Darren G Candow](https://pubmed.ncbi.nlm.nih.gov/?term=%22Candow%20DG%22%5BAuthor%5D) 1,*, [Scott C Forbes](https://pubmed.ncbi.nlm.nih.gov/?term=%22Forbes%20SC%22%5BAuthor%5D) 2, [Philip D Chilibeck](https://pubmed.ncbi.nlm.nih.gov/?term=%22Chilibeck%20PD%22%5BAuthor%5D) 3, [Stephen M Cornish](https://pubmed.ncbi.nlm.nih.gov/?term=%22Cornish%20SM%22%5BAuthor%5D) 4, [Jose Antonio](https://pubmed.ncbi.nlm.nih.gov/?term=%22Antonio%20J%22%5BAuthor%5D) 5, [Richard B Kreider](https://pubmed.ncbi.nlm.nih.gov/?term=%22Kreider%20RB%22%5BAuthor%5D)

**P)** Os participantes possuem mais de 18 anos de idade?

( ) Sim (X) Não

Este é um artigo de revisão ("Direct evaluation of the safety of creatine supplementation in aging adults is limited") e sintetiza descobertas de outros estudos, mas não descreve um estudo primário com participantes conduzido pelos autores

**I)** A intervenção utilizada no estudo é creatina? Vale qualquer formato de administração

( ) Sim ( ) Não

**C)** Teve grupo controle?

( ) Sim ( ) Não

**O)** O estudo apresenta valores de marcadores inflamatórios (citocinas e outros marcadores) antes e após as intervenções de creatina?

( ) Sim ( ) Não

**S)** O estudo é um ensaio clínico randomizado e controlado?

( ) Sim ( ) Não

**Artigo 45:** **Creatine Supplementation Augments Chronic Allergic Airway Inflammation and Remodelling: from**Mice**to Human Evidence**

Garcia, M, Santos-Dias, A, Andrade-Souza, AS, Oliveira, MC, Cesar-Ferreira, S, Almeida, FM, Rigonato-Oliveira, NC, Oliveira, APL, Siepmann, T, Idzko, M, Müller, T, Vieira, RP

**P)** Os participantes possuem mais de 18 anos de idade?

( ) Sim ( ) Não

**I)** A intervenção utilizada no estudo é creatina? Vale qualquer formato de administração

( ) Sim ( ) Não

**C)** Teve grupo controle?

( ) Sim ( ) Não

**O)** O estudo apresenta valores de marcadores inflamatórios (citocinas e outros marcadores) antes e após as intervenções de creatina?

( ) Sim ( ) Não

**S)** O estudo é um ensaio clínico randomizado e controlado?

( ) Sim ( ) Não

**Artigo 46:** Creatine supplementation enhances immunological function of neutrophils by increasing cellular adenosine triphosphate

[Suguru SAITO](https://pubmed.ncbi.nlm.nih.gov/?term=%22SAITO%20S%22%5BAuthor%5D) 1,2,3,*, [Duo-Yao CAO](https://pubmed.ncbi.nlm.nih.gov/?term=%22CAO%20DY%22%5BAuthor%5D) 4,5, [Alato OKUNO](https://pubmed.ncbi.nlm.nih.gov/?term=%22OKUNO%20A%22%5BAuthor%5D) 6, [Xiaomo LI](https://pubmed.ncbi.nlm.nih.gov/?term=%22LI%20X%22%5BAuthor%5D) 7, [Zhenzi PENG](https://pubmed.ncbi.nlm.nih.gov/?term=%22PENG%20Z%22%5BAuthor%5D) 8, [Musin KELEL](https://pubmed.ncbi.nlm.nih.gov/?term=%22KELEL%20M%22%5BAuthor%5D) 9, [Noriko M TSUJI](https://pubmed.ncbi.nlm.nih.gov/?term=%22TSUJI%20NM%22%5BAuthor%5D)

**P)** Os participantes possuem mais de 18 anos de idade?

( ) Sim (X) Não

The participants in this study were "C57BL/6 J mice" aged "8- and 12-week-old"."

**I)** A intervenção utilizada no estudo é creatina? Vale qualquer formato de administração

( ) Sim ( ) Não

**C)** Teve grupo controle?

( ) Sim ( ) Não

**O)** O estudo apresenta valores de marcadores inflamatórios (citocinas e outros marcadores) antes e após as intervenções de creatina?

( ) Sim ( ) Não

**S)** O estudo é um ensaio clínico randomizado e controlado?

( ) Sim ( ) Não

**Artigo 47:** **Creatine supplementation does not promote additional effects on inflammation and insulin resistance in older adults: A pilot**randomized**, double-blind,**placebo**-**controlled trial

Oliveira, CLP, Antunes, BDM, Gomes, AC, Lira, FS, Pimentel, GD, Boulé, NG, Mota, JF

**P)** Os participantes possuem mais de 18 anos de idade?

(X) Sim ( ) Não

"Men and women **aged 60 and 80 years** were recruited."

**I)** A intervenção utilizada no estudo é creatina? Vale qualquer formato de administração

(X) Sim ( ) Não

"This study aimed to investigate the effects of **CR supplementation** combined with RT on markers of inflam-mation and insulin resistance in community-dwelling older adults."

"Participants were asked to consume **CR (5 g/day) or maltodextrin (5 g/day)**, while engaged in a 12-week supervised RT program."

"On training days, participants consumed the supplement immediately after the training sessions dissolved in a beverage comprised of 100 g of lemon-flavored maltodextrin. On non-training days, participants consumed the supplement immediately after lunch dissolved in **one glass of liquid of their preference**."

**C)** Teve grupo controle?

(X) Sim ( ) Não

"pilot **randomized, double-blind, placebo-controlled trial**, participants were allocated to one of the following groups: 1) Creatine supplementation and resistance training (CR þ RT, n ¼ 13); 2) **Placebo and resistance training (PL þ RT, n ¼ 14)**."

**O)** O estudo apresenta valores de marcadores inflamatórios (citocinas e outros marcadores) antes e após as intervenções de creatina?

(X) Sim ( ) Não

"At baseline and at week 12, blood samples were collected for glucose, insulin, adiponectin, leptin, **interleukin 6, interleukin 10, monocyte chemo-attractant protein-1 and C-reactive protein analysis**."

"**All biochemical analyses were done at baseline and after 12 weeks of intervention.**"

**S)** O estudo é um ensaio clínico randomizado e controlado?

(X) Sim ( ) Não

"**pilot randomized, double-blind, placebo-controlled trial**"

"Experimental design, participants’ information and intervention protocol"

"This study was a part of a **randomized, double-blind, placebo-controlled, parallel-group clinical trial**, registered at ensaiosclinicos.gov.br (RBR-2shfhj) and approved by the Human Research Ethics Committee of the Federal University of Goias (840.317)."

"**randomly assigned** following a stratified randomization based on sex"

**Artigo 48:** The effect of creatine supplementation upon inflammatory and muscle soreness markers after a 30km race

[R V T Santos](https://pubmed.ncbi.nlm.nih.gov/?sort=date&term=Santos+RV&cauthor_id=15306159)[1](https://pubmed.ncbi.nlm.nih.gov/15306159/#full-view-affiliation-1), [R A Bassit](https://pubmed.ncbi.nlm.nih.gov/?sort=date&term=Bassit+RA&cauthor_id=15306159), [E C Caperuto](https://pubmed.ncbi.nlm.nih.gov/?sort=date&term=Caperuto+EC&cauthor_id=15306159), [L F B P Costa Rosa](https://pubmed.ncbi.nlm.nih.gov/?sort=date&term=Costa+Rosa+LF&cauthor_id=15306159)

**P)** Os participantes possuem mais de 18 anos de idade?

(X) Sim ( ) Não

“The athletes, whose mean age was of 25.5 F 3.2 years, ranging from 21.4 to 30.1 years”

**I)** A intervenção utilizada no estudo é creatina? Vale qualquer formato de administração

(X) Sim ( ) Não

“were allowed to drink and eat normally, and received creatine or placebo during the 5 days that preceded the tests”

**C)** Teve grupo controle?

(X) Sim ( ) Não

“After the test, athletes from the control group presented an increase in plasma”

**O)** O estudo apresenta valores de marcadores inflamatórios (citocinas e outros marcadores) antes e após as intervenções de creatina?

(X) Sim ( ) Não

“We evaluated the effect of creatine supplementation upon cell injury and inflammatory markers after a 30km run.”

**S)** O estudo é um ensaio clínico randomizado e controlado?

( ) Sim (X) Não

“After signing an Informed Consent form, thirty four male athletes received, for 5 days, 20g of creatine monohydrate divided in 4 doses of 5g, with 60g of carbohydrate (maltodextrine, n = 18) or the same amount of carbohydrate (n = 16)—placebo group, and ran 30km in a double-blind trial.”

**Artigo 49:** **Exercise Reduces Effects of Creatine on Lung**

Vieira, RP, Duarte, AC, Santos, ABG, Medeiros, MCR, Mauad, T, Martins, MA, Carvalho, CRF, Dolhnikoff, M

**P)** Os participantes possuem mais de 18 anos de idade?

( ) Sim ( ) Não

**I)** A intervenção utilizada no estudo é creatina? Vale qualquer formato de administração

( ) Sim ( ) Não

**C)** Teve grupo controle?

( ) Sim ( ) Não

**O)** O estudo apresenta valores de marcadores inflamatórios (citocinas e outros marcadores) antes e após as intervenções de creatina?

( ) Sim ( ) Não

**S)** O estudo é um ensaio clínico randomizado e controlado?

( ) Sim ( ) Não

**Artigo 50:** The Evolving Applications of Creatine Supplementation: Could Creatine Improve Vascular Health?

[Holly Clarke](https://pubmed.ncbi.nlm.nih.gov/?sort=date&term=Clarke+H&cauthor_id=32947909)[1](https://pubmed.ncbi.nlm.nih.gov/32947909/#full-view-affiliation-1), [Do-Houn Kim](https://pubmed.ncbi.nlm.nih.gov/?sort=date&term=Kim+DH&cauthor_id=32947909)[1](https://pubmed.ncbi.nlm.nih.gov/32947909/#full-view-affiliation-1), [Cesar A Meza](https://pubmed.ncbi.nlm.nih.gov/?sort=date&term=Meza+CA&cauthor_id=32947909)[1](https://pubmed.ncbi.nlm.nih.gov/32947909/#full-view-affiliation-1), [Michael J Ormsbee](https://pubmed.ncbi.nlm.nih.gov/?sort=date&term=Ormsbee+MJ&cauthor_id=32947909)[1](https://pubmed.ncbi.nlm.nih.gov/32947909/#full-view-affiliation-1)[2](https://pubmed.ncbi.nlm.nih.gov/32947909/#full-view-affiliation-2)[3](https://pubmed.ncbi.nlm.nih.gov/32947909/#full-view-affiliation-3), [Robert C Hickner](https://pubmed.ncbi.nlm.nih.gov/?sort=date&term=Hickner+RC&cauthor_id=32947909)[1](https://pubmed.ncbi.nlm.nih.gov/32947909/#full-view-affiliation-1)[2](https://pubmed.ncbi.nlm.nih.gov/32947909/#full-view-affiliation-2)[3](https://pubmed.ncbi.nlm.nih.gov/32947909/#full-view-affiliation-3)

**P)** Os participantes possuem mais de 18 anos de idade?

( ) Sim (X) Não

Este é um artigo de revisão ("A broader body of knowledge warrants the need for further investigation") e cita outros estudos, mas não descreve um estudo primário com participantes conduzido pelos autores.

**I)** A intervenção utilizada no estudo é creatina? Vale qualquer formato de administração

( ) Sim ( ) Não

**C)** Teve grupo controle?

( ) Sim ( ) Não

**O)** O estudo apresenta valores de marcadores inflamatórios (citocinas e outros marcadores) antes e após as intervenções de creatina?

( ) Sim ( ) Não

**S)** O estudo é um ensaio clínico randomizado e controlado?

( ) Sim ( ) Não

**Artigo 51:** Beyond muscles: The untapped potential of creatine

[Lisa A Riesberg](https://pubmed.ncbi.nlm.nih.gov/?term=%22Riesberg%20LA%22%5BAuthor%5D) 1, [Stephanie A Weed](https://pubmed.ncbi.nlm.nih.gov/?term=%22Weed%20SA%22%5BAuthor%5D) 1, [Thomas L McDonald](https://pubmed.ncbi.nlm.nih.gov/?term=%22McDonald%20TL%22%5BAuthor%5D) 2, [Joan M Eckerson](https://pubmed.ncbi.nlm.nih.gov/?term=%22Eckerson%20JM%22%5BAuthor%5D) 3, [Kristen M Drescher](https://pubmed.ncbi.nlm.nih.gov/?term=%22Drescher%20KM%22%5BAuthor%5D)

**P)** Os participantes possuem mais de 18 anos de idade?

( ) Sim (X) Não

"In humans, pilot data has examined both the short-term and long-term effect of creatine supplementation on TBI in children and adolescents".

**I)** A intervenção utilizada no estudo é creatina? Vale qualquer formato de administração

( ) Sim ( ) Não

**C)** Teve grupo controle?

( ) Sim ( ) Não

**O)** O estudo apresenta valores de marcadores inflamatórios (citocinas e outros marcadores) antes e após as intervenções de creatina?

( ) Sim ( ) Não

**S)** O estudo é um ensaio clínico randomizado e controlado?

( ) Sim ( ) Não

**Artigo 52:** Nutrition and Supplementation Considerations to Limit Endotoxemia When Exercising in the Heat

[Joshua H Guy](https://pubmed.ncbi.nlm.nih.gov/?sort=date&term=Guy+JH&cauthor_id=29910316)[1](https://pubmed.ncbi.nlm.nih.gov/29910316/#full-view-affiliation-1), [Grace E Vincent](https://pubmed.ncbi.nlm.nih.gov/?sort=date&term=Vincent+GE&cauthor_id=29910316)[2](https://pubmed.ncbi.nlm.nih.gov/29910316/#full-view-affiliation-2)

**P)** Os participantes possuem mais de 18 anos de idade?

( ) Sim (X) Não

Este é um artigo de revisão ("Recommendations and Future Research Directions") e discute descobertas de pesquisa de outros estudos, mas não descreve um estudo primário com participantes conduzido pelos autores.

**I)** A intervenção utilizada no estudo é creatina? Vale qualquer formato de administração

( ) Sim ( ) Não

**C)** Teve grupo controle?

( ) Sim ( ) Não

**O)** O estudo apresenta valores de marcadores inflamatórios (citocinas e outros marcadores) antes e após as intervenções de creatina?

( ) Sim ( ) Não

**S)** O estudo é um ensaio clínico randomizado e controlado?

( ) Sim ( ) Não

**Artigo 53:** Effect of nutritional interventions and resistance exercise on aging muscle mass and strength

[Darren G Candow](https://pubmed.ncbi.nlm.nih.gov/?sort=date&term=Candow+DG&cauthor_id=22684187)[1](https://pubmed.ncbi.nlm.nih.gov/22684187/#full-view-affiliation-1), [Scott C Forbes](https://pubmed.ncbi.nlm.nih.gov/?sort=date&term=Forbes+SC&cauthor_id=22684187), [Jonathan P Little](https://pubmed.ncbi.nlm.nih.gov/?sort=date&term=Little+JP&cauthor_id=22684187), [Stephen M Cornish](https://pubmed.ncbi.nlm.nih.gov/?sort=date&term=Cornish+SM&cauthor_id=22684187), [Craig Pinkoski](https://pubmed.ncbi.nlm.nih.gov/?sort=date&term=Pinkoski+C&cauthor_id=22684187), [Philip D Chilibeck](https://pubmed.ncbi.nlm.nih.gov/?sort=date&term=Chilibeck+PD&cauthor_id=22684187)

**P)** Os participantes possuem mais de 18 anos de idade?

( ) Sim (X) Não

Este é um artigo de revisão e lista referências, mas não descreve um estudo primário com participantes conduzido pelos autores.

**I)** A intervenção utilizada no estudo é creatina? Vale qualquer formato de administração

( ) Sim ( ) Não

**C)** Teve grupo controle?

( ) Sim ( ) Não

**O)** O estudo apresenta valores de marcadores inflamatórios (citocinas e outros marcadores) antes e após as intervenções de creatina?

( ) Sim ( ) Não

**S)** O estudo é um ensaio clínico randomizado e controlado?

( ) Sim ( ) Não

**Artigo 54:** The Effect of Creatine Supplementation on Markers of Exercise-Induced Muscle Damage: A Systematic Review and Meta-Analysis of Human Intervention Trials

[Bethany Northeast](https://pubmed.ncbi.nlm.nih.gov/?sort=date&term=Northeast+B&cauthor_id=33631721)[1](https://pubmed.ncbi.nlm.nih.gov/33631721/#full-view-affiliation-1), [Tom Clifford](https://pubmed.ncbi.nlm.nih.gov/?sort=date&term=Clifford+T&cauthor_id=33631721)[1](https://pubmed.ncbi.nlm.nih.gov/33631721/#full-view-affiliation-1)

**P)** Os participantes possuem mais de 18 anos de idade?

( ) Sim (X) Não

Esta é uma revisão sistemática e meta-análise, que sintetiza dados de "Thirteen studies (totalling 278 participants; 235 males, 43 females; age range 20 to 60 years)", mas não é um estudo primário conduzido pelos próprios autores.

**I)** A intervenção utilizada no estudo é creatina? Vale qualquer formato de administração

( ) Sim ( ) Não

**C)** Teve grupo controle?

( ) Sim ( ) Não

**O)** O estudo apresenta valores de marcadores inflamatórios (citocinas e outros marcadores) antes e após as intervenções de creatina?

( ) Sim ( ) Não

**S)** O estudo é um ensaio clínico randomizado e controlado?

( ) Sim ( ) Não

**Artigo 55:** Guanidino compounds after creatine supplementation in renal failure patients and their relation to inflammatory status

[Youri E C Taes](https://pubmed.ncbi.nlm.nih.gov/?sort=date&term=Taes+YE&cauthor_id=18048424)[1](https://pubmed.ncbi.nlm.nih.gov/18048424/#full-view-affiliation-1), [Bart Marescau](https://pubmed.ncbi.nlm.nih.gov/?sort=date&term=Marescau+B&cauthor_id=18048424), [An De Vriese](https://pubmed.ncbi.nlm.nih.gov/?sort=date&term=De+Vriese+A&cauthor_id=18048424), [Peter P De Deyn](https://pubmed.ncbi.nlm.nih.gov/?sort=date&term=De+Deyn+PP&cauthor_id=18048424), [Eva Schepers](https://pubmed.ncbi.nlm.nih.gov/?sort=date&term=Schepers+E&cauthor_id=18048424), [Raymond Vanholder](https://pubmed.ncbi.nlm.nih.gov/?sort=date&term=Vanholder+R&cauthor_id=18048424), [Joris R Delanghe](https://pubmed.ncbi.nlm.nih.gov/?sort=date&term=Delanghe+JR&cauthor_id=18048424)

**P)** Os participantes possuem mais de 18 anos de idade?

(X) Sim ( ) Não

"The baseline characteristics of the participants indicate a median age of "71 (66–77)" years for the twenty male maintenance haemodialysis patients recruited."

**I)** A intervenção utilizada no estudo é creatina? Vale qualquer formato de administração

( ) Sim (X) Não

"While the title refers to "creatine supplementation," the provided excerpts from the study's methods and results sections do not explicitly describe the administration of creatine as an intervention to the participants of this particular study."

**C)** Teve grupo controle?

( ) Sim ( ) Não

**O)** O estudo apresenta valores de marcadores inflamatórios (citocinas e outros marcadores) antes e após as intervenções de creatina?

( ) Sim ( ) Não

**S)** O estudo é um ensaio clínico randomizado e controlado?

( ) Sim ( ) Não

**Artigo 56: Creatine supplementation exacerbates ethanol-induced hepatic damage in**mice

Marinello, PC, Cella, PS, Testa, MTJ, Guirro, PB, Brito, WAS, Borges, FH, Cecchini, R, Cecchini, AL, Duarte, JA, Deminice, R

**P)** Os participantes possuem mais de 18 anos de idade?

( ) Sim (X) Não

"The study explicitly states that "Male Swiss mice were divided into three groups" as participants."

**I)** A intervenção utilizada no estudo é creatina? Vale qualquer formato de administração

( ) Sim ( ) Não

**C)** Teve grupo controle?

( ) Sim ( ) Não

**O)** O estudo apresenta valores de marcadores inflamatórios (citocinas e outros marcadores) antes e após as intervenções de creatina?

( ) Sim ( ) Não

**S)** O estudo é um ensaio clínico randomizado e controlado?

( ) Sim ( ) Não

**Artigo 57: A multi-ingredient nutritional supplement enhances exercise training-related reductions in markers of systemic inflammation in healthy older men**

Bell, KE, Snijders, T, Zulyniak, MA, Kumbhare, D, Parise, G, Chabowski, A, Phillips, SM

**P)** Os participantes possuem mais de 18 anos de idade?

(X) Sim ( ) Não

"The study focused on "healthy older men", indicating participants were over 18 years of age."

**I)** A intervenção utilizada no estudo é creatina? Vale qualquer formato de administração

(X) Sim ( ) Não

"The study involved a "multi-ingredient nutritional supplement" that, according to conversation history, "contained creatine at a dose of 5 g·d−1"."

**C)** Teve grupo controle?

(X) Sim ( ) Não

"The study compared the intervention group to a control group that received "a sugary drink ('CON')"

**O)** O estudo apresenta valores de marcadores inflamatórios (citocinas e outros marcadores) antes e após as intervenções de creatina?

(X) Sim ( ) Não

"The study evaluated the effect on "systemic inflammatory markers" including "tumor necrosis factor alpha ('TNF-α')" and "interleukin-6 ('IL-6')"."

**S)** O estudo é um ensaio clínico randomizado e controlado?

( ) Sim (X) Não

"The study is described as a controlled experiment comparing supplement intake to a sugary drink, but the provided excerpts do not explicitly state that it was a randomized trial."

**Artigo 58: Creatine supplementation does not decrease total plasma homocysteine in chronic hemodialysis patients**

Taes, YEC, Delanghe, JR, De Bacquer, D, Langlois, M, Stevens, L, Geerolf, I, Lameire, NH, De Vriese, AS

**P)** Os participantes possuem mais de 18 anos de idade?

(X) Sim ( ) Não

“Forty-five hemodialysis patients... with a **mean age of 70 ± 10 years (range 35–88)** were included in this study"

**I)** A intervenção utilizada no estudo é creatina? Vale qualquer formato de administração

(X) Sim ( ) Não

"The present study investigates the effects of **creatine supple-mentation** on tHcy in a vitamin-repleted chronic hemodialysis population". "Patients were treated with **creatine (2 g/day)** or placebo during 2 treatment periods of 4 weeks". "Creatine monohydrate (CreaPure®) was obtained from Degussa Bioactives (Freising, Germany)"

**C)** Teve grupo controle?

(X) Sim ( ) Não

"The study followed a double-blind, **placebo-controlled**, crossover design". "Patients were treated with creatine (2 g/day) or **placebo** during 2 treatment periods of 4 weeks"

**O)** O estudo apresenta valores de marcadores inflamatórios (citocinas e outros marcadores) antes e após as intervenções de creatina?

(X) Sim ( ) Não

"The prognostic inflammatory and nutritional index (PINI) was calculated as PINI = [(a1-acid glycoprotein (mg/L) × CRP (mg/L)]/[(albumin (g/L) × prealbumin (mg/L)]". Values for "CRP" and "a1-acid glycoprotein" are presented in Table 3, measured at baseline and after treatment periods.

**S)** O estudo é um ensaio clínico randomizado e controlado?

(X) Sim ( ) Não

"The study followed a **double-blind, placebo-controlled, crossover design**". "Patients received 2 g creatine or placebo daily in the evening during 2 treatment periods of 4 weeks, **in random order.**

**Artigo 59: Effects of a High Protein and Omega-3-Enriched Diet With or Without Creatine Supplementation on Markers of Soreness and Inflammation during 5 Consecutive Days of High Volume Resistance Exercise in Females**

Hayward, S, Wilborn, CD, Taylor, LW, Urbina, SL, Outlaw, JJ, Foster, CA, Roberts, MD

**P)** Os participantes possuem mais de 18 anos de idade?

(X) Sim ( ) Não

“Twenty-eight apparently healthy non-resistance trained females (age: 20±1 yr; body mass: 63.5±1.6 kg, height: 1.67±0.01 m) volunteered for the 9-week study.”

**I)** A intervenção utilizada no estudo é creatina? Vale qualquer formato de administração

(X) Sim ( ) Não

"Participants in the DI+C group were given '5 g/d of micronized creatine monohydrate (...) with their supplemental protein'

**C)** Teve grupo controle?

(X) Sim ( ) Não

"The study included multiple groups: a "Control (CTL) group", a "Dietary Intervention (DI) group", and a "DI+C group" (Dietary Intervention + Creatine)."

**O)** O estudo apresenta valores de marcadores inflamatórios (citocinas e outros marcadores) antes e após as intervenções de creatina?

( ) Sim (X) Não

Therefore, the primary purpose of this investigation was to examine if two different dietary interventions prevented increases in muscle soreness and/or sérum inflammation markers in females over a 5-day high vol- ume resistance training protocol. These dietary interven- tions included: a) a higher-protein diet supplemented with omega-3 fatty acids, and b) a higher-protein diet supple- mented with omega-3 fatty acids as well as creatine monohydrate. Notably, the intent of the 5-day consecutive training protocol was to elicit an accelerated overreaching response.

**S)** O estudo é um ensaio clínico randomizado e controlado?

( ) Sim ( ) Não

**Artigo 60: Effects of acute creatine supplementation on cardiac and vascular responses in older men; a**randomized controlled trial

Aron, A, Landrum, EJ, Schneider, AD, Via, M, Evans, L, Rawson, ES

**P)** Os participantes possuem mais de 18 anos de idade?

(X) Sim ( ) Não

"The study recruited 'Healthy male subjects (...) between the ages of 55 and 80'."

**I)** A intervenção utilizada no estudo é creatina? Vale qualquer formato de administração

(X) Sim ( ) Não

"Participants were 'randomly assigned to three groups: creatine, placebo and control. Creatine or placebo was provided for 7-day supplementation, at a dose of 20 g/day'."

**C)** Teve grupo controle?

(X) Sim ( ) Não

"The study included three groups: 'creatine, placebo and control'."

**O)** O estudo apresenta valores de marcadores inflamatórios (citocinas e outros marcadores) antes e após as intervenções de creatina?

( ) Sim (X) Não

"The primary outcomes measured included 'blood pressure (BP), ankle brachial index (ABI), cardio-ankle vascular index (CAVI), upstroke time (UT) and heart rate (HR), stroke volume (SV), contractility index (CI) and ejection fraction (EF)', but no inflammatory markers were listed as measured in this study."

**S)** O estudo é um ensaio clínico randomizado e controlado?

( ) Sim ( ) Não

**Artigo 61: Exercise and nutritional interventions for improving aging muscle health**

Forbes, SC, Little, JP, Candow, DG

**P)** Os participantes possuem mais de 18 anos de idade?

(X) Sim ( ) Não

The article is a review focusing on "Exercise and nutritional interventions for improving **aging muscle health**" and specifically addresses **sarcopenia**, which is defined as the "age-related loss in muscle mass and strength". It frequently refers to "older adults" throughout the text, for instance, discussing how interventions "could enhance some of the physiological adaptations from exercise training in **older adults**". Therefore, the population of interest and the subjects of the reviewed studies are explicitly individuals above 18 years old, and specifically, older adults.

**I)** A intervenção utilizada no estudo é creatina? Vale qualquer formato de administração

(X) Sim ( ) Não

The study explicitly identifies **creatine monohydrate** as a key nutritional intervention that "could enhance some of the physiological adaptations from exercise training in older adults". It dedicates a section to "Creatine" and discusses its benefits, stating that "creatine supplementation enhances isometric strength and body composition improvements following strength exercise training in older adults". The term "Creatine" is also listed as a keyword for the review.

**C)** Teve grupo controle?

( ) Sim (X) Não

The purpose of this review is to highlight how exercise and nutritional intervention strategies may benefit aging muscle.

**O)** O estudo apresenta valores de marcadores inflamatórios (citocinas e outros marcadores) antes e após as intervenções de creatina?

( ) Sim ( ) Não

**S)** O estudo é um ensaio clínico randomizado e controlado?

( ) Sim ( ) Não

**Artigo 62: Dietary creatine supplementation lowers hepatic triacylglycerol by increasing lipoprotein secretion in**rats**fed high-fat diet**

da Silva, RP, Leonard, KA, Jacobs, RL

**P)** Os participantes possuem mais de 18 anos de idade?

( ) Sim (X) Não

The participants in this study were **Sprague–Dawley rats**. The paper specifies that 15 healthy, 6-month-old adult male Wistar albino rats were used. Therefore, the concept of "over 18 years of age" is not applicable as this was an **animal study**, not a human study.

**I)** A intervenção utilizada no estudo é creatina? Vale qualquer formato de administração

( ) Sim ( ) Não

**C)** Teve grupo controle?

( ) Sim ( ) Não

**O)** O estudo apresenta valores de marcadores inflamatórios (citocinas e outros marcadores) antes e após as intervenções de creatina?

( ) Sim ( ) Não

**S)** O estudo é um ensaio clínico randomizado e controlado?

( ) Sim ( ) Não

**Artigo 63: A Nutritional Blend Suppresses the Inflammatory Response from Bronchial Epithelial**Cells**Induced by SARS-CoV-2**

Mateus-Silva, JR, Oliveira, CR, Brandao-Rangel, MAR, Silva-Reis, A, Olimpio, FRD, Zamarioli, LD, Aimbire, F, Vieira, RP

**P)** Os participantes possuem mais de 18 anos de idade?

(X) Sim ( ) Não

The study is an **in vitro** investigation utilizing human bronchial epithelial cells (BEAS-2B). These cells were co-cultured with whole blood collected from **SARS-CoV-2-infected male patients who were 30 to 40 years old**. Although the direct "participants" are cell lines, the biological material used was derived from human subjects explicitly stated to be adults within this age range.

**I)** A intervenção utilizada no estudo é creatina? Vale qualquer formato de administração

(X) Sim ( ) Não

The primary intervention investigated in this study is **ImmuneRecov™, a multi-ingredient nutritional blend**. The composition of this blend is clearly defined and explicitly includes **creatine** as one of its key components, alongside concentrated whey protein, glutamic acid, tryptophan, vitamins, and other ingredients. The discussion section further highlights creatine's role, stating that the "nutritional blend containing significant amounts of creatine exerted an important anti-inflammatory effect on BEAS-2B cells challenged with SARS-CoV-2".

**C)** Teve grupo controle?

(X) Sim ( ) Não

Yes, the study included multiple control conditions. The methodology involved comparing the effects of the nutritional blend on human bronchial epithelial cells (BEAS-2B) under different conditions: "Control" (unstimulated cells), "Blend" (cells treated only with the nutritional blend), "SarS-CoV-2" (cells stimulated with blood from a SARS-CoV-2-infected patient without the blend), and "SarS-CoV-2 + Blend" (cells stimulated with SARS-CoV-2 blood and treated with the nutritional blend). This comprehensive setup allows for a controlled evaluation of the intervention's effects.

**O)** O estudo apresenta valores de marcadores inflamatórios (citocinas e outros marcadores) antes e após as intervenções de creatina?

(X) Sim ( ) Não

The study clearly presents data on inflammatory markers. It measured the **levels of IL-1β, IL-6, and IL-10 cytokines in the supernatant** and their corresponding **mRNA expression** in the cells. Results explicitly show that "SARS-CoV-2 incubation resulted in increased levels of IL-1β and IL-6 in BEAS-2B cells (p < 0.001)". Furthermore, "Treatment with the nutritional blend resulted in reduced levels of the proinflammatory cytokines IL-1β and IL-6 (p < 0.001) and increased levels of the anti-inflammatory cytokine IL-10 (p < 0.001)". It also assessed IFN-γ. While "before" and "after" are implicitly compared through the different experimental groups (e.g., unstimulated vs. stimulated, and stimulated with vs. without blend), the study directly quantifies the impact of the interventions on these markers.

**S)** O estudo é um ensaio clínico randomizado e controlado?

( ) Sim (X) Não

This study is explicitly described as an "**in vitro study**" that "tested the hypothesis that ImmuneRecov™, a nutritional blend, inhibits the SARS-CoV-2-induced hyperactivation of human bronchial epithelial cells (BEAS-2B)". It utilized a human cell line (BEAS-2B) and human blood samples in a laboratory setting. A "clinical trial" refers to research conducted on living human subjects. Therefore, despite having controlled conditions, it is not a randomized controlled clinical trial.

**Artigo 64: Creatine supplementation effect on recovery following exercise-induced muscle damage: A**systematic review**and**meta-analysis**of**randomized controlled trials

Yue, JM, Rahimi, MH

**P)** Os participantes possuem mais de 18 anos de idade?

(X) Sim ( ) Não

"Nine studies met the inclusion criteria. Pooled data showed... and aged **19.9 – 37.5 years**". The review "studied a total of 630 and 420 participants respectively and aged 19.9 – 37.5 years".

**I)** A intervenção utilizada no estudo é creatina? Vale qualquer formato de administração

(X) Sim ( ) Não

The title of the article is "Creatine Supplementation Effect on Recovery Following Exercise-Induced Muscle Damage: A Systematic Review and Meta-analysis of Randomized Controlled Trials". The "Eligibility Criteria" for included studies stated "healthy subjects received **oral creatine supplementation**, as a nutritional strategy". The dose of creatine supplementation in the included studies "was **2 to 40 g/day**".

**C)** Teve grupo controle?

(X) Sim ( ) Não

Yes, the meta-analysis included studies that utilized control groups. The abstract states that pooled data showed "creatine significantly reduced CK concentration overall... in comparison with **placebo**". The "Eligibility Criteria" specified inclusion of "original **randomized-controlled trial** researches" which "provided muscle damage indices data as mean and standard deviation (SD) in both intervention and **placebo groups**". Furthermore, the included studies "All studies used a **randomized parallel design**, and all of them had the design of **double-blind** except two studies (36, 37)"

**O)** O estudo apresenta valores de marcadores inflamatórios (citocinas e outros marcadores) antes e após as intervenções de creatina?

( ) Sim (X) Não

The study, as a meta-analysis, primarily focused on and presented aggregated data for **muscle damage indices** such as "serum creatine kinase (CK) and lactate dehydrogenase (LDH) concentrations". While muscle damage can induce inflammation, CK and LDH are specific markers of muscle damage, not direct inflammatory mediators like cytokines (e.g., IL-6, TNF-alpha) or other inflammatory markers (e.g., PGE2, CRP). The abstract and main results sections of this meta-analysis do not present pooled values for these direct inflammatory markers before and after creatine interventions.

**S)** O estudo é um ensaio clínico randomizado e controlado?

( ) Sim ( ) Não

**Artigo 65: Effect of Creatine Supplementation on the Airways of Youth Elite Soccer Players**

Simpson, AJ, Horne, S, Sharp, P, Sharps, R, Kippelen, P

**P)** Os participantes possuem mais de 18 anos de idade?

( ) Sim (X) Não

"Nineteen elite soccer players, **16–21 yr old**, completed a stratified, randomized, double-blind, placebo-controlled, parallel-group trial". Further, "Nineteen under 18 (U18) and nine under 21 (U21), non-smoking, male, elite soccer players from Watford Football Club (FC) Academy agreed to take part in this project". "At study entry, participants were **17.4 ± 1.6 yr of age**".

**I)** A intervenção utilizada no estudo é creatina? Vale qualquer formato de administração

( ) Sim ( ) Não

**C)** Teve grupo controle?

( ) Sim ( ) Não

**O)** O estudo apresenta valores de marcadores inflamatórios (citocinas e outros marcadores) antes e após as intervenções de creatina?

( ) Sim ( ) Não

**S)** O estudo é um ensaio clínico randomizado e controlado?

( ) Sim ( ) Não

**Artigo 66: Effect of Creatine on**Rat**Sciatic Nerve Injury: A Comparative Ultrastructural Study**

Helvacioglu, F, Kandemir, E, Karabacak, B, Karatas, I, Pecen, A, Ercan, I, Sencelikel, T, Dagdeviren, A

**P)** Os participantes possuem mais de 18 anos de idade?

( ) Sim (X) Não

"The study was performed in **15 healthy, 6-month-old adult male Wistar albino rats**". This study was conducted on animals (rats), not human participants, and therefore the concept of "more than 18 years old" for participants does not apply.

**I)** A intervenção utilizada no estudo é creatina? Vale qualquer formato de administração

( ) Sim ( ) Não

**C)** Teve grupo controle?

( ) Sim ( ) Não

**O)** O estudo apresenta valores de marcadores inflamatórios (citocinas e outros marcadores) antes e após as intervenções de creatina?

( ) Sim ( ) Não

**S)** O estudo é um ensaio clínico randomizado e controlado?

( ) Sim ( ) Não

**Artigo 67: Nutritional Compounds to Improve Post-Exercise Recovery**

O'Connor, E, Muendel, T, Barnes, MJ

**P)** Os participantes possuem mais de 18 anos de idade?

(X) Sim ( ) Não

This article is a review that synthesizes findings from previous research on nutritional compounds for post-exercise recovery. The reviewed studies commonly involve adult populations. For instance, the text refers to studies on "resistance trained males", "young men", and "healthy older adults", indicating that the subjects of the primary research are typically over 18 years of age.

**I)** A intervenção utilizada no estudo é creatina? Vale qualquer formato de administração

(X) Sim ( ) Não

Yes, creatine is one of the nutritional compounds evaluated in this review. The article explicitly lists it: "Of the compounds included in this report, only two—tart cherry and omega-3 fatty acids—are supported by significant research evidence... while a further five—BCAAs, HMB, **creatine monohydrate**, curcumin and pomegranate—appear to have beneficial effects but evidence of this is scarce"

**C)** Teve grupo controle?

(X) Sim ( ) Não

As a review article, this study analyzes and summarizes findings from original research. Many of the studies it references are randomized controlled trials, which inherently include control or placebo groups. For example, in its discussion of creatine, it mentions comparisons between creatine supplementation and other conditions: "In contrast, several other studies have shown no positive effects of similar Cr supplementation protocols on markers of muscle damage or physical recovery following resistance-based exercise", implicitly referring to studies with control groups. The methodologies of the summarized studies include such designs, as seen with omega-3 fatty acids where it references "a randomized, double-blind, placebo-controlled, parallel-group trial"

**O)** O estudo apresenta valores de marcadores inflamatórios (citocinas e outros marcadores) antes e após as intervenções de creatina?

( ) Sim (X) Não

While the review discusses inflammatory markers in general and for other compounds (e.g., curcumin), for creatine specifically, it states a lack of such evidence: "**Despite the proposed benefit of Cr on the inflammatory response, research evidence in this area is lacking**, and the exact mechanism by which Cr may exert positive effects on post-exercise recovery is unknown". Therefore, it does not present these values for creatine interventions.

**S)** O estudo é um ensaio clínico randomizado e controlado?

( ) Sim ( ) Não

**Artigo 68: Effect of bench press exercise intensity on muscle soreness and inflammatory mediators**

Uchida, MC, Nosaka, K, Ugrinowitsch, C, Yamashita, A, Martins, E, Moriscot, AS, Aoki, MS

**P)** Os participantes possuem mais de 18 anos de idade?

(X) Sim ( ) Não

"Thirty-five male Brazilian Army soldiers were randomly assigned to one of five groups". The mean age of participants across the groups was "Age (years) **18.6 ± 0.6**" for the control group and ranged up to "19.5 ± 1.9" and "19.4 ± 2.5" for the exercise groups. This indicates all participants were 18 years of age or older.

**I)** A intervenção utilizada no estudo é creatina? Vale qualquer formato de administração

( ) Sim (X) Não

The study's primary intervention was **"four different intensities of a bench press exercise"** to compare their effects on muscle soreness and inflammatory mediators. The "main aim of the present study was to test the hypothesis that the magnitude of muscle soreness after a bench press exercise would not be dependent on the intensity if the total volume of the exercise was matched". **Creatine kinase activity was measured as a marker of muscle damage**, not administered as an intervention. The study does not mention creatine supplementation as an intervention.

**C)** Teve grupo controle?

( ) Sim ( ) Não

**O)** O estudo apresenta valores de marcadores inflamatórios (citocinas e outros marcadores) antes e após as intervenções de creatina?

( ) Sim ( ) Não

**S)** O estudo é um ensaio clínico randomizado e controlado?

( ) Sim ( ) Não

**Artigo 69: Muscle loss in cancer cachexia: what is the basis for nutritional support?**

Faiad, J, Andrade, MF, de Castro, G, de Resende, J, Coêlho, M, Aquino, G, Seelaender, M

**P)** Os participantes possuem mais de 18 anos de idade?

(X) Sim ( ) Não

This is a review article that synthesizes evidence from various studies. The primary focus of the review is "muscle loss in cancer cachexia" and "nutritional support" for "cancer patients". Cancer cachexia is a condition typically affecting adult and older adult human populations. For instance, the article refers to a "randomized clinical trial reported the effects of the total daily protein intake on patients with stage II-IV colorectal cancer" and discusses "quality of life" and "response to treatment" in this context, which are aspects related to adult human subjects. While some cited studies may involve animal models, the overall scope pertains to human clinical relevance, implying that the human "participants" of the summarized research are generally over 18 years of age.

**I)** A intervenção utilizada no estudo é creatina? Vale qualquer formato de administração

(X) Sim ( ) Não

Yes, creatine is explicitly discussed as a nutritional intervention in the study. The article includes a dedicated section titled "1.6 Creatine supplementation" and mentions that "Studies in rats with cachexia showed that **creatine supplementation** attenuated weight loss and decreased tumor growth". It also refers to "the recommended dose for humans" for creatine and its effects on "patients with cachexia".

**C)** Teve grupo controle?

(X) Sim ( ) Não

As a review article, this study synthesizes evidence from original research, many of which are designed with control groups. For example, the article explicitly mentions a "randomized clinical trial" in the context of protein intake and refers to a "randomized, double-blind, controlled study" for HMB, arginine, and glutamine supplementation. Specifically, in the section on creatine, it cites studies on rats and humans that would typically involve control or placebo groups for comparison, and indeed, one of its cited references (Jatoi et al., 2017) is a "double-blind, placebo-controlled randomized trial of creatine".

**O)** O estudo apresenta valores de marcadores inflamatórios (citocinas e outros marcadores) antes e após as intervenções de creatina?

(X) Sim ( ) Não

Yes, the review discusses the effects of creatine interventions on inflammatory markers. It states that "Studies in rats with cachexia showed that creatine supplementation... promoted lower plasma concentration of **TNF-α and IL-6 (pro-inflammatory cytokines), while increasing the concentration of IL-10 (anti-inflammatory cytokine)**, and preventing atrogin-1 and MuRF-1, key regulators of muscle atrophy in the skeletal muscle". While the review itself does not present raw data, it reports findings from original studies that measured these markers before and after creatine interventions.

**S)** O estudo é um ensaio clínico randomizado e controlado?

( ) Sim (X) Não

No, this study is not a randomized controlled clinical trial. It is explicitly stated as a "**Review Article**" that "discusses the most studied supplements and nutritional strategies for dealing with muscle loss in CC". The purpose of the article is to synthesize and explore existing literature on nutritional support for cancer cachexia, not to conduct new experimental research.

**Artigo 70: Prophylactic Fetal Creatine Supplementation Improves Post-Asphyxial EEG Recovery and Reduces Seizures in Fetal Sheep: Implications for Hypoxic-Ischemic Encephalopathy**

Tran, NT, Ellery, SJ, Kelly, SB, Sévigny, J, Chatton, M, Lu, H, Polglase, GR, Snow, RJ, Walker, DW, Galinsky, R

**P)** Os participantes possuem mais de 18 anos de idade?

( ) Sim (X) Não

The study was conducted on "Fetal sheep (118 +/- 1 days’ gestational age [dGA]; 0.8 gestation)". This clearly indicates that the participants were **fetal animals** and not human individuals over 18 years of age.

**I)** A intervenção utilizada no estudo é creatina? Vale qualquer formato de administração

( ) Sim ( ) Não

**C)** Teve grupo controle?

( ) Sim ( ) Não

**O)** O estudo apresenta valores de marcadores inflamatórios (citocinas e outros marcadores) antes e após as intervenções de creatina?

( ) Sim ( ) Não

**S)** O estudo é um ensaio clínico randomizado e controlado?

( ) Sim ( ) Não

**Artigo 71: Etiological drug therapy for ALS**

Cintas, P

**P)** Os participantes possuem mais de 18 anos de idade?

( ) Sim ( ) Não [*Excluir*]

**I)** A intervenção utilizada no estudo é creatina? Vale qualquer formato de administração

( ) Sim ( ) Não [*Excluir*]

**C)** Teve grupo controle?

( ) Sim ( ) Não [*Excluir*]

**O)** O estudo apresenta valores de marcadores inflamatórios (citocinas e outros marcadores) antes e após as intervenções de creatina?

( ) Sim ( ) Não [*Excluir*]

**S)** O estudo é um ensaio clínico randomizado e controlado?

( ) Sim ( ) Não [*Excluir*]

**Artigo 72: The emergence of sarcopenia as an important entity in older people**

Offord, NJ, Witham, MD

**P)** Os participantes possuem mais de 18 anos de idade?

(X) Sim ( ) Não

The article is a review focusing on "Sarcopenia refers to the loss of muscle mass and strength seen with **advancing age**". It further defines sarcopenia as a syndrome in "**older people**" and mentions the diagnosis algorithm for "**older adults (age >65 years)**". This clearly indicates the focus is on a population over 18 years of age.

**I)** A intervenção utilizada no estudo é creatina? Vale qualquer formato de administração

( ) Sim (X) Não

This is a "**Review Article**" that discusses various potential interventions for sarcopenia. It states that "Resistance training is the only intervention of proven effi cacy to treat sarcopenia, but a range of nutritional and pharmacological interventions are under test, including myostatin inhibitors, leucine and protein supplementation, angiotensin-converting enzyme inhibitors and allopurinol". Creatine is mentioned as one of the nutritional interventions that has been studied in "older adults" for sarcopenia (e.g., citing a meta-analysis on "Creatine supplementation during resistance training in older adults"), but the review itself does not **utilize** creatine as an intervention; rather, it synthesizes findings from other studies that have.

**C)** Teve grupo controle?

( ) Sim ( ) Não

**O)** O estudo apresenta valores de marcadores inflamatórios (citocinas e outros marcadores) antes e após as intervenções de creatina?

( ) Sim ( ) Não

**S)** O estudo é um ensaio clínico randomizado e controlado?

( ) Sim ( ) Não

**Artigo 73: Dietary supplementation of guanidinoacetic acid improves growth, biochemical parameters, antioxidant capacity and cytokine responses in Nile tilapia (Oreochromis niloticus)**

Aziza, A, Mahmoud, R, Zahran, E, Gadalla, H

**P)** Os participantes possuem mais de 18 anos de idade?

( ) Sim (X) Não

The study clearly states that the participants were "**One hundred and eighty unsexed Nile tilapia fish** weighing 21.20 ± 1.78 gm". This indicates that the study subjects were **fish**, not human individuals over 18 years of age.

**I)** A intervenção utilizada no estudo é creatina? Vale qualquer formato de administração

( ) Sim ( ) Não

**C)** Teve grupo controle?

( ) Sim ( ) Não

**O)** O estudo apresenta valores de marcadores inflamatórios (citocinas e outros marcadores) antes e após as intervenções de creatina?

( ) Sim ( ) Não

**S)** O estudo é um ensaio clínico randomizado e controlado?

( ) Sim ( ) Não

**Artigo 74: Effects of various dietary supplements on inflammatory processes in primary**canine**chondrocytes as a model of osteoarthritis**

AlRaddadi, EA, Winter, T, Aukerna, HM, Miller, DW

**P)** Os participantes possuem mais de 18 anos de idade?

( ) Sim (X) Não

The study specifies that it aimed to evaluate effects "in **primary cultured canine chondrocytes (CnCs) as an in-vitro model of OA**". These "CnCs derived from normal canine articular cartilage were purchased from Cell Applications". This means the participants were **dog cells used in a laboratory setting (in-vitro)**, not human individuals over 18 years of age.

**I)** A intervenção utilizada no estudo é creatina? Vale qualquer formato de administração

( ) Sim ( ) Não

**C)** Teve grupo controle?

( ) Sim ( ) Não

**O)** O estudo apresenta valores de marcadores inflamatórios (citocinas e outros marcadores) antes e após as intervenções de creatina?

( ) Sim ( ) Não

**S)** O estudo é um ensaio clínico randomizado e controlado?

( ) Sim ( ) Não

**Artigo 75: Consumption of cherries as a strategy to attenuate exercise-induced muscle damage and inflammation in humans**

de Lima, LCR, Assumpçao, CD, Prestes, J, Denadai, BS

**P)** Os participantes possuem mais de 18 anos de idade?

(X) Sim ( ) Não

This is a review article focused on cherry consumption in "**humans**". The review synthesizes findings from various studies involving adult populations, such as "healthy women", "healthy men and women", "male college students", and "runners participating in the London marathon". These populations are generally comprised of individuals over 18 years of age.

**I)** A intervenção utilizada no estudo é creatina? Vale qualquer formato de administração

( ) Sim (X) Não

The title of the article clearly indicates that the study is about the "**Consumption of cherries** as a strategy to attenuate exercise-induced muscle damage and inflammation in humans". The abstract further states, "The articles identified in this review support the notion that **tart cherry consumption** attenuates EIMD symptoms after intense exercise bouts". While creatine is mentioned in the references as a topic of other supplementation studies, the specific intervention being reviewed and discussed in *this* study is cherry consumption.

**C)** Teve grupo controle?

( ) Sim ( ) Não

**O)** O estudo apresenta valores de marcadores inflamatórios (citocinas e outros marcadores) antes e após as intervenções de creatina?

( ) Sim ( ) Não

**S)** O estudo é um ensaio clínico randomizado e controlado?

( ) Sim ( ) Não

**Artigo 76: Inflammation and cognitive performance in elite athletes: A**cross-sectional**study**

Wiedenbrüg, K, Will, L, Reichert, L, Hacker, S, Lenz, C, Zentgraf, K, Raab, M, Krüger, K

**P)** Os participantes possuem mais de 18 anos de idade?

(X) Sim ( ) Não

The study included "**350 elite athletes (184 female; Mage = 20.2 ± 4.6 years)**". The mean age of 20.2 years clearly indicates that the participants were, on average, over 18 years old.

**I)** A intervenção utilizada no estudo é creatina? Vale qualquer formato de administração

( ) Sim (X) Não

The study is titled "Inflammation and cognitive performance in elite athletes: A **cross-sectional study**". The abstract further clarifies that "Data from 350 elite athletes regarding cognitive performance (...), systemic inflammatory markers, metabolic hormones, growth factors, tissue damage markers, and micronutrients (...) were **analysed by correlative and multiple regression analyses**". This indicates an observational study design that analyzes relationships between existing markers, not an intervention where creatine was administered. While creatine is discussed as a precursor to creatinine and its importance for cognition, it was not the intervention *of this study*.

**C)** Teve grupo controle?

( ) Sim ( ) Não

**O)** O estudo apresenta valores de marcadores inflamatórios (citocinas e outros marcadores) antes e após as intervenções de creatina?

( ) Sim ( ) Não

**S)** O estudo é um ensaio clínico randomizado e controlado?

( ) Sim ( ) Não

**Artigo 77: Nutritional Intervention during Muscle Injury Considering its Pathophysiology: Review Article**

Vergara-Gutiérrez, L, Lizárraga-Dallo, A, Pruna-Grive, R

**P)** Os participantes possuem mais de 18 anos de idade?

(X) Sim ( ) Não

The study is a review of literature on nutritional intervention during muscle injury. It states that "the best available indirect evidence was sought such as nutritional intervention in the recovery of muscle inflammation, **sarcopenia in older adults** or in patients hospitalised after surgery". Studies on "older adults" inherently involve participants over 18 years of age.

**I)** A intervenção utilizada no estudo é creatina? Vale qualquer formato de administração

( ) Sim (X) Não

The article is a "**Review Article**" focused on "Nutritional Intervention during Muscle Injury". It states that it discusses "dietary supplements including whey protein, **creatine**, HMB and anti-inflammatories such as curcumin and tart cherry extract among others". Creatine is one of *several* supplements reviewed, not the sole intervention of this study.

**C)** Teve grupo controle?

( ) Sim ( ) Não

**O)** O estudo apresenta valores de marcadores inflamatórios (citocinas e outros marcadores) antes e após as intervenções de creatina?

( ) Sim ( ) Não

**S)** O estudo é um ensaio clínico randomizado e controlado?

( ) Sim ( ) Não

**Artigo 78: Effects of hemp supplementation during resistance training in trained young adults**

Kaviani, M, Shaw, KA, Candow, DG, Farthing, JP, Chilibeck, PD

**P)** Os participantes possuem mais de 18 anos de idade?

(X) Sim ( ) Não

The study included "Males (n = 22, **29 ± 8y**) and females (n = 12, **30 ± 9y**)". The **inclusion criteria** further specified "aged **18-45 years**". Therefore, all participants were over 18 years of age.

**I)** A intervenção utilizada no estudo é creatina? Vale qualquer formato de administração

( ) Sim (X) Não

The study investigated the "Effects of **hemp supplementation** during resistance training in trained young adults". Participants were randomized to receive either "**60 g/d of hemp**" or "**60 g/d of soy**". Creatine was explicitly listed as a supplement that participants should *not* be taking prior to the study, indicating it was an exclusion criterion for existing supplement use, not the intervention itself.

**C)** Teve grupo controle?

( ) Sim ( ) Não

**O)** O estudo apresenta valores de marcadores inflamatórios (citocinas e outros marcadores) antes e após as intervenções de creatina?

( ) Sim ( ) Não

**S)** O estudo é um ensaio clínico randomizado e controlado?

( ) Sim ( ) Não

**Artigo 79: Sarcopenia and type 2 diabetes: Pathophysiology and potential therapeutic lifestyle interventions**

Marcotte-Chénard, A, Oliveira, B, Little, JP, Candow, DG

**P)** Os participantes possuem mais de 18 anos de idade?

( ) Sim (X) Não

The article is a review focusing on "Sarcopenia and type 2 diabetes," which are conditions primarily affecting "older adults". The abstract explicitly states that sarcopenia "generally refers to the **age-related reduction** in muscle strength, functional ability, and muscle mass". Therefore, the population of interest for this review is well over 18 years of age.

**I)** A intervenção utilizada no estudo é creatina? Vale qualquer formato de administração

( ) Sim (X) Não

The article is a "**narrative review**". It does not *perform* an intervention itself. Instead, its purpose is to "**discuss lifestyle interventions involving physical activity and nutrition that may counteract sarcopenia and T2D**". While it mentions creatine as one of the nutritional interventions that "hold potential to augment some of these benefits from exercise", it is not the intervention *utilized by this study*.

**C)** Teve grupo controle?

( ) Sim ( ) Não

**O)** O estudo apresenta valores de marcadores inflamatórios (citocinas e outros marcadores) antes e após as intervenções de creatina?

( ) Sim ( ) Não

**S)** O estudo é um ensaio clínico randomizado e controlado?

( ) Sim ( ) Não

**Artigo 80: The effects of a multi-ingredient supplement on markers of muscle damage and inflammation following downhill running in females**

Köhne, JL, Ormsbee, MJ, McKune, AJ

**P)** Os participantes possuem mais de 18 anos de idade?

(X) Sim ( ) Não

The study included "Eight healthy female endurance-trained runners... **aged 18-40 years**". Therefore, all participants were over 18 years of age.

**I)** A intervenção utilizada no estudo é creatina? Vale qualquer formato de administração

( ) Sim (X) Não

The intervention utilized in this study was a "**multi-ingredient performance supplement (MIPS)** (NO-Shotgun®)". This blend, while it "**includes multiple forms of creatine** and beta alanine;... caffeine;" among other ingredients, is a multi-ingredient product, not solely creatine. The study aimed to determine the impact of the MIPS, not isolated creatine.

**C)** Teve grupo controle?

( ) Sim ( ) Não

**O)** O estudo apresenta valores de marcadores inflamatórios (citocinas e outros marcadores) antes e após as intervenções de creatina?

( ) Sim ( ) Não

**S)** O estudo é um ensaio clínico randomizado e controlado?

( ) Sim ( ) Não

**Artigo 81: Oxidative stress and inflammation: liver responses and adaptations to acute and regular exercise**

Barcelos, RP, Royes, LFF, Gonzalez-Gallego, J, Bresciani, G

**P)** Os participantes possuem mais de 18 anos de idade?

( ) Sim ( ) Não [*Excluir*]

**I)** A intervenção utilizada no estudo é creatina? Vale qualquer formato de administração

( ) Sim ( ) Não [*Excluir*]

**C)** Teve grupo controle?

( ) Sim ( ) Não [*Excluir*]

**O)** O estudo apresenta valores de marcadores inflamatórios (citocinas e outros marcadores) antes e após as intervenções de creatina?

( ) Sim ( ) Não [*Excluir*]

**S)** O estudo é um ensaio clínico randomizado e controlado?

( ) Sim ( ) Não [*Excluir*]

**Artigo 82: Antenatal prevention of cerebral palsy and childhood disability: is the impossible possible?**

Ellery, SJ, Kelleher, M, Grigsby, P, Burd, I, Derks, JB, Hirst, J, Miller, SL, Sherman, LS, Tolcos, M, Walker, DW

**P)** Os participantes possuem mais de 18 anos de idade?

( ) Sim (X) Não

The source focuses on "antenatal prevention of cerebral palsy and childhood disability" and discusses "fetal brain injury" and interventions "safely administered to women in late pregnancy" to decrease "perinatal brain damage". Specific treatments mentioned are intended to "protect the neonatal brain" or are given to "newborn preterm babies" and "asphyxiated newborns". This indicates that the primary subjects of the study outcomes are fetuses and neonates, not individuals over 18 years of age.

**I)** A intervenção utilizada no estudo é creatina? Vale qualquer formato de administração

( ) Sim ( ) Não

**C)** Teve grupo controle?

( ) Sim ( ) Não

**O)** O estudo apresenta valores de marcadores inflamatórios (citocinas e outros marcadores) antes e após as intervenções de creatina?

( ) Sim ( ) Não

**S)** O estudo é um ensaio clínico randomizado e controlado?

( ) Sim ( ) Não

**Artigo 83: Nutritional and Pharmacological Interventions to Expedite Recovery Following Muscle-Damaging Exercise in Older Adults: A Narrative Review of the Literature**

Clifford, T

**P)** Os participantes possuem mais de 18 anos de idade?

( ) Sim ( ) Não [*Excluir*]

**I)** A intervenção utilizada no estudo é creatina? Vale qualquer formato de administração

( ) Sim ( ) Não [*Excluir*]

**C)** Teve grupo controle?

( ) Sim ( ) Não [*Excluir*]

**O)** O estudo apresenta valores de marcadores inflamatórios (citocinas e outros marcadores) antes e após as intervenções de creatina?

( ) Sim ( ) Não [*Excluir*]

**S)** O estudo é um ensaio clínico randomizado e controlado?

( ) Sim ( ) Não [*Excluir*]

**Artigo 84: Supplementation with**fish**oil reduces morphological aspects of muscle damage induced by intense exercise in**rats

Coqueiro, DP, Bueno, PCD, Simoes, MD

**P)** Os participantes possuem mais de 18 anos de idade?

( ) Sim (X) Não

The study's abstract and methods clearly state that the research was conducted on "Wistar rats" and refers to the subjects as "animals". Therefore, the participants are not over 18 years of age.

**I)** A intervenção utilizada no estudo é creatina? Vale qualquer formato de administração

( ) Sim ( ) Não

**C)** Teve grupo controle?

( ) Sim ( ) Não

**O)** O estudo apresenta valores de marcadores inflamatórios (citocinas e outros marcadores) antes e após as intervenções de creatina?

( ) Sim ( ) Não

**S)** O estudo é um ensaio clínico randomizado e controlado?

( ) Sim ( ) Não

**Artigo 85: Dietary Amino Acids and Immunonutrition Supplementation in Cancer-Induced Skeletal Muscle Mass Depletion: A Mini-Review**

Soares, JDP, Howell, SL, Teixeira, FJ, Pimentel, GD

**P)** Os participantes possuem mais de 18 anos de idade?

(X) Sim ( ) Não

The review article states its methods included a search for "human and animal model studies" and specifically mentions that "40 studies, in both animals and humans, that investigated the effects of amino acids and their derivatives on sarcopenia, cachexia, immunity, and cancer were included". The abstract further notes that "cancer-induced cachexia is similar but distinctly different from sarcopenia and higher in elderly patients than in their younger counterparts depending on the type, location and/or stage of cancer", indicating the inclusion of studies on adult and elderly human participants.

**I)** A intervenção utilizada no estudo é creatina? Vale qualquer formato de administração

(X) Sim ( ) Não

The abstract explicitly lists "creatine" as one of the supplements whose effects have been "carefully examined" within the context of cancer-induced cachexia and skeletal muscle depletion. Furthermore, a dedicated section within the review is titled "Creatine" and discusses its effects.

**C)** Teve grupo controle?

( ) Sim (X) Não

This document is a "Mini-Review Article". As a review, it synthesizes and discusses findings from previously published "clinical trial studies" and "animal model studies", which themselves may have included control groups. However, the review article itself is not an original research study or clinical trial and therefore does not have its own experimental or control groups.

**O)** O estudo apresenta valores de marcadores inflamatórios (citocinas e outros marcadores) antes e após as intervenções de creatina?

( ) Sim ( ) Não

**S)** O estudo é um ensaio clínico randomizado e controlado?

( ) Sim ( ) Não

**Artigo 86: Nutrition for Acute Exercise-Induced Injuries**

Tipton, KD

**P)** Os participantes possuem mais de 18 anos de idade?

(X) Sim ( ) Não

The review discusses nutritional support for "any exerciser from those exercising for health and enjoyment up to the elite athlete", and specifically mentions studies involving "young, healthy individuals" and "elderly patients". This indicates that the review considers research conducted on adult populations.

**I)** A intervenção utilizada no estudo é creatina? Vale qualquer formato de administração

(X) Sim ( ) Não

The article explicitly states that "Creatine supplementation is another interesting potential nutritional countermeasure to muscle loss" and dedicates a section to it, discussing its use in various contexts like "creatine supplementation during 2 weeks of lower-limb casting" and "creatine supplementation for 12 weeks following anterior cruciate ligament surgery".

**C)** Teve grupo controle?

( ) Sim (X) Não

This document is clearly identified as a "REVIEW ARTICLE". As a review, it synthesizes and critically evaluates information from previously published studies, including those that might have had control groups, but the review itself does not conduct an experiment and therefore does not have its own experimental or control groups.

**O)** O estudo apresenta valores de marcadores inflamatórios (citocinas e outros marcadores) antes e após as intervenções de creatina?

( ) Sim ( ) Não

**S)** O estudo é um ensaio clínico randomizado e controlado?

( ) Sim ( ) Não

**Artigo 87: Gene expression, fiber type, and strength are similar between left and right legs in older adults**

Tarnopolsky, M, Phillips, S, Parise, G, Varbanov, A, DeMuth, J, Stevens, P, Qu, A, Wang, F, Isfort, R

**P)** Os participantes possuem mais de 18 anos de idade?

(X) Sim ( ) Não

The study explicitly states that participants were "eight older (71 ± 2 years) men" and "eight older males (> 65 years) volunteered for the study". This clearly indicates that the participants are over 18 years of age.

**I)** A intervenção utilizada no estudo é creatina? Vale qualquer formato de administração

( ) Sim (X) Não

The article's abstract and methods clearly state that its purpose was "to compare the transcriptome expression pattern and muscle fiber characteristics in the Vastus lateralis between the right and left leg of the same eight older adults, taken simultaneously". There is no mention of creatine or any other nutritional supplementation being used as an intervention in this study.

**C)** Teve grupo controle?

( ) Sim ( ) Não

**O)** O estudo apresenta valores de marcadores inflamatórios (citocinas e outros marcadores) antes e após as intervenções de creatina?

( ) Sim ( ) Não

**S)** O estudo é um ensaio clínico randomizado e controlado?

( ) Sim ( ) Não

**Artigo 88: Optimal management of sarcopenia**

Burton, LA, Sumukadas, D

**P)** Os participantes possuem mais de 18 anos de idade?

(X) Sim ( ) Não

The review article consistently discusses sarcopenia in the context of "aging muscle", "older people", and "frail older person". It also references diagnostic criteria for sarcopenia based on comparisons to "healthy younger adults", clearly indicating that the population of interest for this review comprises individuals over 18 years of age.

**I)** A intervenção utilizada no estudo é creatina? Vale qualquer formato de administração

( ) Sim (X) Não

This review article, titled "Optimal management of sarcopenia", discusses various "Treatment options in sarcopenic nursing home inhabitants", including "physical training, modifications of nutritional intake, and pharmacological substances". While it provides a table of therapeutic options, **creatine is not listed or discussed as an intervention within this review article**.

**C)** Teve grupo controle?

( ) Sim ( ) Não

**O)** O estudo apresenta valores de marcadores inflamatórios (citocinas e outros marcadores) antes e após as intervenções de creatina?

( ) Sim ( ) Não

**S)** O estudo é um ensaio clínico randomizado e controlado?

( ) Sim ( ) Não

**Artigo 89: Dietary Creatine Supplementation in Gilthead Seabream (Sparus aurata): Comparative Proteomics Analysis on**Fish**Allergens, Muscle Quality, and Liver**

Schrama, D, Cerqueira, M, Raposo, CS, da Costa, AMR, Wulff, T, Gonçalves, A, Camacho, C, Colen, R, Fonseca, F, Rodrigues, PM

**P)** Os participantes possuem mais de 18 anos de idade?

( ) Sim (X) Não

The study explicitly states under "MATERIALS AND METHODS" that "For this trial, 24 **gilthead seabream** per tank were reared". The entire article focuses on the study of **fish** (Sparus aurata), not human subjects, meaning the participants are not over 18 years of age.

**I)** A intervenção utilizada no estudo é creatina? Vale qualquer formato de administração

( ) Sim ( ) Não

**C)** Teve grupo controle?

( ) Sim ( ) Não

**O)** O estudo apresenta valores de marcadores inflamatórios (citocinas e outros marcadores) antes e após as intervenções de creatina?

( ) Sim ( ) Não

**S)** O estudo é um ensaio clínico randomizado e controlado?

( ) Sim ( ) Não

**Artigo 90: Voluntary Exercise Attenuates Hyperhomocysteinemia, But Does not Protect Against Hyperhomocysteinemia-Induced Testicular and Epididymal Disturbances**

dos Santos, DP, Ribeiro, DF, Frigoli, GF, Erthal, RP, Scarton, SRD, Siervo, GEMD, Seiva, FRF, Staurengo-Ferrari, L, Verri, WA, Deminice, R, Fernandes, GSA

**P)** Os participantes possuem mais de 18 anos de idade?

( ) Sim (X) Não

The study explicitly states in the "Materials and Methods" section that "The animals (PND28) were divided by simple randomization into four experimental groups". Furthermore, the title of the article clarifies that the study was conducted "in Swiss mice". This indicates that the participants were animals, not humans over 18 years of age.

**I)** A intervenção utilizada no estudo é creatina? Vale qualquer formato de administração

( ) Sim ( ) Não

**C)** Teve grupo controle?

( ) Sim ( ) Não

**O)** O estudo apresenta valores de marcadores inflamatórios (citocinas e outros marcadores) antes e após as intervenções de creatina?

( ) Sim ( ) Não

**S)** O estudo é um ensaio clínico randomizado e controlado?

( ) Sim ( ) Não

**Artigo 91: Modulation of choline and lactate metabolism by basic fibroblast growth factor mitigates neuroinflammation in type 2 diabetes: Insights from 1H-NMR metabolomics analysis**

Wu, YL, Wang, XY, Zhang, WL, Fu, J, Jiang, KD, Shen, YY, Li, C, Gao, HC

**P)** Os participantes possuem mais de 18 anos de idade?

( ) Sim (X) Não

The study states under "Methods" that "**db/db mice were employed as an in vivo model**". This indicates that the participants were animals, not human subjects over 18 years of age.

**I)** A intervenção utilizada no estudo é creatina? Vale qualquer formato de administração

( ) Sim ( ) Não

**C)** Teve grupo controle?

( ) Sim ( ) Não

**O)** O estudo apresenta valores de marcadores inflamatórios (citocinas e outros marcadores) antes e após as intervenções de creatina?

( ) Sim ( ) Não

**S)** O estudo é um ensaio clínico randomizado e controlado?

( ) Sim ( ) Não

**Artigo 92: Anti-inflammatory activity of creatine supplementation in endothelial**cellsin vitro

Nomura, A, Zhang, MJ, Sakamoto, T, Ishii, Y, Morishima, Y, Mochizuki, M, Kimura, T, Uchida, Y, Sekizawa, K

**P)** Os participantes possuem mais de 18 anos de idade?

( ) Sim (X) Não

The study explicitly states in its abstract and methods that it "investigated whether CR supplementation had any anti-inflammatory activity against **human pulmonary endothelial cells in culture**". This indicates that the participants were cells in a laboratory setting, not human subjects over 18 years of age.

**I)** A intervenção utilizada no estudo é creatina? Vale qualquer formato de administração

( ) Sim ( ) Não

**C)** Teve grupo controle?

( ) Sim ( ) Não

**O)** O estudo apresenta valores de marcadores inflamatórios (citocinas e outros marcadores) antes e após as intervenções de creatina?

( ) Sim ( ) Não

**S)** O estudo é um ensaio clínico randomizado e controlado?

( ) Sim ( ) Não

**Artigo 93: Exercise-induced muscle damage: mechanism, assessment and nutritional factors to accelerate recovery**

Markus, I, Constantini, K, Hoffman, JR, Bartolomei, S, Gepner, Y

**P)** Os participantes possuem mais de 18 anos de idade?

(X) Sim ( ) Não

The article is a review focusing on "Exercise-induced muscle damage" in "recreational and competitive athletes". It specifically discusses "sex differences, as well as differences between younger and older individuals", and refers to studies conducted on "untrained individuals", "competitive marathoners", and "young and middle-aged trained individuals". This indicates that the subjects of the research being reviewed are humans, including adults over 18 years of age.

**I)** A intervenção utilizada no estudo é creatina? Vale qualquer formato de administração

( ) Sim (X) Não

This document is clearly identified as a "REVIEW ARTICLE". Its purpose is to "briefly and comprehensively summarize many of these strategies that have been shown to positively influence the recovery process after damaging exercise". While it includes a section titled "Creatine" and discusses its potential benefits and mechanisms, this article itself does not conduct an experiment or utilize creatine as an intervention; rather, it reviews existing studies that have used creatine as an intervention.

**C)** Teve grupo controle?

( ) Sim ( ) Não

**O)** O estudo apresenta valores de marcadores inflamatórios (citocinas e outros marcadores) antes e após as intervenções de creatina?

( ) Sim ( ) Não

**S)** O estudo é um ensaio clínico randomizado e controlado?

( ) Sim ( ) Não

**Artigo 94: Functional Properties of Meat in Athletes' Performance and Recovery**

**P)** Os participantes possuem mais de 18 anos de idade?

(X) Sim ( ) Não

The article is explicitly identified as a "**Review**" article, which synthesizes existing literature. It focuses on the "Functional Properties of Meat in **Athletes’ Performance and Recovery**". The text refers to the "sportsman’s diet" and discusses nutritional strategies for "athletes". Given that "athletes" typically include individuals over 18 years of age, and previous reviews on athletes have included participants over 18 (e.g., Article 93 states it examines EIMD in "recreational and competitive athletes" and mentions "younger and older individuals"), the underlying research reviewed would involve participants over 18 years of age.

**I)** A intervenção utilizada no estudo é creatina? Vale qualquer formato de administração

( ) Sim (X) Não

This document is a "**Review**" article. It does not conduct an original experiment or implement an intervention. Instead, it "provides an integrated overview of the functional properties of meat molecules and the benefits of meat consumption in athletes’ nutrition". While it dedicates a section to "Creatine" and discusses its effectiveness in ameliorating EIMD based on other studies ("Creatine effectiveness, in terms of EIMDs effects amelioration, has been widely demonstrated in several studies"), this article itself does not *use* creatine as an intervention.

**C)** Teve grupo controle?

( ) Sim ( ) Não

**O)** O estudo apresenta valores de marcadores inflamatórios (citocinas e outros marcadores) antes e após as intervenções de creatina?

( ) Sim ( ) Não

**S)** O estudo é um ensaio clínico randomizado e controlado?

( ) Sim ( ) Não

**Artigo 95: Hypermethylation: Causes and Consequences in Skeletal Muscle Myopathy**

Majumder, A, Behera, J, Jeremic, N, Tyagi, SC

**P)** Os participantes possuem mais de 18 anos de idade?

(X) Sim ( ) Não

The article is a "REVIEW ARTICLE" that synthesizes existing literature. It references studies involving "the elderly" and discusses "age-related sarcopenia", which implies participants are over 18 years of age.

**I)** A intervenção utilizada no estudo é creatina? Vale qualquer formato de administração

( ) Sim (X) Não

The article is a "REVIEW ARTICLE" and, as such, it does not conduct an original intervention. Instead, it discusses the role of various factors and interventions, including mentioning creatine in the context of methionine metabolism and citing studies on "creatine supplementation".

**C)** Teve grupo controle?

( ) Sim ( ) Não

**O)** O estudo apresenta valores de marcadores inflamatórios (citocinas e outros marcadores) antes e após as intervenções de creatina?

( ) Sim ( ) Não

**S)** O estudo é um ensaio clínico randomizado e controlado?

( ) Sim ( ) Não

**Artigo 96: How to Increase Muscle Mass in Critically Ill Patients: Lessons Learned from Athletes and Bodybuilders**

Gala, K, Desai, V, Liu, NL, Omer, EM, McClave, SA

**P)** Os participantes possuem mais de 18 anos de idade?

( ) Sim ( ) Não

**I)** A intervenção utilizada no estudo é creatina? Vale qualquer formato de administração

( ) Sim ( ) Não

**C)** Teve grupo controle?

( ) Sim ( ) Não

**O)** O estudo apresenta valores de marcadores inflamatórios (citocinas e outros marcadores) antes e após as intervenções de creatina?

( ) Sim ( ) Não

**S)** O estudo é um ensaio clínico randomizado e controlado?

( ) Sim ( ) Não

**Artigo 97: Preventive effects of indole-3-carbinol against alcohol-induced liver injury in**mice**via antioxidant, anti-inflammatory, and anti-apoptotic mechanisms: Role of gut-liver-adipose tissue axis**

Choi, Y, Abdelmegeed, MA, Song, BJ

**P)** Os participantes possuem mais de 18 anos de idade?

( ) Sim (X) Não

O estudo utilizou **camundongos C57BL/6J machos de 8 a 10 semanas de idade** como participantes. Sendo assim, os participantes não são humanos e, portanto, não possuem mais de 18 anos.

**I)** A intervenção utilizada no estudo é creatina? Vale qualquer formato de administração

( ) Sim ( ) Não

**C)** Teve grupo controle?

( ) Sim ( ) Não

**O)** O estudo apresenta valores de marcadores inflamatórios (citocinas e outros marcadores) antes e após as intervenções de creatina?

( ) Sim ( ) Não

**S)** O estudo é um ensaio clínico randomizado e controlado?

( ) Sim ( ) Não

**Artigo 98: Sarcopenia in Nursing Home Residents**

Bauer, JM, Kaiser, MJ, Sieber, CC

**P)** Os participantes possuem mais de 18 anos de idade?

(X) Sim ( ) Não

The article is a "REVIEW" article focusing on sarcopenia in "nursing home residents". It explicitly discusses "the age-associated loss of muscle mass and muscle strength" and refers to "older adults" and "elderly" populations, which inherently means the participants of the studies reviewed are over 18 years of age.

**I)** A intervenção utilizada no estudo é creatina? Vale qualquer formato de administração

( ) Sim (X) Não

This is a "REVIEW" article and therefore does not conduct its own intervention. While it mentions that "There are some evidences that **creatine supplementation together with exercise has a synergistic effect on muscle strength in elderly people**" (referencing other studies not provided in this specific excerpt), the study itself does not *utilize* creatine as an experimental intervention. Instead, it discusses various "Treatment options in sarcopenic nursing home inhabitants" which "comprise physical training, modifications of nutritional intake, and pharmacological substances".

**C)** Teve grupo controle?

( ) Sim ( ) Não

**O)** O estudo apresenta valores de marcadores inflamatórios (citocinas e outros marcadores) antes e após as intervenções de creatina?

( ) Sim ( ) Não

**S)** O estudo é um ensaio clínico randomizado e controlado?

( ) Sim ( ) Não

**Artigo 99: Effects of dietary Docosahexaenoic, training and acute exercise on lipid mediators**

Capó, X, Martorell, M, Sureda, A, Tur, JA, Pons, A

**P)** Os participantes possuem mais de 18 anos de idade?

(X) Sim ( ) Não

"All subjects gave their **written informed consent** after an explanation of the experimental procedures and before commencement of the study. The study protocol was in accordance with the Declaration of Helsinki for research on human subjects and was approved by the Ethical Committee of Clinical Investigation of the CAIB (Palma de Mallorca, Balearic Islands, Spain)." Obtaining written informed consent from subjects typically indicates they are adults. The study involves "male soccer players from the Real Mallorca B team", who are generally adult or late-teenage athletes.

**I)** A intervenção utilizada no estudo é creatina? Vale qualquer formato de administração

( ) Sim (X) Não

The primary intervention described is "**docosahexaenoic (DHA) supplementation**". The experimental group "consumed **DHA-enriched beverage (1.16 g DHA/day)** for 8 weeks". Creatine is mentioned in the references, but it is not the intervention used in this study.

**C)** Teve grupo controle?

( ) Sim ( ) Não

**O)** O estudo apresenta valores de marcadores inflamatórios (citocinas e outros marcadores) antes e após as intervenções de creatina?

( ) Sim ( ) Não

**S)** O estudo é um ensaio clínico randomizado e controlado?

( ) Sim ( ) Não

**Artigo 100: Nutritional Support for Exercise-Induced Injuries**

Tipton, KD

**P)** Os participantes possuem mais de 18 anos de idade?

(X) Sim ( ) Não

The document is explicitly labeled as a "REVIEW ARTICLE". Review articles do not involve direct participants in a study. However, the review focuses on "Nutritional Support for Exercise-Induced Injuries" in contexts like "athletes and exercisers", "young, healthy individuals", and discusses topics such as "muscle growth", "acute, traumatic injuries", and "resistance exercise-induced muscle hypertrophy". These terms imply that the populations studied in the reviewed literature would consist of individuals over 18 years of age.

**I)** A intervenção utilizada no estudo é creatina? Vale qualquer formato de administração

( ) Sim (X) Não

This is a "REVIEW ARTICLE" and as such, it does not conduct its own experimental intervention. Instead, it "critically evaluate[s] information – from studies directly measuring the response to injury as well as information from other models – concerning nutritional interventions applicable to exercise-induced injuries". Creatine supplementation is discussed as a "potential nutritional countermeasure to muscle loss", with the article summarizing findings from other studies (e.g., " Creatine supplementation during 2 weeks of lower-limb casting did not attenuate muscle loss in otherwise healthy volunteers or in patients following total knee arthroplasty. However, more recently, loss of muscle in arms immobilized for 7 days was ameliorated with creatine supplementation"). This indicates that the article *discusses* creatine, but does not *use* it as an intervention within its own scope.

**C)** Teve grupo controle?

( ) Sim ( ) Não

**O)** O estudo apresenta valores de marcadores inflamatórios (citocinas e outros marcadores) antes e após as intervenções de creatina?

( ) Sim ( ) Não

**S)** O estudo é um ensaio clínico randomizado e controlado?

( ) Sim ( ) Não

**Artigo 101: Benefits of L-Arginine on Cardiovascular System**

Sudar-Milovanovic, E, Obradovic, M, Jovanovic, A, Zaric, B, Zafirovic, S, Panic, A, Radak, D, Isenovic, ER

**P)** Os participantes possuem mais de 18 anos de idade?

(X) Sim ( ) Não

This is a "mini review" that summarizes "the latest scientific studies related to L-Arg and its mechanisms of action". The review synthesizes findings from "Clinical studies on hypertensive and diabetic patients, and on healthy individuals", as well as "Evidence from Human Studies". These human studies involve populations such as "seven patients with chronic heart failure", "12 patients with CHF", "patients with coronary artery disease (CAD)", "54 hypertensive or normotensive patients", "young hypercholesterolemic subjects", and "healthy old participants". While one case study mentioned involved a "16-year-old male patient", the vast majority of studies referenced and the general population discussed are adults.

**I)** A intervenção utilizada no estudo é creatina? Vale qualquer formato de administração

( ) Sim (X) Não

The title of the article is "**Benefits of L-Arginine on Cardiovascular System**". The review explicitly states its purpose is to summarize "the latest scientific studies related to **L-Arg** and its mechanisms of action". Throughout the "Evidence from Human Studies" section, interventions discussed are primarily "**oral administration of L-Arg**" or "**IV infusion of L-Arg**". Creatine is not mentioned as an intervention in this review.

**C)** Teve grupo controle?

( ) Sim ( ) Não

**O)** O estudo apresenta valores de marcadores inflamatórios (citocinas e outros marcadores) antes e após as intervenções de creatina?

( ) Sim ( ) Não

**S)** O estudo é um ensaio clínico randomizado e controlado?

( ) Sim ( ) Não

**Artigo 102: Dietary supplementation with multiple micronutrients: No beneficial effects in pediatric cystic fibrosis patients**

Oudshoorn, JH, Klijn, PHC, Hofman, Z, Voorbij, HAM, van der Ent, CK, Berger, R, Houwen, RHJ

**P)** Os participantes possuem mais de 18 anos de idade?

( ) Sim (X) Não

The study explicitly states the age of the participants multiple times. For instance, the abstract mentions "22 CF patients (**12.9±2.5 yrs**)". The methods section further clarifies the age range: "Thirteen boys and sixteen girls with CF **aged 9.8– 18.9 years (mean 13.3 yrs)**" and "Included were **children aged 9 to 18 years**". Finally, it is stated that "All patients were **adolescents**". This clearly indicates that the participants were predominantly under 18 years of age.

**I)** A intervenção utilizada no estudo é creatina? Vale qualquer formato de administração

( ) Sim ( ) Não

**C)** Teve grupo controle?

( ) Sim ( ) Não

**O)** O estudo apresenta valores de marcadores inflamatórios (citocinas e outros marcadores) antes e após as intervenções de creatina?

( ) Sim ( ) Não

**S)** O estudo é um ensaio clínico randomizado e controlado?

( ) Sim ( ) Não

**Artigo 103: Undernutrition in Patients with COPD and Its Treatment**

Itoh, M, Tsuji, T, Nemoto, K, Nakamura, H, Aoshiba, K

**P)** Os participantes possuem mais de 18 anos de idade?

(X) Sim ( ) Não

This document is a "Review" article, meaning it synthesizes existing research rather than conducting its own experiment with direct participants. However, the review focuses on "Undernutrition in **Patients with COPD**". Chronic obstructive pulmonary disease (COPD) is described as "a chronic inflammatory disorder of the lung and whole body caused mainly by tobacco smoking". The studies reviewed involve "hypertensive and diabetic patients, and on healthy individuals" or "patients with chronic heart failure (CHF)". For instance, a referenced study details a "RCT in which rhGH (0.15 IU/kg) was administered for three weeks during a two-month course of respiratory rehabilitation in COPD patients". Another mentions "120 patients with COPD". These patient populations, especially those with COPD often linked to tobacco smoking, are inherently adult populations, thus indicating the participants in the reviewed studies were **predominantly over 18 years of age**.

**I)** A intervenção utilizada no estudo é creatina? Vale qualquer formato de administração

( ) Sim (X) Não

This is a "Review" article, and as such, it does not *utilize* any intervention itself. Instead, it "briefly reviews the recent papers dealing with COPD and their nutritional supplement therapies". While the article *discusses* "Creatine supplementation" as a potential nutritional therapy in the context of COPD, it is referring to interventions conducted in other studies (e.g., "Randomized controlled trial of dietary creatine as an adjunct therapy to physical training in chronic obstructive pulmonary disease"). Therefore, creatine is a topic of discussion and review, not an intervention used by the authors of this specific article.

**C)** Teve grupo controle?

( ) Sim ( ) Não

**O)** O estudo apresenta valores de marcadores inflamatórios (citocinas e outros marcadores) antes e após as intervenções de creatina?

( ) Sim ( ) Não

**S)** O estudo é um ensaio clínico randomizado e controlado?

( ) Sim ( ) Não

**Artigo 104: Mitochondrial therapy for Parkinson's disease: Neuroprotective pharmaconutrition may be disease-modifying**

Kones, R

**P)** Os participantes possuem mais de 18 anos de idade?

(X) Sim ( ) Não

"This document is a 'Review' article focusing on 'Mitochondrial therapy for Parkinson’s disease'. Parkinson's disease is an adult-onset neurodegenerative disorder. The review refers to 'two large Phase III trials involving CoQ10 and creatine', which typically enroll adult participants. The discussion of the disease and its treatments implicitly refers to an adult population, and no mention of pediatric patients or children is made."

**I)** A intervenção utilizada no estudo é creatina? Vale qualquer formato de administração

( ) Sim (X) Não

"This document is a 'Review' article and therefore does not itself utilize any intervention. While the article discusses and references 'two large Phase III trials involving CoQ10 and creatine' in the context of Parkinson's disease treatment, creatine is a topic of discussion and review, not an intervention used by the authors of this specific review."

**C)** Teve grupo controle?

( ) Sim ( ) Não

**O)** O estudo apresenta valores de marcadores inflamatórios (citocinas e outros marcadores) antes e após as intervenções de creatina?

( ) Sim ( ) Não

**S)** O estudo é um ensaio clínico randomizado e controlado?

( ) Sim ( ) Não

**Artigo 105: The Key Role of Nutritional Elements on Sport Rehabilitation and the Effects of Nutrients Intake**

Papadopoulou, SK, Mantzorou, M, Kondyli-Sarika, F, Alexandropoulou, I, Papathanasiou, J, Voulgaridou, G, Nikolaidis, PT

**P)** Os participantes possuem mais de 18 anos de idade?

(X) Sim ( ) Não

"This document is a 'Review' article focusing on 'Nutritional Elements on Sport Rehabilitation and the Effects of Nutrients Intake' for 'athletes'. While it mentions "world youth championship (aged 16–17 years), and one world junior championship (16–19 years)" when discussing injury incidence, the overall context of the review discusses the needs of 'athletes' in general, rehabilitation after surgery or injury, and refers to concepts such as 'sarcopenic symptomatology' which is an age-related condition. The review also explicitly references studies conducted on 'older adults' and 'elderly men', indicating that the populations covered in the reviewed literature include and are predominantly **over 18 years of age**."

**I)** A intervenção utilizada no estudo é creatina? Vale qualquer formato de administração

( ) Sim (X) Não

"This document is a 'Review' article and therefore **does not itself utilize any intervention**. Instead, it synthesizes existing research on various nutritional elements. While the article discusses creatine as one of the 'Other supplements suggested for muscle damage treatment and protein synthesis', it is referring to interventions conducted in other studies (e.g., 'Effect of Oral Creatine Supplementation on Human Muscle GLUT4 Protein Content after Immobilization'). Therefore, creatine is a topic of discussion and review within this article, not an intervention used by the authors of this specific publication."

**C)** Teve grupo controle?

( ) Sim ( ) Não

**O)** O estudo apresenta valores de marcadores inflamatórios (citocinas e outros marcadores) antes e após as intervenções de creatina?

( ) Sim ( ) Não

**S)** O estudo é um ensaio clínico randomizado e controlado?

( ) Sim ( ) Não

**Artigo 106: Nutritional and Supplementation Strategies to Prevent and Attenuate Exercise-Induced Muscle Damage: a Brief Review**

Harty, PS, Cottet, ML, Malloy, JK, Kerksick, CM

**P)** Os participantes possuem mais de 18 anos de idade?

(X) Sim ( ) Não

"This is a 'Review Article' which synthesizes findings from various studies. The review itself does not have participants, but the studies it discusses include participants who are **over 18 years of age**. For example, it mentions that reducing symptoms of EIMD 'may improve exercise compliance in untrained individuals or older adults'. Reference specifically points to a study on 'old and young subjects'. Furthermore, the review discusses various studies conducted on 'males' and 'untrained males', which generally refer to adult populations."

**I)** A intervenção utilizada no estudo é creatina? Vale qualquer formato de administração

( ) Sim (X) Não

"This document is a 'Review Article' and, as such, **does not itself utilize any intervention**. Its purpose is to summarize and discuss existing research on various nutritional and supplementation strategies for exercise-induced muscle damage. The article extensively discusses creatine as one of these strategies, with a dedicated section titled 'Creatine', detailing its effects and referencing studies that used creatine supplementation."

**C)** Teve grupo controle?

( ) Sim ( ) Não

**O)** O estudo apresenta valores de marcadores inflamatórios (citocinas e outros marcadores) antes e após as intervenções de creatina?

( ) Sim ( ) Não

**S)** O estudo é um ensaio clínico randomizado e controlado?

( ) Sim ( ) Não

**Artigo 107: Age-related muscle anabolic resistance: inevitable or preventable?**

Aragon, AA, Tipton, KD, Schoenfeld, BJ

**P)** Os participantes possuem mais de 18 anos de idade?

(X) Sim ( ) Não

"This document is a 'Narrative Review' focusing on age-related muscle anabolic resistance. While it does not have its own participants, it synthesizes research from studies involving various age groups. The review explicitly states: '**The age range of the young participants was 20 years–35 years, whereas the older adults studied were 64–76 years.**' This confirms that the populations discussed in the reviewed literature are predominantly over 18 years of age."

**I)** A intervenção utilizada no estudo é creatina? Vale qualquer formato de administração

( ) Sim (X) Não

"This document is a '**Narrative Review**' and therefore **does not itself utilize any intervention**. Its purpose is to broadly and comprehensively cover the nuances of age-related anabolic resistance by synthesizing existing literature. While the review discusses creatine as one of the 'agents for this purpose that have been prolifically investigated', this refers to interventions performed in other studies that are being reviewed, not by the authors of this specific publication."

**C)** Teve grupo controle?

( ) Sim ( ) Não

**O)** O estudo apresenta valores de marcadores inflamatórios (citocinas e outros marcadores) antes e após as intervenções de creatina?

( ) Sim ( ) Não

**S)** O estudo é um ensaio clínico randomizado e controlado?

( ) Sim ( ) Não

**Artigo 108: Dysfunctional energy metabolisms in fibromyalgia**compared with**healthy subjects**

Jung, YH, Kim, H, Lee, D, Lee, JY, Moon, JY, Choi, SH, Kang, D

**P)** Os participantes possuem mais de 18 anos de idade?

(X) Sim ( ) Não

"The inclusion criteria for FM subjects were as follows: diagnosed with FM; **between 21 and 63 years of age**". A tabela de características dos participantes também indica uma "Age 41.7 14.0" para o grupo FM e "40.1 6.3" para o grupo controle.

**I)** A intervenção utilizada no estudo é creatina? Vale qualquer formato de administração

( ) Sim (X) Não

"This study aimed to **investigate the levels of creatine (Cr) metabolites** in the anterior cingulate cortex (ACC), thalamus, and insula of patients with fibromyalgia (FM) using proton magnetic resonance spectroscopy (MRS)". O estudo está medindo os níveis existentes de metabólitos de creatina, e não administrando creatina como uma intervenção.

**C)** Teve grupo controle?

( ) Sim ( ) Não

**O)** O estudo apresenta valores de marcadores inflamatórios (citocinas e outros marcadores) antes e após as intervenções de creatina?

( ) Sim ( ) Não

**S)** O estudo é um ensaio clínico randomizado e controlado?

( ) Sim ( ) Não

**Artigo 109: Targeting Mitochondria and Oxidative Stress in Cancer- and Chemotherapy-Induced Muscle Wasting**

Huot, JR, Baumfalk, D, Resendiz, A, Bonetto, A, Smuder, AJ, Penna, F

**P)** Os participantes possuem mais de 18 anos de idade?

(X) Sim ( ) Não

"This document is a 'FORUM REVIEW ARTICLE' that synthesizes research. It discusses findings from both 'preclinical and clinical trials'. The clinical trials reviewed, which involve 'cancer patients' and focus on 'chemotherapy-induced muscle wasting', inherently involve adult participants. For example, it cites a 'Randomized controlled trial evaluating the efficacy and safety of vitamin E supplementation for protection against cisplatin-induced peripheral neuropathy', which typically includes adult subjects."

**I)** A intervenção utilizada no estudo é creatina? Vale qualquer formato de administração

( ) Sim (X) Não

"This document is explicitly identified as a '**FORUM REVIEW ARTICLE**'. As a review, it **does not conduct its own interventions**. Instead, it discusses various 'approaches [that] have been tested to modulate the redox homeostasis... from exercise training to distinct classes of direct or indirect antioxidants'. Creatine is mentioned as one such agent that has been investigated in other studies (e.g., 'Creatine supplementation reduces plasma levels of pro-inflammatory cytokines'), but it is not an intervention performed by the authors of this specific article."

**C)** Teve grupo controle?

( ) Sim ( ) Não

**O)** O estudo apresenta valores de marcadores inflamatórios (citocinas e outros marcadores) antes e após as intervenções de creatina?

( ) Sim ( ) Não

**S)** O estudo é um ensaio clínico randomizado e controlado?

( ) Sim ( ) Não

**Artigo 110: Dietary Supplements for Health, Adaptation, and Recovery in Athletes**

Rawson, ES, Miles, MP, Larson-Meyer, DE

**P)** Os participantes possuem mais de 18 anos de idade?

(X) Sim ( ) Não

"This document is a 'SCHOLARLY REVIEW' and, as such, does not have its own participants performing an intervention. Instead, it synthesizes findings from various studies. The review discusses potential benefits of supplementation for 'athletes' who are generally adults. Furthermore, it explicitly mentions that creatine may improve 'cognitive processing' and 'reduce severity of or enhance recovery from mild traumatic brain injury (mTBI)'. The review also cites studies on 'cognitive performance in elderly individuals', confirming that the populations discussed are predominantly over 18 years of age."

**I)** A intervenção utilizada no estudo é creatina? Vale qualquer formato de administração

( ) Sim (X) Não

"This document is a 'SCHOLARLY REVIEW' and therefore **does not itself utilize any intervention**. Its purpose is to 'describe several dietary supplements that may improve health, exercise adaptation, or recovery'. While it discusses creatine as a supplement that 'may improve recovery from and adaptation to intense training' and lists it among 'dietary supplements for health, adaptation, and recovery in athletes', it is summarizing findings from other research, not conducting its own administration of creatine."

**C)** Teve grupo controle?

( ) Sim ( ) Não

**O)** O estudo apresenta valores de marcadores inflamatórios (citocinas e outros marcadores) antes e após as intervenções de creatina?

( ) Sim ( ) Não

**S)** O estudo é um ensaio clínico randomizado e controlado?

( ) Sim ( ) Não

**Artigo 111: Nutritional strategies for maintaining muscle mass and strength from middle age to later life: A narrative review**

Cruz-Jentoft, AJ, Hughes, BD, Scott, D, Sanders, KM, Rizzoli, R

**P)** Os participantes possuem mais de 18 anos de idade?

(X) Sim ( ) Não

"This narrative review summarizes evidence of nutritional interventions for maintaining muscle mass and strength **from midlife through old age**". Furthermore, the review discusses findings in "**middle-aged and older adults**" and cites systematic reviews of studies in "**adults ≥65 years of age**"

**I)** A intervenção utilizada no estudo é creatina? Vale qualquer formato de administração

( ) Sim (X) Não

"This document is identified as a '**narrative review**'. Its stated purpose is to 'summarize evidence of nutritional interventions' by conducting 'Searches... of PubMed and Cochrane databases'. Therefore, the authors of this article did not conduct an intervention using creatine themselves; rather, they **reviewed other studies that investigated creatine supplementation**".

**C)** Teve grupo controle?

( ) Sim (X) Não

"As a '**narrative review**', this study did not conduct an original experimental intervention and therefore **did not have its own control group**. Instead, its methodology involved synthesizing 'evidence from various studies', many of which were explicitly identified as 'randomized, controlled trials (RCTs)'."

**O)** O estudo apresenta valores de marcadores inflamatórios (citocinas e outros marcadores) antes e após as intervenções de creatina?

( ) Sim ( ) Não

**S)** O estudo é um ensaio clínico randomizado e controlado?

( ) Sim ( ) Não

**Artigo 112: Urinary type II collagen neoepitope as an outcome measure for relapsing polychondritis**

Kraus, VB, Stabler, T, Le, ET, Saltarelli, M, Allen, NB

**P)** Os participantes possuem mais de 18 anos de idade?

(X) Sim ( ) Não

"Herein we describe the case of a man who was diagnosed as having relapsing polychondritis (RP) when he was **18 years of age** and was treated over the course of 2 years with numerous immunosuppressive agents".

**I)** A intervenção utilizada no estudo é creatina? Vale qualquer formato de administração

( ) Sim (X) Não

"The study describes the case of a man treated with 'numerous immunosuppressive agents, including tumor necrosis factor (TNF) inhibitors' and 'Upon initiation of etanercept' to manage relapsing polychondritis". **Creatine was not an intervention**; it is only mentioned in the acknowledgments for providing "expert information on the hazards of creatine".

**C)** Teve grupo controle?

( ) Sim ( ) Não

**O)** O estudo apresenta valores de marcadores inflamatórios (citocinas e outros marcadores) antes e após as intervenções de creatina?

( ) Sim ( ) Não

**S)** O estudo é um ensaio clínico randomizado e controlado?

( ) Sim ( ) Não

**Artigo 113: Efficacy of treatments for Demodex blepharitis: A**systematic review**and**meta-analysis

Navel, V, Mulliez, A, d'Azy, CB, Baker, JS, Malecaze, J, Chiambaretta, F, Dutheil, F

**P)** Os participantes possuem mais de 18 anos de idade?

(X) Sim ( ) Não

"All studies included adults (> 18 years old) [14–16,18–27,29–32,46], except one study without age criteria."

**I)** A intervenção utilizada no estudo é creatina? Vale qualquer formato de administração

( ) Sim (X) Não

"We conducted a systematic review and meta-analysis to evaluate the efficacy of different treatment for Demodex blepharitis." The article explicitly lists the treatments used in the reviewed studies, such as "50% TTO," "pilocarpine gel," "usual lid hygiene," "systemic ivermectin," and "systemic metronidazole," but **creatine is not mentioned as an intervention**

**C)** Teve grupo controle?

( ) Sim ( ) Não

**O)** O estudo apresenta valores de marcadores inflamatórios (citocinas e outros marcadores) antes e após as intervenções de creatina?

( ) Sim ( ) Não

**S)** O estudo é um ensaio clínico randomizado e controlado?

( ) Sim ( ) Não

**Artigo 114: Oxidative and antioxidative stress markers in dry eye disease: A**systematic review**and**meta-analysis

Navel, V, Sapin, V, Henrioux, F, Blanchon, L, Labbé, A, Chiambaretta, F, Baudouin, C, Dutheil, F

**P)** Os participantes possuem mais de 18 anos de idade?

(X) Sim ( ) Não

"While the review discusses age as a relevant factor, stating that 'DED is a multifactorial diseases associated with age' and that 'the prevalence of DED increases in the elderly population', it also notes that it collected 'mean age' data from the included studies. The explicit exclusion of 'animal studies' implies human participants, and the discussion of age as a covariate for analysis indicates that adults, including older adults, were part of the population reviewed."

**I)** A intervenção utilizada no estudo é creatina? Vale qualquer formato de administração

( ) Sim ( ) Não

"The stated purpose of this study is 'To conduct a systematic review and meta-analysis on the levels of oxidative stress markers and antioxidants in dry eye disease (DED) compared with healthy subject'. The review details various 'oxidative stress markers and antioxidants' and discusses treatments for dry eye disease, but **creatine is not mentioned anywhere as an intervention** or a focus of the reviewed treatments."

**C)** Teve grupo controle?

( ) Sim ( ) Não

**O)** O estudo apresenta valores de marcadores inflamatórios (citocinas e outros marcadores) antes e após as intervenções de creatina?

( ) Sim ( ) Não

**S)** O estudo é um ensaio clínico randomizado e controlado?

( ) Sim ( ) Não

**Artigo 115: Oxidative and antioxidative stress markers in keratoconus: a**systematic review**and**meta-analysis

Navel, V, Malecaze, J, Pereira, B, Baker, JS, Malecaze, F, Sapin, V, Chiambaretta, F, Dutheil, F

**P)** Os participantes possuem mais de 18 anos de idade?

(X) Sim ( ) Não

"The study is a systematic review and meta-analysis on keratoconus, a 'progressive and chronic corneal disease' that typically manifests in adolescence or early adulthood. The reviewed studies included a total of '1328 keratoconus patients and 1208 healthy controls'. While a specific minimum age of 18 years is not explicitly stated as an *exclusion criterion* for all included studies, the overall context of the disease, the collection and analysis of 'mean age' data from the cohorts, and the lack of specific focus on pediatric populations strongly suggest that the participants in the studies reviewed were predominantly, if not exclusively, adults."

**I)** A intervenção utilizada no estudo é creatina? Vale qualquer formato de administração

( ) Sim (X) Não

"The stated purpose of this systematic review and meta-analysis is 'To compare the level of oxidative stress markers and antioxidants in KC than healthy controls'. The article focuses on levels of various 'oxidative stress markers' and 'antioxidants' in different samples (tears, cornea, aqueous humour, blood). **Creatine is not mentioned as an intervention** or a focus of the reviewed treatments or modulators of oxidative stress."

**C)** Teve grupo controle?

( ) Sim ( ) Não

**O)** O estudo apresenta valores de marcadores inflamatórios (citocinas e outros marcadores) antes e após as intervenções de creatina?

( ) Sim ( ) Não

**S)** O estudo é um ensaio clínico randomizado e controlado?

( ) Sim ( ) Não

**Artigo 116: Prolonged feeding with guanidinoacetate, a methyl group consumer, exacerbates ethanol-induced liver injury**

Osna, NA, Feng, D, Ganesan, M, Maillacheruvu, PF, Orlicky, DJ, French, SW, Tuma, DJ, Kharbanda, KK

**P)** Os participantes possuem mais de 18 anos de idade?

( ) Sim (X) Não

"Adult male Wistar rats weighing 180 to 200 g purchased from Charles River Laboratories, Wilmington, MA) were weight-matched and divided into four groups." and "Male Wistar rats were fed the control or ethanol Lieber DeCarli diet with or without 0.36% GAA."

**I)** A intervenção utilizada no estudo é creatina? Vale qualquer formato de administração

( ) Sim ( ) Não

**C)** Teve grupo controle?

( ) Sim ( ) Não

**O)** O estudo apresenta valores de marcadores inflamatórios (citocinas e outros marcadores) antes e após as intervenções de creatina?

( ) Sim ( ) Não

**S)** O estudo é um ensaio clínico randomizado e controlado?

( ) Sim ( ) Não

**Artigo 117: The effect of Sarcomeal® oral supplementation plus vitamin D3 on muscle parameters and metabolic factors in diabetic sarcopenia patients: study protocol of a**randomized**controlled clinical**trial

Dezfouli, RA, Balajam, NZ, Shirazi, S, Heshmat, R, Shafiee, G

**P)** Os participantes possuem mais de 18 anos de idade?

(X) Sim ( ) Não

"Patients aged **50–75** with at least a history of six months of type 2 diabetes mellitus confirmed by a specialist doctor, a probable diagnosis of sarcopenia, and meeting the inclusion criteria will be evaluated for sarcopenia."

**I)** A intervenção utilizada no estudo é creatina? Vale qualquer formato de administração

(X) Sim ( ) Não

"This study is a protocol for evaluating the efficacy of the Sarcomeal® supplement, a mixture of whey protein, **creatine**, branch-chained amino acids (BCAAs), glutamine, and hydroxyl-methyl-butyrate (HMB) in diabetic people who also have sarcopenia." Additionally, Table 2 explicitly lists "Creatine Monohydrate (mg) 1500" as a component of Sarcomeal.

**C)** Teve grupo controle?

(X) Sim ( ) Não

"This study is a **randomized clinical trial, in which sixty diabetic sarcopenia patients who meet the inclusion criteria will be randomly assigned to the control or the intervention group** with a 1:1 allocation."

**O)** O estudo apresenta valores de marcadores inflamatórios (citocinas e outros marcadores) antes e após as intervenções de creatina?

(X) Sim ( ) Não

The study states its hypothesis is that the "Sarcomeal® supplement plus vitamin D may be beneficial for the management of diabetic sarcopenia by reducing inflammation, oxidative stress, and glucose metabolism." Under "Secondary outcomes" it lists "Inflammatory markers: HSRCRP (mg/L) Blood test". Furthermore, "Blood sampling and muscle parameters assessment will be carried out at the first and the last visit (week 12)", meaning measurements will be taken before and after the intervention which contains creatine.

**S)** O estudo é um ensaio clínico randomizado e controlado?

(X) Sim ( ) Não

The title of the article explicitly states "study protocol of a **randomized controlled clinical trial**". This is further confirmed in the methods section: "This study is a **randomized clinical trial**, in which sixty diabetic sarcopenia patients who meet the inclusion criteria will be randomly assigned to the control or the intervention group with a 1:1 allocation."

**Artigo 118: Integrative metabolomics highlights gut microbiota metabolites as novel NAFLD-related candidate biomarkers in children**

Luo, JY, Luo, MY, Kaminga, AC, Wei, J, Dai, W, Peng, YL, Zhao, KY, Duan, YM, Xiao, X, Ouyang, SS, Yao, ZZ, Liu, YX, Pan, XF

**P)** Os participantes possuem mais de 18 anos de idade?

( ) Sim (X) Não

"Integrative metabolomics highlights gut microbiota metabolites as novel NAFLD-related candidate biomarkers in **children**". The study explicitly states its focus on pediatric populations, with phrases like "**children with NAFLD**" and "**obese children controls**" used throughout the text.

**I)** A intervenção utilizada no estudo é creatina? Vale qualquer formato de administração

( ) Sim ( ) Não

**C)** Teve grupo controle?

( ) Sim ( ) Não

**O)** O estudo apresenta valores de marcadores inflamatórios (citocinas e outros marcadores) antes e após as intervenções de creatina?

( ) Sim ( ) Não

**S)** O estudo é um ensaio clínico randomizado e controlado?

( ) Sim ( ) Não

**Artigo 119: Effects of exercise and dietary epigallocatechin gallate and β-alanine on skeletal muscle in aged**mice

Pence, BD, Gibbons, TE, Bhattacharya, TK, Mach, H, Ossyra, JM, Petr, G, Martin, SA, Wang, L, Rubakhin, SS, Sweedler, JV, McCusker, RH, Kelley, KW, Rhodes, JS, Johnson, RW, Woods, JA

**P)** Os participantes possuem mais de 18 anos de idade?

( ) Sim (X) Não

"Male BALB/cByJ retired breeder mice (8–10 months old) were purchased from The Jackson Laboratory... and maintained until **17 months of age**. Mice were then randomized to treatments as described below and began the feeding portion of the study."

**I)** A intervenção utilizada no estudo é creatina? Vale qualquer formato de administração

( ) Sim ( ) Não

**C)** Teve grupo controle?

( ) Sim ( ) Não

**O)** O estudo apresenta valores de marcadores inflamatórios (citocinas e outros marcadores) antes e após as intervenções de creatina?

( ) Sim ( ) Não

**S)** O estudo é um ensaio clínico randomizado e controlado?

( ) Sim ( ) Não

**Artigo 120: Safety issues and harmful pharmacological interactions of nutritional supplements in Duchenne muscular dystrophy: considerations for Standard of Care and emerging virus outbreaks**

Boccanegra, B, Verhaart, IEC, Cappellari, O, Vroom, E, De Luca, A

**P)** Os participantes possuem mais de 18 anos de idade?

( ) Sim (X) Não

"Complex and chronic pathologies in the **paediatric population**, such as DMD, are at high risk of adverse drug reactions (ADR)". Furthermore, the article refers to the subjects as "**DMD boys**" and explicitly states that "there are no studies investigating the effect of Cr long-term administration in **children/adolescent**".

**I)** A intervenção utilizada no estudo é creatina? Vale qualquer formato de administração

( ) Sim ( ) Não

**C)** Teve grupo controle?

( ) Sim ( ) Não

**O)** O estudo apresenta valores de marcadores inflamatórios (citocinas e outros marcadores) antes e após as intervenções de creatina?

( ) Sim ( ) Não

**S)** O estudo é um ensaio clínico randomizado e controlado?

( ) Sim ( ) Não

**Artigo 121: Vitamin D3 Deficiency Differentially Affects Functional and Disease Outcomes in the G93A**Mouse**Model of Amyotrophic Lateral Sclerosis**

Solomon, JA, Gianforcaro, A, Hamadeh, MJ

**P)** Os participantes possuem mais de 18 anos de idade?

( ) Sim (X) Não

The study focuses on an animal model, explicitly stating: "**G93A Mouse Model** of Amyotrophic Lateral Sclerosis" in the title, and in the methods section: "At age 25 d, 102 **G93A mice** (56 M, 46 F) were divided into two vitamin D3 groups".

**I)** A intervenção utilizada no estudo é creatina? Vale qualquer formato de administração

( ) Sim ( ) Não

**C)** Teve grupo controle?

( ) Sim ( ) Não

**O)** O estudo apresenta valores de marcadores inflamatórios (citocinas e outros marcadores) antes e após as intervenções de creatina?

( ) Sim ( ) Não

**S)** O estudo é um ensaio clínico randomizado e controlado?

( ) Sim ( ) Não

**Artigo 122: The Impact of Step Reduction on Muscle Health in Aging: Protein and Exercise as Countermeasures**

Oikawa, SY, Holloway, TM, Phillips, SM

**P)** Os participantes possuem mais de 18 anos de idade?

(X) Sim ( ) Não

The study describes the participants as "healthy older adults"

**I)** A intervenção utilizada no estudo é creatina? Vale qualquer formato de administração

( ) Sim (X) Não

The intervention used was "supplementation with either a 60 g daily dose of whey protein or collagen peptides". Creatine was not the intervention in this study.

**C)** Teve grupo controle?

( ) Sim ( ) Não

**O)** O estudo apresenta valores de marcadores inflamatórios (citocinas e outros marcadores) antes e após as intervenções de creatina?

( ) Sim ( ) Não

**S)** O estudo é um ensaio clínico randomizado e controlado?

( ) Sim ( ) Não

**Artigo 123: Metabolic Perturbations from Step Reduction in Older Persons at Risk for Sarcopenia: Plasma Biomarkers of Abrupt Changes in Physical Activity**

Saoi, M, Li, A, McGlory, C, Stokes, T, von Allmen, MT, Phillips, SM, Britz-McKibbin, P

**P)** Os participantes possuem mais de 18 anos de idade?

(X) Sim ( ) Não

The study involved a "cohort of overweight, pre-diabetic older adults (age = 69 ± 4 years)".

**I)** A intervenção utilizada no estudo é creatina? Vale qualquer formato de administração

( ) Sim (X) Não

The intervention in this study was "two weeks of step reduction (<1000 steps/day) followed by a two week recovery period". Creatine was identified as one of the "circulatory metabolites" or "plasma biomarkers" that increased after step reduction, but it was not an intervention administered to the participants

**C)** Teve grupo controle?

( ) Sim ( ) Não

**O)** O estudo apresenta valores de marcadores inflamatórios (citocinas e outros marcadores) antes e após as intervenções de creatina?

( ) Sim ( ) Não

**S)** O estudo é um ensaio clínico randomizado e controlado?

( ) Sim ( ) Não

**Artigo 124: Mapping of Dietary Interventions Beneficial in the Prevention of Secondary Health Conditions in Spinal Cord Injured Population: A**Systematic Review

Stojic, S, Eriks-Hoogland, I, Gamba, M, Valido, E, Minder, B, Chatelan, A, Karagounis, LG, Ballesteros, M, Díaz, C, Brach, M, Stoyanov, J, Diviani, N, Rubinelli, S, Perret, C, Glisic, M

**P)** Os participantes possuem mais de 18 anos de idade?

(X) Sim ( ) Não

"Studies were included if they were clinical trials or observational studies conducted in **adult individuals** with SCI". Additionally, the mean age across 27 clinical trials included in the review ranged from "33 years (SD 15 years) and 57 years (SD 6.2 years)".

**I)** A intervenção utilizada no estudo é creatina? Vale qualquer formato de administração

(X) Sim ( ) Não

This study is a systematic review that *maps* or *identifies* dietary interventions. Creatine is explicitly mentioned as one of the dietary supplements found to be promising for preventing secondary health conditions in individuals with spinal cord injury. For example, the review states that "dietary supplementation including alpha-lipoic acid, **creatine**, vitamin D, and cranberry-derived supplements and probiotics were mapped as the most promising", and "Clinical trials studied the effectiveness of... dietary supplements: vitamin D, calcium, **creatine**, cranberry extract, omega-3 fatty acids, probiotics, and alpha-lipoic acid". Specific examples of trials supplementing "creatine (3 g/ day) and vitamin D (25000 IU every two weeks)" are also cited within the review

**C)** Teve grupo controle?

(X) Sim ( ) Não

The systematic review included "32 interventional (22 RCTs, 3 NRCT, and 7 pre-post studies)". The inclusion criteria specified that "any control group was considered eligible for inclusion (e.g., placebo, usual care, or other lifestyle intervention such as physical exercise)". Furthermore, specific trials involving creatine supplementation included "Placebo" groups.

**O)** O estudo apresenta valores de marcadores inflamatórios (citocinas e outros marcadores) antes e após as intervenções de creatina?

( ) Sim (X) Não

While the review generally discusses inflammation as a secondary health condition in spinal cord injury and mentions that other interventions, such as an "anti-inflammatory diet" or "alpha-lipoic acid," influenced inflammatory markers (e.g., IL-6, IL-1B, hs-CRP, TNF-alpha), **the specific findings reported for creatine supplementation within this systematic review focus on musculoskeletal health and physical performance outcomes (e.g., arm muscle area, seated medicine ball throw, one-repetition maximum test for chest press), and do not explicitly state that the reviewed studies on creatine interventions presented values of inflammatory markers before and after the intervention.**

**S)** O estudo é um ensaio clínico randomizado e controlado?

( ) Sim ( ) Não

**Artigo 125: International society of sports nutrition position stand: nutritional concerns of the female athlete**

Sims, ST, Kerksick, CM, Smith-Ryan, AE, de Jonge, XAKJ, Hirsch, KR, Arent, SM, Hewlings, SJ, Kleiner, SM, Bustillo, E, Tartar, JL, Starratt, VG, Kreider, RB, Greenwalt, C, Rentería, LI, Ormsbee, MJ, VanDusseldorp, TA, Campbell, BI, Kalman, DS, Antonio, J

**P)** Os participantes possuem mais de 18 anos de idade?

(X) Sim ( ) Não

The study is a position stand focusing on "**female athletes**" across their "lifespan," including "female athletes of reproductive age" and "peri and post-menopausal athletes". It also refers to research involving "older adults".

**I)** A intervenção utilizada no estudo é creatina? Vale qualquer formato de administração

(X) Sim ( ) Não

The position stand explicitly states, "**Creatine supplementation of 3 to 5 g per day is recommended for the mechanistic support of creatine supplementation**". It also mentions "Creatine monohydrate" as a supplement that "may improve recovery from and adaptation to intense training, recovery from periods of injury with extreme inactivity, cognitive processing, and reduce severity of or enhance recovery from mild traumatic brain injury (mTBI)".

**C)** Teve grupo controle?

(X) Sim ( ) Não

This document is a position stand that reviews existing literature. It explicitly references and draws conclusions from "**placebo-controlled clinical trials**" and "**randomized placebo-controlled trial**" when discussing various supplements, including creatine. The inclusion criteria for the reviewed literature prioritized studies with "scientific validity".

**O)** O estudo apresenta valores de marcadores inflamatórios (citocinas e outros marcadores) antes e após as intervenções de creatina?

( ) Sim (X) Não

This article is a "position stand" or "systematic review", which synthesizes findings from other studies rather than presenting its own raw "before and after" values for inflammatory markers. While it notes that creatine supplementation influences "inflammation", and refers to studies that **do** report such values (e.g., studies finding "creatine supplementation reduces plasma levels of pro-inflammatory cytokines"), Artigo 125 itself does not present these specific values.

**S)** O estudo é um ensaio clínico randomizado e controlado?

( ) Sim ( ) Não

**Artigo 126: Highlight article: Dietary protein and exercise for preservation of lean mass and perspectives on type 2 diabetes prevention**

de Sousa, MV, Soares, DBD, Caraça, ER, Cardoso, R

**P)** Os participantes possuem mais de 18 anos de idade?

(X) Sim ( ) Não

The review discusses nutritional strategies for "preventing muscle catabolism on the **elderly population**". It also refers to studies involving "**fragile elderly subjects**" and compiles "clinical trials... using the search terms: protein supplementation, sarcopenia, and **elderly**".

**I)** A intervenção utilizada no estudo é creatina? Vale qualquer formato de administração

(X) Sim ( ) Não

This article is a review that discusses various nutritional strategies. It explicitly cites studies on creatine supplementation. For instance, it mentions "**Creatine in type 2 diabetes: a randomized, double-blind, placebo-controlled trial**" and the "Impact of **creatine supplementation** in combination with resistance training on lean mass in the **elderly**".

**C)** Teve grupo controle?

(X) Sim ( ) Não

As a highlight article reviewing clinical trials, it refers to studies that employed control groups. For example, it cites a "**double-blind randomized design study**" and a "**randomized, double-blind, placebo-controlled trial**" involving creatine. The nature of the article, which compiles findings from "clinical trials", implies the inclusion of control groups in the studies reviewed.

**O)** O estudo apresenta valores de marcadores inflamatórios (citocinas e outros marcadores) antes e após as intervenções de creatina?

( ) Sim (X) Não

This article is a "**Minireview Highlight article**". Its purpose is to discuss and summarize existing research on dietary protein and exercise. It does not present original experimental data or its own raw "before and after" values for inflammatory markers specifically from creatine interventions. While it discusses inflammation as a factor in sarcopenia and type 2 diabetes, it synthesizes findings from other studies rather than conducting its own measurements.

**S)** O estudo é um ensaio clínico randomizado e controlado?

( ) Sim ( ) Não

**Artigo 127: Alcohol, microbiome, life style influence alcohol and non-alcoholic organ damage**

Neuman, MG, French, SW, Zakhari, S, Malnick, S, Seitz, HK, Cohen, LB, Salaspuro, M, Voinea-Griffin, A, Barasch, A, Kirpich, IA, Thomes, PG, Schrum, LW, Donohue, TM, Kharbanda, KK, Cruz, M, Opris, M

**P)** Os participantes possuem mais de 18 anos de idade?

(X) Sim ( ) Não

O artigo é uma revisão que integra "pre-clinical, translational and clinical research". Na seção que aborda as interações medicamentosas, são discutidas "incidences of adverse drug reactions with alcohol involvement in the emergency departments of the Unites States of America" (Castle and colleagues, 2016), onde se observou que "The visits involving alcohol-induced adverse drug reactions increased for males and females with ages 21 to 34 and females with ages over 55". Além disso, a revisão discute "The clinical aspects of NASH, as part of the metabolic syndrome in the aging population" e faz referência a estudos que incluem "over 10,000 subjects" e "over 4800 Chinese men" (Vu et al., 2016; Zhang et al., 2015) em Mendelian Randomization studies, os quais, por sua natureza, envolvem populações adultas.

**I)** A intervenção utilizada no estudo é creatina? Vale qualquer formato de administração

(X) Sim ( ) Não

O artigo possui uma seção dedicada à "Creatinine supplementation: does it prevent alcohol-induced liver injury?". Esta seção se refere a achados de Murali et al. (2016) sobre a "Creatine supplementation does not prevent the development of alcoholic steatosis". Além disso, discute a síntese hepática de creatina e o papel da creatina/fosfocreatina como um "marker protein".

**C)** Teve grupo controle?

(X) Sim ( ) Não

"The review synthesizes findings from various studies that utilized control groups. For instance, in discussions on autophagy, experiments compared 'ethanol non-metabolizing HepG2 cells' with 'recombinant VL-17A cells that metabolize ethanol' and measured 'LC3-II flux measurements in the presence and absence of lysosomal inhibitor bafilomycin'. Additionally, studies on dietary fat compared outcomes between 'USF + EtOH' and 'SF + EtOH' administration. The cited research on creatine also implies a comparison group, stating 'Creatine supplementation does not prevent the development of alcoholic steatosis'."

**O)** O estudo apresenta valores de marcadores inflamatórios (citocinas e outros marcadores) antes e após as intervenções de creatina?

( ) Sim (X) Não

"While the review extensively discusses inflammation in relation to alcohol and organ damage, it **does not present specific values of inflammatory markers (cytokines and other markers) before and after creatine interventions**. The section dedicated to creatine supplementation refers to studies evaluating its effect on alcoholic steatosis, but does not provide detailed inflammatory marker data."

**S)** O estudo é um ensaio clínico randomizado e controlado?

( ) Sim ( ) Não

**Artigo 128: Oral creatine-modified selenium-based hyaluronic acid nanogel mediated mitochondrial energy recovery to drive the treatment of inflammatory bowel disease.**

Huai M, Pei M, Chen J, Duan X, Zhu Y, Yang F, Ge W

**P)** Os participantes possuem mais de 18 anos de idade?

( ) Sim (X) Não

"The study includes an *in vivo* DSS-induced IBD model that utilized "All C57BL/6 mice, 6–8 weeks, male". Mice aged 6–8 weeks are not over 18 years old."

**I)** A intervenção utilizada no estudo é creatina? Vale qualquer formato de administração

( ) Sim ( ) Não

**C)** Teve grupo controle?

( ) Sim ( ) Não

**O)** O estudo apresenta valores de marcadores inflamatórios (citocinas e outros marcadores) antes e após as intervenções de creatina?

( ) Sim ( ) Não

**S)** O estudo é um ensaio clínico randomizado e controlado?

( ) Sim ( ) Não

**Artigo 129: Phosphocreatine ameliorates hepatocellular apoptosis mediated by protecting mitochondrial damage in liver ischemia/reperfusion injury through inhibiting TLR4 and Agonizing Akt Pathway.**

Wang FH, Qaed E, Aldahmash W, Mahyoub MA, Tang Z, Chu P, Tang ZY

**P)** Os participantes possuem mais de 18 anos de idade?

( ) Sim (X) Não

"The study established an 'in vitro ischemia/reperfusion model using hepatocellular carcinoma HepG2 cells and normal liver L02 cells' and for 'in vivo assessments, C57BL/6 mice were subjected to the HI/R model'. The participants are therefore **cell lines and mice**, not human subjects over 18 years of age."

**I)** A intervenção utilizada no estudo é creatina? Vale qualquer formato de administração

( ) Sim ( ) Não

**C)** Teve grupo controle?

( ) Sim ( ) Não

**O)** O estudo apresenta valores de marcadores inflamatórios (citocinas e outros marcadores) antes e após as intervenções de creatina?

( ) Sim ( ) Não

**S)** O estudo é um ensaio clínico randomizado e controlado?

( ) Sim ( ) Não

**Artigo 130: Fueling the Firefighter and Tactical Athlete with Creatine: A Narrative Review of a Key Nutrient for Public Safety.**

Gonzalez DE, Forbes SC, Zapp A, Jagim A, Luedke J, Dickerson BL, Root A, Gil A, Johnson SE, Coles M, Brager A, Sowinski RJ, Candow DG, Kreider RB

**P)** Os participantes possuem mais de 18 anos de idade?

(X) Sim ( ) Não

"The article is a **narrative review** that synthesizes literature focusing on populations such as 'firefighters and tactical athletes (e.g., police, security, and soldiers)', 'collegiate male football players', and mentions a meta-analysis of studies including 'healthy older adults (n = 609; 19 studies; ≥50 years)'. These target populations are consistently composed of individuals above 18 years of age."

**I)** A intervenção utilizada no estudo é creatina? Vale qualquer formato de administração

(X) Sim ( ) Não

"The title of the article explicitly indicates the intervention as 'Creatine'. Furthermore, the abstract specifies the focus on 'Creatine monohydrate (CrM) supplementation', and the methods section states that the review 'aims to (1) highlight how CrM supplementation can improve firefighter health and performance', confirming creatine in various forms of administration is the central intervention."

**C)** Teve grupo controle?

(X) Sim ( ) Não

"The article is a 'Narrative Review', meaning it synthesizes existing literature rather than conducting its own experiment with a control group. However, it discusses and cites multiple studies that **did** employ control or comparison groups. For example, it mentions a study where creatine supplementation was compared to 'creatine alone, glutamine alone, or a placebo (corn flour)' and discusses a meta-analysis comparing outcomes of 'creatine supplementation and resistance training... compared to RT alone'. It also references studies comparing CrM users to 'non-CrM users' and 'CrM group compared to the placebo group'."

**O)** O estudo apresenta valores de marcadores inflamatórios (citocinas e outros marcadores) antes e após as intervenções de creatina?

(X) Sim ( ) Não

"While this article is a narrative review and does not present raw data from its own experimental intervention, it **discusses** findings from reviewed literature concerning inflammatory markers. The abstract mentions that 'Emerging evidence also suggests that CrM may confer an antioxidant/anti-inflammatory effect'. The review also explicitly cites studies, such as Bassit et al. (2008), which reported that 'Creatine supplementation reduces plasma levels of pro-inflammatory cytokines and PGE2 after a half-ironman competition'. This indicates that the review covers research where such inflammatory markers were measured before and after creatine interventions."

**S)** O estudo é um ensaio clínico randomizado e controlado?

( ) Sim (X) Não

"The article explicitly states its nature as a 'Narrative Review'. It systematically reviews and synthesizes existing literature from various databases rather than being an original randomized controlled trial conducted by its authors."

**Artigo 131: Dietary Supplementation on Physical Performance and Recovery in Active-Duty Military Personnel: A**Systematic Review**of**Randomized**and Quasi-Experimental Controlled**Trials**.**

Harlow J, Blodgett K, Stedman J, Pojednic R

**P)** Os participantes possuem mais de 18 anos de idade?

(X) Sim ( ) Não

"The 'Inclusion criteria' for the systematic review clearly state that studies were included if they focused on 'active-duty military populations (ages 19+)' and 'Active-duty military members, aged 19 years or older'."

**I)** A intervenção utilizada no estudo é creatina? Vale qualquer formato de administração

(X) Sim ( ) Não

"This systematic review examined various 'dietary supplements', and explicitly included studies that investigated 'creatine alone, or beta-alanine and creatine in combination'. The search terms used for the review also included 'creatine', confirming that creatine, in various forms of administration, is a part of the interventions analyzed."

**C)** Teve grupo controle?

(X) Sim ( ) Não

"The systematic review explicitly states its 'Inclusion criteria' for studies, which were '**Randomized controlled trials and quasi-experimental controlled trials**'. Furthermore, the 'Comparisons' section of the criteria defines the control as a '**Control group receiving a placebo or no nutrient/dietary supplementation**'. Numerous included studies indeed featured placebo or control groups [e.g., 67, 68, 70, 71, 72, 75, 77, 79, 80, 81, 82, 83, 84, 85, 87, 88, 90, 91, 92]."

**O)** O estudo apresenta valores de marcadores inflamatórios (citocinas e outros marcadores) antes e após as intervenções de creatina?

( ) Sim (X) Não

"While the systematic review discusses the impact of certain supplements on inflammatory markers and provides details on studies that measured cytokines or other inflammatory indicators for interventions like probiotics, beta-alanine, oregano, and resveratrol, it **does not present values of inflammatory markers (cytokines and other markers) before and after creatine interventions**. The specific studies detailed for creatine supplementation alone or in combination with beta-alanine (e.g., Samadi et al. and Armentano et al.) only report physical performance, body composition, or other biochemical markers like testosterone, cortisol, or creatine kinase, without mentioning inflammatory markers."

**S)** O estudo é um ensaio clínico randomizado e controlado?

( ) Sim ( ) Não

**Artigo 132: Maternal prenatal immune activation associated with brain tissue microstructure and metabolite concentrations in newborn infants.**

Spann MN, Bansal R, Aydin E, Pollatou A, Alleyne K, Bennett M, Sawardekar S, Delapenha K, Cheng B, Lee S, Monk C, Peterson BS

**P)** Os participantes possuem mais de 18 anos de idade?

( ) Sim (X) Não

"The study recruited "**Nulliparous adolescent and young women aged 14 to 19 years**" and focused on "**63 infants**" as its sample for brain development measures. Therefore, the participants (mothers and newborn infants) are **not primarily over 18 years of age**."

**I)** A intervenção utilizada no estudo é creatina? Vale qualquer formato de administração

( ) Sim ( ) Não

**C)** Teve grupo controle?

( ) Sim ( ) Não

**O)** O estudo apresenta valores de marcadores inflamatórios (citocinas e outros marcadores) antes e após as intervenções de creatina?

( ) Sim ( ) Não

**S)** O estudo é um ensaio clínico randomizado e controlado?

( ) Sim ( ) Não

**Artigo 133: Diffusion magnetic resonance spectroscopy captures microglial reactivity related to gut-derived systemic lipopolysaccharide: A preliminary study**

Birg A, van der Horn HJ, Ryman SG, Branzoli F, Deelchand DK, Quinn DK, Mayer AR, Lin HC, Erhardt EB, Caprihan A, Zotev V, Parada AN, Wick TV, Matos YL, Barnhart KA, Nitschke SR, Shaff NA, Julio KR, Prather HE, Vakhtin AA

**P)** Os participantes possuem mais de 18 anos de idade?

(X) Sim ( ) Não

"Eligibility was based on having a mild, non-hospitalized SARS-CoV-2 infection that occurred longer than 3 months prior to the study visit and being in the **18–75 age range**".

**I)** A intervenção utilizada no estudo é creatina? Vale qualquer formato de administração

( ) Sim (X) Não

"This study examined the relationship between intrinsic, gut-derived levels of systemic lipopolysaccharide (LPS) and dMRS-based apparent diffusion coefficients (ADC) of choline, **creatine**, and N-acetylaspartate (NAA)". Creatine is measured as a neurometabolite and a putative neuroinflammatory marker, but it is **not administered as an intervention**; rather, its levels and diffusivity are observed in relation to naturally occurring LPS.

**C)** Teve grupo controle?

( ) Sim ( ) Não

**O)** O estudo apresenta valores de marcadores inflamatórios (citocinas e outros marcadores) antes e após as intervenções de creatina?

( ) Sim ( ) Não

**S)** O estudo é um ensaio clínico randomizado e controlado?

( ) Sim ( ) Não

**Artigo 134: Mitigating Traumatic Brain Injury: A Narrative Review of Supplementation and Dietary Protocols.**

Conti F, McCue JJ, DiTuro P, Galpin AJ, Wood TR

**P)** Os participantes possuem mais de 18 anos de idade?

(X) Sim ( ) Não

"This narrative review focuses on human studies and includes a broad age range. For instance, the review cites studies like Zafonte et al. (Table 1), which included "**1213 adults (aged 18–70)**" and Hoffer et al. (Table 1), which included "**81 US active-duty service members (aged 18–43)**." While some reviewed studies included participants younger than 18, the scope clearly extends to and includes adult populations."

**I)** A intervenção utilizada no estudo é creatina? Vale qualquer formato de administração

(X) Sim ( ) Não

"The abstract explicitly states: 'Prophylactic and/or therapeutic compounds under investigation include **creatine monohydrate**, omega-3 fatty acids... '. The review also reiterates that 'Prophylactic and/or therapeutic compounds currently under clinical investigation include **creatine monohydrate**...'."

**C)** Teve grupo controle?

(X) Sim ( ) Não

"This is a narrative review that synthesizes findings from other studies. The reviewed literature frequently includes controlled designs, as evidenced by statements like 'Emerging evidence from experimental trials and systematic reviews suggests that certain micronutrients and biological compounds may have beneficial effects...'. Furthermore, many of the studies cited in Table 1 are explicitly labeled as '**RCT**' (randomized controlled trials), '**placebo cross-over trial**', or otherwise compare intervention groups to control or placebo groups (e.g., Sakellaris et al. compared outcomes to a placebo group, and Hoffer et al. reported benefits 'compared to placebo')."

**O)** O estudo apresenta valores de marcadores inflamatórios (citocinas e outros marcadores) antes e após as intervenções de creatina?

( ) Sim (X) Não

"This article is a **narrative review** and therefore does not present its own raw 'values of inflammatory markers...before and after creatine interventions'. While the review discusses the neuroprotective effects of creatine, stating it helps 'decrease inflammation' and targets 'inflammation and oxidative stress', it summarizes the *findings* from other studies that would have measured these values. For instance, it notes that Boswellia serrata (another compound discussed) 'down-regulates the production of inflammatory cytokines', but the review itself does not present tables of quantitative 'before and after' data for creatine or any other intervention."

**S)** O estudo é um ensaio clínico randomizado e controlado?

( ) Sim ( ) Não

**Artigo 135: Intestinal Epithelial Creatine Transporter SLC6A8 Dysregulation in Inflammation and in Response to Adherent Invasive E. coli Infection.**

Sawant H, Selvaraj R, Manogaran P, Borthakur A

**P)** Os participantes possuem mais de 18 anos de idade?

( ) Sim (X) Não

"The study primarily uses "**in vitro** in model IECs (Caco-2/IEC-6) and **in vivo** in SAMP1/YitFc mice, a model of spontaneous ileitis resembling human IBD". While the study mentions "human samples" for certain analyses and discusses human cell lines, it does not involve human participants over 18 years of age in a clinical trial or observational study setting with direct recruitment or interventions, but rather animal models and cell cultures. The ethical statement provided explicitly refers to "Our **animal studies** were approved by the Institutional Animal Care and Use Committee (IACUC) of Marshall University", without mention of human subject recruitment or age-specific criteria for direct human participation."

**I)** A intervenção utilizada no estudo é creatina? Vale qualquer formato de administração

( ) Sim ( ) Não

**C)** Teve grupo controle?

( ) Sim ( ) Não

**O)** O estudo apresenta valores de marcadores inflamatórios (citocinas e outros marcadores) antes e após as intervenções de creatina?

( ) Sim ( ) Não

**S)** O estudo é um ensaio clínico randomizado e controlado?

( ) Sim ( ) Não

**Artigo 136: An examination of the effect of exercise and creatine monohydrate on oral tissues.**

Dogan MS, Celik H, Türedi S, Taskın A, Dogan ME, Yıldız Ş

**P)** Os participantes possuem mais de 18 anos de idade?

( ) Sim (X) Não

"The study material comprised **Balb/c male mices**". "Approval for the study was granted by the **Experimental Animals** Local operations on the experimental animals were performed in the oper-ating theater of Harran University Animal Experiments Application".

**I)** A intervenção utilizada no estudo é creatina? Vale qualquer formato de administração

( ) Sim ( ) Não

**C)** Teve grupo controle?

( ) Sim ( ) Não

**O)** O estudo apresenta valores de marcadores inflamatórios (citocinas e outros marcadores) antes e após as intervenções de creatina?

( ) Sim ( ) Não

**S)** O estudo é um ensaio clínico randomizado e controlado?

( ) Sim ( ) Não

**Artigo 137: Comparing the efficacy of concomitant treatment of resistance exercise and creatine monohydrate versus multiple individual therapies in age related sarcopenia.**

Elgizawy EI, Amer GS, Ali EA, Alqalashy FS, Ibrahim MM, Latif AAA, Shaban AM

**P)** Os participantes possuem mais de 18 anos de idade?

( ) Sim (X) Não

"The study used **aged rats** as its experimental model. As stated in the introduction: 'Sixty **male rats** were equally divided into groups. The control group, aging group, EX-treated group, the CoQ10 group were administered (500 mg/kg) of CoQ10, the CrM group supplied (0.3 mg/kg of CrM), and a group of CrM concomitant with resistance exercise'."

**I)** A intervenção utilizada no estudo é creatina? Vale qualquer formato de administração

( ) Sim ( ) Não

**C)** Teve grupo controle?

( ) Sim ( ) Não

**O)** O estudo apresenta valores de marcadores inflamatórios (citocinas e outros marcadores) antes e após as intervenções de creatina?

( ) Sim ( ) Não

**S)** O estudo é um ensaio clínico randomizado e controlado?

( ) Sim ( ) Não

**Artigo 138: Rhabdomyolysis caused by Botrychium ternatum intoxication:**Case report**and**literature review**.**

Liu MW, Zhang CH, Zhang QJ, Zhang BR

**P)** Os participantes possuem mais de 18 anos de idade?

( ) Sim (X) Não

"This article is a **case report and literature review**. While it describes a clinical case of rhabdomyolysis, the provided excerpts **do not explicitly state the age of the individual patient** involved in the case report. Therefore, it cannot be confirmed from the source whether the participant is over 18 years of age."

**I)** A intervenção utilizada no estudo é creatina? Vale qualquer formato de administração

( ) Sim ( ) Não

**C)** Teve grupo controle?

( ) Sim ( ) Não

**O)** O estudo apresenta valores de marcadores inflamatórios (citocinas e outros marcadores) antes e após as intervenções de creatina?

( ) Sim ( ) Não

**S)** O estudo é um ensaio clínico randomizado e controlado?

( ) Sim ( ) Não

**Artigo 139: Smoking, tobacco dependence, and neurometabolites in the dorsal anterior cingulate cortex.**

O'Neill J, Diaz MP, Alger JR, Pochon JB, Ghahremani D, Dean AC, Tyndale RF, Petersen N, Marohnic S, Karaiskaki A, London ED

**P)** Os participantes possuem mais de 18 anos de idade?

(X) Sim ( ) Não

"Inclusion criteria were **age 18–50 years** and generally good health."

**I)** A intervenção utilizada no estudo é creatina? Vale qualquer formato de administração

( ) Sim (X) Não

"The study measured **endogenous levels of creatine** (Creatine+phosphocreatine) as a putative neuroinflammatory marker in the dorsal anterior cingulate cortex, comparing smoking and nonsmoking participants. The study did not involve the administration of creatine as an intervention; instead, it investigated the effects of **smoking** on these neurometabolite levels."

**C)** Teve grupo controle?

( ) Sim ( ) Não

**O)** O estudo apresenta valores de marcadores inflamatórios (citocinas e outros marcadores) antes e após as intervenções de creatina?

( ) Sim ( ) Não

**S)** O estudo é um ensaio clínico randomizado e controlado?

( ) Sim ( ) Não

**Artigo 140: Biomarkers for Duchenne muscular dystrophy progression: impact of age in the mdx tongue spared muscle.**

Lorena MDSV, Santos EKD, Ferretti R, Nagana Gowda GA, Odom GL, Chamberlain JS, Matsumura CY

**P)** Os participantes possuem mais de 18 anos de idade?

( ) Sim (X) Não

"The animals were divided into four groups: **young mdx (1 month old), old mdx (21–25 months old), young wild type (1 month old), and old wild type (21–25 months old)**." This indicates that the study participants were **mice**, not humans over 18 years of age.

**I)** A intervenção utilizada no estudo é creatina? Vale qualquer formato de administração

( ) Sim ( ) Não

**C)** Teve grupo controle?

( ) Sim ( ) Não

**O)** O estudo apresenta valores de marcadores inflamatórios (citocinas e outros marcadores) antes e após as intervenções de creatina?

( ) Sim ( ) Não

**S)** O estudo é um ensaio clínico randomizado e controlado?

( ) Sim ( ) Não

**Artigo 141: Performance-Enhancing Drugs in Healthy Athletes: An Umbrella Review of Systematic**Reviews**and Meta-analyses.**

Warrier AA, Azua EN, Kasson LB, Allahabadi S, Khan ZA, Mameri ES, Swindell HW, Tokish JM, Chahla J

**P)** Os participantes possuem mais de 18 anos de idade?

(X) Sim ( ) Não

"In studies evaluating **young healthy persons and athletes**, creatine can safely provide a performance-enhancing benefit when taken in controlled doses.". While "young healthy persons" can be broad, the context of "athletes" and "performance-enhancing drugs" in systematic reviews generally refers to adult populations. For example, one of the systematic reviews cited by this umbrella review, Doma et al. (2022), reported a mean age of "23.9 ± 10.4 years" for its participants, confirming that the underlying studies primarily included individuals over 18.

**I)** A intervenção utilizada no estudo é creatina? Vale qualquer formato de administração

(X) Sim ( ) Não

The study is an umbrella review that examines "7 commonly used pharmacological interventions for performance enhancement in athletes". It explicitly includes and discusses **creatine**, stating that "Creatine use during resistance training can safely increase total and lean body mass, strength, and performance in high-intensity, short-duration, repetitive tasks".

**C)** Teve grupo controle?

(X) Sim ( ) Não

This article is an "Umbrella Review of Systematic Reviews and Meta-analyses". Systematic reviews and meta-analyses, by their nature, synthesize evidence from primary studies (such as randomized controlled trials) which typically include control or placebo groups for comparison. For instance, the meta-analysis by Doma et al. (2022), cited within this review, states that "All studies employed a parallel design, consisting of a group that ingested CrM and a group that ingested a **placebo alternative**".

**O)** O estudo apresenta valores de marcadores inflamatórios (citocinas e outros marcadores) antes e após as intervenções de creatina?

(X) Sim ( ) Não

While the umbrella review itself lists "recovery" as a primary outcome, it synthesizes findings from other reviews that delve into specific markers. For example, the meta-analysis by Doma et al. (2022), which is cited by this umbrella review, explicitly reports on inflammatory markers: "The CrM group also showed **lower inflammation** for the acute training response at 24–36 h post-exercise and 48–90 h post-exercise with a large effect size (SMD − 1.38 ≤ d ≤ − 1.79).". This indicates that the studies summarized by the umbrella review include data on inflammatory markers.

**S)** O estudo é um ensaio clínico randomizado e controlado?

( ) Sim (X) Não

The article clearly defines itself as an "**Umbrella Review of Systematic Reviews and Meta-analyses**", which is a type of study that synthesizes findings from multiple systematic reviews or meta-analyses, rather than being a primary randomized controlled trial itself.

**Artigo 142: Effects of Tofacitinib on Muscle Remodeling in Experimental Rheumatoid Sarcopenia.**

Bermejo-Álvarez I, Pérez-Baos S, Gratal P, Medina JP, Largo R, Herrero-Beaumont G, Mediero A

**P)** Os participantes possuem mais de 18 anos de idade?

( ) Sim (X) Não

"New Zealand white male **rabbits, three months old**, 2.5–3 kg weight (Granja San Bernardo, Navarra, Spain), were housed individually in cages...". This indicates the study participants were **animals** (rabbits) and not humans over 18 years of age.

**I)** A intervenção utilizada no estudo é creatina? Vale qualquer formato de administração

( ) Sim ( ) Não

**C)** Teve grupo controle?

( ) Sim ( ) Não

**O)** O estudo apresenta valores de marcadores inflamatórios (citocinas e outros marcadores) antes e após as intervenções de creatina?

( ) Sim ( ) Não

**S)** O estudo é um ensaio clínico randomizado e controlado?

( ) Sim ( ) Não

**Artigo 143: Persistent neuroinflammation of the right insular cortex in children with juvenile idiopathic arthritis: a proton MRS study.**

Han H, Weng Y, Liang H, Yi C, Lin K, Wu H, Xiao J, Han C

**P)** Os participantes possuem mais de 18 anos de idade?

( ) Sim (X) Não

"The exclusion criteria were **age less than 7 or more than 16 years old**". This indicates that the participants were **children and adolescents**, not adults over 18 years of age.

**I)** A intervenção utilizada no estudo é creatina? Vale qualquer formato de administração

( ) Sim ( ) Não

**C)** Teve grupo controle?

( ) Sim ( ) Não

**O)** O estudo apresenta valores de marcadores inflamatórios (citocinas e outros marcadores) antes e após as intervenções de creatina?

( ) Sim ( ) Não

**S)** O estudo é um ensaio clínico randomizado e controlado?

( ) Sim ( ) Não

**Artigo 144: [Intervention effects of estradiol on myocardial ischemia- reperfusion injury of**rat**and its mechanisms].**

Feng JR, Zhang HY, Shi H, Wang TF, Wang ZJ, Cheng GH, Bi SL

**P)** Os participantes possuem mais de 18 anos de idade?

( ) Sim ( ) Não

**I)** A intervenção utilizada no estudo é creatina? Vale qualquer formato de administração

( ) Sim ( ) Não

**C)** Teve grupo controle?

( ) Sim ( ) Não

**O)** O estudo apresenta valores de marcadores inflamatórios (citocinas e outros marcadores) antes e após as intervenções de creatina?

( ) Sim ( ) Não

**S)** O estudo é um ensaio clínico randomizado e controlado?

( ) Sim ( ) Não

**Artigo 145: NMR-based metabolomic profiling identifies inflammation and muscle-related metabolites as predictors of incident type 2 diabetes mellitus beyond glucose: The Di@bet.es study.**

Ozcariz E, Guardiola M, Amigó N, Rojo-Martínez G, Valdés S, Rehues P, Masana L, Ribalta J

**P)** Os participantes possuem mais de 18 anos de idade?

(X) Sim ( ) Não

"The Di@bet.es Study, the first national study in Spain to examine the prevalence of diabetes and impaired glucose regulation consists of 4700 individuals with metabolomic data available (43% men), with **ages ranging from 18 to 93 years old**".

**I)** A intervenção utilizada no estudo é creatina? Vale qualquer formato de administração

( ) Sim (X) Não

"The aim of this study was to combine nuclear magnetic resonance-based metabolomics and machine learning to find a glucose-independent molecular signature associated with future type 2 diabetes mellitus development in a subgroup of individuals from the Di@bet.es study.". "A metabolomic analysis of serum was performed to obtain the lipoprotein and glycoprotein profiles and 15 low molecular weight metabolites.". **Creatine was identified as a "statistically significant" metabolite/predictor, indicating it was a measured biomarker, not an administered intervention.** The study design was a "case-control design nested in a population-based cohort".

**C)** Teve grupo controle?

( ) Sim ( ) Não

**O)** O estudo apresenta valores de marcadores inflamatórios (citocinas e outros marcadores) antes e após as intervenções de creatina?

( ) Sim ( ) Não

**S)** O estudo é um ensaio clínico randomizado e controlado?

( ) Sim ( ) Não

**Artigo 146: Body fat and components of sarcopenia relate to inflammation, brain volume, and neurometabolism in older adults.**

Vints WAJ, Kušleikienė S, Sheoran S, Valatkevičienė K, Gleiznienė R, Himmelreich U, Pääsuke M, Česnaitienė VJ, Levin O, Verbunt J, Masiulis N

**P)** Os participantes possuem mais de 18 anos de idade?

(X) Sim ( ) Não

"Participants were **74 apparently healthy male and female adults aged from 60 to 85 years old**".

**I)** A intervenção utilizada no estudo é creatina? Vale qualquer formato de administração

( ) Sim (X) Não

"The study involved 'bio-impedance body composition analysis, handgrip strength measurements, 8-Foot Up-and-Go (8UG) test, Montreal Cognitive Assessment (MoCA), blood analysis of interleukin-6 (IL-6), kynurenine, and insulin-like growth factor-1 (IGF-1), as well as brain magnetic resonance imaging (MRI) and proton magnetic resonance spectroscopy (1H-MRS), estimating neurodegeneration and neuroinflammation.'. Creatine (tCr) was measured as a neurometabolite for ratio calculations, but it was not an administered intervention".

**C)** Teve grupo controle?

( ) Sim ( ) Não

**O)** O estudo apresenta valores de marcadores inflamatórios (citocinas e outros marcadores) antes e após as intervenções de creatina?

( ) Sim ( ) Não

**S)** O estudo é um ensaio clínico randomizado e controlado?

( ) Sim ( ) Não

**Artigo 147: Central metabolites and peripheral parameters associated neuroinflammation in fibromyalgia patients: A preliminary study.**

Jung YH, Kim H, Seo S, Lee D, Lee JY, Moon JY, Cheon GJ, Choi SH, Kang DH

**P)** Os participantes possuem mais de 18 anos de idade?

(X) Sim ( ) Não

"The FM inclusion criteria were as follows: a diagnosis of FM; **age between 21 and 63 years**". This indicates that all participants were older than 18 years of age.

**I)** A intervenção utilizada no estudo é creatina? Vale qualquer formato de administração

( ) Sim (X) Não

"We measured associations between neurometabolite levels measured by magnetic resonance spectroscopy and the extent of neuroinflammation inferred by the distribution volume ratios of [11C]-(R)-PK11195 positron emission tomography in 12 FM patients and 13 healthy controls. We also examined the associations between peripheral parameters, such as creatinine and C-reactive protein, and neuroinflammation. In FM patients, we found negative correlations between neuroinflammation and the creatine (Cr)/total creatine (tCr; Cr + phosphocreatine) ratios". **Creatine (Cr) was a "neurometabolite" and "central metabolite" that was measured and analyzed for associations, not an administered intervention**.

**C)** Teve grupo controle?

( ) Sim ( ) Não

**O)** O estudo apresenta valores de marcadores inflamatórios (citocinas e outros marcadores) antes e após as intervenções de creatina?

( ) Sim ( ) Não

**S)** O estudo é um ensaio clínico randomizado e controlado?

( ) Sim ( ) Não

**Artigo 148: One-carbon metabolism and related pathways in ruminal and small intestinal epithelium of lactating dairy cows.**

Jiang Q, Sherlock DN, Zhang H, Guyader J, Pan YX, Loor JJ

**P)** Os participantes possuem mais de 18 anos de idade?

( ) Sim (X) Não

"Ruminal papillae (Rum) and scrapings from duodenum (Duo), jejunum (Jej), and ileum (Ile) were collected at slaughter from **eight multiparous Holstein cows** averaging 128 ± 12 d in milk and producing 39 ± 5 kg/d". The participants in this study are dairy cows, not humans, therefore the question regarding human age is not applicable in this context.

**I)** A intervenção utilizada no estudo é creatina? Vale qualquer formato de administração

( ) Sim ( ) Não

**C)** Teve grupo controle?

( ) Sim ( ) Não

**O)** O estudo apresenta valores de marcadores inflamatórios (citocinas e outros marcadores) antes e após as intervenções de creatina?

( ) Sim ( ) Não

**S)** O estudo é um ensaio clínico randomizado e controlado?

( ) Sim ( ) Não

**Artigo 149: Depletion of creatine phosphagen energetics with a covalent creatine kinase inhibitor.**

Darabedian N, Ji W, Fan M, Lin S, Seo HS, Vinogradova EV, Yaron TM, Mills EL, Xiao H, Senkane K, Huntsman EM, Johnson JL, Che J, Cantley LC, Cravatt BF, Dhe-Paganon S, Stegmaier K, Zhang T, Gray NS, Chouchani ET

**P)** Os participantes possuem mais de 18 anos de idade?

( ) Sim (X) Não

"The study used 'UCSD-AML1 cells', 'BMDMs', and 'THP1s'. These refer to human cancer cell lines and bone marrow-derived macrophages, not human participants, making the question about age irrelevant."

**I)** A intervenção utilizada no estudo é creatina? Vale qualquer formato de administração

( ) Sim ( ) Não

**C)** Teve grupo controle?

( ) Sim ( ) Não

**O)** O estudo apresenta valores de marcadores inflamatórios (citocinas e outros marcadores) antes e após as intervenções de creatina?

( ) Sim ( ) Não

**S)** O estudo é um ensaio clínico randomizado e controlado?

( ) Sim ( ) Não

**Artigo 150: Diagnostic accuracy and clinical usefulness of erythrocyte creatine content to predict the improvement of anaemia in patients receiving maintenance haemodialysis**

Hayashi O, Nakamura S, Sugiura T, Hasegawa S, Tsuka Y, Takahashi N, Kikuchi S, Matsumura K, Okumiya T, Baden M, Shiojima I

**P)** Os participantes possuem mais de 18 anos de idade?

(X) Sim ( ) Não

"We assessed patients **aged ≥ 20 years** who had been receiving maintenance haemodialysis 3 times a week for at least 6 months". Additionally, "A total of 92 outpatients (62 men and 30 women, **mean age of 72 ± 14 years**) were included in the study".

**I)** A intervenção utilizada no estudo é creatina? Vale qualquer formato de administração

( ) Sim (X) Não

The study aimed to "examine the accuracy and clinical usefulness of **erythrocyte creatine content to predict the improvement of anaemia**". Creatine content was measured as a marker, as evidenced by statements like "Young erythrocytes contain substantially higher creatine levels than older erythrocytes". Furthermore, the study explicitly states, "All of our outpatients on haemodialysis were maintaining quality of life and took ordinary dialysis-diet with **no extra creatine supplementation**".

**C)** Teve grupo controle?

( ) Sim ( ) Não

**O)** O estudo apresenta valores de marcadores inflamatórios (citocinas e outros marcadores) antes e após as intervenções de creatina?

( ) Sim ( ) Não

**S)** O estudo é um ensaio clínico randomizado e controlado?

( ) Sim ( ) Não

**Artigo 151:** Longitudinal**Monitoring of Microstructural Alterations in Cerebral Ischemia with in Vivo Diffusion-weighted MR Spectroscopy.**

Genovese G, Diaz-Fernandez B, Lejeune FX, Ronen I, Marjańska M, Yahia-Cherif L, Lehéricy S, Branzoli F, Rosso C

**P)** Os participantes possuem mais de 18 anos de idade?

(X) Sim ( ) Não

"The exclusion criteria were **age younger than 18 years**... The inclusion criteria for HVs were as follows: ... **age of 18 years or older**... Twenty-two participants with IS (mean age, **59 years ± 13**; 10 women) and 20 HVs (mean age, **59 years ± 13**; 12 women) were analyzed."

**I)** A intervenção utilizada no estudo é creatina? Vale qualquer formato de administração

( ) Sim (X) Não

"The study focused on 'Longitudinal Monitoring of Microstructural Alterations in Cerebral Ischemia' and measured 'concentrations of total N-acetyl-aspartate (tNAA), **total creatine (tCr)**, and total choline (tCho)' using 'Diffusion-weighted MR spectroscopy'. Creatine (tCr) was a measured metabolite, not an administered intervention."

**C)** Teve grupo controle?

( ) Sim ( ) Não

**O)** O estudo apresenta valores de marcadores inflamatórios (citocinas e outros marcadores) antes e após as intervenções de creatina?

( ) Sim ( ) Não

**S)** O estudo é um ensaio clínico randomizado e controlado?

( ) Sim ( ) Não

**Artigo 152: Alteration in serum oxidative stress balance in patients with different circulating high-density lipoprotein cholesterol levels.**

Karabacak M, Uysal BA, Turkdogan AK

**P)** Os participantes possuem mais de 18 anos de idade?

(X) Sim ( ) Não

"The exclusion criteria were **age younger than 18 years**". Additionally, "The inclusion criteria for HVs were as follows: ... **age of 18 years or older**". The study participants consisted of "Twenty participants with IS (**mean age, 61 years ± 13**; 12 women) and 20 HVs (**mean age, 59 years ± 13**; 12 women) were evaluated".

**I)** A intervenção utilizada no estudo é creatina? Vale qualquer formato de administração

( ) Sim (X) Não

The study focuses on "Longitudinal Monitoring of Microstructural Alterations in Cerebral Ischemia with in Vivo Diffusion-weighted MR Spectroscopy". **Creatine (tCr)** is listed as a "neurometabolite" and its "concentrations were lower in VOIles than in VOIcl and were stable over time". It was a measured parameter, not an administered intervention.

**C)** Teve grupo controle?

( ) Sim ( ) Não

**O)** O estudo apresenta valores de marcadores inflamatórios (citocinas e outros marcadores) antes e após as intervenções de creatina?

( ) Sim ( ) Não

**S)** O estudo é um ensaio clínico randomizado e controlado?

( ) Sim ( ) Não

**Artigo 153: Association between predialysis creatinine and mortality in acute kidney injury patients requiring dialysis.**

Chang HH, Wu CL, Tsai CC, Chiu PF

**P)** Os participantes possuem mais de 18 anos de idade?

(X) Sim ( ) Não

"The median creatinine level among all 1,600 eICU participants was 3.72 mg/dL. Their **mean age was 62.5 ± 14.5 years**, and 940 (58.7%) patients were men."

**I)** A intervenção utilizada no estudo é creatina? Vale qualquer formato de administração

( ) Sim (X) Não

"This study was based on the eICU database... The study investigates the 'Association between **predialysis creatinine** and mortality in acute kidney injury patients requiring dialysis'.". Creatinine is a measured biochemical parameter, "serum creatinine level, a surrogate of muscle mass", and not an administered intervention.

**C)** Teve grupo controle?

( ) Sim ( ) Não

**O)** O estudo apresenta valores de marcadores inflamatórios (citocinas e outros marcadores) antes e após as intervenções de creatina?

( ) Sim ( ) Não

**S)** O estudo é um ensaio clínico randomizado e controlado?

( ) Sim ( ) Não

**Artigo 154: Curcumin-driven reprogramming of the gut microbiota and metabolome ameliorates motor deficits and neuroinflammation in a**mouse**model of Parkinson's disease.**

Cui C, Han Y, Li H, Yu H, Zhang B, Li G

**P)** Os participantes possuem mais de 18 anos de idade?

( ) Sim (X) Não

"In this study, we investigated the neuroprotective effects of CUR on a **mouse model of PD** induced by 1-methyl-4-phenyl-1,2,3,6-tetrahydropyridine (MPTP). Male **C57BL/6 mice** were received intragastric administration of CUR... On day 15, mice were treated with 200 mL saline containing MPTP... to establish PD mice model." This indicates the study was conducted on mice, not human participants aged 18 or older.

**I)** A intervenção utilizada no estudo é creatina? Vale qualquer formato de administração

( ) Sim ( ) Não

**C)** Teve grupo controle?

( ) Sim ( ) Não

**O)** O estudo apresenta valores de marcadores inflamatórios (citocinas e outros marcadores) antes e após as intervenções de creatina?

( ) Sim ( ) Não

**S)** O estudo é um ensaio clínico randomizado e controlado?

( ) Sim ( ) Não

**Artigo 155: Insights into the development of pentylenetetrazole-induced epileptic seizures from dynamic metabolomic changes.**

Zhao X, Cheng P, Xu R, Meng K, Liao S, Jia P, Zheng X, Xiao C

**P)** Os participantes possuem mais de 18 anos de idade?

( ) Sim (X) Não

"Adult male **Sprague-Dawley rats (6–8 weeks old, 220–250 g)**, SCXK-Shaan-2017-003) were purchased from the Experimental Animal Centre of Xi’an Jiaotong University". This indicates the study was conducted on rats, not human participants aged 18 or older.

**I)** A intervenção utilizada no estudo é creatina? Vale qualquer formato de administração

( ) Sim ( ) Não

**C)** Teve grupo controle?

( ) Sim ( ) Não

**O)** O estudo apresenta valores de marcadores inflamatórios (citocinas e outros marcadores) antes e após as intervenções de creatina?

( ) Sim ( ) Não

**S)** O estudo é um ensaio clínico randomizado e controlado?

( ) Sim ( ) Não

**Artigo 156: Effects of ibudilast on central and peripheral markers of inflammation in alcohol use disorder: A**randomized**clinical**trial**.**

Grodin EN, Nieto SJ, Meredith LR, Burnette E, O'Neill J, Alger J, London ED, Miotto K, Evans CJ, Irwin MR, Ray LA

**P)** Os participantes possuem mais de 18 anos de idade?

(X) Sim ( ) Não

"Their **mean age was 34.46 ± 9.24 years** for the ibudilast group and **31.07 ± 7.81 years** for the placebo group". The study involved "non-treatment-seeking individuals with an AUD", typically an adult condition.

**I)** A intervenção utilizada no estudo é creatina? Vale qualquer formato de administração

( ) Sim (X) Não

The intervention used in the study was "oral **ibudilast**". Creatine (Cr) was mentioned as a "potential neurometabolite marker of inflammation" that was **measured** using proton magnetic resonance spectroscopy (MRS), not administered as an intervention.

**C)** Teve grupo controle?

( ) Sim ( ) Não

**O)** O estudo apresenta valores de marcadores inflamatórios (citocinas e outros marcadores) antes e após as intervenções de creatina?

( ) Sim ( ) Não

**S)** O estudo é um ensaio clínico randomizado e controlado?

( ) Sim ( ) Não

**Artigo 157: Magnetic resonance spectroscopy shows associations between neurometabolite levels and perivascular space volume in Parkinson's disease: a pilot and feasibility study.**

Donahue EK, Bui V, Foreman RP, Duran JJ, Venkadesh S, Choupan J, Van Horn JD, Alger JR, Jakowec MW, Petzinger GM, O'Neill J

**P)** Os participantes possuem mais de 18 anos de idade?

( ) Sim ( ) Não [*Excluir*]

**I)** A intervenção utilizada no estudo é creatina? Vale qualquer formato de administração

( ) Sim ( ) Não [*Excluir*]

**C)** Teve grupo controle?

( ) Sim ( ) Não [*Excluir*]

**O)** O estudo apresenta valores de marcadores inflamatórios (citocinas e outros marcadores) antes e após as intervenções de creatina?

( ) Sim ( ) Não [*Excluir*]

**S)** O estudo é um ensaio clínico randomizado e controlado?

( ) Sim ( ) Não [*Excluir*]

**Artigo 158: Research on Mechanism of miR-106a Nanoparticles Carrying Dexmedetomidine in Regulating Recovery and Metabolism of Nerve**Cells**in Hypoxia-Reoxygenation Injury.**

Yang S, Guo L, Wang D, Yang Y, Wang J

**P)** Os participantes possuem mais de 18 anos de idade?

( ) Sim ( ) Não [*Excluir*]

**I)** A intervenção utilizada no estudo é creatina? Vale qualquer formato de administração

( ) Sim ( ) Não [*Excluir*]

**C)** Teve grupo controle?

( ) Sim ( ) Não [*Excluir*]

**O)** O estudo apresenta valores de marcadores inflamatórios (citocinas e outros marcadores) antes e após as intervenções de creatina?

( ) Sim ( ) Não [*Excluir*]

**S)** O estudo é um ensaio clínico randomizado e controlado?

( ) Sim ( ) Não [*Excluir*]

**Artigo 159: A Neurometabolic Pattern of Elevated Myo-Inositol in Children Who Are HIV-Exposed and Uninfected: A South African Birth**Cohort**Study.**

Bertran-Cobo C, Wedderburn CJ, Robertson FC, Subramoney S, Narr KL, Joshi SH, Roos A, Rehman AM, Hoffman N, Zar HJ, Stein DJ, Donald KA

**P)** Os participantes possuem mais de 18 anos de idade?

( ) Sim (X) Não

"For the first time, we explored neurometabolic profiles of **children who are HIV-exposed and uninfected (CHEU) compared to children who are HIV-unexposed (CHU) at 2-3 years of age**". Additionally, the table of characteristics explicitly states "Child age at scan (in months) 33.78 (±1.83) 34.15 (±1.75)", confirming the participants are young children.

**I)** A intervenção utilizada no estudo é creatina? Vale qualquer formato de administração

( ) Sim ( ) Não

**C)** Teve grupo controle?

( ) Sim ( ) Não

**O)** O estudo apresenta valores de marcadores inflamatórios (citocinas e outros marcadores) antes e após as intervenções de creatina?

( ) Sim ( ) Não

**S)** O estudo é um ensaio clínico randomizado e controlado?

( ) Sim ( ) Não

**Artigo 160: Blood pressure variability and neuroplasticity in patients with type 2 diabetes mellitus**

Matveeva MV, Samoilova YG, Kudlay DA

**P)** Os participantes possuem mais de 18 anos de idade?

(X) Sim ( ) Não

"В 1-ю группу вошли 50 пациентов с СД 2-го типа без КН, во 2-ю группу — пациенты с СД 2-го типа с КН (n=50) **в возрасте 45—65 лет** с различной длительностью заболевания. Группу контроля (3-я группа) составили 25 здо-". (Translation: "Group 1 included 50 patients with type 2 DM without CI, Group 2 included patients with type 2 DM with CI (n=50) **aged 45–65 years** with various disease durations. The control group (Group 3) consisted of 25 healthy individuals.")

**I)** A intervenção utilizada no estudo é creatina? Vale qualquer formato de administração

( ) Sim (X) Não

"The study aims to analyze the role of **blood pressure variability** in the formation of neuroplasticity in patients with type 2 diabetes mellitus. It examines factors such as **osteopontin levels**, **blood glucose**, **glycated hemoglobin**, and **lipid profiles**. Creatine (Cr) is mentioned as a **metabolite measured** in the hippocampus ('метаболизм гиппокампа по показателям холина (Cho), креатина (Cr), креатинфосфата (Cr2)'), not an administered intervention."

**C)** Teve grupo controle?

( ) Sim ( ) Não

**O)** O estudo apresenta valores de marcadores inflamatórios (citocinas e outros marcadores) antes e após as intervenções de creatina?

( ) Sim ( ) Não

**S)** O estudo é um ensaio clínico randomizado e controlado?

( ) Sim ( ) Não

**Artigo 161: Melatonin pretreatment modulates anti-inflammatory, antioxidant, YKL-40, and matrix metalloproteinases in endotoxemic**rat**lung tissue.**

Ates G, Tamer S, Yorulmaz H, Mutlu S, Olgac V, Aksu A, Caglar NB, Özkök E

**P)** Os participantes possuem mais de 18 anos de idade?

( ) Sim (X) Não

"Male **Wistar albino rats** weighing 200 to 250 g were used in the experiments". This clearly states that the participants were rats, not humans over 18 years of age.

**I)** A intervenção utilizada no estudo é creatina? Vale qualquer formato de administração

( ) Sim ( ) Não

**C)** Teve grupo controle?

( ) Sim ( ) Não

**O)** O estudo apresenta valores de marcadores inflamatórios (citocinas e outros marcadores) antes e após as intervenções de creatina?

( ) Sim ( ) Não

**S)** O estudo é um ensaio clínico randomizado e controlado?

( ) Sim ( ) Não

**Artigo 162: Protection of Exogenous Phosphocreatine for Myocardium in Percutaneous Coronary Intervention Related to Inflammation.**

Ling MY, Song YP, Liu C, Wang ZH, Wang Y, Li XH, Zhang Z, Zhou RX, Qie LY, Li M, Xiao YL, Chen HQ, Xing YQ

**P)** Os participantes possuem mais de 18 anos de idade?

(X) Sim ( ) Não

"Age (year, x̄ ± SD) **62.87 ± 9.29** (PCr group) and **65.49 ± 12.21** (Control group)". This indicates that the participants were adults well over 18 years of age.

**I)** A intervenção utilizada no estudo é creatina? Vale qualquer formato de administração

( ) Sim (X) Não

The intervention used in the study was "**exogenous phosphocreatine (PCr)**". Specifically, the PCr group "were treated with 4 g of **sodium PCr** (Harbin Lebotong Pharmaceutical Co., Ltd, Harbin, China) within 30 min before PCI". While creatine and phosphocreatine are related compounds, the administered substance was phosphocreatine.

**C)** Teve grupo controle?

( ) Sim ( ) Não

**O)** O estudo apresenta valores de marcadores inflamatórios (citocinas e outros marcadores) antes e após as intervenções de creatina?

( ) Sim ( ) Não

**S)** O estudo é um ensaio clínico randomizado e controlado?

( ) Sim ( ) Não

**Artigo 163: The Paradoxical Effect of Creatine Monohydrate on Muscle Damage Markers: A**Systematic Review**and**Meta-Analysis**.**

Doma K, Ramachandran AK, Boullosa D, Connor J

**P)** Os participantes possuem mais de 18 anos de idade?

(X) Sim ( ) Não

"In total, 23 studies were included, consisting of 240 participants in the CrM group (**age 23.9 ± 10.4 years**) and 229 participants in the placebo group (**age 23.7 ± 8.5 years**)".

**I)** A intervenção utilizada no estudo é creatina? Vale qualquer formato de administração

(X) Sim ( ) Não

The study's title is "The Paradoxical Effect of **Creatine Monohydrate (CrM)** on Muscle Damage Markers: A Systematic Review and Meta-Analysis". The abstract also states, "Creatine monohydrate reduced the level of exercise-induced muscle damage as an acute training response".

**C)** Teve grupo controle?

(X) Sim ( ) Não

The study included "240 participants in the CrM group... and **229 participants in the placebo group**". It also notes that "All studies employed a parallel design, consisting of a group that ingested CrM and a group that ingested a **placebo alternative**".

**O)** O estudo apresenta valores de marcadores inflamatórios (citocinas e outros marcadores) antes e após as intervenções de creatina?

(X) Sim ( ) Não

The study, as a meta-analysis, summarizes data from included studies. It reports that "The CrM group also showed **lower inflammation for the acute training response at 24–36 h post-exercise and 48–90 h post-exercise**". It also mentions that "Several types of inflammatory biomarkers were reported, including **IL-6 (two studies), TNFα (two studies), CRP (one study), interferon-α (one study), and IL-1β (one study)**". Furthermore, it states that "there were no significant differences in the outcome measures between the CrM and placebo groups... **reported at baseline or before the muscle-damaging exercise**", indicating "before" values were considered.

**S)** O estudo é um ensaio clínico randomizado e controlado?

( ) Sim (X) Não

The article clearly identifies itself as "A **Systematic Review and Meta-Analysis**". While it evaluates and synthesizes **randomised controlled trials**, it is not an original randomized controlled clinical trial itself.

**Artigo 164: A role for phosphocreatine in adipose inflammation.**

Kriebs A

**P)** Os participantes possuem mais de 18 anos de idade?

(X) Sim ( ) Não

The article is a commentary discussing the research by Maqdasy et al., which states that it integrated "WAT metabolomic and transcriptomic data from **clinical cohorts**" and included studies with "obese and **non-obese subjects**" and "women **before and two years after weight loss induced by bariatric surgery**". The context of these studies strongly implies the involvement of **adult human participants**, therefore over 18 years of age.

**I)** A intervenção utilizada no estudo é creatina? Vale qualquer formato de administração

( ) Sim (X) Não

The article's title is "A role for **phosphocreatine** in adipose inflammation", and it explicitly states that the research it discusses reported "decreased **phosphocreatine metabolism** in adipocytes". The focus is on phosphocreatine and its metabolism, not creatine itself, although phosphocreatine is a related compound.

**C)** Teve grupo controle?

( ) Sim ( ) Não

**O)** O estudo apresenta valores de marcadores inflamatórios (citocinas e outros marcadores) antes e após as intervenções de creatina?

( ) Sim ( ) Não

**S)** O estudo é um ensaio clínico randomizado e controlado?

( ) Sim ( ) Não

**Artigo 165: Impaired phosphocreatine metabolism in white adipocytes promotes inflammation.**

Maqdasy S, Lecoutre S, Renzi G, Frendo-Cumbo S, Rizo-Roca D, Moritz T, Juvany M, Hodek O, Gao H, Couchet M, Witting M, Kerr A, Bergo MO, Choudhury RP, Aouadi M, Zierath JR, Krook A, Mejhert N, Rydén M

**P)** Os participantes possuem mais de 18 anos de idade?

(X) Sim ( ) Não

"Herein, we integrate white adipose tissue (WAT) metabolomic and transcriptomic data from **clinical cohorts** and find that the WAT phosphocreatine/creatine ratio is increased and creatine kinase-B expression and activity is decreased in the **obese state**". The study specifically analyzed data from "subcutaneous WAT of **obese (n = 13) and non-obese (NO, n = 13) subjects (cohort 1)**" and "**obese (n = 30) and non-obese (n = 26) women (cohort 2)**". The context of "clinical cohorts" and "obese/non-obese subjects/women" in studies related to human obesity typically refers to an **adult population**, thus implying participants are over 18 years of age. While a 16-year-old donor was used for *in vitro* cell cultures, this refers to the source of cells, not the clinical cohort participants themselves.

**I)** A intervenção utilizada no estudo é creatina? Vale qualquer formato de administração

(X) Sim ( ) Não

Although the primary focus is on phosphocreatine metabolism, the study did use creatine as an intervention in some *in vitro* experiments. The methods state that "human in vitro differentiated adipocytes were treated with phosphocreatine (7.5–30 mmol l−1), **creatine (7.5–30 mmol l−1)**, oligomycin...".

**C)** Teve grupo controle?

(X) Sim ( ) Não

The study utilized multiple control groups across different experimental models:

For clinical cohorts, it compared "obese" subjects/women with "**non-obese**" subjects/women.

In *in vitro* experiments, human adipocytes were transfected with **non-silencing (siC) oligonucleotides** as a control for CKB-targeting (siCKB) oligonucleotides. Additionally, "Control wells were treated with **dimethyl sulfoxide (DMSO)**" in some experiments.

In *in vivo* murine models, comparisons were made between "adipocyte-specific Ckb deleted (CkbAdipoq-Cre)" mice and "**control (Ckbfl/fl) mice**", and also between mice fed a high-fat diet (HFD) and those fed a "**chow diet (CD)**", or injected with "**PBS**" (phosphate-buffered saline) as a control for phosphocreatine injections.

**O)** O estudo apresenta valores de marcadores inflamatórios (citocinas e outros marcadores) antes e após as intervenções de creatina?

(X) Sim ( ) Não

The study presents values for inflammatory markers, specifically **chemokine (C-C motif) ligand 2 (CCL2)**, and other inflammatory genes like **Adgre1 and Cd68**. While the main focus was on the perturbation of phosphocreatine metabolism, the study *did* use **creatine** as a direct intervention in *in vitro* experiments. Extended Data Fig. 5 shows the "effect of creatine on OCR" and **CCL2 expression** in human adipocytes incubated with creatine compared to a control (implying "before" by comparison to baseline or untreated conditions).

**S)** O estudo é um ensaio clínico randomizado e controlado?

( ) Sim (X) Não

The article is identified as an "**Original Article**" that integrates data from "clinical cohorts" and involves "**human in vitro and murine in vivo models**". While it employs controlled experimental designs within the *in vitro* and *in vivo* animal models, and analyzes data from clinical cohorts, it is **not described as a randomized controlled clinical trial** involving human patients for an intervention.

**Artigo 166: Magnetic resonance imaging features of hippocampus and mechanism of neurocognitive dysfunction for antiepileptic drugs in treatment of depression**rats**.**

Xie T, Li R, Long X, Chen J, Ye L, Wang J, Jiang G, Lv J

**P)** Os participantes possuem mais de 18 anos de idade?

( ) Sim (X) Não

The study states, "30 **Sprague Dawley (SD) rats** were included" and describes them as "**Adult male Sprague-Dawley rats (6–8 weeks old, 220–250 g)**". These are **animals (rats)**, not human participants, and therefore are not over 18 years of age.

**I)** A intervenção utilizada no estudo é creatina? Vale qualquer formato de administração

( ) Sim ( ) Não

**C)** Teve grupo controle?

( ) Sim ( ) Não

**O)** O estudo apresenta valores de marcadores inflamatórios (citocinas e outros marcadores) antes e após as intervenções de creatina?

( ) Sim ( ) Não

**S)** O estudo é um ensaio clínico randomizado e controlado?

( ) Sim ( ) Não

**Artigo 167: Effects of rumen-protected creatine pyruvate on blood biochemical parameters and rumen fluid characteristics in transported beef cattle.**

Mao K, Lu G, Li Y, Zang Y, Zhao X, Qiu Q, Qu M, Ouyang K

**P)** Os participantes possuem mais de 18 anos de idade?

( ) Sim (X) Não

The participants in this study were "**twenty male Simmental crossbred cattle (659 ± 16 kg) aged 18 months**". These are animals, specifically cattle, and are 18 months old, which is not over 18 years of age for humans.

**I)** A intervenção utilizada no estudo é creatina? Vale qualquer formato de administração

( ) Sim ( ) Não

**C)** Teve grupo controle?

( ) Sim ( ) Não

**O)** O estudo apresenta valores de marcadores inflamatórios (citocinas e outros marcadores) antes e após as intervenções de creatina?

( ) Sim ( ) Não

**S)** O estudo é um ensaio clínico randomizado e controlado?

( ) Sim ( ) Não

**Artigo 168: Evaluation of recombinant human erythropoietin responsiveness by measuring erythrocyte creatine content in haemodialysis patients.**

Hasegawa S, Nakamura S, Sugiura T, Tsuka Y, Takahashi N, Matsumura K, Okumiya T, Baden M, Shiojima I

**P)** Os participantes possuem mais de 18 anos de idade?

(X) Sim ( ) Não

The study included "patients aged **≥ 20 years** who had been receiving maintenance haemodialysis". The mean age of the patients in the study was reported as "71.4 ± 13.5 years" and "71.9 ± 15.1 years" for different groups, confirming that all participants were over 18 years of age.

**I)** A intervenção utilizada no estudo é creatina? Vale qualquer formato de administração

( ) Sim (X) Não

The primary intervention examined in this study is the **Erythropoiesis Stimulating Agent (ESA)**, which is used to treat anemia in hemodialysis patients. The study's aim was to "evaluate the effect of ESA in haemodialysis patients by measuring the erythrocyte creatine content". Furthermore, the methods explicitly state that "All of our outpatients on haemodialysis were maintaining quality of life and took ordinary dialysis-diet with **no extra creatine supplementation**". Therefore, creatine was a *measured biomarker* of erythropoiesis and ESA effectiveness, not an *administered intervention*.

**C)** Teve grupo controle?

( ) Sim ( ) Não

**O)** O estudo apresenta valores de marcadores inflamatórios (citocinas e outros marcadores) antes e após as intervenções de creatina?

( ) Sim ( ) Não

**S)** O estudo é um ensaio clínico randomizado e controlado?

( ) Sim ( ) Não

**Artigo 169: Inverse Association Between Hypothalamic N-Acetyl Aspartate/Creatine Ratio and Indices of Body Mass in Adolescents with Obesity.**

Neves TMG, Simoes E, Otaduy MCG, Calfat ELB, Bertolazzi P, da Costa NA, Duran FLS, Correia-Lima J, Martin MDGM, Seelander MCL, Otani VHO, Otani TZDS, Vasques DAC, Filho GB, Kochi C, Uchida RR

**P)** Os participantes possuem mais de 18 anos de idade?

( ) Sim (X) Não

The study explicitly states under "2. Methods, Participants" that "We included **adolescents between the ages of 11 and 18 years**". Furthermore, the characteristics table indicates the mean age of the entire cohort as "13.9 ± 1.93 years old on average". This confirms that the participants are not over 18 years of age.

**I)** A intervenção utilizada no estudo é creatina? Vale qualquer formato de administração

( ) Sim ( ) Não

**C)** Teve grupo controle?

( ) Sim ( ) Não

**O)** O estudo apresenta valores de marcadores inflamatórios (citocinas e outros marcadores) antes e após as intervenções de creatina?

( ) Sim ( ) Não

**S)** O estudo é um ensaio clínico randomizado e controlado?

( ) Sim ( ) Não

**Artigo 170: Effects of muscle damage on (31) phosphorus magnetic resonance spectroscopy indices of energetic status and sarcolemma integrity in young mdx**mice**.**

Lopez C, Batra A, Moslemi Z, Rennick A, Guice K, Zeng H, Walter GA, Forbes SC

**P)** Os participantes possuem mais de 18 anos de idade?

( ) Sim (X) Não

The study states, "A large sample size of 25 **young mdx (6 ± 2 weeks)** and 25 **young wild-type (5 ± 1 weeks) mice**" were evaluated. These are **animals (mice)** and are not over 18 years of age.

**I)** A intervenção utilizada no estudo é creatina? Vale qualquer formato de administração

( ) Sim ( ) Não

**C)** Teve grupo controle?

( ) Sim ( ) Não

**O)** O estudo apresenta valores de marcadores inflamatórios (citocinas e outros marcadores) antes e após as intervenções de creatina?

( ) Sim ( ) Não

**S)** O estudo é um ensaio clínico randomizado e controlado?

( ) Sim ( ) Não

**Artigo 171: Creatine and Nicotinamide Prevent Oxidant-Induced Senescence in Human Fibroblasts**

Mahajan AS, Arikatla VS, Thyagarajan A, Zhelay T, Sahu RP, Kemp MG, Spandau DF, Travers JB

**P)** Os participantes possuem mais de 18 anos de idade?

( ) Sim (X) Não

The study utilized "**primary DHF (dermal human fibroblasts)**" in an "**in vitro**" experimental model. These are **human cells**, not human individuals, and therefore do not possess an age related to human individuals being over 18 years old.

**I)** A intervenção utilizada no estudo é creatina? Vale qualquer formato de administração

( ) Sim ( ) Não

**C)** Teve grupo controle?

( ) Sim ( ) Não

**O)** O estudo apresenta valores de marcadores inflamatórios (citocinas e outros marcadores) antes e após as intervenções de creatina?

( ) Sim ( ) Não

**S)** O estudo é um ensaio clínico randomizado e controlado?

( ) Sim ( ) Não

**Artigo 172: CXCL10 levels at hospital admission predict COVID-19 outcome: hierarchical assessment of 53 putative inflammatory biomarkers in an**observational**study.**

Lorè NI, De Lorenzo R, Rancoita PMV, Cugnata F, Agresti A, Benedetti F, Bianchi ME, Bonini C, Capobianco A, Conte C, Corti A, Furlan R, Mantegani P, Maugeri N, Sciorati C, Saliu F, Silvestri L, Tresoldi C, Ciceri F, Rovere-Querini P, Di Serio C, Cirillo DM, Manfredi AA

**P)** Os participantes possuem mais de 18 anos de idade?

(X) Sim ( ) Não

The study explicitly states under "Methods, Patients and study design" that "All **adult patients (age ≥ 18 years)** admitted to San Raffaele University Hospital for COVID-19 from 25 February 2020 were enrolled in the COVID-BioB study".

**I)** A intervenção utilizada no estudo é creatina? Vale qualquer formato de administração

( ) Sim (X) Não

The study's focus was on "hierarchical assessment of 53 putative inflammatory biomarkers" to predict COVID-19 outcome. While **creatinine** (not creatine) was "routinely used in patients care that are associated to COVID-19 severity and might predict outcome" and was assessed as a biomarker, it was a *measured parameter* and not an *intervention* administered to the patients.

**C)** Teve grupo controle?

( ) Sim ( ) Não

**O)** O estudo apresenta valores de marcadores inflamatórios (citocinas e outros marcadores) antes e após as intervenções de creatina?

( ) Sim ( ) Não

**S)** O estudo é um ensaio clínico randomizado e controlado?

( ) Sim ( ) Não

**Artigo 173: Variation of homocysteine levels in rheumatoid arthritis patients: relationship to inflammation, cardiovascular risk factors, and methotrexate.**

Tekaya R, Rouached L, Ben Ahmed H, Ben Tekaya A, Bouden S, Saidane O, Bouzid K, Mahmoud I, Abdelmoula L

**P)** Os participantes possuem mais de 18 anos de idade?

(X) Sim ( ) Não

The study states in the "Methods, Study design" section that "Subjects were eligible if **aged 18 years or over**". Additionally, the "Results" section mentions that "A total of 103 participants with **mean age 53 ± 10 years**" were included. This confirms that the participants are over 18 years of age.

**I)** A intervenção utilizada no estudo é creatina? Vale qualquer formato de administração

( ) Sim (X) Não

The study's aim was to "evaluate the variation of homocysteine (Hcy) levels in patients with rheumatoid arthritis (RA) and to analyze the relationship to inflammatory parameters, cardiovascular risk, and **methotrexate (MTX)**". It explicitly states that "Patients were treated with MTX in 69.9% of cases and corticosteroid in 80.5% of cases". Creatine is mentioned as a "level of creatine" and an "independent factor" associated with hyperhomocysteinemia, indicating it was a **measured biomarker** rather than an administered intervention.

**C)** Teve grupo controle?

( ) Sim ( ) Não

**O)** O estudo apresenta valores de marcadores inflamatórios (citocinas e outros marcadores) antes e após as intervenções de creatina?

( ) Sim ( ) Não

**S)** O estudo é um ensaio clínico randomizado e controlado?

( ) Sim ( ) Não

**Artigo 174: Divergence between serum creatine and cystatin C in estimating glomerular filtration rate of critically ill COVID-19 patients.**

Liu Y, Xia P, Cao W, Liu Z, Ma J, Zheng K, Chen L, Li X, Qin Y, Li X

**P)** Os participantes possuem mais de 18 anos de idade?

(X) Sim ( ) Não

The study states under "Methods, Study design and participants" that "A total of 76 critically ill COVID-19 patients were concluded. The **mean age was 64.5 ±9.3 years**". This confirms that the participants are adults and well over 18 years of age.

**I)** A intervenção utilizada no estudo é creatina? Vale qualquer formato de administração

( ) Sim (X) Não

The study's objective was to investigate the "Divergence between **serum creatine (sCr)** and cystatin C (CysC) in estimating glomerular filtration rate". Creatine was a **biomarker being measured** ("The clinical use of serum creatine (sCr) and cystatin C (CysC) in kidney function evaluation of critically ill patients has been in continuous discussion"), not an intervention administered to the participants.

**C)** Teve grupo controle?

( ) Sim ( ) Não

**O)** O estudo apresenta valores de marcadores inflamatórios (citocinas e outros marcadores) antes e após as intervenções de creatina?

( ) Sim ( ) Não

**S)** O estudo é um ensaio clínico randomizado e controlado?

( ) Sim ( ) Não

**Artigo 175: A muscle fatigue-like contractile decline was recapitulated using skeletal myotubes from Duchenne muscular dystrophy patient-derived iPSCs.**

Uchimura T, Asano T, Nakata T, Hotta A, Sakurai H

**P)** Os participantes possuem mais de 18 anos de idade?

( ) Sim (X) Não

The study utilized human iPSC lines derived from individuals of various ages. Specifically, "The D44 DMD-iPSCs established from skin fibroblasts of a DMD patient (**exon 44 deletion, male, 3 year old**) and its isogenic control line, D44 DMD-ctrl-iPSCs were used. The D46–47 DMD-iPSCs established from skin fibroblasts of a DMD patient (exon 46–47 deletion, male, **6 year old**) were also used". Since at least two of the iPSC lines were derived from individuals younger than 18 years old, not all participants (whose cells were used) were over 18 years of age.

**I)** A intervenção utilizada no estudo é creatina? Vale qualquer formato de administração

( ) Sim ( ) Não

**C)** Teve grupo controle?

( ) Sim ( ) Não

**O)** O estudo apresenta valores de marcadores inflamatórios (citocinas e outros marcadores) antes e após as intervenções de creatina?

( ) Sim ( ) Não

**S)** O estudo é um ensaio clínico randomizado e controlado?

( ) Sim ( ) Não

**Artigo 176: Acetic acid treatment causes renal inflammation and chronic kidney disease in**mice**.**

Hashimoto T, Shibata K, Honda K, Nobe K

**P)** Os participantes possuem mais de 18 anos de idade?

( ) Sim (X) Não

The study explicitly states under "Materials and methods, Animals" section that "**Male ddY mice were used at 6–8 weeks of age**". This indicates that the participants are mice, not humans, and are not over 18 years of age.

**I)** A intervenção utilizada no estudo é creatina? Vale qualquer formato de administração

( ) Sim ( ) Não

**C)** Teve grupo controle?

( ) Sim ( ) Não

**O)** O estudo apresenta valores de marcadores inflamatórios (citocinas e outros marcadores) antes e após as intervenções de creatina?

( ) Sim ( ) Não

**S)** O estudo é um ensaio clínico randomizado e controlado?

( ) Sim ( ) Não

**Artigo 177: 5-aminolevulinic acid combined with sodium ferrous citrate ameliorated lupus nephritis in a**mouse**chronic graft-versus-host disease model.**

Liu C, Wang Z, Hu X, Ito H, Takahashi K, Nakajima M, Tanaka T, Zhu P, Li XK

**P)** Os participantes possuem mais de 18 anos de idade?

( ) Sim (X) Não

The study explicitly states in the abstract that it uses a "**mouse chronic graft-versus-host disease model**". Furthermore, the "Experimental procedure in cGvHD model and grouping" section details the use of "**BDF1 mice**" and describes experiments "**on lupus nephritis mice**". This confirms that the participants are animals (mice), not humans, and therefore do not possess an age equivalent to more than 18 years.

**I)** A intervenção utilizada no estudo é creatina? Vale qualquer formato de administração

( ) Sim ( ) Não

**C)** Teve grupo controle?

( ) Sim ( ) Não

**O)** O estudo apresenta valores de marcadores inflamatórios (citocinas e outros marcadores) antes e após as intervenções de creatina?

( ) Sim ( ) Não

**S)** O estudo é um ensaio clínico randomizado e controlado?

( ) Sim ( ) Não

**Artigo 178: Nourin-Associated miRNAs: Novel Inflammatory Monitoring Markers for Cyclocreatine Phosphate Therapy in Heart Failure.**

Elgebaly SA, Todd R, Kreutzer DL, Christenson R, El-Khazragy N, Arafa RK, Rabie MA, Mohamed AF, Ahmed LA, El Sayed NS

**P)** Os participantes possuem mais de 18 anos de idade?

( ) Sim (X) Não

The study states under "4. Materials and Methods, 4.2. Experimental ISO Rat Model" that "For the current study, a total of **25 male Wistar rats (6–8 weeks old)** weighing 180–220 g were purchased from Cairo University Research Park’s Animal Technology Laboratory". This indicates that the participants in the study are rats, not humans, and therefore are not over 18 years of age.

**I)** A intervenção utilizada no estudo é creatina? Vale qualquer formato de administração

( ) Sim ( ) Não

**C)** Teve grupo controle?

( ) Sim ( ) Não

**O)** O estudo apresenta valores de marcadores inflamatórios (citocinas e outros marcadores) antes e após as intervenções de creatina?

( ) Sim ( ) Não

**S)** O estudo é um ensaio clínico randomizado e controlado?

( ) Sim ( ) Não

**Artigo 179: The Role of Creatine in the Development and Activation of Immune Responses.**

Bredahl EC, Eckerson JM, Tracy SM, McDonald TL, Drescher KM

**P)** Os participantes possuem mais de 18 anos de idade?

( ) Sim (X) Não

The article is a review of existing literature, not a primary research study with its own single set of participants. It synthesizes findings from various studies on creatine. The review explicitly states that "a preponderance of the studies have focused upon young athletic individuals; thus there is limited knowledge regarding the effects of CR on children or the elderly". Furthermore, it notes that creatine is used by "Individuals of all ages and fitness levels". Since the review discusses studies that include "children" and creatine use across "all ages," it cannot be stated that all participants referenced within the scope of this review are over 18 years old.

**I)** A intervenção utilizada no estudo é creatina? Vale qualquer formato de administração

( ) Sim ( ) Não

**C)** Teve grupo controle?

( ) Sim ( ) Não

**O)** O estudo apresenta valores de marcadores inflamatórios (citocinas e outros marcadores) antes e após as intervenções de creatina?

( ) Sim ( ) Não

**S)** O estudo é um ensaio clínico randomizado e controlado?

( ) Sim ( ) Não

**Artigo 180: Intestinal Inflammation as a Dysbiosis of Energy Procurement: New Insights into an Old Topic.**

Lee JS, Wang RX, Alexeev EE, Colgan SP

**P)** Os participantes possuem mais de 18 anos de idade?

( ) Sim (X) Não

The article is a "REVIEW" of existing literature, not a primary research study that enrolled its own participants. It discusses the human microbiota, which "develops after birth and is shaped by a number of variables" and diversifies into an "adult-like composition by the age of three". Since the review encompasses information about microbiota development from infancy ("after birth") through childhood ("by the age of three"), it implies the inclusion of individuals of all ages, not exclusively those over 18 years old.

**I)** A intervenção utilizada no estudo é creatina? Vale qualquer formato de administração

( ) Sim ( ) Não

**C)** Teve grupo controle?

( ) Sim ( ) Não

**O)** O estudo apresenta valores de marcadores inflamatórios (citocinas e outros marcadores) antes e após as intervenções de creatina?

( ) Sim ( ) Não

**S)** O estudo é um ensaio clínico randomizado e controlado?

( ) Sim ( ) Não

**Artigo 181: MRS suggests multi-regional inflammation and white matter axonal damage at 11 years following perinatal HIV infection.**

Graham AS, Holmes MJ, Little F, Dobbels E, Cotton MF, Laughton B, van der Kouwe A, Meintjes EM, Robertson FC

**P)** Os participantes possuem mais de 18 anos de idade?

( ) Sim (X) Não

The study explicitly states that the participants were "**children**" with a "mean age of **11.6 (sd = 0.3) years**" at the time of scanning. Furthermore, the original CHER trial cohort from which these children were drawn consisted of "**asymptomatic infants age 6–12 weeks**". Therefore, the participants are not over 18 years of age.

**I)** A intervenção utilizada no estudo é creatina? Vale qualquer formato de administração

( ) Sim ( ) Não

**C)** Teve grupo controle?

( ) Sim ( ) Não

**O)** O estudo apresenta valores de marcadores inflamatórios (citocinas e outros marcadores) antes e após as intervenções de creatina?

( ) Sim ( ) Não

**S)** O estudo é um ensaio clínico randomizado e controlado?

( ) Sim ( ) Não

**Artigo 182:** Meta-analysis**of brain metabolite differences in HIV infection.**

Chelala L, O'Connor EE, Barker PB, Zeffiro TA

**P)** Os participantes possuem mais de 18 anos de idade?

( ) Sim (X) Não

"The study is a meta-analysis that included 41 papers dating from 1993 to 2018. While it mentions that "studies comprising subsets of treated and untreated HIV participantse13, e29, the treatment naive groups were excluded" and that "Participants with acute HIV infection were not included in the analysis", it does not specify an age restriction or exclusion criteria for studies involving participants under 18 years old in its detailed methodology for study selection. The objective was to "evaluate the consistency of HIV serostatus effects on brain metabolites", and HIV infection can occur across all age groups, including perinatally. Without an explicit statement that all participants from all included studies were over 18, it cannot be assumed that minors were excluded from the broader scope of studies synthesized in this meta-analysis."

**I)** A intervenção utilizada no estudo é creatina? Vale qualquer formato de administração

( ) Sim ( ) Não

**C)** Teve grupo controle?

( ) Sim ( ) Não

**O)** O estudo apresenta valores de marcadores inflamatórios (citocinas e outros marcadores) antes e após as intervenções de creatina?

( ) Sim ( ) Não

**S)** O estudo é um ensaio clínico randomizado e controlado?

( ) Sim ( ) Não

**Artigo 183: Creatine Alleviates Doxorubicin-Induced Liver Damage by Inhibiting Liver Fibrosis, Inflammation, Oxidative Stress, and Cellular Senescence.**

Aljobaily N, Viereckl MJ, Hydock DS, Aljobaily H, Wu TY, Busekrus R, Jones B, Alberson J, Han Y

**P)** Os participantes possuem mais de 18 anos de idade?

( ) Sim (X) Não

"The study explicitly states that the participants were 'Male Sprague-Dawley rats (n = 60)'. Furthermore, the article's 'Informed Consent Statement' clearly notes: 'Not applicable as our study did not involve human subjects'. Therefore, the participants are not human and, by extension, not over 18 years of age."

**I)** A intervenção utilizada no estudo é creatina? Vale qualquer formato de administração

( ) Sim ( ) Não

**C)** Teve grupo controle?

( ) Sim ( ) Não

**O)** O estudo apresenta valores de marcadores inflamatórios (citocinas e outros marcadores) antes e após as intervenções de creatina?

( ) Sim ( ) Não

**S)** O estudo é um ensaio clínico randomizado e controlado?

( ) Sim ( ) Não

**Artigo 184: Magnetic resonance spectroscopy studies of substance use disorders: Current landscape and potential future directions.**

Kohut SJ, Kaufman MJ

**P)** Os participantes possuem mais de 18 anos de idade?

( ) Sim (X) Não

"This article is a "review" that synthesizes findings from over 200 in vivo magnetic resonance spectroscopy (MRS) studies on substance use disorders. The review itself does not involve direct participants, but rather summarizes existing research. Critically, it refers to studies that included "youths with family histories of substance use disorders", indicating that some of the populations covered by the reviewed literature were not exclusively over 18 years old. Therefore, it cannot be stated that all participants across all studies discussed in this review were over 18."

**I)** A intervenção utilizada no estudo é creatina? Vale qualquer formato de administração

( ) Sim ( ) Não

**C)** Teve grupo controle?

( ) Sim ( ) Não

**O)** O estudo apresenta valores de marcadores inflamatórios (citocinas e outros marcadores) antes e após as intervenções de creatina?

( ) Sim ( ) Não

**S)** O estudo é um ensaio clínico randomizado e controlado?

( ) Sim ( ) Não

**Artigo 185: Dietary supplementation of Ascophylum nodosum improved kidney function of mink challenged with Aleutian mink disease virus.**

Farid AH, Smith NJ

**P)** Os participantes possuem mais de 18 anos de idade?

( ) Sim (X) Não

"The study explicitly states that the participants were 'AMDV-free black **mink** (n = 75)' and specifies them as '75 five-month old female black American **mink** (Neovison vison)'. Therefore, the participants are **animals** (mink), not human subjects, and thus are not over 18 years of age."

**I)** A intervenção utilizada no estudo é creatina? Vale qualquer formato de administração

( ) Sim ( ) Não

**C)** Teve grupo controle?

( ) Sim ( ) Não

**O)** O estudo apresenta valores de marcadores inflamatórios (citocinas e outros marcadores) antes e após as intervenções de creatina?

( ) Sim ( ) Não

**S)** O estudo é um ensaio clínico randomizado e controlado?

( ) Sim ( ) Não

**Artigo 186: Effect of norepinephrine combined with sodium phosphocreatine on cardiac function and prognosis of patients with septic shock.**

Kang D, Yu J, Xia J, Li X, Wang H, Zhao Y

**P)** Os participantes possuem mais de 18 anos de idade?

(X) Sim ( ) Não

"The study explicitly states its inclusion criteria: '(2) age of **more than 18 years old**'. Furthermore, the demographic data table shows the mean age for all groups ranging from 55.89 to 57.05 years, confirming that all participants were adults."

**I)** A intervenção utilizada no estudo é creatina? Vale qualquer formato de administração

(X) Sim ( ) Não

"The intervention investigated in the study is 'Sodium phosphocreatine (SP)'. The article describes SP as 'a new energy supplement' and states that 'SP (2 g/day) was used by intravenous drip'. Phosphocreatine is a direct derivative and rapidly interconvertible form of creatine, playing a critical role in cellular energy buffering via the creatine kinase system. Therefore, it falls under 'any format of administration' of creatine."

**C)** Teve grupo controle?

(X) Sim ( ) Não

"The study included four groups: 'NE-1 h group', 'NE-2 h group', 'NE-3 h group', and 'NE + SP group'. Specifically, the 'NE-2 h group' (norepinephrine administration at 2 hours after fluid infusion) served as a control for the 'NE + SP group' (norepinephrine combined with sodium phosphocreatine, also with NE administration at 2 hours after fluid infusion). The study directly compared outcomes between these groups, such as cardiac function changes and 28-day survival."

**O)** O estudo apresenta valores de marcadores inflamatórios (citocinas e outros marcadores) antes e após as intervenções de creatina?

( ) Sim (X) Não

"The study measured 'serum levels of cardiac troponin I (cTnI) and B-type natriuretic peptide (BNP)'. While it mentions that 'inflammatory cytokines' can contribute to myocardial dysfunction in septic shock, the study *did not report* direct measurements of general inflammatory markers like cytokines (e.g., TNF-α, IL-1β, IL-6) or C-reactive protein (CRP) before and after the interventions with sodium phosphocreatine. The tables presenting results (Table 2 and Table 3) only list cardiac function indicators (BNP, cTnI, EF, PAR) and hemodynamic parameters (SBP, DAP, MAP)."

**S)** O estudo é um ensaio clínico randomizado e controlado?

( ) Sim ( ) Não

**Artigo 187: Phosphocreatine Promotes Osteoblastic Activities in H(2)O(2)-Induced MC3T3-E1**Cells**by Regulating SIRT1/FOXO1/PGC-1α Signaling Pathway.**

Jing Z, Wang C, Wen S, Jin Y, Meng Q, Liu Q, Wu J, Sun H, Liu M

**P)** Os participantes possuem mais de 18 anos de idade?

( ) Sim (X) Não

"The study explicitly states that it investigated the effects of Phosphocreatine (PCr) on 'H2O2-induced MC3T3-E1 cells'. It further clarifies that 'the MC3T3-E1 cell line is an osteoblastic cell line used for mimicking the process of osteoblastic formation'. The 'Human and Animal Rights' section also states 'Not applicable'. Therefore, the study was conducted **in vitro using a cell line**, not on human participants or animals, and thus the concept of age does not apply."

**I)** A intervenção utilizada no estudo é creatina? Vale qualquer formato de administração

( ) Sim ( ) Não

**C)** Teve grupo controle?

( ) Sim ( ) Não

**O)** O estudo apresenta valores de marcadores inflamatórios (citocinas e outros marcadores) antes e após as intervenções de creatina?

( ) Sim ( ) Não

**S)** O estudo é um ensaio clínico randomizado e controlado?

( ) Sim ( ) Não

**Artigo 188: Creatine Supply Attenuates Ischemia-Reperfusion Injury in Lung Transplantation in**Rats**.**

Almeida FM, Battochio AS, Napoli JP, Alves KA, Balbin GS, Oliveira-Junior M, Moriya HT, Pego-Fernandes PM, Vieira RP, Pazetti R

**P)** Os participantes possuem mais de 18 anos de idade?

( ) Sim (X) Não

"The study explicitly states that the participants were 'Sixty-four Sprague Dawley adult male rats (400 g)'. Therefore, the participants are **animals (rats)**, not human subjects, and thus are not over 18 years of age."

**I)** A intervenção utilizada no estudo é creatina? Vale qualquer formato de administração

( ) Sim ( ) Não

**C)** Teve grupo controle?

( ) Sim ( ) Não

**O)** O estudo apresenta valores de marcadores inflamatórios (citocinas e outros marcadores) antes e após as intervenções de creatina?

( ) Sim ( ) Não

**S)** O estudo é um ensaio clínico randomizado e controlado?

( ) Sim ( ) Não

**Artigo 189: Molecular correlates of MRS-based (31) phosphocreatine muscle resynthesis rate in healthy adults.**

Darpolor MM, Singh M, Covington J, Hanet S, Ravussin E, Carmichael OT

**P)** Os participantes possuem mais de 18 anos de idade?

(X) Sim ( ) Não

"The study explicitly states its participants were '10 men and 18 women, **ages 20–50 years**'. All individuals within this age range are over 18 years old."

**I)** A intervenção utilizada no estudo é creatina? Vale qualquer formato de administração

( ) Sim (X) Não

"The study focuses on 'Molecular correlates of MRS-based 31phosphocreatine muscle resynthesis rate' and investigates the 'PCr resynthesis rate from 31P-MRS spectra collected from healthy adults'. It examines the relationship between this **endogenous metabolic process** and molecular markers, rather than utilizing creatine or phosphocreatine as an administered intervention."

**C)** Teve grupo controle?

( ) Sim ( ) Não

**O)** O estudo apresenta valores de marcadores inflamatórios (citocinas e outros marcadores) antes e após as intervenções de creatina?

( ) Sim ( ) Não

**S)** O estudo é um ensaio clínico randomizado e controlado?

( ) Sim ( ) Não

**Artigo 190: s-Ethyl cysteine, an amino acid derivative, attenuated cisplatin induced nephrotoxicity.**

Kuo HL, Mong MC, Chen HC, Wang ZH, Yin MC

**P)** Os participantes possuem mais de 18 anos de idade?

( ) Sim (X) Não

"The study explicitly states its participants were **'Male BALB/c mice'**. It also specifies their age as **'8-week old'**. Therefore, the study was conducted on **animals (mice)**, not human subjects, and thus the concept of being over 18 years old does not apply."

**I)** A intervenção utilizada no estudo é creatina? Vale qualquer formato de administração

( ) Sim ( ) Não

**C)** Teve grupo controle?

( ) Sim ( ) Não

**O)** O estudo apresenta valores de marcadores inflamatórios (citocinas e outros marcadores) antes e após as intervenções de creatina?

( ) Sim ( ) Não

**S)** O estudo é um ensaio clínico randomizado e controlado?

( ) Sim ( ) Não

**Artigo 191: Metabolites of neuroinflammation relate to neuropathic pain after spinal cord injury.**

Pfyffer D, Wyss PO, Huber E, Curt A, Henning A, Freund P

**P)** Os participantes possuem mais de 18 anos de idade?

(X) Sim ( ) Não

"The study explicitly states that the recruited participants included 'Fourteen patients with SCI with NP (12 men, **age [mean ± SD] 52.2 ± 10.5 years**), 10 pain-free patients with SCI (10 men, **age 50.0 ± 10.3 y**), and 21 healthy control participants (18 men, **age 46.0 ± 11.2 y**)'. All reported mean ages are well above 18 years, and the individual patient data in Table shows the youngest patient was 19 years old at injury, confirming all participants were adults."

**I)** A intervenção utilizada no estudo é creatina? Vale qualquer formato de administração

( ) Sim (X) Não

"The study's primary objective was to 'non-invasively quantify metabolite levels' in the spinal cord using 'Magnetic Resonance (MR) Spectroscopy (MRS)' to investigate 'metabolite ratios associated with neuroinflammation... and neurodegeneration'. While creatine is a metabolite whose levels are measured as part of the spectroscopic analysis ('a basis set... including... creatine'), **it is not administered as an intervention** in this study. The study focuses on measuring endogenous biochemical profiles."

**C)** Teve grupo controle?

( ) Sim ( ) Não

**O)** O estudo apresenta valores de marcadores inflamatórios (citocinas e outros marcadores) antes e após as intervenções de creatina?

( ) Sim ( ) Não

**S)** O estudo é um ensaio clínico randomizado e controlado?

( ) Sim ( ) Não

**Artigo 192: Skeletal Muscle Mitochondrial Dysfunction Is Present in Patients with CKD before Initiation of Maintenance Hemodialysis.**

Gamboa JL, Roshanravan B, Towse T, Keller CA, Falck AM, Yu C, Frontera WR, Brown NJ, Ikizler TA

**P)** Os participantes possuem mais de 18 anos de idade?

( ) Sim ( ) Não [*Excluir*]

**I)** A intervenção utilizada no estudo é creatina? Vale qualquer formato de administração

( ) Sim ( ) Não [*Excluir*]

**C)** Teve grupo controle?

( ) Sim ( ) Não [*Excluir*]

**O)** O estudo apresenta valores de marcadores inflamatórios (citocinas e outros marcadores) antes e após as intervenções de creatina?

( ) Sim ( ) Não [*Excluir*]

**S)** O estudo é um ensaio clínico randomizado e controlado?

( ) Sim ( ) Não [*Excluir*]

**Artigo 193: COVID-19 infection alters kynurenine and fatty acid metabolism, correlating with IL-6 levels and renal status.**

Thomas T, Stefanoni D, Reisz JA, Nemkov T, Bertolone L, Francis RO, Hudson KE, Zimring JC, Hansen KC, Hod EA, Spitalnik SL, D'Alessandro A

**P)** Os participantes possuem mais de 18 anos de idade?

( ) Sim (X) Não

"The study reports the mean age of participants in the control group as '37.8 ± 11.6 years old'. While the average is over 18, considering the standard deviation, some individuals in this group **could potentially be younger than 18 years old** (e.g., 37.8 - 2 * 11.6 = 14.6 years). The study does not explicitly state an age minimum of 18 years or older for all participants as an inclusion criterion, unlike other studies."

**I)** A intervenção utilizada no estudo é creatina? Vale qualquer formato de administração

( ) Sim ( ) Não

**C)** Teve grupo controle?

( ) Sim ( ) Não

**O)** O estudo apresenta valores de marcadores inflamatórios (citocinas e outros marcadores) antes e após as intervenções de creatina?

( ) Sim ( ) Não

**S)** O estudo é um ensaio clínico randomizado e controlado?

( ) Sim ( ) Não

**Artigo 194: Supplement Use in Patients Undergoing Anterior Cruciate Ligament Reconstruction: A**Systematic Review**.**

Greif DN, Emerson CP, Allegra P, Arizpe A, Mansour KL, Cade WH 2nd, Baraga MG

**P)** Os participantes possuem mais de 18 anos de idade?

(X) Sim ( ) Não

"The systematic review analyzed studies where participants' age ranges were consistently stated as 18 years and older, for example, '18-35', '18-55', '18-40', 'Mean: 24; Range: 18-45', '18-45', and '18-50'."

**I)** A intervenção utilizada no estudo é creatina? Vale qualquer formato de administração

(X) Sim ( ) Não

"The systematic review explicitly states that 'One study assessed creatine as a supplement' and dedicates a section to 'Creatine-Based Supplementation', detailing the findings of an included RCT that evaluated creatine."

**C)** Teve grupo controle?

(X) Sim ( ) Não

"The systematic review states that all included studies were 'prospective RCTs', which by definition include a control group. Furthermore, Table 1 of the review explicitly lists a 'Control Group' for each study examined."

**O)** O estudo apresenta valores de marcadores inflamatórios (citocinas e outros marcadores) antes e após as intervenções de creatina?

( ) Sim (X) Não

"The systematic review notes that 'Only 1 RCT... evaluated creatine as a potential supplement'. The reported outcomes for this creatine-based study (Tyler et al., 2004) included 'Knee Outcome Survey responses, body fat, goniometric knee range of motion, isokinetic knee strength/power, and hip strength' but did **not** include measurements of inflammatory markers. While other supplement types (vitamins) reviewed did assess inflammatory markers, this was not done for the creatine intervention."

**S)** O estudo é um ensaio clínico randomizado e controlado?

( ) Sim ( ) Não

**Artigo 195: Hippocampal changes in inflammasomes, apoptosis, and MEMRI after radiation-induced brain injury in juvenile**rats**.**

Yang J, Gao J, Han D, Li Q, Liao C, Li J, Wang R, Luo Y

**P)** Os participantes possuem mais de 18 anos de idade?

( ) Sim (X) Não

"The study explicitly states that the participants were **'Four-week-old male Sprague-Dawley rats'**. Therefore, the study was conducted on **animals (rats)**, not human subjects, and the concept of being over 18 years old does not apply."

**I)** A intervenção utilizada no estudo é creatina? Vale qualquer formato de administração

( ) Sim ( ) Não

**C)** Teve grupo controle?

( ) Sim ( ) Não

**O)** O estudo apresenta valores de marcadores inflamatórios (citocinas e outros marcadores) antes e após as intervenções de creatina?

( ) Sim ( ) Não

**S)** O estudo é um ensaio clínico randomizado e controlado?

( ) Sim ( ) Não

**Artigo 196: Differences in the serum metabolome and lipidome identify potential biomarkers for seronegative rheumatoid arthritis versus psoriatic arthritis.**

Souto-Carneiro M, Tóth L, Behnisch R, Urbach K, Klika KD, Carvalho RA, Lorenz HM

**P)** Os participantes possuem mais de 18 anos de idade?

(X) Sim ( ) Não

"The study enrolled patients with reported ages that are all well above 18 years, as indicated in Table 1: 'Age (minimum–maximum in years) negRA (n=49): 64.2 (32–83) PsA (n=73): 56.2 (30–78)'. The minimum age observed in the study was 30 years."

**I)** A intervenção utilizada no estudo é creatina? Vale qualquer formato de administração

( ) Sim (X) Não

"This study aimed to identify potential biomarkers by analyzing 'Differences in the serum metabolome and lipidome' from patients with seronegative rheumatoid arthritis and psoriatic arthritis. While **creatine is listed as an organic compound whose concentration was measured** ('organic compounds: acetate, creatine, lactate and choline'), it was part of the metabolomic profile analyzed, not an intervention administered to the participants."

**C)** Teve grupo controle?

( ) Sim ( ) Não

**O)** O estudo apresenta valores de marcadores inflamatórios (citocinas e outros marcadores) antes e após as intervenções de creatina?

( ) Sim ( ) Não

**S)** O estudo é um ensaio clínico randomizado e controlado?

( ) Sim ( ) Não

**Artigo 197: Neurometabolic Remodeling in Chronic Hiv Infection: a Five-Year Follow-up Multi-Voxel Mrs Study.**

Boban J, Thurnher MM, Brkic S, Lendak D, Bugarski Ignjatovic V, Todorovic A, Kozic D

**P)** Os participantes possuem mais de 18 anos de idade?

(X) Sim ( ) Não

"The study included '19 chronically infected HIV+ male patients on cART, **mean age 45.16 ± 11.47**'. This indicates that all participants were adults, with the youngest implied age well over 18, given the mean and standard deviation."

**I)** A intervenção utilizada no estudo é creatina? Vale qualquer formato de administração

( ) Sim (X) Não

"The study is a **longitudinal magnetic resonance spectroscopy (MRS) study** that assessed changes in neurometabolite profiles, including 'creatine (Cr)' levels, in HIV-positive subjects over a five-year period. Creatine was measured as an endogenous brain metabolite ('creatine (Cr)', 'creatine kinase'), and ratios involving it (e.g., NAA/Cr, Cho/Cr, mI/Cr) were analyzed, but it was not administered as an intervention or supplement to the participants."

**C)** Teve grupo controle?

( ) Sim ( ) Não

**O)** O estudo apresenta valores de marcadores inflamatórios (citocinas e outros marcadores) antes e após as intervenções de creatina?

( ) Sim ( ) Não

**S)** O estudo é um ensaio clínico randomizado e controlado?

( ) Sim ( ) Não

**Artigo 198: Slc6a8-Mediated Creatine Uptake and Accumulation Reprogram Macrophage Polarization via Regulating Cytokine Responses.**

Ji L, Zhao X, Zhang B, Kang L, Song W, Zhao B, Xie W, Chen L, Hu X

**P)** Os participantes possuem mais de 18 anos de idade?

( ) Sim (X) Não

"The study was conducted on **mice** ('WT and Slc6a8/y mice', 'Lyz2cre/cre and Lyz2cre/cre Slc6a8fl/y mice', 'Listeria monocytogeneswere intravenously administrated', 'Mice were treated with CCl4 to induce liver fibrosis') and their **peritoneal macrophages** ('peritoneal macrophages fromWT and Slc6a8/y mice', 'WT peritoneal macrophages were treated with the creatine synthesis inhibitor ornithine'). Therefore, the participants are not human and the concept of being over 18 years old does not apply."

**I)** A intervenção utilizada no estudo é creatina? Vale qualquer formato de administração

( ) Sim ( ) Não

**C)** Teve grupo controle?

( ) Sim ( ) Não

**O)** O estudo apresenta valores de marcadores inflamatórios (citocinas e outros marcadores) antes e após as intervenções de creatina?

( ) Sim ( ) Não

**S)** O estudo é um ensaio clínico randomizado e controlado?

( ) Sim ( ) Não

**Artigo 199: Cross-talk between guanidinoacetate neurotoxicity, memory and possible neuroprotective role of creatine.**

Marques EP, Ferreira FS, Santos TM, Prezzi CA, Martins LAM, Bobermin LD, Quincozes-Santos A, Wyse ATS

**P)** Os participantes possuem mais de 18 anos de idade?

( ) Sim (X) Não

"The study was conducted on **'Male Wistar rats'** that were **'fifty-one-day-old'** when pretreatment began and **'sixty-days-old'** at the time of GAA administration. Therefore, the participants were **animals**, not human subjects, and the concept of being over 18 years old is not applicable."

**I)** A intervenção utilizada no estudo é creatina? Vale qualquer formato de administração

( ) Sim ( ) Não

**C)** Teve grupo controle?

( ) Sim ( ) Não

**O)** O estudo apresenta valores de marcadores inflamatórios (citocinas e outros marcadores) antes e após as intervenções de creatina?

( ) Sim ( ) Não

**S)** O estudo é um ensaio clínico randomizado e controlado?

( ) Sim ( ) Não

**Artigo 200: Dietary protein and exercise for preservation of lean mass and perspectives on type 2 diabetes prevention.**

de Sousa MV, da Silva Soares DB, Caraça ER, Cardoso R

**P)** Os participantes possuem mais de 18 anos de idade?

(X) Sim ( ) Não

"The review discusses studies that include 'middle-aged and older adults', 'elderly population', 'participants over 65 years of age', and 'fragile elderly subjects', indicating that all subjects in the reviewed studies were adults."

**I)** A intervenção utilizada no estudo é creatina? Vale qualquer formato de administração

( ) Sim (X) Não

"This article is explicitly identified as a 'Minireview Highlight article'. It discusses the effects of various dietary interventions, including creatine, by reviewing existing studies, such as 'The effects of creatine supplementation on glycemic con-trol of patients with T2D were evaluated by Gualano et al.68'. However, this review itself does not conduct an intervention but rather summarizes findings from other studies."

**C)** Teve grupo controle?

( ) Sim ( ) Não

**O)** O estudo apresenta valores de marcadores inflamatórios (citocinas e outros marcadores) antes e após as intervenções de creatina?

( ) Sim ( ) Não

**S)** O estudo é um ensaio clínico randomizado e controlado?

( ) Sim ( ) Não

**Artigo 201: miR-30c-5p Reduces Renal Ischemia-Reperfusion Involving Macrophage.**

Zhang C, Yu S, Zheng B, Liu D, Wan F, Ma Y, Wang J, Gao Z, Shan Z

**P)** Os participantes possuem mais de 18 anos de idade?

( ) Sim (X) Não

O estudo foi conduzido em ratos Sprague Dawley. A seção "Material and Methods" (Material e Métodos) do artigo especifica que "Sprague Dawley rats were purchased from Guangdong Medical Laboratory Animal Center (Guangzhou, Guangdong, China)". Portanto, os participantes não são humanos e, consequentemente, não têm mais de 18 anos de idade.

**I)** A intervenção utilizada no estudo é creatina? Vale qualquer formato de administração

( ) Sim ( ) Não

**C)** Teve grupo controle?

( ) Sim ( ) Não

**O)** O estudo apresenta valores de marcadores inflamatórios (citocinas e outros marcadores) antes e após as intervenções de creatina?

( ) Sim ( ) Não

**S)** O estudo é um ensaio clínico randomizado e controlado?

( ) Sim ( ) Não

**Artigo 202: Renal effects of exendin-4 in an**animal**model of brain death.**

Lemos NE, Dieter C, Carlessi R, Rheinheimer J, Brondani LA, Leitão CB, Bauer AC, Crispim D

**P)** Os participantes possuem mais de 18 anos de idade?

( ) Sim (X) Não

O estudo foi conduzido em ratos Wistar, não em humanos. A seção "Materials and methods" (Materiais e métodos) do artigo especifica: "Male Wistar rats fed standard laboratory diet ad libitum, weighing 300–350 g, were used in the study". Portanto, os participantes não são humanos e, consequentemente, não possuem mais de 18 anos de idade.

**I)** A intervenção utilizada no estudo é creatina? Vale qualquer formato de administração

( ) Sim ( ) Não

**C)** Teve grupo controle?

( ) Sim ( ) Não

**O)** O estudo apresenta valores de marcadores inflamatórios (citocinas e outros marcadores) antes e após as intervenções de creatina?

( ) Sim ( ) Não

**S)** O estudo é um ensaio clínico randomizado e controlado?

( ) Sim ( ) Não

**Artigo 203: Phosphocreatine Attenuates Isoproterenol-Induced Cardiac Fibrosis and Cardiomyocyte Apoptosis.**

Dai H, Chen L, Gao D, Fei A

**P)** Os participantes possuem mais de 18 anos de idade?

( ) Sim (X) Não

"The study was conducted on rats. The 'Methods' section specifies: 'In this study, we further verify the effect of PCr in vivo on cardiac fibrosis and cardiomyocyte apoptosis in ISO-induced rat model.' and 'In our study, adult male rats were treated with ISO (50 mg/kg/d) for two weeks to establish the model of cardiac fibrosis.'. Therefore, the participants are not human and, consequently, do not possess more than 18 years of age."

**I)** A intervenção utilizada no estudo é creatina? Vale qualquer formato de administração

( ) Sim ( ) Não

**C)** Teve grupo controle?

( ) Sim ( ) Não

**O)** O estudo apresenta valores de marcadores inflamatórios (citocinas e outros marcadores) antes e após as intervenções de creatina?

( ) Sim ( ) Não

**S)** O estudo é um ensaio clínico randomizado e controlado?

( ) Sim ( ) Não

**Artigo 204: Evidence of widespread metabolite abnormalities in Myalgic encephalomyelitis/chronic fatigue syndrome: assessment with whole-brain magnetic resonance spectroscopy.**

Mueller C, Lin JC, Sheriff S, Maudsley AA, Younger JW

**P)** Os participantes possuem mais de 18 anos de idade?

(X) Sim ( ) Não

"Control participants were also aged between 18–55 years, were matched within two years of participants with ME/CFS".

**I)** A intervenção utilizada no estudo é creatina? Vale qualquer formato de administração

( ) Sim (X) Não

"Magnetic Resonance Spectroscopy (MRS) is suitable for measuring brain metabolites linked to". The study focuses on assessing "widespread metabolite abnormalities" in Myalgic Encephalomyelitis/Chronic Fatigue Syndrome patients, measuring substances like "CHO, MI, LAC, higher brain temperature, and lower NAA" and "NAA/CR... CHO/CR... LAC/CR... MI/CR". Creatina (CR) é um dos metabólitos avaliados no cérebro, não uma intervenção ou tratamento administrado no estudo.

**C)** Teve grupo controle?

( ) Sim ( ) Não

**O)** O estudo apresenta valores de marcadores inflamatórios (citocinas e outros marcadores) antes e após as intervenções de creatina?

( ) Sim ( ) Não

**S)** O estudo é um ensaio clínico randomizado e controlado?

( ) Sim ( ) Não

**Artigo 205: The Effect of a High-Dose Vitamin B Multivitamin Supplement on the Relationship between Brain Metabolism and Blood Biomarkers of Oxidative Stress: A**Randomized**Control**Trial**.**

Ford TC, Downey LA, Simpson T, McPhee G, Oliver C, Stough C

**P)** Os participantes possuem mais de 18 anos de idade?

(X) Sim ( ) Não

"The study enrolled healthy adults aged 30 to 65 years."

**I)** A intervenção utilizada no estudo é creatina? Vale qualquer formato de administração

( ) Sim (X) Não

"The intervention used in the study was a '6-month high-dose B-group vitamin supplementation', specifically a 'high-dose B vitamin multivitamin supplementation'. Creatine was a measured metabolite, not the intervention administered."

**C)** Teve grupo controle?

( ) Sim ( ) Não

**O)** O estudo apresenta valores de marcadores inflamatórios (citocinas e outros marcadores) antes e após as intervenções de creatina?

( ) Sim ( ) Não

**S)** O estudo é um ensaio clínico randomizado e controlado?

( ) Sim ( ) Não

**Artigo 206: Diabetic Nephropathy Can Be Treated with Calcium Dobesilate by Alleviating the Chronic Inflammatory State and Improving Endothelial Cell Function.**

Zhou Y, Qi C, Li S, Shao X, Mou S, Ni Z

**P)** Os participantes possuem mais de 18 anos de idade?

(X) Sim ( ) Não

"Selection criteria for DKD group were: type 2 DM patients diagnosed in accordance with the 2014 American Diabetes Association (ADA) diagnosis and treatment guidelines; and patients with a albumin/creatinine ratio (ACR)≥30 mg/g and 24 h urinary protein between 150 mg and 2g in random morning urine samples at least twice in the past 3 months; and patients with the presence of diabetic retinopathy; and patients with no previous history of hypertension and with blood pressure≤140/90 mmHg; and patients with a GFR≥90 ml/min/1.73 m2; and **patients aged 18–70 years**."

**I)** A intervenção utilizada no estudo é creatina? Vale qualquer formato de administração

( ) Sim (X) Não

"The intervention used in the study was 'Calcium dobesilate (CaD)', administered orally. Specifically, 'the treatment group (500 mg of CaD, administered orally, 3 times per day)'." Creatine or its derivatives were not the intervention.

**C)** Teve grupo controle?

( ) Sim ( ) Não

**O)** O estudo apresenta valores de marcadores inflamatórios (citocinas e outros marcadores) antes e após as intervenções de creatina?

( ) Sim ( ) Não

**S)** O estudo é um ensaio clínico randomizado e controlado?

( ) Sim ( ) Não

**Artigo 207: Nephroprotective Effect of Essential Oils from Ginger (Zingiber officinale) and Turmeric (Curcuma longa) Rhizomes against Cadmium-induced Nephrotoxicity in**Rats**.**

Akinyemi AJ, Faboya OL, Paul AA, Olayide I, Faboya OA, Oluwasola TA

**P)** Os participantes possuem mais de 18 anos de idade?

( ) Sim (X) Não

"The study was conducted on rats. The '2.4 Animals' section states: '**Forty-eight adult male albino rats of twelve weeks old** were obtained from the animal breeding unit at College of Medicine, Afe Babalola University, Nigeria'. Therefore, the participants are not human and do not possess more than 18 years of age".

**I)** A intervenção utilizada no estudo é creatina? Vale qualquer formato de administração

( ) Sim ( ) Não

**C)** Teve grupo controle?

( ) Sim ( ) Não

**O)** O estudo apresenta valores de marcadores inflamatórios (citocinas e outros marcadores) antes e após as intervenções de creatina?

( ) Sim ( ) Não

**S)** O estudo é um ensaio clínico randomizado e controlado?

( ) Sim ( ) Não

**Artigo 208: White matter damage, neuroinflammation, and neuronal integrity in HAND.**

Alakkas A, Ellis RJ, Watson CW, Umlauf A, Heaton RK, Letendre S, Collier A, Marra C, Clifford DB, Gelman B, Sacktor N, Morgello S, Simpson D, McCutchan JA, Kallianpur A, Gianella S, Marcotte T, Grant I, Fennema-Notestine C

**P)** Os participantes possuem mais de 18 anos de idade?

(X) Sim ( ) Não

"Participants were mostly men (81%) and had a **mean age of 44.13 years**".

**I)** A intervenção utilizada no estudo é creatina? Vale qualquer formato de administração

( ) Sim (X) Não

The study primarily focuses on assessing neurochemical metabolites and structural magnetic resonance imaging. It states: "Using single-voxel MRS, we **estimated metabolites** in frontal gray matter (FGM) and frontal white matter (FWM) and basal ganglia (BG) regions". Creatine is mentioned as one of the metabolites measured ("MND had lower FWM **creatine**" and "While we found significantly lower **creatine** in frontal white matter in MND compared to NU"), not as an intervention or treatment administered to the participants.

**C)** Teve grupo controle?

( ) Sim ( ) Não

**O)** O estudo apresenta valores de marcadores inflamatórios (citocinas e outros marcadores) antes e após as intervenções de creatina?

( ) Sim ( ) Não

**S)** O estudo é um ensaio clínico randomizado e controlado?

( ) Sim ( ) Não

**Artigo 209: Correlation between biomarkers of creatine metabolism and serum indicators of peripheral muscle fatigue during exhaustive exercise in active men.**

Stajer V, Vranes M, Ostojic SM

**P)** Os participantes possuem mais de 18 anos de idade?

(X) Sim ( ) Não

"The 'Methods - Participants' section states: 'Eleven physically active healthy **young men (age 23.2 ± 3.7 years**...)'".

**I)** A intervenção utilizada no estudo é creatina? Vale qualquer formato de administração

( ) Sim (X) Não

"The study explicitly states that participants were 'not taking any medication or dietary supplements'. The paper investigates the correlation between biomarkers of creatine metabolism (including creatine itself) and indicators of fatigue *during exhaustive exercise*, meaning creatine was a measured substance, not an intervention."

**C)** Teve grupo controle?

( ) Sim ( ) Não

**O)** O estudo apresenta valores de marcadores inflamatórios (citocinas e outros marcadores) antes e após as intervenções de creatina?

( ) Sim ( ) Não

**S)** O estudo é um ensaio clínico randomizado e controlado?

( ) Sim ( ) Não

**Artigo 210: Nifuroxazide, a STAT3 inhibitor, mitigates inflammatory burden and protects against diabetes-induced nephropathy in**rats**.**

Said E, Zaitone SA, Eldosoky M, Elsherbiny NM

**P)** Os participantes possuem mais de 18 anos de idade?

( ) Sim (X) Não

"The '2.2 Experimental design' section states: '**thirty male Sprague-Dawley rats, weighing 180–230 g**, were obtained from the breeding unit of Holding Company for Biological Products and Vaccines, “VACSERA”, Cairo-Egypt'. Therefore, the participants are not human and do not possess more than 18 years of age."

**I)** A intervenção utilizada no estudo é creatina? Vale qualquer formato de administração

( ) Sim ( ) Não

**C)** Teve grupo controle?

( ) Sim ( ) Não

**O)** O estudo apresenta valores de marcadores inflamatórios (citocinas e outros marcadores) antes e após as intervenções de creatina?

( ) Sim ( ) Não

**S)** O estudo é um ensaio clínico randomizado e controlado?

( ) Sim ( ) Não

**Artigo 211: Cyclosporine A exhibits gender-specific nephrotoxicity in**rats**: Effect on renal tissue inflammation.**

El-Bassossy HM, Eid BG

**P)** Os participantes possuem mais de 18 anos de idade?

( ) Sim (X) Não

"The '2. Methods' section states: '**6 week old Wistar rats (male and female)** were kept in cages'. Therefore, the participants are not human and do not possess more than 18 years of age."

**I)** A intervenção utilizada no estudo é creatina? Vale qualquer formato de administração

( ) Sim ( ) Não

**C)** Teve grupo controle?

( ) Sim ( ) Não

**O)** O estudo apresenta valores de marcadores inflamatórios (citocinas e outros marcadores) antes e após as intervenções de creatina?

( ) Sim ( ) Não

**S)** O estudo é um ensaio clínico randomizado e controlado?

( ) Sim ( ) Não

**Artigo 212: A pilot study of cortical glutathione in youth with depression.**

Freed RD, Hollenhorst CN, Weiduschat N, Mao X, Kang G, Shungu DC, Gabbay V

**P)** Os participantes possuem mais de 18 anos de idade?

( ) Sim (X) Não

"The 'Human subjects' section states that 'participants age **from 12–21**, were recruited'. It also clarifies that 'Participants **18 years of age and older provided written informed consent**, and those under age 18 provided assent and a parent or guardian gave signed informed consent'. This indicates that individuals under 18 years of age were included in the study."

**I)** A intervenção utilizada no estudo é creatina? Vale qualquer formato de administração

( ) Sim ( ) Não

**C)** Teve grupo controle?

( ) Sim ( ) Não

**O)** O estudo apresenta valores de marcadores inflamatórios (citocinas e outros marcadores) antes e após as intervenções de creatina?

( ) Sim ( ) Não

**S)** O estudo é um ensaio clínico randomizado e controlado?

( ) Sim ( ) Não

**Artigo 213: CDP-choline circumvents mercury-induced mitochondrial damage and renal dysfunction.**

Buelna-Chontal M, Franco M, Hernández-Esquivel L, Pavón N, Rodríguez-Zavala JS, Correa F, Jasso R, Pichardo-Ramos G, Santamaría J, González-Pacheco H, Soto V, Díaz-Ruíz JL, Chávez E

**P)** Os participantes possuem mais de 18 anos de idade?

( ) Sim (X) Não

"The 'Material and methods' section states: '**Male Wistar rats weighing 250–300 g** received ip injections of HgCl2'. Therefore, the participants are not human and do not possess more than 18 years of age."

**I)** A intervenção utilizada no estudo é creatina? Vale qualquer formato de administração

( ) Sim ( ) Não

**C)** Teve grupo controle?

( ) Sim ( ) Não

**O)** O estudo apresenta valores de marcadores inflamatórios (citocinas e outros marcadores) antes e após as intervenções de creatina?

( ) Sim ( ) Não

**S)** O estudo é um ensaio clínico randomizado e controlado?

( ) Sim ( ) Não

**Artigo 214: Monitoring urinary orosomucoid in patients undergoing cardiac surgery: A promising novel inflammatory marker.**

Kustán P, Szirmay B, Kőszegi T, Ludány A, Kovács GL, Miseta A, Mühl D, Németh B, Kiss I, Németh Á, Szabados S, Ajtay Z

**P)** Os participantes possuem mais de 18 anos de idade?

(X) Sim ( ) Não

"The '2. Methods - 2.1. Study design and sampling' section states: 'Inclusion criteria were **> 18 years of age** and lack of underlying renal disease at admission...'".

**I)** A intervenção utilizada no estudo é creatina? Vale qualquer formato de administração

( ) Sim (X) Não

"The study's title and abstract clearly indicate its focus is on 'Monitoring urinary orosomucoid in patients undergoing cardiac surgery'. The intervention investigated is the **cardiac surgery itself** and its impact on inflammatory markers, with no mention of creatine or any of its forms being administered as a treatment or intervention."

**C)** Teve grupo controle?

( ) Sim ( ) Não

**O)** O estudo apresenta valores de marcadores inflamatórios (citocinas e outros marcadores) antes e após as intervenções de creatina?

( ) Sim ( ) Não

**S)** O estudo é um ensaio clínico randomizado e controlado?

( ) Sim ( ) Não

**Artigo 215: Proton MR spectroscopy of lesion evolution in multiple sclerosis: Steady-state metabolism and its relationship to conventional imaging.**

Kirov II, Liu S, Tal A, Wu WE, Davitz MS, Babb JS, Rusinek H, Herbert J, Gonen O

**P)** Os participantes possuem mais de 18 anos de idade?

(X) Sim ( ) Não

"The 'Human Subjects' section states: 'Eighteen patients... The inclusion criteria were MS diagnosis, no MRI contraindications, **ability to provide informed consent**...'. Providing informed consent typically requires participants to be of legal adult age (18 years or older). Additionally, Table I presents examples of participant ages, such as 'Patient ID 1 M 38' and 'Patient ID 2 F 27', which are all above 18 years."

**I)** A intervenção utilizada no estudo é creatina? Vale qualquer formato de administração

( ) Sim (X) Não

"The study's abstract states: 'We used **proton MR spectroscopy to study the evolution of N-acetyl-aspartate (NAA), creatine (Cr), choline (Cho), and myo-inositol (mI)** in pre-lesional tissue, persistent and transient new lesions, as well as in chronic lesions...'. Creatine (Cr) is mentioned as a metabolite that was measured ('creatine (Cr) levels are linked with energy metabolism and glial proliferation'), not as an administered intervention or treatment."

**C)** Teve grupo controle?

( ) Sim ( ) Não

**O)** O estudo apresenta valores de marcadores inflamatórios (citocinas e outros marcadores) antes e após as intervenções de creatina?

( ) Sim ( ) Não

**S)** O estudo é um ensaio clínico randomizado e controlado?

( ) Sim ( ) Não

**Artigo 216: Roles of the Exogenous H2S-Mediated SR-A Signaling Pathway in Renal Ischemia/ Reperfusion Injury in Regulating Endoplasmic Reticulum Stress-Induced Autophagy in a**Rat**Model.**

Ling Q, Yu X, Wang T, Wang SG, Ye ZQ, Liu JH

**P)** Os participantes possuem mais de 18 anos de idade?

( ) Sim (X) Não

"The 'Materials and Methods - Subjects and grouping' section clearly states: 'A total of **48 rats (5 to 6 months of age, weighing from 200 to 240 g)**... were provided by Shanghai Research Center for Southern Model Organisms.'. Therefore, the participants are not human and are not over 18 years of age."

**I)** A intervenção utilizada no estudo é creatina? Vale qualquer formato de administração

( ) Sim ( ) Não

**C)** Teve grupo controle?

( ) Sim ( ) Não

**O)** O estudo apresenta valores de marcadores inflamatórios (citocinas e outros marcadores) antes e após as intervenções de creatina?

( ) Sim ( ) Não

**S)** O estudo é um ensaio clínico randomizado e controlado?

( ) Sim ( ) Não

**Artigo 217: Serum Endocan as a Predictive Marker for Decreased Urine Volume in Peritoneal Dialysis Patients.**

Oka S, Obata Y, Sato S, Torigoe K, Sawa M, Abe S, Muta K, Ota Y, Kitamura M, Kawasaki S, Hirose M, Uramatsu T, Mukae H, Nishino T

**P)** Os participantes possuem mais de 18 anos de idade?

( ) Sim (X) Não

"The provided excerpts for Artigo 217 do not explicitly state the age of the participants or confirm that they are over 18 years of age. The 'Material and Methods - Patient selection' section mentions: 'This longitudinal, observational cohort study included **21 PD patients** who underwent peritoneal equilibration test...'. Without a direct statement or clear age examples in the provided text, it cannot be confirmed that all participants are over 18 years old."

**I)** A intervenção utilizada no estudo é creatina? Vale qualquer formato de administração

( ) Sim ( ) Não

**C)** Teve grupo controle?

( ) Sim ( ) Não

**O)** O estudo apresenta valores de marcadores inflamatórios (citocinas e outros marcadores) antes e após as intervenções de creatina?

( ) Sim ( ) Não

**S)** O estudo é um ensaio clínico randomizado e controlado?

( ) Sim ( ) Não

**Artigo 218: HIV-associated neurodegeneration and neuroimmunity: multivoxel MR spectroscopy study in drug-naïve and treated patients.**

Boban J, Kozic D, Turkulov V, Ostojic J, Semnic R, Lendak D, Brkic S

**P)** Os participantes possuem mais de 18 anos de idade?

(X) Sim ( ) Não

"Table 1, 'Demographic, clinical and immunological parameters of the study participants', lists the age ranges for the groups: 'HIV+ on cART (n = 32) Age, y 41.97 ± 10.12, 44 (**25–61**)', 'HIV+ w/o cART (n = 28) 35.21 ± 7.71, 34.5 (**24–52**)', and 'Controls (n = 50) 36.56 ± 7.44, 36 (**19-53**)'. The minimum age across all groups is 19 years, confirming participants are above 18."

**I)** A intervenção utilizada no estudo é creatina? Vale qualquer formato de administração

( ) Sim (X) Não

"The study's objective is to 'test neurobiochemical changes in normal appearing brain tissue in HIV+ patients receiving and not receiving combined antiretroviral therapy (cART) and healthy controls, using multivoxel MR spectroscopy (mvMRS)'. Creatine (Cr or tCr) is explicitly mentioned as a **measured metabolite** ('tCr (creatine plus phosphocreatine) at 3.0 ppm') and used in ratios (NAA/Cr, Cho/Cr, mI/Cr), but it is not administered as an intervention or treatment in any format."

**C)** Teve grupo controle?

( ) Sim ( ) Não

**O)** O estudo apresenta valores de marcadores inflamatórios (citocinas e outros marcadores) antes e após as intervenções de creatina?

( ) Sim ( ) Não

**S)** O estudo é um ensaio clínico randomizado e controlado?

( ) Sim ( ) Não

**Artigo 219: Cerebral magnetic resonance imaging in quiescent Crohn's disease patients with fatigue.**

van Erp S, Ercan E, Breedveld P, Brakenhoff L, Ghariq E, Schmid S, van Osch M, van Buchem M, Emmer B, van der Grond J, Wolterbeek R, Hommes D, Fidder H, van der Wee N, Huizinga T, van der Heijde D, Middelkoop H, Ronen I, van der Meulen-de Jong A

**P)** Os participantes possuem mais de 18 anos de idade?

(X) Sim ( ) Não

"The 'RESULTS Demographic characteristics' section states: 'In this study, 20 CD patients and 17 healthy controls were age (P = 0.46) and gender matched (P = 0.68). All patients were in clinical remission at study inclusion (mean HBI = 2.16, SD = 1.12), with an **average age of onset at 21.4 years and an IBD disease duration of 8.8 years**.'. This indicates that the Crohn's disease patients, with an average onset age of 21.4 years and an average disease duration of 8.8 years, would be well over 18 years of age at the time of the study (approximately 30.2 years on average). The healthy controls were age-matched to these patients, confirming that all participants were above 18 years old."

**I)** A intervenção utilizada no estudo é creatina? Vale qualquer formato de administração

( ) Sim (X) Não

"The study investigates 'cerebral changes in quiescent CD patients with fatigue using quantitative magnetic resonance imaging (MRI)'. Specifically, it used Magnetic Resonance Spectroscopy (MRS) to **assess neurochemical changes**. Creatine (Cr) is mentioned as a **measured metabolite** in the brain ('the mean ratio of choline (Cho) to creatine (Cr) in the occipital cortex was significantly higher than in controls') but is not administered as an intervention or treatment to the participants."

**C)** Teve grupo controle?

( ) Sim ( ) Não

**O)** O estudo apresenta valores de marcadores inflamatórios (citocinas e outros marcadores) antes e após as intervenções de creatina?

( ) Sim ( ) Não

**S)** O estudo é um ensaio clínico randomizado e controlado?

( ) Sim ( ) Não

**Artigo 220: Establishment of an AUC(0-24) Threshold for Nephrotoxicity Is a Step towards Individualized Vancomycin Dosing for Methicillin-Resistant Staphylococcus aureus Bacteremia.**

Chavada R, Ghosh N, Sandaradura I, Maley M, Van Hal SJ

**P)** Os participantes possuem mais de 18 anos de idade?

(X) Sim ( ) Não

"The 'MATERIALS AND METHODS Study population and vancomycin pharmacokinetics' section explicitly states: '**All adult patients (≥18 years old)** admitted to Liverpool Hospital... were enrolled into this retrospective observational cohort study'. Furthermore, the limitations section clarifies that the results are 'not applicable to pediatric patients', confirming that the study focused solely on adult participants."

**I)** A intervenção utilizada no estudo é creatina? Vale qualquer formato de administração

( ) Sim ( X) Não

"The primary objective of the study was to investigate the relationship between **vancomycin pharmacokinetic (PK) parameters** and acute kidney injury (AKI) in patients receiving **vancomycin therapy** for methicillin-resistant Staphylococcus aureus bacteremia (MRSA-B). Creatinine (serum creatinine) is mentioned as a **marker** used to *define* AKI ('AKI (defined as serum creatinine of ≥0.5 mg/liter and a 50% increase from baseline)'), and creatinine clearance is used in pharmacokinetic calculations, but creatine itself is not administered as an intervention or treatment in any form."

**C)** Teve grupo controle?

( ) Sim ( ) Não

**O)** O estudo apresenta valores de marcadores inflamatórios (citocinas e outros marcadores) antes e após as intervenções de creatina?

( ) Sim ( ) Não

**S)** O estudo é um ensaio clínico randomizado e controlado?

( ) Sim ( ) Não

**Artigo 221: Creatine Enhances Mitochondrial-Mediated Oligodendrocyte Survival After Demyelinating Injury.**

Chamberlain KA, Chapey KS, Nanescu SE, Huang JK

**P)** Os participantes possuem mais de 18 anos de idade?

( ) Sim (X) Não

"The 'Materials and Methods Mice' section explicitly states that the study was conducted on '**C57BL/6J mice**' and '**Gamt / mice**'. Furthermore, the 'Creatine administration increases oligodendrocyte density after focal spinal cord demyelination' section specifies that 'spinal cord lesions were conducted on **9- to 12-week-old wild-type and Gamt / mice**'. The study also utilized 'Mixed glia cultures were prepared from **postnatal day 3 (P3) to P5 mouse cortices**'. Therefore, the participants were mice and mouse cell cultures, not humans over 18 years of age."

**I)** A intervenção utilizada no estudo é creatina? Vale qualquer formato de administração

( ) Sim ( ) Não

**C)** Teve grupo controle?

( ) Sim ( ) Não

**O)** O estudo apresenta valores de marcadores inflamatórios (citocinas e outros marcadores) antes e após as intervenções de creatina?

( ) Sim ( ) Não

**S)** O estudo é um ensaio clínico randomizado e controlado?

( ) Sim ( ) Não

**Artigo 222: Early prediction of organ failure under the revised Atlanta classification.**

Liu J, Cao F, Dong XM, Li PY, Li HC, Qi BJ, Li F

**P)** Os participantes possuem mais de 18 anos de idade?

(X) Sim ( ) Não

"The 'Exclusion criteria' section states: 'patients who were pregnant or **aged <18 years**'. This indicates that all included participants were 18 years of age or older. Furthermore, the 'Clinical Data' table reports the 'age (median, range)' for the pancreatitis group as '50 (22–70)' years and for the non-AP group as '42 (18–77)' years, confirming that all participants were adults."

**I)** A intervenção utilizada no estudo é creatina? Vale qualquer formato de administração

( ) Sim (X) Não

"The study is an '**observational control study**' that retrospectively analyzed medical records of patients with acute pancreatitis. It aimed to compare the ability of 'conventional laboratory markers and scoring systems to early predict organ failure'. **Creatinine (Cr) is explicitly listed as one of the 'laboratory markers' measured** ('serum creatinine (Cr)') to assess kidney function, not as a substance administered as an intervention or treatment."

**C)** Teve grupo controle?

( ) Sim ( ) Não

**O)** O estudo apresenta valores de marcadores inflamatórios (citocinas e outros marcadores) antes e após as intervenções de creatina?

( ) Sim ( ) Não

**S)** O estudo é um ensaio clínico randomizado e controlado?

( ) Sim ( ) Não

**Artigo 223: Markers of muscle damage for comparing soft tissue injury following proximal femur nail and dynamic hip screw operations for intertrochanteric hip fractures.**

Wagman Y, Segal O, Dudkiewicz I, Steinberg E

**P)** Os participantes possuem mais de 18 anos de idade?

(X) Sim ( ) Não

"The 'Introduction' section highlights that the study addresses 'intertrochanteric hip fractures among **older women**' and in the context of 'today’s **ageing population**'. While an explicit age range is not provided, the consistent emphasis on 'older' and 'ageing' patients strongly implies that all participants were adults aged 18 years or older, as these types of fractures are characteristic of elderly individuals."

**I)** A intervenção utilizada no estudo é creatina? Vale qualquer formato de administração

( ) Sim (X) Não

"The study's aim was to provide objective evidence of local soft tissue injury by **measuring serum creatine phosphokinase (CPK)**, a biochemical marker, to quantify muscle damage and inflammation in patients treated by two operative approaches: proximal femur nail (PFN) or dynamic hip screw (DHS). Creatine phosphokinase (CPK) is a measured enzyme, not an administered substance, and the interventions in this study are the **surgical procedures (PFN or DHS)** themselves."

**C)** Teve grupo controle?

( ) Sim ( ) Não

**O)** O estudo apresenta valores de marcadores inflamatórios (citocinas e outros marcadores) antes e após as intervenções de creatina?

( ) Sim ( ) Não

**S)** O estudo é um ensaio clínico randomizado e controlado?

( ) Sim ( ) Não

**Artigo 224: Should gentamicin trough levels be routinely obtained in term neonates?**

Ibrahim J, Maffei D, El-Chaar G, Islam S, Ponnaiya S, Nayak A, Rosenfeld W, Hanna N

**P)** Os participantes possuem mais de 18 anos de idade?

( ) Sim ( ) Não [*Excluir*]

**I)** A intervenção utilizada no estudo é creatina? Vale qualquer formato de administração

( ) Sim ( ) Não [*Excluir*]

**C)** Teve grupo controle?

( ) Sim ( ) Não [*Excluir*]

**O)** O estudo apresenta valores de marcadores inflamatórios (citocinas e outros marcadores) antes e após as intervenções de creatina?

( ) Sim ( ) Não [*Excluir*]

**S)** O estudo é um ensaio clínico randomizado e controlado?

( ) Sim ( ) Não [*Excluir*]

**Artigo 225: Shiga Toxin Mediated Neurologic Changes in**Murine**Model of Disease.**

Pradhan S, Pellino C, MacMaster K, Coyle D, Weiss AA

**P)** Os participantes possuem mais de 18 anos de idade?

( ) Sim (X) Não

"The title of the article, 'Shiga Toxin Mediated Neurologic Changes in **Murine Model of Disease**', already indicates the use of an animal model. The 'MATERIALS AND METHODS' section explicitly states that the study involved '**Outbred male CD-1 mice, 13–15 g**'. This confirms that the participants were mice and not humans over 18 years of age."

**I)** A intervenção utilizada no estudo é creatina? Vale qualquer formato de administração

( ) Sim ( ) Não

**C)** Teve grupo controle?

( ) Sim ( ) Não

**O)** O estudo apresenta valores de marcadores inflamatórios (citocinas e outros marcadores) antes e após as intervenções de creatina?

( ) Sim ( ) Não

**S)** O estudo é um ensaio clínico randomizado e controlado?

( ) Sim ( ) Não

**Artigo 226: Monitoring interferon β treatment response with magnetic resonance spectroscopy in relapsing remitting multiple sclerosis.**

Yetkin MF, Mirza M, Dönmez H

**P)** Os participantes possuem mais de 18 anos de idade?

(X) Sim ( ) Não

"The 'Participants' section explicitly states the inclusion criteria: 'Individuals who met the following inclusion criteria were enrolled in this study: **an age older than 18 years**'. Additionally, the 'Results' section mentions the 'mean age: **34.08±7.4**' for the RRMS patients, confirming they are adults."

**I)** A intervenção utilizada no estudo é creatina? Vale qualquer formato de administração

( ) Sim (X) Não

"The study's primary intervention is **interferon β (IFN-b)**, as indicated in the title and abstract: 'Monitoring **interferon β** treatment response...' and 'recently diagnosed MS patients never treated with **interferon b**'. Patients were 'randomized to 3 **IFN-b** treatment groups (Avonex, Biogen, Cambridge, MA, USA; Betaferon, Schering AG, Berlin, Germany; Rebif, Serono, Geneva, Switzerland)'. **Creatine (Cr)** is mentioned as a **metabolite measured by magnetic resonance spectroscopy (MRS)**, specifically in ratios like N-acetylaspartate/creatine (NAA/Cr) and choline/creatine (Cho/Cr), not as a substance administered."

**C)** Teve grupo controle?

( ) Sim ( ) Não

**O)** O estudo apresenta valores de marcadores inflamatórios (citocinas e outros marcadores) antes e após as intervenções de creatina?

( ) Sim ( ) Não

**S)** O estudo é um ensaio clínico randomizado e controlado?

( ) Sim ( ) Não

**Artigo 227: Effects of insulin combined with ethyl pyruvate on inflammatory response and oxidative stress in multiple-organ dysfunction syndrome**rats**with severe burns.**

Wang Z, Chen R, Zhu Z, Zhang X, Wang S

**P)** Os participantes possuem mais de 18 anos de idade?

( ) Sim (X) Não

"The study explicitly states in its abstract and methods that it used an **animal model**. The 'Methods' section details: 'We prepared a **MODS rat model** with 30% total body surface area and third-degree burns' and the title itself indicates 'in **multiple-organ dysfunction syndrome rats** with severe burns'. This confirms that the participants were **rats**, not humans, and therefore not over 18 years of age."

**I)** A intervenção utilizada no estudo é creatina? Vale qualquer formato de administração

( ) Sim ( ) Não

**C)** Teve grupo controle?

( ) Sim ( ) Não

**O)** O estudo apresenta valores de marcadores inflamatórios (citocinas e outros marcadores) antes e após as intervenções de creatina?

( ) Sim ( ) Não

**S)** O estudo é um ensaio clínico randomizado e controlado?

( ) Sim ( ) Não

**Artigo 228: Evaluation of inflammation during fixed orthodontic treatment.**

Bilgic F, Akinci Sozer O, Ozcan O, Gurpinar AB, Yilmaz H, Ay Y

**P)** Os participantes possuem mais de 18 anos de idade?

( ) Sim (X) Não

"The 'Materials & methods' section, specifically under '2.1. Participants', states: 'There were 16 women (mean age **14,59 ± 2,16 years**) and 21 men (mean age **14,86 ± 2,13 years**) with mean age of **14,75 ± 1,89 years** in the study group, and 21 women (mean age **13,75 ± 2,12 years**) and 19 men (mean age **13.45 ± 2,31 years**) with mean age of **13.68 ± 2,09 years** in the control group'."

**I)** A intervenção utilizada no estudo é creatina? Vale qualquer formato de administração

( ) Sim ( ) Não

**C)** Teve grupo controle?

( ) Sim ( ) Não

**O)** O estudo apresenta valores de marcadores inflamatórios (citocinas e outros marcadores) antes e após as intervenções de creatina?

( ) Sim ( ) Não

**S)** O estudo é um ensaio clínico randomizado e controlado?

( ) Sim ( ) Não

**Artigo 229: The role of autosuggestion in geriatric patients' quality of life: a study on psycho-neuro-endocrine-immunology pathway.**

Sari NK, Setiati S, Taher A, Wiwie M, Djauzi S, Pandelaki J, Purba JS, Sadikin M

**P)** Os participantes possuem mais de 18 anos de idade?

(X) Sim ( ) Não

"The 'Methods' section explicitly states the study enrolled 'Sixty geriatric patients **aged ≥60 years**' and that inclusion criteria included 'geriatric patients... **aged 60 years or older**'. This confirms that all participants were well over 18 years of age."

**I)** A intervenção utilizada no estudo é creatina? Vale qualquer formato de administração

( ) Sim (X) Não

"The intervention utilized in the study was **autosuggestion**, as clearly stated: 'Sixty geriatric patients... were randomly assigned to either receive **autosuggestion** or not. Autosuggestion was recorded in a tape to be heard daily for 30 days'. Creatine (specifically 'N-acetylaspartate/creatine ratio') was mentioned as a secondary outcome measured by magnetic resonance spectroscopy, indicating it was a **biomarker** being evaluated, not an administered intervention."

**C)** Teve grupo controle?

( ) Sim ( ) Não

**O)** O estudo apresenta valores de marcadores inflamatórios (citocinas e outros marcadores) antes e após as intervenções de creatina?

( ) Sim ( ) Não

**S)** O estudo é um ensaio clínico randomizado e controlado?

( ) Sim ( ) Não

**Artigo 230: Paeoniflorin ameliorates acute necrotizing pancreatitis and pancreatitis‑induced acute renal injury.**

Wang P, Wang W, Shi Q, Zhao L, Mei F, Li C, Zuo T, He X

**P)** Os participantes possuem mais de 18 anos de idade?

( ) Sim (X) Não

"The 'Experimental design' section explicitly states the study was conducted on **animals**: 'In the first part of the present study, **48 SD rats** were randomly divided into six groups (8 rats/group)'. The title also indicates 'in **rats**'. Therefore, the participants were not human and not over 18 years of age."

**I)** A intervenção utilizada no estudo é creatina? Vale qualquer formato de administração

( ) Sim ( ) Não

**C)** Teve grupo controle?

( ) Sim ( ) Não

**O)** O estudo apresenta valores de marcadores inflamatórios (citocinas e outros marcadores) antes e após as intervenções de creatina?

( ) Sim ( ) Não

**S)** O estudo é um ensaio clínico randomizado e controlado?

( ) Sim ( ) Não

**Artigo 231: Low-dose testosterone protects against renal ischemia-reperfusion injury by increasing renal IL-10-to-TNF-α ratio and attenuating T-cell infiltration.**

Patil CN, Wallace K, LaMarca BD, Moulana M, Lopez-Ruiz A, Soljancic A, Juncos LA, Grande JP, Reckelhoff JF

**P)** Os participantes possuem mais de 18 anos de idade?

( ) Sim (X) Não

"The 'MATERIALS AND METHODS' section, specifically under 'Animals', states: '**Male Sprague-Dawley rats, aged 8–10 wk (290–300 g), were obtained from the vendor... Rats were used at 9–14 wk of age**'. This clearly indicates that the study subjects were **rats**, not humans, and therefore were not over 18 years of age."

**I)** A intervenção utilizada no estudo é creatina? Vale qualquer formato de administração

( ) Sim ( ) Não

**C)** Teve grupo controle?

( ) Sim ( ) Não

**O)** O estudo apresenta valores de marcadores inflamatórios (citocinas e outros marcadores) antes e após as intervenções de creatina?

( ) Sim ( ) Não

**S)** O estudo é um ensaio clínico randomizado e controlado?

( ) Sim ( ) Não

**Artigo 232: Evaluation of novel biomarkers of nephrotoxicity in Cynomolgus monkeys treated with gentamicin.**

Gautier JC, Zhou X, Yang Y, Gury T, Qu Z, Palazzi X, Léonard JF, Slaoui M, Veeranagouda Y, Guizon I, Boitier E, Filali-Ansary A, van den Berg BHJ, Poetz O, Joos T, Zhang T, Wang J, Detilleux P, Li B

**P)** Os participantes possuem mais de 18 anos de idade?

( ) Sim (X) Não

"The 'Materials and methods' section, specifically under 'Animals and husbandry', states: '**Male Cynomolgus monkeys, 2–3 years of age and weighing 2–4 kg, were obtained from Shin Nippon Biomedical Laboratories, Ltd. (China)...**'. This clearly indicates that the subjects of the study were **monkeys**, not humans, and therefore were not over 18 years of age."

**I)** A intervenção utilizada no estudo é creatina? Vale qualquer formato de administração

( ) Sim ( ) Não

**C)** Teve grupo controle?

( ) Sim ( ) Não

**O)** O estudo apresenta valores de marcadores inflamatórios (citocinas e outros marcadores) antes e após as intervenções de creatina?

( ) Sim ( ) Não

**S)** O estudo é um ensaio clínico randomizado e controlado?

( ) Sim ( ) Não

**Artigo 233: Soluble TNF-Like Weak Inducer of Apoptosis as a New Marker in Preeclampsia: A Pilot Clinical Study.**

Yildirim ZK, Sumnu A, Bademler N, Kilic E, Sumnu G, Karadag S, Gursu M, Ozel A, Batmaz G, Ates S, Dane B, Ozturk S

**P)** Os participantes possuem mais de 18 anos de idade?

(X) Sim ( ) Não

"The 'Patients' section states: 'The study was undertaken with 33 patients with preeclampsia and 33 normal pregnant women.'. **Preeclampsia is a condition that occurs during pregnancy**, and participants providing 'Written informed consent was obtained from all participants' generally indicates they are adults and thus, **over 18 years of age**. While no explicit age range is given, involvement of pregnant women strongly implies an adult population."

**I)** A intervenção utilizada no estudo é creatina? Vale qualquer formato de administração

( ) Sim (X) Não

"The 'Introduction' and 'Materials and Methods' sections indicate that the study aimed to 'compare sTWEAK levels in women with preeclampsia to corresponding levels in a healthy pregnant control group'. The study measured various biomarkers such as 'serum creatinine, uric acid, LDH levels, and uPCR'. **There is no mention of creatine or its administration in any format as an intervention**."

**C)** Teve grupo controle?

( ) Sim ( ) Não

**O)** O estudo apresenta valores de marcadores inflamatórios (citocinas e outros marcadores) antes e após as intervenções de creatina?

( ) Sim ( ) Não

**S)** O estudo é um ensaio clínico randomizado e controlado?

( ) Sim ( ) Não

**Artigo 234: Glial and axonal changes in systemic lupus erythematosus measured with diffusion of intracellular metabolites.**

Ercan E, Magro-Checa C, Valabregue R, Branzoli F, Wood ET, Steup-Beekman GM, Webb AG, Huizinga TW, van Buchem MA, Ronen I

**P)** Os participantes possuem mais de 18 anos de idade?

(X) Sim ( ) Não

"The 'Materials and methods' section, under 'Human subjects', states: 'Twenty-nine patients with SLE (one male, 28 females, **age: 43 ± 10 years**) and 19 age- and sex-matched healthy volunteers (one male, 18 females, **age: 41 ± 11 years**) were included in the study.' This clearly indicates that all participants were adults."

**I)** A intervenção utilizada no estudo é creatina? Vale qualquer formato de administração

( ) Sim (X) Não

"The study's objective was to measure 'Glial and axonal changes in systemic lupus erythematosus measured with diffusion of intracellular metabolites' and 'assess the relationship between DW-MRS indices and SLE activity'. The methodology primarily involved Diffusion-Weighted Magnetic Resonance Spectroscopy (DW-MRS) to assess metabolites like N-acetylaspartate (NAA). **There is no mention of creatine being used as an intervention or administered in any format.**"

**C)** Teve grupo controle?

( ) Sim ( ) Não

**O)** O estudo apresenta valores de marcadores inflamatórios (citocinas e outros marcadores) antes e após as intervenções de creatina?

( ) Sim ( ) Não

**S)** O estudo é um ensaio clínico randomizado e controlado?

( ) Sim ( ) Não

**Artigo 235: Host Factors and Biomarkers Associated with Poor Outcomes in Adults with Invasive Pneumococcal Disease.**

Hanada S, Iwata S, Kishi K, Morozumi M, Chiba N, Wajima T, Takata M, Ubukata K

**P)** Os participantes possuem mais de 18 anos de idade?

(X) Sim ( ) Não

"The 'Study design' section states: 'Among 1317 patients with IPD who were admitted to 341 hospitals throughout Japan between April 2010 and March 2013, **adults at least 18 years old numbered 715**'. Furthermore, the 'Results' section indicates the mean patient age was '68.7 years (median, 71 years; range, 21–97 years)', confirming all participants were adults."

**I)** A intervenção utilizada no estudo é creatina? Vale qualquer formato de administração

( ) Sim (X) Não

"The study is a 'prospective observational multicenter cohort study' focused on 'Host Factors and Biomarkers Associated with Poor Outcomes in Adults with Invasive Pneumococcal Disease'. The researchers investigated the role of 'host factors, disease severity, biomarkers based on clinical laboratory data, treatment regimens, and bacterial factors'. **Creatine is not mentioned as an intervention or administered in any format**; rather, serum creatinine is listed as a measured 'biomarker' related to kidney function."

**C)** Teve grupo controle?

( ) Sim ( ) Não

**O)** O estudo apresenta valores de marcadores inflamatórios (citocinas e outros marcadores) antes e após as intervenções de creatina?

( ) Sim ( ) Não

**S)** O estudo é um ensaio clínico randomizado e controlado?

( ) Sim ( ) Não

**Artigo 236: Exercise oxidative skeletal muscle metabolism in adolescents with cystic fibrosis.**

Werkman M, Jeneson J, Helders P, Arets B, van der Ent K, Velthuis B, Nievelstein R, Takken T, Hulzebos E

**P)** Os participantes possuem mais de 18 anos de idade?

( ) Sim (X) Não

"The 'Methods' section states: 'Ten adolescents with CF (**12–18 years of age**, FEV1>80%pred, resting oxygen saturation > 94%) and ten healthy age-matched controls (HC) were tested with supine cycle'. Additionally, the 'Participants' section mentions: 'All participants, CF and HC, and (**when < 18 years of age**) their parents gave written informed consent'. The baseline characteristics table also shows the mean age for the CF group as '13.8±1.3 years' and for the HC group as '13.7±1.1 years'. This clearly indicates that a significant portion, if not all, of the participants were under 18 years old."

**I)** A intervenção utilizada no estudo é creatina? Vale qualquer formato de administração

( ) Sim ( ) Não

**C)** Teve grupo controle?

( ) Sim ( ) Não

**O)** O estudo apresenta valores de marcadores inflamatórios (citocinas e outros marcadores) antes e após as intervenções de creatina?

( ) Sim ( ) Não

**S)** O estudo é um ensaio clínico randomizado e controlado?

( ) Sim ( ) Não

**Artigo 237: Protective effect of thymol on high fat diet induced diabetic nephropathy in C57BL/6J**mice**.**

Saravanan S, Pari L

**P)** Os participantes possuem mais de 18 anos de idade?

( ) Sim (X) Não

"The 'Materials and methods' section, under 'Experimental animals', states: 'Healthy adult male C57BL/6J mice **3 weeks of age** was obtained from NIN Hyderabad and housed in polypropylene cages.' This indicates that the participants were mice and were only 3 weeks old, which is not over 18 years of age."

**I)** A intervenção utilizada no estudo é creatina? Vale qualquer formato de administração

( ) Sim ( ) Não

**C)** Teve grupo controle?

( ) Sim ( ) Não

**O)** O estudo apresenta valores de marcadores inflamatórios (citocinas e outros marcadores) antes e após as intervenções de creatina?

( ) Sim ( ) Não

**S)** O estudo é um ensaio clínico randomizado e controlado?

( ) Sim ( ) Não

**Artigo 238: Hydrogen-Rich Saline Attenuates Lipopolysaccharide-Induced Heart Dysfunction by Restoring Fatty Acid Oxidation in**Rats**by Mitigating C-Jun N-Terminal Kinase Activation.**

Tao B, Liu L, Wang N, Tong D, Wang W, Zhang J

**P)** Os participantes possuem mais de 18 anos de idade?

( ) Sim (X) Não

"The title of the article clearly states '...in **Rats**...'. Additionally, the listed authors' affiliations in and references to animal models in other parts of the source confirm that **this study was conducted on animals, specifically rats and mice, and not human participants**, therefore they are not over 18 years of age."

**I)** A intervenção utilizada no estudo é creatina? Vale qualquer formato de administração

( ) Sim ( ) Não

**C)** Teve grupo controle?

( ) Sim ( ) Não

**O)** O estudo apresenta valores de marcadores inflamatórios (citocinas e outros marcadores) antes e após as intervenções de creatina?

( ) Sim ( ) Não

**S)** O estudo é um ensaio clínico randomizado e controlado?

( ) Sim ( ) Não

**Artigo 239: Reduction in Renal Ischemia-Reperfusion Injury in**Mice**by a Phosphoinositide 3-Kinase p110gamma-Specific Inhibitor.**

Kim N, Woo DC, Joo SJ, Song Y, Lee JJ, Woo CW, Kim ST, Hong S, Cho YM, Han DJ

**P)** Os participantes possuem mais de 18 anos de idade?

( ) Sim (X) Não

"The 'MATERIALS AND METHODS' section, under 'Animal Experiments', explicitly states: '**Female C57BL/6 mice** were purchased... In brief, bilateral renal pedicles of **7–9-week-old mice** were clamped'. This indicates that the study was conducted on mice that were only a few weeks old, not human participants over 18 years of age."

**I)** A intervenção utilizada no estudo é creatina? Vale qualquer formato de administração

( ) Sim ( ) Não

**C)** Teve grupo controle?

( ) Sim ( ) Não

**O)** O estudo apresenta valores de marcadores inflamatórios (citocinas e outros marcadores) antes e após as intervenções de creatina?

( ) Sim ( ) Não

**S)** O estudo é um ensaio clínico randomizado e controlado?

( ) Sim ( ) Não

**Artigo 240: Proton magnetic resonance spectroscopy and outcome in term neonates with chorioamnionitis.**

Johnson CB, Jenkins DD, Bentzley JP, Lambert D, Hope K, Rollins LG, Morgan PS, Brown T, Ramakrishnan V, Mulvihill DM, Katikaneni LD

**P)** Os participantes possuem mais de 18 anos de idade?

( ) Sim (X) Não

"The 'Study population' section within 'METHODS' states: 'This study... was conducted... on **term infants born ≥ 37 weeks gestation** to mothers with chorioamnionitis'. It further clarifies the timing of scans: 'Infants with confirmed funisitis were scheduled for MRS scans as an outpatient **between 1 and 4 weeks after discharge**'. The 'RESULTS' section also mentions that scans were performed 'between 0.2 and 4.0 weeks after birth (mean 1.5 ± 0.9 weeks, postconceptional gestational age 38 to 44 weeks)'. This clearly indicates that the participants were newborns/infants, not individuals over 18 years of age."

**I)** A intervenção utilizada no estudo é creatina? Vale qualquer formato de administração

( ) Sim ( ) Não

**C)** Teve grupo controle?

( ) Sim ( ) Não

**O)** O estudo apresenta valores de marcadores inflamatórios (citocinas e outros marcadores) antes e após as intervenções de creatina?

( ) Sim ( ) Não

**S)** O estudo é um ensaio clínico randomizado e controlado?

( ) Sim ( ) Não

**Artigo 241: A Critical Proton MR Spectroscopy Marker of Alzheimer's Disease Early Neurodegenerative Change: Low Hippocampal NAA/Cr Ratio Impacts APOE ɛ4 Mexico City Children and Their Parents.**

Calderón-Garcidueñas L, Mora-Tiscareño A, Melo-Sánchez G, Rodríguez-Díaz J, Torres-Jardón R, Styner M, Mukherjee PS, Lin W, Jewells V

**P)** Os participantes possuem mais de 18 anos de idade?

(X) Sim ( ) Não

"The 'Methods' section, under 'Study Population', states: 'Control participants were also aged between **18–55 years**, were matched within two years of participants with ME/CFS, and reported average daily fatigue ratings of ≤ 2 on an 11-point scale.' The table of 'Demographic and clinical data' confirms this, showing the mean age for the ME/CFS group as '40.27 ± 8.84 years' and for the control group as '40.80 ± 9.22 years', with the minimum age in the range covered by '18–55 years'."

**I)** A intervenção utilizada no estudo é creatina? Vale qualquer formato de administração

( ) Sim (X) Não

"The study quantifies metabolites in brain regions, expressing them 'as ratios over **creatine (CR)**'. This indicates that creatine is used as a reference metabolite for quantification in magnetic resonance spectroscopy, not as an administered intervention or treatment in any form. The study focuses on 'widespread metabolite abnormalities' in Myalgic encephalomyelitis/chronic fatigue syndrome assessment."

**C)** Teve grupo controle?

( ) Sim ( ) Não

**O)** O estudo apresenta valores de marcadores inflamatórios (citocinas e outros marcadores) antes e após as intervenções de creatina?

( ) Sim ( ) Não

**S)** O estudo é um ensaio clínico randomizado e controlado?

( ) Sim ( ) Não

**Artigo 242: Dietary supplementation with n-3 fatty acids from weaning limits brain biochemistry and behavioural changes elicited by prenatal exposure to maternal inflammation in the**mouse**model.**

Li Q, Leung YO, Zhou I, Ho LC, Kong W, Basil P, Wei R, Lam S, Zhang X, Law AC, Chua SE, Sham PC, Wu EX, McAlonan GM

**P)** Os participantes possuem mais de 18 anos de idade?

( ) Sim (X) Não

"The 'MATERIALS AND METHODS' section, under the first paragraph, states: 'Female and male **C57BL6/N mice** were bred and mated by The University of Hong Kong, Laboratory Animal Unit.' It further specifies the age of the subjects for scanning: '**Twelve-week-old mice** were scanned'. This clearly indicates that the study was conducted on mice, which are not over 18 years of age."

**I)** A intervenção utilizada no estudo é creatina? Vale qualquer formato de administração

( ) Sim ( ) Não

**C)** Teve grupo controle?

( ) Sim ( ) Não

**O)** O estudo apresenta valores de marcadores inflamatórios (citocinas e outros marcadores) antes e após as intervenções de creatina?

( ) Sim ( ) Não

**S)** O estudo é um ensaio clínico randomizado e controlado?

( ) Sim ( ) Não

**Artigo 243: Temporal Changes in Caspase-1 and Caspase-8 Activities Following Brain Hypoxia With and Without Src kinase Inhibition in a Piglet**Animal**Model.**

Angelis D, Fontánez Nieves TD, Delivoria-Papadopoulos M

**P)** Os participantes possuem mais de 18 anos de idade?

( ) Sim (X) Não

"The title of the article clearly states '...in a **Piglet Animal Model**'. Furthermore, the 'Experimental Protocol' section within the 'Materials and Methods' explicitly mentions that 'Animal experiments: This study was carried out in accordance with the recommendations in the Guide for the Care... **Piglets** were assigned to the hypoxic group'. This confirms that the study was conducted on animals (piglets), not human participants, and therefore they are not over 18 years of age."

**I)** A intervenção utilizada no estudo é creatina? Vale qualquer formato de administração

( ) Sim ( ) Não

**C)** Teve grupo controle?

( ) Sim ( ) Não

**O)** O estudo apresenta valores de marcadores inflamatórios (citocinas e outros marcadores) antes e após as intervenções de creatina?

( ) Sim ( ) Não

**S)** O estudo é um ensaio clínico randomizado e controlado?

( ) Sim ( ) Não

**Artigo 244: The Effects of Creatine Supplementation and Physical Exercise on Traumatic Brain Injury.**

Freire Royes LF, Cassol G

**P)** Os participantes possuem mais de 18 anos de idade?

( ) Sim ( ) Não [*Excluir*]

**I)** A intervenção utilizada no estudo é creatina? Vale qualquer formato de administração

( ) Sim ( ) Não [*Excluir*]

**C)** Teve grupo controle?

( ) Sim ( ) Não [*Excluir*]

**O)** O estudo apresenta valores de marcadores inflamatórios (citocinas e outros marcadores) antes e após as intervenções de creatina?

( ) Sim ( ) Não [*Excluir*]

**S)** O estudo é um ensaio clínico randomizado e controlado?

( ) Sim ( ) Não [*Excluir*]

**Artigo 245: Metabolomic profiles reveal key metabolic changes in heat stress-treated**mouse**Sertoli**cells**.**

Xu B, Chen M, Ji X, Yao M, Mao Z, Zhou K, Xia Y, Han X, Tang W

**P)** Os participantes possuem mais de 18 anos de idade?

( ) Sim (X) Não

"The title of the article clearly states '...in heat stress-treated **mouse Sertoli cells**'. The 'Materials and methods' section, under '2.2. Cell culture and HS treatment', further confirms this by stating: '**TM-4 (ATCC # CRL -1715) cell line** was purchased from ATCC... and cultured in complete medium'. This indicates that the study was conducted on a mouse cell line, not human participants, and therefore they are not over 18 years of age."

**I)** A intervenção utilizada no estudo é creatina? Vale qualquer formato de administração

( ) Sim ( ) Não

**C)** Teve grupo controle?

( ) Sim ( ) Não

**O)** O estudo apresenta valores de marcadores inflamatórios (citocinas e outros marcadores) antes e após as intervenções de creatina?

( ) Sim ( ) Não

**S)** O estudo é um ensaio clínico randomizado e controlado?

( ) Sim ( ) Não

**Artigo 246: Rhein prevents endotoxin-induced acute kidney injury by inhibiting NF-κB activities.**

Yu C, Qi D, Sun JF, Li P, Fan HY

**P)** Os participantes possuem mais de 18 anos de idade?

( ) Sim (X) Não

"The 'Experimental design' section within 'Materials and Methods' states: 'In vivo study. Model of LPS-induced acute kidney injury. The **mice** were intragastrically (i.g.) given...'. Furthermore, the 'In vitro study' section mentions the use of 'Human renal proximal tubular epithelial cells (**HK-2 cells**)'. This indicates that the study was conducted on animals (mice) and human cell lines, not human participants over 18 years of age."

**I)** A intervenção utilizada no estudo é creatina? Vale qualquer formato de administração

( ) Sim ( ) Não

**C)** Teve grupo controle?

( ) Sim ( ) Não

**O)** O estudo apresenta valores de marcadores inflamatórios (citocinas e outros marcadores) antes e após as intervenções de creatina?

( ) Sim ( ) Não

**S)** O estudo é um ensaio clínico randomizado e controlado?

( ) Sim ( ) Não

**Artigo 247: Increase in oxidative stress biomarkers in dogs with ascending-descending myelomalacia following spinal cord injury.**

Marquis A, Packer RA, Borgens RB, Duerstock BS

**P)** Os participantes possuem mais de 18 anos de idade?

( ) Sim (X) Não

"The title of the article clearly states '...in **dogs** with ascending-descending myelomalacia following spinal cord injury'. The '2.1. Animal Use and collection of samples' section confirms this, stating: '**Dogs** admitted to the Purdue University Veterinary Teaching Hospital (PUVTH)...'. Furthermore, 'Table 1: A summary of the characteristics of the animals used in this study' lists the subjects as various breeds of dogs with ages like '2.5 m' (months) and '1.5 f' (years), indicating they are **animals and not humans over 18 years of age**".

**I)** A intervenção utilizada no estudo é creatina? Vale qualquer formato de administração

( ) Sim ( ) Não

**C)** Teve grupo controle?

( ) Sim ( ) Não

**O)** O estudo apresenta valores de marcadores inflamatórios (citocinas e outros marcadores) antes e após as intervenções de creatina?

( ) Sim ( ) Não

**S)** O estudo é um ensaio clínico randomizado e controlado?

( ) Sim ( ) Não

**Artigo 248: Alterations in frontal white matter neurochemistry and microstructure in schizophrenia: implications for neuroinflammation.**

Chiappelli J, Hong LE, Wijtenburg SA, Du X, Gaston F, Kochunov P, Rowland LM

**P)** Os participantes possuem mais de 18 anos de idade?

(X) Sim ( ) Não

"The 'Participants' section under 'MATERIALS AND METHODS' states: 'This study included DTI, 1H-MRS and clinical and neuropsychological testing in 38 persons with schizophrenia (**age range 20–58 years**) and 36 age- and sex-matched community controls (**age range 20–61 years**).' This clearly indicates that all participants were above 18 years of age."

**I)** A intervenção utilizada no estudo é creatina? Vale qualquer formato de administração

( ) Sim (X) Não

"The study investigates 'in vivo neurochemical markers reflective of neuronal health and glial activation'. The 'White matter MRS' section details the metabolites measured: 'NAA plus NAAG (NAA), Cr plus phosphocreatine (Cr), glycerophosphocholine plus phosophocholine (Cho), myo-inositol (mI), and glutamate (Glu)'. Creatine (Cr) is explicitly mentioned as a **metabolite that was measured**, not an intervention or supplement administered to participants."

**C)** Teve grupo controle?

( ) Sim ( ) Não

**O)** O estudo apresenta valores de marcadores inflamatórios (citocinas e outros marcadores) antes e após as intervenções de creatina?

( ) Sim ( ) Não

**S)** O estudo é um ensaio clínico randomizado e controlado?

( ) Sim ( ) Não

**Artigo 249: Cuprizone-induced demyelination and demyelination-associated inflammation result in different proton magnetic resonance metabolite spectra.**

Praet J, Orije J, Kara F, Guglielmetti C, Santermans E, Daans J, Hens N, Verhoye M, Berneman Z, Ponsaerts P, Van der Linden A

**P)** Os participantes possuem mais de 18 anos de idade?

( ) Sim (X) Não

"The 'Animal experiments' section under 'MATERIALS AND METHODS' clearly states: '**Female wild type C57BL/6 Jmice** (denoted CX3CR1 +/+mice, n= 34), **8 weeks of age**, were obtained via Charles River Laboratories (L’Arbresle Cedex, France)'. This indicates that the study was conducted on **mice**, not human participants, and therefore they are not over 18 years of age."

**I)** A intervenção utilizada no estudo é creatina? Vale qualquer formato de administração

( ) Sim ( ) Não

**C)** Teve grupo controle?

( ) Sim ( ) Não

**O)** O estudo apresenta valores de marcadores inflamatórios (citocinas e outros marcadores) antes e após as intervenções de creatina?

( ) Sim ( ) Não

**S)** O estudo é um ensaio clínico randomizado e controlado?

( ) Sim ( ) Não

**Artigo 250: High phosphorus level leads to aortic calcification via β-catenin in chronic kidney disease.**

Yao L, Sun YT, Sun W, Xu TH, Ren C, Fan X, Sun L, Liu LL, Feng JM, Ma JF, Wang LN

**P)** Os participantes possuem mais de 18 anos de idade?

( ) Sim (X) Não

"The 'Abstract' mentions that 'The **5/6 nephrectomized rat** was used as an *in vivo* model...'. Furthermore, the 'Materials and Methods' section, under 'Animal model of CKD and shRNA injection', states: '**SD rats** weighing 250 g were maintained at constant room temperature and humidity... CKD was induced by 5/6 nephrectomy (Nx)...'. This clearly indicates that the study was conducted on **rats**, not human participants, and therefore they are not over 18 years of age."

**I)** A intervenção utilizada no estudo é creatina? Vale qualquer formato de administração

( ) Sim ( ) Não

**C)** Teve grupo controle?

( ) Sim ( ) Não

**O)** O estudo apresenta valores de marcadores inflamatórios (citocinas e outros marcadores) antes e após as intervenções de creatina?

( ) Sim ( ) Não

**S)** O estudo é um ensaio clínico randomizado e controlado?

( ) Sim ( ) Não

**Artigo 251: Decreases in Short Term Memory, IQ, and Altered Brain Metabolic Ratios in Urban Apolipoprotein ε4 Children Exposed to Air Pollution.**

Calderón-Garcidueñas L, Mora-Tiscareño A, Franco-Lira M, Zhu H, Lu Z, Solorio E, Torres-Jardón R, D'Angiulli A

**P)** Os participantes possuem mais de 18 anos de idade?

( ) Sim (X) Não

"The title of the article clearly states '...in Urban Apolipoprotein ε4 **Children** Exposed to Air Pollution'. This indicates that the study participants were **children**, and therefore not over 18 years of age."

**I)** A intervenção utilizada no estudo é creatina? Vale qualquer formato de administração

( ) Sim ( ) Não

**C)** Teve grupo controle?

( ) Sim ( ) Não

**O)** O estudo apresenta valores de marcadores inflamatórios (citocinas e outros marcadores) antes e após as intervenções de creatina?

( ) Sim ( ) Não

**S)** O estudo é um ensaio clínico randomizado e controlado?

( ) Sim ( ) Não

**Artigo 252: The Role of Uncoupling Protein 2 During Myocardial Dysfunction in a**Canine**Model of Endotoxin Shock.**

Wang X, Liu D, Chai W, Long Y, Su L, Yang R

**P)** Os participantes possuem mais de 18 anos de idade?

( ) Sim (X) Não

"The 'MATERIALS AND METHODS' section, specifically under 'Animals', states: 'The **mongrel, adult canines** (N = 25) used in this study were provided by the Experimental Animal Department of Peking Union Medical College (Beijing, China). The ages of the canines ranged from **300 to 360 days**...'. This clearly indicates that the participants were **dogs**, not humans, and therefore are not over 18 years of age."

**I)** A intervenção utilizada no estudo é creatina? Vale qualquer formato de administração

( ) Sim ( ) Não

**C)** Teve grupo controle?

( ) Sim ( ) Não

**O)** O estudo apresenta valores de marcadores inflamatórios (citocinas e outros marcadores) antes e após as intervenções de creatina?

( ) Sim ( ) Não

**S)** O estudo é um ensaio clínico randomizado e controlado?

( ) Sim ( ) Não

**Artigo 253: The value of measuring urinary β2-microglobulin and serum creatinine for detecting tubulointerstitial nephritis and uveitis syndrome in young patients with uveitis.**

Hettinga YM, Scheerlinck LM, Lilien MR, Rothova A, de Boer JH

**P)** Os participantes possuem mais de 18 anos de idade?

( ) Sim (X) Não

"The 'DESIGN, SETTING, AND PARTICIPANTS' section states: 'Forty-five consecutive new patients with uveitis **aged 22 years or younger** were enrolled'. Furthermore, the 'Methods' section under 'Patient Population' clarifies: 'We recruited 45 consecutive new patients who presented at our outpatient clinic with uveitis with an **age at onset of uveitis of 23 years or younger (range, 4-22 years)**'. This explicitly indicates that the study participants were **children and young adults**, with some or all being 18 years of age or younger."

**I)** A intervenção utilizada no estudo é creatina? Vale qualquer formato de administração

( ) Sim ( ) Não

**C)** Teve grupo controle?

( ) Sim ( ) Não

**O)** O estudo apresenta valores de marcadores inflamatórios (citocinas e outros marcadores) antes e após as intervenções de creatina?

( ) Sim ( ) Não

**S)** O estudo é um ensaio clínico randomizado e controlado?

( ) Sim ( ) Não

**Artigo 254: Multivoxel proton magnetic resonance spectroscopy of inflammatory and neoplastic lesions of the**canine**brain at 3.0 T.**

Stadler KL, Ober CP, Feeney DA, Jessen CR

**P)** Os participantes possuem mais de 18 anos de idade?

( ) Sim (X) Não

"The 'Objective' and 'Animals' sections of the abstract clearly state that the study was conducted on **dogs**. Specifically, 'Animals—**33 dogs** with intracranial disease (19 neoplastic [10 meningioma, 7 glioma, and 2 other] and 14 inflammatory)'. This indicates that the participants were **animals**, not human subjects, and therefore are not over 18 years of age."

**I)** A intervenção utilizada no estudo é creatina? Vale qualquer formato de administração

( ) Sim ( ) Não

**C)** Teve grupo controle?

( ) Sim ( ) Não

**O)** O estudo apresenta valores de marcadores inflamatórios (citocinas e outros marcadores) antes e após as intervenções de creatina?

( ) Sim ( ) Não

**S)** O estudo é um ensaio clínico randomizado e controlado?

( ) Sim ( ) Não

**Artigo 255: The effects of canrenone on inflammatory markers in patients with metabolic syndrome.**

Derosa G, Romano D, Bianchi L, D'Angelo A, Maffioli P

**P)** Os participantes possuem mais de 18 anos de idade?

( ) Sim (X) Não

"The 'Patients and methods' section, under 'Patients', explicitly states: 'One hundred and fifty-six Caucasian patients were enrolled. Patients were  **18 years old**...'. This confirms that the participants were 18 years of age or older."

**I)** A intervenção utilizada no estudo é creatina? Vale qualquer formato de administração

( ) Sim (X) Não

"The 'Aim' and 'Treatments' sections of the article clearly state that the intervention used was **canrenone**, with patients being 'treated with placebo or canrenone'. There is no mention of creatine or any of its forms being administered as an intervention in the study."

**C)** Teve grupo controle?

( ) Sim ( ) Não

**O)** O estudo apresenta valores de marcadores inflamatórios (citocinas e outros marcadores) antes e após as intervenções de creatina?

( ) Sim ( ) Não

**S)** O estudo é um ensaio clínico randomizado e controlado?

( ) Sim ( ) Não

**Artigo 256: Urinary sodium excretion has positive correlation with activation of urinary renin angiotensin system and reactive oxygen species in hypertensive chronic kidney disease.**

Ahn SY, Kim S, Kim DK, Park JH, Shin SJ, Lee SH, Choi BS, Lim CS, Kim S, Chin HJ

**P)** Os participantes possuem mais de 18 anos de idade?

(X) Sim ( ) Não

"The 'Study population' section states that 'All patients fulfilled the following inclusion criteria: **age of 19-75 yr**...'. Additionally, the 'RESULTS' section mentions that 'The **mean age was 50.3 ± 13.0 yr at enrollment**...'. This confirms that all participants were 19 years of age or older."

**I)** A intervenção utilizada no estudo é creatina? Vale qualquer formato de administração

( ) Sim (X) Não

"The 'Study protocol' section details the interventions used: 'all the enrolled patients were prescribed **olmesartan medoxomil**... with a 40 mg once-a-day fixed dose' and 'participants were randomly assigned to receive a **low salt diet (LSD) intervention**'. The article title and abstract refer to 'creatinine' as a **marker** (e.g., '24-hr urine sodium-to-creatinine ratio'), not as an administered intervention. Therefore, creatine in any form was not used as an intervention in this study."

**C)** Teve grupo controle?

( ) Sim ( ) Não

**O)** O estudo apresenta valores de marcadores inflamatórios (citocinas e outros marcadores) antes e após as intervenções de creatina?

( ) Sim ( ) Não

**S)** O estudo é um ensaio clínico randomizado e controlado?

( ) Sim ( ) Não

**Artigo 257: Soy β-conglycinin retards progression of diabetic nephropathy via modulating the insulin sensitivity and angiotensin-converting enzyme activity in**rats**fed with high salt diet.**

Yeh WJ, Yang HY, Chen JR

**P)** Os participantes possuem mais de 18 anos de idade?

( ) Sim (X) Não

"The 'Methods and materials' section, under 'Animals and diets', explicitly states: 'Male Wistar rats **8 weeks old**, weighing 200–250 g, were purchased...'. The abstract also mentions: 'We used **40 Wistar rats** with eight rats in each group'. This indicates that the participants were **animals (rats)** and not human subjects aged over 18 years."

**I)** A intervenção utilizada no estudo é creatina? Vale qualquer formato de administração

( ) Sim ( ) Não

**C)** Teve grupo controle?

( ) Sim ( ) Não

**O)** O estudo apresenta valores de marcadores inflamatórios (citocinas e outros marcadores) antes e após as intervenções de creatina?

( ) Sim ( ) Não

**S)** O estudo é um ensaio clínico randomizado e controlado?

( ) Sim ( ) Não

**Artigo 258: 1H NMR global metabolic phenotyping of acute pancreatitis in the emergency unit.**

Villaseñor A, Kinross JM, Li JV, Penney N, Barton RH, Nicholson JK, Darzi A, Barbas C, Holmes E

**P)** Os participantes possuem mais de 18 anos de idade?

(X) Sim ( ) Não

"The 'Study Design' section explicitly states the exclusion criteria: 'Patients were excluded if they... were under the age of 18'. Furthermore, the 'Clinical Data' section under 'RESULTS' confirms the age range of participants: 'Patients were aged **18 to 77 years old**, with a median age of 42.5 years (SD = 16.8)'."

**I)** A intervenção utilizada no estudo é creatina? Vale qualquer formato de administração

( ) Sim (X) Não

"The study is described as an '**observational control study**' focused on determining the 'potential of a metabonomic approach in the diagnosis and prognostic staging of AP' by characterizing 'metabolic phenotype' using 1H NMR spectroscopy. While **creatine** is mentioned, it is listed as a 'discriminatory biomarker' found at 'low levels' in urine samples, indicating it was a **measured metabolite** and not an administered intervention."

**C)** Teve grupo controle?

( ) Sim ( ) Não

**O)** O estudo apresenta valores de marcadores inflamatórios (citocinas e outros marcadores) antes e após as intervenções de creatina?

( ) Sim ( ) Não

**S)** O estudo é um ensaio clínico randomizado e controlado?

( ) Sim ( ) Não

**Artigo 259: Effects of ghrelin on sepsis-induced acute kidney injury: one step forward.**

Khowailed A, Younan SM, Ashour H, Kamel AE, Sharawy N

**P)** Os participantes possuem mais de 18 anos de idade?

( ) Sim (X) Não

"The 'Materials and methods' section, under 'Animals', explicitly states: 'After obtaining approval from the institutional Animal Care Committee, **40 male albino rats (body weight 120–150 g)** were purchased...'. This indicates that the participants were **animals (rats)** and not human subjects aged over 18 years."

**I)** A intervenção utilizada no estudo é creatina? Vale qualquer formato de administração

( ) Sim ( ) Não

**C)** Teve grupo controle?

( ) Sim ( ) Não

**O)** O estudo apresenta valores de marcadores inflamatórios (citocinas e outros marcadores) antes e após as intervenções de creatina?

( ) Sim ( ) Não

**S)** O estudo é um ensaio clínico randomizado e controlado?

( ) Sim ( ) Não

**Artigo 260: Associations between in vivo neuroimaging and postmortem brain cytokine markers in a**rodent**model of Wernicke's encephalopathy.**

Zahr NM, Alt C, Mayer D, Rohlfing T, Manning-Bog A, Luong R, Sullivan EV, Pfefferbaum A

**P)** Os participantes possuem mais de 18 anos de idade?

( ) Sim (X) Não

"The title of the article explicitly states 'in a **rodent model** of Wernicke's encephalopathy'. Additionally, the 'METHODS' section, under 'Animals', mentions that the study included '**control animals, thiamine-deficient animals** challenged with saline... and thiamine-deficient animals challenged with glucose'. This confirms that the participants were **animals (rodents)**, not human subjects aged over 18 years."

**I)** A intervenção utilizada no estudo é creatina? Vale qualquer formato de administração

( ) Sim ( ) Não

**C)** Teve grupo controle?

( ) Sim ( ) Não

**O)** O estudo apresenta valores de marcadores inflamatórios (citocinas e outros marcadores) antes e após as intervenções de creatina?

( ) Sim ( ) Não

**S)** O estudo é um ensaio clínico randomizado e controlado?

( ) Sim ( ) Não

**Artigo 261: Kre-Celazine(®) as a viable treatment for juvenile rheumatoid arthritis/juvenile idiopathic arthritis - a pilot study.**

Golini J, Jones WL

**P)** Os participantes possuem mais de 18 anos de idade?

( ) Sim (X) Não

"The 'Abstract' clearly states: '**sixteen juveniles, ages 7 through 16 years**, experiencing long-standing, unremitting pain and inflammation'. Additionally, the 'MATERIALS AND METHODS' section, under 'Participants' and 'Inclusion criteria', specifies: 'Juveniles **less than 17 years of age** who were suspected of having arthritis' and 'Juvenile less than 17 years of age'. It further notes: 'The average age of all participants was **13.3 years**'. This confirms that the study participants were children and adolescents, not individuals over 18 years of age".

**I)** A intervenção utilizada no estudo é creatina? Vale qualquer formato de administração

( ) Sim ( ) Não

**C)** Teve grupo controle?

( ) Sim ( ) Não

**O)** O estudo apresenta valores de marcadores inflamatórios (citocinas e outros marcadores) antes e após as intervenções de creatina?

( ) Sim ( ) Não

**S)** O estudo é um ensaio clínico randomizado e controlado?

( ) Sim ( ) Não

**Artigo 262: IFN-alpha-induced cortical and subcortical glutamate changes assessed by magnetic resonance spectroscopy.**

Haroon E, Woolwine BJ, Chen X, Pace TW, Parekh S, Spivey JR, Hu XP, Miller AH

**P)** Os participantes possuem mais de 18 anos de idade?

(X) Sim ( ) Não

"Table 1, under 'Demographic, clinical and immunological parameters of the study participants', explicitly provides the age range of the subjects: 'Age mean years (SD) 54.3 (8.6)' for the IFN-alpha-treated group and '55.6 (3.7)' for the HCV-control group. This indicates that all participants were adults and well over 18 years of age".

**I)** A intervenção utilizada no estudo é creatina? Vale qualquer formato de administração

( ) Sim (X) Não

"The title and abstract specify the intervention as 'IFN-alpha-induced cortical and subcortical glutamate changes'. The 'Study Design' section states that 'Patients in the IFN-alpha treatment group... underwent MRS scans... before starting IFN-alpha administration'. While 'creatine' is mentioned in the study, it is as part of the 'Glu/Cr Ratio', indicating it was a **measured metabolite** used for normalization in magnetic resonance spectroscopy, not an administered intervention."

**C)** Teve grupo controle?

( ) Sim ( ) Não

**O)** O estudo apresenta valores de marcadores inflamatórios (citocinas e outros marcadores) antes e após as intervenções de creatina?

( ) Sim ( ) Não

**S)** O estudo é um ensaio clínico randomizado e controlado?

( ) Sim ( ) Não

**Artigo 263: Human kallistatin administration reduces organ injury and improves survival in a**mouse**model of polymicrobial sepsis.**

**P)** Os participantes possuem mais de 18 anos de idade?

( ) Sim (X) Não

"The title of the article explicitly states 'in a **mouse model** of polymicrobial sepsis'. Furthermore, the 'Materials and methods' section describes the study as being conducted on '**mice**' and states that 'Mice were randomly assigned to one of four groups' and 'Mice were killed 24 hr after CLP'. This confirms that the participants were **animals (mice)** and not human subjects aged over 18 years."

**I)** A intervenção utilizada no estudo é creatina? Vale qualquer formato de administração

( ) Sim ( ) Não

**C)** Teve grupo controle?

( ) Sim ( ) Não

**O)** O estudo apresenta valores de marcadores inflamatórios (citocinas e outros marcadores) antes e após as intervenções de creatina?

( ) Sim ( ) Não

**S)** O estudo é um ensaio clínico randomizado e controlado?

( ) Sim ( ) Não

**Artigo 264: Renoprotective effect of paricalcitol via a modulation of the TLR4-NF-κB pathway in ischemia/reperfusion-induced acute kidney injury.**

Lee JW, Kim SC, Ko YS, Lee HY, Cho E, Kim MG, Jo SK, Cho WY, Kim HK

**P)** Os participantes possuem mais de 18 anos de idade?

( ) Sim (X) Não

"The '2. Materials and methods' section, under '2.1. Animal experiment', states: '**Six-to-eight week old, male C57BL/6 mice** (weight, 20–22 g) were purchased from Orient Bio...'. This explicitly indicates that the participants in the study were **animals (mice)**, not human subjects aged over 18 years."

**I)** A intervenção utilizada no estudo é creatina? Vale qualquer formato de administração

( ) Sim ( ) Não

**C)** Teve grupo controle?

( ) Sim ( ) Não

**O)** O estudo apresenta valores de marcadores inflamatórios (citocinas e outros marcadores) antes e após as intervenções de creatina?

( ) Sim ( ) Não

**S)** O estudo é um ensaio clínico randomizado e controlado?

( ) Sim ( ) Não

**Artigo 265: The carotid plaque imaging in acute stroke (CAPIAS) study: protocol and initial baseline data.**

Bayer-Karpinska A, Schwarz F, Wollenweber FA, Poppert H, Boeckh-Behrens T, Becker A, Clevert DA, Nikolaou K, Opherk C, Dichgans M, Saam T

**P)** Os participantes possuem mais de 18 anos de idade?

(X) Sim ( ) Não

"The 'Methods/Design' section explicitly states that '300 patients (age >49 years)' were enrolled. Furthermore, the 'Patient population - Inclusion and exclusion criteria' section specifies that 'Patients of both genders **older than 49 years**' were recruited, and includes '> 18 years of age' as an inclusion criterion."

**I)** A intervenção utilizada no estudo é creatina? Vale qualquer formato de administração

( ) Sim (X) Não

"The 'Methods' section under 'Study design' explicitly states: '**No randomization and study-related interventions are planned**. Patients are treated following current guidelines for the treatment of stroke'. While 'creatinine' is mentioned as a laboratory parameter for exclusion criteria ('Creatinine levels > 2 times the upper limit of the standard range...'), it is a **measured clinical marker** to assess renal function, not an administered intervention."

**C)** Teve grupo controle?

( ) Sim ( ) Não

**O)** O estudo apresenta valores de marcadores inflamatórios (citocinas e outros marcadores) antes e após as intervenções de creatina?

( ) Sim ( ) Não

**S)** O estudo é um ensaio clínico randomizado e controlado?

( ) Sim ( ) Não

**Artigo 266: Control of creatine metabolism by HIF is an endogenous mechanism of barrier regulation in colitis.**

Glover LE, Bowers BE, Saeedi B, Ehrentraut SF, Campbell EL, Bayless AJ, Dobrinskikh E, Kendrick AA, Kelly CJ, Burgess A, Miller L, Kominsky DJ, Jedlicka P, Colgan SP

**P)** Os participantes possuem mais de 18 anos de idade?

( ) Sim (X) Não

"The study primarily utilizes **mouse models** for its experiments. For instance, the 'Significance' section states that 'Dietary creatine supplementation profoundly attenuates the pathogenic course of mucosal inflammation in **mouse colitis models**'. The 'Results' section further details: '**Mice** (n = 15 per group) were fed regular or 2% Cr-supplemented chow' and 'we sought to evaluate the influence of Cr supplementation on mucosal inflammatory pathogenesis in **murine models of IBD**. **Mice** were fed either normal chow or chow supplemented with 2% Cr'. Although human colon biopsy samples from IBD patients are analyzed for CK expression, the direct intervention and assessment of creatine metabolism's role in barrier regulation are conducted in animal subjects."

**I)** A intervenção utilizada no estudo é creatina? Vale qualquer formato de administração

( ) Sim ( ) Não

**C)** Teve grupo controle?

( ) Sim ( ) Não

**O)** O estudo apresenta valores de marcadores inflamatórios (citocinas e outros marcadores) antes e após as intervenções de creatina?

( ) Sim ( ) Não

**S)** O estudo é um ensaio clínico randomizado e controlado?

( ) Sim ( ) Não

**Artigo 267: Spontaneous resolution of lupus nephritis following withdrawal of etanercept.**

Yahya TM, Dhanyamraju S, Harrington TM, Prichard JW

**P)** Os participantes possuem mais de 18 anos de idade?

(X) Sim ( ) Não

"The article describes a clinical case of 'lupus nephritis' and the effects of 'etanercept' therapy on a 'patient'. Lupus nephritis is a condition typically affecting adults. While the exact age is not specified in the provided excerpts, the clinical context and the duration of follow-up (e.g., 'Seven months after stopping etanercept') are characteristic of studies involving adult human subjects, not pediatric patients or animals. Furthermore, other sources discussing human studies often specify 'patients aged ≥ 18 years old' as an inclusion criteria."

**I)** A intervenção utilizada no estudo é creatina? Vale qualquer formato de administração

( ) Sim (X) Não

"The title of the study clearly states 'Spontaneous resolution of lupus nephritis following withdrawal of **etanercept**'. The text consistently refers to 'TNF inhibitors such as **etanercept**' as the focus of the clinical observation, not creatine."

**C)** Teve grupo controle?

( ) Sim ( ) Não

**O)** O estudo apresenta valores de marcadores inflamatórios (citocinas e outros marcadores) antes e após as intervenções de creatina?

( ) Sim ( ) Não

**S)** O estudo é um ensaio clínico randomizado e controlado?

( ) Sim ( ) Não

**Artigo 268: Preliminary differences in peripheral immune markers and brain metabolites between fatigued and non-fatigued breast cancer survivors: a pilot study.**

Zick SM, Zwickey H, Wood L, Foerster B, Khabir T, Wright B, Ichesco E, Sen A, Harris RE

**P)** Os participantes possuem mais de 18 anos de idade?

(X) Sim ( ) Não

"Eligible participants were **women, 18 years of age and older** who have a diagnosis of breast cancer (stage 0 to IIIa); have completed all cancer-related treatments (i.e., surgery, chemotherapy, radiotherapy, immunotherapy, etc.) except for hormonal therapy and/or Herceptin at least 12 weeks previously".

**I)** A intervenção utilizada no estudo é creatina? Vale qualquer formato de administração

( ) Sim (X) Não

"This was a cross-sectional pilot study comparing peripheral inflammatory immune markers (IL-1β, IL-6, TNF-α, CRP) and the following brain metabolites: Cr, the ratio of creatine to total creatine, which is Cr/tCr, Glx, Cho, Ins, NAA, Glx/ NAAcomparing fatigued to non-fatigued BC survivors". The study *measured* creatine (Cr) as a brain metabolite, but creatine itself was **not an intervention** administered to the participants.

**C)** Teve grupo controle?

( ) Sim ( ) Não

**O)** O estudo apresenta valores de marcadores inflamatórios (citocinas e outros marcadores) antes e após as intervenções de creatina?

( ) Sim ( ) Não

**S)** O estudo é um ensaio clínico randomizado e controlado?

( ) Sim ( ) Não

**Artigo 269: [Effects of adipose-derived stem**cells**on renal injury in burn**mice**with sepsis].**

Li N, Hu DH, Wang YJ, Hu XL, Zhang Y, Li XQ, Shi JH, Bai XZ, Cai WX

**P)** Os participantes possuem mais de 18 anos de idade?

( ) Sim ( ) Não [*Excluir*]

**I)** A intervenção utilizada no estudo é creatina? Vale qualquer formato de administração

( ) Sim ( ) Não [*Excluir*]

**C)** Teve grupo controle?

( ) Sim ( ) Não [*Excluir*]

**O)** O estudo apresenta valores de marcadores inflamatórios (citocinas e outros marcadores) antes e após as intervenções de creatina?

( ) Sim ( ) Não [*Excluir*]

**S)** O estudo é um ensaio clínico randomizado e controlado?

( ) Sim ( ) Não [*Excluir*]

**Artigo 270: Metabolite profile analysis reveals functional effects of 28-day vitamin B-6 restriction on one-carbon metabolism and tryptophan catabolic pathways in healthy men and women.**

da Silva VR, Rios-Avila L, Lamers Y, Ralat MA, Midttun Ø, Quinlivan EP, Garrett TJ, Coats B, Shankar MN, Percival SS, Chi YY, Muller KE, Ueland PM, Stacpoole PW, Gregory JF 3rd

**P)** Os participantes possuem mais de 18 anos de idade?

(X) Sim ( ) Não

"The plasma samples analyzed in this study were obtained from 23 **healthy men and women participants** in 2 identical dietary vitamin B-6 restriction studies previously reported. Baseline characteristics of adults who completed the 28-d dietary vitamin B-6 restriction: Men Age, y **24 ± 6**; Women Age, y **25 ± 6**". This explicitly states the participants are adults within this age range, which is over 18.

**I)** A intervenção utilizada no estudo é creatina? Vale qualquer formato de administração

( ) Sim (X) Não

"We report here the concentration of major constituents of one-carbon metabolic processes and the tryptophan catabolic pathway in plasma from 23 healthy men and women before and after a 28-d controlled dietary **vitamin B-6 restriction** (<0.35 mg/d)". The study's main focus is on the effects of restricting vitamin B-6, not on creatine administration. Creatine is a metabolite whose levels were *measured* in response to the vitamin B-6 restriction, as evidenced by statements like "lower creatine (40% pre- and postprandial; P < 0.0001)" and "creatine (40.2% decrease, adjusted P < 0.0001)".

**C)** Teve grupo controle?

( ) Sim ( ) Não

**O)** O estudo apresenta valores de marcadores inflamatórios (citocinas e outros marcadores) antes e após as intervenções de creatina?

( ) Sim ( ) Não

**S)** O estudo é um ensaio clínico randomizado e controlado?

( ) Sim ( ) Não

**Artigo 271: Bioenergetics of the calf muscle in Friedreich ataxia patients measured by 31P-MRS before and after treatment with recombinant human erythropoietin.**

Nachbauer W, Boesch S, Schneider R, Eigentler A, Wanschitz J, Poewe W, Schocke M

**P)** Os participantes possuem mais de 18 anos de idade?

(X) Sim ( ) Não

"Demographics and concentration of metabolites. FRDA baseline... mean **Age 40.00 ± 14.01**".

**I)** A intervenção utilizada no estudo é creatina? Vale qualquer formato de administração

( ) Sim (X) Não

"Aim of the present pilot study was to investigate mitochondrial metabolism of skeletal muscle tissue in FRDA patients and examine effects of **rhuEPO administration** by phosphorus 31 magnetic resonance spectroscopy (31P MRS)". The intervention in this study is recombinant human erythropoietin (rhuEPO), not creatine.

**C)** Teve grupo controle?

( ) Sim ( ) Não

**O)** O estudo apresenta valores de marcadores inflamatórios (citocinas e outros marcadores) antes e após as intervenções de creatina?

( ) Sim ( ) Não

**S)** O estudo é um ensaio clínico randomizado e controlado?

( ) Sim ( ) Não

**Artigo 272: Less is more: combination antibiotic therapy for the treatment of gram-negative bacteremia in pediatric patients.**

Tamma PD, Turnbull AE, Harris AD, Milstone AM, Hsu AJ, Cosgrove SE

**P)** Os participantes possuem mais de 18 anos de idade?

( ) Sim (X) Não

"Retrospective cohort study including **pediatric patients (aged ≤18 years)** with gram-negative bacteremia".

**I)** A intervenção utilizada no estudo é creatina? Vale qualquer formato de administração

( ) Sim ( ) Não

**C)** Teve grupo controle?

( ) Sim ( ) Não

**O)** O estudo apresenta valores de marcadores inflamatórios (citocinas e outros marcadores) antes e após as intervenções de creatina?

( ) Sim ( ) Não

**S)** O estudo é um ensaio clínico randomizado e controlado?

( ) Sim ( ) Não

**Artigo 273: PNU-282987 improves the hemodynamic parameters by alleviating vasopermeability and tissue edema in dogs subjected to a lethal burns shock.**

Hu Q, Du MH, Hu S, Chai JK, Luo HM, Hu XH, Zhang L, Lin ZL, Ma L, Wang H, Sheng ZY

**P)** Os participantes possuem mais de 18 anos de idade?

( ) Sim (X) Não

"Forty pure-bred Beagle **dogs**, aged 16 to 20 months... were used". This indicates that the participants are animals (dogs) and not humans, thus they are not older than 18 human years.

**I)** A intervenção utilizada no estudo é creatina? Vale qualquer formato de administração

( ) Sim ( ) Não

**C)** Teve grupo controle?

( ) Sim ( ) Não

**O)** O estudo apresenta valores de marcadores inflamatórios (citocinas e outros marcadores) antes e após as intervenções de creatina?

( ) Sim ( ) Não

**S)** O estudo é um ensaio clínico randomizado e controlado?

( ) Sim ( ) Não

**Artigo 274: Skeletal muscle ATP kinetics are impaired in frail**mice**.**

Akki A, Yang H, Gupta A, Chacko VP, Yano T, Leppo MK, Steenbergen C, Walston J, Weiss RG

**P)** Os participantes possuem mais de 18 anos de idade?

( ) Sim (X) Não

"Ninety-two-week-old male IL-10 deficient (IL10tm/tm) and age- and sex-matched C57/BL6 (B6) **mice** were used for this study". This indicates that the participants are animals (mice) and not humans, therefore they are not older than 18 human years.

**I)** A intervenção utilizada no estudo é creatina? Vale qualquer formato de administração

( ) Sim ( ) Não

**C)** Teve grupo controle?

( ) Sim ( ) Não

**O)** O estudo apresenta valores de marcadores inflamatórios (citocinas e outros marcadores) antes e após as intervenções de creatina?

( ) Sim ( ) Não

**S)** O estudo é um ensaio clínico randomizado e controlado?

( ) Sim ( ) Não

**Artigo 275: Evaluation of early cerebral metabolic, perfusion and microstructural changes in HCV-positive patients: a pilot study.**

Bladowska J, Zimny A, Knysz B, Małyszczak K, Kołtowska A, Szewczyk P, Gąsiorowski J, Furdal M, Sąsiadek MJ

**P)** Os participantes possuem mais de 18 anos de idade?

(X) Sim ( ) Não

"Fifteen HCV-positive naive patients (6 women and 9 men; **mean age 39.5 years, range 19–58 years**) and 18 normal control subjects (6 women and 12 men; **mean age 34.69 years, range 19–56 years**) were enrolled in the study".

**I)** A intervenção utilizada no estudo é creatina? Vale qualquer formato de administração

( ) Sim (X) Não

"The aim of the study was to **evaluate early metabolic perfusion, and microstructural cerebral changes in patients with the hepatitis C virus (HCV) infection** and normal appearing brain on plain MR using advanced MR techniques". The study focuses on evaluating changes using magnetic resonance techniques in HCV-positive patients, not on administering creatine.

**C)** Teve grupo controle?

( ) Sim ( ) Não

**O)** O estudo apresenta valores de marcadores inflamatórios (citocinas e outros marcadores) antes e após as intervenções de creatina?

( ) Sim ( ) Não

**S)** O estudo é um ensaio clínico randomizado e controlado?

( ) Sim ( ) Não

**Artigo 276: Biocompatibility and in vivo tolerability of a new class of photoresponsive alkoxylphenacyl-based polycarbonates.**

Wehrung D, Sun S, Chamsaz EA, Joy A, Oyewumi MO

**P)** Os participantes possuem mais de 18 anos de idade?

( ) Sim ( ) Não [*Excluir*]

**I)** A intervenção utilizada no estudo é creatina? Vale qualquer formato de administração

( ) Sim ( ) Não [*Excluir*]

**C)** Teve grupo controle?

( ) Sim ( ) Não [*Excluir*]

**O)** O estudo apresenta valores de marcadores inflamatórios (citocinas e outros marcadores) antes e após as intervenções de creatina?

( ) Sim ( ) Não [*Excluir*]

**S)** O estudo é um ensaio clínico randomizado e controlado?

( ) Sim ( ) Não [*Excluir*]

**Artigo 277: Postexercise phosphocreatine recovery, an index of mitochondrial oxidative phosphorylation, is reduced in diabetic patients with lower extremity complications.**

Tecilazich F, Dinh T, Lyons TE, Guest J, Villafuerte RA, Sampanis C, Gnardellis C, Zuo CS, Veves A

**P)** Os participantes possuem mais de 18 anos de idade?

( ) Sim (X) Não

"We studied five groups of subjects **age 40–80 years**".

**I)** A intervenção utilizada no estudo é creatina? Vale qualquer formato de administração

( ) Sim (X) Não

The study aimed "To identify differences in the post-exercise **phosphocreatine (PCr) recovery**, an index of mitochondrial function". The methods involved "Magnetic Resonance Spectroscopic (MRS) measurements to perform continuous measurements of phosphorous metabolites (PCr and Pi) during a 3-minute graded exercise". The study measures phosphocreatine and its recovery as a biomarker of mitochondrial function, rather than administering creatine as an intervention.

**C)** Teve grupo controle?

( ) Sim ( ) Não

**O)** O estudo apresenta valores de marcadores inflamatórios (citocinas e outros marcadores) antes e após as intervenções de creatina?

( ) Sim ( ) Não

**S)** O estudo é um ensaio clínico randomizado e controlado?

( ) Sim ( ) Não

**Artigo 278: Increased renal expression and urinary excretion of TLR4 in acute kidney injury associated with cirrhosis.**

Shah N, Mohamed FE, Jover-Cobos M, Macnaughtan J, Davies N, Moreau R, Paradis V, Moore K, Mookerjee R, Jalan R

**P)** Os participantes possuem mais de 18 anos de idade?

( ) Sim (X) Não

"We studied five groups of subjects... "Age (years)" for the various groups ranged from "36 ± 2.2" for controls to "59 ± 2" for patients with renal dysfunction, all indicating participants well over 18 years of age.

**I)** A intervenção utilizada no estudo é creatina? Vale qualquer formato de administração

( ) Sim (X) Não

The study investigated "Increased renal expression and urinary excretion of TLR4 in acute kidney injury associated with cirrhosis". It involved measuring "Urinary biomarkers, KIM-1, pGST, aGST and a novel biomarker, urinary TLR4" and analyzing "Renal biopsies" for "TLR4 and caspase-3". While serum creatinine was measured as an indicator of renal function ("Renal dysfunction was defined as a creatinine of >133 lmol/L"), the study did not involve the administration of creatine or any form of it as an intervention.

**C)** Teve grupo controle?

( ) Sim ( ) Não

**O)** O estudo apresenta valores de marcadores inflamatórios (citocinas e outros marcadores) antes e após as intervenções de creatina?

( ) Sim ( ) Não

**S)** O estudo é um ensaio clínico randomizado e controlado?

( ) Sim ( ) Não

**Artigo 279: Predictors of preterm birth in patients with mild systemic lupus erythematosus.**

Clowse ME, Wallace DJ, Weisman M, James A, Criscione-Schreiber LG, Pisetsky DS

**P)** Os participantes possuem mais de 18 anos de idade?

(X) Sim ( ) Não

"Maternal age **29.4 (SD 4.7)**".

**I)** A intervenção utilizada no estudo é creatina? Vale qualquer formato de administração

( ) Sim (X) Não

"This study investigates whether markers of SLE activity, inflammation, placental health and renal function could predict preterm birth in women with SLE". The study focuses on predicting outcomes using existing markers, and while "serum creatinine" is mentioned as a renal function marker, it is a measured biomarker and not an administered intervention.

**C)** Teve grupo controle?

( ) Sim ( ) Não

**O)** O estudo apresenta valores de marcadores inflamatórios (citocinas e outros marcadores) antes e após as intervenções de creatina?

( ) Sim ( ) Não

**S)** O estudo é um ensaio clínico randomizado e controlado?

( ) Sim ( ) Não

**Artigo 280: [Free-radical oxidation in liver during experimental widespread purulent peritonitis].**

Kosinets VA, Iarotskaia NN

**P)** Os participantes possuem mais de 18 anos de idade?

( ) Sim ( ) Não [*Excluir*]

**I)** A intervenção utilizada no estudo é creatina? Vale qualquer formato de administração

( ) Sim ( ) Não [*Excluir*]

**C)** Teve grupo controle?

( ) Sim ( ) Não [*Excluir*]

**O)** O estudo apresenta valores de marcadores inflamatórios (citocinas e outros marcadores) antes e após as intervenções de creatina?

( ) Sim ( ) Não [*Excluir*]

**S)** O estudo é um ensaio clínico randomizado e controlado?

( ) Sim ( ) Não [*Excluir*]

**Artigo 281: Cordyceps sinensis protects against renal ischemia/reperfusion injury in**rats**.**

Wang HP, Liu CW, Chang HW, Tsai JW, Sung YZ, Chang LC

**P)** Os participantes possuem mais de 18 anos de idade?

( ) Sim (X) Não

"This study investigated the protective effects of CS in **rats** post-renal ischemia–reperfusion (I/R)". "All **animal** experiments were conducted under guidelines that were approved by the Ethics Committee for Animal Care and Use of E-Da Hospital/I-Shou University (EDAHP98020 and EDAHP99019) and the National Science Council in Taiwan (NSC 99-2320-B-214-001-MY3)".

**I)** A intervenção utilizada no estudo é creatina? Vale qualquer formato de administração

( ) Sim ( ) Não

**C)** Teve grupo controle?

( ) Sim ( ) Não

**O)** O estudo apresenta valores de marcadores inflamatórios (citocinas e outros marcadores) antes e após as intervenções de creatina?

( ) Sim ( ) Não

**S)** O estudo é um ensaio clínico randomizado e controlado?

( ) Sim ( ) Não

**Artigo 282: Role of the transient receptor potential vanilloid type 1 channel in renal inflammation induced by lipopolysaccharide in**mice**.**

Wang Y, Wang DH

**P)** Os participantes possuem mais de 18 anos de idade?

( ) Sim (X) Não

"**Ten-week-old male TRPV1/ mice or C57/BL6 as WT control mice (weighing 26 to 28 g) were used in this study.**"

**I)** A intervenção utilizada no estudo é creatina? Vale qualquer formato de administração

( ) Sim ( ) Não

**C)** Teve grupo controle?

( ) Sim ( ) Não

**O)** O estudo apresenta valores de marcadores inflamatórios (citocinas e outros marcadores) antes e após as intervenções de creatina?

( ) Sim ( ) Não

**S)** O estudo é um ensaio clínico randomizado e controlado?

( ) Sim ( ) Não

**Artigo 283: Reversible loss of N-acetylaspartate after 15-min transient middle cerebral artery occlusion in**rat**: a**longitudinal**study with in vivo proton magnetic resonance spectroscopy.**

Qian J, Qian B, Lei H

**P)** Os participantes possuem mais de 18 anos de idade?

( ) Sim (X) Não

"The right middle cerebral arteries (MCA) of 24 **male Wistar rats**, weighing 200–250 g, were occluded transiently for 15 min with surgical procedures described previously".

**I)** A intervenção utilizada no estudo é creatina? Vale qualquer formato de administração

( ) Sim ( ) Não

**C)** Teve grupo controle?

( ) Sim ( ) Não

**O)** O estudo apresenta valores de marcadores inflamatórios (citocinas e outros marcadores) antes e após as intervenções de creatina?

( ) Sim ( ) Não

**S)** O estudo é um ensaio clínico randomizado e controlado?

( ) Sim ( ) Não

**Artigo 284: Association between change in normal appearing white matter metabolites and intrathecal inflammation in natalizumab-treated multiple sclerosis.**

Mellergård J, Tisell A, Dahlqvist Leinhard O, Blystad I, Landtblom AM, Blennow K, Olsson B, Dahle C, Ernerudh J, Lundberg P, Vrethem M

**P)** Os participantes possuem mais de 18 anos de idade?

(X) Sim ( ) Não

"Natalizumab treatment... was initiated in 27 patients with active MS (Table 1)." According to Table 1, the "Age, years, median (range)" for MS patients was "**36 (24–50)**", indicating all participants were above 18 years of age.

**I)** A intervenção utilizada no estudo é creatina? Vale qualquer formato de administração

( ) Sim (X) Não

The study investigated "Natalizumab treatment (300 mg given intravenously once a month)" and its effects on "intrathecal inflammation" and "neurodegeneration" in multiple sclerosis patients. While "tCr = total creatine" was a metabolite measured by MRS, it was an outcome measure or a reference standard, not an administered intervention.

**C)** Teve grupo controle?

( ) Sim ( ) Não

**O)** O estudo apresenta valores de marcadores inflamatórios (citocinas e outros marcadores) antes e após as intervenções de creatina?

( ) Sim ( ) Não

**S)** O estudo é um ensaio clínico randomizado e controlado?

( ) Sim ( ) Não

**Artigo 285: Elevated urine heparanase levels are associated with proteinuria and decreased renal allograft function.**

Shafat I, Agbaria A, Boaz M, Schwartz D, Baruch R, Nakash R, Ilan N, Vlodavsky I, Weinstein T

**P)** Os participantes possuem mais de 18 anos de idade?

(X) Sim ( ) Não

"Eligible renal transplant recipients... were recruited... using the following criteria: **adult patients aged**".

**I)** A intervenção utilizada no estudo é creatina? Vale qualquer formato de administração

( ) Sim (X) Não

"Here, we quantified blood and urine heparanase levels in renal transplant recipients and patients with chronic kidney disease (CKD), and assessed whether alterations in heparanase levels correlate with proteinuria and renal function". While "creatinine" levels were measured as a renal function marker, it was a measured biomarker, not an administered intervention.

**C)** Teve grupo controle?

( ) Sim ( ) Não

**O)** O estudo apresenta valores de marcadores inflamatórios (citocinas e outros marcadores) antes e após as intervenções de creatina?

( ) Sim ( ) Não

**S)** O estudo é um ensaio clínico randomizado e controlado?

( ) Sim ( ) Não

**Artigo 286: Delayed relief of ureteral obstruction is implicated in the long-term development of renal damage and arterial hypertension in patients with unilateral ureteral injury.**

Lucarelli G, Ditonno P, Bettocchi C, Grandaliano G, Gesualdo L, Selvaggi FP, Battaglia M

**P)** Os participantes possuem mais de 18 anos de idade?

(X) Sim ( ) Não

"A total of 76 patients with obstructive ureteral injury and treated with reconstructive procedures were prospectively enrolled in the study". The study focuses em "iatrogenic obstructive ureteral injury", with the majority of lesions resulting from "obstetric and gynecologic procedures", which are typically realizadas em pacientes adultos. O estudo não menciona pacientes pediátricos nem um limite de idade inferior a 18 anos, implicando que a população do estudo é composta por adultos, em linha com estudos clínicos semelhantes nas fontes fornecidas

**I)** A intervenção utilizada no estudo é creatina? Vale qualquer formato de administração

( ) Sim (X) Não

The study investigated "the role of the timing of the relief of obstruction in the development of renal failure and arterial hypertension". "Serum creatinine" and "estimated creatinine clearance" were measured parameters for renal function, not administered interventions.

**C)** Teve grupo controle?

( ) Sim ( ) Não

**O)** O estudo apresenta valores de marcadores inflamatórios (citocinas e outros marcadores) antes e após as intervenções de creatina?

( ) Sim ( ) Não

**S)** O estudo é um ensaio clínico randomizado e controlado?

( ) Sim ( ) Não

**Artigo 287: [Correction of bioenergetic processes in small intestine during experimental widespread purulent peritonitis].**

Kosinets VA

**P)** Os participantes possuem mais de 18 anos de idade?

( ) Sim ( ) Não [*Excluir*]

**I)** A intervenção utilizada no estudo é creatina? Vale qualquer formato de administração

( ) Sim ( ) Não [*Excluir*]

**C)** Teve grupo controle?

( ) Sim ( ) Não [*Excluir*]

**O)** O estudo apresenta valores de marcadores inflamatórios (citocinas e outros marcadores) antes e após as intervenções de creatina?

( ) Sim ( ) Não [*Excluir*]

**S)** O estudo é um ensaio clínico randomizado e controlado?

( ) Sim ( ) Não [*Excluir*]

**Artigo 288: [The neurodegenerative process in multiple sclerosis and the possible neuroprotective effect of treatment with Β-interferon 1a (avonex)].**

Davydovskaia MV, Boĭko AN, Podoprigora AE, Pronin IN, Kornienko VN, Gusev EI

**P)** Os participantes possuem mais de 18 anos de idade?

(X) Sim ( ) Não

"Patients were included in the treatment group in compliance with international and Russian guidelines for the use of MSCMA. By decision of the Special Committee of the Moscow Health Department, the agent is approved for MS patients **aged 18 years or older**". Furthermore, the age range of the treated patients was "**22–43 (mean 32.1 ± 7.4) years**".

**I)** A intervenção utilizada no estudo é creatina? Vale qualquer formato de administração

( ) Sim (X) Não

The study investigated "The potential neuroprotective effect of treatment with an **interferon β-1a formulation for intramuscular administration (IFNβ-1a, Avonex)**". While "creatine" is mentioned as part of the "N-acetylaspartate/creatine ratio (NAA/Cr)", this is a measured neurochemical marker used to assess neurodegeneration, not an administered intervention.

**C)** Teve grupo controle?

( ) Sim ( ) Não

**O)** O estudo apresenta valores de marcadores inflamatórios (citocinas e outros marcadores) antes e após as intervenções de creatina?

( ) Sim ( ) Não

**S)** O estudo é um ensaio clínico randomizado e controlado?

( ) Sim ( ) Não

**Artigo 289: [Metabolic correction of structural changes in adrenal glands during experimental widespread purulent peritonitis].**

Kosinets VA, Fedotov DN

**P)** Os participantes possuem mais de 18 anos de idade?

( ) Sim ( ) Não [*Excluir*]

**I)** A intervenção utilizada no estudo é creatina? Vale qualquer formato de administração

( ) Sim ( ) Não [*Excluir*]

**C)** Teve grupo controle?

( ) Sim ( ) Não [*Excluir*]

**O)** O estudo apresenta valores de marcadores inflamatórios (citocinas e outros marcadores) antes e após as intervenções de creatina?

( ) Sim ( ) Não [*Excluir*]

**S)** O estudo é um ensaio clínico randomizado e controlado?

( ) Sim ( ) Não [*Excluir*]

**Artigo 290: [Metabolic correction of the lipid-transport system in experimental diffuse purulent peritonitis].**

Kosinets VA

**P)** Os participantes possuem mais de 18 anos de idade?

( ) Sim ( ) Não [*Excluir*]

**I)** A intervenção utilizada no estudo é creatina? Vale qualquer formato de administração

( ) Sim ( ) Não [*Excluir*]

**C)** Teve grupo controle?

( ) Sim ( ) Não [*Excluir*]

**O)** O estudo apresenta valores de marcadores inflamatórios (citocinas e outros marcadores) antes e após as intervenções de creatina?

( ) Sim ( ) Não [*Excluir*]

**S)** O estudo é um ensaio clínico randomizado e controlado?

( ) Sim ( ) Não [*Excluir*]

**Artigo 291: Comparative serum biochemical changes induced by experimental infection of T. brucei and T. congolense in pigs.**

Omeje JN, Anene BM

**P)** Os participantes possuem mais de 18 anos de idade?

( ) Sim (X) Não

"Twenty-two (22) female pigs of about **3–5 months old** were procured". This indicates that the study participants were young pigs, not humans over 18 years of age.

**I)** A intervenção utilizada no estudo é creatina? Vale qualquer formato de administração

( ) Sim ( ) Não

**C)** Teve grupo controle?

( ) Sim ( ) Não

**O)** O estudo apresenta valores de marcadores inflamatórios (citocinas e outros marcadores) antes e após as intervenções de creatina?

( ) Sim ( ) Não

**S)** O estudo é um ensaio clínico randomizado e controlado?

( ) Sim ( ) Não

**Artigo 292: [Correction of protein-lipid composition in liver mitochondria during experimental widespread purulent peritonitis].**

Kosinets VA, Osochuk SS, Iarotskaia NN

**P)** Os participantes possuem mais de 18 anos de idade?

( ) Sim ( ) Não [*Excluir*]

**I)** A intervenção utilizada no estudo é creatina? Vale qualquer formato de administração

( ) Sim ( ) Não [*Excluir*]

**C)** Teve grupo controle?

( ) Sim ( ) Não [*Excluir*]

**O)** O estudo apresenta valores de marcadores inflamatórios (citocinas e outros marcadores) antes e após as intervenções de creatina?

( ) Sim ( ) Não [*Excluir*]

**S)** O estudo é um ensaio clínico randomizado e controlado?

( ) Sim ( ) Não [*Excluir*]

**Artigo 293: [Structural changes in heart at experimental widespread purulent peritonitis].**

Kosinets VA, Samsonova IV, Ryzhkovskaia EL

**P)** Os participantes possuem mais de 18 anos de idade?

( ) Sim ( ) Não

**I)** A intervenção utilizada no estudo é creatina? Vale qualquer formato de administração

( ) Sim ( ) Não

**C)** Teve grupo controle?

( ) Sim ( ) Não

**O)** O estudo apresenta valores de marcadores inflamatórios (citocinas e outros marcadores) antes e após as intervenções de creatina?

( ) Sim ( ) Não

**S)** O estudo é um ensaio clínico randomizado e controlado?

( ) Sim ( ) Não

**Artigo 294: Changes in inflammatory response after endovascular treatment for type B aortic dissection.**

Cheuk BL, Chan YC, Cheng SW

**P)** Os participantes possuem mais de 18 anos de idade?

( ) Sim (X) Não

"The remaining 22 patients were 15 males, 7 females. (**age range 48–78 years**)". Additionally, the "Thoracic endovascular aortic repaired group" had a "Mean age 60.5±11 yrs" and the "Surveillance control group" had an age range of "52–75 yrs".

**I)** A intervenção utilizada no estudo é creatina? Vale qualquer formato de administração

( ) Sim (X) Não

The study's aim was to "investigate the changes in the inflammatory markers after elective **endovascular treatment of Type B aortic dissection** with aneurysm". While "serum creatinine" was a measured parameter, it was not an intervention administered in the study.

**C)** Teve grupo controle?

( ) Sim ( ) Não

**O)** O estudo apresenta valores de marcadores inflamatórios (citocinas e outros marcadores) antes e após as intervenções de creatina?

( ) Sim ( ) Não

**S)** O estudo é um ensaio clínico randomizado e controlado?

( ) Sim ( ) Não

**Artigo 295: [Metabolic immunocorrection treatment of experimental widespread purulent peritonitis].**

Gostishchev VK, Kosinets VA, Matusevich EA, Adamenko GP

**P)** Os participantes possuem mais de 18 anos de idade?

( ) Sim ( ) Não [*Excluir*]

**I)** A intervenção utilizada no estudo é creatina? Vale qualquer formato de administração

( ) Sim ( ) Não [*Excluir*]

**C)** Teve grupo controle?

( ) Sim ( ) Não [*Excluir*]

**O)** O estudo apresenta valores de marcadores inflamatórios (citocinas e outros marcadores) antes e após as intervenções de creatina?

( ) Sim ( ) Não [*Excluir*]

**S)** O estudo é um ensaio clínico randomizado e controlado?

( ) Sim ( ) Não [*Excluir*]

**Artigo 296: Non-invasive in vivo detection of peripheral limb ischemia improvement in the**rat**after adipose tissue-derived stromal cell transplantation.**

Madonna R, Delli Pizzi S, Di Donato L, Mariotti A, Di Carlo L, D'Ugo E, Teberino MA, Merla A, Tartaro A, De Caterina R

**P)** Os participantes possuem mais de 18 anos de idade?

( ) Sim (X) Não

"Six week-old male Sprague-Dawley rats (350–400 g) were used in the study". This indicates that the participants were young rats, not humans over 18 years of age.

**I)** A intervenção utilizada no estudo é creatina? Vale qualquer formato de administração

( ) Sim ( ) Não

**C)** Teve grupo controle?

( ) Sim ( ) Não

**O)** O estudo apresenta valores de marcadores inflamatórios (citocinas e outros marcadores) antes e após as intervenções de creatina?

( ) Sim ( ) Não

**S)** O estudo é um ensaio clínico randomizado e controlado?

( ) Sim ( ) Não

**Artigo 297: Electrical pulse stimulation of cultured human skeletal muscle**cells**as an**in vitro**model of exercise.**

Nikolić N, Bakke SS, Kase ET, Rudberg I, Flo Halle I, Rustan AC, Thoresen GH, Aas V

**P)** Os participantes possuem mais de 18 anos de idade?

( ) Sim (X) Não

The study focuses on "Electrical Pulse Stimulation of **Cultured Human Skeletal Muscle Cells**" or "cultured human myotubes". These are *in vitro* cell cultures, not human subjects with an age.

**I)** A intervenção utilizada no estudo é creatina? Vale qualquer formato de administração

( ) Sim ( ) Não

**C)** Teve grupo controle?

( ) Sim ( ) Não

**O)** O estudo apresenta valores de marcadores inflamatórios (citocinas e outros marcadores) antes e após as intervenções de creatina?

( ) Sim ( ) Não

**S)** O estudo é um ensaio clínico randomizado e controlado?

( ) Sim ( ) Não

**Artigo 298: Metabonomics reveals plasma metabolic changes and inflammatory marker in polycystic ovary syndrome patients.**

Sun L, Hu W, Liu Q, Hao Q, Sun B, Zhang Q, Mao S, Qiao J, Yan X

**P)** Os participantes possuem mais de 18 anos de idade?

(X) Sim ( ) Não

"Polycystic ovary syndrome (PCOS) is a common, clinically heterogeneous endocrine disorder affecting **women of reproductive age**". Additionally, the "Age (years)" for both controls and PCOS patients had mean values of "26.9 ± 4.1" and "27.2 ± 4.1" respectively, indicating participants were over 18.

**I)** A intervenção utilizada no estudo é creatina? Vale qualquer formato de administração

( ) Sim (X) Não

The study aimed to "**establish the metabolic profile of PCOS and compare it with that of controls**" by measuring "contents of small metabolites and lipids in plasma samples" using "nuclear magnetic resonance (NMR)-based techniques". While "creatine" was one of the metabolites whose levels were observed to be "increase(d)... in PCOS patients compared with the controls", it was a measured biomarker, not an intervention administered to the participants.

**C)** Teve grupo controle?

( ) Sim ( ) Não

**O)** O estudo apresenta valores de marcadores inflamatórios (citocinas e outros marcadores) antes e após as intervenções de creatina?

( ) Sim ( ) Não

**S)** O estudo é um ensaio clínico randomizado e controlado?

( ) Sim ( ) Não

**Artigo 299: Urinary markers of renal inflammation in adolescents with Type 1 diabetes mellitus and normoalbuminuria.**

Cherney DZ, Scholey JW, Daneman D, Dunger DB, Dalton RN, Moineddin R, Mahmud FH, Dekker R, Elia Y, Sochett E, Reich HN

**P)** Os participantes possuem mais de 18 anos de idade?

( ) Sim (X) Não

"Participants had to have achieved a minimum Tanner stage 2 for puberty for inclusion, were **between the ages of 11 and 16 years old**".

**I)** A intervenção utilizada no estudo é creatina? Vale qualquer formato de administração

( ) Sim ( ) Não

**C)** Teve grupo controle?

( ) Sim ( ) Não

**O)** O estudo apresenta valores de marcadores inflamatórios (citocinas e outros marcadores) antes e após as intervenções de creatina?

( ) Sim ( ) Não

**S)** O estudo é um ensaio clínico randomizado e controlado?

( ) Sim ( ) Não

**Artigo 300: BGP-15, a PARP-inhibitor, prevents imatinib-induced cardiotoxicity by activating Akt and suppressing JNK and p38 MAP kinases.**

Sarszegi Z, Bognar E, Gaszner B, Kónyi A, Gallyas F Jr, Sumegi B, Berente Z

**P)** Os participantes possuem mais de 18 anos de idade?

( ) Sim (X) Não

"As of date our study is a pioneering work to elaborate the effect of BGP-15 on imatinib-induced energy run-down, oxidative damages, and signaling mechanisms on an **isolated Langendorff perfused rat heart model**". This indicates that the study was conducted on rat hearts, not human participants.

**I)** A intervenção utilizada no estudo é creatina? Vale qualquer formato de administração

( ) Sim	( ) Não

**C)** Teve grupo controle?

( ) Sim ( ) Não

**O)** O estudo apresenta valores de marcadores inflamatórios (citocinas e outros marcadores) antes e após as intervenções de creatina?

( ) Sim	( ) Não

**S)** O estudo é um ensaio clínico randomizado e controlado?

( ) Sim	( ) Não

**Artigo 301: Systemic soluble tumor necrosis factor receptors 1 and 2 are associated with severity of diabetic retinopathy in Hispanics.**

Kuo JZ, Guo X, Klein R, Klein BE, Cui J, Rotter JI, Ipp E, Chen YD

**P)** Os participantes possuem mais de 18 anos de idade?

(X) Sim	( ) Não

"Four hundred seventy-three Hispanics type II diabetic subjects, in families ascertained via proband with DR". "The mean values of sTNF-R1 ± SEM were: no DR 2.37 ± 0.13, mild NPDR 2.15 ± 0.09, moderate NPDR 3.09 ± 0.24, severe NPDR 3.25 ± 0.46, PDR 5.02 ± 0.61 ng/ml; and of sTNF-R2 ± SEM were: no DR 6.04 ± 0.20, mild NPDR 6.25 ± 0.52, moderate NPDR 7.96 ± 0.70, severe NPDR 8.14 ± 1.13, and PDR 14.83 ± 1.68 ng/ml, respectively (Table 2)". The mean age of participants was approximately **53.7 years for those without retinopathy and 53.6 years for those with retinopathy**. This age range indicates that all participants were well over 18 years of age.

**I)** A intervenção utilizada no estudo é creatina? Vale qualquer formato de administração

( ) Sim	(X) Não

The study measured "the plasma levels of three inflammatory biomarkers, serum amyloid A protein (SAA) and soluble tumor necrosis factor receptors 1 (sTNF-R1) and 2 (sTNF-R2)". There is no mention of creatine or any form of intervention throughout the methods or results sections of the study. It is described as a "Prospective, nonrandomized, cross-sectional, family-based observational cohort study".

**C)** Teve grupo controle?

( ) Sim ( ) Não

**O)** O estudo apresenta valores de marcadores inflamatórios (citocinas e outros marcadores) antes e após as intervenções de creatina?

( ) Sim	( ) Não

**S)** O estudo é um ensaio clínico randomizado e controlado?

( ) Sim	( ) Não

**Artigo 302: Interleukin-1beta does not affect the energy metabolism of** rat **organotypic hippocampal-slice cultures.**

Saggu R, Morrison B 3rd, Lowe JP, Pringle AK

**P)** Os participantes possuem mais de 18 anos de idade?

( ) Sim	(X) Não

"Briefly, **eight to ten-day-old male Wistar rat pups** bred in-house, Biological Services Unit, University of Southampton) were decapitated without anaesthesia, and the hippocampi bluntly dissected out". "Adult male Wistar albino rats, weighing 170–200 g, were used in this study". However, the primary study "Interleukin-1beta does not affect the energy metabolism of rat organotypic hippocampal-slice cultures" specifically used "eight to ten-day-old male Wistar rat pups" to prepare the organotypic hippocampal-slice cultures. Given that these are **rat pups**, they are not over 18 years of age.

**I)** A intervenção utilizada no estudo é creatina? Vale qualquer formato de administração

( ) Sim	( ) Não

**C)** Teve grupo controle?

( ) Sim ( ) Não

**O)** O estudo apresenta valores de marcadores inflamatórios (citocinas e outros marcadores) antes e após as intervenções de creatina?

( ) Sim	( ) Não

**S)** O estudo é um ensaio clínico randomizado e controlado?

( ) Sim	( ) Não

**Artigo 303: Chemokine receptor CCR1 disruption limits renal damage in a** murine **model of hemolytic uremic syndrome.**

Ramos MV, Auvynet C, Poupel L, Rodero M, Mejias MP, Panek CA, Vanzulli S, Combadiere C, Palermo M

**P)** Os participantes possuem mais de 18 anos de idade?

( ) Sim	(X) Não

The study states, "Because the chemokine receptor CCR1 participates in the pathogenesis of several renal diseases by orchestrating myeloid cell kidney infiltration, we specifically addressed the contribution of CCR1 in a **murine model** of HUS". It also specifies, "**CCR1-/- and control C57BL/6 mice** were injected i.v. with Stx2 and survival rate was evaluated". Since the participants are **mice**, they are not over 18 years of age.

**I)** A intervenção utilizada no estudo é creatina? Vale qualquer formato de administração

( ) Sim	( ) Não

**C)** Teve grupo controle?

( ) Sim ( ) Não

**O)** O estudo apresenta valores de marcadores inflamatórios (citocinas e outros marcadores) antes e após as intervenções de creatina?

( ) Sim	( ) Não

**S)** O estudo é um ensaio clínico randomizado e controlado?

( ) Sim	( ) Não

**Artigo 304: Testing a single monthly dose of darbepoetin alpha to maintain hemoglobin levels in continuous ambulatory peritoneal dialysis patients.**

Suzuki H, Inoue T, Watanabe Y, Kikuta T, Sato T, Tsuda M, Uchida K

**P)** Os participantes possuem mais de 18 anos de idade?

( ) Sim	( ) Não [*Excluir*]

**I)** A intervenção utilizada no estudo é creatina? Vale qualquer formato de administração

( ) Sim	(	) Não [*Excluir*]

**C)** Teve grupo controle?

(	) Sim ( ) Não [*Excluir*]

**O)** O estudo apresenta valores de marcadores inflamatórios (citocinas e outros marcadores) antes e após as intervenções de creatina?

( ) Sim	(	) Não [*Excluir*]

**S)** O estudo é um ensaio clínico randomizado e controlado?

(	) Sim	( ) Não

**Artigo 305: Effects of anti-viral therapy and HCV clearance on cerebral metabolism and cognition**

Byrnes V, Miller A, Lowry D, Hill E, Weinstein C, Alsop D, Lenkinski R, Afdhal NH

**P)** Os participantes possuem mais de 18 anos de idade?

(X) Sim	( ) Não

"Fifteen non-cirrhotic HCV positive subjects underwent 1H MR spectroscopy (MRS) before, during, and after treatment with PIFN/R.". The "HCV treatment group (n = 15)" had a mean age of "**48.1 yr (5.8 yr)**". This age clearly indicates that participants were over 18 years old.

**I)** A intervenção utilizada no estudo é creatina? Vale qualquer formato de administração

( ) Sim	(X) Não

The study aimed to evaluate "the effect of **pegylated interferon/ribavirin (PIFN/R)** and HCV clearance on cerebral metabolism, and neuropsychological performance". Creatine (Cr) is mentioned as a "control metabolite" measured by 1H MR spectroscopy, not as an intervention or treatment being administered.

**C)** Teve grupo controle?

( ) Sim ( ) Não

**O)** O estudo apresenta valores de marcadores inflamatórios (citocinas e outros marcadores) antes e após as intervenções de creatina?

( ) Sim	( ) Não

**S)** O estudo é um ensaio clínico randomizado e controlado?

( ) Sim	( ) Não

**Artigo 306: Ozone therapy prevents renal inflammation and fibrosis in a** rat **model of acute pyelonephritis.**

Caliskan B, Guven A, Ozler M, Cayci T, Ozcan A, Bedir O, Surer I, Korkmaz A

**P)** Os participantes possuem mais de 18 anos de idade?

( ) Sim	(X) Não

"Sprague-Dawley **male rats (200 – 250 g)** were housed in an air-conditioned room with 12-h light and dark cycles, where the temperature and relative humidity were kept constant.". Since the study participants are **rats**, they are not over 18 years of age.

**I)** A intervenção utilizada no estudo é creatina? Vale qualquer formato de administração

( ) Sim	( ) Não

**C)** Teve grupo controle?

( ) Sim ( ) Não

**O)** O estudo apresenta valores de marcadores inflamatórios (citocinas e outros marcadores) antes e após as intervenções de creatina?

( ) Sim	( ) Não

**S)** O estudo é um ensaio clínico randomizado e controlado?

( ) Sim	( ) Não

**Artigo 307: Effects of atorvastatin on bone metabolism and bone mineral density in Wistar** rats**.**

Chang B, Yang J, Li H, Lu S, Chen L, Fang P

**P)** Os participantes possuem mais de 18 anos de idade?

( ) Sim	(X) Não

"Sixty healthy **male Wistar rats** were randomly divided into one control group treated with vehicle alone and three drug treatment groups...". Since the participants are **rats**, they are not over 18 years of age.

**I)** A intervenção utilizada no estudo é creatina? Vale qualquer formato de administração

( ) Sim	( ) Não

**C)** Teve grupo controle?

( ) Sim ( ) Não

**O)** O estudo apresenta valores de marcadores inflamatórios (citocinas e outros marcadores) antes e após as intervenções de creatina?

( ) Sim	( ) Não

**S)** O estudo é um ensaio clínico randomizado e controlado?

( ) Sim	( ) Não

**Artigo 308: Protective effect of telmisartan against cadmium-induced nephrotoxicity in** mice**.**

Fouad AA, Jresat I

**P)** Os participantes possuem mais de 18 anos de idade?

( ) Sim	(X) Não

The study states, "**Male albino mice, weighing 25–30 g** were obtained from the Animal House, College of Medicine, King Faisal University". Since the participants are **mice**, they are not over 18 years of age.

**I)** A intervenção utilizada no estudo é creatina? Vale qualquer formato de administração

( ) Sim	( ) Não

**C)** Teve grupo controle?

( ) Sim ( ) Não

**O)** O estudo apresenta valores de marcadores inflamatórios (citocinas e outros marcadores) antes e após as intervenções de creatina?

( ) Sim	( ) Não

**S)** O estudo é um ensaio clínico randomizado e controlado?

( ) Sim	( ) Não

**Artigo 309: Effects of caffeic acid phenethyl ester on endotoxin-induced cardiac stress in** rats**: a possible mechanism of protection.**

Motawi TK, Darwish HA, Abd El Tawab AM

**P)** Os participantes possuem mais de 18 anos de idade?

( ) Sim	(X) Não

"**Adult male Wistar albino rats, weighing 170–200 g, were used in this study.**". Since the participants are **rats**, they are not over 18 years of age.

**I)** A intervenção utilizada no estudo é creatina? Vale qualquer formato de administração

( ) Sim	( ) Não

**C)** Teve grupo controle?

( ) Sim ( ) Não

**O)** O estudo apresenta valores de marcadores inflamatórios (citocinas e outros marcadores) antes e após as intervenções de creatina?

( ) Sim	( ) Não

**S)** O estudo é um ensaio clínico randomizado e controlado?

( ) Sim	( ) Não

**Artigo 310: Reduced glomerular filtration rate, inflammation and HDL cholesterol as main determinants of superoxide production in non-dialysis chronic kidney disease patients.**

Morena M, Patrier L, Jaussent I, Bargnoux AS, Dupuy AM, Badiou S, Leray-Moragues H, Klouche K, Canaud B, Cristol JP

**P)** Os participantes possuem mais de 18 anos de idade?

(X) Sim	( ) Não

"Inclusion criteria were **age ≥ 18 years old**". The median age of the patients was "69.5 [27.4 – 94.6] years".

**I)** A intervenção utilizada no estudo é creatina? Vale qualquer formato de administração

( ) Sim	(X) Não

"to evaluate O2• determinants in this population" of "non-dependent dialysis CKD patients". It evaluated "renal function..., inflammation, lipids, nutritional and bone mineral as well as clinical parameters". Creatinine is mentioned as a measure of renal function ("creatinine plasma level") but not as an intervention or administered substance.

**C)** Teve grupo controle?

( ) Sim ( ) Não

**O)** O estudo apresenta valores de marcadores inflamatórios (citocinas e outros marcadores) antes e após as intervenções de creatina?

( ) Sim	( ) Não

**S)** O estudo é um ensaio clínico randomizado e controlado?

( ) Sim	( ) Não

**Artigo 311: Metabolic assessment of monofocal acute inflammatory demyelination using MR spectroscopy and (11)C-methionine-, (11)C-choline-, and (18)F-fluorodeoxyglucose-PET.**

Takenaka S, Shinoda J, Asano Y, Aki T, Miwa K, Ito T, Yokoyama K, Iwama T

**P)** Os participantes possuem mais de 18 anos de idade?

(X) Sim	( ) Não

Six patients with MAID (3 men and 3 women; mean age, 46.7 years), 19 with AA (14 men and 5 women; mean age, 50.0 years), and 21 with GBM (14 men and 7 women; mean age, 58.2 years) were subjects in this study.

**I)** A intervenção utilizada no estudo é creatina? Vale qualquer formato de administração

( ) Sim	(X) Não

"The study is a 'Metabolic assessment' using 'MR spectroscopy and (11)C-methionine-, (11)C-choline-, and (18)F-fluorodeoxyglucose-PET'. Creatine is a metabolite that can be *measured* in such studies, but it is not administered as an intervention or treatment to the participants."

**C)** Teve grupo controle?

( ) Sim ( ) Não

**O)** O estudo apresenta valores de marcadores inflamatórios (citocinas e outros marcadores) antes e após as intervenções de creatina?

( ) Sim	( ) Não

**S)** O estudo é um ensaio clínico randomizado e controlado?

( ) Sim	( ) Não

**Artigo 312: Chemokines in cerebrospinal fluid correlate with cerebral metabolite patterns in HIV-infected individuals.**

Letendre SL, Zheng JC, Kaul M, Yiannoutsos CT, Ellis RJ, Taylor MJ, Marquie-Beck J, Navia B

**P)** Os participantes possuem mais de 18 anos de idade?

(X) Sim	( ) Não

"The study population for Article 312, drawing data from ACTG protocols, primarily consists of middle-aged subjects. Table 1, which summarizes subject characteristics, indicates a **"Median (IQR) 42 (36–46)" for age at baseline**. This explicitly states that the participants are over 18 years old."

**I)** A intervenção utilizada no estudo é creatina? Vale qualquer formato de administração

( ) Sim	(X) Não

"The study's primary objective was to determine the relationship between chemokines in cerebrospinal fluid (CSF) and cerebral metabolites measured by proton magnetic resonance spectroscopy (MRS) in HIV-infected individuals. Creatine (Cr) is mentioned as a **'control metabolite'** and is used as a **reference point for ratios** with other metabolites like N-acetyl aspartate (NAA) and myo-inositol (MI) (e.g., NAA/Cr ratios, MI/Cr ratios). It is a measured brain metabolite, not an intervention or administered substance in the study."

**C)** Teve grupo controle?

( ) Sim ( ) Não

**O)** O estudo apresenta valores de marcadores inflamatórios (citocinas e outros marcadores) antes e após as intervenções de creatina?

( ) Sim	( ) Não

**S)** O estudo é um ensaio clínico randomizado e controlado?

( ) Sim	( ) Não

**Artigo 313: N-terminal-proB-type natriuretic peptide predicts cardiovascular disease events in HIV-infected patients.**

Duprez DA, Neuhaus J, Tracy R, Kuller LH, Deeks SG, Orkin C, Stoehr A, Woolley IJ, Neaton JD

**P)** Os participantes possuem mais de 18 anos de idade?

(X) Sim	( ) Não

"The study investigates 'cardiovascular disease events in HIV-infected patients', which are conditions typically affecting adults. The participants were drawn from the 'SMART study', which is a large clinical trial for HIV management, and the study analyzes factors like 'hypertension, diabetes and dyslipidemia', and 'prior CVD', which are adult-onset conditions. The study also mentions matching controls 'on country, age (within 5 years), sex' and discusses how 'older age' was associated with higher NT-proBNP levels, implying an adult cohort. Although a precise age range is not explicitly given, the context of the study population and the diseases studied strongly indicate that participants are over 18 years of age."

**I)** A intervenção utilizada no estudo é creatina? Vale qualquer formato de administração

( ) Sim	(X) Não

"The study is primarily an observational 'nested case–control study' that aimed to quantify the risk of CVD events associated with N-terminal pro-B-type natriuretic peptide (NT-proBNP) levels. While 'plasma creatinine' was measured as a covariate, creatine was not used as an intervention or administered substance in any format. The patients were likely receiving 'antiretroviral therapy (ART)' as part of the broader SMART study."

**C)** Teve grupo controle?

( ) Sim ( ) Não

**O)** O estudo apresenta valores de marcadores inflamatórios (citocinas e outros marcadores) antes e após as intervenções de creatina?

( ) Sim	( ) Não

**S)** O estudo é um ensaio clínico randomizado e controlado?

( ) Sim	( ) Não

**Artigo 314: Hsp72 is an early and sensitive biomarker to detect acute kidney injury.**

Barrera-Chimal J, Pérez-Villalva R, Cortés-González C, Ojeda-Cervantes M, Gamba G, Morales-Buenrostro LE, Bobadilla NA

**P)** Os participantes possuem mais de 18 anos de idade?

( ) Sim	(X) Não

"The primary experimental subjects in this study are 'rats', as indicated by sections such as 'Figure 1. Renal functional parameters in rats', 'Figure 3. Renal Hsp72 expression in rats', and 'Figure 6. Urinary Hsp72 levels in rats'. While the study mentions that 'these experimental findings were corroborated in humans' and presents 'Urinary Hsp72 levels as a biomarker of AKI in humans', the detailed methodology and majority of reported data concern the animal model. Human age (over 18 years) is not applicable to rats."

**I)** A intervenção utilizada no estudo é creatina? Vale qualquer formato de administração

( ) Sim	( ) Não

**C)** Teve grupo controle?

( ) Sim ( ) Não

**O)** O estudo apresenta valores de marcadores inflamatórios (citocinas e outros marcadores) antes e após as intervenções de creatina?

( ) Sim	( ) Não

**S)** O estudo é um ensaio clínico randomizado e controlado?

( ) Sim	( ) Não

**Artigo 315: Effects of ligustilide on lipopolysaccharide-induced endotoxic shock in** rabbits**.**

Shao M, Qu K, Liu K, Zhang Y, Zhang L, Lian Z, Chen T, Liu J, Wu A, Tang Y, Zhu H

**P)** Os participantes possuem mais de 18 anos de idade?

( ) Sim	(X) Não

"The study explicitly states that 'Japanese white rabbits (weight, 2.5–3.0 kg; **age, 5–6 months**; half male and half female)' were used as animals. Therefore, the participants are not humans over 18 years of age."

**I)** A intervenção utilizada no estudo é creatina? Vale qualquer formato de administração

( ) Sim	( ) Não

**C)** Teve grupo controle?

( ) Sim ( ) Não

**O)** O estudo apresenta valores de marcadores inflamatórios (citocinas e outros marcadores) antes e após as intervenções de creatina?

( ) Sim	( ) Não

**S)** O estudo é um ensaio clínico randomizado e controlado?

( ) Sim	( ) Não

**Artigo 316: The obesity paradox and mortality associated with surrogates of body size and muscle mass in patients receiving hemodialysis.**

Kalantar-Zadeh K, Streja E, Kovesdy CP, Oreopoulos A, Noori N, Jing J, Nissenson AR, Krishnan M, Kopple JD, Mehrotra R, Anker SD

**P)** Os participantes possuem mais de 18 anos de idade?

(X) Sim	( ) Não

"The study investigates a 'contemporary cohort of 121,762 patients receiving HD', which refers to hemodialysis, a treatment for end-stage renal disease predominantly affecting adults. Although a specific age range for inclusion in this study is not explicitly stated, the mean age in a similar patient cohort (CKD patients, Article 310) was '69.5 [27.4 – 94.6]' years, indicating an adult population. Furthermore, the discussion of 'obesity paradox' and 'mortality' in hemodialysis patients typically pertains to adult and elderly populations."

**I)** A intervenção utilizada no estudo é creatina? Vale qualquer formato de administração

( ) Sim	(X) Não

"The study focuses on observational associations between 'serum creatinine level, a likely surrogate of larger muscle mass,' and 'survival' in patients already undergoing hemodialysis. While 'creatinine, which is formed from creatine,' is mentioned as a biomarker reflecting muscle mass, creatine itself was not administered or used as an intervention in any format."

**C)** Teve grupo controle?

( ) Sim ( ) Não

**O)** O estudo apresenta valores de marcadores inflamatórios (citocinas e outros marcadores) antes e após as intervenções de creatina?

( ) Sim	( ) Não

**S)** O estudo é um ensaio clínico randomizado e controlado?

( ) Sim	( ) Não

**Artigo 317: Inhibition of 20-HETE synthesis and action protects the kidney from ischemia/reperfusion injury.**

Hoff U, Lukitsch I, Chaykovska L, Ladwig M, Arnold C, Manthati VL, Fuller TF, Schneider W, Gollasch M, Muller DN, Flemming B, Seeliger E, Luft FC, Falck JR, Dragun D, Schunck WH

**P)** Os participantes possuem mais de 18 anos de idade?

( ) Sim	(X) Não

"The study explicitly states the use of 'Inbred male Lewis (Lew, RT1) rats, aged 10 weeks' as the animal model. Therefore, the participants are not humans over 18 years of age."

**I)** A intervenção utilizada no estudo é creatina? Vale qualquer formato de administração

( ) Sim	( ) Não

**C)** Teve grupo controle?

( ) Sim ( ) Não

**O)** O estudo apresenta valores de marcadores inflamatórios (citocinas e outros marcadores) antes e após as intervenções de creatina?

( ) Sim	( ) Não

**S)** O estudo é um ensaio clínico randomizado e controlado?

( ) Sim	( ) Não

**Artigo 318: Evidence for neuroinflammation and neuroprotection in HCV infection-associated encephalopathy.**

Bokemeyer M, Ding XQ, Goldbecker A, Raab P, Heeren M, Arvanitis D, Tillmann HL, Lanfermann H, Weissenborn K

**P)** Os participantes possuem mais de 18 anos de idade?

(X) Sim	( ) Não

"The study group consisted of 53 HCV-replicating patients... **aged 52–69 years (range: 32–71 years)**. The healthy control subjects were in the age of **49.7 ± 6.2 years (range: 36–58 years)** and the MRS controls had a mean age of **52.5 ± 12.8 years (range: 29–83 years)**. All participants were clearly above 18 years of age."

**I)** A intervenção utilizada no estudo é creatina? Vale qualquer formato de administração

( ) Sim	(X) Não

"The study measured **creatine** as a brain metabolite using magnetic resonance spectroscopy (MRS). It evaluated the *concentrations* of metabolites like choline and creatine and their *ratios* as indicators of neuroinflammation and brain function. There is no mention of creatine being administered as an intervention in any format."

**C)** Teve grupo controle?

( ) Sim ( ) Não

**O)** O estudo apresenta valores de marcadores inflamatórios (citocinas e outros marcadores) antes e após as intervenções de creatina?

( ) Sim	( ) Não

**S)** O estudo é um ensaio clínico randomizado e controlado?

( ) Sim	( ) Não

**Artigo 319: Effect of surgical portosystemic shunt on** prevalence **of minimal hepatic encephalopathy in children with extrahepatic portal venous obstruction: assessment by magnetic resonance imaging and psychometry.**

Srivastava A, Yadav SK, Lal R, Yachha SK, Thomas MA, Saraswat VA, Gupta RK

**P)** Os participantes possuem mais de 18 anos de idade?

( ) Sim	(X) Não

"The study explicitly states that 'Forty-two **children** with extrahepatic portal venous obstruction... and 20 **healthy children** were evaluated'. Furthermore, the age demographics are provided as '20 healthy controls (mean age **10.6 2.9 years**... and 42 EHPVO patients... [group A **11.4 3.3 years**... group B **13.1 3.9 years**])'. All participants are clearly under 18 years of age."

**I)** A intervenção utilizada no estudo é creatina? Vale qualquer formato de administração

( ) Sim	( ) Não

**C)** Teve grupo controle?

( ) Sim ( ) Não

**O)** O estudo apresenta valores de marcadores inflamatórios (citocinas e outros marcadores) antes e após as intervenções de creatina?

( ) Sim	( ) Não

**S)** O estudo é um ensaio clínico randomizado e controlado?

( ) Sim	( ) Não

**Artigo 320: Quantitative metabolomic profiling of serum and urine in DSS-induced ulcerative colitis of** mice **by (1)H NMR spectroscopy.**

Schicho R, Nazyrova A, Shaykhutdinov R, Duggan G, Vogel HJ, Storr M

**P)** Os participantes possuem mais de 18 anos de idade?

( ) Sim	(X) Não

"The study explicitly states that 'We induced an experimental form of UC in **mice** by treatment with dextran sulfate sodium (DSS)'. Furthermore, it refers to 'DSS-treated **mice**' and provides weight measurements for 'control group 29.3 ( 0.9 g vs DSS-treated group 24.5 ( 1 g; means ( sem; n ) 5-11; Student’s t test)'. This confirms that the participants are animals and not humans aged over 18."

**I)** A intervenção utilizada no estudo é creatina? Vale qualquer formato de administração

( ) Sim	( ) Não

**C)** Teve grupo controle?

( ) Sim ( ) Não

**O)** O estudo apresenta valores de marcadores inflamatórios (citocinas e outros marcadores) antes e após as intervenções de creatina?

( ) Sim	( ) Não

**S)** O estudo é um ensaio clínico randomizado e controlado?

( ) Sim	( ) Não

**Artigo 321: Activin IIB receptor blockade attenuates dystrophic pathology in a** mouse **model of Duchenne muscular dystrophy.**

Morine KJ, Bish LT, Selsby JT, Gazzara JA, Pendrak K, Sleeper MM, Barton ER, Lee SJ, Sweeney HL

**P)** Os participantes possuem mais de 18 anos de idade?

( ) Sim	(X) Não

"The study explicitly states the use of the '**mdx mouse model of Duchenne muscular dystrophy**' and mentions that '**Young mdx mice** were injected' and 'Six-week-old **mdx mice** (n = 5 control, n = 5 treated) were injected'. This indicates that the participants are animals, specifically mice, and not humans over 18 years of age."

**I)** A intervenção utilizada no estudo é creatina? Vale qualquer formato de administração

( ) Sim	( ) Não

**C)** Teve grupo controle?

( ) Sim ( ) Não

**O)** O estudo apresenta valores de marcadores inflamatórios (citocinas e outros marcadores) antes e após as intervenções de creatina?

( ) Sim	( ) Não

**S)** O estudo é um ensaio clínico randomizado e controlado?

( ) Sim	( ) Não

**Artigo 322: Parkinson's disease: mitochondrial molecular pathology, inflammation, statins, and therapeutic neuroprotective nutrition.**

Kones R

**P)** Os participantes possuem mais de 18 anos de idade?

(X) Sim	( ) Não

"In a prospective study of 50,926 Finns without PD at baseline, Hu et al121 found a significant association between serum total cholesterol level and risk of PD after a median follow-up of 18.1 years. Curiously, the association held only for subjects age 54 years or less, well below the average age of PD patients". This indicates that the subjects of the reviewed studies are human and include adults, with many being older adults, and even those described as "54 years or less" are still over 18.

**I)** A intervenção utilizada no estudo é creatina? Vale qualquer formato de administração

(X) Sim	( ) Não

The article is a review that discusses creatine as a therapeutic agent. It mentions "These observations present possible mechanisms for neuroprotective effects of creatine that are not energy related". Furthermore, it explicitly refers to clinical research involving creatine, stating, "A randomized, double-blind, futility clinical trial of creatine and minocycline in early Parkinson disease" and "NIH announces phase III clinical trial of creatine for Parkinson’s disease".

**C)** Teve grupo controle?

(X) Sim ( ) Não

The review discusses clinical trials that included control groups. For example, it mentions "A randomized, double-blind, futility clinical trial of creatine and minocycline in early Parkinson disease". A randomized, double-blind clinical trial inherently involves a control group.

**O)** O estudo apresenta valores de marcadores inflamatórios (citocinas e outros marcadores) antes e após as intervenções de creatina?

( ) Sim	(X) Não

This article is a review and thus does not present original quantitative data from interventions. While it discusses inflammation in the context of Parkinson's disease and mentions that "Statins... reduce superoxide production... and reduces expression of TNF-α and NO. Statins suppress the release of cytokines that leads to induction of NOS and NO release in microglia", it does not present specific quantitative values (e.g., "before and after") of inflammatory markers directly associated with creatine interventions.

**S)** O estudo é um ensaio clínico randomizado e controlado?

( ) Sim	( ) Não

**Artigo 323: Urinary cystatin C is diagnostic of acute kidney injury and sepsis, and predicts mortality in the intensive care unit.**

Nejat M, Pickering JW, Walker RJ, Westhuyzen J, Shaw GM, Frampton CM, Endre ZH

**P)** Os participantes possuem mais de 18 anos de idade?

(X) Sim	( ) Não

"The study explicitly states its exclusion criteria as '1. **under 16 years of age**'. Furthermore, it provides the demographic data that 'The **mean age was 60 ± 18 years** and 39% were women'. This confirms that the participants are adults and thus over 18 years of age."

**I)** A intervenção utilizada no estudo é creatina? Vale qualquer formato de administração

( ) Sim	(X) Não

"The study focuses on evaluating the utility of **urinary cystatin C (uCysC)** as a diagnostic marker for acute kidney injury and sepsis, and as a predictor of mortality. While **plasma creatinine (pCr)** and **urinary creatinine (uCr)** are measured as part of routine biochemical assessments and for normalization purposes, **creatine itself is not an intervention or administered substance in this study**."

**C)** Teve grupo controle?

( ) Sim ( ) Não

**O)** O estudo apresenta valores de marcadores inflamatórios (citocinas e outros marcadores) antes e após as intervenções de creatina?

( ) Sim	( ) Não

**S)** O estudo é um ensaio clínico randomizado e controlado?

( ) Sim	( ) Não

**Artigo 324: [Influence of rhBMP-2 on the renal tissue of** rat **with renal ischemia reperfusion injury and its molecular mechanism].**

Yang S, Pei L

**P)** Os participantes possuem mais de 18 anos de idade?

( ) Sim	( ) Não [*Excluir*]

**I)** A intervenção utilizada no estudo é creatina? Vale qualquer formato de administração

( ) Sim	(	) Não [*Excluir*]

**C)** Teve grupo controle?

(	) Sim ( ) Não [*Excluir*]

**O)** O estudo apresenta valores de marcadores inflamatórios (citocinas e outros marcadores) antes e após as intervenções de creatina?

( ) Sim	(	) Não [*Excluir*]

**S)** O estudo é um ensaio clínico randomizado e controlado?

(	) Sim	( ) Não

**Artigo 325: Prenatal exposure to lipopolysaccharide alters the intrarenal renin-angiotensin system and renal damage in offspring** rats**.**

Hao XQ, Zhang HG, Yuan ZB, Yang DL, Hao LY, Li XH

**P)** Os participantes possuem mais de 18 anos de idade?

( ) Sim	(X) Não

"The study explicitly states that it investigated 'Prenatal exposure to lipopolysaccharide alters the intrarenal renin–angiotensin system and renal damage in **offspring rats**'. Furthermore, it refers to '7-week-old **offspring rats**', confirming that the participants are animals and not humans over 18 years of age."

**I)** A intervenção utilizada no estudo é creatina? Vale qualquer formato de administração

( ) Sim	( ) Não

**C)** Teve grupo controle?

( ) Sim ( ) Não

**O)** O estudo apresenta valores de marcadores inflamatórios (citocinas e outros marcadores) antes e após as intervenções de creatina?

( ) Sim	( ) Não

**S)** O estudo é um ensaio clínico randomizado e controlado?

( ) Sim	( ) Não

**Artigo 326: Brain involvement in rheumatoid arthritis: a magnetic resonance spectroscopy study.**

Emmer BJ, van der Bijl AE, Huizinga TW, Breedveld FC, Steens SC, Th Bosma GP, van Buchem MA, van der Grond J

**P)** Os participantes possuem mais de 18 anos de idade?

(X) Sim	( ) Não

"The study involved '35 RA patients (6 men and 29 women; **mean ± SD age 51.8 ± 14.6 years**) and 28 healthy age- and sex-matched control subjects (9 men and 19 women; **mean ± SD age 50.2 ± 10.4 years**)', clearly indicating that all participants are adults and over 18 years of age."

**I)** A intervenção utilizada no estudo é creatina? Vale qualquer formato de administração

( ) Sim	(X) Não

"The study utilized proton magnetic resonance spectroscopy (1H-MRS) to examine the 'influence of inflammation on cerebral metabolism' and *measured* 'the total creatine peaks' as a metabolite ratio (e.g., choline to creatine ratio). **Creatine was measured as an endogenous metabolite, not administered as an intervention or therapeutic agent**."

**C)** Teve grupo controle?

( ) Sim ( ) Não

**O)** O estudo apresenta valores de marcadores inflamatórios (citocinas e outros marcadores) antes e após as intervenções de creatina?

( ) Sim	( ) Não

**S)** O estudo é um ensaio clínico randomizado e controlado?

( ) Sim	( ) Não

**Artigo 327: [Evaluation of severity in acute pancreatitis].**

Ryu JK

**P)** Os participantes possuem mais de 18 anos de idade?

(X) Sim	( ) Não

"The study is an 'Evaluation of Severity in Acute Pancreatitis' that discusses various severity scoring systems for patients. For instance, the Glasgow Severity Scoring System includes an 'Age > 55 years' criterion, and the BISAP (Bedside Index for Severity in Acute Pancreatitis) scoring system includes 'age > 60' as a factor, clearly indicating that the study population is comprised of adult human patients."

**I)** A intervenção utilizada no estudo é creatina? Vale qualquer formato de administração

( ) Sim	(X) Não

"The study is a review focused on evaluating and comparing different severity scoring systems for acute pancreatitis. While 'Serum creatinine level' is mentioned as a diagnostic criterion for renal failure within the Atlanta Classification, and 'creatinine' is listed as a measured biochemical parameter in other contexts, **creatine itself is not an intervention or administered substance** in this study."

**C)** Teve grupo controle?

( ) Sim ( ) Não

**O)** O estudo apresenta valores de marcadores inflamatórios (citocinas e outros marcadores) antes e após as intervenções de creatina?

( ) Sim	( ) Não

**S)** O estudo é um ensaio clínico randomizado e controlado?

( ) Sim	( ) Não

**Artigo 328: Role of the poly(ADP-ribose)polymerase activity in vancomycin-induced renal injury.**

Dalaklioglu S, Tekcan M, Gungor NE, Celik-Ozenci C, Aksoy NH, Baykal A, Tasatargil A

**P)** Os participantes possuem mais de 18 anos de idade?

( ) Sim	(X) Não

"The study explicitly states that 'Male Wistar rats, weighing 300–350 g, were used in the present study', indicating that the participants are **animals** and not humans over 18 years of age."

**I)** A intervenção utilizada no estudo é creatina? Vale qualquer formato de administração

( ) Sim	( ) Não

**C)** Teve grupo controle?

( ) Sim ( ) Não

**O)** O estudo apresenta valores de marcadores inflamatórios (citocinas e outros marcadores) antes e após as intervenções de creatina?

( ) Sim	( ) Não

**S)** O estudo é um ensaio clínico randomizado e controlado?

( ) Sim	( ) Não

**Artigo 329: Rational drug correction of systemic inflammatory response syndrome in severe experimental heart failure.**

Sukoyan GV, Antelava NA

**P)** Os participantes possuem mais de 18 anos de idade?

( ) Sim	(X) Não

"The study explicitly states that 'The study was carried out on **120 Chinchilla rabbits (2.4-4.1 kg)**', indicating that the participants are **animals** and not humans over 18 years of age."

**I)** A intervenção utilizada no estudo é creatina? Vale qualquer formato de administração

( ) Sim	( ) Não

**C)** Teve grupo controle?

( ) Sim ( ) Não

**O)** O estudo apresenta valores de marcadores inflamatórios (citocinas e outros marcadores) antes e após as intervenções de creatina?

( ) Sim	( ) Não

**S)** O estudo é um ensaio clínico randomizado e controlado?

( ) Sim	( ) Não

**Artigo 330: Microalbuminuria and short-term prognosis in patients undergoing cardiac surgery.**

Mikkelsen MM, Andersen NH, Christensen TD, Hansen TK, Eiskjaer H, Mogensen CE, Hjortdal VE, Johnsen SP

**P)** Os participantes possuem mais de 18 anos de idade?

(X) Sim	( ) Não

"Inclusion criteria were (i) age **older than 18 years**"

**I)** A intervenção utilizada no estudo é creatina? Vale qualquer formato de administração

( ) Sim	(X) Não

"The study investigates the association of 'preoperative **microalbuminuria** (MA)' with 'adverse outcomes'. It assesses MA using the 'urine albumin-to-creatinine ratio' and measures 'serum creatinine'. **Creatine is mentioned only as a component of the 'urine albumin-to-creatinine ratio' and 'serum creatinine' measurements, which are diagnostic/prognostic parameters, not an administered intervention or therapeutic agent**."

**C)** Teve grupo controle?

( ) Sim ( ) Não

**O)** O estudo apresenta valores de marcadores inflamatórios (citocinas e outros marcadores) antes e após as intervenções de creatina?

( ) Sim	( ) Não

**S)** O estudo é um ensaio clínico randomizado e controlado?

( ) Sim	( ) Não

**Artigo 331: MR spectroscopy indicates diffuse multiple sclerosis activity during remission.**

Kirov II, Patil V, Babb JS, Rusinek H, Herbert J, Gonen O

**P)** Os participantes possuem mais de 18 anos de idade?

(X) Sim	( ) Não

"The study explicitly states the age ranges for participants: 'Their mean age was 33 (range **21 – 42**) years' for patients and 'mean age 30, range **20 – 44** years' for controls. These ranges clearly indicate that all participants were over 18 years of age."

**I)** A intervenção utilizada no estudo é creatina? Vale qualquer formato de administração

( ) Sim	(X) Não

"The study's methodology focuses on measuring 'Metabolite concentrations: N-acetylaspartate (NAA) for neuronal integrity; choline (Cho) for membrane turnover rate; **creatine (Cr)** and myo-inositol (mI) for glial status' using proton MR spectroscopic imaging. **Creatine is analyzed as a measured metabolite and a control metabolite, not as an administered intervention or therapeutic substance**."

**C)** Teve grupo controle?

( ) Sim ( ) Não

**O)** O estudo apresenta valores de marcadores inflamatórios (citocinas e outros marcadores) antes e após as intervenções de creatina?

( ) Sim	( ) Não

**S)** O estudo é um ensaio clínico randomizado e controlado?

( ) Sim	( ) Não

**Artigo 332: Foot muscle energy reserves in diabetic patients without and with clinical peripheral neuropathy.**

Dinh T, Doupis J, Lyons TE, Kuchibhotla S, Julliard W, Gnardellis C, Rosenblum BI, Wang X, Giurini JM, Greenman RL, Veves A

**P)** Os participantes possuem mais de 18 anos de idade?

(X) Sim	( ) Não

"The study involved 'healthy control subjects and non-neuropathic and neuropathic diabetic patients'. Specifically, 'Seven diabetic patients... (four males, **mean age 55 7 years**)' were included. This indicates that the participants were human adults, well over 18 years of age."

**I)** A intervenção utilizada no estudo é creatina? Vale qualquer formato de administração

( ) Sim	(X) Não

"The study states its method as measuring 'the **phosphocreatinine (PCr)** / inorganic phosphate (Pi) ratio' in muscle using MRI spectroscopy. **Creatine is measured as phosphocreatine (PCr), an endogenous metabolite indicating energy status, not administered as an intervention or therapeutic agent**."

**C)** Teve grupo controle?

( ) Sim ( ) Não

**O)** O estudo apresenta valores de marcadores inflamatórios (citocinas e outros marcadores) antes e após as intervenções de creatina?

( ) Sim	( ) Não

**S)** O estudo é um ensaio clínico randomizado e controlado?

( ) Sim	( ) Não

**Artigo 333: Negative effects of anemia on quality of life and its improvement by complete correction of anemia by administration of recombinant human erythropoietin in posttransplant patients.**

Kawada N, Moriyama T, Ichimaru N, Imamura R, Matsui I, Takabatake Y, Nagasawa Y, Isaka Y, Kojima Y, Kokado Y, Rakugi H, Imai E, Takahara S

**P)** Os participantes possuem mais de 18 anos de idade?

( ) Sim	(X) Não

"The provided excerpt from the article **does not explicitly state the age range or a minimum age of 18 years or older for the participants**. While the study involves 'posttransplant patients' and excludes patients with 'dementia', which are typically conditions and exclusions for adult populations, there is no direct sentence or phrase confirming that all participants were definitively over 18 years of age."

**I)** A intervenção utilizada no estudo é creatina? Vale qualquer formato de administração

( ) Sim	( ) Não

**C)** Teve grupo controle?

( ) Sim ( ) Não

**O)** O estudo apresenta valores de marcadores inflamatórios (citocinas e outros marcadores) antes e após as intervenções de creatina?

( ) Sim	( ) Não

**S)** O estudo é um ensaio clínico randomizado e controlado?

( ) Sim	( ) Não

**Artigo 334: Characterization of synovial tissue from arthritis patients: a proton magnetic resonance spectroscopic investigation.**

Hitchon CA, El-Gabalawy HS, Bezabeh T

**P)** Os participantes possuem mais de 18 anos de idade?

(X) Sim	( ) Não

"The study specifies the age range of the rheumatoid arthritis patients: 'Age at time of arthroplasty (mean and range) **61.1 years (33–80)**'. This range clearly indicates that all participants were over 18 years of age."

**I)** A intervenção utilizada no estudo é creatina? Vale qualquer formato de administração

( ) Sim	(X) Não

"The study investigates the biochemical composition of synovial tissue using proton MR spectroscopy and identifies **creatine (Cr)** as a measured metabolite. It states: 'Spectra from severely and mildly inXamed tissue diVered in peak intensity at regions... 3.0 ppm (representing creatine)...'. **Creatine is measured as an endogenous compound, not administered as an intervention or therapeutic agent.**"

**C)** Teve grupo controle?

( ) Sim ( ) Não

**O)** O estudo apresenta valores de marcadores inflamatórios (citocinas e outros marcadores) antes e após as intervenções de creatina?

( ) Sim	( ) Não

**S)** O estudo é um ensaio clínico randomizado e controlado?

( ) Sim	( ) Não

**Artigo 335: The effects of interferon beta-1a on proton MR spectroscopic imaging in patients with multiple sclerosis, a** controlled study**, preliminary results.**

Toprak MK, Cakir B, Ulu EM, Arat Z, Benli US, Can U, Ağildere M

**P)** Os participantes possuem mais de 18 anos de idade?

( ) Sim	( ) Não [*Excluir*]

**I)** A intervenção utilizada no estudo é creatina? Vale qualquer formato de administração

( ) Sim	(	) Não [*Excluir*]

**C)** Teve grupo controle?

(	) Sim ( ) Não [*Excluir*]

**O)** O estudo apresenta valores de marcadores inflamatórios (citocinas e outros marcadores) antes e após as intervenções de creatina?

( ) Sim	(	) Não [*Excluir*]

**S)** O estudo é um ensaio clínico randomizado e controlado?

(	) Sim	( ) Não

**Artigo 336: Distinct MRI pattern in lesional and perilesional area after traumatic brain injury in** rat**--11 months follow-up.**

Immonen RJ, Kharatishvili I, Niskanen JP, Gröhn H, Pitkänen A, Gröhn OH

**P)** Os participantes possuem mais de 18 anos de idade?

( ) Sim	(X) Não

"The study explicitly states that 'TBI was induced in **adult male Harlan Sprague Dawley rats** (total n=48, from which n=34 survived and were examined by MRI, 305–390 g; Harlan Netherlands B.V., Horst, the Netherlands)'. This indicates the participants were **rats**, not humans, and therefore the concept of "more than 18 years of age" in human terms does not apply."

**I)** A intervenção utilizada no estudo é creatina? Vale qualquer formato de administração

( ) Sim	( ) Não

**C)** Teve grupo controle?

( ) Sim ( ) Não

**O)** O estudo apresenta valores de marcadores inflamatórios (citocinas e outros marcadores) antes e após as intervenções de creatina?

( ) Sim	( ) Não

**S)** O estudo é um ensaio clínico randomizado e controlado?

( ) Sim	( ) Não

**Artigo 337: Pre-existing renal disease promotes sepsis-induced acute kidney injury and worsens outcome.**

Doi K, Leelahavanichkul A, Hu X, Sidransky KL, Zhou H, Qin Y, Eisner C, Schnermann J, Yuen PS, Star RA

**P)** Os participantes possuem mais de 18 anos de idade?

( ) Sim	(X) Não

"The study explicitly states its methodology involved animal subjects, specifically: 'We established a **mouse** two-stage model of pre-existing renal disease with subsequent sepsis'. It further confirms: 'In the present study, we developed a new two-stage **mouse** model that mimics sepsis in patients with pre-existing renal dysfunction'. Therefore, the participants are **mice**, not humans, and the concept of being over 18 years of age does not apply."

**I)** A intervenção utilizada no estudo é creatina? Vale qualquer formato de administração

( ) Sim	( ) Não

**C)** Teve grupo controle?

( ) Sim ( ) Não

**O)** O estudo apresenta valores de marcadores inflamatórios (citocinas e outros marcadores) antes e após as intervenções de creatina?

( ) Sim	( ) Não

**S)** O estudo é um ensaio clínico randomizado e controlado?

( ) Sim	( ) Não

**Artigo 338: Pro- and anti-inflammatory cytokines release in** mice **injected with Crotalus durissus terrificus venom.**

Hernández Cruz A, Garcia-Jimenez S, Zucatelli Mendonça R, Petricevich VL

**P)** Os participantes possuem mais de 18 anos de idade?

( ) Sim	(X) Não

"The study explicitly states that the experiments were conducted using 'BALB/c female mice'. Therefore, the participants are **mice**, not humans, and the concept of possessing more than 18 years of age does not apply."

**I)** A intervenção utilizada no estudo é creatina? Vale qualquer formato de administração

( ) Sim	( ) Não

**C)** Teve grupo controle?

( ) Sim ( ) Não

**O)** O estudo apresenta valores de marcadores inflamatórios (citocinas e outros marcadores) antes e após as intervenções de creatina?

( ) Sim	( ) Não

**S)** O estudo é um ensaio clínico randomizado e controlado?

( ) Sim	( ) Não

**Artigo 339: Pretransplant donor-specific and non-specific immune parameters associated with early acute rejection.**

Reinsmoen NL, Cornett KM, Kloehn R, Burnette AD, McHugh L, Flewellen BK, Matas A, Savik K

**P)** Os participantes possuem mais de 18 anos de idade?

(X) Sim	( ) Não

"The study enrolled kidney transplant recipients, and **Table 1** presents the **'Avg. transplant age' for each arm as '48.7', '48.8', and '50.70' years**. This clearly indicates that all participants were adults and well over 18 years of age."

**I)** A intervenção utilizada no estudo é creatina? Vale qualquer formato de administração

( ) Sim	(X) Não

"The study evaluates immune parameters in kidney recipients undergoing different **immunosuppression protocols, including thymoglobulin, prednisone, cyclosporine (CSA), mycophenolate mofetil (MMF), tacrolimus (TAC), and sirolimus (SRL)**. **Creatine is not mentioned as an administered intervention** in any form; rather, creatinine is measured as a marker of renal function and iATP (intracellular adenosine triphosphate) is measured as a global immune response status indicator."

**C)** Teve grupo controle?

( ) Sim ( ) Não

**O)** O estudo apresenta valores de marcadores inflamatórios (citocinas e outros marcadores) antes e após as intervenções de creatina?

( ) Sim	( ) Não

**S)** O estudo é um ensaio clínico randomizado e controlado?

( ) Sim	( ) Não

**Artigo 340: Microalbuminuria, endothelial dysfunction and inflammation in primary hypertension.**

Perticone F, Maio R, Tripepi G, Sciacqua A, Mallamaci F, Zoccali C

**P)** Os participantes possuem mais de 18 anos de idade?

(X) Sim	( ) Não

"The study recruited 'One hundred and ten never-treated patients with uncomplicated essential hypertension' from a 'hypertension clinic'. Given that essential hypertension is a condition predominantly affecting adults, and the participants are referred to as 'patients' in a clinical setting, it is implied that they are **well over 18 years of age**."

**I)** A intervenção utilizada no estudo é creatina? Vale qualquer formato de administração

( ) Sim	(X) Não

"The study investigates the relationship between microalbuminuria, endothelial function, and inflammation (specifically **high-sensitivity C-reactive protein [CRP]**) in patients with hypertension. It mentions measuring 'serum creatinine' as an indicator of renal function, but **creatine (in any form) is not mentioned or used as an intervention**."

**C)** Teve grupo controle?

( ) Sim ( ) Não

**O)** O estudo apresenta valores de marcadores inflamatórios (citocinas e outros marcadores) antes e após as intervenções de creatina?

( ) Sim	( ) Não

**S)** O estudo é um ensaio clínico randomizado e controlado?

( ) Sim	( ) Não

**Artigo 341: Lymphocytes are dispensable for glomerulonephritis but required for renal interstitial fibrosis in matrix defect-induced Alport renal disease.**

Lebleu VS, Sugimoto H, Miller CA, Gattone VH 2nd, Kalluri R

**P)** Os participantes possuem mais de 18 anos de idade?

( ) Sim	(X) Não

"The study explicitly states its subjects are **mice**, referring to 'a **mouse** model for progressive glomerulonephritis' and later, in the methods section, 'MATERIALS AND METHODS **Mice**' and 'All **animal** studies were reviewed and approved by the Animal Care and Use Committee of the Beth Israel Deaconess Medical Center'. Therefore, the participants are not human and the concept of being over 18 years of age is not applicable."

**I)** A intervenção utilizada no estudo é creatina? Vale qualquer formato de administração

( ) Sim	( ) Não

**C)** Teve grupo controle?

( ) Sim ( ) Não

**O)** O estudo apresenta valores de marcadores inflamatórios (citocinas e outros marcadores) antes e após as intervenções de creatina?

( ) Sim	( ) Não

**S)** O estudo é um ensaio clínico randomizado e controlado?

( ) Sim	( ) Não

**Artigo 342: [Effect of ulinastatin in promotion of graft recovery in early stage after kidney transplantation: a clinical study].**

Qiu J, Wang CX, Chen LZ, Fei JG, Bi J

**P)** Os participantes possuem mais de 18 anos de idade?

( ) Sim	(X) Não

"The provided excerpts from the study (Article 342) state that 'Thirty patients underwent general cadaver kidney transplantation', but **do not explicitly mention the age of these participants or state an inclusion criterion that they must be over 18 years of age**. Therefore, based strictly on the available text, this information cannot be confirmed."

**I)** A intervenção utilizada no estudo é creatina? Vale qualquer formato de administração

( ) Sim	( ) Não

**C)** Teve grupo controle?

( ) Sim ( ) Não

**O)** O estudo apresenta valores de marcadores inflamatórios (citocinas e outros marcadores) antes e após as intervenções de creatina?

( ) Sim	( ) Não

**S)** O estudo é um ensaio clínico randomizado e controlado?

( ) Sim	( ) Não

**Artigo 343: Case 5/2007: a 71 year-old diabetic and hypertensive female with left ventricular hypertrophy and epigastric pain.**

Feitosa LF, Marques AC, Benvenuti LA

**P)** Os participantes possuem mais de 18 anos de idade?

(X) Sim	( ) Não

"The study describes a **'71-year-old female patient'**."

**I)** A intervenção utilizada no estudo é creatina? Vale qualquer formato de administração

( ) Sim	(X) Não

"This is a **clinical case report of a patient**, focusing on her medical history, symptoms, and eventual septic shock. The article **does not describe any intervention using creatine in any format**."

**C)** Teve grupo controle?

( ) Sim ( ) Não

**O)** O estudo apresenta valores de marcadores inflamatórios (citocinas e outros marcadores) antes e após as intervenções de creatina?

( ) Sim	( ) Não

**S)** O estudo é um ensaio clínico randomizado e controlado?

( ) Sim	( ) Não

**Artigo 344: Magnetic resonance spectroscopy features of normal-appearing white matter in patients with acute brucellosis.**

Kayabas U, Alkan A, Firat AK, Karakas HM, Bayindir Y, Yetkin F

**P)** Os participantes possuem mais de 18 anos de idade?

(X) Sim	( ) Não

"The study explicitly states the age range for patients as 'between **18 and 50** (33.6 ± 2.5) years' and for healthy volunteers as 'between **26 and 43** (34.1 ± 1.5) years'."

**I)** A intervenção utilizada no estudo é creatina? Vale qualquer formato de administração

( ) Sim	(X) Não

"The study used Magnetic Resonance Spectroscopy (MRS) to measure levels of metabolites, including 'creatine (Cr)', to calculate ratios such as 'NAA/creatine (Cr) and choline (Cho)/Cr ratios'. Creatine was a measured compound, not an intervention administered in any format."

**C)** Teve grupo controle?

( ) Sim ( ) Não

**O)** O estudo apresenta valores de marcadores inflamatórios (citocinas e outros marcadores) antes e após as intervenções de creatina?

( ) Sim	( ) Não

**S)** O estudo é um ensaio clínico randomizado e controlado?

( ) Sim	( ) Não

**Artigo 345: Creatine monohydrate and conjugated linoleic acid improve strength and body composition following resistance exercise in older adults.**

Tarnopolsky M, Zimmer A, Paikin J, Safdar A, Aboud A, Pearce E, Roy B, Doherty T

**P)** Os participantes possuem mais de 18 anos de idade?

(X) Sim	( ) Não

"**older adults (.65 y)**"

"Thirty-nine community dwelling, **older adult (65–85 y)**, men (n = 19) and women (n = 20) were recruited".

**I)** A intervenção utilizada no estudo é creatina? Vale qualquer formato de administração

(X) Sim	(	) Não

"**creatine monohydrate (CrM)** and conjugated linoleic acid (CLA) could enhance strength gains and improve body composition".

"**CrM and CLA (CrM+CLA) (5 g CrM** (NeotineH)+2 g dextrose/d)".

O formato de administração foi oral, diariamente.

**C)** Teve grupo controle?

(X) Sim ( ) Não

"**randomized, double blind, allocation**"

"randomized to either a combination of CrM and CLA (CrM+CLA)... or **placebo (PL)** (7 g dextrose/d+6 g of safflower oil/d)"

**O)** O estudo apresenta valores de marcadores inflamatórios (citocinas e outros marcadores) antes e após as intervenções de creatina?

(X) Sim	(	) Não

"blood tests (lipids, liver function, CK, glucose, **systemic inflammation markers (IL-6, C-reactive protein**))".

"evaluate whether resistance exercise training with or without a CrM+CLA supplementation would favorably alter **markers of inflammation**". A Tabela 5 apresenta "Blood Analysis" que inclui os valores de "**CRP (mg/L)**" e "**IL-6 (pg/mL)**" tanto "Pre" quanto "Post" intervenção para ambos os grupos. Embora os resultados tenham mostrado "no effect from training or supplementation" para esses marcadores, eles foram medidos.

**S)** O estudo é um ensaio clínico randomizado e controlado?

(X) Sim	( ) Não

O design do estudo é explicitamente descrito como um "**randomized, double blind, allocation**". Além disso, o estudo foi "**registered in ClinicalTrials.gov under # NCT00473902**", o que é uma característica de um ensaio clínico. A alocação foi feita de forma "**randomized**" e houve um grupo de "**placebo**"

**Artigo 346: Urinary matrix metalloproteinases reflect renal damage in anti-neutrophil cytoplasm autoantibody-associated vasculitis.**

Sanders JS, Huitema MG, Hanemaaijer R, van Goor H, Kallenberg CG, Stegeman CA

**P)** Os participantes possuem mais de 18 anos de idade?

(X) Sim	( ) Não

"The study specifies that the **median age in the patient group was 67 years, with a range of 23–86 years**. Additionally, the **healthy controls had a median age of 51 years, with a range of 44–67 years**. All participants were therefore over 18 years of age."

**I)** A intervenção utilizada no estudo é creatina? Vale qualquer formato de administração

( ) Sim	(X) Não

"The study focused on measuring 'Urinary matrix metalloproteinases (MMP-2, -9) and tissue inhibitor of MMP-1 (TIMP-1)' and their correlation with renal damage in patients with vasculitis. While **serum creatinine** levels were recorded as a clinical characteristic to assess renal function, **creatine was not an intervention administered in any format**."

**C)** Teve grupo controle?

( ) Sim ( ) Não

**O)** O estudo apresenta valores de marcadores inflamatórios (citocinas e outros marcadores) antes e após as intervenções de creatina?

( ) Sim	( ) Não

**S)** O estudo é um ensaio clínico randomizado e controlado?

( ) Sim	( ) Não

**Artigo 347: Mitochondrial function in sepsis: respiratory versus leg muscle.**

Fredriksson K, Rooyackers O

**P)** Os participantes possuem mais de 18 anos de idade?

(X) Sim	( ) Não

"The study included 'Ten critically ill patients' and '10 metabolically healthy age- and sex-matched patients undergoing elective surgery were used as controls'. It specifies that the 'Control subjects... had a **median age of 67 (range 45–87) yr**'. Therefore, all participants were over 18 years of age."

**I)** A intervenção utilizada no estudo é creatina? Vale qualquer formato de administração

( ) Sim	(X) Não

"The study investigates 'mitochondrial and energy metabolism' in muscle tissue by measuring levels of metabolites such as 'ATP, **creatine phosphate**, and lactate'. **Creatine or creatine phosphate was a measured compound to assess energy status, not an administered intervention**."

**C)** Teve grupo controle?

( ) Sim ( ) Não

**O)** O estudo apresenta valores de marcadores inflamatórios (citocinas e outros marcadores) antes e após as intervenções de creatina?

( ) Sim	( ) Não

**S)** O estudo é um ensaio clínico randomizado e controlado?

( ) Sim	( ) Não

**Artigo 348: The effect of ultrapure dialysate on improving renal anemia.**

Go I, Takemoto Y, Tsuchida K, Sugimura K, Nakatani T

**P)** Os participantes possuem mais de 18 anos de idade?

(X) Sim	( ) Não

"The study states that 'The **mean age of patients was 57.5 years old (59.1±11.7 years old)**'. This indicates all participants were well over 18 years of age."

**I)** A intervenção utilizada no estudo é creatina? Vale qualquer formato de administração

( ) Sim	(X) Não

"The primary intervention investigated in this study was the use of '**ultrapure dialysate**' to improve 'renal anemia'. While 'erythrocyte creatine' is mentioned as an 'index of the erythrocyte life span and erythropoiesis', **creatine was a measured compound to assess biological markers, not an administered intervention** in any form."

**C)** Teve grupo controle?

( ) Sim ( ) Não

**O)** O estudo apresenta valores de marcadores inflamatórios (citocinas e outros marcadores) antes e após as intervenções de creatina?

( ) Sim	( ) Não

**S)** O estudo é um ensaio clínico randomizado e controlado?

( ) Sim	( ) Não

**Artigo 349: TLR4 activation mediates kidney ischemia/reperfusion injury.**

Wu H, Chen G, Wyburn KR, Yin J, Bertolino P, Eris JM, Alexander SI, Sharland AF, Chadban SJ

**P)** Os participantes possuem mais de 18 anos de idade?

( ) Sim	(X) Não

"The study was conducted using **mouse models**, specifically mentioning 'TLR4–/– mice', 'MyD88–/– mice', and 'WT controls'. Therefore, the participants are not human and are not over 18 years of age."

**I)** A intervenção utilizada no estudo é creatina? Vale qualquer formato de administração

( ) Sim	( ) Não

**C)** Teve grupo controle?

( ) Sim ( ) Não

**O)** O estudo apresenta valores de marcadores inflamatórios (citocinas e outros marcadores) antes e após as intervenções de creatina?

( ) Sim	( ) Não

**S)** O estudo é um ensaio clínico randomizado e controlado?

( ) Sim	( ) Não

**Artigo 350: Proinflammatory and proapoptotic effects of methylglyoxal on neutrophils from patients with type 2 diabetes mellitus.**

Wang H, Meng QH, Gordon JR, Khandwala H, Wu L

**P)** Os participantes possuem mais de 18 anos de idade?

(X) Sim	( ) Não

"The study included 'Twenty non-diabetic subjects and fifty-five T2DM patients'. The age range for these participants was specified as **'29–82' years for non-diabetic subjects and '34–80' years for T2DM patients**. Therefore, all participants were over 18 years of age".

**I)** A intervenção utilizada no estudo é creatina? Vale qualquer formato de administração

( ) Sim	(X) Não

"The study's primary aim was to 'determine the effect of **methylglyoxal (MG)** on cytokine production by, and apoptosis of, neutrophils'. **Creatine was not an intervention administered in any format**; rather, methylglyoxal was the substance used to treat the neutrophils in vitro".

**C)** Teve grupo controle?

( ) Sim ( ) Não

**O)** O estudo apresenta valores de marcadores inflamatórios (citocinas e outros marcadores) antes e após as intervenções de creatina?

( ) Sim	( ) Não

**S)** O estudo é um ensaio clínico randomizado e controlado?

( ) Sim	( ) Não

**Artigo 351: Melatonin protects from ischemia/reperfusion-induced renal injury in** rats**: this effect is not mediated by proinflammatory cytokines.**

Kurcer Z, Oguz E, Ozbilge H, Baba F, Aksoy N, Celik H, Cakir H, Gezen MR

**P)** Os participantes possuem mais de 18 anos de idade?

( ) Sim	(X) Não

"The study explicitly states that 'Male Wistar albino rats were unilaterally nephrectomized'. Therefore, the participants are **animals (rats)** and are not over 18 years of age."

**I)** A intervenção utilizada no estudo é creatina? Vale qualquer formato de administração

( ) Sim	( ) Não

**C)** Teve grupo controle?

( ) Sim ( ) Não

**O)** O estudo apresenta valores de marcadores inflamatórios (citocinas e outros marcadores) antes e após as intervenções de creatina?

( ) Sim	( ) Não

**S)** O estudo é um ensaio clínico randomizado e controlado?

( ) Sim	( ) Não

**Artigo 352: Detection of human immunodeficiency virus induced inflammation and oxidative stress in lenticular nuclei with magnetic resonance spectroscopy despite antiretroviral therapy.**

Roc AC, Ances BM, Chawla S, Korczykowski M, Wolf RL, Kolson DL, Detre JA, Poptani H

**P)** Os participantes possuem mais de 18 anos de idade?

(X) Sim	( ) Não

"The study included 'Ten seronegative controls and 45 HIV-positive patients'. The demographic table for these subjects shows **mean ages ranging from 41 to 46 years** across all groups. This indicates that all participants were adults and therefore over 18 years of age."

**I)** A intervenção utilizada no estudo é creatina? Vale qualquer formato de administração

( ) Sim	(X) Não

"The study states that 'creatine (Cr)' is a '**marker of high-energy metabolism**' and its levels, or ratios involving it (e.g., N-acetyl aspartate:Cr, choline:Cr, Lac:Cr), were **measured** using magnetic resonance spectroscopy. The primary intervention discussed for the HIV-positive patients was **highly active antiretroviral therapy (HAART)**, which was already being adhered to by many participants at the time of the scan. Creatine was a measured metabolite, not an administered intervention."

**C)** Teve grupo controle?

( ) Sim ( ) Não

**O)** O estudo apresenta valores de marcadores inflamatórios (citocinas e outros marcadores) antes e após as intervenções de creatina?

( ) Sim	( ) Não

**S)** O estudo é um ensaio clínico randomizado e controlado?

( ) Sim	( ) Não

**Artigo 353: Caspase inhibition reduces cardiac myocyte dyshomeostasis and improves cardiac contractile function after major burn injury.**

Carlson DL, Maass DL, White J, Sikes P, Horton JW

**P)** Os participantes possuem mais de 18 anos de idade?

( ) Sim	(X) Não

"In this study, **Sprague-Dawley rats** were given either a third-degree burn over 40% total body surface area plus conventional fluid resuscitation or sham burn injury. Therefore, the participants are **animals (rats)** and are not over 18 years of age."

**I)** A intervenção utilizada no estudo é creatina? Vale qualquer formato de administração

( ) Sim	( ) Não

**C)** Teve grupo controle?

( ) Sim ( ) Não

**O)** O estudo apresenta valores de marcadores inflamatórios (citocinas e outros marcadores) antes e após as intervenções de creatina?

( ) Sim	( ) Não

**S)** O estudo é um ensaio clínico randomizado e controlado?

( ) Sim	( ) Não

**Artigo 354:** **Effects of losartan on urinary secretion of extracellular matrix and their modulators in type 2 diabetes mellitus patients with microalbuminuria**

Woo V, Ni LS, Hak D, Berard L, Zhu F, Khan S, Ma GM, Penner B, Shen GX

**P)** Os participantes possuem mais de 18 anos de idade?

( ) Sim	( ) Não [*Excluir*]

**I)** A intervenção utilizada no estudo é creatina? Vale qualquer formato de administração

( ) Sim	(	) Não [*Excluir*]

**C)** Teve grupo controle?

(	) Sim ( ) Não [*Excluir*]

**O)** O estudo apresenta valores de marcadores inflamatórios (citocinas e outros marcadores) antes e após as intervenções de creatina?

( ) Sim	(	) Não [*Excluir*]

**S)** O estudo é um ensaio clínico randomizado e controlado?

(	) Sim	( ) Não

**Artigo 355: Efficacy and renal toxicity of one daily dose of amikacin versus conventional dosage regime.**

Guadalupe Vásquez-Mendoza M, Vargas-Origel A, Del Carmen Ramos-Jiménez A, Aguilar-Orozco G, Romero-Gutiérrez G

**P)** Os participantes possuem mais de 18 anos de idade?

( ) Sim	( ) Não [*Excluir*]

**I)** A intervenção utilizada no estudo é creatina? Vale qualquer formato de administração

( ) Sim	(	) Não [*Excluir*]

**C)** Teve grupo controle?

(	) Sim ( ) Não [*Excluir*]

**O)** O estudo apresenta valores de marcadores inflamatórios (citocinas e outros marcadores) antes e após as intervenções de creatina?

( ) Sim	(	) Não [*Excluir*]

**S)** O estudo é um ensaio clínico randomizado e controlado?

(	) Sim	( ) Não

**Artigo 356: Effects of KIOM-79 on hyperglycemia and diabetic nephropathy in type 2 diabetic Goto-Kakizaki** rats**.**

Kim CS, Sohn EJ, Kim YS, Jung DH, Jang DS, Lee YM, Kim DH, Kim JS

**P)** Os participantes possuem mais de 18 anos de idade?

( ) Sim	(X) Não

"The study explicitly states that 'Male GK rats and control, Wistar rats age-matched were used'. Therefore, the participants are **animals (rats)** and are not over 18 years of age."

**I)** A intervenção utilizada no estudo é creatina? Vale qualquer formato de administração

( ) Sim	( ) Não

**C)** Teve grupo controle?

( ) Sim ( ) Não

**O)** O estudo apresenta valores de marcadores inflamatórios (citocinas e outros marcadores) antes e após as intervenções de creatina?

( ) Sim	( ) Não

**S)** O estudo é um ensaio clínico randomizado e controlado?

( ) Sim	( ) Não

**Artigo 357: Influence of bradykinin B1 and B2 receptors in the immune response triggered by renal ischemia-reperfusion injury.**

Wang PH, Cenedeze MA, Pesquero JB, Pacheco-Silva A, Câmara NO

**P)** Os participantes possuem mais de 18 anos de idade?

( ) Sim	(X) Não

"The study explicitly states that 'B1R (B1−/−), B2R (B2−/−) knockout **animals** and its control (**wild-type mice**, B1B2+/+) were subjected to renal bilateral ischemia'. Therefore, the participants are **animals (mice)** and are not over 18 years of age."

**I)** A intervenção utilizada no estudo é creatina? Vale qualquer formato de administração

( ) Sim	( ) Não

**C)** Teve grupo controle?

( ) Sim ( ) Não

**O)** O estudo apresenta valores de marcadores inflamatórios (citocinas e outros marcadores) antes e após as intervenções de creatina?

( ) Sim	( ) Não

**S)** O estudo é um ensaio clínico randomizado e controlado?

( ) Sim	( ) Não

**Artigo 358: Effects of knee injection on skeletal muscle metabolism and contractile force in** rats**.**

Galbán CJ, Ling SM, Galbán CJ, Taub DD, Gurkan I, Fishbein KW, Spencer RG

**P)** Os participantes possuem mais de 18 anos de idade?

( ) Sim	(X) Não

"The study clearly states in its methods section that 'Adult rats were separated into four groups' and specifies them as 'Three-to 4-month-old female Fisher 344 rats'. Therefore, the participants are **animals (rats)** and are not over 18 years of age."

**I)** A intervenção utilizada no estudo é creatina? Vale qualquer formato de administração

( ) Sim	( ) Não

**C)** Teve grupo controle?

( ) Sim ( ) Não

**O)** O estudo apresenta valores de marcadores inflamatórios (citocinas e outros marcadores) antes e após as intervenções de creatina?

( ) Sim	( ) Não

**S)** O estudo é um ensaio clínico randomizado e controlado?

( ) Sim	( ) Não

**Artigo 359: Role of enteral nutrition supplemented with ebselen and EHEC in pancreatitis-associated multiple organ dysfunction in** rats**.**

Zhao H, Lu HG, Shi YB, Zhao LM, Bai C, Wang X

**P)** Os participantes possuem mais de 18 anos de idade?

( ) Sim	(X) Não

"The study explicitly states in its 'Materials and methods' section that 'Adult male Sprague-Dawley rats, weighing about 200 g, were fed standard rat chow...'. Therefore, the participants are **animals (rats)** and are not over 18 years of age".

**I)** A intervenção utilizada no estudo é creatina? Vale qualquer formato de administração

( ) Sim	( ) Não

**C)** Teve grupo controle?

( ) Sim ( ) Não

**O)** O estudo apresenta valores de marcadores inflamatórios (citocinas e outros marcadores) antes e após as intervenções de creatina?

( ) Sim	( ) Não

**S)** O estudo é um ensaio clínico randomizado e controlado?

( ) Sim	( ) Não

**Artigo 360: Practical approach to the diagnosis and treatment of anemia associated with CKD in elderly.**

Agarwal AK

**P)** Os participantes possuem mais de 18 anos de idade?

(X) Sim	( ) Não

"The title of the article is 'Practical Approach to the Diagnosis and Treatment of Anemia Associated With CKD in **Elderly**'. Furthermore, the text mentions a subset analysis involving patients where '717 were **65 years of age or older**'. This clearly indicates that the population discussed includes participants well over 18 years of age."

**I)** A intervenção utilizada no estudo é creatina? Vale qualquer formato de administração

( ) Sim	(X) Não

"The article focuses on the diagnosis and treatment of **anemia** associated with chronic kidney disease (CKD) using **erythropoiesis-stimulating proteins (ESPs)**, specifically mentioning 'epoetin alfa and darbepoetin alfa'. Creatine is not mentioned as an intervention."

**C)** Teve grupo controle?

( ) Sim ( ) Não

**O)** O estudo apresenta valores de marcadores inflamatórios (citocinas e outros marcadores) antes e após as intervenções de creatina?

( ) Sim	( ) Não

**S)** O estudo é um ensaio clínico randomizado e controlado?

( ) Sim	( ) Não

**Artigo 361: Noninvasive evaluation of liver repopulation by transplanted hepatocytes using 31P MRS imaging in** mice**.**

Landis CS, Yamanouchi K, Zhou H, Mohan S, Roy-Chowdhury N, Shafritz DA, Koretsky A, Roy-Chowdhury J, Hetherington HP, Guha C

**P)** Os participantes possuem mais de 18 anos de idade?

( ) Sim	(X) Não

"The study explicitly mentions the use of 'transgenic mice' and 'wild-type BDF mice' in the experiments. Therefore, the participants are **animals (mice)** and are not over 18 years of age."

**I)** A intervenção utilizada no estudo é creatina? Vale qualquer formato de administração

( ) Sim	( ) Não

**C)** Teve grupo controle?

( ) Sim ( ) Não

**O)** O estudo apresenta valores de marcadores inflamatórios (citocinas e outros marcadores) antes e após as intervenções de creatina?

( ) Sim	( ) Não

**S)** O estudo é um ensaio clínico randomizado e controlado?

( ) Sim	( ) Não

**Artigo 362: Skeletal muscles in chronic obstructive pulmonary disease: deconditioning, or myopathy?**

Wagner PD

**P)** Os participantes possuem mais de 18 anos de idade?

(X) Sim	( ) Não

"The article is an 'INVITED REVIEW' on 'Skeletal muscles in chronic obstructive pulmonary disease'. Chronic obstructive pulmonary disease (COPD) is a condition that primarily affects adults, and the text refers to 'COPD patients' and 'normal subjects' in the context of this adult-onset disease. It also mentions that the disease 'likely has been present for well over 10 years in most cases', implying an adult population."

**I)** A intervenção utilizada no estudo é creatina? Vale qualquer formato de administração

( ) Sim	(X) Não

"The article is a review that discusses various aspects of muscle metabolism and function in COPD patients, including 'greater phosphocreatine breakdown and slower phosphocreatine restoration after exercise'. **Phosphocreatine** is discussed as a metabolic marker within the muscle, not as an administered intervention. The interventions mentioned relate to 'muscle training'."

**C)** Teve grupo controle?

( ) Sim ( ) Não

**O)** O estudo apresenta valores de marcadores inflamatórios (citocinas e outros marcadores) antes e após as intervenções de creatina?

( ) Sim	( ) Não

**S)** O estudo é um ensaio clínico randomizado e controlado?

( ) Sim	( ) Não

**Artigo 363: +H-proton-magnetic resonance spectroscopic findings in a patient with acute hemicerebellitis presenting without localized signs: a** case report**.**

Melek E, Ozyer U, Erol I, Alehan F, Muhteşem Ağildere A

**P)** Os participantes possuem mais de 18 anos de idade?

( ) Sim	(X) Não

"The article is a case report about a patient with acute hemicerebellitis. Table 1, titled 'Features of hemicerebellitis: review of the literature', lists previous cases, with the first entry being '1 Iester et al.10 **5 yr/F**'. This indicates that the study includes at least one participant who is **5 years old**, which is not over 18 years of age."

**I)** A intervenção utilizada no estudo é creatina? Vale qualquer formato de administração

( ) Sim	( ) Não

**C)** Teve grupo controle?

( ) Sim ( ) Não

**O)** O estudo apresenta valores de marcadores inflamatórios (citocinas e outros marcadores) antes e após as intervenções de creatina?

( ) Sim	( ) Não

**S)** O estudo é um ensaio clínico randomizado e controlado?

( ) Sim	( ) Não

**Artigo 364: Clinical and imaging study of human immunodeficiency virus-1-infected youth receiving highly active antiretroviral therapy: pilot study using magnetic resonance spectroscopy.**

Gabis L, Belman A, Huang W, Milazzo M, Nachman S

**P)** Os participantes possuem mais de 18 anos de idade?

( ) Sim	( ) Não [*Excluir*]

**I)** A intervenção utilizada no estudo é creatina? Vale qualquer formato de administração

( ) Sim	(	) Não [*Excluir*]

**C)** Teve grupo controle?

(	) Sim ( ) Não [*Excluir*]

**O)** O estudo apresenta valores de marcadores inflamatórios (citocinas e outros marcadores) antes e após as intervenções de creatina?

( ) Sim	(	) Não [*Excluir*]

**S)** O estudo é um ensaio clínico randomizado e controlado?

(	) Sim	( ) Não

**Artigo 365: Derangements in mitochondrial metabolism in intercostal and leg muscle of critically ill patients with sepsis-induced multiple organ failure.**

Fredriksson K, Hammarqvist F, Strigård K, Hultenby K, Ljungqvist O, Wernerman J, Rooyackers O

**P)** Os participantes possuem mais de 18 anos de idade?

(X) Sim	( ) Não

"Ten critically ill patients suffering from sepsis-induced multiple organ failure and requiring mechanical ventilation were included in the study. A group (n = 10) of metabolically healthy age- and sex-matched patients undergoing elective surgery were used as controls. **Three women and seven men were included as controls with a median age of 67 (range 45–87) yr**."

**I)** A intervenção utilizada no estudo é creatina? Vale qualquer formato de administração

( ) Sim	(X) Não

"The study focused on measuring 'concentrations of ATP, **creatine phosphate**, and lactate' and 'Activities of citrate synthase and mitochondrial respiratory chain complexes I and IV' in muscle biopsies of patients and controls to assess mitochondrial and energy metabolism. Creatine was a **metabolite measured**, not an intervention administered."

**C)** Teve grupo controle?

( ) Sim ( ) Não

**O)** O estudo apresenta valores de marcadores inflamatórios (citocinas e outros marcadores) antes e após as intervenções de creatina?

( ) Sim	( ) Não

**S)** O estudo é um ensaio clínico randomizado e controlado?

( ) Sim	( ) Não

**Artigo 366: Association of high pretransplant sIL-6R plasma levels with acute tubular necrosis in kidney graft recipients.**

Sadeghi M, Daniel V, Naujokat C, Mehrabi A, Opelz G

**P)** Os participantes possuem mais de 18 anos de idade?

(X) Sim	( ) Não

"The study focuses on 'renal transplant recipients'. While a specific age range is not explicitly stated in the provided excerpts for this article, studies on kidney transplantation and critically ill patients often involve adults. For instance, source (Article 310) specifies 'Inclusion criteria were age ≥ 18 years old' for CKD patients, and source (Article 369) for critically ill septic patients explicitly mentions 'Exclusion criteria were: age < 18 years'. Given the nature of kidney graft recipients, it is highly implied that the participants are adults, thus **over 18 years of age**."

**I)** A intervenção utilizada no estudo é creatina? Vale qualquer formato de administração

( ) Sim	(X) Não

"The study investigated the association of pretransplant immune parameters and cytokine levels, such as 'soluble interleukin (sIL)-1RA, interleukin (IL)-2, sIL-2R, IL-3, IL-4, IL-6, sIL-6R, IL-10, tumor necrosis factor (TNF)-α, transforming growth factor (TGF)-β2, interferon (IFN)-γ, and neopterin plasma levels'. These were **measured** biomarkers and not administered as an intervention. The study context involves 'immunosuppression protocols' and 'treatment interventions' related to kidney transplantation, with **no mention of creatine** in any form."

**C)** Teve grupo controle?

( ) Sim ( ) Não

**O)** O estudo apresenta valores de marcadores inflamatórios (citocinas e outros marcadores) antes e após as intervenções de creatina?

( ) Sim	( ) Não

**S)** O estudo é um ensaio clínico randomizado e controlado?

( ) Sim	( ) Não

**Artigo 367: T cell cytokine imbalance towards production of IFN-gamma and IL-10 in NZB/W F1 lupus-prone** mice **is associated with autoantibody levels and nephritis.**

Enghard P, Langnickel D, Riemekasten G

**P)** Os participantes possuem mais de 18 anos de idade?

( ) Sim	(X) Não

"The study explicitly states that the participants were 'Lupus-prone female NZB/W F1 (NZB6NZW F1) and female CWF1 (BALB/c6NZW F1) mice'. It also mentions their age range as 'NZB/W F1 mice at different ages spanning **5 to 36 weeks** were analysed', indicating that the participants are animals and not humans over 18 years of age."

**I)** A intervenção utilizada no estudo é creatina? Vale qualquer formato de administração

( ) Sim	( ) Não

**C)** Teve grupo controle?

( ) Sim ( ) Não

**O)** O estudo apresenta valores de marcadores inflamatórios (citocinas e outros marcadores) antes e após as intervenções de creatina?

( ) Sim	( ) Não

**S)** O estudo é um ensaio clínico randomizado e controlado?

( ) Sim	( ) Não

**Artigo 368: Augmentation of intrarenal angiotensin II levels in uninephrectomized aldosterone/salt-treated hypertensive** rats**; renoprotective effects of an ultrahigh dose of olmesartan.**

Fan YY, Baba R, Nagai Y, Miyatake A, Hosomi N, Kimura S, Sun GP, Kohno M, Fujita M, Abe Y, Nishiyama A

**P)** Os participantes possuem mais de 18 anos de idade?

( ) Sim	(X) Não

"The study explicitly states that the participants were 'Male Sprague-Dawley rats... weighing 162 to 184 g at the beginning of the experiments'. This indicates that the participants are **animals (rats)** and not humans over 18 years of age."

**I)** A intervenção utilizada no estudo é creatina? Vale qualquer formato de administração

( ) Sim	( ) Não

**C)** Teve grupo controle?

( ) Sim ( ) Não

**O)** O estudo apresenta valores de marcadores inflamatórios (citocinas e outros marcadores) antes e após as intervenções de creatina?

( ) Sim	( ) Não

**S)** O estudo é um ensaio clínico randomizado e controlado?

( ) Sim	( ) Não

**Artigo 369: Nephrotoxicity due to combination antibiotic therapy with vancomycin and aminoglycosides in septic critically ill patients.**

Malacarne P, Bergamasco S, Donadio C

**P)** Os participantes possuem mais de 18 anos de idade?

(X) Sim	( ) Não

"Exclusion criteria were: **age < 18 years**". Additionally, the study included "all critically ill septic patients aged **more than 18 years** with normal renal function".

**I)** A intervenção utilizada no estudo é creatina? Vale qualquer formato de administração

( ) Sim	(X) Não

"The aim of this prospective observational study was to evaluate the incidence of nephrotoxicity due to combination therapy with **vancomycin and aminoglycosides** in septic critically ill patients". The study measures "Serum creatinine and urea concentrations, creatinine clearance", where **creatinine is a measured marker of renal function**, not an administered intervention. There is no mention of creatine being used as a treatment.

**C)** Teve grupo controle?

( ) Sim ( ) Não

**O)** O estudo apresenta valores de marcadores inflamatórios (citocinas e outros marcadores) antes e após as intervenções de creatina?

( ) Sim	( ) Não

**S)** O estudo é um ensaio clínico randomizado e controlado?

( ) Sim	( ) Não

**Artigo 370: Comparison of systemic and renal effects of dopexamine and dopamine in norepinephrine-treated septic shock.**

Schmoelz M, Schelling G, Dunker M, Irlbeck M

**P)** Os participantes possuem mais de 18 anos de idade?

(X) Sim	( ) Não

"Inclusion criteria for this study were the presence of septic shock defined using established criteria,10 the need for norepinephrine in a dose of at least 0.05 g/kg/min, and **patient’s age 18 years**".

**I)** A intervenção utilizada no estudo é creatina? Vale qualquer formato de administração

( ) Sim	(X) Não

"The authors compared the effects of the vasodilatatory agent **dopexamine against renal-dose dopamine and placebo** in patients with norepinephrine therapy and septic shock". The study focuses on "24-hour serum creatinine clearance (Ccrea)" as a measure of renal function, not an intervention.

**C)** Teve grupo controle?

( ) Sim ( ) Não

**O)** O estudo apresenta valores de marcadores inflamatórios (citocinas e outros marcadores) antes e após as intervenções de creatina?

( ) Sim	( ) Não

**S)** O estudo é um ensaio clínico randomizado e controlado?

( ) Sim	( ) Não

**Artigo 371: A comparison of 2 extrafine hydrofluoroalkane-134a-beclomethasone formulations on methacholine hyperresponsiveness.**

Fardon TC, Burns P, Barnes ML, Lipworth BJ

**P)** Os participantes possuem mais de 18 anos de idade?

(X) Sim	( ) Não

"Patients who were recruited were between the ages of **18 and 65 years**".

**I)** A intervenção utilizada no estudo é creatina? Vale qualquer formato de administração

( ) Sim	(X) Não

"The study explicitly aimed 'To compare 2 extrafine solution hydrofluoroalkane-134a formulations of **beclomethasone dipropionate (Beclate and Qvar)**', which are inhaled corticosteroids. While 'creatinine' measurements are mentioned in the context of 'urinary cortisol-creatinine ratio', this refers to a measured physiological marker and not an administered intervention."

**C)** Teve grupo controle?

( ) Sim ( ) Não

**O)** O estudo apresenta valores de marcadores inflamatórios (citocinas e outros marcadores) antes e após as intervenções de creatina?

( ) Sim	( ) Não

**S)** O estudo é um ensaio clínico randomizado e controlado?

( ) Sim	( ) Não

**Artigo 372: [Non-invasive investigation of muscle function using 31P magnetic resonance spectroscopy and 1H MR imaging]**

Bendahan D, Mattei JP, Guis S, Kozak-Ribbens G, Cozzone PJ

**P)** Os participantes possuem mais de 18 anos de idade?

(X) Sim	( ) Não

"The CRMBM protocol has been applied to **more than 4 000 patients and healthy volunteers** over a period of 15 years". The study discusses metabolic changes in "malnourished elderly patients" and in "human" muscle, implicitly covering adult populations. While also mentioning screening "all members of a family at risk including **children**" for malignant hyperthermia, the overall scope includes individuals over 18.

**I)** A intervenção utilizada no estudo é creatina? Vale qualquer formato de administração

(X) Sim	( ) Não

"The latest [therapeutic trial] suggests a beneficial effect of **creatine** which has been demonstrated by a non-invasive **P-31 magnetic resonance spectroscopy** study". Additionally, "**creatine**" is mentioned as one of the "palliative therapies" explored for "mitochondrial myopathies" with its effects "detected on PCr recovery profiles after exercise".

**C)** Teve grupo controle?

(X) Sim ( ) Não

The study highlights the importance of "the definition of normality criteria taking into account the inter-individual heterogeneity characteristic of any normally distributed population". It states that the "comparative analysis of **normal and diseased muscle** remains a major requirement". Furthermore, the authors contributed to "the definition of metabolic normality criteria by analyzing the simultaneous variations of several metabolic parameters on **large cohorts of controls and patients**".

**O)** O estudo apresenta valores de marcadores inflamatórios (citocinas e outros marcadores) antes e após as intervenções de creatina?

( ) Sim	(X) Não

While the study discusses "inflammatory processes" and "muscle inflammation", and mentions "interleukin-1 expression", it primarily focuses on metabolic parameters via MRS. When discussing "metabolic anomalies of inflammatory processes," the study explicitly states that "no direct relationship between these anomalies and the intensity of the inflammatory process has been clearly demonstrated to date". The discussion of creatine as an intervention focuses on its effect on "mitochondrial respiration" and "PCr recovery profiles", with no mention of measuring inflammatory markers (like cytokines) in conjunction with creatine interventions.

**S)** O estudo é um ensaio clínico randomizado e controlado?

( ) Sim	( ) Não

**Artigo 373: Mechanisms and biomarkers of cardiovascular injury induced by phosphodiesterase inhibitor III SK&F 95654 in the spontaneously hypertensive** rat**.**

Zhang J, Herman EH, Robertson DG, Reily MD, Knapton A, Ratajczak HV, Rifai N, Honchel R, Blanchard KT, Stoll RE, Sistare FD

**P)** Os participantes possuem mais de 18 anos de idade?

( ) Sim	(X) Não

"Thirty-seven male SHR **rats** (10 weeks old) were supplied by Harlan Laboratories, Inc. (Indianapolis, IN)." This indicates the study was conducted on animals, not human participants who would be assessed for an age greater than 18 years.

**I)** A intervenção utilizada no estudo é creatina? Vale qualquer formato de administração

( ) Sim	( ) Não

**C)** Teve grupo controle?

( ) Sim ( ) Não

**O)** O estudo apresenta valores de marcadores inflamatórios (citocinas e outros marcadores) antes e após as intervenções de creatina?

( ) Sim	( ) Não

**S)** O estudo é um ensaio clínico randomizado e controlado?

( ) Sim	( ) Não

**Artigo 374: Profibrotic cytokines and lymphocyte proliferation in stable renal allograft recipients treated with or without cyclosporine A.**

Waiser J, Schneider M, Eljazyfer S, Slowinski T, Glander P, Einecke G, Budde K, Neumayer HH, Böhler T

**P)** Os participantes possuem mais de 18 anos de idade?

(X) Sim	( ) Não

"Recipient age (years) **52.9 ± 9.1** (Group A) and **52.6 ± 9.7** (Group B)".

**I)** A intervenção utilizada no estudo é creatina? Vale qualquer formato de administração

( ) Sim	(X) Não

"We assessed the effect of **maintenance immunosuppression with or without cyclosporine A** on TGF-h1 and ET-1 expression as well as lymphocyte proliferation in renal allograft recipients". The study mentions "serum creatinine" and "creatinine clearance" as measurements in other provided sources, but this specific article focuses on immunosuppressive drugs (azathioprine, methylprednisolone, cyclosporine A) as interventions.

**C)** Teve grupo controle?

( ) Sim ( ) Não

**O)** O estudo apresenta valores de marcadores inflamatórios (citocinas e outros marcadores) antes e após as intervenções de creatina?

( ) Sim	( ) Não

**S)** O estudo é um ensaio clínico randomizado e controlado?

( ) Sim	( ) Não

**Artigo 375: Quantitative analysis of viral RNA in the** murine **heart and pancreas with different concentration of coxsackievirus B3.**

Kim EO, Joo CH, Ye JS, Jun EJ, Lee HS, Min WK, Lee MS, Lee H, Kim YK

**P)** Os participantes possuem mais de 18 anos de idade?

( ) Sim	(X) Não

"Forty inbred male **5-week-old A/J mice** (weight range, 20.0–23.4 g) were divided equally into 4 groups and were inoculated intraperitoneally with either 5 ! 10 4 PFU (group 1), 1 ! 10 5 PFU (group 2), 5 ! 10 5 PFU (group 3), or 1 ! 10 6 PFU (group 4) of CVB3 in 200 μl Vero cell lysate".

**I)** A intervenção utilizada no estudo é creatina? Vale qualquer formato de administração

( ) Sim	( ) Não

**C)** Teve grupo controle?

( ) Sim ( ) Não

**O)** O estudo apresenta valores de marcadores inflamatórios (citocinas e outros marcadores) antes e após as intervenções de creatina?

( ) Sim	( ) Não

**S)** O estudo é um ensaio clínico randomizado e controlado?

( ) Sim	( ) Não

**Artigo 376: Urinary transforming growth factor-beta1 in feline chronic renal failure.**

Arata S, Ohmi A, Mizukoshi F, Baba K, Ohno K, Setoguchi A, Tsujimoto H

**P)** Os participantes possuem mais de 18 anos de idade?

( ) Sim	(X) Não

"Cats diagnosed with chronic renal failure (CRF), acute renal failure (ARF), or diabetes mellitus (DM) were selected for this study from **cats** referred to Veterinary Medical Center of the University of Tokyo by other practical veterinarians from June, 2003, to October, 2004.". This study was conducted on **feline (cat) subjects**, not human participants.

**I)** A intervenção utilizada no estudo é creatina? Vale qualquer formato de administração

( ) Sim	( ) Não

**C)** Teve grupo controle?

( ) Sim ( ) Não

**O)** O estudo apresenta valores de marcadores inflamatórios (citocinas e outros marcadores) antes e após as intervenções de creatina?

( ) Sim	( ) Não

**S)** O estudo é um ensaio clínico randomizado e controlado?

( ) Sim	( ) Não

**Artigo 377: Uroperitoneum in 32 foals: influence of intravenous fluid therapy, infection, and sepsis.**

Dunkel B, Palmer JE, Olson KN, Boston RC, Wilkins PA

**P)** Os participantes possuem mais de 18 anos de idade?

( ) Sim	(X) Não

The study was conducted on "32 **Foals**". Foals are young horses, and their age is reported in "days", indicating they are not human participants over 18 years old.

**I)** A intervenção utilizada no estudo é creatina? Vale qualquer formato de administração

( ) Sim	( ) Não

**C)** Teve grupo controle?

( ) Sim ( ) Não

**O)** O estudo apresenta valores de marcadores inflamatórios (citocinas e outros marcadores) antes e após as intervenções de creatina?

( ) Sim	( ) Não

**S)** O estudo é um ensaio clínico randomizado e controlado?

( ) Sim	( ) Não

**Artigo 378: Results from a human renal allograft tolerance** trial **evaluating T-cell depletion with alemtuzumab combined with deoxyspergualin.**

Kirk AD, Mannon RB, Kleiner DE, Swanson JS, Kampen RL, Cendales LK, Elster EA, Wakefield T, Chamberlain C, Hoffmann SC, Hale DA

**P)** Os participantes possuem mais de 18 anos de idade?

(X) Sim	( ) Não

"Five patients aged **28-54 years** (Table 1) scheduled to receive their first live-donor kidney transplant were enrolled after informed consent for study participation was obtained."

**I)** A intervenção utilizada no estudo é creatina? Vale qualquer formato de administração

( ) Sim	(X) Não

"Five recipients of live donor kidneys were treated perioperatively with **alemtuzumab and DSG** [deoxyspergualin] and followed postoperatively without maintenance immunosuppression." The study also mentions that "Deoxyspergualin is a polyamine-like antibiotic known to prevent NFκB nuclear translocation in monocytes and macrophages and to inhibit antigen presentation and activation of these cells". Creatine is not mentioned as an intervention.

**C)** Teve grupo controle?

( ) Sim ( ) Não

**O)** O estudo apresenta valores de marcadores inflamatórios (citocinas e outros marcadores) antes e após as intervenções de creatina?

( ) Sim	( ) Não

**S)** O estudo é um ensaio clínico randomizado e controlado?

( ) Sim	( ) Não

**Artigo 379: Ginkgo biloba extract improves oxidative organ damage in a** rat **model of thermal trauma.**

Sakarcan A, Sehirli O, Velioglu-Ovünç A, Ercan F, Erkanl G, Gedik N, Sener G

**P)** Os participantes possuem mais de 18 anos de idade?

( ) Sim	(X) Não

"Wistar albino **rats of both sexes, weighing 200 to 250 g**, were fasted for 12 h but were allowed free access to water before experiments.". The study was conducted on **rats**, not human participants.

**I)** A intervenção utilizada no estudo é creatina? Vale qualquer formato de administração

( ) Sim	( ) Não

**C)** Teve grupo controle?

( ) Sim ( ) Não

**O)** O estudo apresenta valores de marcadores inflamatórios (citocinas e outros marcadores) antes e após as intervenções de creatina?

( ) Sim	( ) Não

**S)** O estudo é um ensaio clínico randomizado e controlado?

( ) Sim	( ) Não

**Artigo 380: Effect of energy source on the efficiency of translational termination during cell-free protein synthesis.**

Ahn JH, Choi CY, Kim DM

**P)** Os participantes possuem mais de 18 anos de idade?

( ) Sim	(X) Não

The study investigates "translational termination during **cell-free protein synthesis**". It explicitly mentions the use of "crude extract derived from the cytosol of **E. coli**" for this process, indicating that it does not involve human participants.

**I)** A intervenção utilizada no estudo é creatina? Vale qualquer formato de administração

( ) Sim	( ) Não

**C)** Teve grupo controle?

( ) Sim ( ) Não

**O)** O estudo apresenta valores de marcadores inflamatórios (citocinas e outros marcadores) antes e após as intervenções de creatina?

( ) Sim	( ) Não

**S)** O estudo é um ensaio clínico randomizado e controlado?

( ) Sim	( ) Não

**Artigo 381: Clinical efficacy of intravenous immunoglobulin for patients with MPO-ANCA-associated rapidly progressive glomerulonephritis.**

Ito-Ihara T, Ono T, Nogaki F, Suyama K, Tanaka M, Yonemoto S, Fukatsu A, Kita T, Suzuki K, Muso E

**P)** Os participantes possuem mais de 18 anos de idade?

(X) Sim	( ) Não

"Twelve consecutive patients... **mean age 72 years; range 57–83 years**".

**I)** A intervenção utilizada no estudo é creatina? Vale qualquer formato de administração

( ) Sim	(X) Não

The study evaluated the effects of the intervention "by comparing renal function, clinical score... and circulating TNF-α levels **before and after IVIg treatment**" within the same group of 12 patients. While "normal controls" were used for baseline comparison of certain markers like TNF-α levels, there was no concurrent control group receiving a different intervention or placebo as part of the study's primary design.

**C)** Teve grupo controle?

( ) Sim ( ) Não

**O)** O estudo apresenta valores de marcadores inflamatórios (citocinas e outros marcadores) antes e após as intervenções de creatina?

( ) Sim	( ) Não

**S)** O estudo é um ensaio clínico randomizado e controlado?

( ) Sim	( ) Não

**Artigo 382: [Pharmacological treatments for Duchenne and Becker dystrophies].**

Voisin V, de la Porte S

**P)** Os participantes possuem mais de 18 anos de idade?

( ) Sim	(X) Não

"La dystrophie musculaire de Duchenne (DMD) ... touche **1 garçon sur 3 500**. Les premiers signes cliniques surviennent au moment de l’acquisition de la marche, et deviennent évidents vers **l’âge de 5-6 ans**." "la dystrophie musculaire de Becker (DMB) ... L’âge de début de la maladie se situe **entre 2 et 20 ans (en moyenne vers 12 ans)**".

**I)** A intervenção utilizada no estudo é creatina? Vale qualquer formato de administração

( ) Sim	( ) Não

**C)** Teve grupo controle?

( ) Sim ( ) Não

**O)** O estudo apresenta valores de marcadores inflamatórios (citocinas e outros marcadores) antes e após as intervenções de creatina?

( ) Sim	( ) Não

**S)** O estudo é um ensaio clínico randomizado e controlado?

( ) Sim	( ) Não

**Artigo 383: Medication and supplement use by athletes.**

Ciocca M

**P)** Os participantes possuem mais de 18 anos de idade?

( ) Sim	(X) Não

"This article first reviews common medicines that athletes use and their potential efficacy and interactions with exercise and environment, then reviews dietary supplements and the data on their efficacy for performance enhancement. Finally, current and future doping issues are addressed". This indicates that the article is a **review article** and, therefore, does not have its own direct study participants.

**I)** A intervenção utilizada no estudo é creatina? Vale qualquer formato de administração

( ) Sim	( ) Não

**C)** Teve grupo controle?

( ) Sim ( ) Não

**O)** O estudo apresenta valores de marcadores inflamatórios (citocinas e outros marcadores) antes e após as intervenções de creatina?

( ) Sim	( ) Não

**S)** O estudo é um ensaio clínico randomizado e controlado?

( ) Sim	( ) Não

**Artigo 384: Relocation of truncated bid plays an important role in suppression of tumor necrosis factor alpha induced apoptosis in hepatocytes isolated from** transgenic mouse**.**

Shiotani T, Yamanokuchi S, Hatano E, Ikai I

**P)** Os participantes possuem mais de 18 anos de idade?

( ) Sim	(X) Não

"Nine-week-old wild-type and CK transgenic male **mice** were anesthetized by an intraperitoneal injection of ketamine/acepromazine malate."

**I)** A intervenção utilizada no estudo é creatina? Vale qualquer formato de administração

( ) Sim	( ) Não

**C)** Teve grupo controle?

( ) Sim ( ) Não

**O)** O estudo apresenta valores de marcadores inflamatórios (citocinas e outros marcadores) antes e após as intervenções de creatina?

( ) Sim	( ) Não

**S)** O estudo é um ensaio clínico randomizado e controlado?

( ) Sim	( ) Não

**Artigo 385: Inhibition of matrix metalloproteinases during chronic allograft nephropathy in** rats

Lutz J, Yao Y, Song E, Antus B, Hamar P, Liu S, Heemann U

**P)** Os participantes possuem mais de 18 anos de idade?

( ) Sim	(X) Não

"Methods. **Fisher kidneys were orthotopically transplanted into Lewis recipients that were treated with BAY 12–9566** (15 mg/kg per day) or vehicle...". The study was conducted on **rats**, not human participants.

**I)** A intervenção utilizada no estudo é creatina? Vale qualquer formato de administração

( ) Sim	( ) Não

**C)** Teve grupo controle?

( ) Sim ( ) Não

**O)** O estudo apresenta valores de marcadores inflamatórios (citocinas e outros marcadores) antes e após as intervenções de creatina?

( ) Sim	( ) Não

**S)** O estudo é um ensaio clínico randomizado e controlado?

( ) Sim	( ) Não

**Artigo 386: The synthetic pentasaccharide fondaparinux reduces coagulation, inflammation and neutrophil accumulation in kidney ischemia-reperfusion injury.**

Frank RD, Schabbauer G, Holscher T, Sato Y, Tencati M, Pawlinski R, Mackman N

**P)** Os participantes possuem mais de 18 anos de idade?

( ) Sim	(X) Não

The study utilized "**A murine model of kidney I/R was established**", indicating that the participants were **mice**, not human subjects.

**I)** A intervenção utilizada no estudo é creatina? Vale qualquer formato de administração

( ) Sim	( ) Não

**C)** Teve grupo controle?

( ) Sim ( ) Não

**O)** O estudo apresenta valores de marcadores inflamatórios (citocinas e outros marcadores) antes e após as intervenções de creatina?

( ) Sim	( ) Não

**S)** O estudo é um ensaio clínico randomizado e controlado?

( ) Sim	( ) Não

**Artigo 387: A technique for the measurement of renal ATP in a large** animal **model of septic shock.**

May C, Wan L, Williams J, Wellard MR, Pell G, Jackson G, Bellomo R

**P)** Os participantes possuem mais de 18 anos de idade?

( ) Sim	(X) Não

"A technique for the measurement of renal ATP in a **large animal model of septic shock**" and "It is possible to sequentially monitor renal bioenergetics in a **large mammal** during septic shock". The study was conducted on animals, not human participants.

**I)** A intervenção utilizada no estudo é creatina? Vale qualquer formato de administração

( ) Sim	( ) Não

**C)** Teve grupo controle?

( ) Sim ( ) Não

**O)** O estudo apresenta valores de marcadores inflamatórios (citocinas e outros marcadores) antes e após as intervenções de creatina?

( ) Sim	( ) Não

**S)** O estudo é um ensaio clínico randomizado e controlado?

( ) Sim	( ) Não

**Artigo 388: Ratio of cyclase activating and cyclase inactive parathormone (CAP/CIP) in dialysis patients: correlations with other markers of bone disease.**

Grzegorzewska AE, Młot M

**P)** Os participantes possuem mais de 18 anos de idade?

(X) Sim	( ) Não

“The study was performed in 30 patients (22 were treated with HD, 8 – with PD). Mean patients’ age was 61.6 (36.7 - 79.6) years. Patients were treated with dialysis for 15.2 (1.3 - 186.3) months. Dialysis duration was significantly longer in HD patients than in PD ones (p = 0.014; 26.7, 3.5 - 186.3 months for HD vs 5.0, 1.3 - 26.7 months for PD)."

**I)** A intervenção utilizada no estudo é creatina? Vale qualquer formato de administração

( ) Sim	(X) Não

“ In this study we checked correlation of CAP/CIP with osteoprotegrin (OPG), its soluble ligand (OPGL) and routinely measured parameters of bone turnover in patients treated with peritoneal dialysis (PD) or hemodialysis (HD). “

**C)** Teve grupo controle?

( ) Sim ( ) Não

**O)** O estudo apresenta valores de marcadores inflamatórios (citocinas e outros marcadores) antes e após as intervenções de creatina?

( ) Sim	( ) Não

**S)** O estudo é um ensaio clínico randomizado e controlado?

( ) Sim	( ) Não

**Artigo 389: Stay alert for performance-enhancing substance use.**

Jarvi JF

**P)** Os participantes possuem mais de 18 anos de idade?

( ) Sim	( ) Não [*Excluir*]

**I)** A intervenção utilizada no estudo é creatina? Vale qualquer formato de administração

( ) Sim	(	) Não [*Excluir*]

**C)** Teve grupo controle?

(	) Sim ( ) Não [*Excluir*]

**O)** O estudo apresenta valores de marcadores inflamatórios (citocinas e outros marcadores) antes e após as intervenções de creatina?

( ) Sim	(	) Não [*Excluir*]

**S)** O estudo é um ensaio clínico randomizado e controlado?

(	) Sim	( ) Não

**Artigo 390: A multicenter in vivo proton-MRS study of HIV-associated dementia and its relationship to age.**

Chang L, Lee PL, Yiannoutsos CT, Ernst T, Marra CM, Richards T, Kolson D, Schifitto G, Jarvik JG, Miller EN, Lenkinski R, Gonzalez G, Navia BA

**P)** Os participantes possuem mais de 18 anos de idade?

(X) Sim	( ) Não

"A group of 161 subjects, 121 HIV-positive subjects, and 40 SN controls, fulfilled the study criteria and were recruited for the study". The participants included "HIV patients" and "seronegative controls" with mean ages (e.g., "34 years" for SN controls and "37 years" for NAS controls) indicative of an adult population. Furthermore, "All subjects signed consent forms approved by the institutional review board at each institution as well as by the ACTG", which is standard practice for adult human subjects in research studies. Other similar studies in the provided sources explicitly state inclusion criteria for participants being 18 years or older (e.g., "age 18–65 years" or "age ≥ 18 years old").

**I)** A intervenção utilizada no estudo é creatina? Vale qualquer formato de administração

( ) Sim	(X) Não

The study is described as a "proton-MRS study" that measured "myoinositol-to-creatine ratio (MI/Cr)", "choline compounds (Cho)/Cr", and "N-acetylaspartate-to-creatine ratio (NA/Cr)". Creatine (Cr) served as a reference metabolite for calculating ratios of other brain metabolites, not as an intervention or administered substance in any format.

**C)** Teve grupo controle?

( ) Sim ( ) Não

**O)** O estudo apresenta valores de marcadores inflamatórios (citocinas e outros marcadores) antes e após as intervenções de creatina?

( ) Sim	( ) Não

**S)** O estudo é um ensaio clínico randomizado e controlado?

( ) Sim	( ) Não

**Artigo 391: Helicobacter bilis-associated hepatitis in outbred** mice**.**

Fox JG, Rogers AB, Whary MT, Taylor NS, Xu S, Feng Y, Keys S

**P)** Os participantes possuem mais de 18 anos de idade?

( ) Sim	( ) Não [*Excluir*]

**I)** A intervenção utilizada no estudo é creatina? Vale qualquer formato de administração

( ) Sim	(	) Não [*Excluir*]

**C)** Teve grupo controle?

(	) Sim ( ) Não [*Excluir*]

**O)** O estudo apresenta valores de marcadores inflamatórios (citocinas e outros marcadores) antes e após as intervenções de creatina?

( ) Sim	(	) Não [*Excluir*]

**S)** O estudo é um ensaio clínico randomizado e controlado?

(	) Sim	( ) Não

**Artigo 392: Effect of retinoic acid in experimental diabetic nephropathy.**

Han SY, So GA, Jee YH, Han KH, Kang YS, Kim HK, Kang SW, Han DS, Han JY, Cha DR

**P)** Os participantes possuem mais de 18 anos de idade?

( ) Sim	(X) Não

"Male Sprague–Dawley rats, weighing 162 to 184 g at the beginning of the experiments were subjected to right uninephrectomy under anesthesia with sodium pentobarbital (50 mg/kg, i.p.). After 10 days, rats were randomly treated with one of the following combinations for 3 or 6 weeks". "Diabetic rats were confirmed when the tail blood sugar concentration was greater than 300 mg/dL, 48 h after STZ injection". The study was conducted on **rats**, not human subjects, therefore, they do not possess more than 18 years of age.

**I)** A intervenção utilizada no estudo é creatina? Vale qualquer formato de administração

( ) Sim	( ) Não

**C)** Teve grupo controle?

( ) Sim ( ) Não

**O)** O estudo apresenta valores de marcadores inflamatórios (citocinas e outros marcadores) antes e após as intervenções de creatina?

( ) Sim	( ) Não

**S)** O estudo é um ensaio clínico randomizado e controlado?

( ) Sim	( ) Não

**Artigo 393: Sensitive noninvasive marker for the diagnosis of probable bacterial or viral infection**

Jortani SA, Pugia MJ, Elin RJ, Thomas M, Womack EP, Cast T, Valdes R Jr

**P)** Os participantes possuem mais de 18 anos de idade?

(X) Sim	( ) Não

"The mean age for enrolled controls (n=90) was **39.6±11.6 years**, which was not statistically different from that of the patients (n=171; **41.1±16.1 years**; P=0.39)". This indicates that all participants, both controls and patients, were adults with mean ages well above 18 years.

**I)** A intervenção utilizada no estudo é creatina? Vale qualquer formato de administração

( ) Sim	(X) Não

The study's objective was "to compare the sensitivity of uTi in urine with that of serum quantitative C-reactive protein (CRP) for diagnosing infection". While "creatinine concentrations" were measured in urine samples to normalize uTi values, **creatine itself was not an intervention or administered substance** in any form. The study focused on diagnostic markers for infection, not on the effects of creatine administration.

**C)** Teve grupo controle?

( ) Sim ( ) Não

**O)** O estudo apresenta valores de marcadores inflamatórios (citocinas e outros marcadores) antes e após as intervenções de creatina?

( ) Sim	( ) Não

**S)** O estudo é um ensaio clínico randomizado e controlado?

( ) Sim	( ) Não

**Artigo 394: Relapsing-remitting multiple sclerosis: metabolic abnormality in nonenhancing lesions and normal-appearing white matter at MR imaging: initial experience.**

He J, Inglese M, Li BS, Babb JS, Grossman RI, Gonen O

**P)** Os participantes possuem mais de 18 anos de idade?

(X) Sim	( ) Não

"Nine patients with relapsing-remitting MS (six women, three men; mean age, 38.9 years; **age range, 26–47 years**) were recruited from an ongoing serial study". Additionally, "Nine age-matched healthy volunteers (seven women, two men; mean age, 38 years; **age range, 21–48 years**) underwent the same MR imaging and MR spectroscopy procedures". Both patient and control groups consisted of individuals well over 18 years of age.

**I)** A intervenção utilizada no estudo é creatina? Vale qualquer formato de administração

( ) Sim	(X) Não

The study's purpose was "To quantify, with three-dimensional proton magnetic resonance (MR) spectroscopy, metabolic characteristics of normal-appearing white matter and non-enhancing lesions in patients with relapsing-remitting multiple sclerosis (MS)". **Creatine (Cr)** was measured as one of the "absolute... levels" of brain metabolites along with N-acetylaspartate (NAA) and choline (Cho), serving as a reference metabolite in spectroscopy. It was not administered or used as an intervention.

**C)** Teve grupo controle?

( ) Sim ( ) Não

**O)** O estudo apresenta valores de marcadores inflamatórios (citocinas e outros marcadores) antes e após as intervenções de creatina?

( ) Sim	( ) Não

**S)** O estudo é um ensaio clínico randomizado e controlado?

( ) Sim	( ) Não

**Artigo 395: Elevation of myoinositol is associated with disease containment in progressive multifocal leukoencephalopathy.**

Katz-Brull R, Lenkinski RE, Du Pasquier RA, Koralnik IJ

**P)** Os participantes possuem mais de 18 anos de idade?

( ) Sim	(X) Não

"The provided excerpt for Artigo 395 refers to 'patients with progressive multifocal leukoencephalopathy (PML)', but **does not explicitly state the age of the participants or provide a range to confirm they are over 18 years old.**"

**I)** A intervenção utilizada no estudo é creatina? Vale qualquer formato de administração

( ) Sim	( ) Não

**C)** Teve grupo controle?

( ) Sim ( ) Não

**O)** O estudo apresenta valores de marcadores inflamatórios (citocinas e outros marcadores) antes e após as intervenções de creatina?

( ) Sim	( ) Não

**S)** O estudo é um ensaio clínico randomizado e controlado?

( ) Sim	( ) Não

**Artigo 396: Renal effects of norepinephrine in septic and nonseptic patients.**

Albanèse J, Leone M, Garnier F, Bourgoin A, Antonini F, Martin C

**P)** Os participantes possuem mais de 18 anos de idade?

(X) Sim	( ) Não

"During a 16-month period, 14 consecutive patients (10 men and four women; **mean [ SD] age, 51 12 years**) with septic shock were prospectively studied". The mean age of 51 years with a standard deviation of 12 years strongly indicates that all participants were adults, well over 18 years old.

**I)** A intervenção utilizada no estudo é creatina? Vale qualquer formato de administração

( ) Sim	(X) Não

The study's objective was "To assess the effects of a **norepinephrine-induced vasoconstriction** on renal function in septic and nonseptic patients". Patients received "norepinephrine infusion" as the intervention. Creatine was not an intervention or administered substance in any format.

**C)** Teve grupo controle?

( ) Sim ( ) Não

**O)** O estudo apresenta valores de marcadores inflamatórios (citocinas e outros marcadores) antes e após as intervenções de creatina?

( ) Sim	( ) Não

**S)** O estudo é um ensaio clínico randomizado e controlado?

( ) Sim	( ) Não

**Artigo 397: Antiretroviral treatment alters relationship between MCP-1 and neurometabolites in HIV patients.**

Chang L, Ernst T, St Hillaire C, Conant K

**P)** Os participantes possuem mais de 18 anos de idade?

(X) Sim	( ) Não

"Thirty-nine HIV patients naive to antiretroviral medications (36 men and three women, **age 34.9 ±1.5 years**) were recruited for this prospective study. [...] they fulfilled the inclusion criteria: 1) **age 18–65 years**".

**I)** A intervenção utilizada no estudo é creatina? Vale qualquer formato de administração

( ) Sim	(X) Não

The study's objective was to evaluate "the relationships between neurometabolites and macrophage chemoattractant protein (MCP-1) in serum and cerebrospinal fluid (CSF) [...] before and after **antiretroviral treatment**". Specifically, patients were given "highly active antiretroviral therapy (HAART)". Creatine (Cr) was a "control metabolite" measured by proton magnetic resonance spectroscopy (1H MRS) to assess neurometabolite ratios, not an administered intervention.

**C)** Teve grupo controle?

( ) Sim ( ) Não

**O)** O estudo apresenta valores de marcadores inflamatórios (citocinas e outros marcadores) antes e após as intervenções de creatina?

( ) Sim	( ) Não

**S)** O estudo é um ensaio clínico randomizado e controlado?

( ) Sim	( ) Não

**Artigo 398: In vivo 1H MRS of brain injury and repair during acute SIV infection in the macaque model of neuroAIDS.**

Greco JB, Westmoreland SV, Ratai EM, Lentz MR, Sakaie K, He J, Sehgal PK, Masliah E, Lackner AA, González RG

**P)** Os participantes possuem mais de 18 anos de idade?

( ) Sim	(X) Não

"Fifteen **juvenile (ages 1.5–3 years) rhesus macaques** (Macaca mulatta), including 10 males and 5 females, were inoculated intravenously with SIVmac251 (50 ng p27/kg)." This indicates the participants are young non-human primates, not humans over 18 years of age.

**I)** A intervenção utilizada no estudo é creatina? Vale qualquer formato de administração

( ) Sim	( ) Não

**C)** Teve grupo controle?

( ) Sim ( ) Não

**O)** O estudo apresenta valores de marcadores inflamatórios (citocinas e outros marcadores) antes e após as intervenções de creatina?

( ) Sim	( ) Não

**S)** O estudo é um ensaio clínico randomizado e controlado?

( ) Sim	( ) Não

**Artigo 399: A prospective** longitudinal **in vivo 1H MR spectroscopy study of the SIV/macaque model of neuroAIDS.**

Fuller RA, Westmoreland SV, Ratai E, Greco JB, Kim JP, Lentz MR, He J, Sehgal PK, Masliah E, Halpern E, Lackner AA, González RG

**P)** Os participantes possuem mais de 18 anos de idade?

( ) Sim	(X) Não

"Eight **juvenile rhesus macaques** (Macaca mulatta) were inoculated intravenously with SIVmac251 (50 ng p27/kg)". This indicates the participants are young non-human primates, not humans over 18 years of age.

**I)** A intervenção utilizada no estudo é creatina? Vale qualquer formato de administração

( ) Sim	( ) Não

**C)** Teve grupo controle?

( ) Sim ( ) Não

**O)** O estudo apresenta valores de marcadores inflamatórios (citocinas e outros marcadores) antes e após as intervenções de creatina?

( ) Sim	( ) Não

**S)** O estudo é um ensaio clínico randomizado e controlado?

( ) Sim	( ) Não

**Artigo 400: Macrophage index predicts short-term renal allograft function and graft survival.**

Srinivas TR, Kubilis PS, Croker BP

**P)** Os participantes possuem mais de 18 anos de idade?

(X) Sim	( ) Não

"The study population (n=41) was drawn from patients who had received transplants at the University of Florida and had undergone a renal allograft biopsy between February 1993 and June 1994". The study focuses on "renal allograft dysfunction" and "graft survival". While a specific age range is not explicitly stated, kidney transplantation and its complications are medical conditions typically affecting adults. Other sources provided, dealing with similar patient populations and clinical settings, explicitly include participants "aged more than 18 years" or report mean ages well above 18, supporting the inference that these patients were adults.

**I)** A intervenção utilizada no estudo é creatina? Vale qualquer formato de administração

( ) Sim	(X) Não

The study's objective was to "predict renal allograft dysfunction in the short term and predict graft survival by quantifying the macrophage infiltrate in allograft renal biopsies". The interventions mentioned relate to standard care for transplant patients: "Maintenance immunosuppression was cyclosporine, azathioprine and prednisone. Primary acute rejection therapy was intravenous methylprednisone pulse. OKT3 was employed for steroid-resistant acute rejection". Creatine was not used as an intervention in any form.

**C)** Teve grupo controle?

( ) Sim ( ) Não

**O)** O estudo apresenta valores de marcadores inflamatórios (citocinas e outros marcadores) antes e após as intervenções de creatina?

( ) Sim	( ) Não

**S)** O estudo é um ensaio clínico randomizado e controlado?

( ) Sim	( ) Não

**Artigo 401: Erythropoietin receptor expression in adult** rat **cardiomyocytes is associated with an acute cardioprotective effect for recombinant erythropoietin during ischemia-reperfusion injury.**

Wright GL, Hanlon P, Amin K, Steenbergen C, Murphy E, Arcasoy MO

**P)** Os participantes possuem mais de 18 anos de idade?

( ) Sim	(X) Não

"Male Sprague-Dawley **rats** weighing ~250–300 g were used in this study". This indicates the participants are non-human animals (rats), not humans over 18 years of age.

**I)** A intervenção utilizada no estudo é creatina? Vale qualquer formato de administração

( ) Sim	( ) Não

**C)** Teve grupo controle?

( ) Sim ( ) Não

**O)** O estudo apresenta valores de marcadores inflamatórios (citocinas e outros marcadores) antes e após as intervenções de creatina?

( ) Sim	( ) Não

**S)** O estudo é um ensaio clínico randomizado e controlado?

( ) Sim	( ) Não

**Artigo 402: Comparative effects of vasopressin, norepinephrine, and L-canavanine, a selective inhibitor of inducible nitric oxide synthase, in endotoxic shock.**

Levy B, Vallée C, Lauzier F, Plante GE, Mansart A, Mallie JP, Lesur O

**P)** Os participantes possuem mais de 18 anos de idade?

( ) Sim	(X) Não

"Wistar rats (300–320 g) fasted overnight were anesthetized with ketamine-xylazine (50 and 20 mg/kg, respectively)". The study was conducted on **rats**, not human subjects.

**I)** A intervenção utilizada no estudo é creatina? Vale qualquer formato de administração

( ) Sim	( ) Não

**C)** Teve grupo controle?

( ) Sim ( ) Não

**O)** O estudo apresenta valores de marcadores inflamatórios (citocinas e outros marcadores) antes e após as intervenções de creatina?

( ) Sim	( ) Não

**S)** O estudo é um ensaio clínico randomizado e controlado?

( ) Sim	( ) Não

**Artigo 403: Additive neuroprotective effects of creatine and cyclooxygenase 2 inhibitors in a** transgenic mouse **model of amyotrophic lateral sclerosis.**

Klivenyi P, Kiaei M, Gardian G, Calingasan NY, Beal MF

**P)** Os participantes possuem mais de 18 anos de idade?

( ) Sim	(X) Não

"Transgenic **mice** with the G93A mutation of the human SOD1 gene". The study was conducted on **mice**, not human subjects.

**I)** A intervenção utilizada no estudo é creatina? Vale qualquer formato de administração

( ) Sim	( ) Não

**C)** Teve grupo controle?

( ) Sim ( ) Não

**O)** O estudo apresenta valores de marcadores inflamatórios (citocinas e outros marcadores) antes e após as intervenções de creatina?

( ) Sim	( ) Não

**S)** O estudo é um ensaio clínico randomizado e controlado?

( ) Sim	( ) Não

**Artigo 404: Prediction of viability of** porcine **neurocysticercosis with proton magnetic resonance spectroscopy: correlation with histopathology.**

Chawla S, Gupta RK, Husain N, Garg M, Kumar R, Kumar S

**P)** Os participantes possuem mais de 18 anos de idade?

( ) Sim	(X) Não

"Prediction of viability of **porcine neurocysticercosis** with proton magnetic resonance spectroscopy: Correlation with histopathology". The study was conducted on **pigs**, not human subjects.

**I)** A intervenção utilizada no estudo é creatina? Vale qualquer formato de administração

( ) Sim	( ) Não

**C)** Teve grupo controle?

( ) Sim ( ) Não

**O)** O estudo apresenta valores de marcadores inflamatórios (citocinas e outros marcadores) antes e após as intervenções de creatina?

( ) Sim	( ) Não

**S)** O estudo é um ensaio clínico randomizado e controlado?

( ) Sim	( ) Não

**Artigo 405: Additive neuroprotective effects of creatine and a cyclooxygenase 2 inhibitor against dopamine depletion in the 1-methyl-4-phenyl-1,2,3,6-tetrahydropyridine (MPTP)** mouse**model of Parkinson's disease.**

Klivenyi P, Gardian G, Calingasan NY, Yang L, Beal MF

**P)** Os participantes possuem mais de 18 anos de idade?

( ) Sim	(X) Não

"Male **3-mo-old Swiss-Webster mice** were fed lab chow diets supplemented with 2% creatine, or 0.005% rofecoxib or their combination for 1 wk before MPTP administration". The study was conducted on **mice**, not human subjects.

**I)** A intervenção utilizada no estudo é creatina? Vale qualquer formato de administração

( ) Sim	( ) Não

**C)** Teve grupo controle?

( ) Sim ( ) Não

**O)** O estudo apresenta valores de marcadores inflamatórios (citocinas e outros marcadores) antes e após as intervenções de creatina?

( ) Sim	( ) Não

**S)** O estudo é um ensaio clínico randomizado e controlado?

( ) Sim	( ) Não

**Artigo 406: LPS-induced changes in myocardial markers in neonatal** rats**.**

Ravindranath TM, Goto M, Bakr S, Ramasamy R

**P)** Os participantes possuem mais de 18 anos de idade?

( ) Sim	(X) Não

"Ten-day-old Sprague-Dawley rats were given an i.p. injection of 0.1 mg/kg of Salmonella enteritidis endotoxin (LPS; Difco, Detroit, Mich., USA) or 0.2 ml saline (control) after a 4-hour fast". The study was conducted on **neonatal rats**, not human subjects.

**I)** A intervenção utilizada no estudo é creatina? Vale qualquer formato de administração

( ) Sim	( ) Não

**C)** Teve grupo controle?

( ) Sim ( ) Não

**O)** O estudo apresenta valores de marcadores inflamatórios (citocinas e outros marcadores) antes e após as intervenções de creatina?

( ) Sim	( ) Não

**S)** O estudo é um ensaio clínico randomizado e controlado?

( ) Sim	( ) Não

**Artigo 407: Metabolic changes in the vicinity of brain contusions: a proton magnetic resonance spectroscopy and histology study.**

Schuhmann MU, Stiller D, Skardelly M, Bernarding J, Klinge PM, Samii A, Samii M, Brinker T

**P)** Os participantes possuem mais de 18 anos de idade?

( ) Sim	( ) Não [*Excluir*]

**I)** A intervenção utilizada no estudo é creatina? Vale qualquer formato de administração

( ) Sim	(	) Não [*Excluir*]

**C)** Teve grupo controle?

(	) Sim ( ) Não [*Excluir*]

**O)** O estudo apresenta valores de marcadores inflamatórios (citocinas e outros marcadores) antes e após as intervenções de creatina?

( ) Sim	(	) Não [*Excluir*]

**S)** O estudo é um ensaio clínico randomizado e controlado?

(	) Sim	( ) Não

**Artigo 408: Increased glial metabolites predict increased working memory network activation in HIV brain injury.**

Ernst T, Chang L, Arnold S

**P)** Os participantes possuem mais de 18 anos de idade?

(X) Sim	( ) Não

"Fourteen HIV-1-positive men (**age 37.8 ± 7.1 years**) were scanned with 1H MRS and fMRI".

**I)** A intervenção utilizada no estudo é creatina? Vale qualquer formato de administração

( ) Sim	(X) Não

The study "measured metabolite concentrations with 1H MRS in the frontal gray and white matter, and basal ganglia" which included "**total creatine**" as a "glial metabolite", indicating it was a measured compound, not an administered intervention.

**C)** Teve grupo controle?

( ) Sim ( ) Não

**O)** O estudo apresenta valores de marcadores inflamatórios (citocinas e outros marcadores) antes e após as intervenções de creatina?

( ) Sim	( ) Não

**S)** O estudo é um ensaio clínico randomizado e controlado?

( ) Sim	( ) Não

**Artigo 409: Isotretinoin ameliorates renal damage in experimental acute renal allograft rejection.**

Kiss E, Adams J, Gröne HJ, Wagner J

**P)** Os participantes possuem mais de 18 anos de idade?

( ) Sim	(X) Não

"Kidneys of **Fisher rats** (F344, RT11v1) were orthotopically grafted to Lewis rats (RT11)". The study was conducted on **rats**, not human subjects.

**I)** A intervenção utilizada no estudo é creatina? Vale qualquer formato de administração

( ) Sim	( ) Não

**C)** Teve grupo controle?

( ) Sim ( ) Não

**O)** O estudo apresenta valores de marcadores inflamatórios (citocinas e outros marcadores) antes e após as intervenções de creatina?

( ) Sim	( ) Não

**S)** O estudo é um ensaio clínico randomizado e controlado?

( ) Sim	( ) Não

**Artigo 410: Mitochondria, oxidative damage, and inflammation in Parkinson's disease.**

Beal MF

**P)** Os participantes possuem mais de 18 anos de idade?

( ) Sim	(X) Não

"The provided article is a review paper titled 'Mitochondria, oxidative damage, and inflammation in Parkinson's disease'. As a review, it synthesizes findings from multiple studies rather than presenting data from a single, original study with its own participants. While the article discusses various studies, including a 'small phase II clinical trial' involving 'patients' with Parkinson's disease, and Parkinson's disease is typically an adult-onset condition, there is no explicit statement within the provided text that specifies the age of these participants or confirms they are over 18 years of age. Therefore, the requested information cannot be directly transcribed from the source."

**I)** A intervenção utilizada no estudo é creatina? Vale qualquer formato de administração

( ) Sim	( ) Não

**C)** Teve grupo controle?

( ) Sim ( ) Não

**O)** O estudo apresenta valores de marcadores inflamatórios (citocinas e outros marcadores) antes e após as intervenções de creatina?

( ) Sim	( ) Não

**S)** O estudo é um ensaio clínico randomizado e controlado?

( ) Sim	( ) Não

**Artigo 411: Diffusely elevated cerebral choline and creatine in relapsing-remitting multiple sclerosis.**

Inglese M, Li BS, Rusinek H, Babb JS, Grossman RI, Gonen O

**P)** Os participantes possuem mais de 18 anos de idade?

(X) Sim	( ) Não

"11 MS patients (9 women, two men), **mean age 32 (range 21–48) years**, were recruited for this study."

**I)** A intervenção utilizada no estudo é creatina? Vale qualquer formato de administração

( ) Sim	(X) Não

The study aimed to "compare the absolute levels of N-acetylaspartate (NAA), **creatine (Cr)**, and choline (Cho) in the normal-appearing white matter (NAWM) between relapsing-remitting (RR) MS patients and controls". It "assessed the metabolic characteristics of RR MS patients" by "comparing the concentrations, [NAA], [Cho], and **[Cr]**, with their matched controls". Creatine was a measured metabolite, not an administered intervention.

**C)** Teve grupo controle?

( ) Sim ( ) Não

**O)** O estudo apresenta valores de marcadores inflamatórios (citocinas e outros marcadores) antes e após as intervenções de creatina?

( ) Sim	( ) Não

**S)** O estudo é um ensaio clínico randomizado e controlado?

( ) Sim	( ) Não

**Artigo 412: TGF-beta1 expression and chronic allograft nephropathy in protocol kidney graft biopsy.**

Viklický O, Matl I, Voska L, Böhmová R, Jaresová M, Lácha J, Lodererová A, Stríz I, Teplan V, Vítko S

**P)** Os participantes possuem mais de 18 anos de idade?

(X) Sim	( ) Não

"A total of **67 patients (mean age 52.1±11.9 years**, 47 males and 20 females) were enrolled into the study."

**I)** A intervenção utilizada no estudo é creatina? Vale qualquer formato de administração

( ) Sim	(X) Não

The study investigates "TGF-β1 expression and chronic allograft nephropathy". It states that "All patients received triple immunosuppression based on cyclosporine A". **Serum creatinine** was a "clinical data" point measured to estimate glomerular filtration rate, but creatine itself was not an intervention administered in any format.

**C)** Teve grupo controle?

( ) Sim ( ) Não

**O)** O estudo apresenta valores de marcadores inflamatórios (citocinas e outros marcadores) antes e após as intervenções de creatina?

( ) Sim	( ) Não

**S)** O estudo é um ensaio clínico randomizado e controlado?

( ) Sim	( ) Não

**Artigo 413: Early- and late-state subacute sclerosing panencephalitis: chemical shift imaging and single-voxel MR spectroscopy.**

Alkan A, Sarac K, Kutlu R, Yakinci C, Sigirci A, Aslan M, Baysal T

**P)** Os participantes possuem mais de 18 anos de idade?

( ) Sim	(X) Não

"The study group included six consecutive children (three boys, three girls; **mean age, 6.8 years**) with the diagnosis of SSPE... Children with SSPE were compared with 10 healthy, age-matched children (six boys and four girls) aged 6–9 years (**mean age, 7.8 years**)". The study was conducted on children, not adults.

**I)** A intervenção utilizada no estudo é creatina? Vale qualquer formato de administração

( ) Sim	( ) Não

**C)** Teve grupo controle?

( ) Sim ( ) Não

**O)** O estudo apresenta valores de marcadores inflamatórios (citocinas e outros marcadores) antes e após as intervenções de creatina?

( ) Sim	( ) Não

**S)** O estudo é um ensaio clínico randomizado e controlado?

( ) Sim	( ) Não

**Artigo 414: Effects of epinephrine and norepinephrine on hemodynamics, oxidative metabolism, and organ energetics in endotoxemic** rats**.**

Levy B, Mansart A, Bollaert PE, Franck P, Mallie JP

**P)** Os participantes possuem mais de 18 anos de idade?

( ) Sim	(X) Não

"Wistar **rats** (300–320 g) fasted overnight were anesthetized with thiopental sodium (30 mg/kg intraperitoneally)". The study was conducted on **rats**, not human subjects.

**I)** A intervenção utilizada no estudo é creatina? Vale qualquer formato de administração

( ) Sim	( ) Não

**C)** Teve grupo controle?

( ) Sim ( ) Não

**O)** O estudo apresenta valores de marcadores inflamatórios (citocinas e outros marcadores) antes e após as intervenções de creatina?

( ) Sim	( ) Não

**S)** O estudo é um ensaio clínico randomizado e controlado?

( ) Sim	( ) Não

**Artigo 415: Beta-Interferon treatment does not always slow the progression of axonal injury in multiple sclerosis.**

Parry A, Corkill R, Blamire AM, Palace J, Narayanan S, Arnold D, Styles P, Matthews PM

**P)** Os participantes possuem mais de 18 anos de idade?

(X) Sim	( ) Não

"We studied eleven patients with clinically definite RRMS (6 men and 5 women, **median age 36 years, range 22–40**, Table 1) and seven normal healthy control subjects (4 men, 3 women, median age 30 years, range 24–37)".

**I)** A intervenção utilizada no estudo é creatina? Vale qualquer formato de administração

( ) Sim	(X) Não

"Treatment with **Beta-interferon (BIFN)** has repeatedly been shown to reduce the frequency of relapses and inflammation in patients with relapsing-remitting multiple sclerosis (RRMS)". The study "used magnetic resonance spectroscopy measures to test whether **BIFN** can reverse or arrest progression of axonal injury in patients with MS". Creatine is mentioned as part of a ratio (NAA/creatine ratio) used for measurement, not as an intervention.

**C)** Teve grupo controle?

( ) Sim ( ) Não

**O)** O estudo apresenta valores de marcadores inflamatórios (citocinas e outros marcadores) antes e após as intervenções de creatina?

( ) Sim	( ) Não

**S)** O estudo é um ensaio clínico randomizado e controlado?

( ) Sim	( ) Não

**Artigo 416: Proton MR spectroscopy of cerebellitis.**

Guerrini L, Belli G, Cellerini M, Nencini P, Mascalchi M

**P)** Os participantes possuem mais de 18 anos de idade?

( ) Sim	(X) Não

"Cerebellitis is a disease of **children and adults** in which a selective damage of the cerebellum ensues during or after an infectious disease". The study then refers to "two patients with cerebellitis" without specifying their ages, meaning it cannot be confirmed that all participants were over 18 years of age.

**I)** A intervenção utilizada no estudo é creatina? Vale qualquer formato de administração

( ) Sim	( ) Não

**C)** Teve grupo controle?

( ) Sim ( ) Não

**O)** O estudo apresenta valores de marcadores inflamatórios (citocinas e outros marcadores) antes e após as intervenções de creatina?

( ) Sim	( ) Não

**S)** O estudo é um ensaio clínico randomizado e controlado?

( ) Sim	( ) Não

**Artigo 417: IGF-I/IGFBP-3 equilibrates ratios of pro- to anti-inflammatory cytokines, which are predictors for organ function in severely burned pediatric patients.**

Jeschke MG, Barrow RE, Suzuki F, Rai J, Benjamin D, Herndon DN

**P)** Os participantes possuem mais de 18 anos de idade?

( ) Sim	(X) Não

"Twenty-four severely thermally injured **children** were randomly divided (block randomization 3:1) to receive recombinant human IGF-I in combination with its principle binding protein 3 (IGF-I/BP-3, n = 17) or saline (control, n = 7). Inclusion criteria were **patients under 15 years of age**". Additionally, "The age range for the control group was **2–10 years**, the age range in the treatment group was **2–13 years**".

**I)** A intervenção utilizada no estudo é creatina? Vale qualquer formato de administração

( ) Sim	( ) Não

**C)** Teve grupo controle?

( ) Sim ( ) Não

**O)** O estudo apresenta valores de marcadores inflamatórios (citocinas e outros marcadores) antes e após as intervenções de creatina?

( ) Sim	( ) Não

**S)** O estudo é um ensaio clínico randomizado e controlado?

( ) Sim	( ) Não

**Artigo 418: Hemofiltration and peritoneal dialysis in infection-associated acute renal failure in Vietnam.**

Phu NH, Hien TT, Mai NT, Chau TT, Chuong LV, Loc PP, Winearls C, Farrar J, White N, Day N

**P)** Os participantes possuem mais de 18 anos de idade?

( ) Sim	(X) Não

"Any patient in whom urgent renal-replacement therapy was indicated to treat acute renal failure was considered eligible for the study, except for patients who were pregnant, who were **less than 15 years of age**, or who had previously received renal-replacement therapy of any type during the current illness. Written informed consent was obtained either from the patient or from his or her attendant relative if the patient was comatose or **less than 18 years of age**". Additionally, the age range is stated as "**15–74**".

**I)** A intervenção utilizada no estudo é creatina? Vale qualquer formato de administração

( ) Sim	( ) Não

**C)** Teve grupo controle?

( ) Sim ( ) Não

**O)** O estudo apresenta valores de marcadores inflamatórios (citocinas e outros marcadores) antes e após as intervenções de creatina?

( ) Sim	( ) Não

**S)** O estudo é um ensaio clínico randomizado e controlado?

( ) Sim	( ) Não

**Artigo 419: Complement regulatory protein CD59 involves c-SRC related tyrosine phosphorylation of the creatine transporter in skeletal muscle during sepsis.**

Wang W, Shang LH, Jacobs DO

**P)** Os participantes possuem mais de 18 anos de idade?

( ) Sim	(X) Não

"**Male Sprague-Dawley rats** (250 to 300 g) were randomized to undergo cecal ligation and puncture (CLP) or sham operation". The study was conducted on **rats**, not human subjects.

**I)** A intervenção utilizada no estudo é creatina? Vale qualquer formato de administração

( ) Sim	( ) Não

**C)** Teve grupo controle?

( ) Sim ( ) Não

**O)** O estudo apresenta valores de marcadores inflamatórios (citocinas e outros marcadores) antes e após as intervenções de creatina?

( ) Sim	( ) Não

**S)** O estudo é um ensaio clínico randomizado e controlado?

( ) Sim	( ) Não

**Artigo 420: Experience with a once-daily dosing program of aminoglycosides in critically ill patients.**

Buijk SE, Mouton JW, Gyssens IC, Verbrugh HA, Bruining HA

**P)** Os participantes possuem mais de 18 anos de idade?

(X) Sim	( ) Não

"Age (mean, range) (years) **57 (87–20)**".

**I)** A intervenção utilizada no estudo é creatina? Vale qualquer formato de administração

( ) Sim	(X) Não

"Interventions: A first dose of 7 mg/kg/24 h of **gentamicin or tobramycin** was given to every patient independent of renal function".

**C)** Teve grupo controle?

( ) Sim ( ) Não

**O)** O estudo apresenta valores de marcadores inflamatórios (citocinas e outros marcadores) antes e após as intervenções de creatina?

( ) Sim	( ) Não

**S)** O estudo é um ensaio clínico randomizado e controlado?

( ) Sim	( ) Não

**Artigo 421: Serial 1H-MRS in relapsing-remitting multiple sclerosis: effects of interferon-beta therapy on absolute metabolite concentrations.**

Schubert F, Seifert F, Elster C, Link A, Walzel M, Mientus S, Haas J, Rinneberg H

**P)** Os participantes possuem mais de 18 anos de idade?

(X) Sim	( ) Não

"Ten patients (four female, six male, **mean age 38 years, range 22–58 years**) having clinically definite relapsing-remitting MS were studied."

**I)** A intervenção utilizada no estudo é creatina? Vale qualquer formato de administração

( ) Sim	(X) Não

"a longitudinal 1H-MRS study at 3 T was carried out on ten patients having relapsing-remitting multiple sclerosis (MS) who, after baseline examination, received **interferon-β (IFN) lb**." The study also states that "MR examination was carried out... after first **IFN injection**".

**C)** Teve grupo controle?

( ) Sim ( ) Não

**O)** O estudo apresenta valores de marcadores inflamatórios (citocinas e outros marcadores) antes e após as intervenções de creatina?

( ) Sim	( ) Não

**S)** O estudo é um ensaio clínico randomizado e controlado?

( ) Sim	( ) Não

**Artigo 422: Reduced oxidative phosphorylation and proton efflux suggest reduced capillary blood supply in skeletal muscle of patients with dermatomyositis and polymyositis: a quantitative 31P-magnetic resonance spectroscopy and MRI study.**

Cea G, Bendahan D, Manners D, Hilton-Jones D, Lodi R, Styles P, Taylor DJ

**P)** Os participantes possuem mais de 18 anos de idade?

(X) Sim	( ) Não

"Nine patients with DM (**mean age 6 SD, 57 6 14 years**) and ®ve with PM (**42 6 12 years**) and with age at disease onset 53 6 16 and 38 6 12 years, respectively, were included in the study together with **18 age-matched controls**.".

**I)** A intervenção utilizada no estudo é creatina? Vale qualquer formato de administração

( ) Sim	(X) Não

"Quantitative MRI and phosphorus magnetic resonance spectroscopy (**31P-MRS**) were used to investigate skeletal muscle metabolism in vivo in patients with dermatomyositis (DM) and polymyositis (PM)". The study measured creatine (Cr) as a metabolite, but **creatine was not the intervention** administered in the study.

**C)** Teve grupo controle?

( ) Sim ( ) Não

**O)** O estudo apresenta valores de marcadores inflamatórios (citocinas e outros marcadores) antes e após as intervenções de creatina?

( ) Sim	( ) Não

**S)** O estudo é um ensaio clínico randomizado e controlado?

( ) Sim	( ) Não

**Artigo 423: Serum cystatin C reliably detects renal dysfunction in patients with various renal diseases.**

Kazama JJ, Kutsuwada K, Ataka K, Maruyama H, Gejyo F

**P)** Os participantes possuem mais de 18 anos de idade?

( ) Sim	(X) Não

"Two hundred and twelve patients (**47 ± 18 years old, range 13– 85**; 104 males, 108 females) who underwent renal biopsy at Niigata University Hospital between January 1994 and March 1999 enrolled in the study". Since the age range includes individuals as young as 13 years old, not all participants possessed more than 18 years of age.

**I)** A intervenção utilizada no estudo é creatina? Vale qualquer formato de administração

( ) Sim	( ) Não

**C)** Teve grupo controle?

( ) Sim ( ) Não

**O)** O estudo apresenta valores de marcadores inflamatórios (citocinas e outros marcadores) antes e após as intervenções de creatina?

( ) Sim	( ) Não

**S)** O estudo é um ensaio clínico randomizado e controlado?

( ) Sim	( ) Não

**Artigo 424: Role of skeletal muscle Na+-K+ ATPase activity in increased lactate production in sub-acute sepsis.**

McCarter FD, Nierman SR, James JH, Wang L, King JK, Friend LA, Fischer JE

**P)** Os participantes possuem mais de 18 anos de idade?

( ) Sim	(X) Não

The provided excerpt for Article 424 does not explicitly state the type of participants. However, other studies by authors of this paper (e.g., Wang, Jacobs, James) on similar topics within the provided sources indicate the use of rats. For example, Article 419 states, "**Male Sprague-Dawley rats** (250 to 300 g) were randomized to undergo cecal ligation and puncture (CLP) or sham operation". Similarly, Article 425 mentions, "**Male Sprague-Dawley rats** (Harlan Sprague Dawley, Indianapolis, IN), weighing 250–300 g", and Article 453 specifies, "**Thirty-eight adult male Wistar rats** (110-121 g)". Therefore, it is highly probable that this study was also conducted on rats, which are not human subjects over 18 years of age.

**I)** A intervenção utilizada no estudo é creatina? Vale qualquer formato de administração

( ) Sim	( ) Não

**C)** Teve grupo controle?

( ) Sim ( ) Não

**O)** O estudo apresenta valores de marcadores inflamatórios (citocinas e outros marcadores) antes e após as intervenções de creatina?

( ) Sim	( ) Não

**S)** O estudo é um ensaio clínico randomizado e controlado?

( ) Sim	( ) Não

**Artigo 425: Cr supplementation decreases tyrosine phosphorylation of the CreaT in skeletal muscle during sepsis.**

Wang W, Jobst MA, Bell B, Zhao CR, Shang LH, Jacobs DO

**P)** Os participantes possuem mais de 18 anos de idade?

( ) Sim	(X) Não

"**Male Sprague-Dawley rats** (Harlan Sprague Dawley, Indianapolis, IN), weighing 250–300 g, were acclimatized to a 12-hour light/dark cycle for 3 days and housed in a temperature-controlled room (22°C) with 50% to 70% humidity before any experiments were initiated.".

**I)** A intervenção utilizada no estudo é creatina? Vale qualquer formato de administração

( ) Sim	( ) Não

**C)** Teve grupo controle?

( ) Sim ( ) Não

**O)** O estudo apresenta valores de marcadores inflamatórios (citocinas e outros marcadores) antes e após as intervenções de creatina?

( ) Sim	( ) Não

**S)** O estudo é um ensaio clínico randomizado e controlado?

( ) Sim	( ) Não

**Artigo 426: Inflammation in acute coronary syndromes**

Maseri A

**P)** Os participantes possuem mais de 18 anos de idade?

( ) Sim	(X) Não

The source, "Inflammation in acute coronary syndromes," is a **REVIEW** article. Review articles synthesize existing scientific literature and **do not involve direct participants** in the way original research studies do. Therefore, the concept of participants having a specific age does not apply to this type of publication.

**I)** A intervenção utilizada no estudo é creatina? Vale qualquer formato de administração

( ) Sim	( ) Não

**C)** Teve grupo controle?

( ) Sim ( ) Não

**O)** O estudo apresenta valores de marcadores inflamatórios (citocinas e outros marcadores) antes e após as intervenções de creatina?

( ) Sim	( ) Não

**S)** O estudo é um ensaio clínico randomizado e controlado?

( ) Sim	( ) Não

**Artigo 427: Induction of peritoneal sepsis increases the susceptibility of isolated hearts to a calcium paradox-mediated injury.**

Omachi A, Sharma AC, Alden KJ, Sam AD, Ferguson JL

**P)** Os participantes possuem mais de 18 anos de idade?

( ) Sim	(X) Não

"Male Sprague-Dawley **rats** were made septic using 200 mg of cecal material (obtained from a donor rat) suspended in 5 mL of 5% dextrose in sterile water (1) D5 W/kg.". The study was conducted on animals (rats), not human subjects over 18 years of age.

**I)** A intervenção utilizada no estudo é creatina? Vale qualquer formato de administração

( ) Sim	( ) Não

**C)** Teve grupo controle?

( ) Sim ( ) Não

**O)** O estudo apresenta valores de marcadores inflamatórios (citocinas e outros marcadores) antes e após as intervenções de creatina?

( ) Sim	( ) Não

**S)** O estudo é um ensaio clínico randomizado e controlado?

( ) Sim	( ) Não

**Artigo 428: Effect of creatine monohydrate on cardiac function in a** rat **model of endotoxemia.**

Vona-Davis L, Wearden PD, Karne NH, Hill RC

**P)** Os participantes possuem mais de 18 anos de idade?

( ) Sim	(X) Não

"This study was undertaken to investigate whether creatine (Cr) administered during perfusion could enhance myocardial protection and improve recovery of cardiac function in a **rat model of endotoxemia**." Additionally, "Acute endotoxemia was induced in **rats** by a bolus injection of Escherichia coli endotoxin (LPS: 4 mg/kg, ip) while control **rats** were injected with an equal volume of 0.9% normal saline." The study was conducted on animals (rats), not human subjects over 18 years of age.

**I)** A intervenção utilizada no estudo é creatina? Vale qualquer formato de administração

( ) Sim	( ) Não

**C)** Teve grupo controle?

( ) Sim ( ) Não

**O)** O estudo apresenta valores de marcadores inflamatórios (citocinas e outros marcadores) antes e após as intervenções de creatina?

( ) Sim	( ) Não

**S)** O estudo é um ensaio clínico randomizado e controlado?

( ) Sim	( ) Não

**Artigo 429: Hepatitis C and cognitive impairment in a** cohort **of patients with mild liver disease.**

Forton DM, Thomas HC, Murphy CA, Allsop JM, Foster GR, Main J, Wesnes KA, Taylor-Robinson SD

**P)** Os participantes possuem mais de 18 anos de idade?

(X) Sim	( ) Não

"The mean age (range) was **44.5 (28-69) years**" for the HCV-infected patient group. For the subgroup that underwent 1H MRS, "The mean age (range) was **42.1 (28-57) years**". The HCV-cleared group had a "mean age (range) was **42.3 (32-60) years**", and the healthy control group had a "mean (range) age was **42 (30-54) years**". All age ranges indicate that all participants were older than 18 years of age.

**I)** A intervenção utilizada no estudo é creatina? Vale qualquer formato de administração

( ) Sim	(X) Não

The study aimed to determine "whether HCV infection has a direct effect on the central nervous system, resulting in cognitive and cerebral metabolite abnormalities". It reports on measurements, such as "The choline/creatine ratio was elevated in the basal ganglia and white matter in this group", and notes that "The creatine resonance reflects cellular energy status and is a largely stable entity". The study describes **measuring** creatine-related metabolites rather than administering creatine as an intervention.

**C)** Teve grupo controle?

( ) Sim ( ) Não

**O)** O estudo apresenta valores de marcadores inflamatórios (citocinas e outros marcadores) antes e após as intervenções de creatina?

( ) Sim	( ) Não

**S)** O estudo é um ensaio clínico randomizado e controlado?

( ) Sim	( ) Não

**Artigo 430: Dopamine dose and renal damage.**

Sánchez Garcia M, Alvarez Gonzáez M, Velo Plaza M

**P)** Os participantes possuem mais de 18 anos de idade?

( ) Sim	(X) Não

"Any patient... who were **less than 15 years of age**, or who had previously received renal-replacement therapy of any type during the current illness." and "Written informed consent was obtained either from the patient or from his or her attendant relative if the patient was comatose or **less than 18 years of age**.". Additionally, the age range for participants is "15–74", indicating that some participants were under 18 years old.

**I)** A intervenção utilizada no estudo é creatina? Vale qualquer formato de administração

( ) Sim	( ) Não

**C)** Teve grupo controle?

( ) Sim ( ) Não

**O)** O estudo apresenta valores de marcadores inflamatórios (citocinas e outros marcadores) antes e após as intervenções de creatina?

( ) Sim	( ) Não

**S)** O estudo é um ensaio clínico randomizado e controlado?

( ) Sim	( ) Não

**Artigo 431: Therapies for improving muscle function in neuromuscular disorders.**

Lynch GS

**P)** Os participantes possuem mais de 18 anos de idade?

( ) Sim	(X) Não

"This review examines recent pharmacologic or nutrition interventions for ameliorating wasting and improving muscle function in neuromuscular disorders". As indicated by the abstract, this article is a **review** and, as such, does not involve direct participants or experimental subjects in the way an original research study would. It synthesizes existing literature, which may include studies on individuals of various ages, including "boys with DMD", but the review itself does not have participants.

**I)** A intervenção utilizada no estudo é creatina? Vale qualquer formato de administração

( ) Sim	( ) Não

**C)** Teve grupo controle?

( ) Sim ( ) Não

**O)** O estudo apresenta valores de marcadores inflamatórios (citocinas e outros marcadores) antes e após as intervenções de creatina?

( ) Sim	( ) Não

**S)** O estudo é um ensaio clínico randomizado e controlado?

( ) Sim	( ) Não

**Artigo 432: Role of ischemia and of hypoxia-inducible genes in arteriogenesis after femoral artery occlusion in the** rabbit**.**

Deindl E, Buschmann I, Hoefer IE, Podzuweit T, Boengler K, Vogel S, van Royen N, Fernandez B, Schaper W

**P)** Os participantes possuem mais de 18 anos de idade?

( ) Sim	(X) Não

"The present study was performed with the permission of the State of Hessen, Regierungspraesidium Darmstadt, according to section 8 of the German Law for the Protection of Animals. It conforms to the Guide for the Care and Use of Laboratory Animals published by the US National Institutes of Health (NIH publication No. 85-23, revised 1996)." and "Right femoral arteries of **rabbits** (18 rabbits; 8 male, 10 female; body weight 3.7±0.5 kg; **age 3 to 4 months**) were ligated with 2 ligatures ~1.5 cm apart (but not transected), and the animals were randomly assigned to receive either monocyte chemotactic protein (MCP)-1 (3 mg at a rate of 10µL/h; Peprotech), VEGF165 (100mg at a rate of 10 µL/h; Peprotech), or solvent locally via osmotic minipump (2 ML-1, Alza Corp) for 7 days.". The study was conducted on animals (rabbits), not human subjects over 18 years of age.

**I)** A intervenção utilizada no estudo é creatina? Vale qualquer formato de administração

( ) Sim	( ) Não

**C)** Teve grupo controle?

( ) Sim ( ) Não

**O)** O estudo apresenta valores de marcadores inflamatórios (citocinas e outros marcadores) antes e após as intervenções de creatina?

( ) Sim	( ) Não

**S)** O estudo é um ensaio clínico randomizado e controlado?

( ) Sim	( ) Não

**Artigo 433: Fenoldopam: renal and splanchnic effects in patients undergoing coronary artery bypass grafting.**

Halpenny M, Lakshmi S, O'Donnell A, O'Callaghan-Enright S, Shorten GD

**P)** Os participantes possuem mais de 18 anos de idade?

(X) Sim	( ) Não

"Inclusion criteria were: **age < 70 years**". Furthermore, the demographic data shows ages ranging from 42-69 years for the placebo group and 41-70 years for the fenoldopam group, indicating all participants were above 18 years of age.

**I)** A intervenção utilizada no estudo é creatina? Vale qualquer formato de administração

( ) Sim	(X) Não

The intervention used in this study was "fenoldopam, a selective dopamine receptor agonist". The study aimed to test its "renoprotective effects and increases mesenteric blood flow".

**C)** Teve grupo controle?

( ) Sim ( ) Não

**O)** O estudo apresenta valores de marcadores inflamatórios (citocinas e outros marcadores) antes e após as intervenções de creatina?

( ) Sim	( ) Não

**S)** O estudo é um ensaio clínico randomizado e controlado?

( ) Sim	( ) Não

**Artigo 434: Inhibition of tumor necrosis factor-alpha improves postischemic recovery of hypertrophied hearts.**

Stamm C, Friehs I, Cowan DB, Moran AM, Cao-Danh H, Duebener LF, del Nido PJ, McGowan FX Jr

**P)** Os participantes possuem mais de 18 anos de idade?

( ) Sim	(X) Não

"Methods and Results—**Neonatal rabbits** underwent aortic banding to induce LV hypertrophy. Myocardial TNF-alpha protein expression increased progressively with LV hypertrophy.". The study was conducted on animals (neonatal rabbits), not human subjects over 18 years of age.

**I)** A intervenção utilizada no estudo é creatina? Vale qualquer formato de administração

( ) Sim	( ) Não

**C)** Teve grupo controle?

( ) Sim ( ) Não

**O)** O estudo apresenta valores de marcadores inflamatórios (citocinas e outros marcadores) antes e após as intervenções de creatina?

( ) Sim	( ) Não

**S)** O estudo é um ensaio clínico randomizado e controlado?

( ) Sim	( ) Não

**Artigo 435: Chronic uremia induces permeability changes, increased nitric oxide synthase expression, and structural modifications in the peritoneum.**

Combet S, Ferrier ML, Landschoot MV, Stoenoiu M, Moulin P, Miyata T, Lameire N, Devuyst O

**P)** Os participantes possuem mais de 18 anos de idade?

( ) Sim	(X) Não

"Adult (8 wk) male **Sprague-Dawley rats** (Iffa Credo, Brussels, Belgium) weighing 300 ± 3 g were randomly assigned to uremic and sham-operated groups." O estudo foi conduzido em animais (ratos), não em sujeitos humanos com mais de 18 anos de idade.

**I)** A intervenção utilizada no estudo é creatina? Vale qualquer formato de administração

( ) Sim	( ) Não

**C)** Teve grupo controle?

( ) Sim ( ) Não

**O)** O estudo apresenta valores de marcadores inflamatórios (citocinas e outros marcadores) antes e após as intervenções de creatina?

( ) Sim	( ) Não

**S)** O estudo é um ensaio clínico randomizado e controlado?

( ) Sim	( ) Não

**Artigo 436: Genetically modified bone marrow continuously supplies anti-inflammatory** cells **and suppresses renal injury in** mouse **Goodpasture syndrome.**

Yokoo T, Ohashi T, Utsunomiya Y, Shen JS, Hisada Y, Eto Y, Kawamura T, Hosoya T

**P)** Os participantes possuem mais de 18 anos de idade?

( ) Sim	(X) Não

"Seven-week-old-female DBA/2j **mice** were purchased from Nippon Crea (Tokyo, Japan)". This study was conducted on animals (mice) that were 7 weeks old, not human participants over 18 years of age.

**I)** A intervenção utilizada no estudo é creatina? Vale qualquer formato de administração

( ) Sim	( ) Não

**C)** Teve grupo controle?

( ) Sim ( ) Não

**O)** O estudo apresenta valores de marcadores inflamatórios (citocinas e outros marcadores) antes e após as intervenções de creatina?

( ) Sim	( ) Não

**S)** O estudo é um ensaio clínico randomizado e controlado?

( ) Sim	( ) Não

**Artigo 437: Proton MR spectroscopy in multiple sclerosis.**

Arnold DL, De Stefano N, Narayanan S, Matthews PM

**P)** Os participantes possuem mais de 18 anos de idade?

(X) Sim	( ) Não

"This review is divided into three major sections: ... 2) review of MRS and its application to MS lesions, NAWM, gray matter, and spinal cord, and 3) role of MRS in clinical trials.". The review summarizes findings from multiple studies where participants were over 18, such as one study on relapsing-remitting MS patients (**mean age 38 years, range 22-58 years**) and age-matched healthy controls, and another study with MS patients (mean age 39, range 26–48 years) and healthy subjects (mean age 32, range 21–48 years).

**I)** A intervenção utilizada no estudo é creatina? Vale qualquer formato de administração

( ) Sim	(X) Não "This is a review article that discusses the application of proton MR spectroscopy to multiple sclerosis. The interventions discussed in the context of clinical trials for multiple sclerosis in this review predominantly include "immunomodulatory therapies, including glatiramer acetate (GA), interferon (IFN)-β, and natalizumab". Creatine (Cr) is consistently referred to as a **metabolite whose levels are measured by MRS** in the brain of MS patients and controls, not as a therapeutic intervention for multiple sclerosis within the studies reviewed." While the review does cite studies where creatine *supplementation* was used, these are in the context of *other* neurological or muscular disorders (e.g., mitochondrial diseases, ALS, Huntington's disease, myotonic dystrophy, dermatomyositis).

**C)** Teve grupo controle?

( ) Sim ( ) Não

**O)** O estudo apresenta valores de marcadores inflamatórios (citocinas e outros marcadores) antes e após as intervenções de creatina?

( ) Sim	( ) Não

**S)** O estudo é um ensaio clínico randomizado e controlado?

( ) Sim	( ) Não

**Artigo 438: Possible role of uncoupling protein in regulation of myocardial energy metabolism in aortic regurgitation model** rats**.**

Noma T, Nishiyama A, Mizushige K, Murakami K, Tsuji T, Kohno M, Rahman M, Fukui T, Abe Y, Kimura S

**P)** Os participantes possuem mais de 18 anos de idade?

( ) Sim	(X) Não

"We focused on serial changes in the cardiac performance and adenosine triphosphate (ATP) biosynthesis in relation to the gene expression of uncoupling protein 2 (UCP-2), an inner mitochondrial membrane proton transporter, in a **rat model of aortic regurgitation (AR)** that gradually developed heart failure.". The study was conducted on animals (rats), not human subjects over 18 years of age.

**I)** A intervenção utilizada no estudo é creatina? Vale qualquer formato de administração

( ) Sim	( ) Não

**C)** Teve grupo controle?

( ) Sim ( ) Não

**O)** O estudo apresenta valores de marcadores inflamatórios (citocinas e outros marcadores) antes e após as intervenções de creatina?

( ) Sim	( ) Não

**S)** O estudo é um ensaio clínico randomizado e controlado?

( ) Sim	( ) Não

**Artigo 439: Tubular cell senescence and expression of TGF-beta1 and p21(WAF1/CIP1) in tubulointerstitial fibrosis of aging** rats**.**

Ding G, Franki N, Kapasi AA, Reddy K, Gibbons N, Singhal PC

**P)** Os participantes possuem mais de 18 anos de idade?

( ) Sim	(X) Não

"Sprague–Dawley **rats** at the ages of 3, 12, and 24 months were used for this study.". This indicates the study was conducted on animals (rats), not human participants over 18 years of age.

**I)** A intervenção utilizada no estudo é creatina? Vale qualquer formato de administração

( ) Sim	( ) Não

**C)** Teve grupo controle?

( ) Sim ( ) Não

**O)** O estudo apresenta valores de marcadores inflamatórios (citocinas e outros marcadores) antes e após as intervenções de creatina?

( ) Sim	( ) Não

**S)** O estudo é um ensaio clínico randomizado e controlado?

( ) Sim	( ) Não

**Artigo 440: [Septic-toxic shock due to rhabdomyolysis in a patient treated with fenofibrate].**

Duda-Król W, Jórasz I, Dabrowska M, Polubiec A, Kusz-Rynkun A

**P)** Os participantes possuem mais de 18 anos de idade?

( ) Sim	( ) Não [*Excluir*]

**I)** A intervenção utilizada no estudo é creatina? Vale qualquer formato de administração

( ) Sim	(	) Não [*Excluir*]

**C)** Teve grupo controle?

(	) Sim ( ) Não [*Excluir*]

**O)** O estudo apresenta valores de marcadores inflamatórios (citocinas e outros marcadores) antes e após as intervenções de creatina?

( ) Sim	(	) Não [*Excluir*]

**S)** O estudo é um ensaio clínico randomizado e controlado?

(	) Sim	( ) Não

**Artigo 441: Effect of dopexamine on intestinal tissue concentrations of high-energy phosphates and intestinal release of purine compounds in endotoxemic** rats**.**

Schmidt H, Weigand MA, Schmidt W, Plaschke K, Martin E, Bardenheuer HJ

**P)** Os participantes possuem mais de 18 anos de idade?

( ) Sim	(X) Não

"Subjects: Twenty-one male Wistar **rats**.". The study was conducted on animals (rats), not human participants over 18 years of age.

**I)** A intervenção utilizada no estudo é creatina? Vale qualquer formato de administração

( ) Sim	( ) Não

**C)** Teve grupo controle?

( ) Sim ( ) Não

**O)** O estudo apresenta valores de marcadores inflamatórios (citocinas e outros marcadores) antes e após as intervenções de creatina?

( ) Sim	( ) Não

**S)** O estudo é um ensaio clínico randomizado e controlado?

( ) Sim	( ) Não

**Artigo 442: Beneficial effects of sanguisorbae radix in renal dysfunction caused by endotoxin in vivo.**

Chen CP, Yokozawa T, Kitani K

**P)** Os participantes possuem mais de 18 anos de idade?

( ) Sim	(X) Não

O estudo utilizou **animais**, especificamente "Male LWH Wistar rats of 125-130 g" e "Male adult dogs of 12.5-20.0 kg". Não há menção de participantes humanos ou sua idade nas fontes fornecidas para este artigo.

**I)** A intervenção utilizada no estudo é creatina? Vale qualquer formato de administração

( ) Sim	( ) Não

**C)** Teve grupo controle?

( ) Sim ( ) Não

**O)** O estudo apresenta valores de marcadores inflamatórios (citocinas e outros marcadores) antes e após as intervenções de creatina?

( ) Sim	( ) Não

**S)** O estudo é um ensaio clínico randomizado e controlado?

( ) Sim	( ) Não

**Artigo 443: Role of TNF-alpha in myocardial dysfunction after hemorrhagic shock and lower-torso ischemia.**

Shahani R, Marshall JG, Rubin BB, Li RK, Walker PM, Lindsay TF

**P)** Os participantes possuem mais de 18 anos de idade?

( ) Sim	(X) Não

"In a model of RAAA repair designed in our laboratory, **adult male Sprague-Dawley rats** weighing 350–400 g (Charles River, Wilmington, MA) were allowed to acclimatize for 5 days with water and rat chow ad libitum.". This indicates that the study was conducted on **rats**, not human participants over 18 years of age.

**I)** A intervenção utilizada no estudo é creatina? Vale qualquer formato de administração

( ) Sim	( ) Não

**C)** Teve grupo controle?

( ) Sim ( ) Não

**O)** O estudo apresenta valores de marcadores inflamatórios (citocinas e outros marcadores) antes e após as intervenções de creatina?

( ) Sim	( ) Não

**S)** O estudo é um ensaio clínico randomizado e controlado?

( ) Sim	( ) Não

**Artigo 444: Effect of genetic disruption of poly (ADP-ribose) synthetase on delayed production of inflammatory mediators and delayed necrosis during myocardial ischemia-reperfusion injury.**

Yang Z, Zingarelli B, Szabó C

**P)** Os participantes possuem mais de 18 anos de idade?

( ) Sim	(X) Não

"Serum creatinine phosphate kinase, an enzyme contained in viable myocytes, is released into the bloodstream during myocardial injury as measured as an index of myocyte necrosis in **PARS−/− mice and PARS+/+ mice** subjected to myocardial ischemia-reperfusion." and "Serum levels of TNF-alpha and IL-10, as measured by specific ELISA kits in **PARS+/+ and PARS−/− mice** subjected to 30 min of occlusion of LAD and 24 h of reperfusion". This indicates the study was conducted on animals (mice), not human participants over 18 years of age.

**I)** A intervenção utilizada no estudo é creatina? Vale qualquer formato de administração

( ) Sim	( ) Não

**C)** Teve grupo controle?

( ) Sim ( ) Não

**O)** O estudo apresenta valores de marcadores inflamatórios (citocinas e outros marcadores) antes e após as intervenções de creatina?

( ) Sim	( ) Não

**S)** O estudo é um ensaio clínico randomizado e controlado?

( ) Sim	( ) Não

**Artigo 445: Quasi-total-body exposure to an oxygen-ozone mixture in a sauna cabin.**

Bocci V, Borrelli E, Valacchi G, Luzzi E

**P)** Os participantes possuem mais de 18 anos de idade?

(X) Sim	( ) Não

"Seven healthy male volunteers... **They were aged between 32 and 48 years [mean 41 (5)]**."

**I)** A intervenção utilizada no estudo é creatina? Vale qualquer formato de administração

( ) Sim	(X) Não

"We have investigated the effects of quasi-total-body exposure of healthy volunteers to either an **oxygen-ozone mixture (O2-O3) or to oxygen (O2) alone** during a short period in a sauna cabin." "The flow of gas through the cabin (either a gas mixture composed of about 97% O2 and 3% O3, or pure O2) is 1 litre/min". While "Creatine (mg/dl)" levels were measured, it was a monitored parameter, not the intervention.

**C)** Teve grupo controle?

( ) Sim ( ) Não

**O)** O estudo apresenta valores de marcadores inflamatórios (citocinas e outros marcadores) antes e após as intervenções de creatina?

( ) Sim	( ) Não

**S)** O estudo é um ensaio clínico randomizado e controlado?

( ) Sim	( ) Não

**Artigo 446: Glycine improves survival after hemorrhagic shock in the** rat**.**

Zhong Z, Enomoto N, Connor HD, Moss N, Mason RP, Thurman RG

**P)** Os participantes possuem mais de 18 anos de idade?

( ) Sim	(X) Não

The study was conducted using **animals**, specifically "Female Sprague-Dawley rats". The article title itself indicates the study was "in the rat".

**I)** A intervenção utilizada no estudo é creatina? Vale qualquer formato de administração

( ) Sim	( ) Não

**C)** Teve grupo controle?

( ) Sim ( ) Não

**O)** O estudo apresenta valores de marcadores inflamatórios (citocinas e outros marcadores) antes e após as intervenções de creatina?

( ) Sim	( ) Não

**S)** O estudo é um ensaio clínico randomizado e controlado?

( ) Sim	( ) Não

**Artigo 447: Effects of combined parenteral vitamins C and E administration on the severity of anaemia, hepatic and renal damage in Trypanosoma brucei brucei infected** rabbits**.**

Umar IA, Wuro-Chekke AU, Gidado A, Igbokwe IO

**P)** Os participantes possuem mais de 18 anos de idade?

( ) Sim	(X) Não

"Twenty healthy male **adult rabbits** (mixed breed) weighing 1.1± 0.3 kg were purchased from local farmers and maintained in laboratory cages.". This indicates that the study was conducted on **rabbits**, not human participants over 18 years of age.

**I)** A intervenção utilizada no estudo é creatina? Vale qualquer formato de administração

( ) Sim	( ) Não

**C)** Teve grupo controle?

( ) Sim ( ) Não

**O)** O estudo apresenta valores de marcadores inflamatórios (citocinas e outros marcadores) antes e após as intervenções de creatina?

( ) Sim	( ) Não

**S)** O estudo é um ensaio clínico randomizado e controlado?

( ) Sim	( ) Não

**Artigo 448: Serum amyloid A (SAA): a concise review of biology, assay methods and clinical usefulness.**

Yamada T

**P)** Os participantes possuem mais de 18 anos de idade?

( ) Sim	(X) Não

"Serum Amyloid A (SAA): a Concise Review of Biology, Assay Methods and Clinical Usefulness". **This article is a review, not an original research study involving participants.** A review article synthesizes and discusses existing literature, rather than conducting new experiments with its own set of human subjects or animals. Therefore, the concept of "participants" in the context of an intervention study does not apply to this type of publication.

**I)** A intervenção utilizada no estudo é creatina? Vale qualquer formato de administração

( ) Sim	( ) Não

**C)** Teve grupo controle?

( ) Sim ( ) Não

**O)** O estudo apresenta valores de marcadores inflamatórios (citocinas e outros marcadores) antes e após as intervenções de creatina?

( ) Sim	( ) Não

**S)** O estudo é um ensaio clínico randomizado e controlado?

( ) Sim	( ) Não

**Artigo 449: Beneficial effects of L-canavanine, a selective inhibitor of inducible nitric oxide synthase, on lactate metabolism and muscle high energy phosphates during endotoxic shock in** rats**.**

Levy B, Valtier M, de Chillou C, Bollaert PE, Cane D, Mallie JP

**P)** Os participantes possuem mais de 18 anos de idade?

( ) Sim	(X) Não

"Experiments were conducted in adherence to the 'Guiding Principles for Research Involving Animals and Human Beings' of the American Physiological Society. **Wistar rats (300–320 g)** fasted overnight were anesthetized...". Furthermore, the abstract of Article 449 (source) explicitly mentions "...**rats** received an intravenous infusion of 15 mg/kg of endotoxin", confirming the use of animal subjects.

**I)** A intervenção utilizada no estudo é creatina? Vale qualquer formato de administração

( ) Sim	( ) Não

**C)** Teve grupo controle?

( ) Sim ( ) Não

**O)** O estudo apresenta valores de marcadores inflamatórios (citocinas e outros marcadores) antes e após as intervenções de creatina?

( ) Sim	( ) Não

**S)** O estudo é um ensaio clínico randomizado e controlado?

( ) Sim	( ) Não

**Artigo 450: Interleukin-6 localization and the prognosis of IgA nephropathy.**

Taniguchi Y, Yorioka N, Kumagai J, Katsutani M, Kuratsune M, Amimoto D, Yamakido M

**P)** Os participantes possuem mais de 18 anos de idade?

( ) Sim	(X) Não

"The subjects were 90 patients with IgA nephropathy (42 males and 48 females with a median age of 32.7 ± 13.8 years)." and "The patients were 42 males and 48 females, **aged from 9 to 68 years** (mean ± SD, 32.7 ± 13.8 years)." This indicates that some participants were under 18 years of age.

**I)** A intervenção utilizada no estudo é creatina? Vale qualquer formato de administração

( ) Sim	( ) Não

**C)** Teve grupo controle?

( ) Sim ( ) Não

**O)** O estudo apresenta valores de marcadores inflamatórios (citocinas e outros marcadores) antes e após as intervenções de creatina?

( ) Sim	( ) Não

**S)** O estudo é um ensaio clínico randomizado e controlado?

( ) Sim	( ) Não

**Artigo 451: The beneficial effect of phosphocreatine accumulation in the creatine kinase** transgenic mouse **liver in endotoxin-induced hepatic cell death.**

Kanazawa A, Tanaka A, Iwata S, Satoh S, Hatano E, Shinohara H, Kitai T, Tsunekawa S, Ikai I, Yamamoto M, Takahashi R, Chance B, Yamaoka Y

**P)** Os participantes possuem mais de 18 anos de idade?

( ) Sim	(X) Não

"Creatine kinase transgenic mice were fed with normal rodent chow or chow containing 10% creatine for 5 days". Additionally, the section "Experimental animals" states: "Transgenic mice expressing the brain form of creatine kinase (CK-BB) in their livers were produced from the B6D2 line mice".

**I)** A intervenção utilizada no estudo é creatina? Vale qualquer formato de administração

( ) Sim	( ) Não

**C)** Teve grupo controle?

( ) Sim ( ) Não

**O)** O estudo apresenta valores de marcadores inflamatórios (citocinas e outros marcadores) antes e após as intervenções de creatina?

( ) Sim	( ) Não

**S)** O estudo é um ensaio clínico randomizado e controlado?

( ) Sim	( ) Não

**Artigo 452: Proton MR spectroscopic findings correspond to neuropsychological function in traumatic brain injury.**

Friedman SD, Brooks WM, Jung RE, Hart BL, Yeo RA

**P)** Os participantes possuem mais de 18 anos de idade?

(X) Sim	( ) Não

"Twelve patients (11 men and one woman, aged 34 6 12 years [range, **18 to 49 years**]..." and "Fourteen control subjects... aged 31 6 11 years [range, **19 to 52 years**]) were also included in the study".

**I)** A intervenção utilizada no estudo é creatina? Vale qualquer formato de administração

( ) Sim	(X) Não

The study aimed "to investigate whether proton MR spectroscopic measures of diffuse cellular integrity were related to neuropsychological dysfunction after TBI". **Creatine was measured** as one of the neurometabolites, alongside N-acetylaspartate (NAA) and choline, using proton MR spectroscopy. There is no mention of creatine being administered as an intervention in any format.

**C)** Teve grupo controle?

( ) Sim ( ) Não

**O)** O estudo apresenta valores de marcadores inflamatórios (citocinas e outros marcadores) antes e após as intervenções de creatina?

( ) Sim	( ) Não

**S)** O estudo é um ensaio clínico randomizado e controlado?

( ) Sim	( ) Não

**Artigo 453: Skeletal muscle phosphocreatine depletion depresses myocellular energy status during sepsis.**

Lara TM, Wong MS, Rounds J, Robinson MK, Wilmore DW, Jacobs DO

**P)** Os participantes possuem mais de 18 anos de idade?

( ) Sim	(X) Não

"Materials: **Thirty-eight adult male Wistar rats** (110-121 g)".

**I)** A intervenção utilizada no estudo é creatina? Vale qualquer formato de administração

( ) Sim	( ) Não

**C)** Teve grupo controle?

( ) Sim ( ) Não

**O)** O estudo apresenta valores de marcadores inflamatórios (citocinas e outros marcadores) antes e após as intervenções de creatina?

( ) Sim	( ) Não

**S)** O estudo é um ensaio clínico randomizado e controlado?

( ) Sim	( ) Não

**Artigo 454: Aminoguanidine reduces glomerular inducible nitric oxide synthase (iNOS) and transforming growth factor-beta 1 (TGF-beta1) mRNA expression and diminishes glomerulosclerosis in NZB/W F1** mice**.**

Yang CW, Yu CC, Ko YC, Huang CC

**P)** Os participantes possuem mais de 18 anos de idade?

( ) Sim	(X) Não

"**Female NZB/W F1 mice** (n¼ 8) were treated with aminoguanidine...starting at age 2 months before the onset of glomerulonephritis". Additionally, "Female NZB/W F1 mice, bred in the National Taiwan University Animal Centre, were treated with aminoguanidine (1 g/l) (n¼ 8)...".

**I)** A intervenção utilizada no estudo é creatina? Vale qualquer formato de administração

( ) Sim	( ) Não

**C)** Teve grupo controle?

( ) Sim ( ) Não

**O)** O estudo apresenta valores de marcadores inflamatórios (citocinas e outros marcadores) antes e após as intervenções de creatina?

( ) Sim	( ) Não

**S)** O estudo é um ensaio clínico randomizado e controlado?

( ) Sim	( ) Não

**Artigo 455: Growth requirements and nitrogen metabolism of Malassezia furfur.**

Mayser P, Imkampe A, Winkeler M, Papavassilis C

**P)** Os participantes possuem mais de 18 anos de idade?

( ) Sim	(X) Não

"Investigations on the nutrient requirements of **Malassezia yeasts** led to the preparation of a new minimal medium...". Additionally, "Lipophilic **yeasts of the genus Malassezia** have been known to be components of the microflora of human skin and of many warm-blooded animals for more than 100 years...". The "Materials and methods" section further specifies "Organisms The **strains tested are listed in Table 1**", confirming the subjects are microorganisms, not human participants.

**I)** A intervenção utilizada no estudo é creatina? Vale qualquer formato de administração

( ) Sim	( ) Não

**C)** Teve grupo controle?

( ) Sim ( ) Não

**O)** O estudo apresenta valores de marcadores inflamatórios (citocinas e outros marcadores) antes e após as intervenções de creatina?

( ) Sim	( ) Não

**S)** O estudo é um ensaio clínico randomizado e controlado?

( ) Sim	( ) Não

**Artigo 456: Brain lactate and N-acetylaspartate in pediatric AIDS encephalopathy.**

Pavlakis SG, Lu D, Frank Y, Wiznia A, Eidelberg D, Barnett T, Hyman RA

**P)** Os participantes possuem mais de 18 anos de idade?

( ) Sim	(X) Não

"Sixty-three children (45 with AIDS and 18 control subjects), **age 2 weeks through 17 years**, underwent MR spectroscopy as part of a prior study (2)".

**I)** A intervenção utilizada no estudo é creatina? Vale qualquer formato de administração

( ) Sim	( ) Não

**C)** Teve grupo controle?

( ) Sim ( ) Não

**O)** O estudo apresenta valores de marcadores inflamatórios (citocinas e outros marcadores) antes e após as intervenções de creatina?

( ) Sim	( ) Não

**S)** O estudo é um ensaio clínico randomizado e controlado?

( ) Sim	( ) Não

**Artigo 457: The pharmacokinetics of intravenous ciprofloxacin 400 mg 12 hourly in patients with severe sepsis: the effect of renal function and intra-abdominal disease.**

Jones EM, McMullin CM, Hedges AJ, Lovering AM, White LO, Reeves DS, MacGowan AP

**P)** Os participantes possuem mais de 18 anos de idade?

(X) Sim	( ) Não

"The mean age was 60 years, range **29–76 years** (Table)."

**I)** A intervenção utilizada no estudo é creatina? Vale qualquer formato de administração

( ) Sim	(X) Não

The study investigated the pharmacokinetics of "**iv ciprofloxacin 400 mg bd**". There is no mention of creatine being used as an intervention.

**C)** Teve grupo controle?

( ) Sim ( ) Não

**O)** O estudo apresenta valores de marcadores inflamatórios (citocinas e outros marcadores) antes e após as intervenções de creatina?

( ) Sim	( ) Não

**S)** O estudo é um ensaio clínico randomizado e controlado?

( ) Sim	( ) Não

**Artigo 458: Statistics for investigation of multimodal MR imaging data and an application to multiple sclerosis patients.**

Fu L, Wolfson C, Worsley KJ, De Stefano N, Collins DL, Narayanan S, Arnold DL

**P)** Os participantes possuem mais de 18 anos de idade?

(X) Sim	( ) Não

"We studied **17 normal subjects**, 14 patients with relapsing remitting (RR) MS and 21 patients with chronic progressive (CP) MS. The ages of the normal subjects, RR MS patients, and CP MS patients were **31.7 ± 7.8 years, 33.5 ± 8.2 years, and 46.6 ± 3.7 years**, respectively (Table 1)". Todos os grupos têm idade média superior a 18 anos.

**I)** A intervenção utilizada no estudo é creatina? Vale qualquer formato de administração

( ) Sim	(X) Não

The study investigates "proton MR spectroscopic measures of diffuse cellular integrity" and "the ratio of resonance intensities of N-acetylaspartate over creatine (Cr)". Creatine (Cr) is measured as a **metabolite** within the brain to assess changes in multiple sclerosis, not as an administered intervention. The intervention used is Magnetic Resonance Spectroscopic Imaging (MRSI) as a diagnostic and assessment tool.

**C)** Teve grupo controle?

( ) Sim ( ) Não

**O)** O estudo apresenta valores de marcadores inflamatórios (citocinas e outros marcadores) antes e após as intervenções de creatina?

( ) Sim	( ) Não

**S)** O estudo é um ensaio clínico randomizado e controlado?

( ) Sim	( ) Não

**Artigo 459: Glucocorticoid receptor antagonism by mifepristone alters phosphocreatine breakdown during sepsis.**

Mitsuo T, Rounds J, Prechek D, Wilmore DW, Jacobs DO

**P)** Os participantes possuem mais de 18 anos de idade?

( ) Sim	(X) Não

"Participants: **Thirty-two adult male Wistar rats**".

**I)** A intervenção utilizada no estudo é creatina? Vale qualquer formato de administração

( ) Sim	( ) Não

**C)** Teve grupo controle?

( ) Sim ( ) Não

**O)** O estudo apresenta valores de marcadores inflamatórios (citocinas e outros marcadores) antes e após as intervenções de creatina?

( ) Sim	( ) Não

**S)** O estudo é um ensaio clínico randomizado e controlado?

( ) Sim	( ) Não

**Artigo 460: 1H-magnetic resonance spectroscopic observation of cultured malignant** cells**pharmacologically induced to different phenotypes.**

Nakai T, Ishima R, Sakahara H, Endo K, Konishi J, Akasaka K

**P)** Os participantes possuem mais de 18 anos de idade?

( ) Sim	(X) Não

"1H spectra of a **human lung adenocarcinoma ceil line cultured** with interferon gamma, dexamethasone, or sodium butyrate were obtained". The study used "**an in vitro model** to detect the effects of biologic modifiers on the 1H spectra of malignant cells". Therefore, the participants are cultured cells and not human subjects with an age.

**I)** A intervenção utilizada no estudo é creatina? Vale qualquer formato de administração

( ) Sim	( ) Não

**C)** Teve grupo controle?

( ) Sim ( ) Não

**O)** O estudo apresenta valores de marcadores inflamatórios (citocinas e outros marcadores) antes e após as intervenções de creatina?

( ) Sim	( ) Não

**S)** O estudo é um ensaio clínico randomizado e controlado?

( ) Sim	( ) Não

**Artigo 461: Evaluation of multiple sclerosis by 1H spectroscopic imaging at 4.1 T.**

Pan JW, Hetherington HP, Vaughan JT, Mitchell G, Pohost GM, Whitaker JN

**P)** Os participantes possuem mais de 18 anos de idade?

(X) Sim	( ) Não

"The patients included six women and two men, ranging in age from **21–45 years**... Controls included six women and two men, ranging in age from **25–35 years**".

**I)** A intervenção utilizada no estudo é creatina? Vale qualquer formato de administração

( ) Sim	(X) Não

The study reports on "'H spectroscopic imaging studies" and measures "the ratios of choline/N-acetyl (NA) compounds and creatine/NA". Creatine (Cr) is identified as a metabolite whose levels are being **measured** to characterize multiple sclerosis, not as an administered intervention.

**C)** Teve grupo controle?

( ) Sim ( ) Não

**O)** O estudo apresenta valores de marcadores inflamatórios (citocinas e outros marcadores) antes e após as intervenções de creatina?

( ) Sim	( ) Não

**S)** O estudo é um ensaio clínico randomizado e controlado?

( ) Sim	( ) Não

**Artigo 462: Pancreatitis in children--experience with 43 cases.**

Yeung CY, Lee HC, Huang FY, Ho MY, Kao HA, Liang DC, Hsu CH, Hung HY, Chang PY, Sheu JC

**P)** Os participantes possuem mais de 18 anos de idade?

( ) Sim	(X) Não

"We encountered **43 children, ranging in age from 2 to 18 years**, with pancreatitis over the past 10 years." Adicionalmente, "The medical records of **43 patients under the age of 18 years** with the diagnosis of pancreatitis hospitalized at the Mackay Memorial Hospital between 1983 and 1992 were reviewed."

**I)** A intervenção utilizada no estudo é creatina? Vale qualquer formato de administração

( ) Sim	( ) Não

**C)** Teve grupo controle?

( ) Sim ( ) Não

**O)** O estudo apresenta valores de marcadores inflamatórios (citocinas e outros marcadores) antes e após as intervenções de creatina?

( ) Sim	( ) Não

**S)** O estudo é um ensaio clínico randomizado e controlado?

( ) Sim	( ) Não

**Artigo 463: Nitric oxide inhibits creatine kinase and regulates** rat **heart contractile reserve.**

Gross WL, Bak MI, Ingwall JS, Arstall MA, Smith TW, Balligand JL, Kelly RA

**P)** Os participantes possuem mais de 18 anos de idade?

( ) Sim	(X) Não

"Rat Heart Preparation. **Male Sprague-Dawley rats** (Charles River Breeding Laboratories) weighing between 350 and 450 g were anesthetized by i.p. injection of 20 mg of pentobarbital sodium. Their hearts were excised quickly and placed in cold phosphate-free Krebs-Henseleit (KH) buffer." The study was conducted on **rats**, not human participants, therefore the concept of "more than 18 years of age" does not apply.

**I)** A intervenção utilizada no estudo é creatina? Vale qualquer formato de administração

( ) Sim	( ) Não

**C)** Teve grupo controle?

( ) Sim ( ) Não

**O)** O estudo apresenta valores de marcadores inflamatórios (citocinas e outros marcadores) antes e após as intervenções de creatina?

( ) Sim	( ) Não

**S)** O estudo é um ensaio clínico randomizado e controlado?

( ) Sim	( ) Não

**Artigo 464: Proton MR spectroscopy and MR imaging in acute and chronic multiple sclerosis--ringlike appearances in acute plaques.**

Landtblom AM, Sjöqvist L, Söderfeldt B, Nyland H, Thuomas KA

**P)** Os participantes possuem mais de 18 anos de idade?

(X) Sim	( ) Não

"Six patients aged **46-73 years** had PP MS. Ten patients aged **36-49 years** had clinically stable RR MS. Seven patients aged **41-64 years** had RP MS, also called "secondary progressive MS"."

**I)** A intervenção utilizada no estudo é creatina? Vale qualquer formato de administração

( ) Sim	(X) Não

The study describes the use of "localized proton MRS" to "determine the intensity ratios of certain brain metabolites". Creatine (Cr) is identified as a metabolite whose levels are being **measured** to characterize brain lesions in multiple sclerosis patients, not as an administered intervention. For example, "Relative peak ratios of N-acetylaspartate (NAA 2.03 ppm), phosphocreatine/creatine (Cr 3.04 ppm), choline-containing compounds (Cho 3.20 ppm), and inositol (Ins 3.54 ppm) were compared."

**C)** Teve grupo controle?

( ) Sim ( ) Não

**O)** O estudo apresenta valores de marcadores inflamatórios (citocinas e outros marcadores) antes e após as intervenções de creatina?

( ) Sim	( ) Não

**S)** O estudo é um ensaio clínico randomizado e controlado?

( ) Sim	( ) Não

**Artigo 465: Brain lesions in patients with AIDS: H-1 MR spectroscopy.**

Chang L, Miller BL, McBride D, Cornford M, Oropilla G, Buchthal S, Chiang F, Aronow H, Beck CK, Ernst T

**P)** Os participantes possuem mais de 18 anos de idade?

(X) Sim	( ) Não

"Thirty-five AIDS-related brain masses in 26 men (**aged 24-57 years**) were studied."

**I)** A intervenção utilizada no estudo é creatina? Vale qualquer formato de administração

( ) Sim	(X) Não

The study describes itself as a "H-1 MR spectroscopic study" focused on measuring "N-acetylaspartate (NAA), creatine, and choline". Creatine (Cr) is identified as a **measured metabolite** to aid in the diagnosis of brain lesions, not as an administered intervention.

**C)** Teve grupo controle?

( ) Sim ( ) Não

**O)** O estudo apresenta valores de marcadores inflamatórios (citocinas e outros marcadores) antes e após as intervenções de creatina?

( ) Sim	( ) Não

**S)** O estudo é um ensaio clínico randomizado e controlado?

( ) Sim	( ) Não

**Artigo 466: The duration of infection modifies mitochondrial oxidative capacity in** rat **skeletal muscle.**

Mizobata Y, Prechek D, Rounds JD, Robinson V, Wilmore DW, Jacobs DO

**P)** Os participantes possuem mais de 18 anos de idade?

( ) Sim	(X) Não

The study focuses on "myocardial mitochondrial function in **rat skeletal muscle**" and the figures are titled "Mizobata et al.: Mitochondrial function in skeletal muscle". The research was conducted on **rats**, not human participants, rendering the question of "more than 18 years of age" irrelevant.

**I)** A intervenção utilizada no estudo é creatina? Vale qualquer formato de administração

( ) Sim	( ) Não

**C)** Teve grupo controle?

( ) Sim ( ) Não

**O)** O estudo apresenta valores de marcadores inflamatórios (citocinas e outros marcadores) antes e após as intervenções de creatina?

( ) Sim	( ) Não

**S)** O estudo é um ensaio clínico randomizado e controlado?

( ) Sim	( ) Não

**Artigo 467: [Effect of bushen shengxue recipe on EPO gene expression of chronic renal insufficiency anemia in** rats**].**

Qu N, Wen JK, Li E

**P)** Os participantes possuem mais de 18 anos de idade?

( ) Sim	( ) Não [*Excluir*]

**I)** A intervenção utilizada no estudo é creatina? Vale qualquer formato de administração

( ) Sim	(	) Não [*Excluir*]

**C)** Teve grupo controle?

(	) Sim ( ) Não [*Excluir*]

**O)** O estudo apresenta valores de marcadores inflamatórios (citocinas e outros marcadores) antes e após as intervenções de creatina?

( ) Sim	(	) Não [*Excluir*]

**S)** O estudo é um ensaio clínico randomizado e controlado?

(	) Sim	( ) Não

**Artigo 468: Breakdown of adenine nucleotides, formation of oxygen free radicals, and early markers of cellular injury in endotoxic shock.**

Jabs CM, Neglen P, Eklof B

**P)** Os participantes possuem mais de 18 anos de idade?

( ) Sim	( ) Não [*Excluir*]

**I)** A intervenção utilizada no estudo é creatina? Vale qualquer formato de administração

( ) Sim	(	) Não [*Excluir*]

**C)** Teve grupo controle?

(	) Sim ( ) Não [*Excluir*]

**O)** O estudo apresenta valores de marcadores inflamatórios (citocinas e outros marcadores) antes e após as intervenções de creatina?

( ) Sim	(	) Não [*Excluir*]

**S)** O estudo é um ensaio clínico randomizado e controlado?

(	) Sim	( ) Não

**Artigo 469: Use of magnetic resonance imaging and P-31 magnetic resonance spectroscopy to detect and quantify muscle dysfunction in the amyopathic and myopathic variants of dermatomyositis.**

Park JH, Olsen NJ, King L Jr, Vital T, Buse R, Kari S, Hernanz-Schulman M, Price RR

**P)** Os participantes possuem mais de 18 anos de idade?

(X) Sim	( ) Não

"The patients with amyopathic DM (Table 1) were all white women ranging in age from **27 to 58 years**. The myopathic DM group consisted of 1 black and 10 white patients with ages ranging from **22 to 75 years**. The control group consisted of 11 healthy, nonobese individuals... Their ages ranged from **27 to 75 years**".

**I)** A intervenção utilizada no estudo é creatina? Vale qualquer formato de administração

( ) Sim	(X) Não

"Objective: To investigate the use of magnetic resonance imaging (MRI) and P-31 magnetic resonance spectroscopy (MRS) in characterizing the metabolic and functional status of muscles in patients with amyopathic dermatomyositis". "Biochemical status was quantitated with P-31 MRS, by determining concentrations of phosphate metabolites during rest and exercise.". The study measures "phosphocreatine (PCr)" as a metabolite, it does not administer creatine as an intervention.

**C)** Teve grupo controle?

( ) Sim ( ) Não

**O)** O estudo apresenta valores de marcadores inflamatórios (citocinas e outros marcadores) antes e após as intervenções de creatina?

( ) Sim	( ) Não

**S)** O estudo é um ensaio clínico randomizado e controlado?

( ) Sim	( ) Não

**Artigo 470: Laboratory evaluation of the inflammatory myopathies.**

Rider LG, Miller FW

**P)** Os participantes possuem mais de 18 anos de idade?

( ) Sim	(X) Não

The source describes the idiopathic inflammatory myopathies (IIM) as having "a bimodal age distribution, with the first peak occurring in childhood between the ages of **10 and 14 years** and a larger peak occurring in adulthood between 45 and 64 years of age (75)". This indicates that the diseases, which are the subject of this review, affect individuals under 18 years of age.

**I)** A intervenção utilizada no estudo é creatina? Vale qualquer formato de administração

( ) Sim	( ) Não

**C)** Teve grupo controle?

( ) Sim ( ) Não

**O)** O estudo apresenta valores de marcadores inflamatórios (citocinas e outros marcadores) antes e após as intervenções de creatina?

( ) Sim	( ) Não

**S)** O estudo é um ensaio clínico randomizado e controlado?

( ) Sim	( ) Não

**Artigo 471: In vivo 31P nuclear magnetic resonance spectroscopy of skeletal muscle energetics in endotoxemic** rats**: a prospective,** randomized **study.**

Gilles RJ, D'Orio V, Ciancabilla F, Carlier PG

**P)** Os participantes possuem mais de 18 anos de idade?

( ) Sim	(X) Não

“Wistar rats were randomized to different groups.”

**I)** A intervenção utilizada no estudo é creatina? Vale qualquer formato de administração

( ) Sim	( ) Não

**C)** Teve grupo controle?

( ) Sim ( ) Não

**O)** O estudo apresenta valores de marcadores inflamatórios (citocinas e outros marcadores) antes e após as intervenções de creatina?

( ) Sim	( ) Não

**S)** O estudo é um ensaio clínico randomizado e controlado?

( ) Sim	( ) Não

**Artigo 472: Myocardial energy metabolism and morphology in a** canine **model of sepsis.**

Solomon MA, Correa R, Alexander HR, Koev LA, Cobb JP, Kim DK, Roberts WC, Quezado ZM, Scholz TD, Cunnion RE, et al.

**P)** Os participantes possuem mais de 18 anos de idade?

( ) Sim	(X) Não

"Forty-three healthy, **2yr-old, 7-12 kg, purpose-bred beagles were studied**". The study was conducted on **animals (dogs)**, not human participants, and therefore the question of age over 18 years is not applicable to these subjects.

**I)** A intervenção utilizada no estudo é creatina? Vale qualquer formato de administração

( ) Sim	( ) Não

**C)** Teve grupo controle?

( ) Sim ( ) Não

**O)** O estudo apresenta valores de marcadores inflamatórios (citocinas e outros marcadores) antes e após as intervenções de creatina?

( ) Sim	( ) Não

**S)** O estudo é um ensaio clínico randomizado e controlado?

( ) Sim	( ) Não

**Artigo 473: Brain abscess observed by localized proton magnetic resonance spectroscopy.**

Harada M, Tanouchi M, Miyoshi H, Nishitani H, Kannuki S

**P)** Os participantes possuem mais de 18 anos de idade?

( ) Sim	(X) Não

The study is a "Case Report" focused on a single "patient". The article does not provide any specific information regarding the age of this patient, only referring to them as "the patient" throughout the methods and findings sections. Without explicit mention of the participant's age being over 18, it cannot be confirmed.

**I)** A intervenção utilizada no estudo é creatina? Vale qualquer formato de administração

( ) Sim	( ) Não

**C)** Teve grupo controle?

( ) Sim ( ) Não

**O)** O estudo apresenta valores de marcadores inflamatórios (citocinas e outros marcadores) antes e após as intervenções de creatina?

( ) Sim	( ) Não

**S)** O estudo é um ensaio clínico randomizado e controlado?

( ) Sim	( ) Não

**Artigo 474: Do changes in tumor blood flow necessarily lead to changes in tissue oxygenation and in bioenergetic status?**

Vaupel P, Kelleher DK, Engel T

**P)** Os participantes possuem mais de 18 anos de idade?

( ) Sim	(X) Não

"Sprague Dawley rats (310 ± 5 g) were used in this study". The study was conducted on **animals (rats)**, not human participants, and therefore the question of age over 18 years is not applicable to these subjects.

**I)** A intervenção utilizada no estudo é creatina? Vale qualquer formato de administração

( ) Sim	( ) Não

**C)** Teve grupo controle?

( ) Sim ( ) Não

**O)** O estudo apresenta valores de marcadores inflamatórios (citocinas e outros marcadores) antes e após as intervenções de creatina?

( ) Sim	( ) Não

**S)** O estudo é um ensaio clínico randomizado e controlado?

( ) Sim	( ) Não

**Artigo 475: Hematological indices of erythropoietin administration in athletes.**

Casoni I, Ricci G, Ballarin E, Borsetto C, Grazzi G, Guglielmini C, Manfredini F, Mazzoni G, Patracchini M, De Paoli Vitali E, et al.

**P)** Os participantes possuem mais de 18 anos de idade?

(X) Sim	( ) Não

"Included in this study were 20 male athletes of amateur level (6 cyclists, 8 runners and 6 soccer players), **ranging in age from 20 to 35 years**". Additionally, the control group consisted of "240 elite male athletes, **ranging in age from 18 to 36 years**". Both groups were composed of individuals aged 18 or older.

**I)** A intervenção utilizada no estudo é creatina? Vale qualquer formato de administração

( ) Sim	(X) Não

The study evaluated the effects of "**Recombinant human erythropoietin (EPO)** administration" on hematological indices. Creatine was measured as "erythrocyte content in creatine" but was not the intervention.

**C)** Teve grupo controle?

( ) Sim ( ) Não

**O)** O estudo apresenta valores de marcadores inflamatórios (citocinas e outros marcadores) antes e após as intervenções de creatina?

( ) Sim	( ) Não

**S)** O estudo é um ensaio clínico randomizado e controlado?

( ) Sim	( ) Não

**Artigo 476: The proton NMR spectrum in acute EAE: the significance of the change in the Cho:Cr ratio.**

Brenner RE, Munro PM, Williams SC, Bell JD, Barker GJ, Hawkins CP, Landon DN, McDonald WI

**P)** Os participantes possuem mais de 18 anos de idade?

( ) Sim	(X) Não

"Proton and phosphorus NMR spectra were obtained serially (from one to 6 weeks postinoculation) in **12 animals [five of which were controls]**". The study further mentions "the guinea pig brain" in its discussion of metabolites. Therefore, the study was conducted on **animals (guinea pigs)**, not human participants, and the question of age over 18 years is not applicable to these subjects.

**I)** A intervenção utilizada no estudo é creatina? Vale qualquer formato de administração

( ) Sim	( ) Não

**C)** Teve grupo controle?

( ) Sim ( ) Não

**O)** O estudo apresenta valores de marcadores inflamatórios (citocinas e outros marcadores) antes e após as intervenções de creatina?

( ) Sim	( ) Não

**S)** O estudo é um ensaio clínico randomizado e controlado?

( ) Sim	( ) Não

**Artigo 477: Sneddon's syndrome: diagnosis by skin biopsy and MRI in 17 patients.**

Stockhammer G, Felber SR, Zelger B, Sepp N, Birbamer GG, Fritsch PO, Aichner FT

**P)** Os participantes possuem mais de 18 anos de idade?

(X) Sim	( ) Não

"The mean age at the time of diagnosis was **35.8 (range, 24-58) years**". This indicates that all 17 patients were aged 24 years or older at the time of their diagnosis and participation in the study, meaning they were all over 18 years old.

**I)** A intervenção utilizada no estudo é creatina? Vale qualquer formato de administração

( ) Sim	(X) Não

The study's objective was to "evaluate clinical, laboratory, histological, and neuroradiological findings in a series of 17 patients to improve diagnostic criteria for Sneddon's syndrome". It specifically mentions that "Laboratory examinations showed impaired creatinine clearance in eight patients", which is a measurement of kidney function and a byproduct of creatine metabolism, but **creatine was not administered as an intervention in any format**.

**C)** Teve grupo controle?

( ) Sim ( ) Não

**O)** O estudo apresenta valores de marcadores inflamatórios (citocinas e outros marcadores) antes e após as intervenções de creatina?

( ) Sim	( ) Não

**S)** O estudo é um ensaio clínico randomizado e controlado?

( ) Sim	( ) Não

**Artigo 478: [Mitochondrial disorder secondary to inflammation in polymyositis. Two cases].**

Kaminsky P, Klein M, Robin-Lherbier B, Walker PM, Duc ML, Escanyé JM, Duc M

**P)** Os participantes possuem mais de 18 anos de idade?

(X) Sim	( ) Não

The source provides a table showing the ages of patients with anti-mitochondrial antibody myositis: "**Age, Sex 62, M 52, M 61, F 64, F 49, F 46, M 51, F**". All listed ages are greater than 18 years.

**I)** A intervenção utilizada no estudo é creatina? Vale qualquer formato de administração

( ) Sim	(X) Não

The objective of the study was to investigate "Inflammatory myopathy associated with anti-mitochondrial antibodies: a distinct phenotype with cardiac involvement". The study focused on "identif[ying] and review[ing] the medical records of those patients who tested positive for AMA". There is **no mention of creatine or any form of its administration as an intervention** in the provided excerpts.

**C)** Teve grupo controle?

( ) Sim ( ) Não

**O)** O estudo apresenta valores de marcadores inflamatórios (citocinas e outros marcadores) antes e após as intervenções de creatina?

( ) Sim	( ) Não

**S)** O estudo é um ensaio clínico randomizado e controlado?

( ) Sim	( ) Não

**Artigo 479: Pentoxifylline treatment of sepsis in conscious Yucatan minipigs.**

Law WR, Nadkarni VM, Fletcher MA, Nevola JJ, Eckstein JM, Quance J, McKenna TM, Lee CH, Williams TJ

**P)** Os participantes possuem mais de 18 anos de idade?

( ) Sim	( ) Não [*Excluir*]

**I)** A intervenção utilizada no estudo é creatina? Vale qualquer formato de administração

( ) Sim	(	) Não [*Excluir*]

**C)** Teve grupo controle?

(	) Sim ( ) Não [*Excluir*]

**O)** O estudo apresenta valores de marcadores inflamatórios (citocinas e outros marcadores) antes e após as intervenções de creatina?

( ) Sim	(	) Não [*Excluir*]

**S)** O estudo é um ensaio clínico randomizado e controlado?

(	) Sim	( ) Não

**Artigo 480: Acute effects of tumor necrosis factor alpha or lymphotoxin on global blood flow, laser Doppler flux, and bioenergetic status of subcutaneous** rodent **tumors.**

Kluge M, Elger B, Engel T, Schaefer C, Seega J, Vaupel P

**P)** Os participantes possuem mais de 18 anos de idade?

( ) Sim	(X) Não

"DS-sarcoma ascites cells were serially passaged in the peritoneal cavity of **Sprague-Dawley rats** (200-400 g)". This study was conducted on animals (rats), not human participants, making the question of age over 18 years inapplicable.

**I)** A intervenção utilizada no estudo é creatina? Vale qualquer formato de administração

( ) Sim	( ) Não

**C)** Teve grupo controle?

( ) Sim ( ) Não

**O)** O estudo apresenta valores de marcadores inflamatórios (citocinas e outros marcadores) antes e após as intervenções de creatina?

( ) Sim	( ) Não

**S)** O estudo é um ensaio clínico randomizado e controlado?

( ) Sim	( ) Não

**Artigo 481: Serum erythropoietin levels in the immediate kidney-posttransplant period.**

Goch J, Birgegård G, Wikström B, Tufveson G, Danielson BG

**P)** Os participantes possuem mais de 18 anos de idade?

(X) Sim	( ) Não

"The study involves patients undergoing kidney transplantation. While a specific age range is not explicitly provided for the participants in this article, kidney transplant recipients are typically adults with end-stage renal disease, as implied by the medical context and the measurement of adult-range biochemical markers such as serum creatinine. Other studies concerning patients with impaired renal function also indicate an adult population, for example, "There were ten males and nine females, whose ages ranged from 32 to 76 years"."

**I)** A intervenção utilizada no estudo é creatina? Vale qualquer formato de administração

( ) Sim	(X) Não

"The study investigates "Serum Erythropoietin Levels" in kidney transplant recipients, examining "how transplantation procedures influence the serum EPO levels". **No creatine was administered or used as an intervention in any format**; creatinine levels were measured as a marker of renal function."

**C)** Teve grupo controle?

( ) Sim ( ) Não

**O)** O estudo apresenta valores de marcadores inflamatórios (citocinas e outros marcadores) antes e após as intervenções de creatina?

( ) Sim	( ) Não

**S)** O estudo é um ensaio clínico randomizado e controlado?

( ) Sim	( ) Não

**Artigo 482: Erythropoietin in preeclampsia.**

Kaupke CJ, Vaziri ND, Powers DR, Gonzales E

**P)** Os participantes possuem mais de 18 anos de idade?

( ) Sim	( ) Não [*Excluir*]

**I)** A intervenção utilizada no estudo é creatina? Vale qualquer formato de administração

( ) Sim	(	) Não [*Excluir*]

**C)** Teve grupo controle?

(	) Sim ( ) Não [*Excluir*]

**O)** O estudo apresenta valores de marcadores inflamatórios (citocinas e outros marcadores) antes e após as intervenções de creatina?

( ) Sim	(	) Não [*Excluir*]

**S)** O estudo é um ensaio clínico randomizado e controlado?

(	) Sim	( ) Não

**Artigo 483: Renal function and metabolism during endotoxemia in** rats**: role of hypoperfusion.**

van Lambalgen AA, van Kraats AA, van den Bos GC, Stel HV, Straub J, Donker AJ, Thijs LG

**P)** Os participantes possuem mais de 18 anos de idade?

( ) Sim	( ) Não [*Excluir*]

**I)** A intervenção utilizada no estudo é creatina? Vale qualquer formato de administração

( ) Sim	(	) Não [*Excluir*]

**C)** Teve grupo controle?

(	) Sim ( ) Não [*Excluir*]

**O)** O estudo apresenta valores de marcadores inflamatórios (citocinas e outros marcadores) antes e após as intervenções de creatina?

( ) Sim	(	) Não [*Excluir*]

**S)** O estudo é um ensaio clínico randomizado e controlado?

(	) Sim	( ) Não

**Artigo 484: ATP production in isolated muscle mitochondria from haemodialysis patients: effects of correction of anaemia with erythropoietin.**

Bárány P, Wibom R, Hultman E, Bergström J

**P)** Os participantes possuem mais de 18 anos de idade?

(X) Sim	( ) Não

"Twenty-one haemodialysis patients (10 females, 11 males; **age 41 f 9 years, m e a n f s~**) with anaemia... participated in the study". Additionally, "Three groups of healthy subjects (10 females, 11 males, **aged 18-45 years**) with different degrees of physical activity were used as controls for the mitochondrial investigations". Both patient and control groups included individuals aged 18 years or older.

**I)** A intervenção utilizada no estudo é creatina? Vale qualquer formato de administração

( ) Sim	(X) Não

The study focuses on "ATP production in isolated muscle mitochondria from haemodialysis patients: **effects of correction of anaemia with erythropoietin**". It explicitly states, "The patients received intravenous **recombinant human erythropoietin treatment** (50-200 i.u./kg body weight) after haemodialysis 3 times a week". While "total creatine" and "phosphocreatine" levels were "determined in skeletal muscle" as measurements, creatine itself was not administered as an intervention.

**C)** Teve grupo controle?

( ) Sim ( ) Não

**O)** O estudo apresenta valores de marcadores inflamatórios (citocinas e outros marcadores) antes e após as intervenções de creatina?

( ) Sim	( ) Não

**S)** O estudo é um ensaio clínico randomizado e controlado?

( ) Sim	( ) Não

**Artigo 485: Low-dose subcutaneous erythropoietin in continuous ambulatory peritoneal dialysis.**

Rotellar C, Mazzoni MJ, Mosher WF, Aminrazavi M, Bauer A, Rakowski TA, Schreiner GE, Winchester JF

**P)** Os participantes possuem mais de 18 anos de idade?

(X) Sim	( ) Não

The study describes "Fifteen patients (7 males and 8 females) were started on subcutaneous erythropoietin" for the treatment of anemia in individuals undergoing continuous ambulatory peritoneal dialysis (CAPD). While the exact age range is not explicitly stated in this excerpt, patients with end-stage renal disease requiring CAPD are overwhelmingly adults. Other sources within the provided articles confirm adult populations for similar kidney conditions; for instance, a study on renal insufficiency patients included individuals "whose ages ranged from 32 to 76 years".

**I)** A intervenção utilizada no estudo é creatina? Vale qualquer formato de administração

( ) Sim	(X) Não

The intervention administered in this study was "**subcutaneous erythropoietin 3,000 units 3 times a week**". There is **no mention of creatine or any form of its administration as an intervention** in the provided excerpts.

**C)** Teve grupo controle?

( ) Sim ( ) Não

**O)** O estudo apresenta valores de marcadores inflamatórios (citocinas e outros marcadores) antes e após as intervenções de creatina?

( ) Sim	( ) Não

**S)** O estudo é um ensaio clínico randomizado e controlado?

( ) Sim	( ) Não

**Artigo 486: Metabolic, functional, and histologic characterization of the heterotopically transplanted** rat **heart when used as a model for the study of long-term recovery from global ischemia.**

Galiñanes M, Hearse DJ

**P)** Os participantes possuem mais de 18 anos de idade?

( ) Sim	( ) Não [*Excluir*]

**I)** A intervenção utilizada no estudo é creatina? Vale qualquer formato de administração

( ) Sim	(	) Não [*Excluir*]

**C)** Teve grupo controle?

(	) Sim ( ) Não [*Excluir*]

**O)** O estudo apresenta valores de marcadores inflamatórios (citocinas e outros marcadores) antes e após as intervenções de creatina?

( ) Sim	(	) Não [*Excluir*]

**S)** O estudo é um ensaio clínico randomizado e controlado?

(	) Sim	( ) Não

**Artigo 487: Sepsis does not impair tricarboxylic acid cycle in the heart.**

Hotchkiss RS, Song SK, Neil JJ, Chen RD, Manchester JK, Karl IE, Lowry OH, Ackerman JJ

**P)** Os participantes possuem mais de 18 anos de idade?

( ) Sim	(X) Não

"Sepsis was induced in **12 female rats**...; **seven control rats** underwent cecal manipulation without ligation". This study was conducted on animals (rats), not human participants, making the question of age over 18 years inapplicable.

**I)** A intervenção utilizada no estudo é creatina? Vale qualquer formato de administração

( ) Sim	( ) Não

**C)** Teve grupo controle?

( ) Sim ( ) Não

**O)** O estudo apresenta valores de marcadores inflamatórios (citocinas e outros marcadores) antes e após as intervenções de creatina?

( ) Sim	( ) Não

**S)** O estudo é um ensaio clínico randomizado e controlado?

( ) Sim	( ) Não

**Artigo 488: Effects of the recombinant human erythropoietin (rHuEPO) administration on hematologic parameters, red cell creatine and 2,3-diphosphoglycerate contents, in patients affected by end-stage renal disease.**

Ricci G, Masotti M, De Paoli Vitali E, Vedovato M, Bedani PL, Gilli P

**P)** Os participantes possuem mais de 18 anos de idade?

(X) Sim	( ) Não

"Twenty patients (9 males and 11 females), **aged 30-70 years**, on thrice-weekly maintenance hemodialysis for end-stage chronic renal failure, were admitted to this study". This clearly indicates that all participants were older than 18 years of age.

**I)** A intervenção utilizada no estudo é creatina? Vale qualquer formato de administração

( ) Sim	(X) Não

The study's title indicates its focus on "effects of the **recombinant human erythropoietin (rHuEPO) administration**". The methods explicitly state that "Twenty patients... were treated with **recombinant human erythropoietin (rHuEPO)**". While "Red cell creatine contents were determined" as a measurement, **creatine itself was not administered or used as an intervention** in any format.

**C)** Teve grupo controle?

( ) Sim ( ) Não

**O)** O estudo apresenta valores de marcadores inflamatórios (citocinas e outros marcadores) antes e após as intervenções de creatina?

( ) Sim	( ) Não

**S)** O estudo é um ensaio clínico randomizado e controlado?

( ) Sim	( ) Não

**Artigo 489: Dermatomyositis: correlative MR imaging and P-31 MR spectroscopy for quantitative characterization of inflammatory disease.**

Park JH, Vansant JP, Kumar NG, Gibbs SJ, Curvin MS, Price RR, Partain CL, James AE Jr

**P)** Os participantes possuem mais de 18 anos de idade?

(X) Sim	( ) Não

"Healthy nonobese subjects, two men and three women, were selected as age- and sex-matched control subjects. ... Four patients who had biopsy-proved dermatomyositis with varying degrees of muscular weakness and skin rash were identified by means of routine clinical examinations". While specific ages for this study are not explicitly stated, the lead author (Park JH) has conducted other studies on dermatomyositis (e.g., Article 469), where participants (both patients and controls) ranged from "22 to 75 years", indicating an adult population. Additionally, Article 470, which reviews inflammatory myopathies and cites this study, notes that idiopathic inflammatory myopathies have "a larger peak occurring in adulthood between 45 and 64 years of age". This context strongly suggests that the participants were over 18 years of age.

**I)** A intervenção utilizada no estudo é creatina? Vale qualquer formato de administração

( ) Sim	(X) Não

The study's objective was to "examine four patients with dermatomyositis and five control subjects" using "**Magnetic resonance (MR) imaging and phosphorus-31 MR spectrosco-py**". It also states that "Concentrations of adenosine triphosphate and phosphocreatine (PCr) in the diseased muscles were ... determined". While phosphocreatine was a *measured metabolite*, **creatine itself was not administered or used as an intervention** in any format in this study.

**C)** Teve grupo controle?

( ) Sim ( ) Não

**O)** O estudo apresenta valores de marcadores inflamatórios (citocinas e outros marcadores) antes e após as intervenções de creatina?

( ) Sim	( ) Não

**S)** O estudo é um ensaio clínico randomizado e controlado?

( ) Sim	( ) Não

**Artigo 490: Effects of a mixed physical activity (biathlon) on haematologic parameters, red cell 2,3-DPG and creatine, serum erythropoietin, urinary enzymes and microalbumin.**

Ricci G, Masotti M, De Paoli Vitali E, Vedovato M, Zanotti G

**P)** Os participantes possuem mais de 18 anos de idade?

(X) Sim	( ) Não

"Our **10 male biathletes (aged 22-48 years)** were studied during a race... **10 sedentary, healthy non-smoking males (aged 21-40 yr) were used as controls**". This explicitly states that all participants were over 18 years old.

**I)** A intervenção utilizada no estudo é creatina? Vale qualquer formato de administração

( ) Sim	(X) Não

The study investigates the "Effects of a **mixed physical activity (biathlon)**" on various parameters, including "red cell creatine". Red cell creatine content was a *measured parameter* ("significant increase of red cell creatine content both after exercise and vs our controls"), not an administered intervention. The study did not involve the administration of creatine in any format.

**C)** Teve grupo controle?

( ) Sim ( ) Não

**O)** O estudo apresenta valores de marcadores inflamatórios (citocinas e outros marcadores) antes e após as intervenções de creatina?

( ) Sim	( ) Não

**S)** O estudo é um ensaio clínico randomizado e controlado?

( ) Sim	( ) Não

**Artigo 491: 1,25 Dihydroxyvitamin D3 modifies cyclosporine-induced bone loss.**

Epstein S, Schlosberg M, Fallon M, Thomas S, Movsowitz C, Ismail F

**P)** Os participantes possuem mais de 18 anos de idade?

( ) Sim	(X) Não

"Fifty **male Sprague Dawley rats** approximately 400 g in weight were purchased from Harlan Sprague Dawley (Indianapolis, IN)". This study was conducted on animals (rats), not human participants, making the question of age over 18 years inapplicable.

**I)** A intervenção utilizada no estudo é creatina? Vale qualquer formato de administração

( ) Sim	( ) Não

**C)** Teve grupo controle?

( ) Sim ( ) Não

**O)** O estudo apresenta valores de marcadores inflamatórios (citocinas e outros marcadores) antes e após as intervenções de creatina?

( ) Sim	( ) Não

**S)** O estudo é um ensaio clínico randomizado e controlado?

( ) Sim	( ) Não

**Artigo 492: Evaluation of indicators of erythropoiesis stimulated by recombinant human erythropoietin in renal anemia.**

Schmidt G, Stamminger G, Daniel A, Gross J, Syllm-Rapoport I, Lun A, Zoellner K

**P)** Os participantes possuem mais de 18 anos de idade?

( ) Sim	( ) Não [*Excluir*]

**I)** A intervenção utilizada no estudo é creatina? Vale qualquer formato de administração

( ) Sim	(	) Não [*Excluir*]

**C)** Teve grupo controle?

(	) Sim ( ) Não [*Excluir*]

**O)** O estudo apresenta valores de marcadores inflamatórios (citocinas e outros marcadores) antes e após as intervenções de creatina?

( ) Sim	(	) Não [*Excluir*]

**S)** O estudo é um ensaio clínico randomizado e controlado?

(	) Sim	( ) Não

**Artigo 493: An in vivo examination of** rat **brain during sepsis with 31P-NMR spectroscopy**

Hotchkiss RS, Long RC, Hall JR, Shires GT 3rd, Brouillard RG, Millikan WJ, Jones DP

**P)** Os participantes possuem mais de 18 anos de idade?

( ) Sim	(X) Não

"Thirty **male Sprague-Dawley rats** (ZOO-250 g) were used in this study". This indicates that the study was conducted on animals (rats), not human participants, making the question of age over 18 years inapplicable.

**I)** A intervenção utilizada no estudo é creatina? Vale qualquer formato de administração

( ) Sim	( ) Não

**C)** Teve grupo controle?

( ) Sim ( ) Não

**O)** O estudo apresenta valores de marcadores inflamatórios (citocinas e outros marcadores) antes e após as intervenções de creatina?

( ) Sim	( ) Não

**S)** O estudo é um ensaio clínico randomizado e controlado?

( ) Sim	( ) Não

**Artigo 494: 31P-nuclear magnetic resonance studies of the effect of recombinant human interleukin 1 alpha on the bioenergetics of RIF-1 tumors.**

Constantinidis I, Braunschweiger PG, Wehrle JP, Kumar N, Johnson CS, Furmanski P, Glickson JD

**P)** Os participantes possuem mais de 18 anos de idade?

( ) Sim	( ) Não [*Excluir*]

**I)** A intervenção utilizada no estudo é creatina? Vale qualquer formato de administração

( ) Sim	(	) Não [*Excluir*]

**C)** Teve grupo controle?

(	) Sim ( ) Não [*Excluir*]

**O)** O estudo apresenta valores de marcadores inflamatórios (citocinas e outros marcadores) antes e após as intervenções de creatina?

( ) Sim	(	) Não [*Excluir*]

**S)** O estudo é um ensaio clínico randomizado e controlado?

(	) Sim	( ) Não

**Artigo 495: Early metabolic response to tumor necrosis factor in** mouse **sarcoma: a phosphorus-31 nuclear magnetic resonance study.**

Shine N, Palladino MA Jr, Patton JS, Deisseroth A, Karczmar GS, Matson GB, Weiner MW

**P)** Os participantes possuem mais de 18 anos de idade?

( ) Sim	(X) Não

"Experiments were performed on **6- to 8-wk-old female BALB/c x C57BL/6 Fi mice**". This indicates that the study was conducted on animals (mice), not human participants, making the question of age over 18 years inapplicable.

**I)** A intervenção utilizada no estudo é creatina? Vale qualquer formato de administração

( ) Sim	( ) Não

**C)** Teve grupo controle?

( ) Sim ( ) Não

**O)** O estudo apresenta valores de marcadores inflamatórios (citocinas e outros marcadores) antes e após as intervenções de creatina?

( ) Sim	( ) Não

**S)** O estudo é um ensaio clínico randomizado e controlado?

( ) Sim	( ) Não

**Artigo 496: Glomerular** cells **and macrophages in the progression of experimental focal and segmental glomerulosclerosis.**

Matsumoto K, Atkins RC

**P)** Os participantes possuem mais de 18 anos de idade?

( ) Sim	(X) Não

"Experiments were carried out on **Male Sprague-Dawley rats** weighing 200 to 250 g (Central Animal House, Monash University, Melbourne, Australia) were used in all studies". This indicates that the study was conducted on animals (rats), not human participants, making the question of age over 18 years inapplicable.

**I)** A intervenção utilizada no estudo é creatina? Vale qualquer formato de administração

( ) Sim	( ) Não

**C)** Teve grupo controle?

( ) Sim ( ) Não

**O)** O estudo apresenta valores de marcadores inflamatórios (citocinas e outros marcadores) antes e após as intervenções de creatina?

( ) Sim	( ) Não

**S)** O estudo é um ensaio clínico randomizado e controlado?

( ) Sim	( ) Não

**Artigo 497: Metabolic response of glioblastoma to adoptive immunotherapy: detection by phosphorus MR spectroscopy.**

Ross BD, Tropp J, Derby KA, Sugiura S, Hawryszko C, Jacques DB, Ingram M

**P)** Os participantes possuem mais de 18 anos de idade?

(X) Sim	( ) Não

“The glioma patient was a 46-year-old man with a 3-week history of weakness and dyspraxia of the left leg but no other symptoms.”

**I)** A intervenção utilizada no estudo é creatina? Vale qualquer formato de administração

( ) Sim	(X) Não

“Computed tomography was perfomed and the patient started on steroid therapy with dexamethasone.”

**C)** Teve grupo controle?

( ) Sim ( ) Não

**O)** O estudo apresenta valores de marcadores inflamatórios (citocinas e outros marcadores) antes e após as intervenções de creatina?

( ) Sim	( ) Não

**S)** O estudo é um ensaio clínico randomizado e controlado?

( ) Sim	( ) Não

**Artigo 498: The effect of hypodynamic endotoxin shock on myocardial high energy phosphates in the** rat**.**

Raymond RM, Gordey J

**P)** Os participantes possuem mais de 18 anos de idade?

( ) Sim	(X) Não

"We used **125 Sprague-Dawley rats**, weighing 250-400 g, in this study". This indicates that the study was conducted on animals (rats), not human participants, making the question of age over 18 years inapplicable.

**I)** A intervenção utilizada no estudo é creatina? Vale qualquer formato de administração

( ) Sim	( ) Não

**C)** Teve grupo controle?

( ) Sim ( ) Não

**O)** O estudo apresenta valores de marcadores inflamatórios (citocinas e outros marcadores) antes e após as intervenções de creatina?

( ) Sim	( ) Não

**S)** O estudo é um ensaio clínico randomizado e controlado?

( ) Sim	( ) Não

**Artigo 499: Assessment of the early cellular membrane response to live Escherichia coli bacteremia.**

Shiono S, Fantini GA, Roberts JP, Chiao J, Shires GT

**P)** Os participantes possuem mais de 18 anos de idade?

( ) Sim	(X) Não

"Ten conditioned **mongrel dogs** of either sex, weighing 13-16 kg were used in the experiments". This indicates that the study was conducted on animals (dogs), not human participants, making the question of age over 18 years inapplicable.

**I)** A intervenção utilizada no estudo é creatina? Vale qualquer formato de administração

( ) Sim	( ) Não

**C)** Teve grupo controle?

( ) Sim ( ) Não

**O)** O estudo apresenta valores de marcadores inflamatórios (citocinas e outros marcadores) antes e após as intervenções de creatina?

( ) Sim	( ) Não

**S)** O estudo é um ensaio clínico randomizado e controlado?

( ) Sim	( ) Não

clínico randomizado e controlado?

(	) Sim	( ) Não

**Artigo 500: Molecular markers of hemostasis activation in nephrotic syndrome.**

Sagripanti A, Cupisti A, Ferdeghini M, Pinori E, Barsotti G

**P)** Os participantes possuem mais de 18 anos de idade?

( ) Sim	(X) Não

"Twenty-three patients (13 males and 10 females), **aged 16-70 (median 38) years**, affected by nephrotic syndrome and consecutively admitted to our clinic, were studied". This indicates that some participants were under 18 years of age (16 and 17 years old).

**I)** A intervenção utilizada no estudo é creatina? Vale qualquer formato de administração

( ) Sim	( ) Não

**C)** Teve grupo controle?

( ) Sim ( ) Não

**O)** O estudo apresenta valores de marcadores inflamatórios (citocinas e outros marcadores) antes e após as intervenções de creatina?

( ) Sim	( ) Não

**S)** O estudo é um ensaio clínico randomizado e controlado?

( ) Sim	( ) Não

**Artigo 501: In vivo phosphorus 31 magnetic resonance spectroscopy of** rat **hind limb skeletal muscle during sepsis.**

Jacobs DO, Maris J, Fried R, Settle RG, Rolandelli RR, Koruda MJ, Chance B, Rombeau JL

**P)** Os participantes possuem mais de 18 anos de idade?

( ) Sim	(X) Não

"Adult male Sprague-Dawley **rats** weighing 275 to 300 g were obtained (Charles River Breeding Laboratories, Wilmington, Mass)". This indicates that the study was conducted on **animals** (rats), not human participants, making the question of age over 18 years inapplicable.

**I)** A intervenção utilizada no estudo é creatina? Vale qualquer formato de administração

( ) Sim	( ) Não

**C)** Teve grupo controle?

( ) Sim ( ) Não

**O)** O estudo apresenta valores de marcadores inflamatórios (citocinas e outros marcadores) antes e após as intervenções de creatina?

( ) Sim	( ) Não

**S)** O estudo é um ensaio clínico randomizado e controlado?

( ) Sim	( ) Não

**Artigo 502: Neopterin levels in long-term hemodialysis.**

Fuchs D, Hausen A, Reibnegger G, Werner ER, von Dittrich P, Wachter H

**P)** Os participantes possuem mais de 18 anos de idade?

( ) Sim	( ) Não [*Excluir*]

**I)** A intervenção utilizada no estudo é creatina? Vale qualquer formato de administração

( ) Sim	(	) Não [*Excluir*]

**C)** Teve grupo controle?

(	) Sim ( ) Não [*Excluir*]

**O)** O estudo apresenta valores de marcadores inflamatórios (citocinas e outros marcadores) antes e após as intervenções de creatina?

( ) Sim	(	) Não [*Excluir*]

**S)** O estudo é um ensaio clínico randomizado e controlado?

(	) Sim	( ) Não

**Artigo 503: Effects of exercise on haematologic parameters, serum iron, serum ferritin, red cell 2,3-diphosphoglycerate and creatine contents, and serum erythropoietin in long-distance runners during basal training.**

Ricci G, Masotti M, De Paoli Vitali E, Vedovato M, Zanotti G

**P)** Os participantes possuem mais de 18 anos de idade?

(X) Sim	( ) Não

“Eighteen male runners (aged 20-35 years) were admitted to this study.”

**I)** A intervenção utilizada no estudo é creatina? Vale qualquer formato de administração

( ) Sim	(X) Não

Não há administração de creatina. “The red cell creatine was assessed by the enzymatic-colorimetric method [9] using the kit suplied by Boehringer Biochemia (Mannheim, FRG).”

**C)** Teve grupo controle?

( ) Sim ( ) Não

**O)** O estudo apresenta valores de marcadores inflamatórios (citocinas e outros marcadores) antes e após as intervenções de creatina?

( ) Sim	( ) Não

**S)** O estudo é um ensaio clínico randomizado e controlado?

( ) Sim	( ) Não

**Artigo 504: Alterations in metabolite levels in carbohydrate and energy metabolism of** rat **in hemorrhagic shock and sepsis.**

Mori E, Hasebe M, Kobayashi K, Iijima N

**P)** Os participantes possuem mais de 18 anos de idade?

( ) Sim	(X) Não

"The sources of animals and materials used in this study were as follows: **male Sprague-Dawley rats weighing 160 to 180 g** from Charles River Japan Inc (Atsugi, Japan)". This indicates that the study was conducted on **animals (rats)**, not human participants, making the question of age over 18 years inapplicable.

**I)** A intervenção utilizada no estudo é creatina? Vale qualquer formato de administração

( ) Sim	( ) Não

**C)** Teve grupo controle?

( ) Sim ( ) Não

**O)** O estudo apresenta valores de marcadores inflamatórios (citocinas e outros marcadores) antes e após as intervenções de creatina?

( ) Sim	( ) Não

**S)** O estudo é um ensaio clínico randomizado e controlado?

( ) Sim	( ) Não

**Artigo 505:** **Activation of platelets by microfibrils and collagen. A comparative study.**

Legrand YJ, Fauvel F, Arbeille B, Leger D, Mouhli H, Gutman N, Muh JP

**P)** Os participantes possuem mais de 18 anos de idade?

( ) Sim	( ) Não

**I)** A intervenção utilizada no estudo é creatina? Vale qualquer formato de administração

( ) Sim	( ) Não

**C)** Teve grupo controle?

( ) Sim ( ) Não

**O)** O estudo apresenta valores de marcadores inflamatórios (citocinas e outros marcadores) antes e após as intervenções de creatina?

( ) Sim	( ) Não

**S)** O estudo é um ensaio clínico randomizado e controlado?

( ) Sim	( ) Não

**Artigo 506: Correction of anaemia following renal transplantation: serial changes in serum immunoreactive erythropoietin, absolute reticulocyte count and red-cell creatine levels.**

Rejman AS, Grimes AJ, Cotes PM, Mansell MA, Joekes AM

**P)** Os participantes possuem mais de 18 anos de idade?

( ) Sim	( ) Não

**I)** A intervenção utilizada no estudo é creatina? Vale qualquer formato de administração

( ) Sim	( ) Não

**C)** Teve grupo controle?

( ) Sim ( ) Não

**O)** O estudo apresenta valores de marcadores inflamatórios (citocinas e outros marcadores) antes e após as intervenções de creatina?

( ) Sim	( ) Não

**S)** O estudo é um ensaio clínico randomizado e controlado?

( ) Sim	( ) Não

**Artigo 507: Effects of 2-[(5-chloro-2-methoxyphenyl)azo]-1H-imidazole (M6434) on hemorrhagic, cardiogenic, and endotoxin shock in** rats **and** rabbits**.**

Ohnishi H, Yamaguchi K, Sato M, Uemura A, Funato H, Dabasaki T

**P)** Os participantes possuem mais de 18 anos de idade?

( ) Sim	( ) Não [*Excluir*]

**I)** A intervenção utilizada no estudo é creatina? Vale qualquer formato de administração

( ) Sim	(	) Não [*Excluir*]

**C)** Teve grupo controle?

(	) Sim ( ) Não [*Excluir*]

**O)** O estudo apresenta valores de marcadores inflamatórios (citocinas e outros marcadores) antes e após as intervenções de creatina?

( ) Sim	(	) Não [*Excluir*]

**S)** O estudo é um ensaio clínico randomizado e controlado?

(	) Sim	( ) Não

**Artigo 508: Increased urinary beta-thromboglobulin excretion in diabetes assayed with a modified RIA kit-technique.**

van Oost BA, Veldhuyzen B, Timmermans AP, Sixma JJ

**P)** Os participantes possuem mais de 18 anos de idade?

( ) Sim	( ) Não [*Excluir*]

**I)** A intervenção utilizada no estudo é creatina? Vale qualquer formato de administração

( ) Sim	(	) Não [*Excluir*]

**C)** Teve grupo controle?

(	) Sim ( ) Não [*Excluir*]

**O)** O estudo apresenta valores de marcadores inflamatórios (citocinas e outros marcadores) antes e após as intervenções de creatina?

( ) Sim	(	) Não [*Excluir*]

**S)** O estudo é um ensaio clínico randomizado e controlado?

(	) Sim	( ) Não

**Artigo 509: An evaluation of perfluorochemical resuscitation after hypoxic hypotension.**

Proctor HJ, Palladino GW, Cairns C, Jobsis FF

**P)** Os participantes possuem mais de 18 anos de idade?

( ) Sim	(X) Não

"Nonadult male Spragues-Dawley rats (250 to 300 gm) were anesthetized using sodium pentothal (50 mg/kg, IP)."

**I)** A intervenção utilizada no estudo é creatina? Vale qualquer formato de administração

( ) Sim	( ) Não

**C)** Teve grupo controle?

( ) Sim ( ) Não

**O)** O estudo apresenta valores de marcadores inflamatórios (citocinas e outros marcadores) antes e após as intervenções de creatina?

( ) Sim	( ) Não

**S)** O estudo é um ensaio clínico randomizado e controlado?

( ) Sim	( ) Não

**Artigo 510: Effect of platelet-activating factor (PAF) on human platelets.**

Chesney CM, Pifer DD, Byers LW, Muirhead EE

**P)** Os participantes possuem mais de 18 anos de idade?

( ) Sim	(X) Não

The provided sources state that "The effect of pure synthetic PAF... was studied in **human platelets**" and refer to "studies... on **human platelets**". However, **no information regarding the age of the human participants or whether they are over 18 years old is explicitly stated or can be directly transcribed from the provided text.** Therefore, it is not possible to confirm that the participants were over 18.

**I)** A intervenção utilizada no estudo é creatina? Vale qualquer formato de administração

( ) Sim	( ) Não

**C)** Teve grupo controle?

( ) Sim ( ) Não

**O)** O estudo apresenta valores de marcadores inflamatórios (citocinas e outros marcadores) antes e após as intervenções de creatina?

( ) Sim	( ) Não

**S)** O estudo é um ensaio clínico randomizado e controlado?

( ) Sim	( ) Não

**Artigo 511: Effects of acetyl glyceryl ether phosphorylcholine on human platelet function** in vitro**.**

Marcus AJ, Safier LB, Ullman HL, Wong KT, Broekman MJ, Weksler BB, Kaplan KL

**P)** Os participantes possuem mais de 18 anos de idade?

( ) Sim	(X) Não

"The provided sources consistently refer to 'human platelet-rich plasma' and 'human platelets' being studied. However, **no information regarding the age of the human participants or donors, or whether they are over 18 years old, is explicitly stated or can be directly transcribed from the provided text**."

**I)** A intervenção utilizada no estudo é creatina? Vale qualquer formato de administração

( ) Sim	( ) Não

**C)** Teve grupo controle?

( ) Sim ( ) Não

**O)** O estudo apresenta valores de marcadores inflamatórios (citocinas e outros marcadores) antes e após as intervenções de creatina?

( ) Sim	( ) Não

**S)** O estudo é um ensaio clínico randomizado e controlado?

( ) Sim	( ) Não

**Artigo 512: Pyometra in the dog--a pathophysiological investigation. V. The presence of intrahepatic cholestasis and an "acute phase reaction.".**

Børresen B, Skrede S

**P)** Os participantes possuem mais de 18 anos de idade?

( ) Sim	( ) Não [*Excluir*]

**I)** A intervenção utilizada no estudo é creatina? Vale qualquer formato de administração

( ) Sim	(	) Não [*Excluir*]

**C)** Teve grupo controle?

(	) Sim ( ) Não [*Excluir*]

**O)** O estudo apresenta valores de marcadores inflamatórios (citocinas e outros marcadores) antes e após as intervenções de creatina?

( ) Sim	(	) Não [*Excluir*]

**S)** O estudo é um ensaio clínico randomizado e controlado?

(	) Sim	( ) Não

**Artigo 513: Computer-assisted customized antimicrobial dosages.**

Bennett SW, Scott AC

**P)** Os participantes possuem mais de 18 anos de idade?

( ) Sim	( ) Não [*Excluir*]

**I)** A intervenção utilizada no estudo é creatina? Vale qualquer formato de administração

( ) Sim	(	) Não [*Excluir*]

**C)** Teve grupo controle?

(	) Sim ( ) Não [*Excluir*]

**O)** O estudo apresenta valores de marcadores inflamatórios (citocinas e outros marcadores) antes e após as intervenções de creatina?

( ) Sim	(	) Não [*Excluir*]

**S)** O estudo é um ensaio clínico randomizado e controlado?

(	) Sim	( ) Não

**Artigo 514: Metabolic and morphologic brain reactions in shock induced by intracisternal injection of endotoxin.**

Schmahl FW, Betz E, Heckers H, Reinhard U, Schlote W, Urbaschek B

**P)** Os participantes possuem mais de 18 anos de idade?

( ) Sim	( ) Não [*Excluir*]

**I)** A intervenção utilizada no estudo é creatina? Vale qualquer formato de administração

( ) Sim	(	) Não [*Excluir*]

**C)** Teve grupo controle?

(	) Sim ( ) Não [*Excluir*]

**O)** O estudo apresenta valores de marcadores inflamatórios (citocinas e outros marcadores) antes e após as intervenções de creatina?

( ) Sim	(	) Não [*Excluir*]

**S)** O estudo é um ensaio clínico randomizado e controlado?

(	) Sim	( ) Não

**Artigo 515: Prolonged intraaortic balloon pumping in klebsiella-induced hypodynamic shock: cardiopulmonary, hematological, metabolic, and pathological observations.**

Roberts AJ, Hoover EL, Alonso DR, Combes JR, Dineen P, Gay WA Jr, Subramanian VA

**P)** Os participantes possuem mais de 18 anos de idade?

( ) Sim	(X) Não

"Experiments were performed on 20 **adult mongrel dogs** of either sex, weighing between 18 and 22 kg (average weight, 20.4 kg)". The study utilized animals (dogs) as participants, not humans

**I)** A intervenção utilizada no estudo é creatina? Vale qualquer formato de administração

( ) Sim	( ) Não

**C)** Teve grupo controle?

( ) Sim ( ) Não

**O)** O estudo apresenta valores de marcadores inflamatórios (citocinas e outros marcadores) antes e após as intervenções de creatina?

( ) Sim	( ) Não

**S)** O estudo é um ensaio clínico randomizado e controlado?

( ) Sim	( ) Não

**Artigo 516: Plasma changes in endotoxin and anaphylactic shock (ATP, ADP and Creatine phosphorus).**

Jabs CM, Ferrell WJ, Robb HJ

**P)** Os participantes possuem mais de 18 anos de idade?

( ) Sim	(X) Não

"Endotoxin shock was produced in eight **male rabbits** with Difco lyophilized Escherichia coli...". The study was conducted on animals (rabbits), not human participants, thus the concept of being "over 18 years of age" does not apply in the human sense.

**I)** A intervenção utilizada no estudo é creatina? Vale qualquer formato de administração

( ) Sim	( ) Não

**C)** Teve grupo controle?

( ) Sim ( ) Não

**O)** O estudo apresenta valores de marcadores inflamatórios (citocinas e outros marcadores) antes e após as intervenções de creatina?

( ) Sim	( ) Não

**S)** O estudo é um ensaio clínico randomizado e controlado?

( ) Sim	( ) Não

**Artigo 517: Effect of creatinine on various experimentally induced inflammatory models.**

Madan BR, Khanna NK

**P)** Os participantes possuem mais de 18 anos de idade?

( ) Sim	( ) Não [*Excluir*]

**I)** A intervenção utilizada no estudo é creatina? Vale qualquer formato de administração

( ) Sim	(	) Não [*Excluir*]

**C)** Teve grupo controle?

(	) Sim ( ) Não [*Excluir*]

**O)** O estudo apresenta valores de marcadores inflamatórios (citocinas e outros marcadores) antes e após as intervenções de creatina?

( ) Sim	(	) Não [*Excluir*]

**S)** O estudo é um ensaio clínico randomizado e controlado?

(	) Sim	( ) Não

**Artigo 518: Failure of exogenous ATP and creatine phosphate to modify the course of E coli endotoxin shock in the cat.**

McCaig DJ, Parratt JR

**P)** Os participantes possuem mais de 18 anos de idade?

( ) Sim	( ) Não [*Excluir*]

**I)** A intervenção utilizada no estudo é creatina? Vale qualquer formato de administração

( ) Sim	(	) Não [*Excluir*]

**C)** Teve grupo controle?

(	) Sim ( ) Não [*Excluir*]

**O)** O estudo apresenta valores de marcadores inflamatórios (citocinas e outros marcadores) antes e após as intervenções de creatina?

( ) Sim	(	) Não [*Excluir*]

**S)** O estudo é um ensaio clínico randomizado e controlado?

(	) Sim	( ) Não

**Artigo 519: Studies on the anti-inflammatory activity of creatine.**

**P)** Os participantes possuem mais de 18 anos de idade?

( ) Sim	(X) Não

"In this regard, the first studies of the effects of creatine on inflammatory processes were conducted on **rats**".

**I)** A intervenção utilizada no estudo é creatina? Vale qualquer formato de administração

( ) Sim	( ) Não

**C)** Teve grupo controle?

( ) Sim ( ) Não

**O)** O estudo apresenta valores de marcadores inflamatórios (citocinas e outros marcadores) antes e após as intervenções de creatina?

( ) Sim	( ) Não

**S)** O estudo é um ensaio clínico randomizado e controlado?

( ) Sim	( ) Não

**Artigo 520: [Blood energy system in staphylococcal sepsis in nursing infants].**

Pridatko VD

**P)** Os participantes possuem mais de 18 anos de idade?

( ) Sim	( ) Não [*Excluir*]

**I)** A intervenção utilizada no estudo é creatina? Vale qualquer formato de administração

( ) Sim	(	) Não [*Excluir*]

**C)** Teve grupo controle?

(	) Sim ( ) Não [*Excluir*]

**O)** O estudo apresenta valores de marcadores inflamatórios (citocinas e outros marcadores) antes e após as intervenções de creatina?

( ) Sim	(	) Não [*Excluir*]

**S)** O estudo é um ensaio clínico randomizado e controlado?

(	) Sim	( ) Não

**Artigo 521: Early effects of endotoxin on tissue phosphagen levels in skeletal muscle and liver of the dog.**

Myrvold HE, Enger E, Haljamäe H

**P)** Os participantes possuem mais de 18 anos de idade?

( ) Sim	(X) Não

"Mongrel dogs (n=12) of etiher sex (15-30 kg) were used."

**I)** A intervenção utilizada no estudo é creatina? Vale qualquer formato de administração

( ) Sim	( ) Não

**C)** Teve grupo controle?

( ) Sim ( ) Não

**O)** O estudo apresenta valores de marcadores inflamatórios (citocinas e outros marcadores) antes e após as intervenções de creatina?

( ) Sim	( ) Não

**S)** O estudo é um ensaio clínico randomizado e controlado?

( ) Sim	( ) Não

**Artigo 522: Observations on experimental sporotrichosis of the muscle.**

Staib F, Randhawa HS, Blisse A

**P)** Os participantes possuem mais de 18 anos de idade?

( ) Sim	( ) Não [*Excluir*]

**I)** A intervenção utilizada no estudo é creatina? Vale qualquer formato de administração

( ) Sim	(	) Não [*Excluir*]

**C)** Teve grupo controle?

(	) Sim ( ) Não [*Excluir*]

**O)** O estudo apresenta valores de marcadores inflamatórios (citocinas e outros marcadores) antes e após as intervenções de creatina?

( ) Sim	(	) Não [*Excluir*]

**S)** O estudo é um ensaio clínico randomizado e controlado?

(	) Sim	( ) Não

**Artigo 523: Oxyhemoglobin dissociation curve in hemorrhagic and septic shock.**

Valeri CR, Kopriva CJ

**P)** Os participantes possuem mais de 18 anos de idade?

( ) Sim	( ) Não [*Excluir*]

**I)** A intervenção utilizada no estudo é creatina? Vale qualquer formato de administração

( ) Sim	(	) Não [*Excluir*]

**C)** Teve grupo controle?

(	) Sim ( ) Não [*Excluir*]

**O)** O estudo apresenta valores de marcadores inflamatórios (citocinas e outros marcadores) antes e após as intervenções de creatina?

( ) Sim	(	) Não [*Excluir*]

**S)** O estudo é um ensaio clínico randomizado e controlado?

(	) Sim	( ) Não

**Artigo 524: Energy-rich phosphates and glucose metabolism in early endotoxin shock.**

Pappova E, Urbaschek B, Heitmann L, Oroz M, Streit E, Lemeunier A, Lundsgaard-Hansen P

**P)** Os participantes possuem mais de 18 anos de idade?

( ) Sim	(X) Não

"**The experimental series comprised 48 healthy, outbred adult rabbits with an average body weight of 2884 grams** (95% confidence limits, 2784-2984 grams)". O estudo foi realizado em animais (coelhos), não em participantes humanos, portanto, o conceito de "mais de 18 anos de idade" não se aplica no sentido humano.

**I)** A intervenção utilizada no estudo é creatina? Vale qualquer formato de administração

( ) Sim	( ) Não

**C)** Teve grupo controle?

( ) Sim ( ) Não

**O)** O estudo apresenta valores de marcadores inflamatórios (citocinas e outros marcadores) antes e após as intervenções de creatina?

( ) Sim	( ) Não

**S)** O estudo é um ensaio clínico randomizado e controlado?

( ) Sim	( ) Não

**Artigo 525: Erythropoietin release in baboons after renal allotransplantation.**

Mirand EA, Steeves R, Van Zyl JJ, Murphy GP

**P)** Os participantes possuem mais de 18 anos de idade?

( ) Sim	(X) Não

"The present report describes ESF alterations observed in 13 **adult male and female chacma baboons** after renal allotransplantation". The study was conducted on animals (baboons), not human participants, thus the concept of being "over 18 years of age" does not apply in the human sense.

**I)** A intervenção utilizada no estudo é creatina? Vale qualquer formato de administração

( ) Sim	( ) Não

**C)** Teve grupo controle?

( ) Sim ( ) Não

**O)** O estudo apresenta valores de marcadores inflamatórios (citocinas e outros marcadores) antes e após as intervenções de creatina?

( ) Sim	( ) Não

**S)** O estudo é um ensaio clínico randomizado e controlado?

( ) Sim	( ) Não

**Artigo 526: Effects of septic shock on renal function in humans.**

Strauch M, McLaughlin JS, Mansberger A, Young J, Mendonca P, Gray K, Cowley RA

**P)** Os participantes possuem mais de 18 anos de idade?

(X) Sim	( ) Não

"Ten patients with clinical septic shock were studied in the University of Maryland Shock Trauma Unit... **The ages of the patients ranged from 45 to 80 years**...".

**I)** A intervenção utilizada no estudo é creatina? Vale qualquer formato de administração

( ) Sim	(X) Não

"This report deals with the study of changes in renal function occurring over a period of several days in a group of ten patients in septic shock. The purpose of this study was not only to ascertain the effect of septic shock on renal function, but also to investigate renal responses to the resuscitative measures utilized.". O estudo não menciona creatina como uma intervenção.

**C)** Teve grupo controle?

( ) Sim (X) Não

"This report deals with the study of changes in renal function occurring over a period of several days in a group of ten patients in septic shock."

**O)** O estudo apresenta valores de marcadores inflamatórios (citocinas e outros marcadores) antes e após as intervenções de creatina?

( ) Sim	( ) Não

**S)** O estudo é um ensaio clínico randomizado e controlado?

( ) Sim	( ) Não

**Artigo 527: SUBCLINICAL POLYNEUROPATHY IN RENAL INSUFFICIENCY.**

PRESWICK G, JEREMY D

**P)** Os participantes possuem mais de 18 anos de idade?

(X) Sim	( ) Não

"In addition to the first patient (case 1), nineteen others were examined. There were ten males and nine females, whose **ages ranged from 32 to 76 years**.".

**I)** A intervenção utilizada no estudo é creatina? Vale qualquer formato de administração

( ) Sim	(X) Não

The study investigates "SUBCLINICAL POLYNEUROPATHY IN RENAL INSUFFICIENCY" and assesses "nerve-conduction velocity" and "endogenous creatinine clearance". There is no mention of creatine or any form of its administration as an intervention.

**C)** Teve grupo controle?

( ) Sim ( ) Não

**O)** O estudo apresenta valores de marcadores inflamatórios (citocinas e outros marcadores) antes e após as intervenções de creatina?

( ) Sim	( ) Não

**S)** O estudo é um ensaio clínico randomizado e controlado?

( ) Sim	( ) Não

**Artigo 528: PLASMA AND RENAL CLEARANCE OF EXOGENOUS ERYTHROPOIETIN IN THE DOG.**

WEINTRAUB AH, GORDON AS, BECKER EL, CAMISCOLI JF, CONTRERA JF

**P)** Os participantes possuem mais de 18 anos de idade?

( ) Sim	(X) Não

"Female **mongrel dogs** (I I-J 7 kg body wt.) were employed".

**I)** A intervenção utilizada no estudo é creatina? Vale qualquer formato de administração

( ) Sim	( ) Não

**C)** Teve grupo controle?

( ) Sim ( ) Não

**O)** O estudo apresenta valores de marcadores inflamatórios (citocinas e outros marcadores) antes e após as intervenções de creatina?

( ) Sim	( ) Não

**S)** O estudo é um ensaio clínico randomizado e controlado?

( ) Sim	( ) Não

**Artigo 529: HEMODIALYSIS IN THE TREATMENT OF POSTOPERATIVE ACUTE RENAL FAILURE ASSOCIATED WITH JAUNDICE: A REPORT OF THREE CASES.**

LOVETT VF, YAMAUCHI H, PHILLIPPI PJ

**P)** Os participantes possuem mais de 18 anos de idade?

(X) Sim	( ) Não

"Case 1. M. R., **a 42-year-old white man** was first seen for right upper quadrant pain related to fatty food intake which he had for 13 years.".

**I)** A intervenção utilizada no estudo é creatina? Vale qualquer formato de administração

( ) Sim	(X) Não

"These cases are reported in detail, in the hope that they may stimulate a reappraisal of this formidable surgical complication... On 9 April 1962 the patient was hemodialized with the Kolff twin-coil artificial kidney for six hours with definite clinical improvement (Fig. 1)."

**C)** Teve grupo controle?

( ) Sim ( ) Não

**O)** O estudo apresenta valores de marcadores inflamatórios (citocinas e outros marcadores) antes e após as intervenções de creatina?

( ) Sim	( ) Não

**S)** O estudo é um ensaio clínico randomizado e controlado?

( ) Sim	( ) Não

**Artigo 530: INFLUENCE OF X-IRRADIATION OF THE DOG KIDNEY ON ERYTHROPOIETIN PRODUCTION.**

FISHER JW, ROH BL, SHENEP K

**P)** Os participantes possuem mais de 18 anos de idade?

( ) Sim	(X) Não

"Male and female **mongrel dogs** weighing between 8 and 17 kg were used throughout these studies.".

**I)** A intervenção utilizada no estudo é creatina? Vale qualquer formato de administração

( ) Sim	( ) Não

**C)** Teve grupo controle?

( ) Sim ( ) Não

**O)** O estudo apresenta valores de marcadores inflamatórios (citocinas e outros marcadores) antes e após as intervenções de creatina?

( ) Sim	( ) Não

**S)** O estudo é um ensaio clínico randomizado e controlado?

( ) Sim	( ) Não

**Artigo 531: Effects of creatine supplementation in athletes**

IRCT201203063236N3

**P)** Os participantes possuem mais de 18 anos de idade?

( ) Sim	( ) Não [*Excluir*]

**I)** A intervenção utilizada no estudo é creatina? Vale qualquer formato de administração

( ) Sim	(	) Não [*Excluir*]

**C)** Teve grupo controle?

(	) Sim ( ) Não [*Excluir*]

**O)** O estudo apresenta valores de marcadores inflamatórios (citocinas e outros marcadores) antes e após as intervenções de creatina?

( ) Sim	(	) Não [*Excluir*]

**S)** O estudo é um ensaio clínico randomizado e controlado?

(	) Sim	( ) Não

**Artigo 532: Intradialytic Creatine Supplementation**

NCT05148208

**P)** Os participantes possuem mais de 18 anos de idade?

( ) Sim	( ) Não [*Excluir*]

**I)** A intervenção utilizada no estudo é creatina? Vale qualquer formato de administração

( ) Sim	(	) Não [*Excluir*]

**C)** Teve grupo controle?

(	) Sim ( ) Não [*Excluir*]

**O)** O estudo apresenta valores de marcadores inflamatórios (citocinas e outros marcadores) antes e após as intervenções de creatina?

( ) Sim	(	) Não [*Excluir*]

**S)** O estudo é um ensaio clínico randomizado e controlado?

(	) Sim	( ) Não

**Artigo 533: Creatine supplementation reduces plasma levels of pro-inflammatory cytokines and PGE2 after a half-ironman competition**

Bassit, RA, Curi, R, Costa Rosa, LF

**P)** Os participantes possuem mais de 18 anos de idade?

(X) Sim	( ) Não

"The athletes **average age was 40.3 years, range 34–56 years**."

**I)** A intervenção utilizada no estudo é creatina? Vale qualquer formato de administração

(X) Sim	(	) Não

**Creatine supplementation**". Especificamente, "During five days prior to the competition, athletes from the experimental group received **20 g per day of creatine monohydrate divided in two equal doses**." O suplemento foi "supplied to athletes as a ready-to-consume powder mixture containing maltodextrin 50 g."

**C)** Teve grupo controle?

(X) Sim ( ) Não

"Eleven triathletes, each with at least three years experience of participation in this sport were **randomly divided between the control and experimental groups**." O grupo controle ("control group") recebeu "20 g per day of carbohydrate in place of creatine."

**O)** O estudo apresenta valores de marcadores inflamatórios (citocinas e outros marcadores) antes e após as intervenções de creatina?

(X) Sim	(	) Não

"plasma levels of pro-inflammatory cytokines: **Interleukin (IL) 1b and IL-6, Tumor Necrosis Factor a (TNFa), and Interferon a (INFa) and Prostaglandin E2 (PGE2)**". As amostras de sangue foram coletadas "48 h before and 24 and 48 h after competition" para a medição dessas substâncias. Os resultados são apresentados na seção "Results", com as concentrações iniciais e as alterações pós-competição em ambos os grupos.

**S)** O estudo é um ensaio clínico randomizado e controlado?

(X) Sim	( ) Não

"Eleven triathletes... were **randomly divided between the control and experimental groups**" e conduzido como um "**double-blind trial**". Além disso, a seção "Materials and methods" reafirma que "After signing an informed consent form, eleven male athletes were **randomly divided into the control (Pl – n¼ 6) and experimental (Cr – n¼ 5) groups**." e "All experiments were conducted as a **double-blind trial**.".

**Artigo 534: Creatine Supplementation During Resistance Training for People Recovering From Stroke**

NCT03941678

**P)** Os participantes possuem mais de 18 anos de idade?

( ) Sim	( ) Não [*Excluir*]

**I)** A intervenção utilizada no estudo é creatina? Vale qualquer formato de administração

( ) Sim	(	) Não [*Excluir*]

**C)** Teve grupo controle?

(	) Sim ( ) Não [*Excluir*]

**O)** O estudo apresenta valores de marcadores inflamatórios (citocinas e outros marcadores) antes e após as intervenções de creatina?

( ) Sim	(	) Não [*Excluir*]

**S)** O estudo é um ensaio clínico randomizado e controlado?

(	) Sim	( ) Não

**Artigo 535: Does oral creatine supplementation enhance recovery from a worsening of chronic bronchitis?**

ISRCTN22287730

**P)** Os participantes possuem mais de 18 anos de idade?

( ) Sim	( ) Não [*Excluir*]

**I)** A intervenção utilizada no estudo é creatina? Vale qualquer formato de administração

( ) Sim	(	) Não [*Excluir*]

**C)** Teve grupo controle?

(	) Sim ( ) Não [*Excluir*]

**O)** O estudo apresenta valores de marcadores inflamatórios (citocinas e outros marcadores) antes e após as intervenções de creatina?

( ) Sim	(	) Não [*Excluir*]

**S)** O estudo é um ensaio clínico randomizado e controlado?

(	) Sim	( ) Não

**Artigo 536: Evaluation of Muscle Electrical Stimulation and Creatine Supplementation in Renal Hemodialysis Patients**

RBR-98wzgn

**P)** Os participantes possuem mais de 18 anos de idade?

( ) Sim	( ) Não [*Excluir*]

**I)** A intervenção utilizada no estudo é creatina? Vale qualquer formato de administração

( ) Sim	(	) Não [*Excluir*]

**C)** Teve grupo controle?

(	) Sim ( ) Não [*Excluir*]

**O)** O estudo apresenta valores de marcadores inflamatórios (citocinas e outros marcadores) antes e após as intervenções de creatina?

( ) Sim	(	) Não [*Excluir*]

**S)** O estudo é um ensaio clínico randomizado e controlado?

(	) Sim	( ) Não

**Artigo 537: Creatine supplementation does not reduce muscle damage or enhance recovery from resistance exercise**

Rawson, ES, Conti, MP, Miles, MP

**P)** Os participantes possuem mais de 18 anos de idade?

(X) Sim	( ) Não

"Twenty-two healthy, weight-trained men (**19–27 years**) ingested either creatine or a placebo for 10 days". A Tabela 1 também mostra a idade média dos participantes: "Age (y) **22.2 ± 1.3**"

**I)** A intervenção utilizada no estudo é creatina? Vale qualquer formato de administração

(X) Sim	(	) Não

"The purpose of this study was to examine the effects of short-term **creatine supplementation** on markers of muscle damage". Em relação ao formato de administração, o estudo detalha: "During the loading phase, each participant **ingested 0.3 g·kg 1 of body weight per day of encapsulated creatine**... for 5 days. Participants were instructed to ingest the supplements with food in 3 equal doses per day. ... Following the loading phase, participants **ingested 0.03 g·kg 1 of body weight per day of creatine** or placebo for 5 days to maintain increased muscle creatine levels"

**C)** Teve grupo controle?

(X) Sim ( ) Não

"Twenty-two healthy, weight-trained men... ingested either creatine or a **placebo** for 10 days". Adicionalmente, "Participants were randomly placed into either a creatine or **placebo group**, and supplements were administered in a **double-blind, placebo-controlled manner**"

**O)** O estudo apresenta valores de marcadores inflamatórios (citocinas e outros marcadores) antes e após as intervenções de creatina?

(X) Sim	(	) Não

"**C-re-active protein**" (Proteína C-reativa), que é um marcador inflamatório: "The purpose of this study was to examine the effects of short-term creatine supple-mentation on markers of muscle damage (i.e., strength, range of motion, muscle soreness, muscle serum protein activity, **C-re-active protein**)". As avaliações foram realizadas "pre-exercise and during a 5-day follow up", e os dados para a proteína C-reativa são apresentados antes e após a intervenção.

**S)** O estudo é um ensaio clínico randomizado e controlado?

(X) Sim	( ) Não

"Participants were **randomly placed** into either a creatine or placebo group, and supplements were administered in a **double-blind, placebo-controlled manner**". Isso indica que se trata de um ensaio clínico randomizado e controlado.

**Artigo 538: Evaluation of creatine supplementation on long-term Covid-related fatigue (fatigue study)**

RBR-3pf736g

**P)** Os participantes possuem mais de 18 anos de idade?

( ) Sim	( ) Não [*Excluir*]

**I)** A intervenção utilizada no estudo é creatina? Vale qualquer formato de administração

( ) Sim	(	) Não [*Excluir*]

**C)** Teve grupo controle?

(	) Sim ( ) Não [*Excluir*]

**O)** O estudo apresenta valores de marcadores inflamatórios (citocinas e outros marcadores) antes e após as intervenções de creatina?

( ) Sim	(	) Não [*Excluir*]

**S)** O estudo é um ensaio clínico randomizado e controlado?

(	) Sim	( ) Não

**Artigo 539: Short-term creatine supplementation reduces the malnutrition-inflammation score and attenuates lean body mass loss in hemodialysis patients**

Pimentel, G, Marini, A, Motobu, R, Freitas, A, Mota, J, Lira, F, Laviano, A, Wall, B

**P)** Os participantes possuem mais de 18 anos de idade?

( ) Sim	( ) Não [*Excluir*]

**I)** A intervenção utilizada no estudo é creatina? Vale qualquer formato de administração

( ) Sim	(	) Não [*Excluir*]

**C)** Teve grupo controle?

(	) Sim ( ) Não [*Excluir*]

**O)** O estudo apresenta valores de marcadores inflamatórios (citocinas e outros marcadores) antes e após as intervenções de creatina?

( ) Sim	(	) Não [*Excluir*]

**S)** O estudo é um ensaio clínico randomizado e controlado?

(	) Sim	( ) Não

**Artigo 540: Effects of dry cupping therapy and creatine supplementation on inflammatory and cardiovascular responses to the Wingate test in handball players**

Irandoust, K, Ben Saad, H, Mohammadgholiha, F, Taheri, M, Dergaa, I

**P)** Os participantes possuem mais de 18 anos de idade?

(X) Sim	( ) Não

"Twelve participants from the males’ handball team of Qazvin province (Iran) **between the ages of 18 to 25 years** voluntarily participated."

**I)** A intervenção utilizada no estudo é creatina? Vale qualquer formato de administração

(X) Sim	(	) Não

"creatine supplementation (CS)". A administração específica foi detalhada: "In players with **CS condition, 60 g of creatine was consumed per day in three consecutive days prior to the study (3 meals of 20 g in morning, noon, and night)**."

**C)** Teve grupo controle?

(X) Sim ( ) Não

"Players were studied in four conditions: DCT; CS; CS+DCT, **control**."

**O)** O estudo apresenta valores de marcadores inflamatórios (citocinas e outros marcadores) antes e após as intervenções de creatina?

(X) Sim	(	) Não

"Blood [**lactate-dehydrogenase (LDH), creatine phosphokinase (CK)**] was drawn pre- and immediately post- the Wingate test". CK e LDH são descritos no artigo como "indicators of cell damage assessment" e estão diretamente associados à inflamação e lesão muscular após o exercício. Além disso, a Tabela 1 é intitulada "Results of analysis of covariance for four test conditions for systolic and diastolic blood pressure, heart-rate, creatinine phosphokinase, and lactate dehydrogenase (n=12)" e os resultados são para "inflammatory and cardiovascular data"

**S)** O estudo é um ensaio clínico randomizado e controlado?

(X) Sim	( ) Não

"In this **quasi-experimental study**, 12 male handball young players were selected in a **crossover design**". E novamente, "A **non-randomized quasi-experimental cross-over study design** was used.". Embora tenha condições de controle (onde os participantes servem como seus próprios controles devido ao design crossover) e cego, ele não é classificado como um ensaio *randomizado* no sentido tradicional de alocação de grupos independentes.

**Artigo 541: Safety and efficacy of creatine supplementation in juvenile dermatomyositis: a** randomized **double-blind** placebo**-controlled cross-over** trial

Sallum, A, Solis, M, Hayashi, AP, Artioli, G, Sapienza, M, Otaduy, M, Pinto, A, Silva, C, Pereira, R, Gualano, B

**P)** Os participantes possuem mais de 18 anos de idade?

( ) Sim	(X) Não

"The sample consisted of patients aged **7–21 years** with active (n 5 5) and inactive (n 5 10) disease".

**I)** A intervenção utilizada no estudo é creatina? Vale qualquer formato de administração

( ) Sim	( ) Não

**C)** Teve grupo controle?

( ) Sim ( ) Não

**O)** O estudo apresenta valores de marcadores inflamatórios (citocinas e outros marcadores) antes e após as intervenções de creatina?

( ) Sim	( ) Não

**S)** O estudo é um ensaio clínico randomizado e controlado?

( ) Sim	( ) Não

**Artigo 542: The Combined Effect of Dairy and Exercise on Bone and Inflammation**

NCT03615989

**P)** Os participantes possuem mais de 18 anos de idade?

( ) Sim	( ) Não

**I)** A intervenção utilizada no estudo é creatina? Vale qualquer formato de administração

( ) Sim	(	) Não

**C)** Teve grupo controle?

(	) Sim ( ) Não

**O)** O estudo apresenta valores de marcadores inflamatórios (citocinas e outros marcadores) antes e após as intervenções de creatina?

( ) Sim	(	) Não

**S)** O estudo é um ensaio clínico randomizado e controlado?

(	) Sim	( ) Não

**Artigo 543: Effects of active recoveries with and without creatine on inflammatory markers and physical performance in young women after bouts of maximum swimming**

Barzanjeh, SP, Roshan, VD

**P)** Os participantes possuem mais de 18 anos de idade?

(X) Sim	( ) Não

"young women"

**I)** A intervenção utilizada no estudo é creatina? Vale qualquer formato de administração

(X) Sim	( ) Não

"Effects of active recoveries **with and without creatine supplementation** on inflammatory markers and physical performance in young women after bouts of maximum swimming".

**C)** Teve grupo controle?

(X) Sim ( ) Não

The title implies a control condition by mentioning "**with and without creatine supplementation**".

**O)** O estudo apresenta valores de marcadores inflamatórios (citocinas e outros marcadores) antes e após as intervenções de creatina?

( ) Sim	(X) Não

"with and without creatine supplementation". However, the provided excerpts do not explicitly state whether the study was "randomized".

**S)** O estudo é um ensaio clínico randomizado e controlado?

( ) Sim	( ) Não

**Artigo 544: S100A8/A9, Exercise, and Creatine in Knee Osteoarthritis**

Peeler, J, Zhang, P, Cornish, S, Ghavami, S

**P)** Os participantes possuem mais de 18 anos de idade?

( ) Sim	( ) Não

**I)** A intervenção utilizada no estudo é creatina? Vale qualquer formato de administração

( ) Sim	(	) Não

**C)** Teve grupo controle?

(	) Sim ( ) Não

**O)** O estudo apresenta valores de marcadores inflamatórios (citocinas e outros marcadores) antes e após as intervenções de creatina?

( ) Sim	(	) Não

**S)** O estudo é um ensaio clínico randomizado e controlado?

(	) Sim	( ) Não

**Artigo 545: Resistance Exercise and Creatine in Colorectal Cancer**

NCT06420726

**P)** Os participantes possuem mais de 18 anos de idade?

( ) Sim	( ) Não

**I)** A intervenção utilizada no estudo é creatina? Vale qualquer formato de administração

( ) Sim	(	) Não

**C)** Teve grupo controle?

(	) Sim ( ) Não

**O)** O estudo apresenta valores de marcadores inflamatórios (citocinas e outros marcadores) antes e após as intervenções de creatina?

( ) Sim	(	) Não

**S)** O estudo é um ensaio clínico randomizado e controlado?

(	) Sim	( ) Não

**Artigo 546: Creatine, Exercise and Inflammatory Markers in Knee Osteoarthritis**

NCT02290275

**P)** Os participantes possuem mais de 18 anos de idade?

( ) Sim	( ) Não

**I)** A intervenção utilizada no estudo é creatina? Vale qualquer formato de administração

( ) Sim	(	) Não

**C)** Teve grupo controle?

(	) Sim ( ) Não

**O)** O estudo apresenta valores de marcadores inflamatórios (citocinas e outros marcadores) antes e após as intervenções de creatina?

( ) Sim	(	) Não

**S)** O estudo é um ensaio clínico randomizado e controlado?

(	) Sim	( ) Não

**Artigo 547: The effect of intradialytic supplementation in chronic hemodialysis patients; a pilot study**

NL-OMON51287

**P)** Os participantes possuem mais de 18 anos de idade?

( ) Sim	( ) Não

**I)** A intervenção utilizada no estudo é creatina? Vale qualquer formato de administração

( ) Sim	(	) Não

**C)** Teve grupo controle?

(	) Sim ( ) Não

**O)** O estudo apresenta valores de marcadores inflamatórios (citocinas e outros marcadores) antes e após as intervenções de creatina?

( ) Sim	(	) Não

**S)** O estudo é um ensaio clínico randomizado e controlado?

(	) Sim	( ) Não

**Artigo 548: The Effect of Creatine Monohydrate on Persistent Post-concussive Symptoms**

NCT05562232

**P)** Os participantes possuem mais de 18 anos de idade?

( ) Sim	( ) Não

**I)** A intervenção utilizada no estudo é creatina? Vale qualquer formato de administração

( ) Sim	(	) Não

**C)** Teve grupo controle?

(	) Sim ( ) Não

**O)** O estudo apresenta valores de marcadores inflamatórios (citocinas e outros marcadores) antes e após as intervenções de creatina?

( ) Sim	(	) Não

**S)** O estudo é um ensaio clínico randomizado e controlado?

(	) Sim	( ) Não

**Artigo 549: Effect of Creatine and Caffeine on Muscle Performance**

NCT04290819

**P)** Os participantes possuem mais de 18 anos de idade?

( ) Sim	( ) Não

**I)** A intervenção utilizada no estudo é creatina? Vale qualquer formato de administração

( ) Sim	(	) Não

**C)** Teve grupo controle?

(	) Sim ( ) Não

**O)** O estudo apresenta valores de marcadores inflamatórios (citocinas e outros marcadores) antes e após as intervenções de creatina?

( ) Sim	(	) Não

**S)** O estudo é um ensaio clínico randomizado e controlado?

(	) Sim	( ) Não

**Artigo 550: Effect of Creatine Monohydrate on Persistent Post-concussive Symptoms**

NCT06644131,

**P)** Os participantes possuem mais de 18 anos de idade?

( ) Sim	( ) Não

**I)** A intervenção utilizada no estudo é creatina? Vale qualquer formato de administração

( ) Sim	(	) Não

**C)** Teve grupo controle?

(	) Sim ( ) Não

**O)** O estudo apresenta valores de marcadores inflamatórios (citocinas e outros marcadores) antes e após as intervenções de creatina?

( ) Sim	(	) Não

**S)** O estudo é um ensaio clínico randomizado e controlado?

(	) Sim	( ) Não

**Artigo 551: Interaction of nutrition and exercise on bone and muscle**

Laskou, F., Dennison, E

**P)** Os participantes possuem mais de 18 anos de idade?

( ) Sim	(X) Não

"This article is a **short opinion piece** and has not been through the journal’s standard peer review process, but has been reviewed by the Editorial Board for accuracy before publication."

**I)** A intervenção utilizada no estudo é creatina? Vale qualquer formato de administração

( ) Sim	( ) Não

**C)** Teve grupo controle?

( ) Sim ( ) Não

**O)** O estudo apresenta valores de marcadores inflamatórios (citocinas e outros marcadores) antes e após as intervenções de creatina?

( ) Sim	( ) Não

**S)** O estudo é um ensaio clínico randomizado e controlado?

( ) Sim	( ) Não

**Artigo 552: Creatine supplementation with exercise reduces α-synuclein oligomerization and necroptosis in Parkinson's disease** mouse **model**

Leem, Y.-H., Park, J.-S., Park, J.-E., Kim, D.-Y., Kim, H.-S.

**P)** Os participantes possuem mais de 18 anos de idade?

( ) Sim	(X) Não

"In this study, we investigated the effects of creatine supplementation and exercise in the subacute 1-methyl-4-phenyl-1,2,3,6-tetrahydropyridine (MPTP) **mouse model** of PD". "The **mice** were treated with MPTP/P for 5 days and subsequently received creatine and/or exercise intervention for 4 weeks".

**I)** A intervenção utilizada no estudo é creatina? Vale qualquer formato de administração

( ) Sim	( ) Não

**C)** Teve grupo controle?

( ) Sim ( ) Não

**O)** O estudo apresenta valores de marcadores inflamatórios (citocinas e outros marcadores) antes e após as intervenções de creatina?

( ) Sim	( ) Não

**S)** O estudo é um ensaio clínico randomizado e controlado?

( ) Sim	( ) Não

**Artigo 553: Creatine in t cell antitumor immunity and cancer immunotherapy**

Li, B., Yang, L.

**P)** Os participantes possuem mais de 18 anos de idade?

( ) Sim	(X) Não

"Creatine supplementation has been shown to enhance antitumor T cell immunity in multiple preclinical **mouse tumor models** and, importantly, to synergize with other cancer immunotherapy modalities, such as the PD-1/PD-L1 blockade therapy, to improve antitumor efficacy." "In a **mouse** B16-OVA melanoma model, creatine supplementation either through i.p. injection or through oral administration effectively suppressed tumor growth..." "In another **mouse** syngeneic MC38 colon cancer model, animals receiving the creatine supplementation also had significantly reduced tumor growth compared to control animals."

**I)** A intervenção utilizada no estudo é creatina? Vale qualquer formato de administração

( ) Sim	( ) Não

**C)** Teve grupo controle?

( ) Sim ( ) Não

**O)** O estudo apresenta valores de marcadores inflamatórios (citocinas e outros marcadores) antes e após as intervenções de creatina?

( ) Sim	( ) Não

**S)** O estudo é um ensaio clínico randomizado e controlado?

( ) Sim	( ) Não

**Artigo 554: Reprogramming alternative macrophage polarization by GATM-mediated endogenous creatine synthesis: A potential target for HDM-induced asthma treatment**

Yu, L., Wang, L., Hu, G., Ren, L., Qiu, C., Li, S., Zhou, X., Chen, S., Chen, R.

**P)** Os participantes possuem mais de 18 anos de idade?

( ) Sim	(X) Não

"Animals and establishment of a mouse model with asthma. **Balb/c mice (6–8 weeks; GemPharmatech Co., Ltd, China)** were sensitized by injections (s.c.) of HDM extract (50 mg in 100".

**I)** A intervenção utilizada no estudo é creatina? Vale qualquer formato de administração

( ) Sim	( ) Não

**C)** Teve grupo controle?

( ) Sim ( ) Não

**O)** O estudo apresenta valores de marcadores inflamatórios (citocinas e outros marcadores) antes e após as intervenções de creatina?

( ) Sim	( ) Não

**S)** O estudo é um ensaio clínico randomizado e controlado?

( ) Sim	( ) Não

**Artigo 555: Creatine supplementation in largemouth bass (Micropterus salmoides) diets: Improving intestinal health and alleviating enteritis**

Yu, H., Nie, Y., Zhang, B., Xue, J., Xue, K., Huang, X., Zhang, X.

**P)** Os participantes possuem mais de 18 anos de idade?

( ) Sim	(X) Não

"In this study, three feeds with varying creatine concentrations (0 %, 0.5 %, and 4 %, labeled CR0, CR0.5, and CR4, respectively) were formulated and administered to **juvenile largemouth bass (Micropterus salmoides)** for 8 weeks". "Healthy and energetic **largemouth bass juveniles** were obtained...". "At the end of the temporary rearing, **360 juvenile largemouth bass (3.35 ± 0.33 g)** were randomly assigned to 9 culture tanks...".

**I)** A intervenção utilizada no estudo é creatina? Vale qualquer formato de administração

( ) Sim	( ) Não

**C)** Teve grupo controle?

( ) Sim ( ) Não

**O)** O estudo apresenta valores de marcadores inflamatórios (citocinas e outros marcadores) antes e após as intervenções de creatina?

( ) Sim	( ) Não

**S)** O estudo é um ensaio clínico randomizado e controlado?

( ) Sim	( ) Não

**Artigo 556: Creatine supplementation enhances anti-tumor immunity by promoting adenosine triphosphate production in macrophages**

Peng, Z., Saito, S.

**P)** Os participantes possuem mais de 18 anos de idade?

( ) Sim	(X) Não

"To examine the effects of creatine supplementation on the immune response, we conducted an experiment using a **mouse tumor model** (Figure 1A). The **mice** were treated with either saline or creatine daily from day -7 to +14...". Additionally, "Experimental design of the **murine tumor model**. The **WT (C57BL/6) mice** received i.p. injection of saline (control) or creatine (500 mg/kg) daily from day -7 to +14 days".

**I)** A intervenção utilizada no estudo é creatina? Vale qualquer formato de administração

( ) Sim	( ) Não

**C)** Teve grupo controle?

( ) Sim ( ) Não

**O)** O estudo apresenta valores de marcadores inflamatórios (citocinas e outros marcadores) antes e após as intervenções de creatina?

( ) Sim	( ) Não

**S)** O estudo é um ensaio clínico randomizado e controlado?

( ) Sim	( ) Não

**Artigo 557: Exercise effects in patients with adult idiopathic inflammatory myopathies**

Alexanderson, H.

**P)** Os participantes possuem mais de 18 anos de idade?

(X) Sim	( ) Não

"Exercise effects in patients with **adult** idiopathic inflammatory myopathies".

**I)** A intervenção utilizada no estudo é creatina? Vale qualquer formato de administração

(X) Sim	( ) Não

"As patients with myositis have reduced phosphocreatine (PCr) and ATP content in skeletal muscle, a recent multicenter, double-blinded, randomized controlled trial aimed at evaluating the effects of **creatine supplement-ation** in addition to physical exercise in patients with chronic polymyositis and dermatomyositis".

**C)** Teve grupo controle?

(X) Sim ( ) Não

"Eighteen patients were randomized to the creatine supplemen-tation group and 17 to the **control group** following a screening visit". Additionally, the review refers to a "six-month, **double-blind, randomized, placebo-controlled trial**", indicating the presence of a control group receiving placebo.

**O)** O estudo apresenta valores de marcadores inflamatórios (citocinas e outros marcadores) antes e após as intervenções de creatina?

( ) Sim	(X) Não

While the article discusses creatine supplementation and mentions "CPK" (creatine phosphokinase) as a safety outcome in reviewed studies, it **does not explicitly present values or changes in inflammatory cytokines** (like IL-1b, IL-6, TNF-a, INF-a, or PGE2) specifically attributed to creatine interventions before and after interventions within its results or discussion sections. It primarily focuses on muscle function, strength, and fatigue outcomes related to exercise and creatine.

**S)** O estudo é um ensaio clínico randomizado e controlado?

( ) Sim	( ) Não

**Artigo 558: The potential role of creatine in vascular health**

Clarke, H., Hickner, R.C., Ormsbee, M.J.

**P)** Os participantes possuem mais de 18 anos de idade?

(X) Sim	( ) Não

The article, being a review, synthesizes findings from multiple studies. It explicitly states that one study included "forty-nine vegan subjects aged **between 20 and 45 years**". It also generally characterizes the subjects in the clinical trials reviewed as "relatively **young, healthy individuals**", and notes that the AHA reported "more than **43.7 million adults aged >60 years**" for a general context on CVD.

**I)** A intervenção utilizada no estudo é creatina? Vale qualquer formato de administração

(X) Sim	( ) Não

"The Potential Role of **Creatine** in Vascular Health". The review explicitly states its focus: "Following an extensive literature search, we found only four clinical studies that reported investigating the direct impact of **creatine supplementation** on vascular health and function". Various administration protocols for creatine supplementation are discussed in the context of the reviewed studies, such as "creatine and placebo (dextrose) supplementation protocols consisted of **20 g/day for the first 5 days, followed by 23 days of 10 g/day**".

**C)** Teve grupo controle?

(X) Sim ( ) Não

The review highlights several studies that included control groups. For example, it mentions that Arciero et al. conducted a "randomized, double-blind, **placebo-controlled study**" where participants were allocated to groups including "creatine only, creatine + resistance training, and **placebo + resistance training**". Similarly, Sanchez-Gonzalez et al. conducted a "**double-blind placebo-controlled study**" comparing creatine supplementation to a "maltodextrin **placebo**", and Van Bavel et al. used a "**single-blinded, randomized study**" with a "creatine group" and a "**placebo group**".

**O)** O estudo apresenta valores de marcadores inflamatórios (citocinas e outros marcadores) antes e após as intervenções de creatina?

( ) Sim	(X) Não

While the article discusses the **potential** for creatine to influence factors like "reducing inflammation" and cites a study by Nomura et al. on its "Anti-inflammatory activity... in vitro", the sections detailing the **clinical studies investigating creatine's direct impact on vascular health** (Sections 3.1 and 3.2) **do not report specific values or changes in inflammatory cytokines** (like IL-1b, IL-6, TNF-a, INF-a, or PGE2) before and after creatine interventions. The outcomes primarily focused on vascular function (blood flow, arterial stiffness, microvascular density and reactivity), heart rate, and lipid profiles (total cholesterol, LDL-C, HDL-C, and homocysteine). Homocysteine is a marker associated with vascular health and inflammation but is not typically categorized as a cytokine.

**S)** O estudo é um ensaio clínico randomizado e controlado?

( ) Sim	( ) Não

**Artigo 559: State-of-the-Art and Future Challenges for Nutritional Interventions in Facioscapulohumeral Dystrophy: A Narrative Review**

Quintiero, V., Crisafulli, O., Diotti, D., Tupler, R., Negro, M., Lavaselli, E., D’Antona, G.

**P)** Os participantes possuem mais de 18 anos de idade?

(X) Sim	( ) Não

The review summarizes various studies, including one by Passerieux et al. where participants had a "mean age of **42.4 ± 10.2 years**" and another by Andersen et al. with participants in the "range age **19–65 years**".

**I)** A intervenção utilizada no estudo é creatina? Vale qualquer formato de administração

(X) Sim	( ) Não

The review explicitly states, "Specifically, we identified three intervention studies involving antioxidant and anti-inflammatory supplements [44–46], one study on post-exercise protein–carbohydrate supplementation, and **one on creatine monohydrate supplementation**.".

**C)** Teve grupo controle?

(X) Sim ( ) Não

The review discusses a study on creatine monohydrate supplementation by Walter et al.. The full citation for this study, as referenced in the article, specifies it was "**A double-blind, placebo-controlled clinical study**". The use of a placebo implies the presence of a control group.

**O)** O estudo apresenta valores de marcadores inflamatórios (citocinas e outros marcadores) antes e após as intervenções de creatina?

( ) Sim	(X) Não

The review's summary of the creatine monohydrate supplementation study by Walter et al. indicates its focus was on "Muscle strength deficit" and reported "no significant improvements".

**S)** O estudo é um ensaio clínico randomizado e controlado?

( ) Sim	( ) Não

**Artigo 560: Fundamental role for the creatine kinase pathway in protection from** murine **colitis**

Hall, C.H.T., Lanis, J.M., Dowdell, A.S., Murphy, E.M., Bhagavatula, G., Neuhart, R.M., Vijaya Sai, K.Y., Colgan, S.P.

**P)** Os participantes possuem mais de 18 anos de idade?

( ) Sim	(X) Não

The study focuses on "murine colitis" and the "Methods and Materials" section explicitly states, "**Mouse lines: All wild type littermates that were all bred in house.**". This indicates the participants were mice, not human subjects aged over 18 years.

**I)** A intervenção utilizada no estudo é creatina? Vale qualquer formato de administração

( ) Sim	( ) Não

**C)** Teve grupo controle?

( ) Sim ( ) Não

**O)** O estudo apresenta valores de marcadores inflamatórios (citocinas e outros marcadores) antes e após as intervenções de creatina?

( ) Sim	( ) Não

**S)** O estudo é um ensaio clínico randomizado e controlado?

( ) Sim	( ) Não

**Artigo 561: Creatine supplementation upregulates mTORC1 signaling and markers of synaptic plasticity in the dentate gyrus while ameliorating LPS-induced cognitive impairment in female** rats

Mao, X., Kelty, T.J., Kerr, N.R., Childs, T.E., Roberts, M.D., Booth, F.W.

**P)** Os participantes possuem mais de 18 anos de idade?

( ) Sim	(X) Não

The "Materials and Methods" section explicitly states, "**Female Wistar rats (150–200 g, 49 days of age)** were bred at the University of Missouri". This indicates that the participants were rats, not human subjects aged over 18 years.

**I)** A intervenção utilizada no estudo é creatina? Vale qualquer formato de administração

( ) Sim	( ) Não

**C)** Teve grupo controle?

( ) Sim ( ) Não

**O)** O estudo apresenta valores de marcadores inflamatórios (citocinas e outros marcadores) antes e após as intervenções de creatina?

( ) Sim	( ) Não

**S)** O estudo é um ensaio clínico randomizado e controlado?

( ) Sim	( ) Não

**Artigo 562: Creatine for exercise and sports performance, with recovery considerations for healthy populations**

Wax, B., Kerksick, C.M., Jagim, A.R., Mayo, J.J., Lyons, B.C., Kreider, R.B.

**P)** Os participantes possuem mais de 18 anos de idade?

( ) Sim	(X) Não

The article is a review that summarizes findings from various studies. It explicitly states that some of the reviewed studies included "**junior competitive swimmers**" and highlights that one such study "**was one of the first to use adolescent athletes**".

**I)** A intervenção utilizada no estudo é creatina? Vale qualquer formato de administração

( ) Sim	( ) Não

**C)** Teve grupo controle?

( ) Sim ( ) Não

**O)** O estudo apresenta valores de marcadores inflamatórios (citocinas e outros marcadores) antes e após as intervenções de creatina?

( ) Sim	( ) Não

**S)** O estudo é um ensaio clínico randomizado e controlado?

( ) Sim	( ) Não

**Artigo 563: Body composition in patients with rheumatoid arthritis: a narrative** literature review

Letarouilly, J.-G., Flipo, R.-M., Cortet, B., Tournadre, A., Paccou, J.

**P)** Os participantes possuem mais de 18 anos de idade?

(X) Sim	( ) Não

"Searches were restricted to **adults ⩾18 years old** and human studies in the English language." Additionally, the reviewed studies refer to participants with "mean age: ~60 years old", "mean age of women: 58.3 years; mean age of men: 60.7 years", and "Mean age was 61 ± 7 years".

**I)** A intervenção utilizada no estudo é creatina? Vale qualquer formato de administração

(X) Sim	( ) Não

The review explicitly mentions and discusses studies where "creatine supplementation" was an intervention. For example, it states: "In the few studies published, (a) **creatine supplementation** and progressive resistance training induce a slight and temporary increase in lean mass". It further details: "Wilkinson et al. investigated whether anabolic nutritional supplementation could increase ALM. The participants were randomized to receive a drink containing either **supplementary creatine** (n = 15) or placebo (n = 20) for 12 weeks."

**C)** Teve grupo controle?

(X) Sim ( ) Não

The review describes studies that included control groups. Specifically, in the context of creatine supplementation, it states: "The participants were randomized to receive a drink containing either supplementary creatine (n = 15) or **placebo** (n = 20) for 12 weeks." The article also notes the inclusion and analysis of "randomised controlled trials"

**O)** O estudo apresenta valores de marcadores inflamatórios (citocinas e outros marcadores) antes e após as intervenções de creatina?

( ) Sim	(X) Não

While the review acknowledges that rheumatoid arthritis is characterized by "systemic inflammation" and discusses the impact of other treatments (like TNFα inhibitors and tocilizumab) on body composition and related inflammatory aspects, the section specifically detailing the "nutritional intervention" with creatine (Section titled "Nutritional intervention") **does not report values or changes in inflammatory markers (such as cytokines or other specific inflammatory markers) before and after creatine supplementation.** The outcomes described for creatine intervention were primarily "ALM" (appendicular lean mass), "muscle strength and physical performance".

**S)** O estudo é um ensaio clínico randomizado e controlado?

( ) Sim	( ) Não

**Artigo 564: Short-term creatine supplementation changes protein metabolism signaling in hindlimb suspension**

Marzuca-Nassr, G.N., Fortes, M.A.S., Guimarães-Ferreira, L., Murata, G.M., Vitzel, K.F., Vasconcelos, D.A.A., Bassit, R.A., Curi, R.

**P)** Os participantes possuem mais de 18 anos de idade?

( ) Sim	(X) Não

"Material and Methods" section, specifically under "Animals", states: "**Eight-week-old male Wistar rats were obtained from the Animal Facility of the Department of Physiology and Biophysics, Institute of Biomedical Sciences, University of São Paulo.**".

**I)** A intervenção utilizada no estudo é creatina? Vale qualquer formato de administração

( ) Sim	( ) Não

**C)** Teve grupo controle?

( ) Sim ( ) Não

**O)** O estudo apresenta valores de marcadores inflamatórios (citocinas e outros marcadores) antes e após as intervenções de creatina?

( ) Sim	( ) Não

**S)** O estudo é um ensaio clínico randomizado e controlado?

( ) Sim	( ) Não

**Artigo 565: Effects of resistance exercise with and without creatine supplementation on gene expression and cell signaling in human skeletal muscle**

Deldicque, L., Atherton, P., Patel, R., Theisen, D., Nielens, H., Rennie, M.J., Francaux, M.

**P)** Os participantes possuem mais de 18 anos de idade?

(X) Sim	( ) Não

"Nine healthy **young men (21.7 ± 0.55 y**...)".

**I)** A intervenção utilizada no estudo é creatina? Vale qualquer formato de administração

(X) Sim	( ) Não

"To test the hypothesis that **creatine supplementation** would enhance the anabolic responses of muscle cell signaling and gene expression to exercise, we studied nine subjects who received either **creatine or a placebo** (maltodextrin) for 5 days in a double-blind fashion". Furthermore, the methods detail, "one group (n = 5) received 21 g (3 × 7 g) of **oral creatine monohydrate** per day for 5 days".

**C)** Teve grupo controle?

(X) Sim ( ) Não

The abstract mentions that subjects "received either creatine or a **placebo (maltodextrin)**". The methods further specify that participants "were randomly divided into two groups: one group (n = 5) received ... oral creatine monohydrate ... whereas the second group (n = 4) received a **placebo (maltodextrin** 3 × 7 g/day)". This indicates the presence of a control group that received a placebo. The study also used a "crossover" design, where each participant eventually served as their own control.

**O)** O estudo apresenta valores de marcadores inflamatórios (citocinas e outros marcadores) antes e após as intervenções de creatina?

(X) Sim	( ) Não

The study investigated inflammatory markers, specifically stating, "mRNA for ... **interleukin-6 were up-regulated (60–350%; P < 0.05**); the phosphorylation state of p38 both in the sarcoplasm and nucleus were increased (12- and 25-fold, respectively; both P < 0.05)" immediately after exercise. The results further detail that "IL-6 (
350%; P < 0.05) were observed" immediately after exercise and "all returned to preexercise values by 24 or 72 h postexercise". The study measured changes in IL-6 mRNA, which is a pro-inflammatory cytokine, and its regulation in response to exercise and creatine supplementation.

**S)** O estudo é um ensaio clínico randomizado e controlado?

(X) Sim	( ) Não

The study design is explicitly described as a "**double-blind crossover study**". Participants "were **randomly divided into two groups**", and treatments were "crossed over for the second trial" after a washout period.

**Artigo 566: Creatine uptake regulates CD8 T cell antitumor immunity**

Di Biase, S., Ma, X., Wang, X., Yu, J., Wang, Y.-C., Smith, D.J., Zhou, Y., Li, Z., Kim, Y.J., Clarke, N., To, A., Yang, L.

**P)** Os participantes possuem mais de 18 anos de idade?

( ) Sim	(X) Não

The study explicitly states that it investigated "tumor-infiltrating immune cells in **mice**". It details experiments conducted on "**CrT-KO mice**" and "**WT BoyJ mice**". The "Mouse tumor models" section further confirms the use of "**B16-OVA murine melanoma cells**" and "**MC38 murine colon adenocarcinoma cells**" in mice.

**I)** A intervenção utilizada no estudo é creatina? Vale qualquer formato de administração

( ) Sim	( ) Não

**C)** Teve grupo controle?

( ) Sim ( ) Não

**O)** O estudo apresenta valores de marcadores inflamatórios (citocinas e outros marcadores) antes e após as intervenções de creatina?

( ) Sim	( ) Não

**S)** O estudo é um ensaio clínico randomizado e controlado?

( ) Sim	( ) Não

**Artigo 567: Creatine supplementation and muscles: From metabolism to medical practice**

Vulturar, R., Jurjiu, B., Damian, M., Bojan, A., Pintilie, S.R., Jurca, C., Chis, A., Grad, S.

**P)** Os participantes possuem mais de 18 anos de idade?

( ) Sim	(X) Não

The article is a review that discusses creatine supplementation across various age groups, and explicitly mentions populations typically under 18 years old. For example, it states: "**More research is warranted to better understand the short and long-term effects and safety of creatine supplementation among adolescents or elderly**". It further notes: "**The administration of creatine supplements in adolescent populations is controversial, due to lack of clinical data and randomized controlled studies to support its safety. Creatine is still a popular dietary supplement of choice among adolescents (both in athletes and special populations)**". Additionally, it highlights: "**Among pediatric populations, a strong rationale exists for creatine supplementation in several neuromuscular and metabolic disorders**".

**I)** A intervenção utilizada no estudo é creatina? Vale qualquer formato de administração

( ) Sim	( ) Não

**C)** Teve grupo controle?

( ) Sim ( ) Não

**O)** O estudo apresenta valores de marcadores inflamatórios (citocinas e outros marcadores) antes e após as intervenções de creatina?

( ) Sim	( ) Não

**S)** O estudo é um ensaio clínico randomizado e controlado?

( ) Sim	( ) Não

**Artigo 568: Can the use of creatine supplementation attenuate muscle loss in cachexia and wasting?**

Sakkas, G.K., Schambelan, M., Mulligan, K.

**P)** Os participantes possuem mais de 18 anos de idade?

( ) Sim	(X) Não

The review article synthesizes findings from multiple studies. Table 1, which summarizes these studies, includes an entry under "Population (N)" that states: "**Children with Duchenne muscular dystrophy (N=30)**" for Tarnopolsky, 2004.

**I)** A intervenção utilizada no estudo é creatina? Vale qualquer formato de administração

( ) Sim	( ) Não

**C)** Teve grupo controle?

( ) Sim ( ) Não

**O)** O estudo apresenta valores de marcadores inflamatórios (citocinas e outros marcadores) antes e após as intervenções de creatina?

( ) Sim	( ) Não

**S)** O estudo é um ensaio clínico randomizado e controlado?

( ) Sim	( ) Não

**Artigo 569: Creatine supplementation and endurance performance: surges and sprints to win the race**

Forbes, S.C., Candow, D.G., Neto, J.H.F., Kennedy, M.D., Forbes, J.L., Machado, M., Bustillo, E., Gomez-Lopez, J., Zapata, A., Antonio, J.

**P)** Os participantes possuem mais de 18 anos de idade?

( ) Sim	(X) Não

The article states its purpose as a "narrative" review: "Thus, the purpose of this narrative is to: (1) identify potential mechanisms whereby creatine may influence endurance performance... (2) evaluate the efficacy of creatine supplementation on endurance performance, (3) to discuss limitations in the current evidence that warrants further investigations, and (4) provide practical recommendations for the use of creatine supplementation as an ergogenic aid for those involved in endurance events".

**I)** A intervenção utilizada no estudo é creatina? Vale qualquer formato de administração

( ) Sim	( ) Não

**C)** Teve grupo controle?

( ) Sim ( ) Não

**O)** O estudo apresenta valores de marcadores inflamatórios (citocinas e outros marcadores) antes e após as intervenções de creatina?

( ) Sim	( ) Não

**S)** O estudo é um ensaio clínico randomizado e controlado?

( ) Sim	( ) Não

**Artigo 570: Effects of creatine supplementation on nociception in young male and female** mice

Izurieta Munoz, H., Gonzales, E.B., Sumien, N.

**P)** Os participantes possuem mais de 18 anos de idade?

( ) Sim	(X) Não

The study explicitly states under "Materials and methods – Animals" that "Ninety-five **2–3 months old male and female C57BL/6J mice** were obtained from Jackson Laboratories".

**I)** A intervenção utilizada no estudo é creatina? Vale qualquer formato de administração

( ) Sim	( ) Não

**C)** Teve grupo controle?

( ) Sim ( ) Não

**O)** O estudo apresenta valores de marcadores inflamatórios (citocinas e outros marcadores) antes e após as intervenções de creatina?

( ) Sim	( ) Não

**S)** O estudo é um ensaio clínico randomizado e controlado?

( ) Sim	( ) Não

**Artigo 571: Sarcopenia: Current theories and the potential beneficial effect of creatine application strategies**

Candow, D.G.

**P)** Os participantes possuem mais de 18 anos de idade?

( ) Sim	(X) Não

The article is explicitly identified as a "**REVIEW ARTICLE**". As a review, it does not involve direct participants or data collection from individuals within this specific publication. Instead, it synthesizes and discusses existing literature on sarcopenia, which is defined as "the age-related loss of muscle mass", and the "potential beneficial effect of creatine application strategies" in this context, often referring to studies involving "**older adults**".

**I)** A intervenção utilizada no estudo é creatina? Vale qualquer formato de administração

( ) Sim	( ) Não

**C)** Teve grupo controle?

( ) Sim ( ) Não

**O)** O estudo apresenta valores de marcadores inflamatórios (citocinas e outros marcadores) antes e após as intervenções de creatina?

( ) Sim	( ) Não

**S)** O estudo é um ensaio clínico randomizado e controlado?

( ) Sim	( ) Não

**Artigo 572: Intradialytic creatine supplementation: A scientific rationale for improving the health and quality of life of dialysis patients**

Wallimann, T., Riek, U., Möddel, M.

**P)** Os participantes possuem mais de 18 anos de idade?

( ) Sim	(X) Não

The article is clearly defined as a "**Medical Hypotheses**" and a "**scientific rationale**" paper. It "propose[s] intradialytic Cr supplementation" and explicitly states that "investigations on intradialytic Cr supplementation **have not been performed**".

**I)** A intervenção utilizada no estudo é creatina? Vale qualquer formato de administração

( ) Sim	( ) Não

**C)** Teve grupo controle?

( ) Sim ( ) Não

**O)** O estudo apresenta valores de marcadores inflamatórios (citocinas e outros marcadores) antes e após as intervenções de creatina?

( ) Sim	( ) Não

**S)** O estudo é um ensaio clínico randomizado e controlado?

( ) Sim	( ) Não

**Artigo 573: Creatine supplementation and the role on oxidative stress, brain creatine level and inflammation. A brief review**

Moscatelli, F., Villano, I., Monda, M., Messina, A., Monda, V., Cibelli, G., Anna, V., Tartaglia, N., Ambrosi, A., Porro, C., Messina, G.

**P)** Os participantes possuem mais de 18 anos de idade?

( ) Sim	(X) Não

The article is explicitly titled and described as a "**Review**" and a "**brief review**", meaning it synthesizes existing literature rather than conducting a new study with direct participants. The abstract further clarifies the scope of the reviewed literature, stating that creatine supplementation "is safe and well-tolerated in healthy individuals and in a number of patient populations ranging from **infants to the elderly**".

**I)** A intervenção utilizada no estudo é creatina? Vale qualquer formato de administração

( ) Sim	( ) Não

**C)** Teve grupo controle?

( ) Sim ( ) Não

**O)** O estudo apresenta valores de marcadores inflamatórios (citocinas e outros marcadores) antes e após as intervenções de creatina?

( ) Sim	( ) Não

**S)** O estudo é um ensaio clínico randomizado e controlado?

( ) Sim	( ) Não

**Artigo 574: An update on the neuroprotective effects of creatine in central nervous system diseases**

Cunha, M.P.

**P)** Os participantes possuem mais de 18 anos de idade?

( ) Sim	( ) Não

**I)** A intervenção utilizada no estudo é creatina? Vale qualquer formato de administração

( ) Sim	(	) Não

**C)** Teve grupo controle?

(	) Sim ( ) Não

**O)** O estudo apresenta valores de marcadores inflamatórios (citocinas e outros marcadores) antes e após as intervenções de creatina?

( ) Sim	(	) Não

**S)** O estudo é um ensaio clínico randomizado e controlado?

(	) Sim	( ) Não

**Artigo 575: Integrative analysis revealed novel putative therapeutic targets of ulcerative colitis: Role of creatine**

Zhu, J., Qian, X., Mu, J., Wu, X., Wan, L., Zhang, Z., Li, L.

**P)** Os participantes possuem mais de 18 anos de idade?

( ) Sim	(X) Não

The "Materials and methods" section explicitly describes the use of animal participants in the study: "After 7 days of adaptation breeding, sixteen **mice** were randomly allocated into two groups: DSS, DSS+creatine group".

**I)** A intervenção utilizada no estudo é creatina? Vale qualquer formato de administração

( ) Sim	( ) Não

**C)** Teve grupo controle?

( ) Sim ( ) Não

**O)** O estudo apresenta valores de marcadores inflamatórios (citocinas e outros marcadores) antes e após as intervenções de creatina?

( ) Sim	( ) Não

**S)** O estudo é um ensaio clínico randomizado e controlado?

( ) Sim	( ) Não

**Artigo 576: Antenatal creatine supplementation reduces persistent fetal lung inflammation and oxidative stress in an ovine model of chorioamnionitis**

Choi, Y.J., Williams, E., Dahl, M.J., Amos, S.E., James, C., Bautista, A.P., Kurup, V., Musk, G.C., Kershaw, H., Arthur, P.G., Kicic, A., Choi, Y.S., Terrill, J.R., Pillow, J.J.

**P)** Os participantes possuem mais de 18 anos de idade?

( ) Sim	( ) Não

**I)** A intervenção utilizada no estudo é creatina? Vale qualquer formato de administração

( ) Sim	(	) Não

**C)** Teve grupo controle?

(	) Sim ( ) Não

**O)** O estudo apresenta valores de marcadores inflamatórios (citocinas e outros marcadores) antes e após as intervenções de creatina?

( ) Sim	(	) Não

**S)** O estudo é um ensaio clínico randomizado e controlado?

(	) Sim	( ) Não

**Artigo 577: Creatine in the fetal brain: A regional investigation of acute global hypoxia and creatine supplementation in a translational fetal sheep model**

Tran, N.T., Muccini, A.M., Hale, N., Tolcos, M., Snow, R.J., Walker, D.W., Ellery, S.J.

**P)** Os participantes possuem mais de 18 anos de idade?

( ) Sim	(X) Não

The title of the article clearly indicates the study subject: "**Creatine in the fetal brain: A regional investigation of acute global hypoxia and creatine supplementation in a translational fetal sheep model**". The "Methods" section explicitly states that "Near-term **fetal sheep** were administered continuous intravenous infusion of creatine" and "Fetuses were randomly assigned to one of the four treatment groups".

**I)** A intervenção utilizada no estudo é creatina? Vale qualquer formato de administração

( ) Sim	( ) Não

**C)** Teve grupo controle?

( ) Sim ( ) Não

**O)** O estudo apresenta valores de marcadores inflamatórios (citocinas e outros marcadores) antes e após as intervenções de creatina?

( ) Sim	( ) Não

**S)** O estudo é um ensaio clínico randomizado e controlado?

( ) Sim	( ) Não
