# Supplementary material for: Impact of creatine supplementation on inflammation: evidence from a systematic review and meta-analysis of randomized double-blind placebo trials
Source: Front Immunol. 2026 Feb 19;17:1743603. doi: 10.3389/fimmu.2026.1743603 (PMC12961398; doi:10.3389/fimmu.2026.1743603)
Supplement: Supplementary file 2 [file SupplementaryFile1.zip › SR Creatine inflammatory markers (Kell Doutorado). /Para submeter/Modelo cover letter.doc]

Dear Dr.,

Editor

In accordance to the rules for application, find enclosed the research article entitled “Autonomic heart rate regulation in children with language disorder: pilot study”, by Elaine Osório, Célia M. Giacheti, Ariany G. Silva, Marcela L. Nogueira, David M. Garner and Vitor E. Valenti to submission to Integrative Psychological and Behavioral Science. We had no prior interaction with Integrative Psychological and Behavioral Science regarding the submitted manuscript and suggest no opposed reviewer.

All authors declare that the manuscript is original and that the text has not been nor will be submitted for publication in any other journal, the manuscript has been approved by all authors, who agree to cede the copyrights to the Journal, disclose all sources of funding and declare all potential conflicts of interest, the study was endorsed by an Internal Review Board (Ethics Committee) (No. CEP-1022/2014, May 25^th^ 2014).

The authors also accept full responsibility for the accuracy of all content, release and assign all rights for the publication of the manuscript to Integrative Psychological and Behavioral Science and state that there are no conflicts of interest related to the research or the manuscript. Authorship has been granted to those individuals who have contributed substantially to the research or manuscript.

As you know XXXXXXXXX.

We have also published several important reports in this field (REFERENCES)

Among the methodological relevance of our study that makes the difference we include: 1) data collection in a laboratory condition, since we aimed to control humidity, ambient and body temperature. A real ambient would influence those variables; 2) Maximal oxygen consumption was analyzed through direct expired gases analysis; 3) The crossover and randomized design helps to improve reproducibility and physiological interpretation of our data.

In our study, we show that although linear behavior of vagal heart rate control was unaltered in the children with language disorder investigated in our study, there was decreased complexity of heart rate dynamics, suggesting that children with language disorder may have impaired autonomic regulation of heart rate.

We decided to submit our manuscript to Integrative Psychological and Behavioral Science because it is an important journal, which help us to share our findings. Moreover, Integrative Psychological and Behavioral Science has a broad coverage of different subject areas. We believe that it will be of great interest to your readers and hope to contribute to this important journal.

The author’s contributions are listed below:

Elaine Osório: collected data, performed conduction of experiments and draft the manuscript.

Célia M. Giacheti: draft the manuscript, followed the journal guidelines and reviewed statistical analysis.

Ariany G. Silva: collected data and performed conduction of experiments.

Marcela L. Nogueira: collected data and performed conduction of experiments.

David M. Garner performed statistical analysis, draft the manuscript, extensively reviewed the manuscript, English Grammar and Spelling.

Vitor E. Valenti supervised the study, draft the manuscript, wrote introduction and discussion section and gave final approval for the version submitted for publication.

We suggest the following reviewers:

Moacir Godoy: Faculdade de Medicina de São José do Rio Preto. E-mail: [mf60204@gmail.com](mailto:mf60204@gmail.com)

Carlos B. M. Monteiro: USP, E-mail: [carlosfisi@uol.com.br](mailto:carlosfisi@uol.com.br)

Luiz Carlos M. Vanderlei. UNESP, Presidente Prudente. E-mail: [lcmvanderlei@fct.unesp.br](mailto:lcmvanderlei@fct.unesp.br)

Marek Sykora: Dept. of Neurology, University of Heidelberg, Heidelberg, Germany. E-mail: [marek.sykora@med.sfu.ac.at](mailto:marek.sykora@med.sfu.ac.at)

Rajesh Goit: Department of Physiology, Nepalgunj Medical College, Nepal. Electronic address: [goit_rajesh@yahoo.com](mailto:goit_rajesh@yahoo.com)

The authors declare that the manuscript has been approved by all authors, who agree to cede the copyrights to the Journal, disclose all sources of funding and declare all potential conflicts of interest.

With our best wishes,

Elaine Osório

Célia M. Giacheti

Ariany G. Silva

Marcela L. Nogueira

David M. Garner

Vitor E. Valenti
