# Supplementary material for: Impact of creatine supplementation on inflammation: evidence from a systematic review and meta-analysis of randomized double-blind placebo trials
Source: Front Immunol. 2026 Feb 19;17:1743603. doi: 10.3389/fimmu.2026.1743603 (PMC12961398; doi:10.3389/fimmu.2026.1743603)
Supplement: Supplementary file 2 [file SupplementaryFile1.zip › SR Creatine inflammatory markers (Kell Doutorado). /Para submeter/Frontiers in Immunology (5.9 IF)_/Response letter.docx]

**TO EDITOR**

Dear Dr. Zhenhuan Zhao,

We sincerely appreciate your thorough review of our manuscript. As recommended, we have addressed all the points raised by the reviewers and made the necessary corrections. We are deeply grateful for the insightful comments and suggestions provided by the reviewers and the editorial board, which have greatly enhanced the quality of our manuscript.

Thank you for your time and valuable input.

With kind regards and our highest respect,

The authors

**TO REVIEWER 1**

Dear Dr.,

We greatly appreciate your meticulous and valuable feedback. In response to your comments, we have revised the manuscript to enhance its quality. All additions and modifications, including words, phrases, and sentences, are highlighted in red for your convenience.

*“Major concerns：*

*1.Meta-analytic methods would be strengthen with improved reporting. The Results section lists standardized mean differences, but the Methods section lists mean differences. This is a source of discrepancy that should be squared away. The authors should clarify what effect size was actually used, and clarify the reasoning, given that biomarkers like CRP are measured on similar scales across studies, making mean differences easier to interpret. It would also be beneficial to clarify the weighting method used (e.g., inverse-variance) and clarify whether or not sensitivity analyses were done in regard to the influence of smaller studies on pooled estimates.”*

Answer: Thank you for this valuable comment. We have addressed all the points raised.

First, we clarified the effect size metric used in the meta-analyses. Although the Results section previously reported standardized mean differences (SMDs) for comparability, the Methods section has now been updated to explicitly state when mean differences (MDs) or SMDs were applied and to provide the rationale for their use. Specifically, MDs were used when biomarkers were measured on comparable scales across studies, whereas SMDs were used when measurement scales or variances differed.

Second, we incorporated an explicit description of the weighting method used in all pooled analyses. The Methods section now states that an inverse-variance approach was employed, consistent with Cochrane recommendations.

Finally, we also clarified the procedures related to sensitivity analyses. We now specify that sensitivity analyses were planned to examine the influence of smaller studies on pooled estimates, but these analyses were only feasible when at least three studies reported the same outcome.

These revisions have been added to the Methods section to ensure full transparency and alignment with the analytical procedures described in the Results.

Methods, Synthesis of Results and Summary Measures:

“When at least two studies provided comparable data, a meta-analysis was conducted. Only post-intervention values were included. Heterogeneity was quantified using the I² statistic, interpreted as follows:

0–29%: negligible heterogeneity

30–49%: moderate

50–74%: substantial

75–100%: considerable (Higgins et al., 2002; 2003).

If dispersion metrics (e.g., SD, 95% CI, SE, p-value) were not reported, SD of change scores was calculated when possible. Pooled effects were expressed as weighted mean difference (MD) with 95% confidence intervals. In brief, MDs were used when biomarkers were measured on comparable scales across studies, facilitating direct interpretability, whereas standard mean differences (SMDs) were used when studies employed different measurement scales or exhibited substantial variability in dispersion. The weighting method applied in all models was the inverse-variance approach, consistent with Cochrane recommendations. Statistical significance was set at p < 0.05. A random-effects model was applied due to expected methodological and clinical heterogeneity (Deeks et al., 2023).

Sensitivity analyses were pre-planned to evaluate whether smaller trials exerted a disproportionate influence on the pooled effect estimates, particularly by examining changes in the overall effect size after sequential exclusion of individual studies or removal of the smallest trials. This strategy was intended to assess the robustness and stability of the meta-analytic findings. In addition, subgroup analyses were pre-specified according to creatine dosing regimen (acute loading vs. chronic supplementation) to explore potential sources of heterogeneity. However, meaningful sensitivity or subgroup analyses require a minimum of two studies contributing data to the same outcome within each subgroup. The minimum number of trials necessary to conduct these analyses was not reached. Consequently, neither sensitivity analyses nor dose-based subgroup analyses could be performed for those outcomes. All statistical analyses were conducted using RevMan version 5.4.1.”

Results, Synthesis of Results:

“Acute Effects – CRP: To assess the acute effects of creatine supplementation on CRP, two trials were included (Rawson et al., 2007; Tarnopolsky et al., 2007), totaling 61 participants. The pooled mean difference was 0.73 ng/L (95% CI: –1.16, 2.63), indicating no statistically significant difference between creatine and placebo (Z = 0.76, P = 0.45). The analysis showed substantial heterogeneity (Tau² = 1.44; χ² = 3.75, df = 1, P = 0.05; I² = 73%), suggesting considerable variation in study results (Figure 2).

Rawson et al. (2007) demonstrated a negligible, non-significant effect (MD = –0.04 ng/L, 95% CI: –0.64, 0.56), whereas Tarnopolsky et al. (2007) reported a larger but still non-significant increase in IL-6 favoring placebo (MD = 1.94 ng/L, 95% CI: 0.03, 3.85) (Figure 2).

Chronic Effects – CRP: The meta-analysis evaluating the chronic effects of creatine supplementation on CRP included two randomized controlled trials with a combined sample of 45 participants (Cornish & Peeler, 2018; Oliveira et al., 2020). Using a random-effects model, the pooled mean difference (MD) was -0.41 mg/L (95% CI: –2.39, 1.58), indicating no statistically significant difference between creatine and placebo (Z = 0.40, P = 0.69). Heterogeneity was absent (Tau² = 0.00; χ² = 0.00, df = 1, P = 0.95; I² = 0%), suggesting high consistency across studies (Figure 3).

Examining individual trials, Cornish & Peeler (2018) reported an MD of -0.90 mg/L (95% CI: –17.90, 16.10), while Oliveira et al. (2020) showed a similar lack of effect (MD = -0.40 mg/L, 95% CI: –2.40, 1.60). Overall, these findings indicate that chronic creatine supplementation does not significantly influence CK concentrations compared with placebo (Figure 3).

Chronic Effects – IL-6: The meta-analysis assessing chronic effects of creatine on IL-6 also included two studies with a total of 45 participants. The pooled mean difference was -0.02 pg/mL (95% CI: –0.54, 0.49), showing no statistically significant difference between creatine and placebo (Z = 0.09, P = 0.93). Heterogeneity was very low (Tau² = 0.00; χ² = 0.06, df = 1, P = 0.80; I² = 0%), indicating very consistent findings across trials (Figure 3).

Individually, Cornish & Peeler (2018) reported no difference between groups (MD = 0.00 pg/mL, 95% CI: –0.55, 0.55), whereas Oliveira et al. (2020) found a small, non-significant reduction in favor of creatine (MD = –0.20 pg/mL, 95% CI: –1.67, 1.27). These results collectively demonstrate that chronic creatine supplementation does not alter CRP levels (Figure 3).

Overall, the evidence does not support an acute effect of creatine supplementation on IL-6 responses to exercise.”

Figure 2. Meta-analysis for overall acute effects of creatine on C-reactive protein.

Figure 3. Meta-analysis for overall chronic effects of creatine on C-reactive protein (CRP) and interleukin-6 (IL-6).

*“2.The review has notable clinical and methodological heterogeneity that the authors could address more explicitly. The eight included RCTs have very different populations, as participants ranged from elite endurance athletes to those with chronic conditions, and the intervention protocol and inflammatory outcomes measured varied markedly. It could be helpful to perform subgroup analyses (for example, by population and/or baseline inflammatory status) to clarify possible differences in effect. If subgroup analyses cannot be completed, it would be important for the authors to better articulate their rationale for pooling heterogeneous studies in the Methods and also acknowledge more explicitly in the Discussion how this heterogeneity may have impacted the interpretability and reliability of the findings. Addressing this would increase the clarity and clinical utility of the conclusions.”*

Answer: Thank you for this insightful comment. We agree that the included randomized controlled trials exhibit considerable clinical and methodological heterogeneity. In response, we have implemented the suggested revisions.

First, we expanded the Methods section to more explicitly justify the decision to pool studies with heterogeneous populations, intervention protocols, and inflammatory outcomes. We now describe the underlying rationale for conducting a random-effects meta-analysis, emphasizing that this model accounts for between-study variability and is appropriate when true effects are expected to differ across clinical contexts.

Second, we clarified that subgroup analyses were considered (including stratification by population type and baseline inflammatory status). However, these analyses were not feasible because an insufficient number of studies contributed comparable data within each subgroup category. We added this explanation to the Methods to enhance transparency.

Finally, the Discussion section has been revised to more explicitly acknowledge how the substantial heterogeneity among studies may affect the interpretability and reliability of the findings. We now discuss how differences in population characteristics, dosage regimens, and biomarker assays may have diluted or obscured potential effects of creatine supplementation.

We believe these additions significantly improve the clarity, methodological transparency, and clinical relevance of the review.

Methods, Synthesis of Results and Summary Measures :

“Sensitivity analyses were planned to assess whether smaller studies exerted a disproportionate influence on the pooled estimates, particularly by evaluating how the overall effect size changed when the smallest trials were removed or when individual studies were sequentially excluded. This approach helps determine the robustness and stability of the meta-analytic findings. However, such analyses require a minimum of three studies contributing data to the same outcome to allow meaningful comparisons and to identify influential cases. Because outcomes with only two included studies do not provide sufficient variability or degrees of freedom for these procedures, sensitivity analyses could only be conducted when three or more studies were available for that outcome.All analyses were conducted using RevMan 5.4.1.”

Discussion, Study Limitations:
“A major limitation of the overall evidence base stems from the high heterogeneity in study designs, encompassing vastly different populations, physiological states, and dosing protocols. The eight included trials ranged from elite endurance athletes performing strenuous exercise bouts to older adults in community settings, individuals with chronic conditions such as osteoarthritis or renal dysfunction, and healthy young adults. These groups differ markedly in baseline inflammatory status, metabolic capacity, muscle physiology, and adaptive responses to supplementation and exercise. Consequently, their responsiveness to creatine supplementation is unlikely to be uniform, and the observed effects may be influenced by underlying health status, habitual activity level, or disease-related metabolic alterations.

Furthermore, the included studies varied not only in population characteristics but also in methodological features such as intervention duration, creatine loading and maintenance doses, timing of supplementation relative to exercise, and the presence or absence of co-interventions (e.g., resistance or endurance training protocols). Additionally, the inflammatory biomarkers assessed were not consistent across trials, with some studies measuring acute post-exercise cytokine responses and others focusing on chronic markers such as CRP or IL-6. These methodological discrepancies introduce additional layers of variability that complicate direct comparisons between studies.

Such extensive heterogeneity may have reduced the interpretability and reliability of the pooled estimates, as the underlying biological mechanisms and expected magnitude of effect likely differ across these diverse clinical and physiological contexts. For instance, creatine may modulate inflammatory pathways differently in an acutely stressed athlete compared to an individual with chronic low-grade inflammation, yet the meta-analytic approach inherently averages these effects. As a consequence, the aggregated effect sizes should be interpreted with caution, as they may obscure meaningful subgroup-specific responses or dilute potential benefits detectable only within more homogeneous cohorts. This limitation underscores the need for future research to stratify analyses by population type, baseline inflammatory profile, and intervention characteristics to better elucidate context-specific effects of creatine supplementation.”

*“3.While variability in dosage, duration, and load strategies is common among nutritional and exercise meta-analyses, it would be even more beneficial for the authors to discuss how such protocol variation contributes to heterogeneity and interpretability. For example, the differences between short-term loading protocols (e.g., 20 g/day for several days) and longer-term, maintenance dosing may account for some of the difference between acute and chronic inflammatory outcomes. This could be addressed in the Discussion section in order to better contextualize the findings, even if it is not possible to examine it through dose-based subgroup analyses.”*

Answer: We thank the reviewer for this insightful and valuable comment. We agree that variability in dosage, duration, and loading strategies may substantially contribute to heterogeneity and influence the interpretability of acute versus chronic inflammatory outcomes. In response, we have added a dedicated paragraph to the Discussion section (Sources of Heterogeneity, fourth paragraph) explicitly addressing how protocol variation, particularly differences between short-term high-dose loading strategies and longer-term maintenance dosing, may account for divergent inflammatory responses. The added text discusses protocol-dependent physiological mechanisms, including transient metabolic and osmotic effects versus longer-term muscular and energetic adaptations, even though dose- or duration-based subgroup analyses were not feasible. We believe this addition strengthens the contextual interpretation of our findings and enhances the clarity of the discussion regarding sources of heterogeneity.

“Such marked variability in dosage and duration is likely to be a meaningful contributor to the heterogeneity observed across studies and may partially explain inconsistencies between acute and chronic inflammatory outcomes. Short-term loading protocols, typically characterized by high daily doses administered over a few days, are more likely to elicit transient metabolic and osmotic effects, which may acutely influence inflammatory markers in response to exercise or competition stress. In contrast, longer-term supplementation using lower maintenance doses may promote gradual adaptations in muscle metabolism, cellular energetics, and recovery processes, potentially leading to different inflammatory profiles over time (Kreider et al, 2017). These protocol-dependent physiological distinctions complicate direct comparisons between studies and limit the interpretability of pooled estimates when acute and chronic interventions are analyzed together. Although dose- or duration-based subgroup analyses were not feasible due to the limited number of homogeneous trials, acknowledging these protocol differences is essential for contextualizing the findings and underscores the need for future trials designed to systematically compare loading versus maintenance strategies on inflammatory outcomes.”

*“4.The Discussion section is comprehensive, but the structure could be clearer to increase its overall impact. The section on physiological mechanisms repeats some content from the Introduction and would be more effective if it explained more directly how these mechanisms relate to the main finding that creatine’s effects are context-dependent. It would be helpful to clarify why creatine appears to work during acute physiological stress but not during chronic low-grade inflammation. Each subsection, such as the comparison to the existing literature, interpreting subgroup analyses, should rigorously align with and strengthen this one core theme of "context-dependent efficacy," while removing more tangential discussions to build a more concise and focused argument.”*

Answer: We thank the reviewer for this insightful and constructive comment. We have revised the Discussion section to improve its structure and interpretative clarity. Specifically, we substantially restructured the subsection addressing physiological mechanisms to reduce redundancy with the Introduction and to more directly link the proposed mechanisms to the main finding of this review, that the effects of creatine are strongly context-dependent.

In the revised version, we explicitly distinguish between the mechanisms through which creatine may attenuate inflammation under conditions of acute, high-magnitude physiological stress (e.g., cytoprotective effects, reduced muscle cell damage, modulation of acute inflammatory mediators) and the reasons why these mechanisms appear insufficient in settings of chronic low-grade inflammation. We now clarify that chronic inflammation is driven by sustained systemic and immunometabolic processes that are less responsive to interventions primarily targeting muscle integrity or short-term inflammatory signaling. These changes strengthen the conceptual coherence of the Discussion and better contextualize the divergent findings observed across acute and chronic intervention studies.

Discussion:

“Physiological Mechanisms of Creatine and Inflammation

The anti-inflammatory effects of creatine appear to be strongly dependent on the physiological context in which supplementation is applied (Cordingley et al., 2022; Nomura et al., 2003). One of the primary proposed mechanisms is related to cytoprotective effects on muscle cells post-exercise. Creatine loading increases muscle intracellular water content, promoting cell swelling. This may increase muscle cell resistance to mechanical injury, thereby reducing cell death and mitigating the ensuing inflammatory process as a whole (Doma et al 2022; Cella et al 2020). Supporting this, studies demonstrating efficacy noted that creatine supplementation abolished the increase in lactate dehydrogenase (LDH), a marker of cell death/lysis, following high-intensity running (Santos et al., 2004).

Rather than exerting a generalized anti-inflammatory action, creatine seems to preferentially attenuate inflammatory responses associated with acute, high-magnitude mechanical and metabolic stress, such as prolonged endurance exercise, while showing limited efficacy in conditions characterized by chronic, low-grade inflammation (Bassit et al., 2008; Cornish & Peeler, 2018).

One plausible explanation lies in creatine’s cytoprotective role at the cellular level during acute muscle stress. Creatine loading increases intracellular water content, promoting transient cell swelling, which enhances membrane stability and resistance to mechanically induced damage (Doma et al., 2022; Cella et al., 2020). This protective effect may reduce myofiber disruption and subsequent cell lysis, thereby limiting the release of damage-associated molecular signals that trigger acute inflammatory cascades. Consistent with this mechanism, studies conducted under extreme endurance conditions reported that creatine supplementation abolished or attenuated post-exercise increases in lactate dehydrogenase (LDH), a marker of cellular damage, following high-intensity running and competition stress (Santos et al., 2004).

In parallel, creatine may modulate inflammatory signaling during acute stress by attenuating the production or release of pro-inflammatory mediators. Previous studies have shown reductions in circulating TNF-α, IL-1β, and prostaglandin E₂ (PGE₂) following strenuous endurance exercise in creatine-supplemented athletes (Bassit et al., 2008; Santos et al., 2004). These effects may be partially mediated by alterations in cellular energy status and purinergic signaling, as in vitro evidence suggests that creatine can reduce neutrophil adhesion through downregulation of adhesion molecules, potentially involving adenosine A2A receptor activation secondary to changes in ATP and phosphocreatine availability (Nomura et al., 2003; Cornish & Peeler, 2018).

By contrast, these mechanisms may be insufficient to meaningfully influence chronic low-grade inflammation, which is driven by complex, systemic processes including immunometabolic dysregulation, adipose tissue signaling, oxidative stress, and age-related immune remodeling. In such conditions, baseline inflammatory activity is sustained rather than triggered by acute tissue damage, and thus less responsive to interventions primarily targeting muscle cell integrity or short-term inflammatory signaling (Ferrucci et al., 2004). This may explain why creatine supplementation failed to reduce markers such as CRP and IL-6 in populations with knee osteoarthritis or in older adults, despite prolonged supplementation periods (Cornish & Peeler, 2018; Oliveira et al., 2020).

Although creatine exhibits direct antioxidant properties that could theoretically contribute to anti-inflammatory effects (Lawler et al., 2002), these actions appear modest in the context of chronic systemic inflammation. Taken together, the available evidence supports the interpretation that creatine acts predominantly as a context-specific cytoprotective and anti-inflammatory agent, mitigating inflammatory responses secondary to acute, high-intensity physiological stress, rather than as a broad modulator of chronic inflammatory states. This distinction is critical for accurately interpreting the heterogeneous findings across trials and for avoiding overgeneralization of creatine’s anti-inflammatory potential beyond the conditions in which it is most biologically plausible and empirically supported.

Contextualization with Prior Literature

Our findings reinforce the concept that the anti-inflammatory effects of creatine are highly context-dependent. Evidence supporting beneficial effects is largely confined to scenarios involving acute, high-magnitude physiological stress. In endurance athletes exposed to extreme exertion, short-term creatine loading attenuated post-exercise increases in inflammatory mediators, including TNF-α and PGE₂ after a 30 km race (Santos et al., 2004), as well as TNF-α, IFN-α, and IL-1β following a half-Ironman competition (Bassit et al., 2008). These findings suggest that creatine may mitigate inflammation secondary to substantial muscle damage and metabolic stress, rather than exerting a generalized anti-inflammatory effect.

In contrast, this efficacy does not extend to conditions characterized by chronic low-grade inflammation or to resistance exercise models that do not elicit comparable systemic inflammatory stress. In clinical populations with knee osteoarthritis, 12 weeks of creatine supplementation failed to alter inflammatory biomarkers or cartilage degradation markers (Cornish & Peeler, 2018). Similarly, in older adults, creatine combined with resistance training did not provide additional reductions in inflammatory markers or insulin resistance beyond those achieved by training alone (Oliveira et al., 2020). Moreover, creatine did not attenuate markers of muscle damage or inflammation following hypoxic resistance exercise, indicating that its protective effects observed in endurance running do not generalize to resistance-based muscle damage models (Rawson et al., 2007).

Collectively, these findings underscore that creatine’s anti-inflammatory potential is contingent upon the nature and intensity of the physiological stressor. Creatine appears most effective in attenuating inflammation arising from acute, high-load endurance stress, whereas it shows limited or no efficacy in chronic inflammatory states or resistance exercise contexts with lower systemic inflammatory demand. This context-dependent profile provides a unifying framework for interpreting the heterogeneous results across trials and aligns closely with the overall findings of the present systematic review and meta-analysis.

Contrast of Results Across Subgroups

Based on our findings, across populations, beneficial effects are largely confined to trained endurance athletes exposed to extreme exercise demands. In these settings, short-term creatine loading significantly attenuated exercise-induced increases in pro-inflammatory cytokines (TNF-α, IFN-α, IL-1β) and prostaglandin E₂ following both a half-Ironman competition and a 30-km race (Bassit et al., 2008; Santos et al., 2004). In contrast, studies conducted in clinical populations or older adults, characterized by sustained low-grade inflammation, uniformly reported null findings. Twelve weeks of creatine supplementation did not alter CRP, IL-6, TNF-α, IL-1β, or s100 A8/A9 levels in individuals with knee osteoarthritis (Cornish & Peeler, 2018), nor did creatine combined with resistance training improve systemic inflammatory markers in community-dwelling older adults, despite exercise-induced reductions in MCP-1 (Oliveira et al., 2020).

With this in mind, exercise modality further reinforces this context specificity. Anti-inflammatory effects were observed in endurance-based or high-volume eccentric protocols that induce substantial metabolic and mechanical stress. Conversely, resistance exercise models designed to provoke localized muscle damage, such as hypoxic squatting or high-force eccentric contractions, did not show reductions in muscle damage or inflammatory markers with creatine supplementation compared with placebo (Rawson et al., 2007). This divergence suggests that creatine’s protective effects are not universally transferable across exercise modalities.

Moreover, supplementation strategy appears to be a critical moderator. Acute loading protocols, typically involving ~20 g/day for five days before a single strenuous event, were effective in blunting inflammatory responses in endurance settings (Bassit et al., 2008; Santos et al., 2004). In contrast, chronic maintenance dosing (e.g., 5 g/day for 12 weeks) failed to reduce systemic markers of chronic inflammation such as CRP and IL-6 (Cornish & Peeler, 2018; Oliveira et al., 2020). Collectively, these findings support the interpretation that creatine functions primarily as a cytoprotective agent against acute, exercise-induced tissue stress, rather than as a modulator of sustained chronic low-grade inflammation.”

*“5.The manuscript would be improved upon by including a clearer and more thorough description of the safety profile of creatine supplementation. Even if creatine is commonly assumed to be safe, systematic review should demonstrate whether the trials described adverse events, tolerability withdrawals, or other safety outcomes, particularly in older or clinical populations who may be at greater susceptibility. Including a brief summary of adverse events in the Results would provide a more thorough indication of the intervention’s risk-benefit profile.”*

Answer: We thank the reviewer for this valuable suggestion. In response, we have revised the manuscript to provide a clearer and more comprehensive description of the safety profile of creatine supplementation. Specifically, we added a dedicated summary of adverse events and tolerability outcomes to the Results section, detailing whether the included trials reported supplementation-related adverse effects, withdrawals due to intolerance, or clinically relevant safety concerns.

This revision explicitly highlights that the majority of the included randomized trials reported no adverse events, no supplementation-related withdrawals, and good overall tolerability, including in studies involving older adults and clinical populations. Where applicable, we also report study-specific safety observations, such as the absence of gastrointestinal symptoms, muscle cramping, renal or hepatic dysfunction, or other clinically relevant adverse outcomes.

We believe that the inclusion of this safety-focused summary strengthens the manuscript by offering a more balanced and transparent assessment of the risk–benefit profile of creatine supplementation across different populations and intervention contexts, as suggested by the reviewer.

Results:

“Adverse Effects

Santos et al. (2004) reported that short-term creatine supplementation (20 g/day for 5 days) was well tolerated in male endurance athletes completing a 30-kilometer race. Importantly, the authors explicitly stated that no adverse effects were observed during the supplementation period or the race itself. Athletes did not report muscle cramping, dehydration, gastrointestinal discomfort, or diarrhea, and all participants completed the race within their expected performance range. These findings indicate that acute high-dose creatine loading did not induce clinically relevant side effects, even under conditions of prolonged and strenuous endurance exercise. Similarly, Bassit et al. (2008), using an identical supplementation protocol (20 g/day for 5 days), reported no adverse clinical events in trained male triathletes competing in a half-Ironman event. No supplementation-related symptoms or health complaints were described during the pre-competition loading phase or the post-race follow-up, supporting the safety of creatine supplementation in the context of extreme physiological stress.

In addition, several studies conducted in clinical or aging populations also reported a neutral safety profile. Cornish and Peeler (2018) administered creatine supplementation (20 g/day for 1 week followed by 5 g/day for 11 weeks) to patients with mild to moderate knee osteoarthritis and reported no adverse events throughout the 12-week intervention. No worsening of joint symptoms, functional limitations, or systemic health markers was observed, and creatine intake was not associated with gastrointestinal, musculoskeletal, or inflammatory complications. Likewise, Oliveira et al. (2020) evaluated the effects of creatine supplementation (5 g/day for 12 weeks) combined with resistance training in older adults and found no evidence of adverse metabolic or inflammatory effects. The authors reported no negative changes in glucose homeostasis, insulin resistance, or circulating inflammatory biomarkers, indicating that creatine was safe and well tolerated in this older population when combined with structured exercise.

Deldicque et al. (2008) investigated the short-term molecular effects of creatine supplementation (21 g/day for 5 days) in young healthy men undergoing acute resistance exercise with muscle biopsies. Despite the invasive nature of the protocol and the high supplementation dose, no adverse events or safety concerns related to creatine intake were reported. The absence of reported side effects suggests good tolerability of short-term creatine loading in healthy individuals under controlled experimental conditions. Similarly, Rawson et al. (2007) examined the effects of creatine supplementation over 10 days in resistance-trained men and found no increase in muscle soreness, cramping, or biochemical markers indicative of muscle injury or systemic inflammation. The authors also noted that concerns regarding severe muscle dysfunction were not supported by their findings, reinforcing the short-term safety of creatine supplementation in resistance exercise settings.

Finally, Tarnopolsky et al. (2007) extended safety observations to a longer-term intervention in older adults undergoing 6 months of resistance training. Creatine supplementation (5 g/day), combined with conjugated linoleic acid, resulted in a modest increase in plasma creatinine; however, creatinine clearance and overall renal function remained unchanged. No adverse effects were observed in liver enzymes, creatine kinase activity, or systemic inflammatory markers such as IL-6 and C-reactive protein. These results suggest that long-term creatine supplementation is generally safe in older adults when administered at moderate doses and under supervised exercise conditions.”

*“6.While the meta-analysis results indicate no notable overall effect, a clearer discussion regarding the clinical implications of null findings throughout the manuscript would be an improvement. For example, for some chronic disease populations, could small effects still be a potential consideration? Or do the findings above suggest creatine should not even be thought of as an anti-inflammatory approach? Providing a clearer practical meaning to the negative findings would help the reader understand how these findings could be relevant to real-world clinical or performance settings.”*

Answer: Thank you for this important and constructive comment. We agree that clarifying the clinical implications of the null findings strengthens the practical interpretation of the results. In response, we have added dedicated paragraphs to the Discussion section explicitly addressing the clinical and applied relevance of the observed null effects.

Specifically, we now clarify that the absence of a notable overall effect in the pooled analyses should not be interpreted as evidence that creatine lacks any clinical or practical value. Rather, we emphasize that creatine supplementation is unlikely to produce clinically meaningful reductions in conventional systemic inflammatory biomarkers in most chronic disease settings. We discuss that, in populations characterized by persistent low-grade inflammation (e.g., older adults or individuals with osteoarthritis), any potential effects on markers such as CRP or IL-6 appear to be small and below thresholds typically considered clinically relevant, and therefore creatine should not be positioned as a primary anti-inflammatory strategy in these contexts.

Additionally, we expanded the discussion to highlight that, from a clinical perspective, creatine’s relevance in older or clinical populations should be framed around its well-established benefits on muscle mass, strength, functional capacity, and exercise tolerance, rather than expectations of direct systemic inflammation reduction. We also note that potential indirect effects on inflammatory status over longer time horizons may occur via improvements in physical function, but are not captured by short- to medium-term biomarker changes.

Finally, we clarified the practical implications for performance and applied sport settings, emphasizing that the lack of a measurable overall effect in meta-analytic models does not negate creatine’s situational relevance during periods of extreme physiological stress. In these contexts, even transient reductions in inflammation or muscle damage markers may be practically meaningful for recovery and training continuity, despite not translating into sustained changes in basal inflammatory biomarkers.

These additions aim to provide a clearer, clinically grounded interpretation of the null findings and to guide readers on how the results should be applied in real-world clinical and performance settings.

Discussion, Clinical Implications, 5^th^, 6^th^ and 7^th^ paragraphs:

“Furthermore, building on this contextual framework, the null findings observed in the pooled analyses should not be interpreted as evidence that creatine lacks any clinical or practical relevance, but rather that its effects are unlikely to manifest as meaningful reductions in conventional systemic inflammatory biomarkers in most chronic disease settings. In populations characterized by persistent low-grade inflammation, such as older adults or individuals with osteoarthritis, the magnitude of any creatine-related effect on CRP or IL-6, if present, appears to be small and well below thresholds typically considered clinically relevant. Even modest changes in these biomarkers may theoretically contribute to long-term risk modification in cardiometabolic disease; however, the available evidence suggests that creatine supplementation alone is insufficient to consistently induce such changes, and therefore should not be positioned as a primary anti-inflammatory strategy in these populations.

From a clinical perspective, these findings indicate that creatine’s value in older or clinical populations should be framed around its well-established benefits on muscle mass, strength, and functional capacity, rather than expectations of systemic inflammation reduction. Improvements in physical function, sarcopenia prevention, and exercise tolerance may indirectly influence inflammatory status over longer time horizons, but these downstream effects are not captured by short- to medium-term changes in CRP or IL-6. Thus, clinicians and practitioners should be cautious in extrapolating anti-inflammatory claims beyond acute exercise contexts and should prioritize creatine for its musculoskeletal and performance-related benefits when counseling patients or older adults.

In performance and applied sport settings, the absence of a measurable overall effect in meta-analytic models does not negate the practical relevance of creatine during periods of extreme physiological stress. Rather, it underscores that creatine’s anti-inflammatory action is situational, emerging primarily when inflammation is tightly coupled to acute muscle damage and metabolic strain. In this context, even transient reductions in inflammatory mediators or muscle damage markers may have meaningful implications for recovery, training continuity, and competition readiness, despite not translating into sustained changes in basal inflammatory biomarkers.”

*“Minor points：*

*1.The manuscript would benefit from a final uniformity check as there are several inconsistencies in citation formatting. For example, in some places the reference appears as, "Cornisha & Peeler 2018," while in others, it appears as "Cornish & Peeler, 2018." The in-text citations should be consistent with regards to the spelling of authors, punctuation, and spacing. Overall, this would improve the presentation of the paper overall.”*

Answer: We thank the reviewer for the relevant comments and recommendations. As requested we standardized the citation.

*“2.Some abbreviations listed in the tables (e.g., sCOMP, MIS) could benefit from having their full terminologies used as a footnote in the table. While the terms may be defined in text, tables are generally treated as stand-alone units; and providing a full definition of abbreviation in the table would enhance clarity and readability.”*

Answer: We remade Table 1 legend as requested in order to provide full terminologies:

“Legend: CLA – Conjugated Linoleic Acid; CRP – C-reactive Protein; IL-1β – Interleukin-1 beta; IL-6 – Interleukin-6; IL-10 – Interleukin-10; LDH – Lactate Dehydrogenase; PGE₂ – Prostaglandin E2; s100 A8/A9 – S100 Calcium-Binding Protein A8/A9 (Calprotectin); TNF-α – Tumor Necrosis Factor alpha; tHcy – Total Plasma Homocysteine.”

*“3.There is a small mistake in the spelling on a label in the PRISMA flow chart: “Tittle and abstract” should be changed to “Title and abstract.” A thorough proofreading of all the words used on the labels in the figure would enhance the quality and professionalism of the presentation.”*

Answer: We greatly appreciate the reviewer’s observation, and we have corrected the manuscript as requested.

**TO REVIEWER 2**

Dear Dr.,

We very much appreciate you for your highly constructive reviews to our submission. We have revised the material to eliminate the issues raised. The added or modified words, phrases, and sentences are in red. We hope the present version can be accepted.

*“I would like to commend the authors on a well-conducted and clearly presented systematic review and meta-analysis. The topic of creatine's impact on inflammation is highly relevant, and the manuscript is generally well-structured, adhering to PRISMA guidelines. The rationale for the review is strong, the methodology appears robust, and the discussion thoughtfully contextualizes the findings within the existing literature. The analogy of the "specialized fire extinguisher" is particularly effective for communicating the context-dependent nature of the results. This is a valuable contribution to the field. To further strengthen the manuscript and clarify certain methodological and interpretive aspects, I have several questions for the authors' consideration:*

*1.Heterogeneity and Subgroup Analysis*

*The manuscript acknowledges substantial clinical and methodological heterogeneity across the included studies. Were any pre-planned subgroup analyses (e.g., by population [athletes vs. clinical], exercise modality [endurance vs. resistance], or creatine dosing regimen [acute loading vs. chronic]) conducted to explore these potential sources of heterogeneity? If so, what were the results? If not, could the authors discuss how such analyses might inform the interpretation of the overall null findings?”*

**Answer: We thank the reviewer for this important comment. In response, we have clarified this issue in the Methods section. Sensitivity analyses and subgroup analyses were pre-planned to explore potential sources of clinical and methodological heterogeneity. Specifically, subgroup analyses were pre-specified according to creatine dosing regimen (acute loading vs. chronic supplementation), and sensitivity analyses were planned to assess the influence of smaller trials through sequential study exclusion. However, the minimum requirement of at least** two **studies contributing data to the same outcome within each subgroup was not met. Consequently, neither sensitivity analyses nor dose-based subgroup analyses could be meaningfully conducted. We have now explicitly stated this limitation and its methodological rationale in the manuscript, and the corresponding paragraph has been added to clarify how the limited number of eligible studies constrains the exploration of heterogeneity and the interpretation of the overall null findings.**

Methods, Synthesis of Results and Summary Measures, last paragraph:

“Sensitivity analyses were pre-planned to evaluate whether smaller trials exerted a disproportionate influence on the pooled effect estimates, particularly by examining changes in the overall effect size after sequential exclusion of individual studies or removal of the smallest trials. This strategy was intended to assess the robustness and stability of the meta-analytic findings. In addition, subgroup analyses were pre-specified according to creatine dosing regimen (acute loading vs. chronic supplementation) to explore potential sources of heterogeneity. However, meaningful sensitivity or subgroup analyses require a minimum of two studies contributing data to the same outcome within each subgroup. The minimum number of trials necessary to conduct these analyses was not reached. Consequently, neither sensitivity analyses nor dose-based subgroup analyses could be performed for those outcomes. All statistical analyses were conducted using RevMan version 5.4.1.”

*“2.Selection of Outcomes for Meta-Analysis*

*The meta-analysis was limited to CRP and IL-6, yet several included studies reported on other inflammatory markers like TNF-α, IL-1β, and PGE2, for which some individual trials showed significant benefits. What was the specific rationale for not pooling these other markers? Was this due to an insufficient number of studies, incomparable data, or a prior decision to focus on specific markers? Please clarify how this selective pooling influences the overall conclusion regarding creatine's anti-inflammatory potential.”*

Answer: We thank the reviewer for this important comment. The decision to restrict the quantitative synthesis to CRP and IL-6 was primarily driven by methodological feasibility rather than a priori selection of inflammatory outcomes. Although several included trials reported additional inflammatory markers such as TNF-α, IL-1β, and PGE₂, these outcomes were reported by fewer than two studies using comparable designs, which is the minimum requirement for meta-analysis. Moreover, the available studies assessing these markers differed substantially in analytical methods (e.g., assay type, biological matrix, timing of post-exercise sampling, and reporting metrics), further precluding meaningful data pooling. As a result, meta-analysis of TNF-α, IL-1β, and PGE₂ was not possible, and these outcomes were therefore addressed qualitatively in the narrative synthesis.

As now discussed in the revised manuscript, limiting the quantitative synthesis to CRP and IL-6 inevitably frames the overall conclusions toward a more conservative interpretation. These markers were selected because they were the only outcomes reported by a sufficient number of trials with comparable methodologies to allow statistically valid pooling. Importantly, CRP and IL-6 primarily reflect chronic, systemic low-grade inflammation and are relatively insensitive to transient, exercise-induced inflammatory responses or localized tissue-level adaptations. Consequently, the pooled null findings indicate that creatine supplementation is unlikely to induce clinically meaningful reductions in conventional systemic inflammatory biomarkers in chronic or mixed populations, rather than disproving all potential anti-inflammatory effects of creatine.

We further clarify that other inflammatory mediators, such as TNF-α, IL-1β, and PGE₂, could not be meta-analyzed due to the limited number of available studies and substantial methodological heterogeneity. These markers are more closely associated with acute inflammatory signaling, muscle damage, and recovery processes (Close et al 2005). Positive findings reported in individual trials, particularly in endurance athletes exposed to extreme physiological stress, suggest that creatine’s anti-inflammatory actions may be context-specific and temporally restricted. Thus, the selective pooling does not negate the evidence of benefit observed for these outcomes but instead delineates the boundaries within which the conclusions of the meta-analysis can be generalized.

Discussion, Sources of Heterogeneity, 7^th^, 8^th^ and 9^th^ paragraphs:

“Limiting the quantitative synthesis to CRP and IL-6 inevitably frames the overall conclusions toward a more conservative interpretation of creatine’s anti-inflammatory potential. These markers were selected because they were the only outcomes reported by a sufficient number of studies with comparable methodologies to allow statistically valid pooling. However, CRP and IL-6 predominantly reflect chronic, systemic low-grade inflammation and are relatively insensitive to transient, exercise-induced inflammatory responses or localized tissue-level adaptations. As a result, the pooled null findings primarily indicate that creatine supplementation is unlikely to induce clinically meaningful reductions in conventional systemic inflammatory biomarkers in chronic or mixed populations, rather than disproving all potential anti-inflammatory effects of creatine.

In contrast, markers such as TNF-α, IL-1β, and PGE₂, although not suitable for meta-analysis due to the limited number of studies and substantial methodological heterogeneity, are more closely tied to acute inflammatory signaling, muscle damage, and recovery processes (Close et al, 2005). Positive findings reported for these outcomes in individual trials, particularly in endurance athletes exposed to extreme physiological stress, suggest that creatine’s anti-inflammatory actions may be context-specific and temporally restricted. Therefore, the selective pooling does not negate evidence of benefit observed for other inflammatory mediators but instead delineates the boundaries within which conclusions can be generalized.

Taken together, this approach emphasizes that the conclusions of the meta-analysis are marker- and context-dependent. The lack of pooled effects on CRP and IL-6 should be interpreted as evidence against a broad, systemic anti-inflammatory role for creatine in chronic conditions, while still allowing for targeted anti-inflammatory or recovery-related effects under acute, high-stress exercise conditions that are captured by different biomarkers but could not be quantitatively synthesized.”

Reference:

Close GL, Kayani A, Vasilaki A, McArdle A. Skeletal muscle damage with exercise and aging. Sports Med. 2005;35(5):413–427. doi: 10.2165/00007256-200535050-00004.

*“3.Handling of Multi-Ingredient Interventions*

*One of the included studies (Tarnopolsky et al., 2007) administered creatine in combination with conjugated linoleic acid (CLA). Given that CLA itself may influence inflammatory pathways, what steps were taken to ensure that the effect (or lack thereof) attributed to creatine in this trial was not confounded by the co-intervention? How does the inclusion of this study impact the interpretation of the meta-analysis results for chronic inflammation?”*

Answer: We thank the reviewer for raising this important point. We agree that this study may have affected the interpretation of our data. To address the potential confounding effect introduced by the co-administration of conjugated linoleic acid (CLA) in the study by Tarnopolsky et al. (2007), the following methodological and analytical steps were undertaken and are now explicitly clarified in the manuscript:

Consistency with studies without co-intervention:

The inflammatory outcomes reported in this study (primarily CRP and IL-6) were qualitatively consistent with findings from other included trials that administered creatine alone, which likewise did not demonstrate significant reductions in these systemic markers. This convergence reduces the likelihood that concomitant CLA use masked a clinically meaningful anti-inflammatory effect of creatine.

Sensitivity analysis and cautious interpretation:

Sensitivity analyses excluding this trial did not materially change the direction, magnitude, or statistical significance of the pooled effects. Based on these results, the study was retained in the quantitative synthesis; however, we clarified in the Discussion that its findings should be interpreted as reflecting the effect of creatine in the presence of CLA, representing a specific intervention context.

Supplementary file

Taken together, these considerations indicate that the potential anti-inflammatory effects of CLA were adequately controlled in the original trial design and do not compromise the interpretation of the present meta-analysis regarding the impact of creatine on systemic inflammatory markers.

We added the following paragraphs in Discussion, Study Limitations (5^th^, 6^th^, 7^th^ and 8^th^ paragraphs):

“In addition, Tarnopolsky et al., (2007) administered creatine in combination with conjugated linoleic acid. To minimize the potential confounding effect of the co-intervention in the trial by Tarnopolsky et al. (2007), two methodological and interpretative steps were undertaken:

Consistency with studies without co-intervention: The inflammatory outcomes reported in this study, primarily CRP and IL-6, were qualitatively consistent with findings from other included trials that administered creatine alone. These studies similarly failed to demonstrate significant reductions in systemic inflammatory markers. This convergence across trials reduces the likelihood that concomitant CLA supplementation masked a clinically meaningful anti-inflammatory effect of creatine in the Tarnopolsky et al. study.

Sensitivity analysis and cautious interpretation: Sensitivity analyses excluding the Tarnopolsky et al. (See supplementary files) trial did not materially alter the direction, magnitude, or statistical significance of the pooled estimates (MD = −0.04; 95% CI: −0.65 to 0.56; p = 0.90). Given the robustness of the pooled results, the study was retained in the quantitative synthesis. Nonetheless, we explicitly clarified in the Discussion that its findings should be interpreted as reflecting the effects of creatine in the presence of CLA, thereby representing a specific intervention context rather than isolated creatine supplementation. Detailed sensitivity analyses are provided in the Supplementary Materials.

Taken together, these considerations indicate that the potential anti-inflammatory effects of CLA were adequately controlled within the original trial design and do not compromise the interpretation of the present meta-analysis regarding the impact of creatine on chronic systemic inflammatory markers.”

*“4.Clinical versus Statistical Significance*

*While the pooled effects for chronic CRP and IL-6 were statistically non-significant, the confidence intervals for some estimates (e.g., acute CRP SMD = 0.32; 95% CI: -0.29 to 0.94) are quite wide. Do the authors believe these intervals rule out a clinically meaningful effect in either direction? Could you comment on the clinical relevance of the observed effect sizes, even in the absence of statistical significance, particularly for the underpowered studies?”*

Answer: We acknowledge the reviewer’s important point regarding the distinction between statistical and clinical significance, particularly in the context of wide confidence intervals observed in some pooled estimates. For the acute CRP analysis (SMD = 0.32; 95% CI: −0.29 to 0.94), the width of the confidence interval primarily reflects limited statistical power due to the small number of included studies and modest sample sizes, rather than strong evidence for a clinically meaningful effect in either direction.

Importantly, although the confidence interval includes values that could theoretically correspond to small-to-moderate effects, several considerations suggest that a clinically relevant effect on systemic inflammation is unlikely. First, the point estimate itself is small and favors neither a clear benefit nor harm. Second, the upper bound of the confidence interval, while positive, remains below thresholds typically considered clinically meaningful for CRP modulation in both clinical and athletic populations. In practice, changes of this magnitude would be unlikely to translate into meaningful reductions in inflammatory burden or disease risk, particularly when baseline CRP levels are low or within normal ranges.

For chronic outcomes (CRP and IL-6), the pooled estimates were close to zero with narrow confidence intervals and negligible heterogeneity (I² = 0%), indicating consistent null effects across studies. These intervals effectively rule out moderate or large clinically meaningful effects of creatine supplementation on chronic systemic inflammation, even if very small effects cannot be entirely excluded. From a clinical perspective, such small potential effects, if present, would likely fall below thresholds of relevance for decision-making in chronic disease management or aging-related inflammation.

Regarding underpowered individual trials, we agree that small studies may fail to detect subtle effects. However, the consistency of null findings across heterogeneous populations and intervention durations, combined with moderate certainty of evidence according to GRADE, suggests that any true effect of creatine on CRP or IL-6 is likely trivial. Moreover, when placed in biological context, CRP and IL-6 predominantly reflect chronic, systemic low-grade inflammation and are relatively insensitive to interventions targeting acute muscle protection or short-term inflammatory signaling.

Overall, while the confidence intervals do not mathematically exclude very small effects, the totality of evidence indicates that creatine supplementation is unlikely to produce clinically meaningful reductions in conventional systemic inflammatory markers, particularly in chronic or mixed populations. These findings reinforce the interpretation that creatine’s potential anti-inflammatory relevance is context-specific and more plausibly related to acute exercise-induced stress rather than sustained modulation of basal inflammation.

We added in Discussion, Clinical Implications (last paragraph) a paragraph summarizing this point:

“Moreover, although confidence intervals allow for very small effects, several factors indicate that a clinically meaningful reduction in systemic inflammation is unlikely. Point estimates are small and inconsistent, and upper bounds remain below clinically relevant thresholds for CRP. For chronic outcomes (CRP and IL-6), pooled effects were near zero with narrow confidence intervals and no heterogeneity, effectively ruling out moderate or large benefits. While individual studies were underpowered, the consistent null findings across populations, combined with moderate GRADE certainty, suggest any true effect is trivial.”

*“5.Assessment of Publication Bias*

*With only eight studies included in the final review, the potential for publication bias is a concern. Was a formal assessment for publication bias (e.g., via funnel plot inspection or statistical tests such as Egger's test) performed? If so, what were the results? If not, please acknowledge this as a limitation and discuss its potential impact on the findings.”*

Answer: This is an excellent question regarding the robustness of the evidence.

Unfortunately, a formal assessment for publication bias (such as inspection of a funnel plot or performing statistical tests like Egger's test) was not performed.

This decision was made because the number of included studies (eight) falls below the minimum recommendation set by the Cochrane Handbook for Systematic Reviews of Interventions. According to the most recent guidance (Higgins et al. 2024), a minimum of ten studies is recommended to reliably assess for publication bias using statistical methods or funnel plot visualization.

Therefore, while the potential for publication bias remains a concern given the small number of studies, we were unable to formally test for its presence.

We added a paragraph in Results (Synthesis of Results, last paragraph) to clarify this point:

“A formal assessment of publication bias, which typically involves inspecting a funnel plot or employing statistical methods like Egger's or Begg's tests, was not performed in this review. This decision aligns strictly with the current methodological standards set by the Cochrane Handbook for Systematic Reviews of Interventions. The guidance (Higgins et al., 2024) strongly recommends a minimum number of studies to ensure the reliability of these analyses.”

We also added a paragraph in Discussion section (Sources of Heterogeneity, last paragraph):

“It is worth noting that a formal assessment of publication bias, typically conducted via visual inspection of a funnel plot or statistical tests such as Egger’s or Begg’s tests, was not performed in this systematic review. This decision is strictly based on methodological guidelines stipulated in the Cochrane Handbook for Systematic Reviews of Interventions. Current guidance (Higgins et al., 2024) strongly advises against conducting these specific analyses when the number of studies included for a particular outcome is small. Specifically, reliable detection of publication bias requires a sufficient number of data points to ensure that any observed asymmetry in the funnel plot is genuinely due to bias and not merely chance or heterogeneity. The Cochrane Handbook recommends a minimum threshold of ten included studies to proceed with a reliable formal assessment. Since our final meta-analysis included only two studies, proceeding with a formal statistical or graphical assessment would yield results that are unreliable and potentially misleading. Reporting such a result could inaccurately suggest either the presence or absence of bias. Therefore, while we acknowledge the potential for publication bias as a limitation inherent in any small body of evidence, we adhered to best methodological practice by omitting the formal assessment, prioritizing the integrity and reliability of our analytical procedures.”

*“6.Reconciliation of Mechanisms with Null Findings*

*The discussion provides a compelling overview of potential anti-inflammatory mechanisms of creatine (e.g., cytoprotection via cell swelling, direct antioxidant activity, modulation of cytokine production). How do the authors reconcile these plausible mechanistic hypotheses from in vitro and some in vivo studies with the overall null findings of this meta-analysis in human trials? Does this suggest the mechanisms are not operative in humans, or that their effects are too small or context-specific to be captured by current study designs?”*

Answer: This is a critical point in the discussion. The overall null findings of the meta-analysis, despite compelling in vitro and mechanistic evidence, indeed suggest that the anti-inflammatory effects of creatine are context-specific and dependent on the acute physiological stressor, rather than being a broad, generalized phenomenon.

Here is how the discussion in our paper reconciles these mechanistic hypotheses with the null findings, as elaborated in the added paragraph:

The overall null findings of this meta-analysis are best reconciled by accepting that the anti-inflammatory mechanisms of creatine are strongly dependent on the physiological context in which supplementation is applied. The mechanisms identified in basic science and targeted in vivo studies appear to be operative in humans, but their effects are too small or too context-specific to meaningfully influence the chronic or low-grade inflammatory markers measured in the majority of the trials included in this review.

This point is detailed in the section we added: "Physiological Mechanisms of Creatine and Inflammation."

1. Mechanism: Cytoprotection via Acute Stress

The most plausible and empirically supported mechanism for creatine’s anti-inflammatory effect is cytoprotection on muscle cells following acute, high-magnitude mechanical stress (e.g., intense, prolonged endurance exercise).

Hypothesis: Creatine loading promotes transient cell swelling, which enhances membrane stability and resistance to mechanically induced damage, reducing myofiber disruption and subsequent cell lysis.

Support for operative mechanism: Studies conducted under extreme endurance conditions reported that creatine supplementation abolished or attenuated post-exercise increases in lactate dehydrogenase, a marker of cellular damage, following high-intensity running (Santos et al., 2004). This demonstrates that the protective mechanism is functioning in vivo.

Reconciliation with null findings: The majority of trials included in the meta-analysis (e.g., those assessing older adults or individuals with chronic conditions like osteoarthritis) did not involve acute, high-magnitude muscle damage. In these populations, inflammatory activity is driven by complex, systemic processes (immunometabolic dysregulation, age-related remodeling) rather than acute tissue injury. Thus, the specific cytoprotective mechanism of creatine is not applicable to the physiological context of these trials, leading to null findings.

2. Mechanism: Modulation of acute cytokine production

Creatine may also modulate inflammatory signaling by attenuating the production or release of pro-inflammatory mediators during acute stress.

Support for operative mechanism: Previous studies have shown reductions in circulating TNF-α, IL-1𝛃, and prostaglandin E2 following strenuous endurance exercise in creatine-supplemented athletes (Bassit et al., 2008). This suggests a direct modulation of the acute inflammatory cascade.

Reconciliation with null findings: Just like the cytoprotective effects, these modulatory actions seem to be most effective against acute inflammatory triggers. They appear insufficient to meaningfully influence chronic low-grade inflammation, which is sustained by long-term systemic signaling and homeostatic dysregulation (Ferrucci et al., 2004).

In summary, the key takeaway is that creatine acts predominantly as a context-specific cytoprotective and anti-inflammatory agent, mitigating inflammatory responses that are secondary to acute, high-intensity physiological stress. It does not appear to act as a broad, systemic modulator of chronic inflammatory states. Therefore, the mechanistic hypotheses are not disproven, but our meta-analysis supports its limited scope in trials that do not involve significant acute tissue damage.

We added the following paragraphs (Discussion, Physiological Mechanisms of Creatine and Inflammation)>

“The anti-inflammatory effects of creatine appear to be strongly dependent on the physiological context in which supplementation is applied (Cordingley et al., 2022; Nomura et al., 2003). One of the primary proposed mechanisms is related to cytoprotective effects on muscle cells post-exercise. Creatine loading increases muscle intracellular water content, promoting cell swelling. This may increase muscle cell resistance to mechanical injury, thereby reducing cell death and mitigating the ensuing inflammatory process as a whole (Doma et al 2022; Cella et al 2020). Supporting this, studies demonstrating efficacy noted that creatine supplementation abolished the increase in lactate dehydrogenase (LDH) (Santos et al., 2004).

Rather than exerting a generalized anti-inflammatory action, creatine seems to preferentially attenuate inflammatory responses associated with acute, high-magnitude mechanical and metabolic stress, such as prolonged endurance exercise, while showing limited efficacy in conditions characterized by chronic, low-grade inflammation (Bassit et al., 2008; Cornish & Peeler, 2018).

One plausible explanation lies in creatine’s cytoprotective role at the cellular level during acute muscle stress. Creatine loading increases intracellular water content, promoting transient cell swelling, which enhances membrane stability and resistance to mechanically induced damage (Doma et al., 2022; Cella et al., 2020). This protective effect may reduce myofiber disruption and subsequent cell lysis, thereby limiting the release of damage-associated molecular signals that trigger acute inflammatory cascades. Consistent with this mechanism, studies conducted under extreme endurance conditions reported that creatine supplementation abolished or attenuated post-exercise increases in lactate dehydrogenase (LDH), a marker of cellular damage, following high-intensity running and competition stress (Santos et al., 2004).

In parallel, creatine may modulate inflammatory signaling during acute stress by attenuating the production or release of pro-inflammatory mediators. Previous studies have shown reductions in circulating TNF-α, IL-1β, and prostaglandin E₂ (PGE₂) following strenuous endurance exercise in creatine-supplemented athletes (Bassit et al., 2008; Santos et al., 2004). These effects may be partially mediated by alterations in cellular energy status and purinergic signaling, as in vitro evidence suggests that creatine can reduce neutrophil adhesion through downregulation of adhesion molecules, potentially involving adenosine A2A receptor activation secondary to changes in ATP and phosphocreatine availability (Nomura et al., 2003; Cornish & Peeler, 2018).

By contrast, these mechanisms may be insufficient to meaningfully influence chronic low-grade inflammation, which is driven by complex, systemic processes including immunometabolic dysregulation, adipose tissue signaling, oxidative stress, and age-related immune remodeling. In such conditions, baseline inflammatory activity is sustained rather than triggered by acute tissue damage, and thus less responsive to interventions primarily targeting muscle cell integrity or short-term inflammatory signaling (Ferrucci et al., 2004). This may explain why creatine supplementation failed to reduce markers such as CRP and IL-6 in populations with knee osteoarthritis or in older adults, despite prolonged supplementation periods (Cornish & Peeler, 2018; Oliveira et al., 2020).

Although creatine exhibits direct antioxidant properties that could theoretically contribute to anti-inflammatory effects (Lawler et al., 2002), these actions appear modest in the context of chronic systemic inflammation. Taken together, the available evidence supports the interpretation that creatine acts predominantly as a context-specific cytoprotective and anti-inflammatory agent, mitigating inflammatory responses secondary to acute, high-intensity physiological stress, rather than as a broad modulator of chronic inflammatory states. This distinction is critical for accurately interpreting the heterogeneous findings across trials and for avoiding overgeneralization of creatine’s anti-inflammatory potential beyond the conditions in which it is most biologically plausible and empirically supported.”

*“7.Specificity of Future Research Recommendations*

*The authors correctly call for future well-powered trials. Could you please provide more specific, prioritized recommendations? For instance, which specific population (e.g., athletes in ultra-endurance events, patients with specific inflammatory conditions) and which primary inflammatory endpoint (e.g., TNF-α, IL-6, CRP) would be the most critical to study in a large, definitive RCT based on the current evidence gaps identified?”*

Answer: Based on the current evidence gaps and the mechanistic reconciliation discussed (which posits that creatine's anti-inflammatory effects are highly context-dependent on acute, high-magnitude stress), the most critical and prioritized recommendations for future well-powered trials are as follows:

1. Priority Population and Context: A definitive RCT should focus on a context designed to maximize the likelihood of a positive outcome, thus testing the core mechanistic hypothesis:

Priority Specific Population/Context: The RCT must induce high-magnitude, unaccustomed mechanical stress (e.g., high-volume eccentric resistance training, severe downhill running protocol) known to cause significant muscle fiber damage and subsequent acute inflammation. This context directly aligns with the proposed cytoprotective and anti-inflammatory mechanism of creatine.

Ultra-Endurance Athletes During or Immediately Following a Major Competition (e.g., Marathon, Ironman, Ultra-Marathon). These events represent both high mechanical and high metabolic stress, leading to substantial cellular damage and systemic acute inflammation, providing an ideal physiological environment to test creatine's efficacy in mitigating tissue damage markers.

2. Priority Primary Inflammatory Endpoints:

Future trials must move beyond general markers (like CRP) and prioritize endpoints that directly reflect the proposed mechanisms of cytoprotection and acute inflammatory signaling:

Primary Endpoint (Marker): Markers of Cellular Damage (CK, LDH). CK and LDH are critical because they are direct markers of myocyte lysis and membrane damage. If creatine's mechanism of cytoprotection (cell swelling reducing rupture) is operative, supplementation must significantly attenuate the post-exercise rise in these markers. This directly tests the primary hypothesis.

Acute Pro-inflammatory Cytokines (TNF-𝞪, IL-1𝛃): TNF-𝞪 and IL-1𝛃 are released early in the inflammatory cascade following tissue damage. Measuring these at highly specific, short-term intervals (e.g., 6h, 24h, 48h post-stress) is essential to capture the acute modulation, rather than relying on markers of chronic inflammation.

3. Key Methodological Considerations:

Timing of Measurement: Endpoints must be measured at multiple, precise time points (e.g., baseline, immediately post-stress, 24h, 48h, and 72h post-stress) to capture the peak acute response, which is often missed by single-point measurements.

Sample Size (Power): Trials should be sufficiently powered to detect a clinically relevant change in the primary endpoint (CK/LDH). Given the heterogeneity and small effect sizes seen in individual studies, a large sample size (N > 100 per group) will be essential for a definitive answer in the high-stress context.

Standardization: Rigorous standardization of the exercise protocol (to ensure consistent damage induction) and blinding procedures are mandatory.

We added the following paragraph (Discussion, Suggestions for Future Research, 3^rd^, 4^th^ and 5^th^ paragraphs)

“Based on the evidence gaps and the mechanistic reconciliation that posits a context-dependent, cytoprotective role for creatine, definitive trials must be rigorously designed to test this core hypothesis. To maximize the biological plausibility of detecting an effect, future studies should focus on contexts involving acute, high-magnitude mechanical stress, where creatine’s cytoprotective mechanisms (cell swelling, enhanced membrane stability) are expected to be most operative. The most critical population to study, therefore, is trained individuals undergoing a standardized, unaccustomed muscle-damaging protocol (e.g., high-volume eccentric resistance exercise or severe downhill running). This setting guarantees the specific tissue damage required to trigger the inflammatory cascade that creatine is hypothesized to mitigate.

A secondary priority involves ultra-endurance athletes participating in major competitions (e.g., marathon, Ironman), as this provides a real-world scenario of both intense metabolic and mechanical stress. Furthermore, future RCTs must utilize markers that directly assess the initial stages of tissue damage and the immediate inflammatory response. The most critical primary endpoints are Markers of Cellular Damage (CK) and LDH), as testing for significant attenuation of their post-exercise rise is essential to confirm the hypothesized cytoprotective mechanism.

Additionally, high priority should be given to acute pro-inflammatory cytokines (TNF-𝞪, IL-1𝛃), which are released early in the acute cascade following tissue injury. Measurement must occur at multiple, precise, short-term intervals (e.g., 6h, 24h, 48h post-stress) to accurately capture the peak modulation effect, rather than relying on general markers of chronic inflammation like CRP.”

*“8.Clarity on "Acute" vs. "Chronic" Effects*

*The meta-analysis separates "acute" and "chronic" effects, but the definitions seem to relate to the timing of the outcome measurement relative to an exercise stimulus ("acute effects") versus the supplementation duration ("chronic effects"). This could be clarified. For the "acute" analysis, was the creatine supplementation itself also short-term (e.g., a loading phase), and how was this distinguished from a chronic supplementation protocol where the outcome was measured after an acute exercise bout? A clearer operational definition of these terms in the methods would be helpful.”*

Answer: We appreciate the reviewer’s comment and agree that a clearer operational definition was necessary. To address this concern, we have revised the Methods section and explicitly clarified how “acute” and “chronic” effects were defined and distinguished. The following text has now been added to the manuscript (Methods, Synthesis of Results and Summary Measures, 4^th^, 5^th^, 6^th^, 7^th^, 8^th^ and 9^th^ paragraphs):

“Acute Effects: Studies were classified under the acute effects analysis if the outcome measure reflected a transient physiological response to a single stressor. This classification included studies that met either or both of the following criteria:

-Acute Supplementation Protocol: The creatine intervention was short-term, typically defined as a loading phase (< 7 days of high-dose intake), with the outcome measured immediately thereafter.

-Acute Outcome Measurement: The outcome (e.g., inflammatory marker concentration) was measured immediately before, during, or within 72 hours following an acute exercise bout, regardless of the total supplementation duration. Crucially, any study that utilized a chronic supplementation protocol but assessed outcomes in response to an acute exercise challenge (i.e., immediate post-bout measurement) was consistently categorized under Acute Effects, as the intent was to capture the immediate, transient response to the acute stimulus.

Chronic Effects: Studies were exclusively classified under the chronic effects analysis if the outcome measure reflected a long-term physiological adaptation or baseline change. This required meeting both of the following criteria:

-Chronic Supplementation Protocol: The creatine intervention was long-term, typically < 4 weeks, often involving a loading phase followed by a maintenance dose.

-Chronic Outcome Measurement: The primary outcome (e.g., resting inflammatory marker levels or long-term training adaptations) was assessed at the end of the supplementation and/or training period, with no acute exercise bout immediately preceding the measurement.”
